# Integrating Single-Cell and Spatial Transcriptomics to Uncover and Elucidate GP73-Mediated Pro-Angiogenic Regulatory Networks in Hepatocellular Carcinoma

**DOI:** 10.34133/research.0387

**Published:** 2024-06-27

**Authors:** Jiazhou Ye, Xing Gao, Xi Huang, Shilin Huang, Dandan Zeng, Wenfeng Luo, Can Zeng, Cheng Lu, Lu Lu, Hongyang Huang, Kaixiang Mo, Julu Huang, Shizhou Li, Minchao Tang, Tianzhun Wu, Rongyun Mai, Min Luo, Mingzhi Xie, Shan Wang, Yongqiang Li, Yan Lin, Rong Liang

**Affiliations:** ^1^ Department of Hepatobiliary Surgery, Guangxi Medical University Cancer Hospital, Nanning 530021, China.; ^2^ Guangxi Liver Cancer Diagnosis and Treatment Project Technology Research Center, Nanning 530021, China.; ^3^ Guangxi Key Laboratory of Basic and Translational Research for Colorectal Cancer, Nanning 530021, China.; ^4^ Department of Digestive Oncology, Guangxi Medical University Cancer Hospital, Nanning 530021, China.; ^5^ Department of Research, Guangxi Medical University Cancer Hospital, Nanning 530021, China.

## Abstract

Hepatocellular carcinoma (HCC) was characterized as being hypervascular. In the present study, we generated a single-cell spatial transcriptomic landscape of the vasculogenic etiology of HCC and illustrated overexpressed Golgi phosphoprotein 73 (GP73) HCC cells exerting cellular communication with vascular endothelial cells with high pro-angiogenesis potential via multiple receptor–ligand interactions in the process of tumor vascular development. Specifically, we uncovered an interactive GP73-mediated regulatory network coordinated with c-Myc, lactate, Janus kinase 2/signal transducer and activator of transcription 3 (JAK2/STAT3) pathway, and endoplasmic reticulum stress (ERS) signals in HCC cells and elucidated its pro-angiogenic roles in vitro and in vivo. Mechanistically, we found that GP73, the pivotal hub gene, was activated by histone lactylation and c-Myc, which stimulated the phosphorylation of downstream STAT3 by directly binding STAT3 and simultaneously enhancing glucose-regulated protein 78 (GRP78)-induced ERS. STAT3 potentiates GP73-mediated pro-angiogenic functions. Clinically, serum GP73 levels were positively correlated with HCC response to anti-angiogenic regimens and were essential for a prognostic nomogram showing good predictive performance for determining 6-month and 1-year survival in patients with HCC treated with anti-angiogenic therapy. Taken together, the aforementioned data characterized the pro-angiogenic roles and mechanisms of a GP73-mediated network and proved that GP73 is a crucial tumor angiogenesis niche gene with favorable anti-angiogenic potential in the treatment of HCC.

## Introduction

Hepatocellular carcinoma (HCC) is the sixth most common cancer and the third leading cause of cancer-related death worldwide [1]. This lethal malignancy is distinctively characterized by its hypervascularity. Neoangiogenesis is a crucial prerequisite for transportation of oxygen, blood-borne nutrients, metabolites, and energy, which favor cancer cell growth, metastasis, and the development of multidrug resistance [2,3]. Meanwhile, during the rapid expansion of tumor cells, the inefficient supplementation of nutrients and oxygen usually contributes to the abnormal activation of pro-angiogenic signals, resulting in the uncontrolled stimulation of tumor angiogenesis [4].

Hypervascular features hold the cornerstone of anti-angiogenic regimens, primarily as the inhibition of vascular endothelial growth factor and its receptor (VEGF/VEGFR) and fibroblast growth factor and its receptor (FGF/FGFR) pathways in advanced HCC, yielding uniquely high objective response rates (ORRs) [5]. However, the initial nonresponsiveness or adaptive resistance, which are usually restricted, goes beyond the antitumor improvements in clinical practice [6]. Tumor vasculature development appears to be the results of multistep reaction processes acting on interactive networks. Therefore, it is crucial that numerous efforts are made to better describe the molecular pathogenesis of neoangiogenesis and discover novel targets with anti-angiogenic potential.

Golgi phosphoprotein 73 (GP73) is a type II Golgi membrane protein that resides in the cis- and medial-Golgi cisternae. Purified GP73 cleaved with ubiquitous proprotein convertase (PC) furin was found to cycle among various membranous compartments, such as sorting endosomes and the plasma membrane [7,8]. Normally, GP73 is primarily expressed by biliary epithelial cells and is rarely detected in normal hepatocytes; however, it is strongly up-regulated in hepatocytes with chronic liver diseases and HCC [9]. In particular, elevated serum GP73 levels have been proven to be a clinically substantial early warning of diagnosed HCC and disease progression [10,11].

Emerging studies have demonstrated that GP73 is a driver oncogene that triggers intra- and intercellular signal transduction cascades, which promote the aggressiveness of HCC [12]. Ye et al. [13] reported that GP73 could directly bind and interact with epidermal growth factor receptor (EGFR) and serve as a specific cargo adaptor to assist EGFR/receptor tyrosine kinase (EGFR/RTK) recycling back to the plasma membrane for positive feedback, supporting the sustained activation of downstream kinases to promote HCC invasion and metastasis. They also demonstrated that cholesterol, dependent on GP73, protected RTKs from autophagy degradation and suppressed the efficacy of multiple tyrosine kinase inhibitors (TKIs) in HCC [14]. Recent studies have provided new insights into the roles of GP73 in reshaping the tumor microenvironment in HCC. The extracellular secretion of GP73, stimulated by endoplasmic reticulum stress (ERS), could interact with glucose-regulated protein 78 (GRP78) to amplify and transmit ERS signals to neighboring macrophages, which accelerated the differentiation of the tumor-associated macrophage (TAM) M2 phenotype [15]. GP73 exacerbates CD8^+^ T cell suppression in the setting of HCC by stabilizing programmed death-ligand 1 (PD-L1) via deubiquitination and the promotion of exosomal PD-L1 transport into TAMs [16]. Despite the fact that GP73 plays crucial roles in molecular signal transduction during the progression of HCC, very little data are available on the pro-angiogenic roles of GP73 in HCC.

Our previous multi-omics analysis at the bulk level revealed that GP73 overexpression in HCC was closely correlated with angiogenesis [17]. The present study was the first to generate a single-cell spatial transcriptome landscape of the vasculogenic etiology of HCC, uncover a GP73-mediated pro-angiogenic network, and elucidate the pro-angiogenic roles and mechanisms of GP73 in HCC.

## Results

### Positive correlation between serum GP73 levels and HCC response to anti-angiogenic agent, and between GP73 and CD34 expressions in HCC tissues

**Fig. 1.**
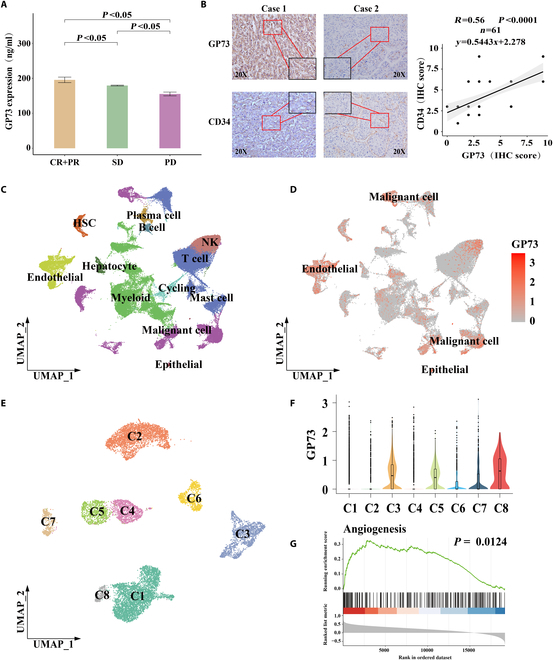
Serum GP73 was positively correlated with HCC response to anti-angiogenic agent and histology, and single-cell transcriptomics indicated that GP73 is positively associated with tumor angiogenesis in HCC tissues. (A) Correlation between serum GP73 levels and HCC response to anti-angiogenic agent lenvatinib among 238 advanced HCC patients. (B) Correlation between expressions of GP73 and CD34 in HCC tissues determined by IHC staining (*P* < 0.0001; *r* = 0.56; n = 61). (C to G) Single-cell transcriptomic profiling indicated that overexpressed GP73 HCC cells promoted tumor angiogenesis. (C) UMAP plot showing the cell types from single-cell transcriptome in all the studied samples, with different color codes denoting different cell clusters. (D) UMAP plots showing the GP73 expression in all the cell subclusters. (E) UMAP plot showing the HCC cell subclusters from all samples, with different color codes denoting individual cell subclusters. (F) Violin plot showing GP73 expressions in the HCC cell subclusters. (G) GSEA indicated that GP73 gene was significantly positively correlated with angiogenesis.

We evaluated the serum GP73 levels in 238 patients with HCC treated with lenvatinib. During the follow-up period, 55 patients (23.11%) had a complete response (CR) or partial response (PR), 128 (53.78%) had stable disease (SD), and 55 (23.11%) experienced progressive disease (PD). Serum GP73 levels were higher in CR + PR patients than in SD (*P* < 0.05) and PD (*P* < 0.05) patients, and higher in SD patients than in PD patients (*P* < 0.05) (Fig. 1A). Clinicopathological characteristics and serum GP73 levels were analyzed using univariate logistic regression. Indicators that were statistically significant (*P* < 0.05) were included in the multivariate logistic regression analysis, the results of which revealed that the serum GP73 level was an independent factor for tumor response to lenvatinib treatment (*P* < 0.001) (Table S3). Immunohistochemical (IHC) examinations showed that GP73 expression was positively correlated with CD34 expression in the 61 HCC tissues (*y* = 0.5443*x* + 2.278; *P* < 0.0001) (Fig. 1B). Clinicopathological characteristics are summarized in Table 1 and Table S1.

**Table 1.** Baseline and clinicopathological characteristics of 238 patients with advanced HCC treated with anti-angiogenesis in the entire cohort, training cohort, and validation cohort

Variables	Entire cohort (*n* = 238)	Training cohort (*n* = 119)	Validation cohort (*n* = 119)	*P*
Age (years), M(P25, P75)	50.00 (44.00, 58.00)	50.00 (43.00, 58.00)	50.00 (44, 57.00)	0.567
Sex, *n* (%)				0.406
Female	26 (10.92%)	15 (12.61%)	11 (9.24%)	
Male	212 (89.08%)	104 (87.39%)	108 (90.76%)	
HBV infection				0.290
No	25 (10.50%)	15 (12.61%)	10 (8.40%)	
Yes	213 (89.50%)	104 (87.39%)	109 (91.60%)	
HCV infection				>0.999
No	233 (97.90%)	117 (98.32%)	116 (97.48%)	
Yes	5 (2.10%)	2 (1.68%)	3 (2.52%)	
Serum AFP (ng/ml), *n* (%)				0.516
<400	127 (53.36%)	66 (55.46%)	61 (51.26%)	
≥400	111 (46.64%)	53 (44.54%)	58 (48.74%)	
Serum GP73 (ng/ml), median (IQR)	178 (89.75, 208.25)	183.50 (86.76, 210.50)	165.81 (93.48, 207.12)	0.429
Child–Pugh scores, *n* (%)				0.695
5–6	133 (55.88%)	65 (54.62%)	68 (57.14%)	
7	105 (44.12%)	54 (45.38%)	51 (42.86%)	
Tumor size (cm), *n* (%)				0.181
≤5	90 (37.82%)	40 (33.61%)	50 (42.02%)	
>5	148 (62.18%)	79 (66.39%)	69 (57.98%)	
Tumor number, *n* (%)				0.296
Single	104 (43.70%)	48 (40.34%)	56 (47.06%)	
Multiple	134 (56.30%)	71 (59.66%)	63 (52.94%)	
Macrovascular invasion, *n* (%)				0.604
No	114 (47.90%)	59 (49.58%)	55 (46.22%)	
Yes	124 (52.10%)	60 (50.42%)	64 (53.78%)	
Extrahepatic metastasis, *n* (%)				0.380
No	174 (73.11%)	90 (75.63%)	84 (70.59%)	
Yes	64 (26.89%)	29 (24.37%)	35 (29.41%)	
BCLC stages, *n* (%)				0.886
B	69 (28.99%)	35 (29.41%)	34 (28.57%)	
C	169 (71.01%)	84 (70.59%)	85 (71.43%)	
Combined ICI therapy, *n* (%)				0.663
No	65 (27.31%)	34 (28.57%)	31 (26.05%)	
Yes	173 (72.69%)	85 (71.43%)	88 (73.95%)	
Efficacy evaluation				0.939
CR + PR	55 (23.11%)	28 (23.53%)	27 (22.69%)	
SD	128 (53.78%)	65 (54.62%)	63 (52.94%)	
PD	55 (23.11%)	26 (21.85%)	29 (24.37%)	

HBV, hepatitis B virus; HCV, hepatitis C virus; BCLC, Barcelona Clinic Liver Cancer; IQR, interquartile range; AFP, α-fetoprotein; ICI, immune checkpoint inhibitor; PR, partial response; SD, stable disease; CR, complete response; PD, progression disease

To investigate the clinical applications of GP73 as an anti-angiogenic target in HCC, the 238 HCC patients treated with lenvatinib were included for the generation and validation of a serum GP73-based prognostic nomogram for determining the estimated survivals (Supplementary Results). The predicative performance of the nomogram is shown in Fig. S1A to L.

### Single-cell transcriptomic profiling revealed that overexpressed GP73 HCC cells promoted tumor angiogenesis

We then constructed a single-cell spatial transcriptomic landscape of the vasculogenic etiology of HCC using 6 HCC tissues and a normal liver tissue. Histological examinations demonstrated that the HCC samples from patients HCC1, HCC3, HCC4, and HCC5 were characterized by high microvascular density (MVD) ≥ 50/200 × high-power field of view (HPF). After standardized data processing and quality control, a total of 72,166 single-cell transcriptional profiles and 15,046 spatial resolution spots were obtained. The single cells, spatial spots, and clinicopathological features are listed in Table S2. Additionally, 48 HCC single-cell datasets and 5 normal liver single-cell datasets were collected from the Gene Expression Omnibus (GEO) database for validation (Table S2). Through Uniform Manifold Approximation and Projection (UMAP), 12 cell types were visually identified, among which malignant cells [serine protease inhibitor-1 (SERPINA1), glypican-3 (GPC3), and α-fetoprotein (AFP)] accounted for the highest proportion, although an abundance of endothelial cells [secreted protein acidic and rich in cysteine-like protein-1 (SPARCL1), cadherin 5 (CDH5), and von Willebrand factor (VWF)] was also captured (Fig. 1C and Fig. S1M and N). We found that GP73 was commonly overexpressed in HCC and endothelial cells (Fig. 1D), especially in patients HCC1, HCC5, and HCC6 (Fig. S1O). Then, HCC cells were isolated and subdivided into 8 subpopulations (Fig. 1E), among which GP73 expression was significantly elevated in cell clusters C3, C5, and C8 (Fig. 1F). Gene set enrichment analysis (GSEA) indicated that angiogenesis was activated with the overexpression of GP73 in HCC cells (Fig. 1G). The results of these analyses implied that GP73 overexpression in HCC cells potentially promoted tumor angiogenesis.

### Single-cell spatial transcriptomics suggested that overexpressed GP73 HCC cells promoted HCC angiogenesis by regulating diverse pro-angiogenic factors

**Fig. 2.**
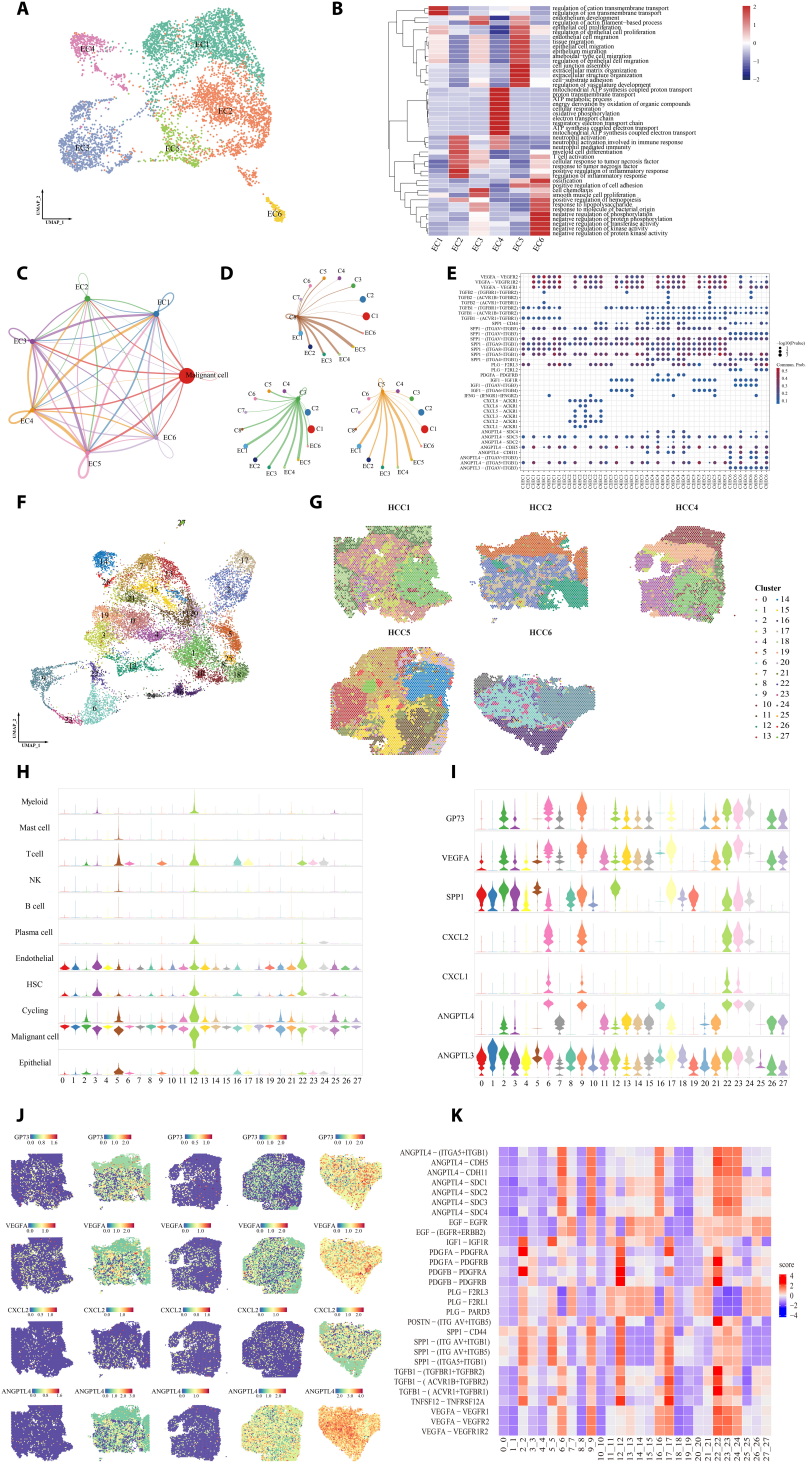
Single-cell spatial transcriptomics suggested that overexpressed GP73 HCC cells promoted tumor angiogenesis by regulating diverse angiogenic factors. (A) UMAP plot showing the endothelial cell types from all the studied HCC samples, with different color codes denoting individual cell subclusters. (B) Heatmap representing the GO enrichment analysis of endothelial cell subclusters from all the studied HCC samples. (C and D) Differences in the number and strength of communications between HCC cells and endothelial cell subclusters. The thicker the line, the greater the difference. (E) Heatmap representing the receptor–ligand pairs between HCC cells subclusters and endothelial cells EC1 and EC5. (F) UMAP plot showing the cell clusters from spatial transcriptome in all the studied samples, with different color codes denoting individual cell clusters. (G) Spatial plot mapping distributions of the cell clusters in all the studied HCC samples. (H) Violin plot showing the distribution of cell types in all the cell clusters. (I) Violin plot showing the expression of GP73 and ligands associated with angiogenesis in all the cell clusters. (J) Spatial plot showing the distribution of GP73 and ligands associated with angiogenesis. (K) Heatmap representing the receptor–ligand pairs in all the cell clusters.

To investigate the intercellular communication between the overexpressed GP73 HCC cells and endothelial cells, we divided the endothelial cells into 6 subpopulations (Fig. 2A). Gene ontology (GO) enrichment analysis showed that EC1 and EC5 cells were primarily involved in endothelial development, regulation of vasculature development, regulation of vasculature epithelial cell proliferation, and epithelial cell migration, suggesting that these cells have high pro-angiogenic potential (Fig. 2B). The CellChat analysis revealed that HCC cells exhibited the strongest intercellular communication with EC1 and EC5 cells (Fig. 2C). HCC C8 cells expressed the highest levels of GP73 and presented the strongest communication with EC1 and EC5 cells, and EC5 cells in particular (Fig. 2D). The heatmap visualized showed that overexpressed GP73 HCC cell clusters interacted with endothelial cells through multiple angiogenic receptor–ligand reactions, including CXCL5–ACKR1, VEGFA–VEGFR1/R2, TGFB1–(TGFBR1 + TGFBR2), SPP1–(ITGAV + ITGB1), and ANGPTL4–CDH5 (Fig. 2E). Correlation analysis revealed that GP73 could interact with a variety of pro-angiogenic factors; in particular, it showed the highest correlation coefficient with GP73 and VEGFA (Spearman’s *r* = 0.643; *P* < 0.001) and CXCL5 (Spearman’s *r* = 0.628; *P* < 0.001) (Fig. S2A to F).

Considering that adjacent cells are more likely to interact directly, we utilized spatial transcriptomics to compensate for the spatial information lost during single-cell transcriptomics [18]. A total of 28 cell subpopulations were identified with UMAP (Fig. 2F), and their spatial distribution features were mapped (Fig. 2G). Based on the specific positive markers in individual single cells, violin plots showed that HCC cells were frequently a high proportion of all cell subclusters, while endothelial cells accounted for the highest proportion in cell clusters 3, 12, and 22 (Fig. 2H). Interestingly, we found that elevated expression of the angiogenic ligands VEGFA, SPP1, CXCL2, and ANGPTL4 was highly consistent with increased GP73 expression in each cell subcluster (Fig. 2I), while their distributions showed strong spatial activation in overexpressed GP73 regions (Fig. 2J). The heatmap (Fig. 2K) showed that elevated VEGFA–VEGFR expression was correlated with increased GP73 in cell clusters 6, 9, 16, 17, 22, 23, and 24, while cell clusters 3, 12, and 22, rich in vascular endothelial cells, strongly interacted with overexpressed GP73 HCC cells through the receptor–ligand interactions VEGFA–VEGFR1/R2, TGFB1–(TGFBR1 + TGFBR2), SPP1–(ITGAV + ITGB1), and ANGPTL4–CDH5. Taken together, the single-cell spatial transcriptomic data suggested that HCC cells stimulated diverse pro-angiogenic factors, which was consistent with the results of the validation by expanding HCC single-cell samples from the GEO databases (GSE1566256, GSE149614, and GSE151530) (Fig. S2G to N and Supplementary Results).

### GP73 promoted HCC angiogenesis in vitro and in vivo

**Fig. 3.**
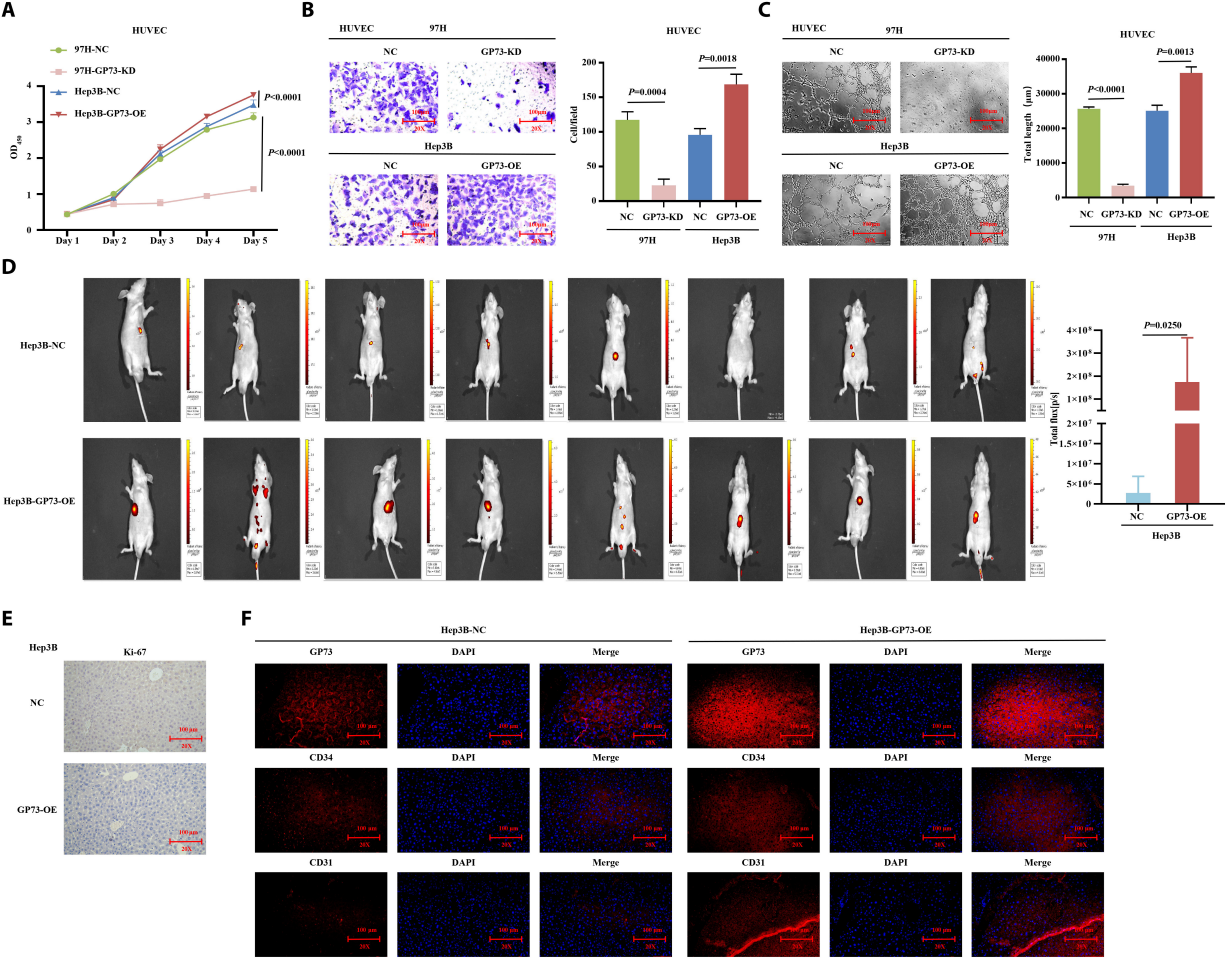
GP73 promotes HCC angiogenesis in vivo and in vitro. (A to C) Modulation of GP73 expression in human liver cancer cells affects HUVEC proliferation, migration, and tubule formation abilities. Data are mean values of 3 separate experiments ± SEM. (A) The growth of cells over 5 d was measured using cell counting kit (CCK-8) assays. GP73-KD indicates that GP73 was knocked down in MHCC97H cells, and GP73-OE indicates that GP73 was overexpressed in Hep3B cells; NC indicates MHCC97H and Hep3B cells transfected with a vector-expressing green fluorescent protein (GFP). (B) Transwell Matrigel invasion assay. Representative photographs and quantification are shown. Columns, average of 3 independent experiments; original magnification, ×200. (C) Matrix tubule formation assay. Representative photographs and quantification are shown. Columns, average of 3 independent experiments; original magnification, ×200. (D to F) GP73 promoted the HCC tumor angiogenesis in vivo. (D) Hep3B-GP73-OE and control cells were orthotopically injected into the hepatic subcapsular of nude mice. Tumorigenicity and metastasis followed up by bioluminescence fluorescence over 6 weeks presented larger and multiple tumors with stronger fluorescence intensity that originated from Hep3B-GP73-OE cells compared to the control. (E) IHC staining showing increased Ki-67 levels in resected tumors that originated from the Hep3B-GP73-OE cells compared to the control. Original magnification, ×200. (F) Immunofluorescence staining showing increased expressions of GP73, CD34, and CD31 in resected tumors that originated from the Hep3B-GP73-OE cells compared to the control. Original magnification, ×200.

We investigated the pro-angiogenic roles of GP73 in vitro and in vivo. Our previous study [19] revealed that MHCC97H cells show the highest GP73 expression, whereas Hep3B cells show the lowest. In the present study, we generated MHCC97H-GP73-KD and Hep3B-GP73-OE cell lines (Fig. S3A and B) and cocultured their supernatant with HUVECs. In vitro studies demonstrated that knocked down GP73 in MHCC97H cells decreased proliferation (*P* < 0.001), migration (*P* = 0.0004), and tube formation ability (*P* < 0.001) of HUVECs, while overexpressed GP73 in Hep3B cells enhanced their proliferation (*P* < 0.001), migration (*P* = 0.0018), and tube formation ability (*P* = 0.0013) (Fig. 3A to C). In vivo studies using bioluminescence fluorescence to evaluate tumor growth and metastasis in orthotopic xenograft mouse liver tumor models over a 6-week period showed larger and multiple tumors with stronger fluorescence intensity originating from Hep3B-GP73-OE cells than the control (Fig. 3D). IHC revealed increased Ki-67 levels in resected tumors originating from Hep3B-GP73-OE cells (Fig. 3E). MVD is an important marker of tumor microangiogenesis that has been widely utilized in clinical practice and described in numerous studies. CD31 [platelet and endothelial cell adhesion molecule 1 (PECAM1)] and CD34 (hematopoietic progenitor cell antigen 34) are the most widely used markers and are known to be positively correlated with the density and grade of MVD [20]. Immunofluorescence analysis showed increased MVD with increased CD34 and CD31 expression in overexpressed GP73 tumor-bearing tissues (Fig. 3F and Fig. S3C). These data suggest that GP73 induces the in vitro growth and migration of HUVECs and promotes HCC tumor angiogenesis in vivo.

### GP73 activated the JAK2/STAT3 pathway in stimulating HCC angiogenesis

**Fig. 4.**
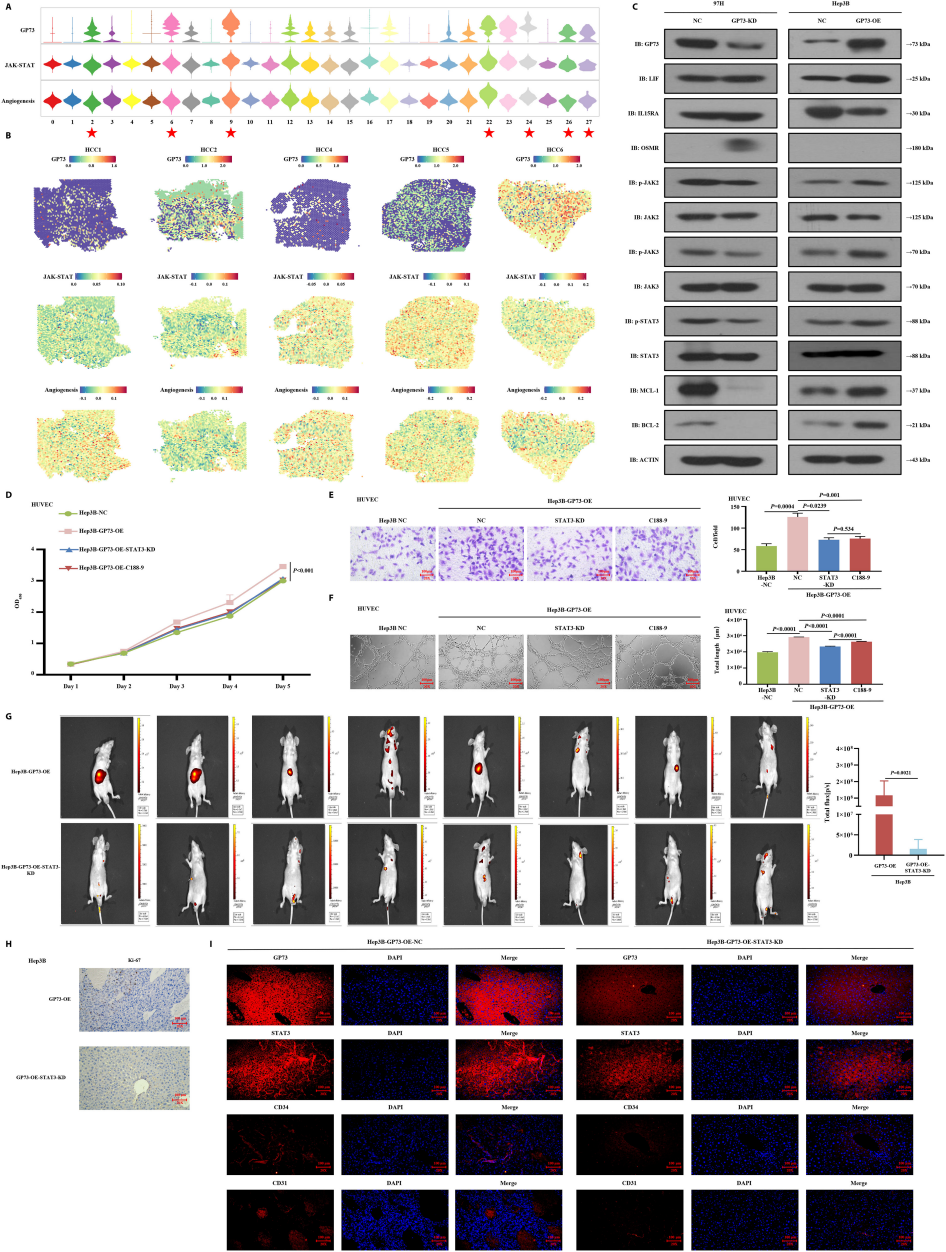
GP73 activated the JAK2/STAT3 signaling in stimulating HCC angiogenesis in vivo and in vitro. (A) Violin plot showing the GP73, JAK/STAT pathway, and angiogenesis signaling scores among the HCC cell clusters. ★: High GP73 expression of HCC cell clusters 2, 6, 9, 22, 24, 26, and 27. (B) Spatial plot showing the transcription activity of GP73, JAK/STAT, and angiogenesis pathway signaling. (C) Western blot detection of key gene expressions in the JAK2/STAT3 pathway in MHCC97H-GP73-KD, MHCC97H-GP73-NC, Hep3B-GP73-OE, and Hep3B-GP73-NC cells. Data are representative of 3 similar observations or are shown as means ± SD of 3 experiments. (D to F) Down-regulation of STAT3 in overexpressed GP73 HCC cells and STAT3-specific inhibitor (C188-9) affects HUVEC proliferation, migration, and tubule formation abilities. Data are mean values of 3 separate experiments ± SEM. (D) The growth of cells over 5 d was measured using CCK-8 assays. NC indicates that Hep3B cells were transfected with a vector-expressing GFP; GP73-OE indicates that GP73 was overexpressed in Hep3B cells; STAT3-KD indicates that GP73 was knocked down in Hep3B-GP73-OE cells; C188-9 indicates that the coculture of Hep3B-GP73-OE cells and HUVECs was treated with C188-9 (10 μM) for 24 h. (E) Transwell Matrigel invasion assay. Representative photographs and quantification are shown. Columns, average of 3 independent experiments; original magnification, ×200. (F) Matrix tubule formation assay. Representative photographs and quantification are shown. Columns, average of 3 independent experiments; original magnification, ×200. (G to I) Down-regulation of STAT3 suppressed the GP73-induced tumor angiogenesis in vivo. (G) Hep3B-GP73-OE and Hep3B-GP73-OE-STAT3-KD cells were orthotopically injected into the hepatic subcapsular of nude mice. Tumorigenicity and metastasis followed up by bioluminescence fluorescence over 6 weeks presented smaller and less tumors with weaker fluorescence intensity that originated from Hep3B-GP73-OE-STAT3-KD cells compared to the Hep3B-GP73-OE cells. (H) IHC staining showing decreased Ki-67 levels in resected tumors originating from the Hep3B-GP73-OE-STAT3-KD cells compared the Hep3B-GP73-OE cells. Original magnification, ×200. (I) Immunofluorescence staining showing decreased expressions of GP73, STAT3, CD34, and CD31 in resected tumors originating from the Hep3B-Gp73-OE-STAT3-KD cells compared to the Hep3B-GP73-OE cells. Original magnification, ×200.

Single-cell spatial transcriptomic data showed that Janus kinase/signal transducer and activator of transcription (JAK/STAT) and angiogenic signals were activated in overexpressed GP73 HCC cell clusters (Fig. S4A and B), showing particularly high scores in GP73 HCC cell clusters 2, 6, 9, 22, 24, 26, and 27 (Fig. 4A) and exhibiting strong spatial activation in GP73 overexpression regions (Fig. 4B and Fig. S4C and D), suggesting that GP73 potentially activates the JAK/STAT pathway to stimulate HCC angiogenesis. Western blot analysis showed that the expression of JAK2, pJAK2, STAT3, and pSTAT3 in the JAK2/STAT3 pathway and the downstream targets MCL-1 and BCL-2 was increased in Hep3B-GP73-OE cells but was decreased in MHCC97H-GP73-KD cells (Fig. 4C and Fig. S4E). When STAT3 was down-regulated in Hep3B-GP73-OE cells, the messenger RNA (mRNA) and protein expression of MCL-1 and BCL-2 were inhibited (Fig. S4F and G).

Next, in vitro rescue studies demonstrated that knocked down STAT3 in Hep3B-GP73-OE cells suppressed the proliferation (*P* < 0.001), migration (*P* = 0.0239), and tube formation abilities (*P* < 0.001) of HUVECs (Fig. 4D to F). When the cocultured Hep3B-GP73-OE cells and HUVECs were treated with a STAT3-specific inhibitor (C188-9), the proliferation (*P* < 0.001), migration (*P* = 0.001), and tube formation abilities (*P* < 0.001) of HUVECs were decreased (Fig. 4D to F). Additionally, by using an angiogenesis quantitative polymerase chain reaction (qPCR) array to investigate the GP73-mediated differentially expressed genes (DEGs) and a heatmap for visualization (Fig. S4H), we found that the pro-angiogenic factors CXCL5 [21], MCP-1 [22], and TPO [23] were significantly activated when GP73 was up-regulated in Hep3B-GP73 cells, but inhibited when Hep3B-GP73-OE cells were treated with C188-9 and GP73 was down-regulated in MHCC97H cells (Fig. S4I), indicating that CXCL5, MCP-1, and TPO were activated by GP73 and JAK2/STAT3 signaling.

Furthermore, by generating orthotopic xenograft mouse liver tumor models and monitoring tumor growth and metastasis via bioluminescence over a 6-week period, we found that knocked down STAT3 suppressed the tumorigenicity and metastasis of Hep3B-GP73-OE cells with weaker fluorescence intensity (Fig. 4G), suggesting that the down-regulation of STAT3 impaired GP73-induced tumor growth and metastasis in vivo. IHC examinations showed decreased Ki-67 levels in resected tumors originating from Hep3B-GP73-OE-STAT3-KD cells compared to Hep3B-GP73-OE cells (Fig. 4H). Additionally, immunofluorescence analysis showed a lower MVD with decreased CD34 and CD31 expression in knocked down STAT3 tumor-bearing tissues (Fig. 4I and Fig. S4J). Taken together, these results suggest that GP73 targets the activation of the JAK2/STAT3 pathway to stimulate HUVEC growth and migration in vitro and promotes HCC tumor angiogenesis in vivo*.*

### GP73 directly bound STAT3 and simultaneously enhanced GRP78-induced ERS for stimulating STAT3 phosphorylation

**Fig. 5.**
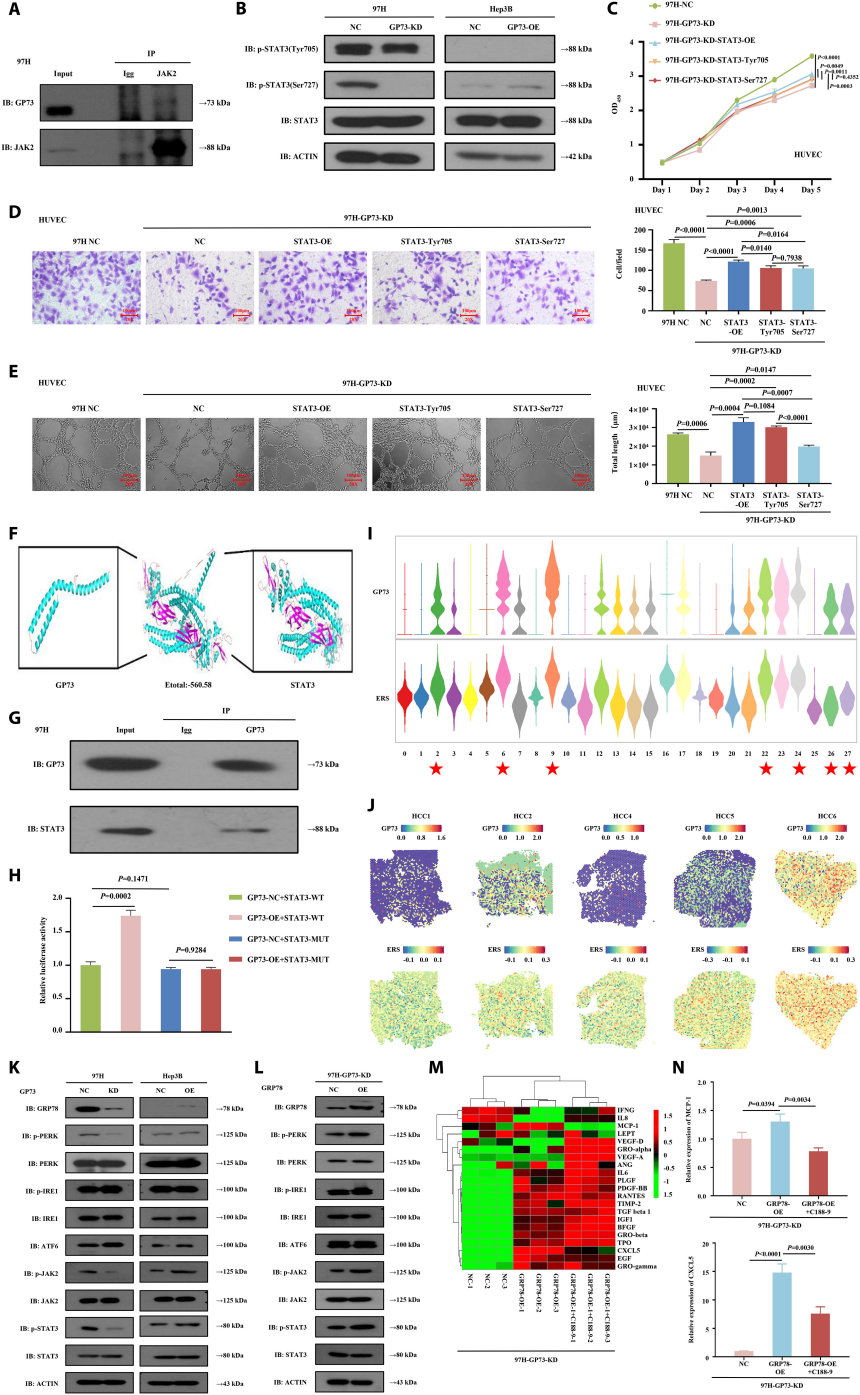
GP73 activated the JAK2/STAT3 signaling by simultaneously directly binding STAT3 and indirectly enhancing a GRP78-induced ERS signal for the stimulation of STAT3 phosphorylation. (A) Co-IP assays detected the interaction between GP73 and JAK2. (B to H) GP73 directly binds STAT3 for the stimulation of STAT3 phosphorylation. (B) Western blot detection of the expressions of STAT3 and the phosphorylated STAT3 proteins STAT3-Tyr^705^ and STAT3-Ser^727^ in MHCC97H-GP73-KD, MHCC97H-GP73-NC, Hep3B-GP73-OE, and Hep3B-GP73-NC cells. Data are representative of 3 similar observations or are shown as means ± SD of 3 experiments. (C to E) Point mutations of STAT3-Tyr^705^ and STAT3-Ser^727^ in liver cancer cells affects HUVEC proliferation, migration, and tubule formation abilities. Data are mean values of 3 separate experiments ± SEM. (C) The growth of cells over 5 d was measured using CCK-8 assays. NC indicates that MHCC97H cells were transfected with a vector-expressing GFP; GP73-KD indicates that GP73 was knocked down in MHCC97H cells; STAT3-OE indicates that STAT3 was overexpressed in MHCC97H-GP73-KD cells. STAT3-Tyr^705^ and STAT3-Ser^727^ indicates the point mutations of STAT3-Tyr^705^ and STAT3-Ser^727^ in MHCC97H-GP73-KD cells. (D) Transwell Matrigel invasion assay. Representative photographs and quantification are shown. Columns, average of 3 independent experiments; original magnification, ×200. (E) Matrix tubule formation assay. Representative photographs and quantification are shown. Columns, average of 3 independent experiments; original magnification, ×200. (F) Molecular docking study showing the molecular binding ability between GP73 and STAT3. (G) Co-IP assays detected the interaction between GP73 and STAT3. (H) Luciferase reporter assay showed the influence of up-regulation of GP73 on STAT3-WT and STAT3-MT transcriptions. (I and J) GP73 stimulated phosphorylation of STAT3 by indirectly enhancing a GRP78-induced ERS signal. (I) Violin plot showing the GP73 and ERS signal scores among the HCC cell clusters. ★: High GP73 expression of HCC cell clusters 2, 6, 9, 22, 24, 26, and 27. (J) Spatial plot showing the transcription activity of GP73 and ERS signal. (K) Western blot detection of key gene expressions in the JAK2/STAT3 and ERS pathway in MHCC97H-GP73-KD, MHCC97H-GP73-NC, Hep3B-GP73-OE, and Hep3B-GP73-NC cells. Data are representative of 3 similar observations or are shown as means ± SD of 3 experiments. (L) Western blots determined the impact of overexpressed GRP78 on the alterations of key gene expressions in the JAK2/STAT3 and ERS pathways in MHCC97H-GP73-KD cells. Data are representative of 3 similar observations or are shown as means ± SD of 3 experiments. (M) Heatmap showing differential expressions of 21 key angiogenic factors among MHCC97H-GP73-KD, MHCC97H-GP73-KD-GRP78-OE, and MHCC97H-GP73-KD-GRP78-OE-C188-9 cells. C188-9 indicates that MHCC97H-GP73-KD-GRP78-OE cells were treated with C188-9 (10 μM) for 24 h. (N) Determination of expressions of pro-angiogenic factors CXCL5 and MCP-1 in MHCC97H-GP73-KD, MHCC97H-GP73-KD-GRP78-OE, and MHCC97H-GP73-KD-GRP78-OE-C188-9 cells by angiogenesis qPCR array.

Interestingly, the co-immunoprecipitation (co-IP) experiment showed no interaction between GP73 and JAK2 (Fig. 5A and Fig. S5A); however, we did find that the mRNA and protein expression levels of MCL-1 and BCL-2 were reversely increased when STAT3 was reduplicatively up-regulated in MHCC97H-GP73-KD cells (Fig. S5B to D). STAT3 has 2 common conserved amino acid residues (Tyr^705^ and Ser^727^), and their phosphorylation is essential for the constitutive activation of STAT3. Western blot analysis demonstrated that pSTAT3-Ser^727^ expression was suppressed in MHCC97H-GP73-KD cells but increased in Hep3B-GP73-OE cells (Fig. 5B and Fig. S5E and F). However, regardless of the presence of up- or down-regulation of GP73, no significant alteration in pSTAT3-Tyr^705^ expression was observed. We created point mutations of pSTAT3-Ser^727^ and pSTAT3-Tyr^705^ in MHCC97H-GP73-KD-STAT3-OE cells (Fig. S5G and H), from which in vitro studies demonstrated that pSTAT3-Ser^727^ mutations led to significant inhibition of the proliferation (*P* = 0.0011), migration (*P* = 0.0164), and tube formation abilities (*P* = 0.0007) of HUVECs (Fig. 5C to E). Meanwhile, pSTAT3-Tyr^705^ mutations also decreased the proliferation (*P* = 0.0049) and migration (*P* = 0.0140) of HUVECs, but not their tube formation abilities (*P* = 0.1084) (Fig. 5C to E). These results suggest that GP73 activates the JAK2/STAT3 pathway primarily by stimulating STAT3-Ser^727^ phosphorylation.

Furthermore, a molecular docking study showed favorable molecular binding abilities between GP73 and STAT3 with a van der Waals force of −560.58 kJ/mol (Fig. 5F), and the co-IP assay showed direct interactions between GP73 and STAT3 (Fig. 5G and Fig. S5I). To clarify the effects of GP73 on the function of STAT3 as a transcription factor (TF), we constructed a mutant (MUT) of the STAT3 binding site at TTCCCGTAA, which influenced the TF function of STAT3. A dual-luciferase reporter assay showed that the overexpression of GP73 increased the transcription of STAT3-wild type (STAT3-WT; *P* = 0.0002), but not STAT3-MUT (*P* = 0.9284), in MHCC97H cells (Fig. 5H). These results indicate that GP73 directly binds to STAT3 to stimulate STAT3-Ser^727^ phosphorylation.

We also noticed that ERS manifested high scores in overexpressed GP73 HCC cell clusters 2, 6, 9, 22, 24, 26, and 27 (Fig. 5I) and exhibited strong spatial activation in regions of GP73 overexpression (Fig. 5J and Fig. S5J), suggesting that GP73 potentially enhanced ERS to promote tumor angiogenesis. Western blot analysis demonstrated that the expressions of pERK (phosphorylated extracellular signal-regulated kinase), pJAK2, pSTAT3, and the ERS-related gene GRP78 [17] were increased in Hep3B-GP73-OE cells, while reduced in MHCC97H-GP73-KD cells (Fig. 5K and Fig. S5K and L) but reversely increased after reduplicatively overexpressed GRP78 (Fig. 5L and Fig. S5M)*.* Using angiogenesis qPCR array detection and a heatmap (Fig. 5M), we found that CXCL5 and MCP-1 were activated in MHCC97H-GP73-KD-GRP78-OE cells but inhibited in MHCC97H-GP73-KD-GRP78-OE-C188-9 cells (Fig. 5N), and that MCP-1 and CXCL5 were verified to be cotargeted by JAK2/STAT3 and ERS signals. These results indicated that GP73 indirectly stimulates STAT3-Ser^727^ phosphorylation by enhancing GRP78-induced ERS.

### Histone lactylation promoted GP73 expression

**Fig. 6.**
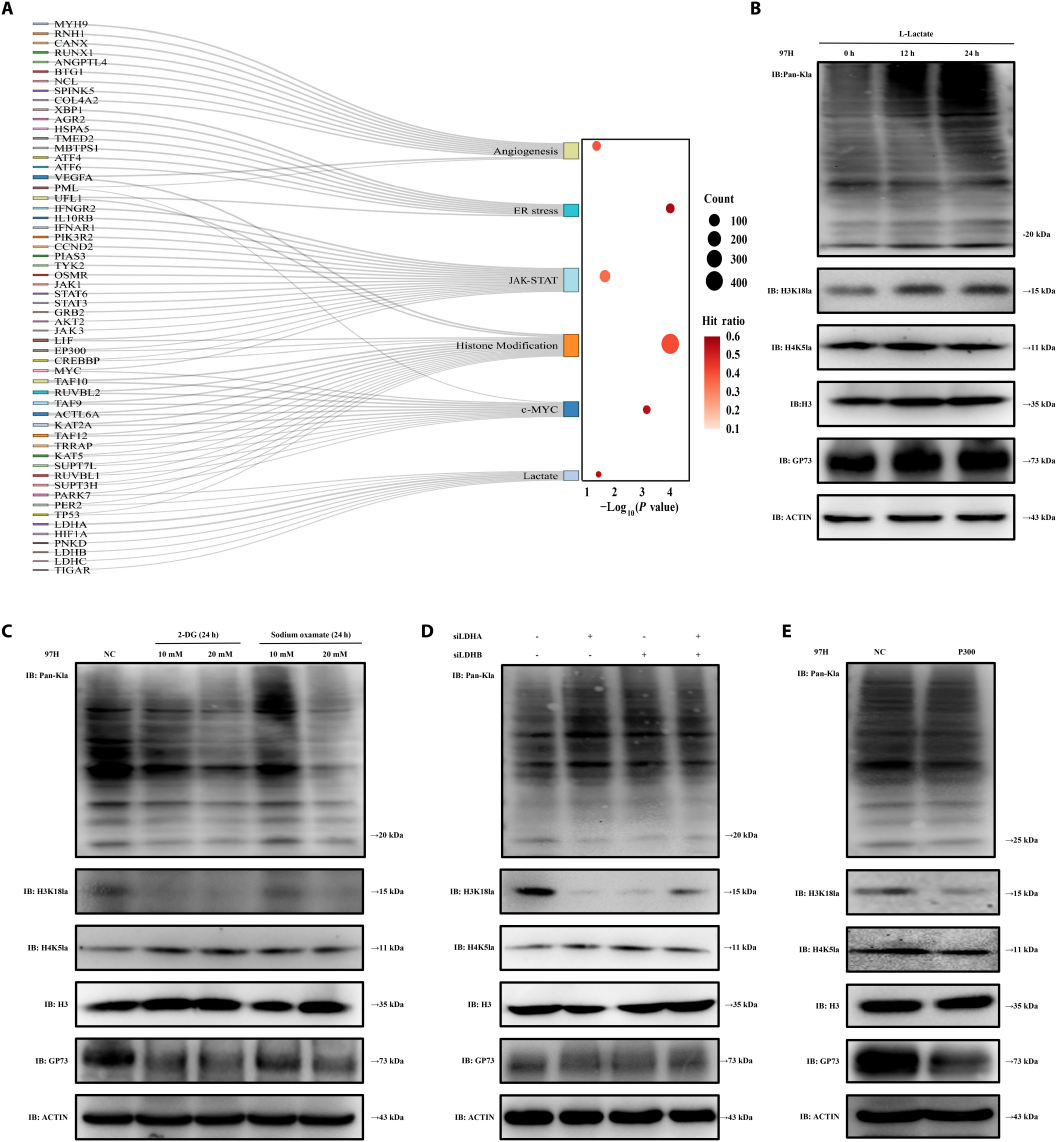
Histone lactation promoted GP73. (A) Sankey flow diagram was generated using spatial single-cell transcriptomics, indicating that GP73 gene was significantly positively correlated with c-Myc, histone modification, lactation, JAK-STAT, ERS, and angiogenesis. (B and C) Western blot detection of Pan-Kla, H3K18la, H4K5la, and GP73 expressions in MHCC97H cells. 2-DG and LDH-A inhibitor (sodium oxalate) indicate that MHCC97H cells were treated with 2-DG (10 mM, 20 mM) and LDH-A inhibitors (sodium oxalate) (10 and 20 mM) for 24 h. Data are representative of 3 similar observations or are shown as means ± SD of 3 experiments. (D) Western blot detection of Pan-Kla, H3K18la, H4K5la, and GP73 expressions in MHCC97H-LDHA-KD, MHCC97H-LDHB-KD, and MHCC97H-LDHA-KD-LDHB-KD cells. (E) Western blot detection of Pan-Kla, H3K18la, H4K5la, and GP73 expressions in MHCC97H-P300-NC and MHCC97H-P300-KD cells. Data are representative of 3 similar observations or are shown as means ± SD of 3 experiments.

We further explored the mechanisms by which upstream factors activated GP73 expression. A Sankey flow diagram was generated using spatial single-cell transcriptomic data, which strongly indicated that c-Myc, lactate, histone modification, JAK/STAT, and ERS signals were involved in the GP73-mediated pro-angiogenic network (Fig. 6A). Meanwhile, GSEA indicated that overexpressed GP73 HCC cells presented increased c-Myc, ERS, and lactate (Fig. S6A to C). Western blot analysis revealed that Pan-Kla and GP73 expressions were elevated along with an increased lactate concentration gradient in MHCC97H cells, suggesting that lactate induced increased GP73 expression (Fig. 6B and Fig. S6D). We subsequently found that when MHCC97H cells were treated with a glycolytic inhibitor [2-deoxy-D-glucose (2-DG)] and a lactate dehydrogenase A (LDH-A) inhibitor (odium oxamate), the expressions of Pan-Kla, H3K18la, and GP73 were reduced other than H4K5la (Fig. 6C and Fig. S6E). Meanwhile, the expressions of Pan-Kla, H3K18la, and GP73, but not H4K5la, were inhibited after the knockdown of LDHA or LDHB, but their alteration status was not stronger when LDHA and LDHB were simultaneously down-regulated (Fig. 6D and Fig. S6F to H). Additionally, the knockdown of P300 decreased the expressions of Pan-Kla, H3K18la, and GP73 (Fig. 6E and Fig. S6I to K), implying that P300 is required for H3K18la lactylation. These results suggested that histone lactylation promoted GP73 expression and that P300 might be a potential “writer” enzyme.

### c-Myc separately up-regulated GP73 and STAT3, while STAT3 up-regulated GP73

**Fig. 7.**
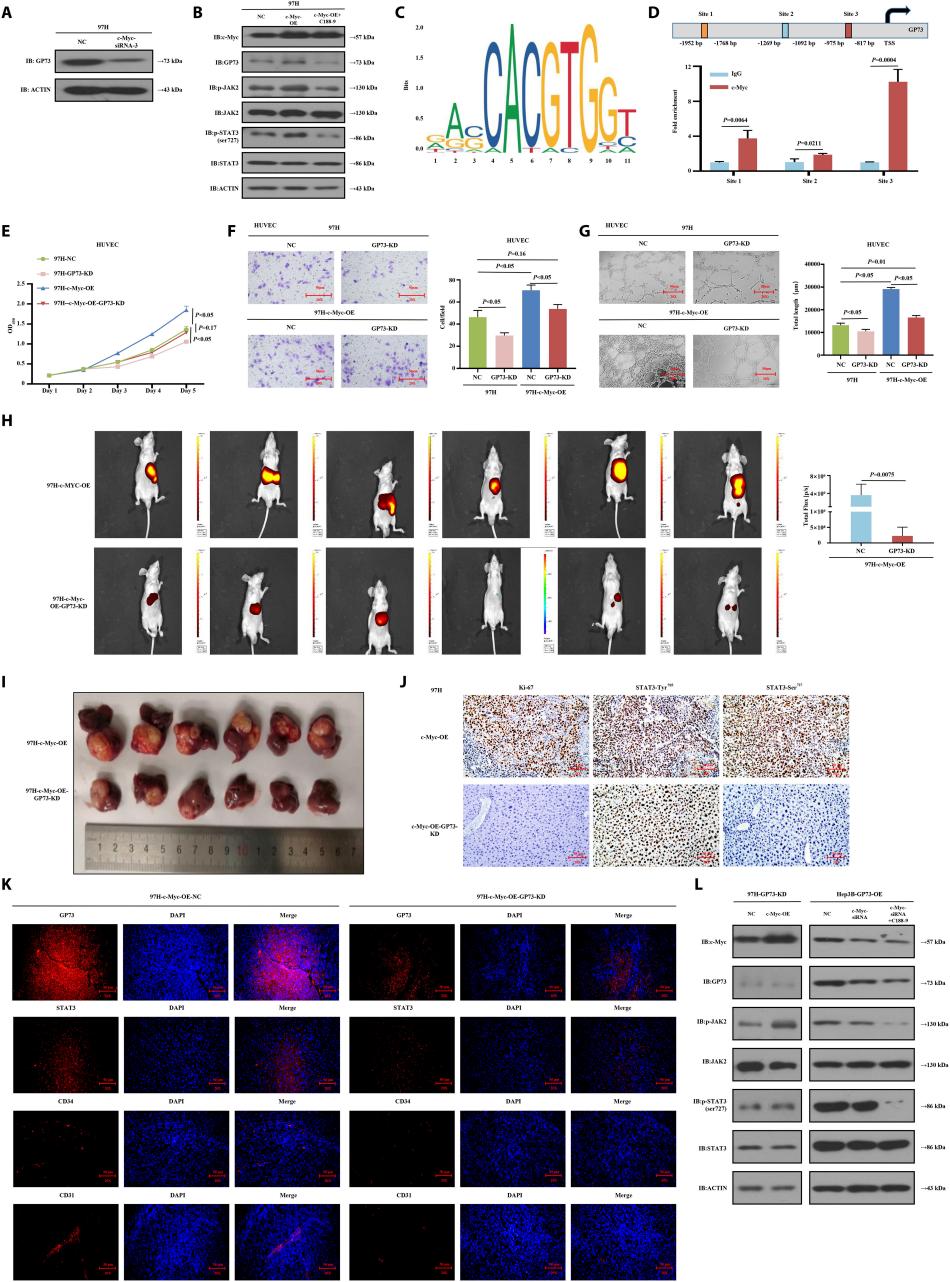
c-Myc separately up-regulated GP73 and STAT3, while STAT3 up-regulated GP73. (A) small interfering RNA-3 (siRNA-3) with the highest c-Myc knockdown efficiency was selected for follow-up experiments to detect and verify the changes of GP73 protein in MHCC97H HCC cells. (B) Western blot detection of the expressions of GP73 and key genes in the JAK2/STAT3 pathway in MHCC97H-c-Myc-NC, MHCC97H-c-Myc-OE, and MHCC97H-c-Myc-OE-C188-9 cells. C188-9 indicates that MHCC97H-c-Myc-OE cells were treated with C188-9 (10 μM) for 24 h. Data are representative of 3 similar observations or are shown as means ± SD of 3 experiments. (C) Motif sequence structure diagram showing the potential binding sites of c-Myc and DNA promoter sequences of GP73 genes. (D) ChIP-qPCR assay determined the binding sites of c-Myc and GP73. (E to G) Down-regulation of GP73 in MHCC97H-c-Myc-OE cells affects proliferation, migration, and tubule formation abilities of HUVECs. Data are mean values of 3 separate experiments ± SEM. (E) The growth of cells over 5 d was measured using CCK-8 assays. NC indicates that MHCC97H cells were transfected with a vector-expressing GFP; c-Myc-OE indicates that c-Myc was overexpressed in MHCC97H cells; GP73-KD indicates that GP73 was knocked down in MHCC97H cells and MHCC97H-c-Myc-OE cells. (F) Transwell Matrigel invasion assay. Representative photographs and quantification are shown. Columns, average of 3 independent experiments; original magnification, ×200. (G) Matrix tubule formation assay. Representative photographs and quantification are shown. Columns, average of 3 independent experiments; original magnification, ×200. (H to K) Down-regulation of STAT3 suppressed the GP73-induced HCC tumor angiogenesis in vivo. (H) MHCC97H-c-Myc-OE and MHCC97H-c-Myc-OE-GP73-KD cells were orthotopically injected into the hepatic subcapsular of nude mice. Tumorigenicity and metastasis followed up by bioluminescence fluorescence over 6 weeks presented larger and multiple tumors with stronger fluorescence intensity that originated from MHCC97H-c-Myc-OE cells compared to MHCC97H-c-Myc-OE-GP73-KD cells. (I) MHCC97H-c-Myc-OE-GP73-KD cells formed smaller and lighter tumor compared to MHCC97H-c-Myc-OE cells (NC). Data are mean values of experiments ± SEM. (J) IHC staining showing decreased Ki-67, pSTAT3-Tyr^705^, and pSTAT3-Ser^727^ expressions in the resected tumors that originated from MHCC97H-c-Myc-OE-GP73-KD cells compared to MHCC97H-c-Myc-OE cells. Original magnification, ×200. (K) Immunofluorescence staining showing decreased expressions of GP73, STAT3, CD34, and CD31 in the resected tumors that originated from MHCC97H-c-Myc-OE-GP73-KD cells compared to MHCC97H-c-Myc-OE cells. Original magnification, ×200. Data are representative of 3 similar observations or are shown as means ± SD of 3 experiments. (L) Western blot detection determined the impact of modulation of c-Myc and C188-9 on the alteration of expressions of GP73, c-Myc, and key genes in the JAK2/STAT3 pathway in MHCC97H-GP73-KD and Hep3B-GP73-OE cells. Data are representative of 3 similar observations or are shown as means ± SD of 3 experiments.

Based on the aforementioned results (Fig. 6A and Fig. S6A), we further investigated the role of c-Myc in GP73-mediated pro-angiogenic networks. Given that c-Myc is a crucial TF [24], we explored its regulatory role in GP73 modulation and found that knocked down c-Myc suppressed the mRNA and protein expressions of GP73 (Fig. 7A and Fig. S7A to C), whereas the overexpression of c-Myc increased the expressions of GP73, JAK2, pJAK2, STAT3, pSTAT3, and pSTAT3-Ser^727^ in MHCC97H cells (Fig. 7B and Fig. S7D and E). Using the JASPAR database, motif sequence diagrams displayed the potential binding sequences of c-Myc and GP73 DNA promoter (Fig. 7C), while ChIP-qPCR showed that the GP73 DNA promoter sequences of −915 to −817 base pairs (bp) (site 3) had the closest binding ability with c-Myc (Fig. 7D).

We then constructed MHCC97H-NC, MHCC97H-GP73-KD, MHCC97H-c-Myc-OE, and MHCC97H-c-Myc-OE-GP73-KD cell lines (Fig. S7F) and cocultured these cells with HUVECs. In vitro studies demonstrated that knocked down GP73 in MHCC97H cells significantly inhibited the proliferation (*P* < 0.05), migration (*P* < 0.05), and tube formation abilities (*P* < 0.05) of HUVECs, whereas overexpression of c-Myc in MHCC97H cells enhanced their proliferation (*P* < 0.05), migration (*P* < 0.05), and tube formation abilities (*P* < 0.05) of HUVECs (Fig. 7E to G). Even after c-Myc overexpression, however, knocked down GP73 still significantly suppressed the HUVEC phenotype (*P* < 0.05) (Fig. 7E to G). The in vivo study demonstrated that MHCC97H-c-Myc-OE cells generated larger and more numerous tumors with stronger fluorescence intensity than MHCC97H-c-Myc-OE-GP73-KD cells in orthotopic xenograft mouse liver tumor models (Fig. 7H and I). IHC examinations showed decreased Ki-67 and pSTAT3-Ser^727^ levels in resected tumors originating from MHCC97H-c-Myc-OE- GP73-KD cells compared to MHCC97H-c-Myc-OE cells (Fig. 7J), while immunofluorescence analysis showed lower MVD and decreased CD34 and CD31 expressions in knocked down GP73 tumor-bearing tissues (Fig. 7K and Fig. S7G). These results depicted the functional dependence of c-Myc on GP73 in promoting angiogenesis in HCC.

Among Myc family members, c-Myc, which regulates diverse target genes, especially STAT3, has been widely studied [25]. STAT3 is a pivotal TF, which regulates the expression of multiple genes [26]. As shown in Fig. 7B, GP73 expression was significantly inhibited after treatment with C188-9, indicating that the inhibition of the JAK2/STAT3 pathway suppressed GP73 expression. These results suggest a multipoint loop of c-Myc/GP73/STAT3, rather than a unidirectional cascade regulation, in promoting HCC angiogenesis. To verify this hypothesis, we performed Western blot analysis, which showed that the expressions of GP73, pJAK2, STAT3, and pSTAT3-Ser^727^ were increased in MHCC97H-GP73-KD-cMyc-OE cells but decreased in Hep3B-GP73-OE-cMyc-KD cells (Fig. 7L and Fig. S7H), suggesting that c-Myc activated JAK2/STAT3 signaling independent of GP73. Moreover, the expression levels of GP73, pJAK2, STAT3, and pSTAT3-Ser^727^ were inhibited in MHCC97H (Fig. 7B and Fig. S7E) and Hep3B (Fig. 7L and Fig. S7H) cells after treatment with C188-9. Angiogenesis qPCR array results showed that MCP-1 and TPO were regulated by the c-Myc and JAK2/STAT3 pathways (Fig. S7I to N). These results demonstrated that c-Myc separately up-regulated GP73 and STAT3 and that STAT3, in turn, up-regulated GP73 for the creation of an internal positive feedback GP73-STAT3 loop.

## Discussion

The hypervascular nature of HCC contributes to the wide application of anti-angiogenic treatment regimens, yielding a unique ORR in advanced HCC [27]. Nonetheless, emerging clinical trials have evidently indicated that substantial improvements in disease suppression via the inhibition of VEGF/VEGFR and PDGF/PDGFR signals for destabilizing tumor vasculature and simultaneously inducing cell cycle arrest and apoptosis [28] remain difficult to achieve [29]. HCC progression appears to be an integral result of varied regulation induced by multiple pathways and interactive networks, impeding successful antitumor improvements through the suppression of a single target.

GP73 is an important component of the Golgi complex, which plays crucial physiological roles in the sorting and modification of cargo proteins in the rough endoplasmic reticulum, as well as in protein transportation through the Golgi apparatus, facilitating proteins into specific subcellular location or extracellular secretion [30,31]. Elevated serum GP73 levels have been shown to be an indicator for the alerted early diagnosis of, and a marker for progression in, multiple neoplastic hematologic [32] and solid tumors [33–38]. As a novel HCC marker, ectopic GP73 expression strongly correlates with rapid disease progression and an extremely poor prognosis [39]. A previous study, using public databases, revealed that GP73 drove comprehensive pro-angiogenic behaviors in HCC [17]. In the present study, we elucidated the roles and mechanisms of overexpressed GP73-HCC cells in the induction of tumor angiogenesis and found positive correlations between serum GP73 levels and the ORR of the anti-angiogenesis regimen among patients with HCC. GP73 expression was coordinated with the specific vascular endothelial cell marker CD34 in HCC tissues, indicating that GP73 is a potential anti-angiogenic target.

Based on the single-cell spatial transcriptomic landscape of vasculogenic etiology from different stages of HCC, we analyzed pro-angiogenic signal alterations in temporal and spatial dimensions that occurred during HCC progression, and found strong cellular communications between overexpressed GP73 HCC and endothelial cells with high pro-angiogenesis potential via multiple receptor–ligand interactions at the single-cell level, which was consistent with the validation performed by expanding HCC single-cell samples from public databases. Of note, this malignant-endothelial cell interaction showed strong spatial distribution in regions with overexpressed GP73. Therefore, our single-cell spatial transcriptomic profiling revealed that GP73 may be a novel vascular niche gene that modulates tumor vascular ecosystems through a GP73-mediated network in HCC cells.

Angiogenesis is the process by which new blood vessels develop from existing capillaries or venules and eventually create mature vasculature from budding or nonbudding forms. This process includes the activation, proliferation, and migration of vascular endothelial cells, which are important signs of angiogenesis and the necessary early steps of the angiogenic cascade [40,41]. The basic principle of angiogenesis experiments is that endothelial cells retain their ability to divide and migrate in response to angiogenic signals; therefore, HUVEC proliferation and migration experiments have been widely used to simulate and reflect the process of tumor angiogenesis [42,43]. In the present study, in vitro and in vivo experiments demonstrated that GP73 overexpression in HCC cells stimulated tumor angiogenesis, which, under physiological conditions, is strictly regulated by various cytokines and is crucial for embryonic development, trauma repair, reproduction, and menstrual cycles [44]. Nonetheless, tumor angiogenesis appears to be the result of multilevel molecular interaction that contributes to the uncontrolled stimulation of abundant pro-angiogenic factor secretion and endothelial cell proliferation and migration under microenvironmental stress [40]. At the molecular level, we demonstrated that the overexpression of GP73 in HCC cells could promote the secretion of the pro-angiogenic factors CXCL5, MCP-1, and TPO, which are directly correlated with vasculature development during tumor progression. Additionally, Liu et al. [45] demonstrated the elevated expression of GP73 in HUVECs, although the modulation of GP73 had no influence on the proliferation of HUVECs. The results of the present study, however, demonstrated that the up- or down-regulation of GP73 in HCC cells significantly enhanced or inhibited the growth of HUVECs, respectively. Taken together, these studies elucidated that GP73 in HCC cells, through intercellular communication, activates pro-angiogenic signals via a nonautocrine mechanism while emphasizing the core roles of HCC cells in remodeling the tumor microenvironment. To the authors’ knowledge, the present study is the first to elucidate the pro-angiogenic role of GP73 in HCC.

We then explored the GP73 downstream signaling cascades and found increased JAK2/STAT3 and angiogenesis scores in overexpressed GP73 HCC cell clusters overexpressing the single-cell transcriptomic data. The JAK/STAT pathway is important for the penetration of extracellular signals across the cell membrane directly into the nucleus and responds to transcriptional reactions in DNA [46]. Diverse cellular stresses contribute to the uninterrupted abnormal activation of JAK/STAT signaling in HCC to regulate angiogenesis, immunity, and metabolic reprogramming [47]. From the single-cell spatial map, we obtained a specific GP73-mediated pro-angiogenic landscape presenting strong JAK2/STAT signaling activation in vibrant angiogenesis spatial blocks, suggesting that GP73 activates the JAK2/STAT3 pathway in promoting HCC angiogenesis. Next, in vivo and in vitro studies demonstrated that overexpressed GP73 induced the activation of JAK2/STAT3 signaling, and the loss of STAT3 function manifested the inhibitory effect of down-regulation of GP73 on HCC angiogenesis. These results are consistent with the observed effects of STAT3 silencing in HCC [48,49], suggesting that JAK2/STAT3 signaling is essential for GP73-induced angiogenesis.

Ye and colleagues [50] demonstrated the indirect modulation of GP73 to enhance the phosphorylation of STAT3-Tyr^705^ by promoting the phosphorylation of EGFR. In the present study, we elucidated the novel mechanisms by which GP73 up-regulates STAT3, independently of JAK2, through dual regulation. GP73 directly binds to STAT3 and stimulates STAT3-Ser^727^ phosphorylation. However, the down-regulation of GP73 and STAT3 did not completely unrestrict HUVEC growth and migration, implying that additional regulation is involved. From single-cell spatial transcriptomic data, we found that activated ERS and JAK2/STAT3 signals coupled with GP73 overexpression, and demonstrated that GP73 enhanced GRP78-induced ERS to stimulate STAT3-Ser^727^ phosphorylation. A previous study [15] demonstrated the importance of GP73 in regulating the tumor microenvironment, in part by interacting with GRP78 to enhance ERS signaling in neighboring macrophages. In the present study, we demonstrated the contribution of GP73 to the amplification and transmission of GRP78-induced ERS signals for the activation of JAK2/STAT3 signals in the processing of tumor vasculature.

For exploring the potential upstream activators of GP73, through the single-cell spatial transcriptomic data, we analyzed the gene expression profiles of overexpressed GP73 HCC subsets, which closely communicated with endothelial cell subsets. We found that a series of GP73-related pro-angiogenic genes that mediated histone modification, lactate metabolism, c-MYC, JAK2–STAT3, and ERS pathways were activated, and new cross-talk among these signals was thought to be involved in an interactive GP73-mediated pro-angiogenetic network. Lactate has been proven to be a multifunctional signaling molecule, in addition to being a metabolite [51], exerting novel roles in stimulating the lactylation of histone lysine residues [52] and participating in multiple disease progressions as driver of oncogenes [53,54]. Contributions of histone lactylation on HCC development have been recognized [55], although in the present study we demonstrated that histone lactylation could stimulate GP73 overexpression, and identified H3K4, H3K18, H4K5, and H4K as the lactylation sites on core histones [53], in particular, increased histone lactylation in H3K18 is required for the up-regulation of GP73. Zhao and colleagues [52] characterized P300 as the “writer” enzyme of histone lactylation. Similarly, we demonstrated that P300 is a potential “writer” of lactylation in GP73 and that knockdown of P300 inhibited GP73 expression.

c-Myc is a classic nuclear protein-like proto-oncogene and a TF that binds to chromosomal DNA and widely supports the transcription and amplification of abundant human oncogenes [56]. Under hypoxic stress, c-MYC enhances GP73 transcriptional activity to promote epithelial-mesenchymal transition (EMT)-related metastasis in HCC [57]. In the present study, we demonstrated that under normoxic conditions, c-MYC also strengthened GP73 transcriptional activity by binding to its promoter. In vitro studies showed the effect of c-Myc-mediated transactivation of GP73 on HCC angiogenesis. Additionally, in vivo studies demonstrated that the c-MYC/GP73/STAT3 cascade signals function in the central pro-angiogenic effect of GP73 in HCC.

Interestingly, despite the down-regulation of GP73 and STAT3, HUVECs did not show complete restriction of proliferation and migration, implying that additional mechanisms are involved in HCC angiogenesis. The present study elucidated an intricate multipoint loop of c-Myc/GP73/STAT3 rather than a unidirectional cascade regulation. In particular, STAT3 could be additionally activated by c-Myc regardless of GP73 and, in turn, feedback up-regulates GP73. Therefore, the rapid expansion of HCC cells possesses this positive loop for the reinforcement of inducible angiogenesis and better microenvironmental adaptation in processing the HCC vasculature.

Moreover, although we generated and validated a serum GP73-based prognostic nomogram for determining the 6-month and 1-year survival of patients with HCC treated with anti-angiogenic agents, we demonstrated the favorable anti-angiogenic potential of GP73 in clinical practice.

It is worth noting that different heterogeneous populations may include different oncogenic drivers. Heterogeneity widely exists at multiple levels, including in the genome, transcriptome, and microenvironment. Here, considerable heterogeneity in gene expression within HCC and endothelial cells was identified using single-cell transcriptomic data. It is necessary to comprehensively analyze heterogeneity through multi-omics data, extract commonalities, correlate the findings with the clinicopathological characteristics of patients with HCC, and conduct in vivo and in vitro experiments to better elucidate the angiogenic manifestations of HCC. Therefore, on the basis of discovering the positive correlation of serum GP73 expression level with HCC response to the anti-angiogenesis treatment, the present study was the first to construct single-cell spatial transcriptomic landscape of HCC vasculogenic etiology and characterized a GP73-mediated pro-angiogenic network. In vitro and in vivo experiments demonstrated that GP73 is the hub gene coordinated with c-MYC, lactate/histone modification, the JAK2–STAT3 pathway, and ERS signals through interactive cascade regulations for stimulating HCC vasculature. Altogether, our study, consistent with multidimensional evidence, elucidated the significance of GP73 in the regulation of HCC angiogenesis.

## Methods

### Study population and tissues

Patients who received lenvatinib for the treatment of unresectable HCC from January 2018 through December 2022 at Guangxi Medical University Cancer Hospital were considered for inclusion in the present study. The inclusion criteria were as follows: (a) Eastern Cooperative Oncology Group Performance Status (ECOG PS) score, 0 to 1; (b) Child–Pugh liver function score, 5 to 7; (c) clinical or pathological diagnosis of HCC using the Barcelona Clinical Liver Cancer (BCLC) standard; (d) received ≥2 cycles of lenvatinib; (e) without other simultaneous malignancies; (f) without severe renal, cerebral, or cardiopulmonary dysfunction or failure; and (g) without grade III to IV adverse events leading to the discontinuation of treatment. Tumor response was assessed using the modified Response Evaluation Criteria in Solid Tumors 1.1 (mRECIST1.1) every 4 or 8 weeks through the follow-up deadline of August 2023. A 2-ml blood sample was obtained from each patient prior to the initiation of treatment with lenvatinib. After centrifugation, the serum was aliquoted and stored at −80 °C to be used for serum CP73 determination. The clinicopathological characteristics of the patients are shown in Table 1.

In addition, a total of 61 resected HCC samples were obtained from 61 patients who underwent hepatectomy as their initial treatment, for the IHC examination of GP73 and CD34. The clinicopathological characteristics of the patients are presented in Table S1.

### Samples for single-cell spatial RNA sequencing

Six HCC samples were collected from 6 patients who underwent hepatectomy as their initial treatment, and a noncarcinoma liver tissue was obtained from one patient (control donor) who underwent surgical resection for hepatic hemangioma and was used as the control at Guangxi Medical University Cancer Hospital. The tissue samples were subjected to single-cell and spatial RNA sequencing. The clinicopathological characteristics of the patients are presented in Table S2.

### Single-cell RNA sequencing

Samples were prepared for the creation of a complementary DNA (cDNA) library using a 10× Genomics Single Cell 3’v3.1 kit (catalog no. PN-1000269, 10× Genomics, California, USA) following the manufacturer’s instructions [58]. In brief, using microfluidic technology, single cells and the reagents required for the reaction were wrapped in gel bead in emulsion (GEM) droplets together with glue beads with a cell tag sequence (cell barcode) on the chip, and the GEM droplets containing the cells were collected. The lysed cells and released mRNA were combined in the GEM droplets with the cell barcode primer on the beads to complete the reverse transcription reaction. When the GEMs were disrupted, the cDNA was recovered and enriched by PCR amplification, and a cDNA library was thus constructed. The insert fragment size of cDNA was detected using a Qseq400 biological analyzer (Qsep400, Taiwan, China) and qualified with the peak type as single and without a hetero-peak, splice, or primer dimer. The cDNA products and library concentrations were determined using a Qubit4.0 fluorescence quantitative instrument, and the Illumina NovaSeq 6000 platform was used to sequence the sample libraries. The original image files acquired offline were converted into sequenced reads by CASAVA through base calling and stored in the fastq format. Sequencing results were quantified and analyzed using CellRanger, an official 10× Genomics software.

### Spatial transcriptome sequencing

Tissues were obtained from patients and were gently washed with cold 1× phosphate-buffered saline (PBS), stored at −80 °C, and formalin-fixed paraffin-embedded (FFPE) before cryosectioning for RNA extraction. Each tumor cryosection was cut in 5-μm-thick slices using the cryostat (Leica CM1950, Germany), dewaxed with xylene (214736, Sigma-Aldrich), and stained with hematoxylin and eosin (H&E). After visualizing and scanning the whole slide, decrosslinking was performed using a tris-ethylenediaminetetraacetic acid (TE) buffer to release the RNA. An RNA integrity number (RIN) value ≥ 7, for a 28*S*/18*S* ratio of 1.8 to 2.0, indicates that the extracted RNA has good integrity without basic degradation. Tissue optimization was performed to test whether the samples were suitable for spatial transcription experiments to determine the optimal penetration time. Tissue permeabilization and mRNA reverse transcription were performed to create cDNA, followed by the synthesis and denaturation of the second strand of the cDNA, in accordance with the permeabilization time established by tissue optimization. Using qPCR, cDNA amplification cycles were identified, and the cDNA was purified and quality-checked, from which a gene expression library was constructed. The Illumina NovaSeq6000 was used to sequence the qualified visible spatial gene expression library. The original image files obtained offline were converted into sequenced reads by CASAVA through base calling and stored in fastq format. SpaceRanger, an official 10× Genomics software, was used for data comparison, gene quantification, and site identification.

### Single-cell RNA sequencing data processing

The R package Seurat (version 4.1.1) was used for quality control and bioinformatic analyses [59]. Quality filtering was performed to remove cells with mitochondrial gene counts >30%, expressed genes <300 or >7,000, or unique molecular identifiers (UMIs) <1,000. The gene expression matrices were log-normalized by the total expression and multiplied by a scale factor (10,000 by default) for each of the remaining cells. Linear dimensional reduction [principal components analysis (PCA)] was performed on the scaled data based on highly variable genes (top 2,000). To cluster the cells, the first 20 principal components were used to construct a K-nearest neighbor (KNN) graph using the “FindNeighbors” function in Seurat. The clusters were then identified through the Louvain algorithm on the KNN graph using “FindClusters” function in Seurat, in which the resolution parameter was set as 0.5. Nonlinear dimensional reduction was performed using UMAP software to visualize the single-cell RNA (scRNA) dataset. The cell identity of each cluster was then determined based on the expression of known marker genes.

### Spatial transcriptomic data analysis

For the gene spot matrices generated by Space Ranger, the R package Seurat (version 4.1.1) was used for basic downstream analysis and visualization [59,60]. Each section of the expression matrices from each patient was combined and underwent normalization with the “SCTransform” function in Seurat. Next, highly variable genes were identified and underwent PCA. The clusters were identified through the Louvain algorithm on the KNN graph using the “FindClusters” function in Seurat. Individual observations using spatial transcriptomic technologies may reveal contributions from several cells, making it difficult to identify cell type-specific geographical patterns of localization and expression. We identified the spatial transcriptomic cell types using the R package Spacexr (v3.16.1) [61], using a computational technique known as robust cell-type decomposition (RCTD), which decomposes cell-type mixtures while accounting for variations in sequencing technology using scRNA sequencing. The “AddModuleScore” method [62] was utilized to generate the gene signature scores for a single location.

### Cell culture

MHCC97H and Hep3B cells, as well as human umbilical vein endothelial cells (HUVECs) were procured from the Shanghai Institute of Biological Sciences, Chinese Academy of Sciences (Shanghai, China) and cultured in Dulbecco’s modified Eagle’s medium (DMEM; Invitrogen, CA, USA) supplemented with 10% fetal bovine serum (FBS; Gibco, Thermo Fisher Scientific, Massachusetts, USA) and 1% penicillin–streptomycin (Gibco) at 37 °C under 5% CO_2_. The medium was replaced every 3 to 4 d, and the cells were passaged with 0.25% trypsin (Hyclone, Logan, UT, USA).

### Mice orthotopic transplanted liver tumor model

Specific pathogen-free (SPF)-grade athymic BALB/C nude mice (5 to 6 weeks; 20 to 22 g body weight) were obtained from the Shanghai Laboratory Animal Center (SLAC; Shanghai, China) and maintained in an SPF environment with free access to food and water. The HCC cells were digested into cell suspensions at a concentration of 1 × 10^7^ cells/ml. Anesthetized nude mice were disinfected with 75% alcohol and then subcutaneously inoculated with 200 μl of cell suspension in the middle of the right axilla. Approximately 10 d later, when the subcutaneous tumor grew to 1 cm^3^, the mice were euthanized via cervical dislocation. The tumor tissue was removed, separated into 1 mm^3^ cubes under sterile conditions, and stored in saline for orthotopic liver transplantation.

Athymic BALB/C nude mice (6 to 8 weeks; 22 to 28 g body weight) were anesthetized under aseptic conditions, after which an incision was made along the abdominal white line, and a prefabricated tumor was embedded into the liver and blocked with a gelatin sponge to prevent tumor mass and liver bleeding. The wound was closed with sutures after ensuring that there was no bleeding or complications. Three weeks later, the mice were injected with DiR liposomes (D4006, Amresco, USA) to monitor tumor growth and metastasis, and imaged using Living Image v4.2 software (Perkin Elmer). The mice were then euthanized via cervical dislocation, and the liver tumor tissues were collected for volume measurement, IHC staining, and cell immunofluorescence experiments.

The protocol for the present study was approved by the ethics committee of Guangxi Medical University (no. KY-2023-290; File S1) and was performed in accordance with the Animal Research: Reporting of In Vivo Experiments (ARRIVE 2.0) guidelines.

### ChIP-qPCR assay

The chromatin immunoprecipitation-qPCR (ChIP-qPCR) assay was performed using a SimpleChIP Enzymatic Chromatin IP kit (catalog no. 9003, Cell Signaling Technology, USA) following the manufacturer’s protocol. In brief, cells were cross-linked with 1% formaldehyde (164187-25G, Sigma) at room temperature for 10 min, after which the reaction was stopped with glycine. The cells were then washed 3 times with PBS and underwent cell lysis and nuclear protein separation using a lysis buffer. Nuclear lysates were sonicated to shear chromatin to an average length ranging from 200 to 500 bp followed by immunoprecipitation at 4 °C for 16 h using immunoglobulin G (IgG; 2729, Cell Signaling Technology, USA) or c-Myc antibody (9402, Cell Signaling Technology, USA). Real-time PCR (RT-PCR) was used to detect DNA fragment enrichment at the GP73 promoter binding sites.

### Statistical analysis

Data were analyzed using R software (v4.0.2) [63] and visualized using the R package ggplot2 [v3.3.5] [64]. Heatmaps were plotted using the “pheatmap” package (v1.0.12) in R for visualization [65]. Statistical analyses were conducted using SPSS23.0 (IBM Corp., Armonk, NY, USA) and GraphPad Prism 8.0 (GraphPad Software Inc., San Diego, CA, USA). All experiments were performed in triplicate, and data are presented as mean ± SD. Student’s *t* test was used to analyze differences between 2 groups, and one-way analysis of variance (ANOVA) was used to compare the differences among 3 or more groups. A *P* value of <0.05 was considered statistically significant.

### Data availability

Additional information on the materials and methods is included in the Supplementary Materials.

## Data Availability

All patients included in the present study consented to the sequencing and experimental protocol carried out for all research procedures, and approval was provided by the Ethics Review Committee of Guangxi Medical University Cancer Hospital (LW2022040). The animal experiments were reviewed and approved by the Animal Research Ethics Committee of Guangxi Medical University (ethics file ID: KY-2023-290) and performed in compliance with the ARRIVE guidelines.

## Data Availability

The accession number for the processed expression data of scRNA-seq, ST-seq reported in this paper is HRA002748 (https://ngdc.cncb.ac.cn/gsa-human/browse/HRA002748). Further information and request for reagents should be directed to and will be fulfilled by the lead contact, R.L. (email: liangrong@gxmu.edu.cn).

## Supplementary Materials

Supplementary 1 Supplementary Methods Supplementary Results Figs. S1 to S7 Tables S1 to S9 Files S1 to S4

## References

[1] Sung H, Ferlay J, Siegel RL, Laversanne M, Soerjomataram I, Jemal A, Bray F. Global cancer statistics 2020: GLOBOCAN estimates of incidence and mortality worldwide for 36 cancers in 185 countries. CA Cancer J Clin. 2021;71(3):209–249. 33538338 10.3322/caac.21660

[2] Hanahan D. Hallmarks of cancer: New dimensions. Cancer Discov. 2022;12(1):31–46. 35022204 10.1158/2159-8290.CD-21-1059

[3] Jiang X, Wang J, Deng X, Xiong F, Zhang S, Gong Z, Li X, Cao K, Deng H, He Y, et al. The role of microenvironment in tumor angiogenesis. J Exp Clin Cancer Res. 2020;39(1):204. 32993787 10.1186/s13046-020-01709-5 PMC7526376

[4] Tzeng H-T, Huang Y-J. Tumor vasculature as an emerging pharmacological target to promote anti-tumor immunity. Int J Mol Sci. 2023;24(5):4422. 36901858 10.3390/ijms24054422 PMC10002465

[5] Liu G, Chen T, Ding Z, Wang Y, Wei Y, Wei X. Inhibition of FGF-FGFR and VEGF-VEGFR signalling in cancer treatment. Cell Prolif. 2021;54(4): Article e13009. 33655556 10.1111/cpr.13009 PMC8016646

[6] Bergers G, Hanahan D. Modes of resistance to anti-angiogenic therapy. Nat Rev Cancer. 2008;8(8):592–603. 18650835 10.1038/nrc2442 PMC2874834

[7] Bachert C, Fimmel C, Linstedt AD. Endosomal trafficking and proprotein convertase cleavage of cis Golgi protein GP73 produces marker for hepatocellular carcinoma. Traffic. 2007;8(10):1415–1423. 17662025 10.1111/j.1600-0854.2007.00621.x

[8] Yang X, Fan X, Feng J, Fan T, Li J, Huang L, Wan L, Yang H, Li H, Gong J, et al. GP73 blockade alleviates abnormal glucose homeostasis in diabetic mice. J Mol Endocrinol. 2023;70(2): Article e220103. 36394986 10.1530/JME-22-0103

[9] Gatselis NK, Tornai T, Shums Z, Zachou K, Saitis A, Gabeta S, Albesa R, Norman GL, Papp M, Dalekos GN. Golgi protein-73: A biomarker for assessing cirrhosis and prognosis of liver disease patients. World J Gastroenterol. 2020;26(34):5130–5145. 32982114 10.3748/wjg.v26.i34.5130 PMC7495033

[10] Wang W, Wei C. Advances in the early diagnosis of hepatocellular carcinoma. Genes Dis. 2020;7(3):308–319. 32884985 10.1016/j.gendis.2020.01.014 PMC7452544

[11] Sai W-L, Yao M, Shen S-J, Zheng W-J, Sun J-Y, Wu M-N, Wang L, Yao DF. Dynamic expression of hepatic GP73 mRNA and protein and circulating GP73 during hepatocytes malignant transformation. Hepatobiliary Pancreat Dis Int. 2020;19(5):449–454. 32171652 10.1016/j.hbpd.2020.02.009

[12] Chen X, Wang Y, Tao J, Shi Y, Gai X, Huang F, Ma Q, Zhou Z, Chen H, Zhang H, et al. mTORC1 up-regulates GP73 to promote proliferation and migration of hepatocellular carcinoma cells and growth of xenograft tumors in mice. Gastroenterology. 2015;149(3):741–752.e14. 25980751 10.1053/j.gastro.2015.05.005

[13] Ye Q-H, Zhu W-W, Zhang J-B, Qin Y, Lu M, Lin G-L, Guo L, Zhang B, Lin ZH, Roessler S, et al. GOLM1 modulates EGFR/RTK cell-surface recycling to drive hepatocellular carcinoma metastasis. Cancer Cell. 2016;30(3):444–458. 27569582 10.1016/j.ccell.2016.07.017 PMC5021625

[14] Shao W-Q, Zhu W-W, Luo M-J, Fan M-H, Li Q, Wang S-H, Lin ZF, Zhao J, Zheng Y, Dong QZ, et al. Cholesterol suppresses GOLM1-dependent selective autophagy of RTKs in hepatocellular carcinoma. Cell Rep. 2022;39(3): Article 110712. 35443161 10.1016/j.celrep.2022.110712

[15] Wei C, Yang X, Liu N, Geng J, Tai Y, Sun Z, Mei G, Zhou P, Peng Y, Wang C, et al. Tumor microenvironment regulation by the endoplasmic reticulum stress transmission mediator Golgi protein 73 in mice. Hepatology. 2019;70(3):851–870. 30723919 10.1002/hep.30549

[16] Chen J, Lin Z, Liu L, Zhang R, Geng Y, Fan M, Zhu W, Lu M, Lu L, Jia H, et al. GOLM1 exacerbates CD8+ T cell suppression in hepatocellular carcinoma by promoting exosomal PD-L1 transport into tumor-associated macrophages. Signal Transduct Target Ther. 2021;6(1):397. 34795203 10.1038/s41392-021-00784-0 PMC8602261

[17] Lin Y, He Z, Gao X, Lu L, Lu C, Huang J, Luo M, Ye J, Liang R. Integrative analysis reveals the potential role and prognostic value of GOLM1 in hepatocellular carcinoma. Oxidative Med Cell Longev. 2022;2022:8284500. 10.1155/2022/8284500 PMC9535134 36211823

[18] Liu Z, Sun D, Wang C. Evaluation of cell-cell interaction methods by integrating single-cell RNA sequencing data with spatial information. Genome Biol. 2022;23:218. 36253792 10.1186/s13059-022-02783-y PMC9575221

[19] Ye J-Z, Yan S-M, Yuan C-L, Wu H-N, Zhang J-Y, Liu Z-H, Li YQ, Luo XL, Lin Y, Liang R. GP73 level determines chemotherapeutic resistance in human hepatocellular carcinoma cells. J Cancer. 2018;9(2):415–423. 29344288 10.7150/jca.19185 PMC5771349

[20] Liao Y, Wang C, Yang Z, Liu W, Yuan Y, Li K, Zhang Y, Wang Y, Shi Y, Qiu Y, et al. Dysregulated Sp1/miR-130b-3p/HOXA5 axis contributes to tumor angiogenesis and progression of hepatocellular carcinoma. Theranostics. 2020;10(12):5209–5224. 32373208 10.7150/thno.43640 PMC7196310

[21] Chen C, Xu Z-Q, Zong Y-P, Ou B-C, Shen X-H, Feng H, Zheng MH, Zhao JK, Lu AG. CXCL5 induces tumor angiogenesis via enhancing the expression of FOXD1 mediated by the AKT/NF-κB pathway in colorectal cancer. Cell Death Dis. 2019;10(3):178. 30792394 10.1038/s41419-019-1431-6 PMC6385313

[22] Feng H, Liu K, Shen X, Liang J, Wang C, Qiu W, Cheng X, Zhao R. Correction: Targeting tumor cell-derived CCL2 as a strategy to overcome bevacizumab resistance in ETV5+ colorectal cancer. Cell Death Dis. 2020;11(10):1006. 33230188 10.1038/s41419-020-03208-z PMC7683607

[23] Lamanuzzi A, Saltarella I, Frassanito MA, Ribatti D, Melaccio A, Desantis V, Solimando AG, Ria R, Vacca A. Thrombopoietin promotes angiogenesis and disease progression in patients with multiple myeloma. Am J Pathol. 2021;191(4):748–758. 33516787 10.1016/j.ajpath.2020.12.016

[24] Ahmadi SE, Rahimi S, Zarandi B, Chegeni R, Safa M. MYC: A multipurpose oncogene with prognostic and therapeutic implications in blood malignancies. J Hematol Oncol. 2021;14(1):121. 34372899 10.1186/s13045-021-01111-4 PMC8351444

[25] Li H, Qi Z, Niu Y, Yang Y, Li M, Pang Y, Liu M, Cheng X, Xu M, Wang Z. FBP1 regulates proliferation, metastasis, and chemoresistance by participating in C-MYC/STAT3 signaling axis in ovarian cancer. Oncogene. 2021;40(40):5938–5949. 34363022 10.1038/s41388-021-01957-5 PMC8497274

[26] Dong J, Cheng X-D, Zhang W-D, Qin J-J. Recent update on development of small-molecule STAT3 inhibitors for cancer therapy: From phosphorylation inhibition to protein degradation. J Med Chem. 2021;64(13):8884–8915. 34170703 10.1021/acs.jmedchem.1c00629

[27] Yao C, Wu S, Kong J, Sun Y, Bai Y, Zhu R, Li Z, Sun W, Zheng L. Angiogenesis in hepatocellular carcinoma: Mechanisms and anti-angiogenic therapies. Cancer Biol Med. 2023;20(1):25–43. 36647777 10.20892/j.issn.2095-3941.2022.0449 PMC9843448

[28] Yang M, Su Y, Wang Z, Du D, Wei S, Liao Z, Zhang Q, Zhao L, Zhang X, Han L, et al. C118P, a novel microtubule inhibitor with anti-angiogenic and vascular disrupting activities, exerts anti-tumor effects against hepatocellular carcinoma. Biochem Pharmacol. 2021;190: Article 114641. 34077738 10.1016/j.bcp.2021.114641

[29] Yang Z, Suda G, Maehara O, Ohara M, Yoda T, Sasaki T, Kohya R, Yoshida S, Hosoda S, Tokuchi Y, et al. Changes in serum growth factors during resistance to atezolizumab plus bevacizumab treatment in patients with unresectable hepatocellular carcinoma. Cancers. 2023;15(3):593. 36765554 10.3390/cancers15030593 PMC9913372

[30] Wang Y, Wan Y-JY. Golgi protein 73, hepatocellular carcinoma and other types of cancers. Liver Res. 2020;4(4):161–167. 33343966 10.1016/j.livres.2020.09.003 PMC7743997

[31] Liu Y, Hu X, Liu S, Zhou S, Chen Z, Jin H. Golgi phosphoprotein 73: The driver of epithelial-mesenchymal transition in cancer. Front Oncol. 2021;11: Article 783860. 34950590 10.3389/fonc.2021.783860 PMC8688837

[32] Zi Z, Du S, Zhang L, Wang Y, Ding L, Zhang C, Wang H, Pawlicki J, Cai Y, Yao Y, et al. Aberrant expression of GOLM1 protects ALK+ anaplastic large cell lymphoma from apoptosis by enhancing BCL-XL stability. Blood Adv. 2023;7:4049–4063. 36763539 10.1182/bloodadvances.2022008384 PMC10388734

[33] Ke MY, Wu XN, Zhang Y, Wang S, Lv Y, Dong J. Serum GP73 predicts posthepatectomy outcomes in patients with hepatocellular carcinoma. J Transl Med. 2019;17:140. 31046807 10.1186/s12967-019-1889-0 PMC6498666

[34] Li Z, Li Y, Wang X, Liang Y, Luo D, Han D, Li C, Chen T, Zhang H, Liu Y, et al. LINC01977 promotes breast cancer progression and chemoresistance to doxorubicin by targeting miR-212-3p/GOLM1 axis. Front Oncol. 2021;11: Article 657094. 33869063 10.3389/fonc.2021.657094 PMC8046671

[35] Wang B, Sun X, Huang K-J, Zhou L-S, Qiu Z-J. Long non-coding RNA TP73-AS1 promotes pancreatic cancer growth and metastasis through miRNA-128-3p/GOLM1 axis. World J Gastroenterol. 2021;27(17):1993–2014. 34007135 10.3748/wjg.v27.i17.1993 PMC8108040

[36] Song Q, He X, Xiong Y, Wang J, Zhang L, Leung EL-H, Li G. The functional landscape of Golgi membrane protein 1 (GOLM1) phosphoproteome reveal GOLM1 regulating P53 that promotes malignancy. Cell Death Discov. 2021;7(1):42. 33649292 10.1038/s41420-021-00422-2 PMC7921442

[37] Dang Y, Yu J, Zhao S, Jin L, Cao X, Wang Q. GOLM1 drives colorectal cancer metastasis by regulating myeloid-derived suppressor cells. J Cancer. 2021;12(23):7158–7166. 34729117 10.7150/jca.61567 PMC8558645

[38] Liang H, Ai-Jun J, Ji-Zong Z, Jian-Bo H, Liang Z, Yong-Xiang Y, Chen Y. Clinicopathological significance of miR-27b targeting Golgi protein 73 in patients with hepatocellular carcinoma. Anti-Cancer Drugs. 2019;30(2):186–194. 30418194 10.1097/CAD.0000000000000711

[39] Zhang J, Zhang M, Ma H, Song X, He L, Ye X, Li X. A meta-analysis of the prognostic significance of Golgi protein 73 in hepatocellular carcinoma in Chinese patients. Arch Med Sci. 2020;16(5):1104–1110. 32863999 10.5114/aoms.2019.83821 PMC7444708

[40] Liu Z-L, Chen H-H, Zheng L-L, Sun L-P, Shi L. Angiogenic signaling pathways and anti-angiogenic therapy for cancer. Signal Transduct Target Ther. 2023;8:198. 37169756 10.1038/s41392-023-01460-1 PMC10175505

[41] Kretschmer M, Rüdiger D, Zahler S. Mechanical aspects of angiogenesis. Cancers. 2021;13(19):4987. 34638470 10.3390/cancers13194987 PMC8508205

[42] Nowak-Sliwinska P, Alitalo K, Allen E, Anisimov A, Aplin AC, Auerbach R, Augustin HG, Bates DO, van Beijnum JR, Bender RHF, et al. Consensus guidelines for the use and interpretation of angiogenesis assays. Angiogenesis. 2018;21(3):425–532. 29766399 10.1007/s10456-018-9613-x PMC6237663

[43] Meng J, Liu Y, Han J, Tan Q, Chen S, Qiao K, Zhou H, Sun T, Yang C. Hsp90β promoted endothelial cell-dependent tumor angiogenesis in hepatocellular carcinoma. Mol Cancer. 2017;16(1):72. 28359326 10.1186/s12943-017-0640-9 PMC5374580

[44] Aspriţoiu VM, Stoica I, Bleotu C, Diaconu CC. Epigenetic regulation of angiogenesis in development and tumors progression: Potential implications for cancer treatment. Front Cell Dev Biol. 2021;9: Article 689962. 34552922 10.3389/fcell.2021.689962 PMC8451900

[45] Liu Y, Zou Z, Zhu B, Hu Z, Zeng P. CXCL10 decreases GP73 expression in hepatoma cells at the early stage of hepatitis C virus (HCV) infection. Int J Mol Sci. 2013;14(12):24230–24241. 24351813 10.3390/ijms141224230 PMC3876107

[46] Haselager MV, Bax D, Both D, De Boer F, Mackay S, Dubois J, Mellink C, Kater AP, Eldering E. JAK-STAT signalling shapes the NF-kappaB response in CLL towards venetoclax sensitivity or resistance via Bcl-XL. Mol Oncol. 2023;17:1112–1128. 36550750 10.1002/1878-0261.13364 PMC10257415

[47] Hin Tang JJ, Hao Thng DK, Lim JJ, Toh TB. JAK/STAT signaling in hepatocellular carcinoma. Hepat. Hepat Oncol. 2020;7(1):HEP18. 32273976 10.2217/hep-2020-0001 PMC7137178

[48] Chen Z, Han ZC. STAT3: A critical transcription activator in angiogenesis. Med Res Rev. 2008;28(2):185–200. 17457812 10.1002/med.20101

[49] Chen R-Y, Yen C-J, Liu Y-W, Guo C-G, Weng C-Y, Lai C-H, Wang JM, Lin YJ, Hung LY. CPAP promotes angiogenesis and metastasis by enhancing STAT3 activity. Cell Death Differ. 2020;27:1259–1273. 31511651 10.1038/s41418-019-0413-7 PMC7206147

[50] Yan J, Zhou B, Guo L, Chen Z, Zhang B, Liu S, Zhang W, Yu M, Xu Y, Xiao Y, et al. GOLM1 upregulates expression of PD-L1 through EGFR/STAT3 pathway in hepatocellular carcinoma. Am J Cancer Res. 2020;10(11):3705–3720. 33294262 PMC7716143

[51] Xu X, Peng Q, Jiang X, Tan S, Yang Y, Yang W, Han Y, Chen Y, Oyang L, Lin J, et al. Metabolic reprogramming and epigenetic modifications in cancer: From the impacts and mechanisms to the treatment potential. Exp Mol Med. 2023;55(7):1357–1370. 37394582 10.1038/s12276-023-01020-1 PMC10394076

[52] Zhang D, Tang Z, Huang H, Zhou G, Cui C, Weng Y, Liu W, Kim S, Lee S, Perez-Neut M, et al. Metabolic regulation of gene expression by histone lactylation. Nature. 2019;574:575–580. 31645732 10.1038/s41586-019-1678-1 PMC6818755

[53] Yu J, Chai P, Xie M, Ge S, Ruan J, Fan X, Jia R. Histone lactylation drives oncogenesis by facilitating m(6)A reader protein YTHDF2 expression in ocular melanoma. Genome Biol. 2021;22(1):85. 33726814 10.1186/s13059-021-02308-z PMC7962360

[54] Jiang J, Huang D, Jiang Y, Hou J, Tian M, Li J, Sun L, Zhang Y, Zhang T, Li Z, et al. Lactate modulates cellular metabolism through histone lactylation-mediated gene expression in non-small cell lung cancer. Front Oncol. 2021;11: Article 647559. 34150616 10.3389/fonc.2021.647559 PMC8208031

[55] Pan L, Feng F, Wu J, Fan S, Han J, Wang S, Yang L, Liu W, Wang C, Xu K. Demethylzeylasteral targets lactate by inhibiting histone lactylation to suppress the tumorigenicity of liver cancer stem cells. Pharmacol Res. 2022;181: Article 106270. 35605812 10.1016/j.phrs.2022.106270

[56] Beaulieu ME, Castillo F, Soucek L. Structural and biophysical insights into the function of the intrinsically disordered Myc oncoprotein. Cells. 2020;9(4):1038. 32331235 10.3390/cells9041038 PMC7226237

[57] Liu Y, Zhou S, Shi J, Zhang X, Shentu L, Chen Z, Zhou L. C-Myc transactivates GP73 and promotes metastasis of hepatocellular carcinoma cells through GP73-mediated MMP-7 trafficking in a mildly hypoxic microenvironment. Oncogenesis. 2019;8(10):58. 31591387 10.1038/s41389-019-0166-7 PMC6779757

[58] Zheng P, Zhang N, Ren D, Yu C, Zhao B, Zhang Y. Integrated spatial transcriptome and metabolism study reveals metabolic heterogeneity in human injured brain. Cell Rep Med. 2023;4(6): Article 101057. 37263268 10.1016/j.xcrm.2023.101057 PMC10313933

[59] Stuart T, Butler A, Hoffman P, Hafemeister C, Papalexi E, Mauck WM, Hao Y, Stoeckius M, Smibert P, Satija R. Comprehensive integration of single-cell data. Cell. 2019;177(7):1888–1902.e21. 31178118 10.1016/j.cell.2019.05.031 PMC6687398

[60] Butler A, Hoffman P, Smibert P, Papalexi E, Satija R. Integrating single-cell transcriptomic data across different conditions, technologies, and species. Nat Biotechnol. 2018;36(5):411–420. 29608179 10.1038/nbt.4096 PMC6700744

[61] Cable DM, Murray E, Shanmugam V, Zhang S, Zou LS, Diao M, Chen H, Macosko EZ, Irizarry RA, Chen F. Cell type-specific inference of differential expression in spatial transcriptomics. Nat Methods. 2022;19(9):1076–1087. 36050488 10.1038/s41592-022-01575-3 PMC10463137

[62] Wei W, Liu Y, Shen Y, Yang T, Dong Y, Han Z, Wang Y, Liu Z, Chai Y, Zhang M, et al. In situ tissue profile of rat trigeminal nerve in trigeminal neuralgia using spatial transcriptome sequencing. Int J Surg. 2024;110(3):1463–1474. 38270619 10.1097/JS9.0000000000001110 PMC10942187

[63] Yuan Y, Chen Y, Yao F, Zeng M, Xie Q, Shafiq M, Noman SM, Jiao X. Microbiomes and resistomes in biopsy tissue and intestinal lavage fluid of colorectal cancer. Front Cell Dev Biol. 2021;9: Article 736994. 34604238 10.3389/fcell.2021.736994 PMC8484797

[64] Gustavsson EK, Zhang D, Reynolds RH, Garcia-Ruiz S, Ryten M. ggtranscript: An R package for the visualization and interpretation of transcript isoforms using ggplot2. Bioinformatics. 2022;38(15):3844–3846. 35751589 10.1093/bioinformatics/btac409 PMC9344834

[65] Zhang X, Chao P, Zhang L, Xu L, Cui X, Wang S, Wusiman M, Jiang H, Lu C. Single-cell RNA and transcriptome sequencing profiles identify immune-associated key genes in the development of diabetic kidney disease. Front Immunol. 2023;14:1030198. 37063851 10.3389/fimmu.2023.1030198 PMC10091903

