# Supplementary material for: Integrating Single-Cell and Spatial Transcriptomics to Uncover and Elucidate GP73-Mediated Pro-Angiogenic Regulatory Networks in Hepatocellular Carcinoma
Source: Research (Wash D C). 2024 Jun 27;7:0387. doi: 10.34133/research.0387 (PMC11208919; doi:10.34133/research.0387)
Supplement: Supplementary 1 — Supplementary Methods Supplementary Results Figs. S1 to S7 Tables S1 to S9 Files S1 to S4 [file research.0387.f1.zip › Supplement Figure Legends.docx]

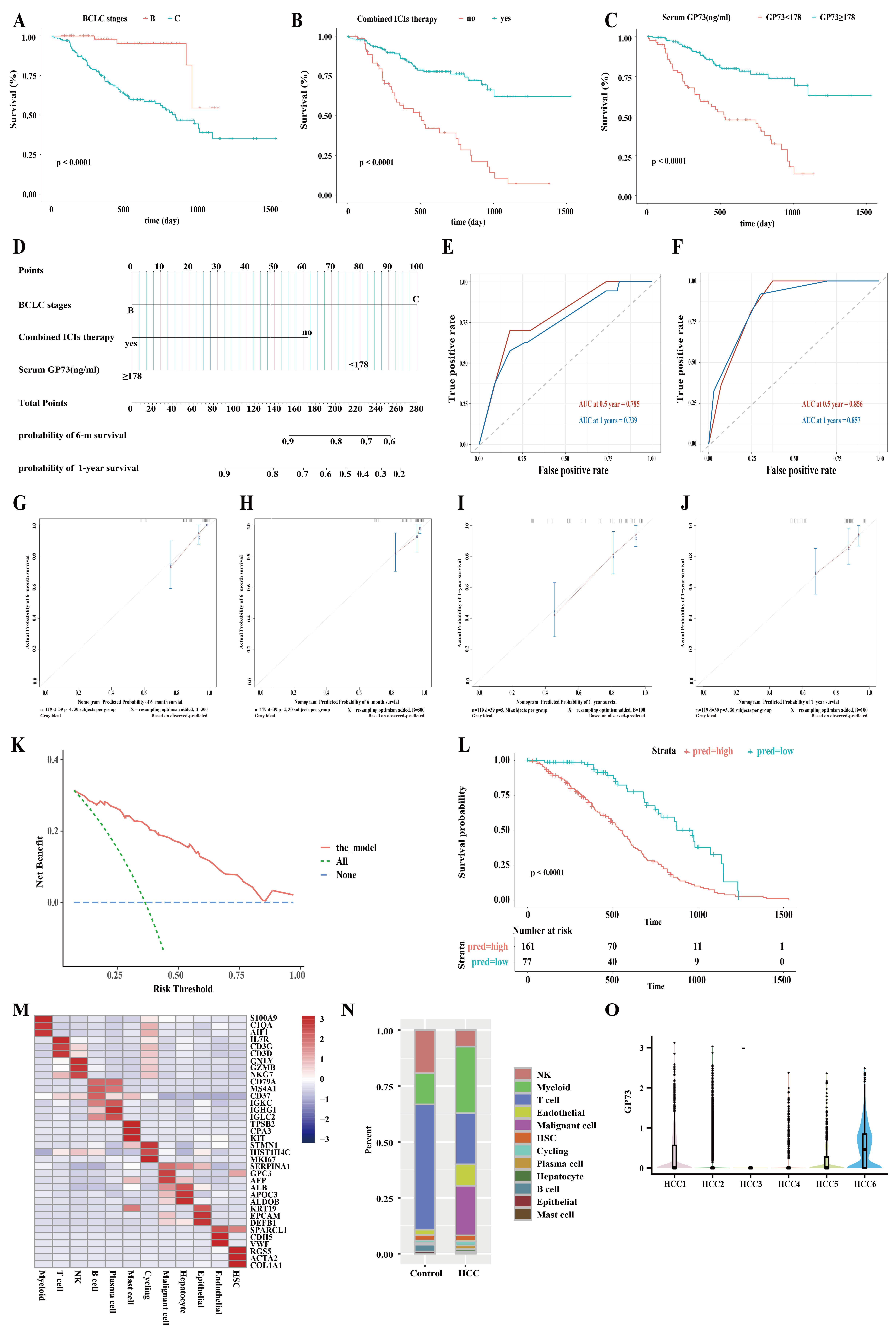


**Figure S1. Construction of a nomogram for determining the estimated survivals for HCC patients treated with anti-angiogenesis therapy and classification of the cell types and determination of GP73 expression in HCC tisuses by the single-cell transcriptomics**

**(A-C)** Kaplan-Meier curves for evaluating the overall survival in HCC patients treated with lenvatinib according to the independent indicators **(A)** BCLC stages, **(B)** combined ICIs treatment, and **(C)** serum GP73 levels. (**D)** Nomogram for predicting 6-month and 1-year OS in 238 HCC patients treated with lenvatinib. The ROC curves of nomogram for predicting 6-month and 1-year OS (**E**) in the training cohort and (**F**) in the validation cohort. The calibration curves of nomogram for predicting 6-month OS **(G)** in the training cohort and (**H**) in the validation cohort. The calibration curves of nomogram for predicting 1-year OS (**I**) in the training cohort and (**J**) in the validation cohort. **(K)** The DCA plot of nomogram for predicting 1-year OS in the entire cohort. **(L)** 1-year OS-risk stratification of the nomogram for HCC patients in the entire cohort. **(M)** Heatmap showing the expressions of specific markers corresponding to specific cell types. **(N)** Histogram indicating the proportions of different cells clusters in HCC and normal liver (control) tissues, with different colors codes denoting individual cell clusters. **(O)** Violin plot showing GP73 expressions in HCC tissues.

**Abbreviations:** HCC, hepatocellular carcinoma; BCLC, Barcelona Clinical Liver Cancer; ICI, immune checkpoint inhibitors; OS, overall survival; DCA, Decision Curve Analysis.


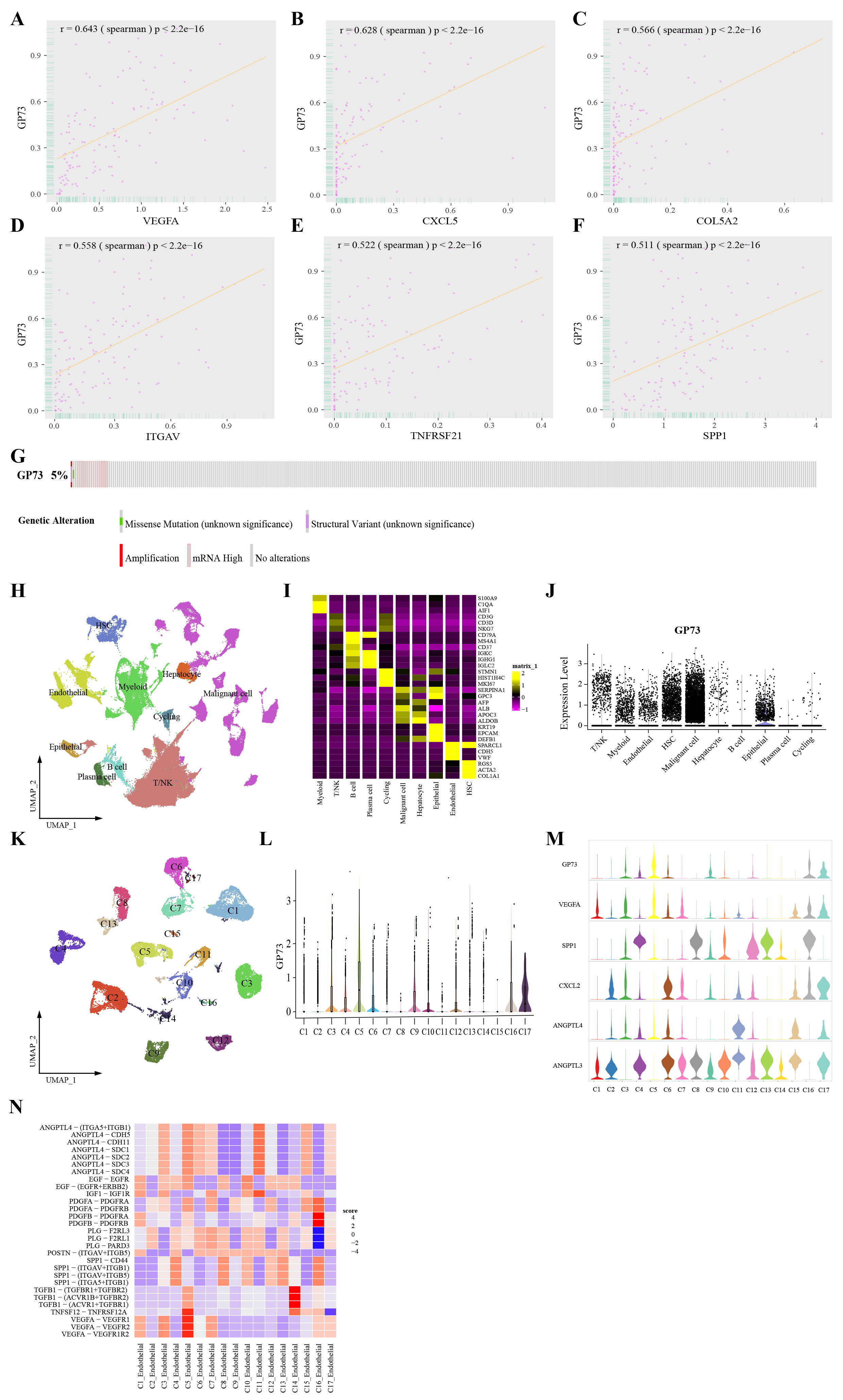


**Figure S2. Correlation analysis between GP73 and pro-angiogenic genes by using the Single-cell transcriptomics from GEO database**

**(A–F)** Scatter plot showing the correlation between GP73 and **(A)** VEGFA (Spearman’s r = 0.643, *P* = 0.000), **(B)** CXCL5 (Spearman’s r = 0.628, *P* = 0.000), **(C)** COL5A2 (Spearman’s r = 0.566, *P* = 0.000), **(D)** ITGAV (Spearman’s r = 0.558, *P* = 0.000), **(E)** TNFRSF21(Spearman’s r = 0.522, *P* = 0.000), and **(F)** SPP1 (Spearman’s r = 0.511, *P* = 0.000). **(G)** Genetic alteration analysis of GP73 (cBioPortal, <http://cbioportal.org/>, Liver Hepatocellular Carcinoma (TCGA, Firehose Legacy, 379 total samples), accessed on June 7th, 2023). **(H)** UMAP plot showing the single cell clusters identified by single-cell transcriptome from the GEO datasets, with different colors codes denoting individual cell clusters. **(I)** Heatmap showing the expressions of specific markers corresponding to specific cell types. **(J)** Scatter plot showing the GP73 expressions corresponding to specific cell types. **(K)** UMAP plot showing the HCC cell subclusters in GEO datasets, with different colors codes denoting different cell clusters. **(L)** Violin plot showing the GP73 expression in the HCC cell subclusters. **(M)** Violin plot showing the expressions of GP73 and pro-angiogenic ligands in different cells clusters. **(N)** Heatmap representing the receptor-ligand pairs in all the HCC cell clusters.

**Abbreviations:** GEO, Gene ontology; HCC, hepatocellular carcinoma.

**Figure S3. Construction and validation of over-expressed and knocked down GP73 in HCC cell lines**


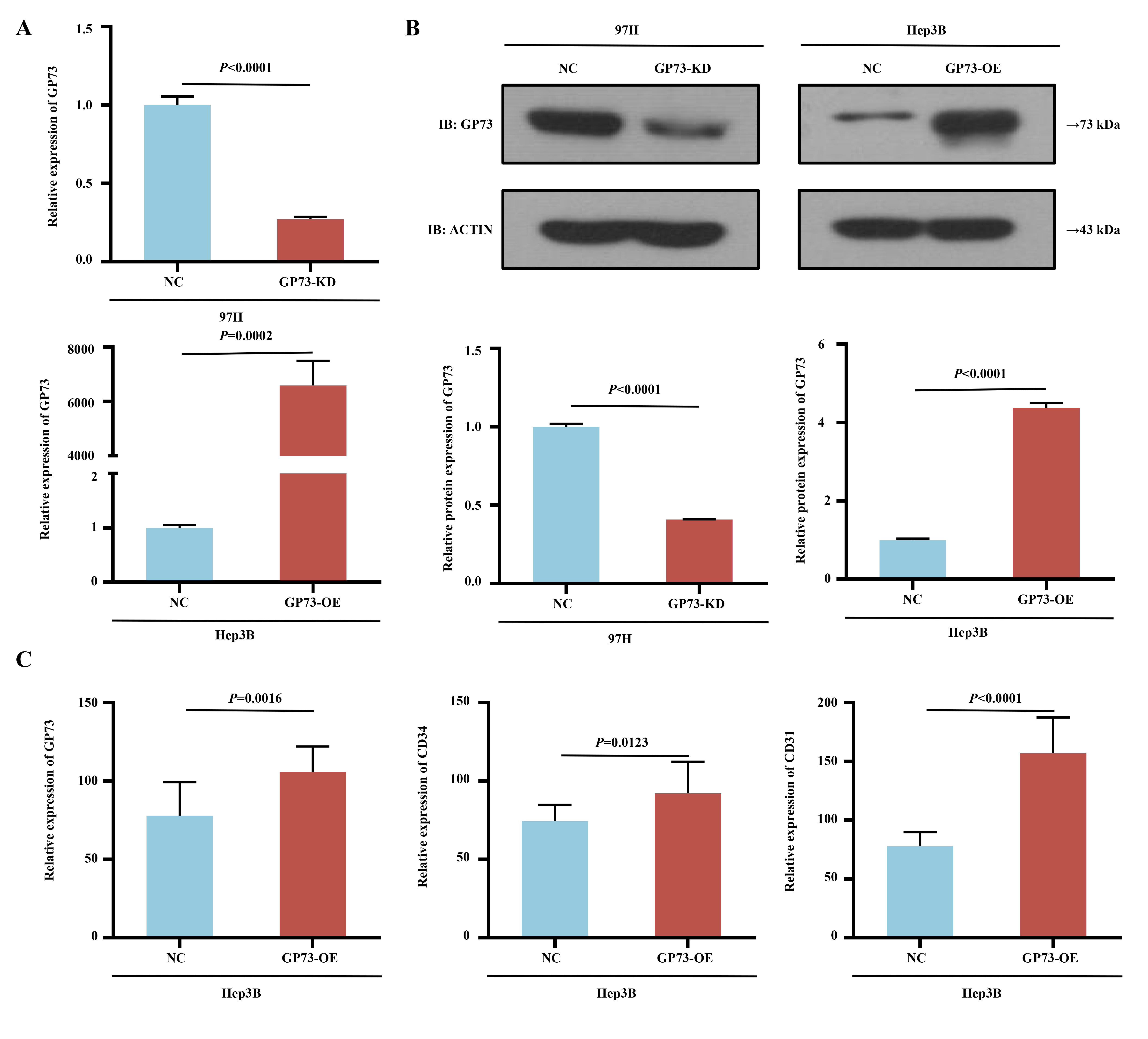


**(A)** RT-qPCR and **(B)** western blot detection for evaluating the efficiency of knocked down GP73 in MHCC97H cells and over-expressed GP73 in Hep3B cells. **(C)** Quantitative and statistical analyses of immunofluorescence staining intensities of GP73, CD34 and CD31 in Hep3B-GP73-OE and Hep3B-NC cells.

**Abbreviations:** GEO, Gene ontology; HCC, hepatocellular carcinoma.


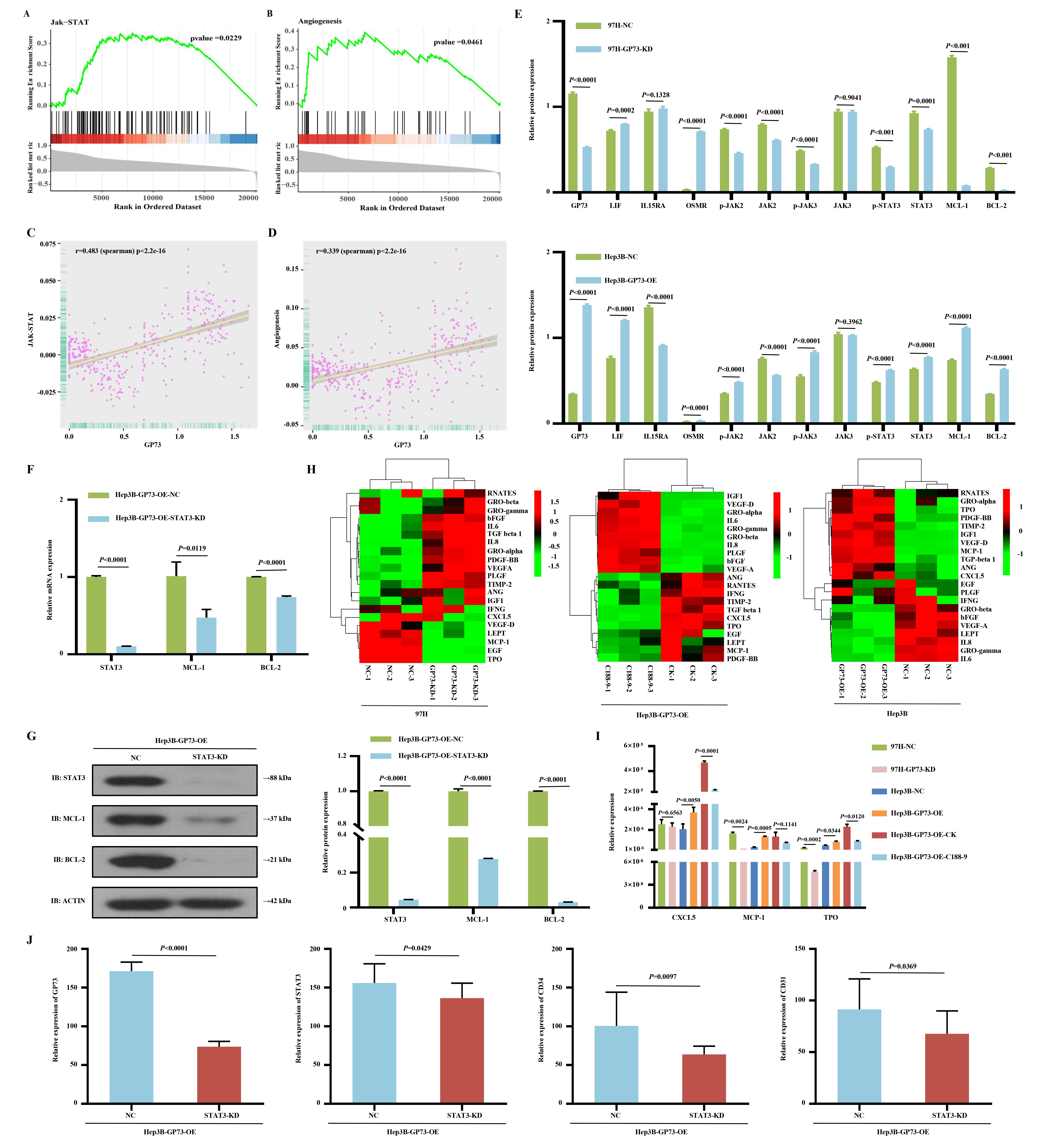


**Figure S4. GSEA analysis by using spatial single-cell transcriptomics to evaluate the correlation between GP73 gene and JAK2-STAT3 and angiogenesis pathways in HCC cell subclusters**

**(A–B)** GSEA analysis by using the single cell-spatial transcriptomics showing the GP73 expression was significant positively correlated with **(A)** JAK-STAT pathway and **(B)** angiogenesis pathway. **(C–D)** Correlation analysis of GP73 with **(C)** JAK-STAT pathway and **(D)** angiogenesis pathway scores in HCC cells clusters. **(E)** Quantitative analysis of protein expression levels of the core genes in JAK2/STAT3 pathway in MHCC97H-GP73-NC, MHCC97H-GP73-KD, Hep3B-GP73-NC and Hep3B-GP73-OE cells. **(F–G)** Quantitative and statistical analyses of **(F)** mRNA and **(G)** protein expressions of STAT3, MCL-1 and BCL-2 in Hep3B-GP73-OE and Hep3B-GP73-OE-STAT3-KD cells. **(H)** Heat map showing differential expressions of 21 key angiogenic factors among MHCC97H-NC, MHCC97H-GP73-KD, Hep3B-NC, Hep3B-GP73-OE, Hep3B-GP73-OE-C188-9 cells. C188-9 indicates the cells was treated with C188-9 (10 uM) for 24 hours. **(I)** Determination of mRNA expressions of pro-angiogenic factors CXCL5, MCP-1 and TPO in MHCC97H-NC, MHCC97H-GP73-KD, Hep3B-NC and Hep3B-GP73-OE, Hep3B-GP73-OE-C188-9 cells by angiogenesis qPCR Array. **(J)** Quantitative analysis of immunofluorescence staining intensities of GP73, STAT3, CD34 and CD31 in Hep3B-GP73-OE and Hep3B-GP73-OE-STAT3-KD cells.

**Abbreviations:** HCC, hepatocellular carcinoma; GSEA, gene set enrichment analysis


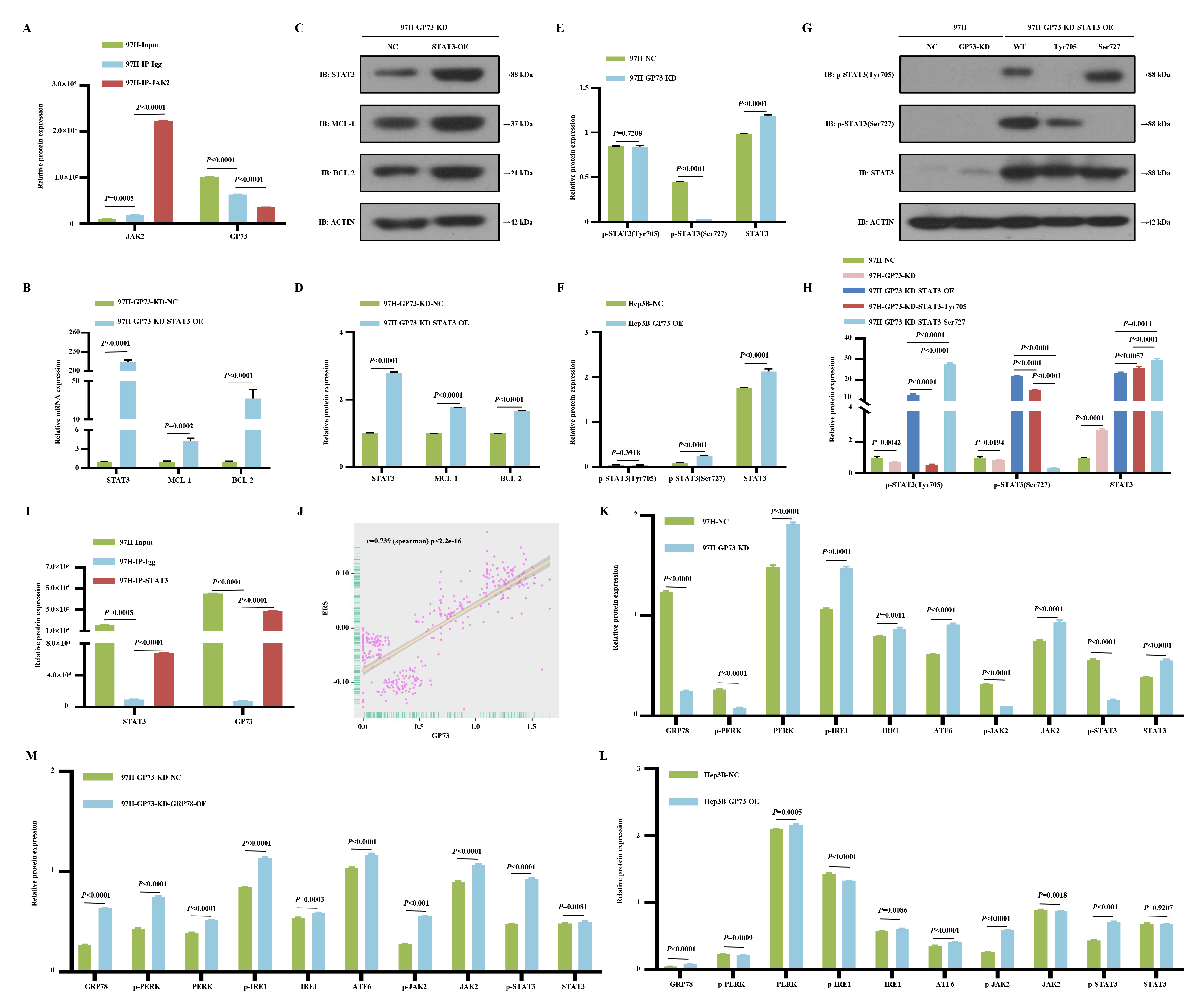


**Figure S5. Construction and validation of STAT3 over-expression and point mutations of pSTAT3-Tyr705 and pSTAT3-Ser727 in HCC cell lines**

**(A)** Co-IP assays detected the interaction between GP73 and JAK2 in MHCC97H cells. **(B)** Quantitative analysis of mRNA expressions of STAT3, MCL-1, BCL-2 in MHCC97H-GP73-KD and MHCC97H-GP73-KD-STAT3-OE cells. **(C)** Western blot detection and **(D)** quantitative analysis of protein expressions of STAT3, MCL-1, BCL-2 in MHCC97H-GP73-KD and MHCC97H-GP73-KD-STAT3-OE cells. **(E–F)** Quantitative analysis of protein expressions of STAT3, pSTAT3-Ser727, pSTAT3-Tyr705 in MHCC-97H-GP73-KD, MHCC-97H-GP73-NC, Hep3B-GP73-OE and Hep3B-GP73-NC cells. **(G)** Western blot detection of the expressions of STAT3, pSTAT3-Ser727, pSTAT3-Tyr705 in MHCC97H-NC, MHCC97H-GP73-KD, MHCC97H-GP73-KD-STAT3-OE, MHCC97H-GP73-KD-STAT3-Tyr705 and MHCC97H-GP73-KD-STAT3-Ser727 cells. **(H)** Quantitative analysis of protein expressions of STAT3, pSTAT3-Ser727, pSTAT3-Tyr705 in MHCC-97H-GP73-KD, MHCC-97H-GP73-NC, Hep3B-GP73-OE and Hep3B-GP73-NC cells. **(I)** Co-IP assays detected the interaction between GP73 and STAT3 in MHCC97H cells. **(J)** Correlation analysis of GP73 with ERS signal scores in HCC cells clusters. **(K–L)** Quantitative analysis of protein expressions of the key genes in JAk2-STAT3 pathway in MHCC97H-GP73-NC, MHCC97H-GP73-KD, Hep3B-GP73-NC and Hep3B-GP73-OE cells. **(M)** Quantitative analysis of protein expressions of the key genes in JAK2-STAT3 pathway in MHCC97H-GP73-KD-GRP78-OE and MHCC97H-GP73-KD cells.


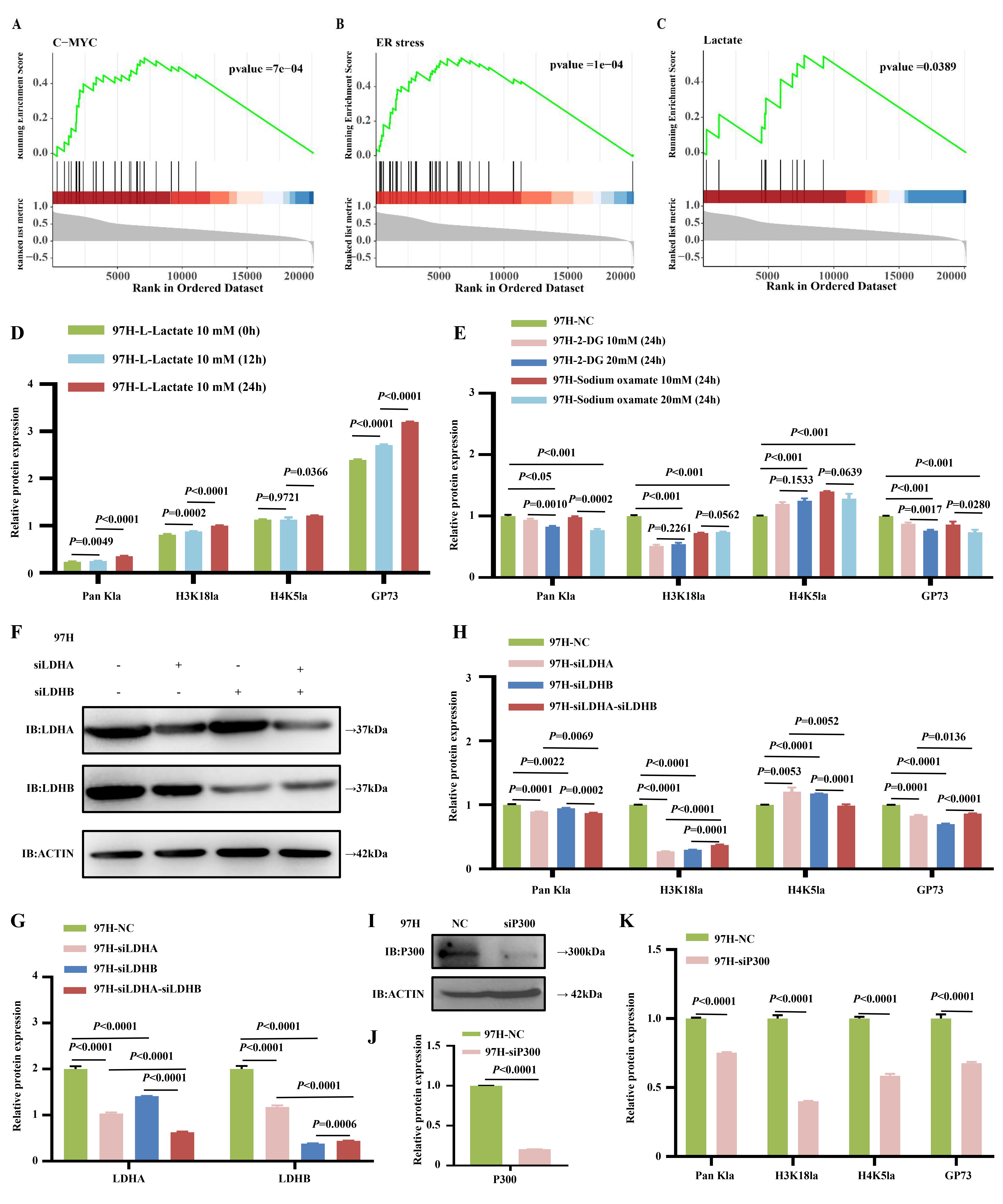


**Figure S6. GSEA analysis by using spatial single-cell transcriptomics to evaluate the correlation between GP73 gene and c-Myc, ERS and lactate signals in HCC cell subclusters**

**(A–C)** GSEA analysis by using single cell-spatial transcriptomics showing GP73 expression was significant positively correlated with **(A)** c-Myc, **(B)** ERS and **(C)** Lactate signals. **(D)** Quantitative analysis of protein expressions of Pan Kla, H3K18la, H4K5la and GP73 in MHCC97H cells being treated with lactate (10mM) for 0, 12 and 24 hours. **(E)** Quantitative analysis of protein expressions of Pan Kla, H3K18la, H4K5la and GP73 in MHCC97H cells being treated with 2-DG (10mM and 20mM) for 0, and 24 hours, and with sodium oxalate (10mM and 20mM) for 0, and 24 hours. **(F)** Western blot detection and (**G)** quantitative analysis of LDHA and LDHB expressions in MHCC97H-NC, MHCC97H-LDHA-KD, MHCC97H-LDHB-KD and MHCC97H-LDHA-KD-LDHB-KD cells. **(H)** Quantitative analysis of protein expressions of Pan Kla, H3K18la, H4K5la and GP73 in MHCC97H-LDHA-KD, MHCC97H-LDHB-KD and MHCC97H-LDHA-KD-LDHB-KD cells. **(I)** Western blot detection and quantitative analysis of P300 expression in MHCC97H-NC and MHCC97H-P300-KD cells. **(K)** Quantitative analysis of protein expressions of Pan Kla, H3K18la, H4K5la and GP73 in MHCC97H-P300-NC and MHCC97H-P300-KD cells.

**Abbreviations:** GSEA, gene set enrichment analysis; ERS, endoplasmic reticulum stress.


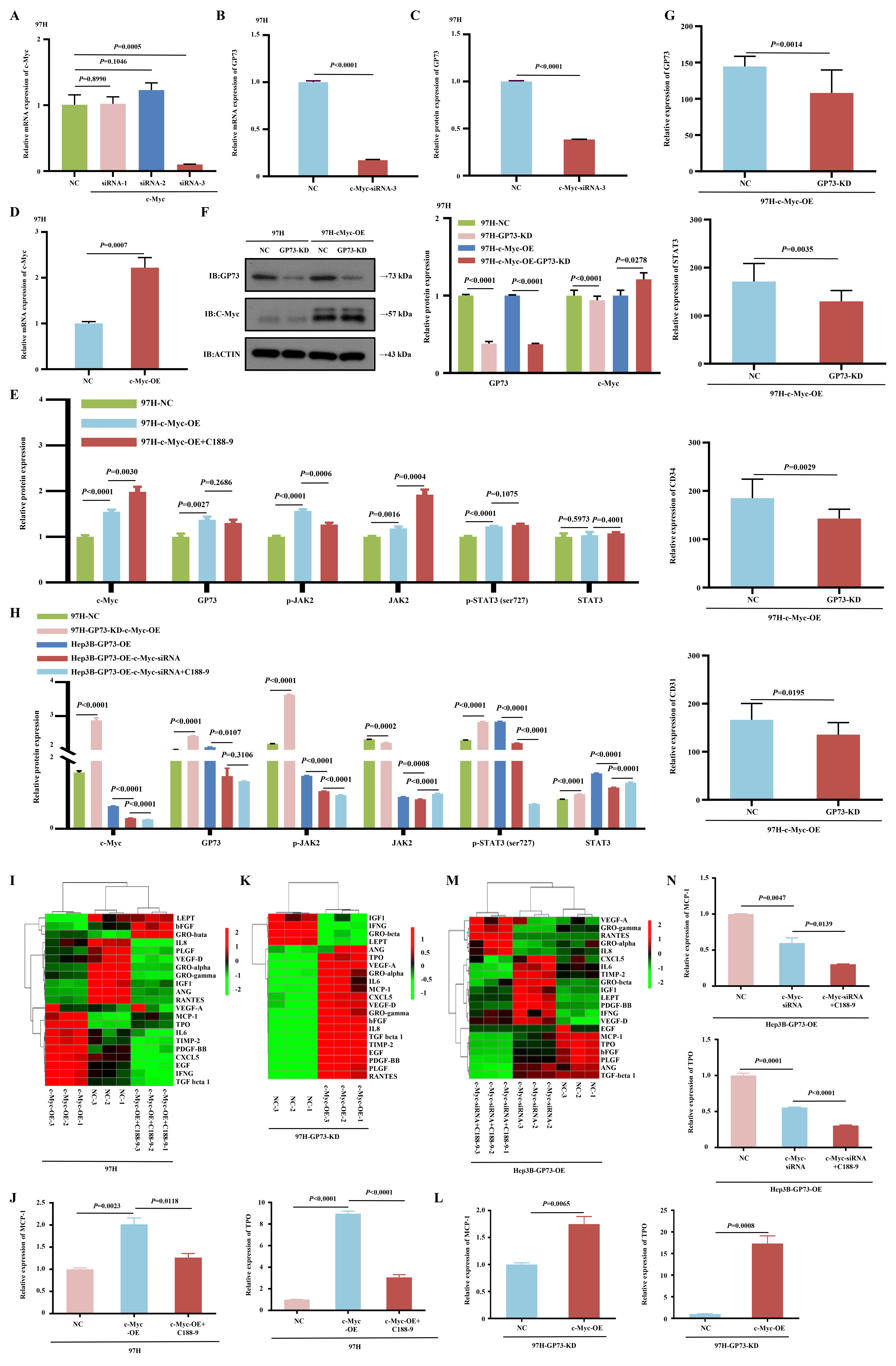


**Figure S7. Angiogenesis qPCR Array determined the differential expressions of pro-angiogenic factors in HCC cell lines**

**(A)** qRT-PCR determined the efficiency of knocked down c-Myc in MHCC-97H cells. siRNA-3 with the highest efficiency of knocked down c-Myc was selected to the follow-up experiment. **(B)** qRT-PCR and **(C)** western blot detected the efficiency of knocked down c-Myc in MHCC-97H cells. **(D)** qRT-PCR determined the efficiency of over expressed c-Myc in MHCC-97H cells. **(E)** Quantitative analysis of protein expressions of c-Myc, GP73 and key genes in JAK2/STAT3 pathway in MHCC97H-c-Myc-NC, MHCC97H-c-Myc-OE and MHCC97H-c-Myc-OE-C188-9 cells. C188-9 indicates the cells was treated with C188-9 (10 uM) for 24 hours. **(F)** Quantitative analysis of c-Myc and GP73 protein expressions in MHCC97H-NC, MHCC97H-GP73-KD, MHCC97H-c-Myc-OE and MHCC97H-c-Myc-OE-GP73-KD cells. **(G)** Quantitative analysis of immunofluorescence staining intensities of GP73, STAT3, CD34 and CD31 in MHCC97H-c-Myc-OE and MHCC97H-c-Myc-OE-GP73-KD cells. **(H)** Quantitative analysis of protein expressions of c-Myc, GP73 and key genes in JAK2/STAT3 signaling pathway in MHCC97H-GP73-KD, MHCC97H-GP73-KD-c-Myc-OE, Hep3B-GP73-OE, Hep3B-GP73-OE-cMyc-KD and Hep3B-GP73-OE-cMyc-KD-C188-9 cells. C188-9 indicates the cells was treated with C188-9 (10 uM) for 24 hours. **(I–N)** Angiogenesis qPCR Array detected the differential expression of pro-angiogenic factors in MHCC97H-NC，MHCC97H-c-Myc-OE, MHCC97H-c-Myc-OE-C188-9, MHCC97H-GP73-KD, MHCC97H-GP73-KD-c-Myc-OE, Hep3B-GP73-OE, Hep3B-GP73-OE-c-Myc-KD and Hep3B-GP73-OE-CA188-9 cells. C188-9 indicates the cells was treated with C188-9 (10uM) for 24 hours.
