# Supplementary material for: Integrating Single-Cell and Spatial Transcriptomics to Uncover and Elucidate GP73-Mediated Pro-Angiogenic Regulatory Networks in Hepatocellular Carcinoma
Source: Research (Wash D C). 2024 Jun 27;7:0387. doi: 10.34133/research.0387 (PMC11208919; doi:10.34133/research.0387)
Supplement: Supplementary 1 — Supplementary Methods Supplementary Results Figs. S1 to S7 Tables S1 to S9 Files S1 to S4 [file research.0387.f1.zip › Supplement Table.docx]

**Table S1. Baseline and clinicopathological Characteristics of 61 HCC patients underwent hepatectomy**

| **Variables** | **Entire cohort** |
| --- | --- |
| Age (years), Mean ±SD | 54.44 (51.94, 56.94) |
| Sex, n (%) |  |
| Female | 9 (14.8%) |
| Male | 52 (85.2%) |
| HBV infection |  |
| No | 19 (31%) |
| Yes | 42 (69%) |
| HCV infection |  |
| No | 56 (92%) |
| Yes | 5 (8%) |
| Serum AFP (ng/ml), n (%) |  |
| <400 | 40 (65.58%) |
| ≥400 | 21 (34.42%) |
| Child-Pugh scores, n (%) |  |
| 5-6 | 59 (96.72%) |
| 7 | 2 (3.28%) |
| Tumor size (cm), n (%) |  |
| ≤5 | 28 (45.909%) |
| >5 | 33 (54.10%) |
| Tumor number, n (%) |  |
| Single | 40 (65.57%) |
| Mutiple | 21 (34.43%) |
| Macrovascular invasion, n (%) |  |
| No | 51 (83.61%) |
| Yes | 10 (16.39%) |
| Extrahepatic metastasis, n (%) |  |
| No | 61 (100%) |
| Yes | 0 (0%) |
| BCLC stages, n (%) |  |
| 0/A | 35 (57.38%) |
| B | 16 (26.23%) |
| C | 10 (16.39%) |
| Edmondson-Steiner grades, n (%) |  |
| I/II | 27 (44.26%) |
| III//IV | 34 (55.74%) |
| CD34 IHC staining strength, n (%) |  |
| Negative (-) | 0 (0%) |
| Weakly positive (+) | 1 (1.64%) |
| Positive (++) | 2 (3.28%) |
| Strongly positive (+++) | 58 (95.08%) |
| Percentage of CD34 positive cells, n (%) |  |
| ≤25 | 33 (54.10%) |
| 26-50 | 20 (32.79%) |
| 51-75 | 8 (13.11%) |
| >75 | 0 (0%) |
| GP73 IHC staining strength, n (%) |  |
| Negative (-) | 1 (1.64%) |
| Weakly positive (+) | 2 (3.28%) |
| Positive (++) | 8 (13.11%) |
| Strongly positive (+++) | 50 (81.97%) |
| Percentage of GP73 positive cells, n (%) |  |
| ≤25 | 32 (52.46%) |
| 26-50 | 23 (37.70%) |
| 51-75 | 6 (9.84%) |
| >75 | 0 (0%) |
| CD34 IHC scores, Mean ±SD | 4.70 (2.49, 6.91) |
| GP73 IHC scores, Mean ±SD | 4.46 (2.19, 6.73) |

**Note:** CD34 IHC score=The intensity score×The percentage of positive cells score; GP73 IHC score= The intensity score×The percentage of positive cells score;

**Abbreviations:** HBV, hepatitis B virus; HCV, hepatitis C virus; BCLC, Barcelona Clinic Liver Cancer; AFP, alpha-fetoprotein; IHC, immunohistochemistry.

**Table S2. Clinicopathological characteristics of 6 HCC samples and 1 normal liver tissue subjected to single cell-spatial RNA sequencing**

| **Patients/**  **Samples** | **Gender** | **Age (Years)** | **BMI (kg/m^2^)** | **Hepatitis** | **Serum AFP**  **(ng/ml)** | **BCLC stages** | **PVTT** | **Tumor Size （cm）** | **ES**  **grades** | **MVD**  **(HFP)** | **Single**  **Cells** | **Spatial**  **Spots** |
| --- | --- | --- | --- | --- | --- | --- | --- | --- | --- | --- | --- | --- |
| HCC1 | Male | 63 | 18.2 | HBV | 2.35 | C | Right posterior superior branch | 4.8*3.3 | II | >50 | 9854 | 3320 |
| HCC2 | Male | 50 | 24.4 | HBV | 35469 | C | Right posterior inferior branch | 11.8*7.5 | Ⅲ-Ⅳ | ＜50 | 10452 | 2811 |
| HCC3 | Male | 67 | 19.8 | None | 3.31 | A | / | 6.2*5.5 | II | ＞200 | 10840 | / |
| HCC4 | Male | 64 | 22.2 | HBV | 9.22 | B | / | 3.5*2.7 | II | ＞50 | 11217 | 2461 |
| HCC5 | Male | 56 | 25.7 | HBV | 131.6 | A | / | 15.5*12.4 | II-III | ＞50 | 8534 | 4046 |
| HCC6 | Female | 48 | 21.42 | HBV | 3.29 | 0 | / | 3.1*1.8 | II | ＜50 | 9370 | 2408 |
| Control | Female | 42 | 20.1 | HBV | 1.66 | / | / | / | / | / | 11899 | / |
| GSE115469 | / | / | / | / | / | / | / | / | / | / | 8416 | / |
| GSE149614 | / | / | / | / | / | / | / | / | / | / | 29391 | / |
| GSE151530 | / | / | / | / | / | / | / | / | / | / | 29744 | / |
| GSE156625 | / | / | / | / | / | / | / | / | / | / | 49623 | / |

**Abbreviations:** HCC, hepatocullar carcinoma; BMI, Body Mass Index; HBV, hepatitis B virals; AFP, alpha-fetoprotein; BCLC, Barcelona Clinical Liver Cancer; PVTT, portal vein tumor thrumbus; ES grade, Edmondson-Steiner grade; MVD, microvascular density.

**Table S3. Univariate and multivariate logistic regression analyses of prognostic factors of HCC response to anti-angiogenic agent in 238 advanced HCC patients treated with lenvatinib.**

| **Variable** | **Univariate** | | | | | |  | **Multivariate** | | | | | |
| --- | --- | --- | --- | --- | --- | --- | --- | --- | --- | --- | --- | --- | --- |
|  | **β** | **SE** | **Wald** | ***P*** | **OR** | **OR (95%CI)** |  | **β** | **SE** | **Wald** | ***P*** | **OR** | **OR (95%CI)** |
| Age (years), Mean ±SD | -0.011 | 0.015 | 0.534 | 0.465 | 0.989 | 0.959-1.019 |  |  |  |  |  |  |  |
| Sex, male | -0.260 | 0.523 | 0.246 | 0.620 | 0.771 | 0.277-2.151 |  |  |  |  |  |  |  |
| HBV infection, yes | 0.508 | 0.459 | 1.223 | 0.269 | 1.662 | 0.675-4.090 |  |  |  |  |  |  |  |
| HCV infection, yes | 0.188 | 1.129 | 0.028 | 0.868 | 1.207 | 0.132-11.026 |  |  |  |  |  |  |  |
| Serum AFP level, ≥ 400 ng/ml | -0.128 | 0.308 | 0.173 | 0.678 | 0.880 | 0.481-1.609 |  |  |  |  |  |  |  |
| Serum GP73 level, ng/ml | 0.021 | 0.005 | 21.317 | 0.000 | 1.021 | 1.012-1.030 |  | 0.02 | 0.005 | 18.582 | 0.000 | 1.020 | 1.011-1.030 |
| Child-Pugh scores 7 | 0.025 | 0.310 | 0.007 | 0.935 | 1.026 | 0.559-1.883 |  |  |  |  |  |  |  |
| Tumor size > 5cm | -0.081 | 0.319 | 0.064 | 0.800 | 0.922 | 0.494-1.723 |  |  |  |  |  |  |  |
| Multiple tumors | -0.296 | 0.315 | 0.881 | 0.348 | 0.744 | 0.402-1.379 |  |  |  |  |  |  |  |
| Macrovascular invasion, yes | -0.128 | 0.309 | 0.171 | 0.679 | 0.880 | 0.481-1.611 |  |  |  |  |  |  |  |
| Extrahepatic metastasis, yes | -0.591 | 0.329 | 3.216 | 0.073 | 0.554 | 0.29-1.056 |  |  |  |  |  |  |  |
| BCLC-C stage | -0.354 | 0.356 | 0.990 | 0.320 | 0.702 | 0.35-1.409 |  |  |  |  |  |  |  |
| Combined ICIs therapy, yes | 0.668 | 0.327 | 4.178 | 0.041 | 1.951 | 1.028-3.704 |  | 0.275 | 0.359 | 0.586 | 0.444 | 1.317 | 0.651-2.662 |

**Abbreviations:** HBV, hepatitis B virus; HCV, hepatitis C virus; AFP, alpha-fetoprotein; GP73, golgi protein 73; BCLC, Barcelona Clinical Liver Cancer; ICIs, immune checkpoint inhibitors.

**Table S4**. **Univariate and multivariate Cox analyses of prognostic factors of overall survival in 119 advanced HCC patients treated with anti-angiogenesis agent in the training cohort**

|  | **Univariate Cox regression** | | |  | **Multivariate Cox regression** | | |
| --- | --- | --- | --- | --- | --- | --- | --- |
| **Variables** | **HR** | **95% CI** | ***P*-value** |  | **HR** | **95% CI** | ***P*-value** |
| Age (years), Mean ±SD | 1.01 | 0.96, 1.05 | 0.8 |  |  |  |  |
| Sex, male | 0.62 | 0.17, 2.23 | 0.5 |  |  |  |  |
| HBV infection, yes | 0.47 | 0.17, 1.32 | 0.2 |  |  |  |  |
| HCV infection, yes | 1.52 | 0.15, 15.9 | 0.7 |  |  |  |  |
| Serum AFP level, ≥ 400 ng/ml | 1.65 | 0.77, 3.52 | 0.2 |  |  |  |  |
| Serum GP73 level, ≥178ng/ml | 0.22 | 0.10, 0.52 | <0.001 |  | 0.22 | 0.11, 0.44 | <0.001 |
| Child-Pugh scores 7 | 0.63 | 0.31, 1.30 | 0.2 |  |  |  |  |
| Tumor size > 5cm | 1.02 | 0.45, 2.32 | >0.9 |  |  |  |  |
| Multiple tumors | 0.76 | 0.36, 1.62 | 0.5 |  |  |  |  |
| Macrovascular invasion, yes | 0.88 | 0.39, 2.02 | 0.8 |  |  |  |  |
| Extrahepatic metastasis, yes | 0.55 | 0.20, 1.51 | 0.2 |  |  |  |  |
| BCLC-C stage | 8.54 | 1.63, 44.8 | 0.011 |  | 6.44 | 1.52, 27.3 | 0.012 |
| Combined ICIs therapy, yes | 0.28 | 0.14, 0.57 | <0.001 |  | 0.30 | 0.16, 0.57 | <0.001 |

Abbreviations: HBV, hepatitis B virus; HCV, hepatitis C virus; AFP, alpha-fetoprotein; GP73, golgi protein 73; BCLC, Barcelona Clinical Liver Cancer; ICIs, immune checkpoint inhibitors.

**Table S5. PCR Primers sequence used in this study**

| **Gene** | **Sequences (5’-3’)** | |
| --- | --- | --- |
| GP73 | Forward | -ATCACCACAGGTGAGAGGCTCA- |
|  | Reverse | -ACTTCCTCTCCAGGTTGGTCTG- |
| LIF | Forward | -ATCAGGAGCCAACTGGCACA- |
|  | Reverse | -CCACATAGCTTGTCCAGGTTG- |
| IL15RA | Forward | -GCTATCTCCACGTCCACTGT- |
|  | Reverse | -GGCTTCCATTTCAACGCTGG- |
| OSMR | Forward | -GGTGTTCCTACCAAATCTGCG- |
|  | Reverse | -CCACCCTCTGTGCCTGCAA- |
| JAK2 | Forward | -CAGATGGAAACTGTTCGCTCAG- |
|  | Reverse | -GAGGTTGGTACATCAGAAACACC- |
| JAK3 | Forward | -TGACCCTCACTTCCTGCTGT- |
|  | Reverse | -GGCTGAACCAAGGATGATGTGG- |
| STAT3 | Forward | -CTTTGAGACCGAGGTGTATCACC- |
|  | Reverse | -GGTCAGCATGTTGTACCACAGG- |
| MCL-1 | Forward | -GCTCATAGGCATCTCCATCGC- |
|  | Reverse | -TGCAGCACTACCTCCTTGGAAG- |
| BCL-2 | Forward | -ATGTGTGTGGAGAGCGTCAA- |
|  | Reverse | -ACAGTTCCACAAAGGCATCC- |
| ACTIN | Forward | -TCTGGCACCACACCTTCTAC- |
|  | Reverse | -GATAGCACAGCCTGGATAGCA- |
| c-MYC | Forward | -CCTGGTGCTCCATGAGGAGAC- |
|  | Reverse | -CAGACTCTGACCTTTTGCCAGG- |

**Table S6. Western Blot antibodies used in this study**

| **Antibodies** | **Company** | **Host species** | **Antibody Numbering** | **Dilution Ratio** |
| --- | --- | --- | --- | --- |
| GP73 | Abways, China | Rabbit | CY5578 | 1/1000 |
| LIF | Proteintech, USA | Rabbit | 26757-1-AP | 1/1000 |
| IL15RA | proteintech, USA | Rabbit | 16744-1-AP | 1/1000 |
| OSMR | proteintech, USA | Rabbit | 10982-1-AP | 1/1000 |
| p-JAK2 | cst, USA | Rabbit | 8082 | 1/1000 |
| JAK2 | cst, USA | Rabbit | 3230 | 1/1000 |
| p-JAK3 | cst, USA | Rabbit | 5031 | 1/1000 |
| JAK3 | cst, USA | Rabbit | 8827 | 1/1000 |
| p-STAT3（Tyr705） | cst, USA | Rabbit | 52075 | 1/1000 |
| p-STAT3（Ser727） | cst, USA | Rabbit | 9134 | 1/1000 |
| STAT3 | cst, USA | Rabbit | 12640 | 1/1000 |
| MCL-1 | cst, USA | Rabbit | 94296 | 1/1000 |
| BCL-2 | cst, USA | Rabbit | 4223 | 1/1000 |
| c-MYC | Abcam, UK | Rabbit | Ab32072 | 1/1000 |
| GRP78 | Abways, China | Rabbit | Cy5166 | 1/1000 |
| p-PERK | bioss, China | Rabbit | bs-3330R | 1/1000 |
| PERK | abways, China | Rabbit | CY2759 | 1/1000 |
| pIRE1 | abways, China | Rabbit | Cy5605 | 1/1000 |
| IRE1 | bioss, China | Rabbit | bs-8680R | 1/1000 |
| ATF6α | bioss, China | Rabbit | bs-23093R | 1/1000 |
| Pan Kla | PTM Bio, China | Rabbit | PTM-1401 | 1/1000 |
| H3K18la | PTM Bio, China | Rabbit | PTM1406-RM | 1/750 |
| H4K5la | PTM Bio, China | Rabbit | PTM-1407 | 1/1000 |
| LDHA | proteintech, China | Rabbit | 19987-1-AP | 1/5000 |
| LDHB | proteintech, China | Rabbit | 19988-1-AP | 1/3000 |
| P300 | Santa Cruz, USA | Rabbit | Sc-48343 | 1/200 |
| Histone-H3 | proteintech, China | Rabbit | 17168-1-AP | 1/2000 |
| ACTIN | abways, China | Rabbit | AB2001 | 1/10000 |
| HRP | Cst, USA | Rabbit | 7076 | 1/1000 |

**Table S7. shRNA, siRNA and full-length sequence used in this study**

| **Gene** | **SiRNA primers Sequences (5’-3’)** |
| --- | --- |
| GP73 | shRNA 1：GCAGAAACTGAGAGGAGAA |
|  | shRNA 2：AGGGAAACGTGCTTGGTAA |
|  | shRNA 3：GAATAGAAGAGGTCACCAA |
| STAT3 | shRNA 1：GCAACAGATTGCCTGCATTGG |
|  | shRNA 2：GCGTCCAGTTCACTACTAAAG |
|  | shRNA 3：GCAGCCTCTCTGCAGAATTCA |
| JAK2 | shRNA 1：GCCCAGATGAGATCTATAT |
|  | shRNA 2：CTGGCAACGAGAAATATAT |
|  | shRNA 3：GCAGTCCTAAGGACTTTAA |
| c-MYC | shRNA 1：GAGGAGACATGGTGAACCA |
|  | shRNA 2：GGGTCAAGTTGGACAGTGT |
|  | shRNA 3：CGACGAGACCTTCATCAAA |
| LDHA | siRNA 1：CGAACUGGGCAGUAUAAAC |
|  | siRNA 2：GCCUGUGCCAUCAGUAUCUUA |
|  | siRNA 3：GCAAACUCCAAGCUGGUCAUU |
| LDHB | siRNA 4：CCACCAUGAUUAAGGGUCUUU |
|  | siRNA 1：GGAUAUACCAACUGGGCUAUU |
| P300 | siRNA 1：GCAGCUCAACCAUCCACUA |
|  | siRNA 2：GCACAAAUGUCUAGUUCUU |
|  | siRNA 3：GCAAAGGAAUUGCCUUAUU |

**GP73 OE sequence：**

Atgatgggcttgggaaacgggcgtcgcagcatgaagtcgccgcccctcgtgctggccgccctggtggcctgcatcatcgtcttgggcttcaactactggattgcgagctcccggagcgtggacctccagacacggatcatggagctggaaggcagggtccgcagggcggctgcagagagaggcgccgtggagctgaagaagaacgagttccagggagagctggagaagcagcgggagcagcttgacaaaatccagtccagccacaacttccagctggagagcgtcaacaagctgtaccaggacgaaaaggcggttttggtgaataacatcaccacaggtgagaggctcatccgagtgctgcaagaccagttaaagaccctgcagaggaattacggcaggctgcagcaggatgtcctccagtttcagaagaaccagaccaacctggagaggaagttctcctacgacctgagccagtgcatcaatcagatgaaggaggtgaaggaacagtgtgaggagcgaatagaagaggtcaccaaaaaggggaatgaagctgtagcttccagagacctgagtgaaaacaacgaccagagacagcagctccaagccctcagtgagcctcagcccaggctgcaggcagcaggcctgccacacacagaggtgccacaagggaagggaaacgtgcttggtaacagcaagtcccagacaccagcccccagttccgaagtggttttggattcaaagagacaagttgagaaagaggaaaccaatgagatccaggtggtgaatgaggagcctcagagggacaggctgccgcaggagccaggccgggagcaggtggtggaagacagacctgtaggtggaagaggcttcgggggagccggagaactgggccagaccccacaggtgcaggctgccctgtcagtgagccaggaaaatccagagatggagggccctgagcgagaccagcttgtcatccccgacggacaggaggaggagcaggaagctgccggggaagggagaaaccagcagaaactgagaggagaagatgactacaacatggatgaaaatgaagcagaatctgagacagacaagcaagcagccctggcagggaatgacagaaacatagatgtttttaatgttgaagatcagaaaagagacaccataaatttacttgatcagcgtgaaaagcggaatcatacactctga

**c-Myc OE sequence：**

atgcccctcaacgttagcttcaccaacaggaactatgacctcgactacgactcggtgcagccgtatttctactgcgacgaggaggagaacttctaccagcagcagcagcagagcgagctgcagcccccggcgcccagcgaggatatctggaagaaattcgagctgctgcccaccccgcccctgtcccctagccgccgctccgggctctgctcgccctcctacgttgcggtcacacccttctcccttcggggagacaacgacggcggtggcgggagcttctccacggccgaccagctggagatggtgaccgagctgctgggaggagacatggtgaaccagagtttcatctgcgacccggacgacgagaccttcatcaaaaacatcatcatccaggactgtatgtggagcggcttctcggccgccgccaagctcgtctcagagaagctggcctcctaccaggctgcgcgcaaagacagcggcagcccgaaccccgcccgcggccacagcgtctgctccacctccagcttgtacctgcaggatctgagcgccgccgcctcagagtgcatcgacccctcggtggtcttcccctaccctctcaacgacagcagctcgcccaagtcctgcgcctcgcaagactccagcgccttctctccgtcctcggattctctgctctcctcgacggagtcctccccgcagggcagccccgagcccctggtgctccatgaggagacaccgcccaccaccagcagcgactctgaggaggaacaagaagatgaggaagaaatcgatgttgtttctgtggaaaagaggcaggctcctggcaaaaggtcagagtctggatcaccttctgctggaggccacagcaaacctcctcacagcccactggtcctcaagaggtgccacgtctccacacatcagcacaactacgcagcgcctccctccactcggaaggactatcctgctgccaagagggtcaagttggacagtgtcagagtcctgagacagatcagcaacaaccgaaaatgcaccagccccaggtcctcggacaccgaggagaatgtcaagaggcgaacacacaacgtcttggagcgccagaggaggaacgagctaaaacggagcttttttgccctgcgtgaccagatcccggagttggaaaacaatgaaaaggcccccaaggtagttatccttaaaaaagccacagcatacatcctgtccgtccaagcagaggagcaaaagctcatttctgaagaggacttgttgcggaaacgacgagaacagttgaaacacaaacttgaacagctacggaactcttgtgcgtaa

**GRP78 OE sequence：**

atgaagctctccctggtggccgcgatgctgctgctgctcagcgcggcgcgggccgaggaggaggacaagaaggaggacgtgggcacggtggtcggcatcgacctggggaccacctactcctgcgtcggcgtgttcaagaacggccgcgtggagatcatcgccaacgatcagggcaaccgcatcacgccgtcctatgtcgccttcactcctgaaggggaacgtctgattggcgatgccgccaagaaccagctcacctccaaccccgagaacacggtctttgacgccaagcggctcatcggccgcacgtggaatgacccgtctgtgcagcaggacatcaagttcttgccgttcaaggtggttgaaaagaaaactaaaccatacattcaagttgatattggaggtgggcaaacaaagacatttgctcctgaagaaatttctgccatggttctcactaaaatgaaagaaaccgctgaggcttatttgggaaagaaggttacccatgcagttgttactgtaccagcctattttaatgatgcccaacgccaagcaaccaaagacgctggaactattgctggcctaaatgttatgaggatcatcaacgagcctacggcagctgctattgcttatggcctggataagagggagggggagaagaacatcctggtgtttgacctgggtggcggaaccttcgatgtgtctcttctcaccattgacaatggtgtcttcgaagttgtggccactaatggagatactcatctgggtggagaagactttgaccagcgtgtcatggaacacttcatcaaactgtacaaaaagaagacgggcaaagatgtcaggaaagacaatagagctgtgcagaaactccggcgcgaggtagaaaaggccaaacgggccctgtcttctcagcatcaagcaagaattgaaattgagtccttctatgaaggagaagacttttctgagaccctgactcgggccaaatttgaagagctcaacatggatctgttccggtctactatgaagcccgtccagaaagtgttggaagattctgatttgaagaagtctgatattgatgaaattgttcttgttggtggctcgactcgaattccaaagattcagcaactggttaaagagttcttcaatggcaaggaaccatcccgtggcataaacccagatgaagctgtagcgtatggtgctgctgtccaggctggtgtgctctctggtgatcaagatacaggtgacctggtactgcttgatgtatgtccccttacacttggtattgaaactgtgggaggtgtcatgaccaaactgattccaaggaacacagtggtgcctaccaagaagtctcagatcttttctacagcttctgataatcaaccaactgttacaatcaaggtctatgaaggtgaaagacccctgacaaaagacaatcatcttctgggtacatttgatctgactggaattcctcctgctcctcgtggggtcccacagattgaagtcacctttgagatagatgtgaatggtattcttcgagtgacagctgaagacaagggtacagggaacaaaaataagatcacaatcaccaatgaccagaatcgcctgacacctgaagaaatcgaaaggatggttaatgatgctgagaagtttgctgaggaagacaaaaagctcaaggagcgcattgatactagaaatgagttggaaagctatgcctattctctaaagaatcagattggagataaagaaaagctgggaggtaaactttcctctgaagataaggagaccatggaaaaagctgtagaagaaaagattgaatggctggaaagccaccaagatgctgacattgaagacttcaaagctaagaagaaggaactggaagaaattgttcaaccaattatcagcaaactctatggaagtgcaggccctcccccaactggtgaagaggatacagcagaaaaagatgagttgtag

**STAT3 OE sequence：**

atggcccaatggaatcagctacagcagcttgacacacggtacctggagcagctccatcagctctacagtgacagcttcccaatggagctgcggcagtttctggccccttggattgagagtcaagattgggcatatgcggccagcaaagaatcacatgccactttggtgtttcataatctcctgggagagattgaccagcagtatagccgcttcctgcaagagtcgaatgttctctatcagcacaatctacgaagaatcaagcagtttcttcagagcaggtatcttgagaagccaatggagattgcccggattgtggcccggtgcctgtgggaagaatcacgccttctacagactgcagccactgcggcccagcaagggggccaggccaaccaccccacagcagccgtggtgacggagaagcagcagatgctggagcagcaccttcaggatgtccggaagagagtgcaggatctagaacagaaaatgaaagtggtagagaatctccaggatgactttgatttcaactataaaaccctcaagagtcaaggagacatgcaagatctgaatggaaacaaccagtcagtgaccaggcagaagatgcagcagctggaacagatgctcactgcgctggaccagatgcggagaagcatcgtgagtgagctggcggggcttttgtcagcgatggagtacgtgcagaaaactctcacggacgaggagctggctgactggaagaggcggcaacagattgcctgcattggaggcccgcccaacatctgcctagatcggctagaaaactggataacgtcattagcagaatctcaacttcagacccgtcaacaaattaagaaactggaggagttgcagcaaaaagtttcctacaaaggggaccccattgtacagcaccggccgatgctggaggagagaatcgtggagctgtttagaaacttaatgaaaagtgcctttgtggtggagcggcagccctgcatgcccatgcatcctgaccggcccctcgtcatcaagaccggcgtccagttcactactaaagtcaggttgctggtcaaattccctgagttgaattatcagcttaaaattaaagtgtgcattgacaaagactctggggacgttgcagctctcagaggatcccggaaatttaacattctgggcacaaacacaaaagtgatgaacatggaagaatccaacaacggcagcctctctgcagaattcaaacacttgaccctgagggagcagagatgtgggaatgggggccgagccaattgtgatgcttccctgattgtgactgaggagctgcacctgatcacctttgagaccgaggtgtatcaccaaggcctcaagattgacctagagacccactccttgccagttgtggtgatctccaacatctgtcagatgccaaatgcctgggcgtccatcctgtggtacaacatgctgaccaacaatcccaagaatgtaaacttttttaccaagcccccaattggaacctgggatcaagtggccgaggtcctgagctggcagttctcctccaccaccaagcgaggactgagcatcgagcagctgactacactggcagagaaactcttgggacctggtgtgaattattcagggtgtcagatcacatgggctaaattttgcaaagaaaacatggctggcaagggcttctccttctgggtctggctggacaatatcattgaccttgtgaaaaagtacatcctggccctttggaacgaagggtacatcatgggctttatcagtaaggagcgggagcgggccatcttgagcactaagcctccaggcaccttcctgctaagattcagtgaaagcagcaaagaaggaggcgtcactttcacttgggtggagaaggacatcagcggtaagacccagatccagtccgtggaaccatacacaaagcagcagctgaacaacatgtcatttgctgaaatcatcatgggctataagatcatggatgctaccaatatcctggtgtctccactggtctatctctatcctgacattcccaaggaggaggcattcggaaagtattgtcggccagagagccaggagcatcctgaagctgacccaggtagcgctgccccatacctgaagaccaagtttatctgtgtgacaccaacgacctgcagcaataccattgacctgccgatgtccccccgcactttagattcattgatgcagtttggaaataatggtgaaggtgctgaaccctcagcaggagggcagtttgagtccctcacctttgacatggagttgacctcggagtgcgctacctcccccatgtga

**STAT3(Ser727) OE sequence：**

atggcccaatggaatcagctacagcagcttgacacacggtacctggagcagctccatcagctctacagtgacagcttcccaatggagctgcggcagtttctggccccttggattgagagtcaagattgggcatatgcggccagcaaagaatcacatgccactttggtgtttcataatctcctgggagagattgaccagcagtatagccgcttcctgcaagagtcgaatgttctctatcagcacaatctacgaagaatcaagcagtttcttcagagcaggtatcttgagaagccaatggagattgcccggattgtggcccggtgcctgtgggaagaatcacgccttctacagactgcagccactgcggcccagcaagggggccaggccaaccaccccacagcagccgtggtgacggagaagcagcagatgctggagcagcaccttcaggatgtccggaagagagtgcaggatctagaacagaaaatgaaagtggtagagaatctccaggatgactttgatttcaactataaaaccctcaagagtcaaggagacatgcaagatctgaatggaaacaaccagtcagtgaccaggcagaagatgcagcagctggaacagatgctcactgcgctggaccagatgcggagaagcatcgtgagtgagctggcggggcttttgtcagcgatggagtacgtgcagaaaactctcacggacgaggagctggctgactggaagaggcggcaacagattgcctgcattggaggcccgcccaacatctgcctagatcggctagaaaactggataacgtcattagcagaatctcaacttcagacccgtcaacaaattaagaaactggaggagttgcagcaaaaagtttcctacaaaggggaccccattgtacagcaccggccgatgctggaggagagaatcgtggagctgtttagaaacttaatgaaaagtgcctttgtggtggagcggcagccctgcatgcccatgcatcctgaccggcccctcgtcatcaagaccggcgtccagttcactactaaagtcaggttgctggtcaaattccctgagttgaattatcagcttaaaattaaagtgtgcattgacaaagactctggggacgttgcagctctcagaggatcccggaaatttaacattctgggcacaaacacaaaagtgatgaacatggaagaatccaacaacggcagcctctctgcagaattcaaacacttgaccctgagggagcagagatgtgggaatgggggccgagccaattgtgatgcttccctgattgtgactgaggagctgcacctgatcacctttgagaccgaggtgtatcaccaaggcctcaagattgacctagagacccactccttgccagttgtggtgatctccaacatctgtcagatgccaaatgcctgggcgtccatcctgtggtacaacatgctgaccaacaatcccaagaatgtaaacttttttaccaagcccccaattggaacctgggatcaagtggccgaggtcctgagctggcagttctcctccaccaccaagcgaggactgagcatcgagcagctgactacactggcagagaaactcttgggacctggtgtgaattattcagggtgtcagatcacatgggctaaattttgcaaagaaaacatggctggcaagggcttctccttctgggtctggctggacaatatcattgaccttgtgaaaaagtacatcctggccctttggaacgaagggtacatcatgggctttatcagtaaggagcgggagcgggccatcttgagcactaagcctccaggcaccttcctgctaagattcagtgaaagcagcaaagaaggaggcgtcactttcacttgggtggagaaggacatcagcggtaagacccagatccagtccgtggaaccatacacaaagcagcagctgaacaacatgtcatttgctgaaatcatcatgggctataagatcatggatgctaccaatatcctggtgtctccactggtctatctctatcctgacattcccaaggaggaggcattcggaaagtattgtcggccagagagccaggagcatcctgaagctgacccaggtagcgctgccccatacctgaagaccaagtttatctgtgtgacaccaacgacctgcagcaataccattgacctgccgatgGccccccgcactttagattcattgatgcagtttggaaataatggtgaaggtgctgaaccctcagcaggagggcagtttgagtccctcacctttgacatggagttgacctcggagtgcgctacctcccccatgtga

**STAT3(Tyr705) OE sequence：**

atggcccaatggaatcagctacagcagcttgacacacggtacctggagcagctccatcagctctacagtgacagcttcccaatggagctgcggcagtttctggccccttggattgagagtcaagattgggcatatgcggccagcaaagaatcacatgccactttggtgtttcataatctcctgggagagattgaccagcagtatagccgcttcctgcaagagtcgaatgttctctatcagcacaatctacgaagaatcaagcagtttcttcagagcaggtatcttgagaagccaatggagattgcccggattgtggcccggtgcctgtgggaagaatcacgccttctacagactgcagccactgcggcccagcaagggggccaggccaaccaccccacagcagccgtggtgacggagaagcagcagatgctggagcagcaccttcaggatgtccggaagagagtgcaggatctagaacagaaaatgaaagtggtagagaatctccaggatgactttgatttcaactataaaaccctcaagagtcaaggagacatgcaagatctgaatggaaacaaccagtcagtgaccaggcagaagatgcagcagctggaacagatgctcactgcgctggaccagatgcggagaagcatcgtgagtgagctggcggggcttttgtcagcgatggagtacgtgcagaaaactctcacggacgaggagctggctgactggaagaggcggcaacagattgcctgcattggaggcccgcccaacatctgcctagatcggctagaaaactggataacgtcattagcagaatctcaacttcagacccgtcaacaaattaagaaactggaggagttgcagcaaaaagtttcctacaaaggggaccccattgtacagcaccggccgatgctggaggagagaatcgtggagctgtttagaaacttaatgaaaagtgcctttgtggtggagcggcagccctgcatgcccatgcatcctgaccggcccctcgtcatcaagaccggcgtccagttcactactaaagtcaggttgctggtcaaattccctgagttgaattatcagcttaaaattaaagtgtgcattgacaaagactctggggacgttgcagctctcagaggatcccggaaatttaacattctgggcacaaacacaaaagtgatgaacatggaagaatccaacaacggcagcctctctgcagaattcaaacacttgaccctgagggagcagagatgtgggaatgggggccgagccaattgtgatgcttccctgattgtgactgaggagctgcacctgatcacctttgagaccgaggtgtatcaccaaggcctcaagattgacctagagacccactccttgccagttgtggtgatctccaacatctgtcagatgccaaatgcctgggcgtccatcctgtggtacaacatgctgaccaacaatcccaagaatgtaaacttttttaccaagcccccaattggaacctgggatcaagtggccgaggtcctgagctggcagttctcctccaccaccaagcgaggactgagcatcgagcagctgactacactggcagagaaactcttgggacctggtgtgaattattcagggtgtcagatcacatgggctaaattttgcaaagaaaacatggctggcaagggcttctccttctgggtctggctggacaatatcattgaccttgtgaaaaagtacatcctggccctttggaacgaagggtacatcatgggctttatcagtaaggagcgggagcgggccatcttgagcactaagcctccaggcaccttcctgctaagattcagtgaaagcagcaaagaaggaggcgtcactttcacttgggtggagaaggacatcagcggtaagacccagatccagtccgtggaaccatacacaaagcagcagctgaacaacatgtcatttgctgaaatcatcatgggctataagatcatggatgctaccaatatcctggtgtctccactggtctatctctatcctgacattcccaaggaggaggcattcggaaagtattgtcggccagagagccaggagcatcctgaagctgacccaggtagcgctgccccaGCcctgaagaccaagtttatctgtgtgacaccaacgacctgcagcaataccattgacctgccgatgtccccccgcactttagattcattgatgcagtttggaaataatggtgaaggtgctgaaccctcagcaggagggcagtttgagtccctcacctttgacatggagttgacctcggagtgcgctacctcccccatgtga

**Table S8. Immunohistochemical antibodies used in this study**

| **Antibodies** | **Company** | **Host species** | **Antibody Numbering** | **Dilution Ratio** |
| --- | --- | --- | --- | --- |
| GP73 | Proteintech, China | Rabbit | 15126-1-AP | 1/200 |
| CD34 | Proteintech, China | Rabbit | 14486-1-AP | 1/200 |
| Ki-67 | Cst, USA | Mouse | 62548 | 1/100 |
| p-STAT3（Tyr705） | cst, USA | Rabbit | 9145 | 1/100 |
| p-STAT3（Ser727） | abways, China | Rabbit | CY6500 | 1/100 |
| HRP | cst, USA | Rabbit | 8114 | 1/100 |
| HRP | cst, USA | Mouse | 8125 | 1/100 |

**Table S9. Immunofluorescence antibodies used in this study**

| **Antibodies** | **Company** | **Host species** | **Antibody Numbering** | **Dilution Ratio** |
| --- | --- | --- | --- | --- |
| GP73 | Abways, China | Rabbit | cy5578 | 1:100 |
| CD34 | abways, China | Rabbit | CY5196 | 1:100 |
| CD31 | Abcam, UK | Rabbit | ab222783 | 1:100 |
| STAT3 | abways, China | Rabbit | CY5165 | 1:200 |
