# Supplementary material for: Integrating Single-Cell and Spatial Transcriptomics to Uncover and Elucidate GP73-Mediated Pro-Angiogenic Regulatory Networks in Hepatocellular Carcinoma
Source: Research (Wash D C). 2024 Jun 27;7:0387. doi: 10.34133/research.0387 (PMC11208919; doi:10.34133/research.0387)
Supplement: Supplementary 1 — Supplementary Methods Supplementary Results Figs. S1 to S7 Tables S1 to S9 Files S1 to S4 [file research.0387.f1.zip › Supplemental file 1.pdf]

项目申请中涉及动物实验伦理资格审查意见表

NO. KY-2023-290

申请人填写相关信息

申请单位：广西医科大学附属肿瘤医院

申请人：梁嵘　　所在部门：消化肿瘤内科　　联系方式：18677070811

申请项目名称：GP73 促进肝癌血管生成的表观遗传修饰机制研究

实验目的：验证动物体内动态表观修饰机制协同 GP73 促进肝癌血管生成的机制

拟用动物情况

动物来源：广西医科大学实验动物中心

品种品系：BALB/c 小鼠、BALB/c 裸鼠、C57BL/6 小鼠、SD 大鼠

等级：SPF 级

规格：5-7 周龄

数量：80 只(♀ 40 只；♂ 40 只)注：

拟申请日期：2024 年 01 月 01 日　　拟结束日期：2027 年 12 月 31 日

1.实验要点，实验方法、观测指标：

构建转基因小鼠肝癌模型：构建 $GP73^{-/-}$ 小鼠模型，野生型 Balb/c 小鼠对照，腹腔注射 25 mg/kg 二乙基亚硝胺，每周 1 次，共 4 次，期间用超声进行肝脏动态观测成瘤情况，于 24 周终止实验。

建立人肝癌裸鼠皮下-肝原位移植瘤模型：选用 6 周龄 BALB/c 雄性裸小鼠，无菌条件下去除对数生长期的 MHCC-97H 细胞分别接种于右侧颈部。经 7-10d 成瘤后，将瘤块取出切割。将裸鼠全麻（20mg/kg 的戊巴比妥钠）后，在左上腹做长 1cm 横切口，暴露肝脏，将上述已切割好的人源肝癌组织块植入，缝合固定于肝实质中。成瘤后分组皮下注射靶向 GP73 的 siRNA 药物及对照生理盐水。

采用 SD 大鼠和 C57 小鼠尾静脉注射靶向 GP73 的 siRNA 药物，对照生理盐水，进行单次毒性研究。

2.实验终点或仁慈终点：

负荷重量大于鼠体重 1/10 或者移植瘤接种 5 周后。

3.实验结束后处死动物的方法：

安乐死：腹腔注射致死量的戊巴比妥钠（三倍于常规麻醉量）

4.动物替代、减少动物用量、降低动物痛苦伤害的主要措施等：

通过减少实验分组、统计学预测所需最少样本数、缩短实验观察周期、麻醉下操作、实验终点安乐死等方法（3R）。

审查结果（是否同意申请人的实验方案）

审查意见：　　☑同意　　□不同意

盖章：广西医科大学附属肿瘤医院 动物试验伦理委员会

备注：项目申请人除上交《涉及动物实验伦理资格审查意见表》外，还需提供项目正式申请书中涉及动物实验研究方案及技术路线等内容的纸质材料以供存档。
