# Supplementary material for: Integrating Single-Cell and Spatial Transcriptomics to Uncover and Elucidate GP73-Mediated Pro-Angiogenic Regulatory Networks in Hepatocellular Carcinoma
Source: Research (Wash D C). 2024 Jun 27;7:0387. doi: 10.34133/research.0387 (PMC11208919; doi:10.34133/research.0387)

Figure 4C

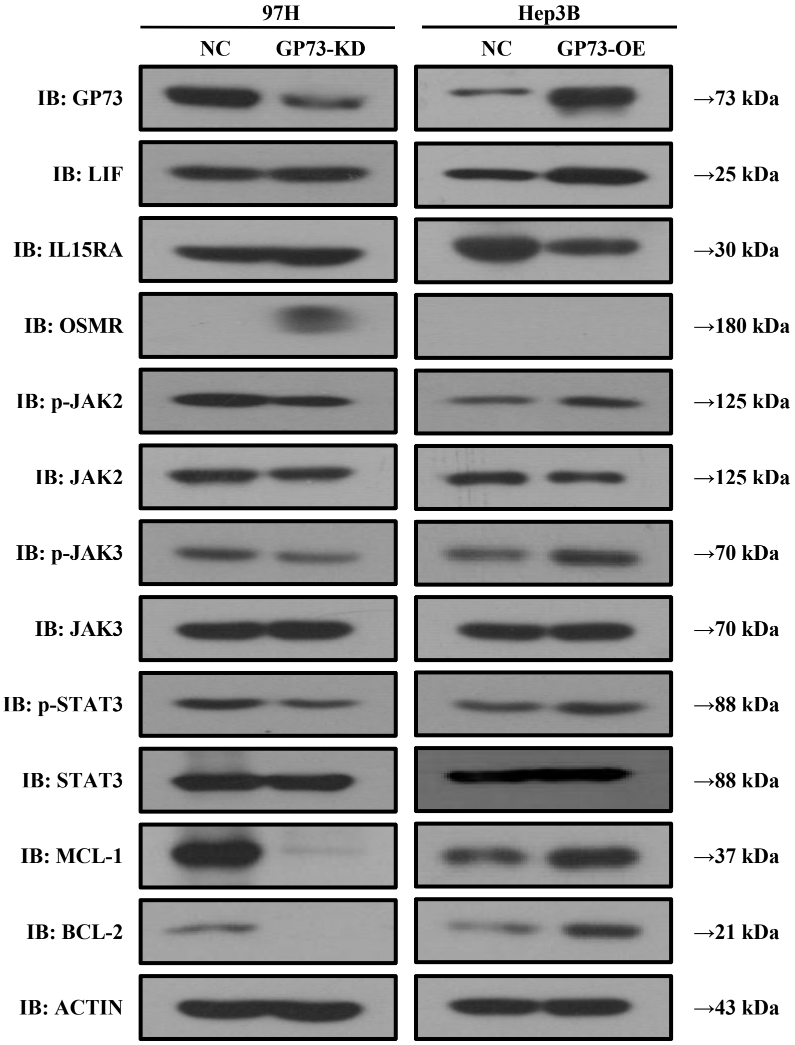

Western blot detection of key genes expressions in the JAK2/STAT3 pathway in MHCC97H-GP73-KD, MHCC97H-GP73-NC, Hep3B-GP73-OE and Hep3B-GP73-NC cells. Data were representative of three similar observations or were shown as the mean ± SD of three experiments

Figure4C

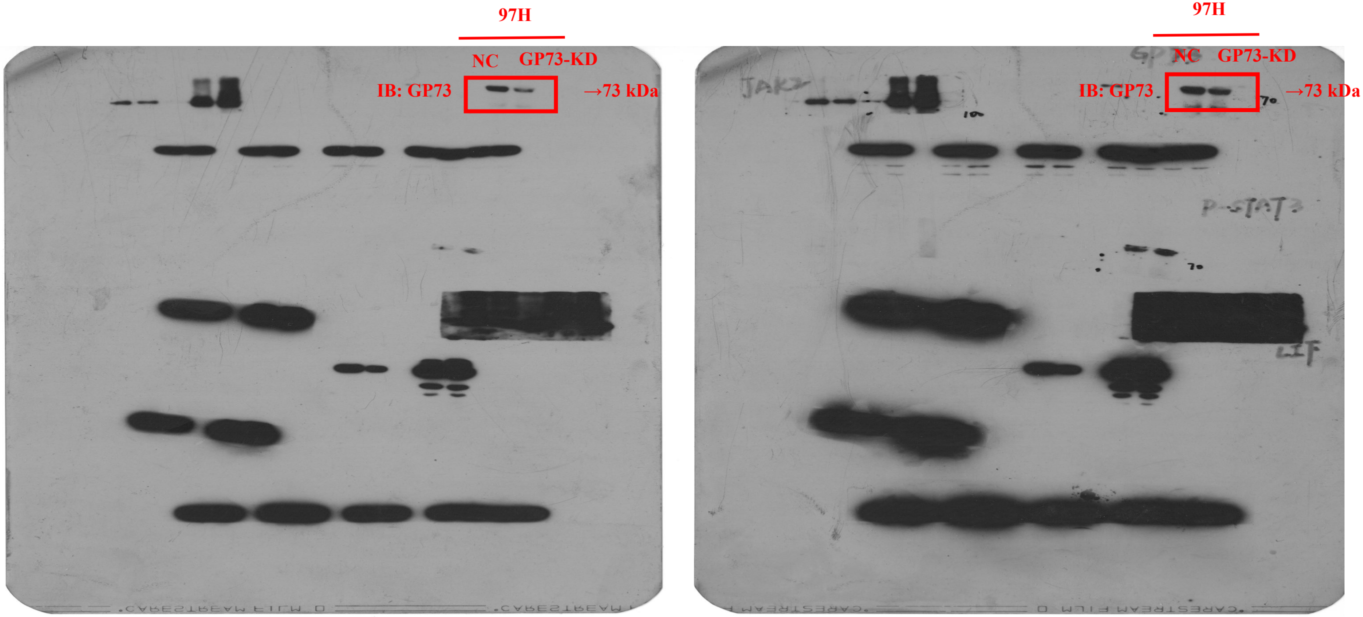

Figure4C

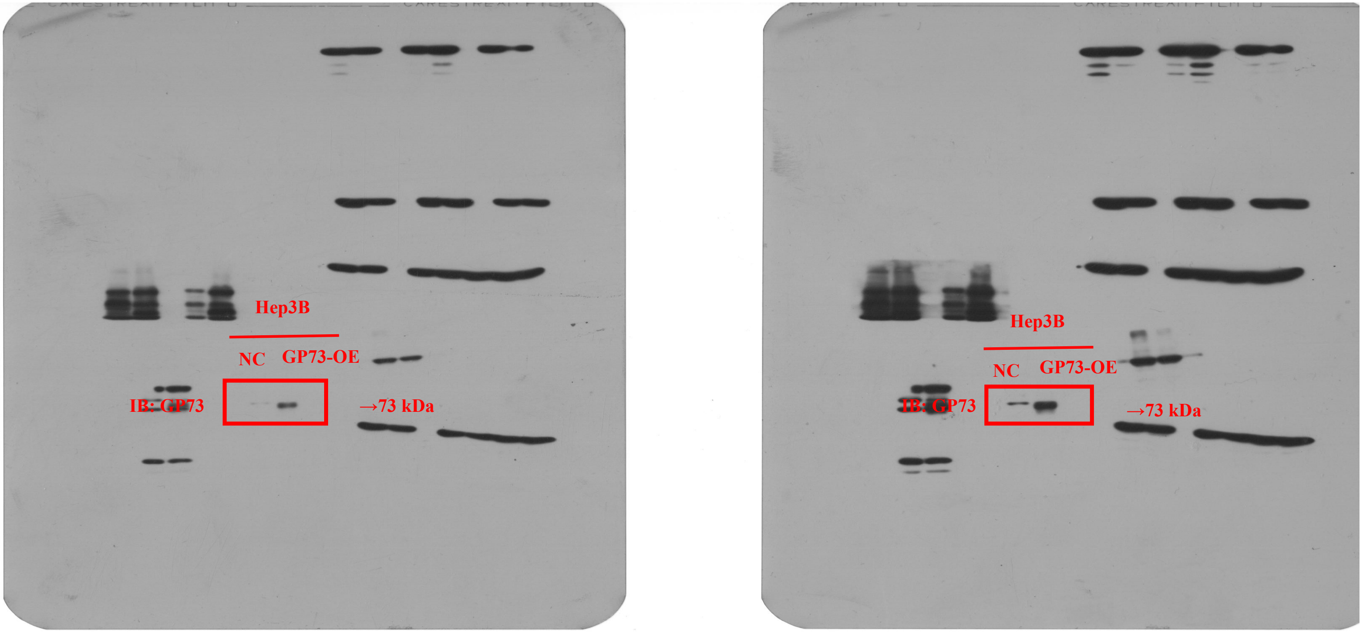

Figure4C

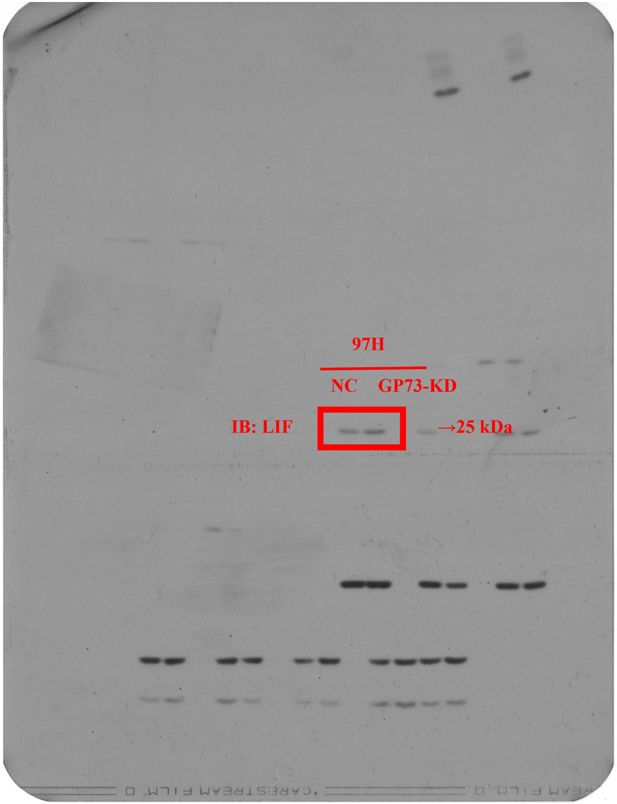

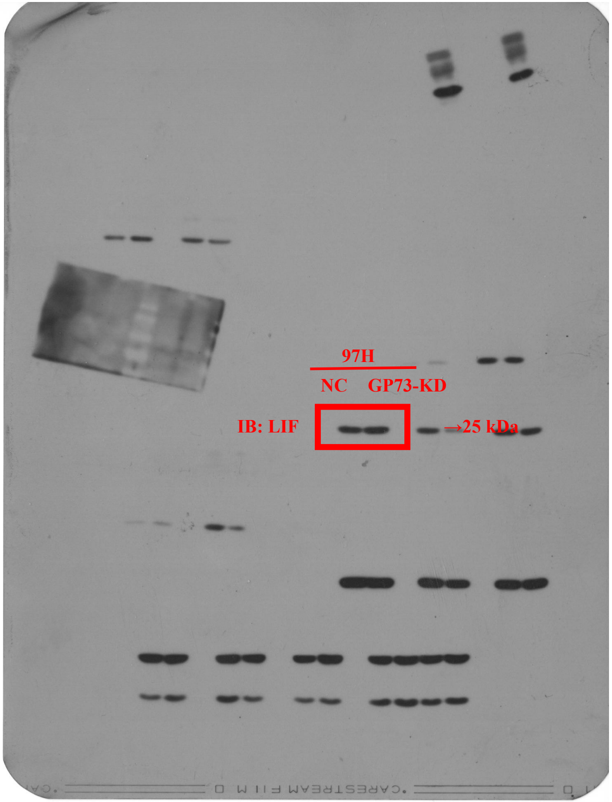

Figure4C

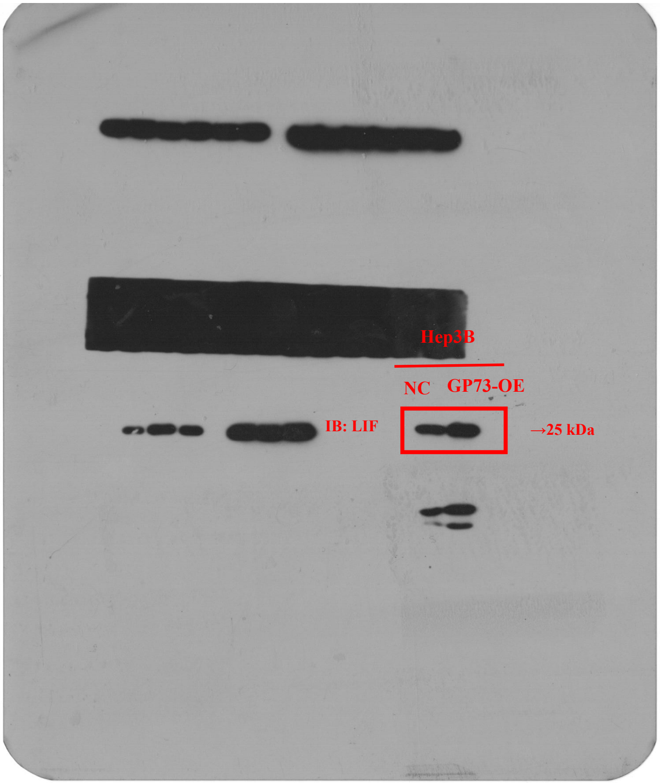

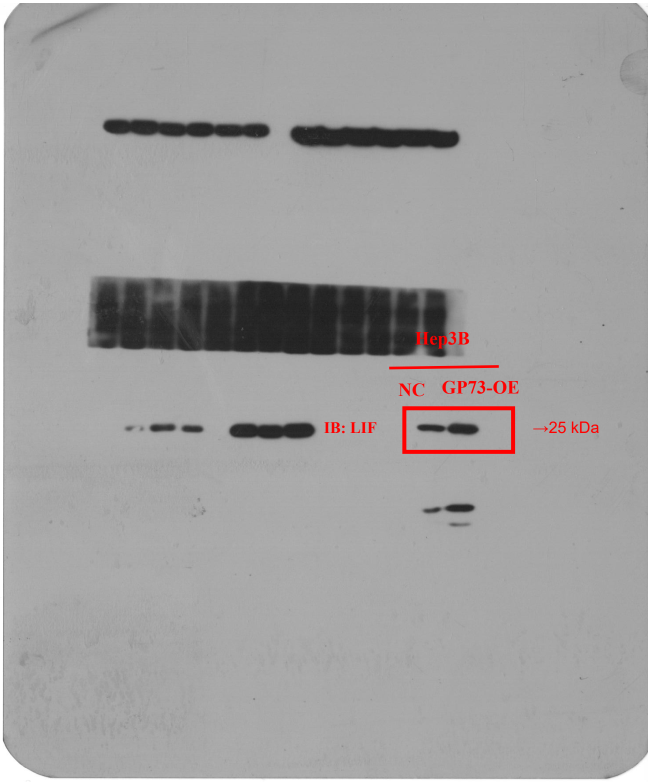

Figure4C

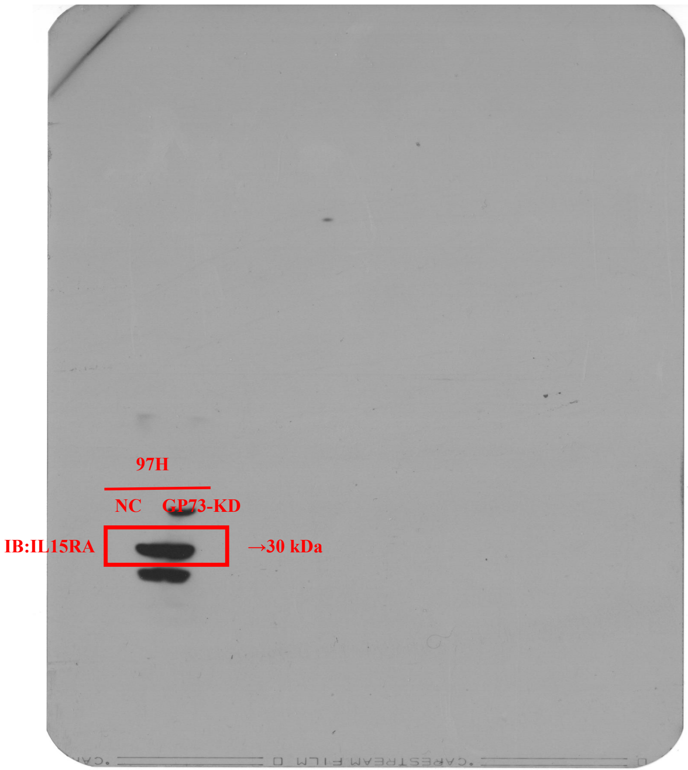

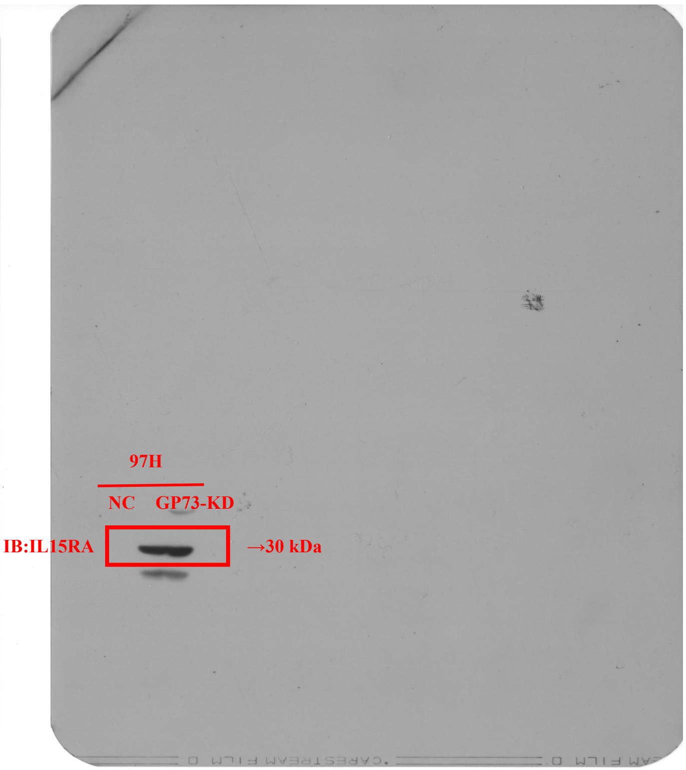

Figure4C

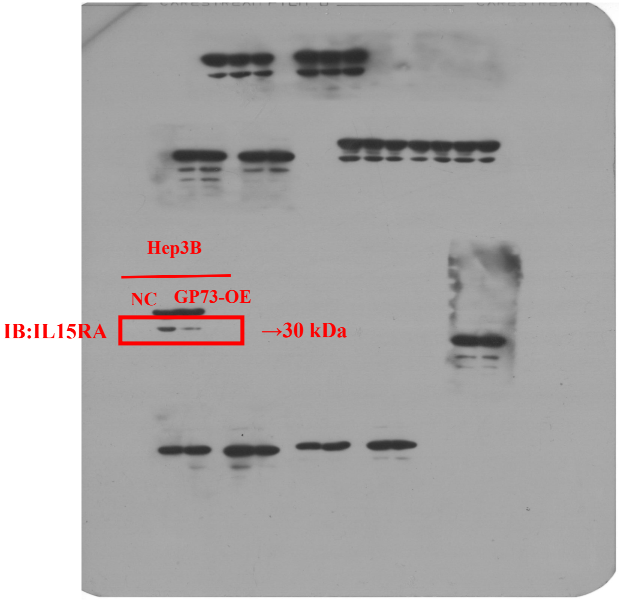
Hep3B
NC
GP73-OE
IB:IL15RA
→30 kDa

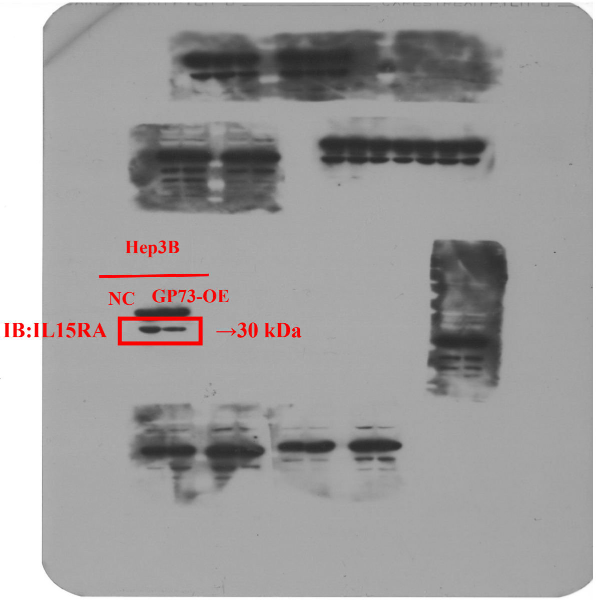
Hep3B
NC
GP73-OE
IB:IL15RA
→30 kDa

Figure4C

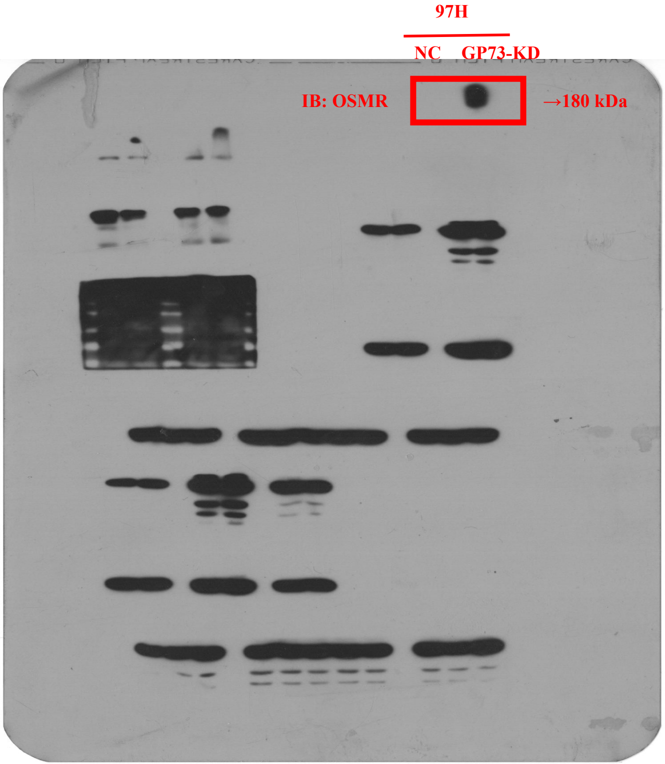

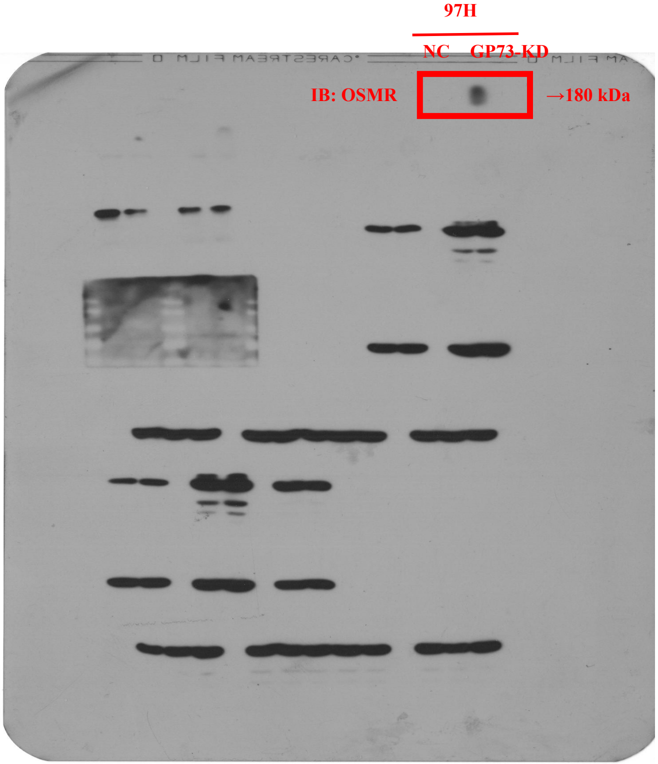

Figure4C

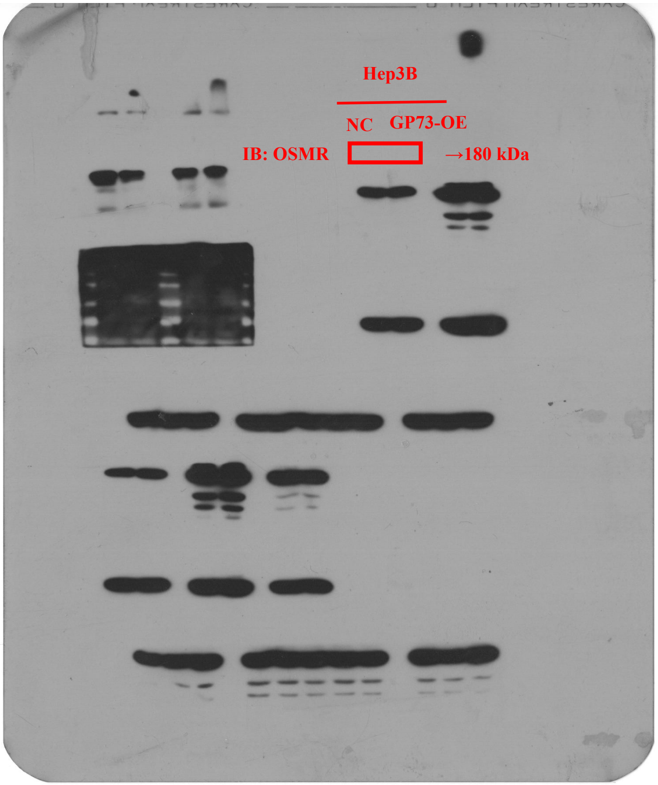
Hep3B
NC
GP73-OE
IB: OSMR
→180 kDa

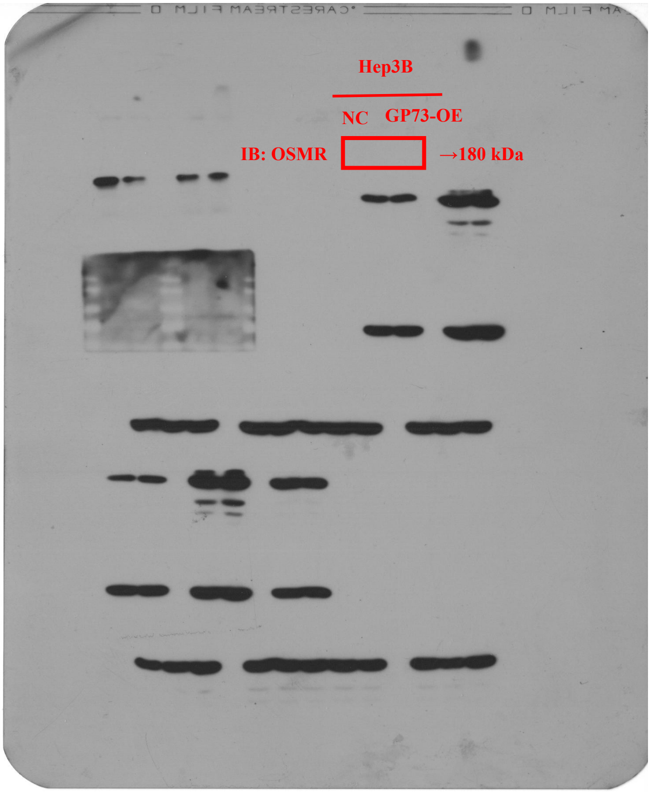
Hep3B
NC
GP73-OE
IB: OSMR
→180 kDa

Figure4C

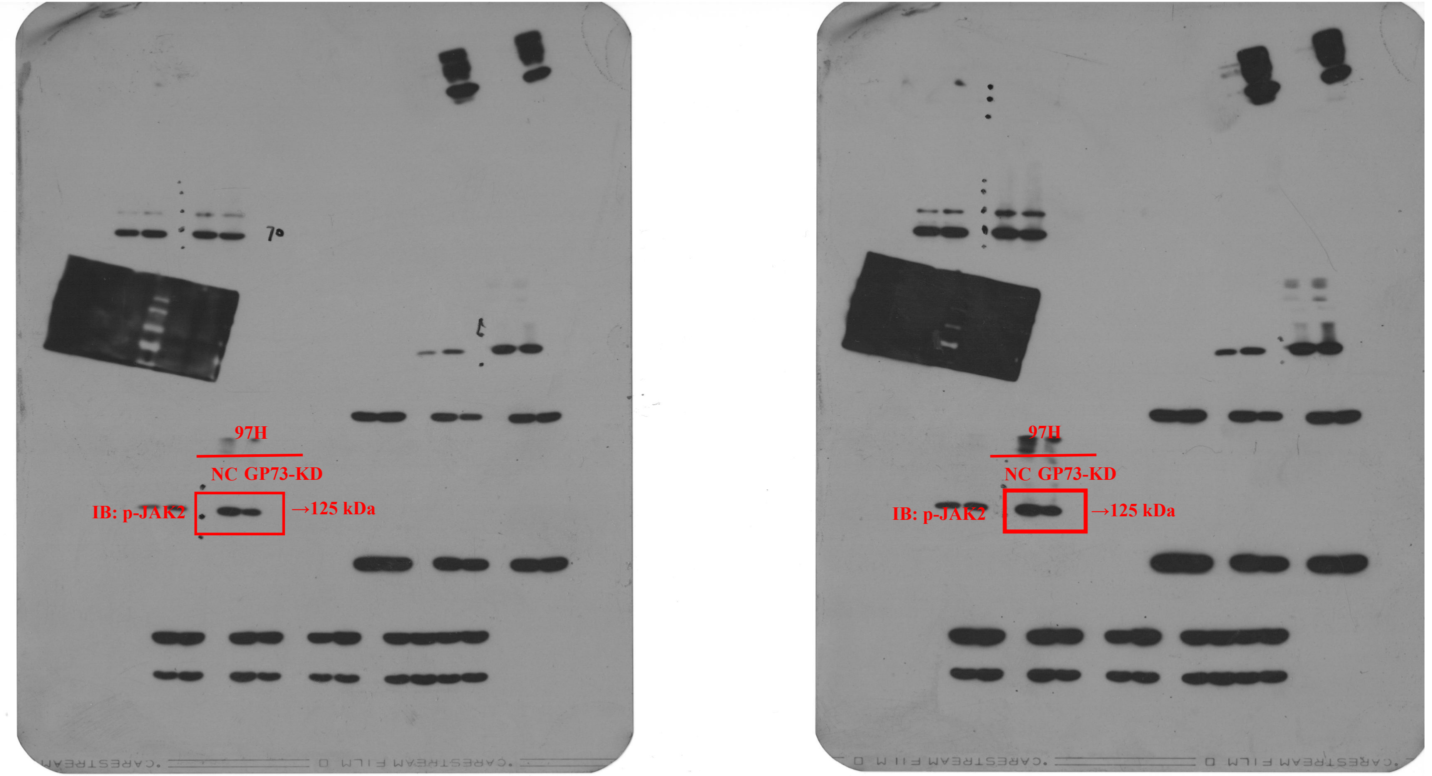
70
97H
NC GP73-KD
IB: p-JAK2
→125 kDa
97H
NC GP73-KD
IB: p-JAK2
→125 kDa

Figure4C

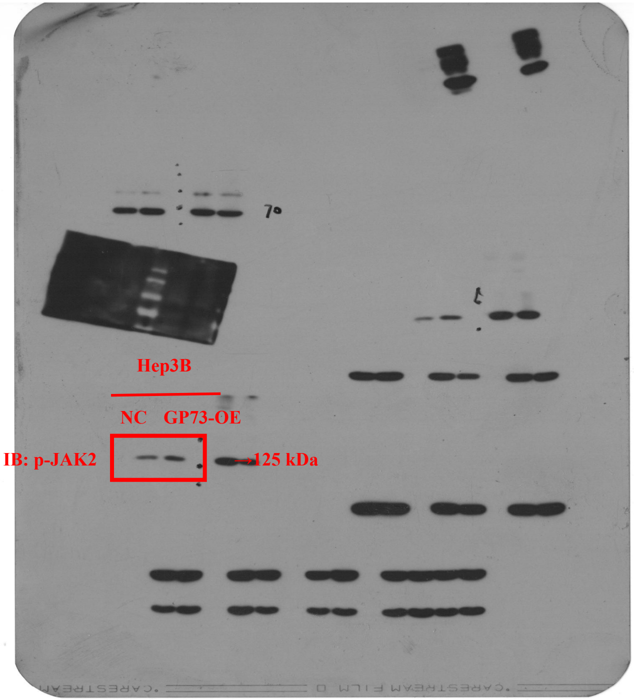

70
Hep3B
NC GP73-OE
IB: p-JAK2
→125 kDa

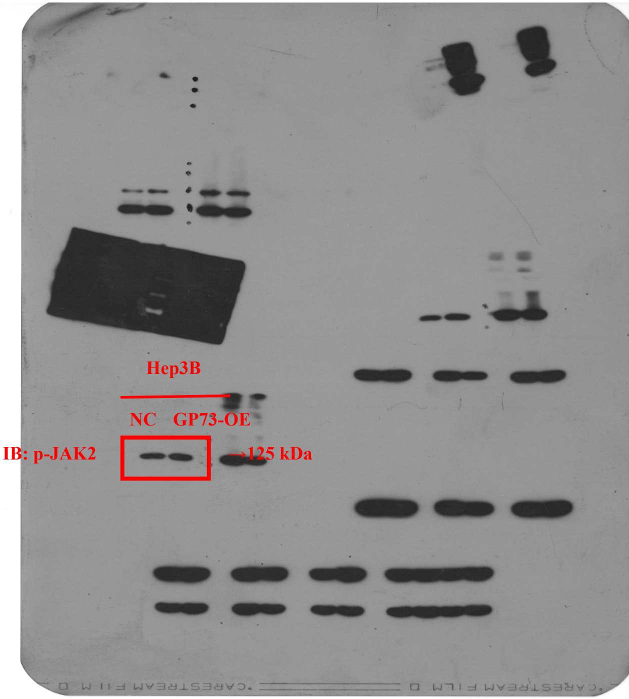

Hep3B
NC GP73-OE
IB: p-JAK2
→125 kDa

Figure4C

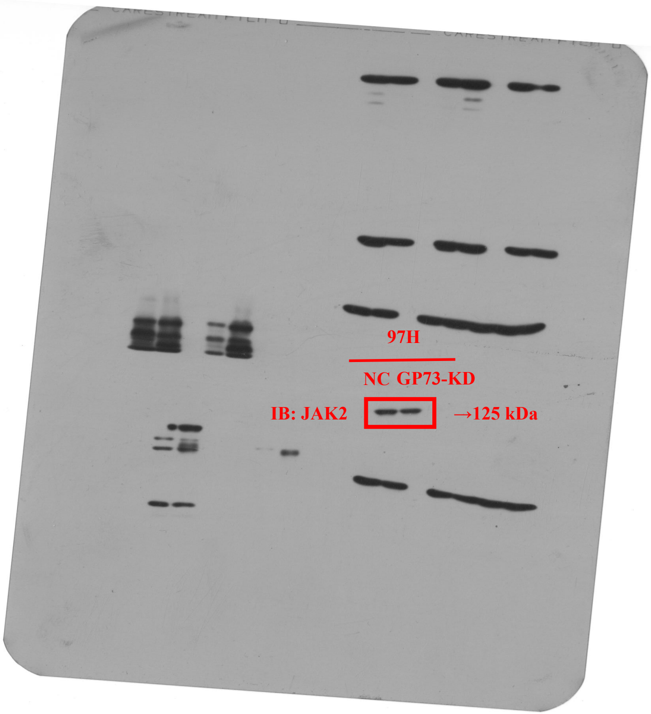

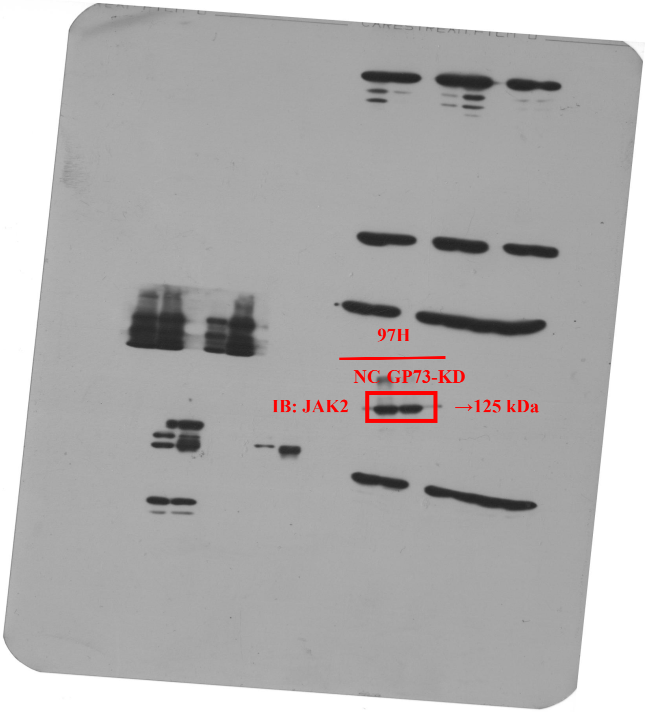

Figure4C

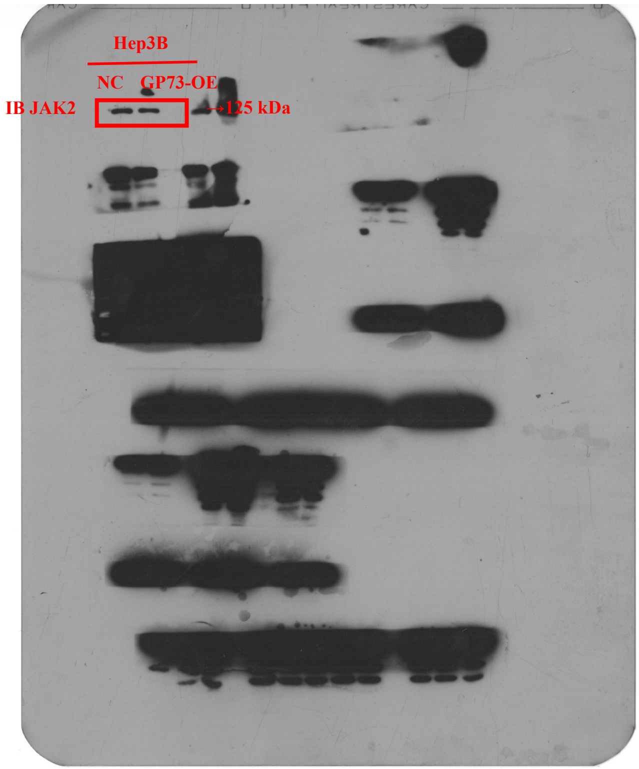
Hep3B
NC
GP73-OE
IB JAK2
→125 kDa

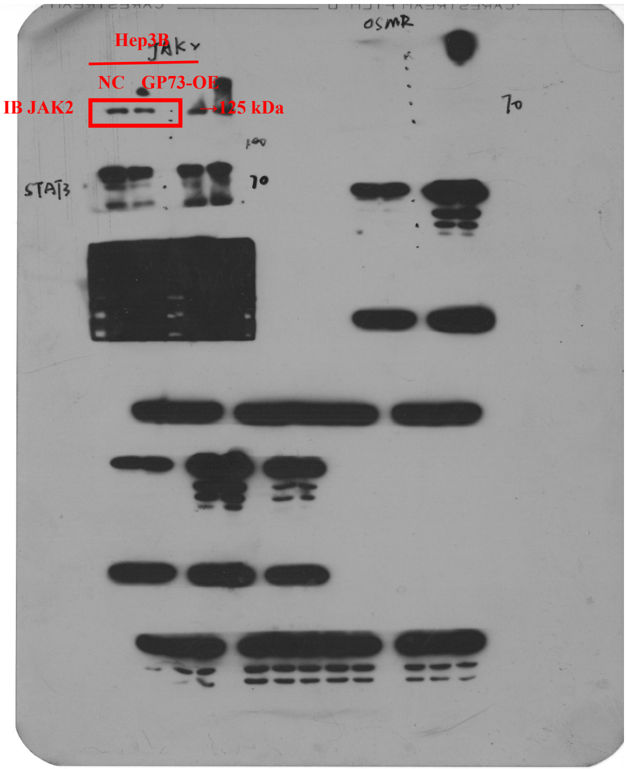
Hep3B
NC
GP73-OE
IB JAK2
→125 kDa
OSMR
STAT3
70
100
70

Figure4C

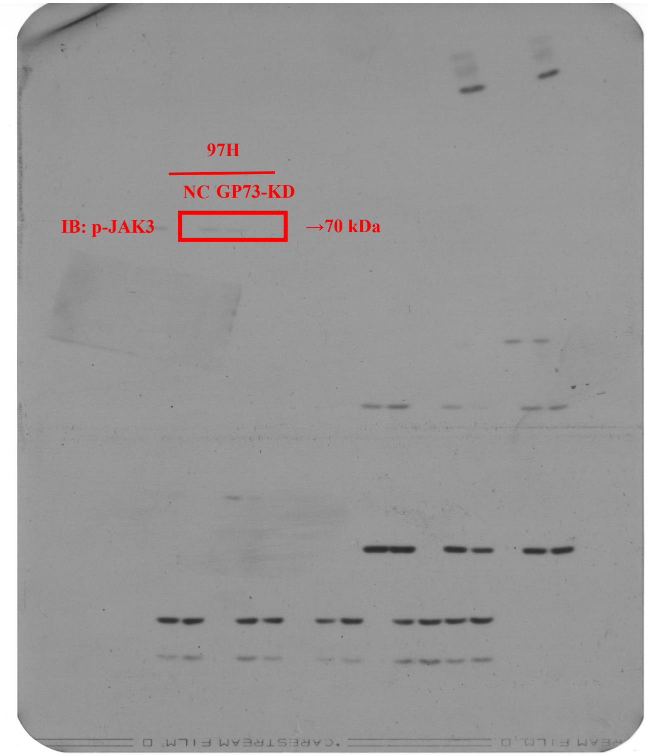
97H
NC GP73-KD
IB: p-JAK3
→70 kDa
CARESTREAM FILM

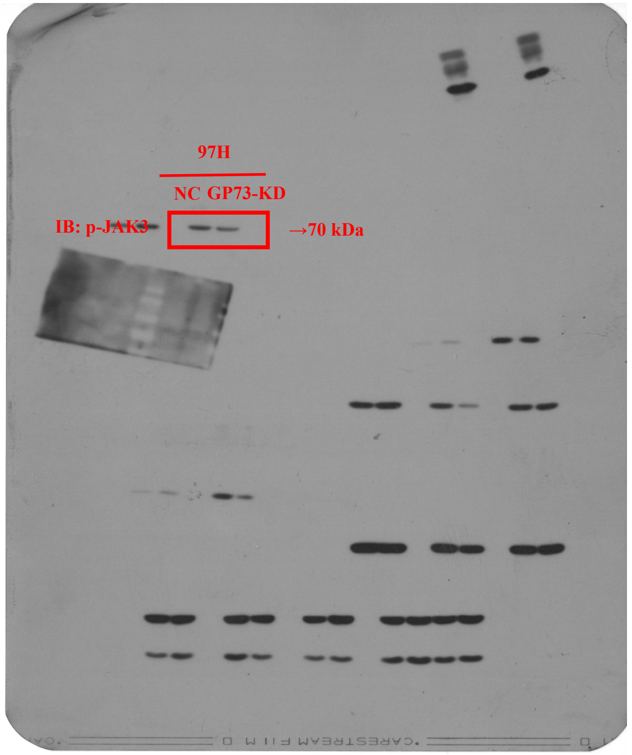
97H
NC GP73-KD
IB: p-JAK3
→70 kDa
CARESTREAM FILM

Figure4C

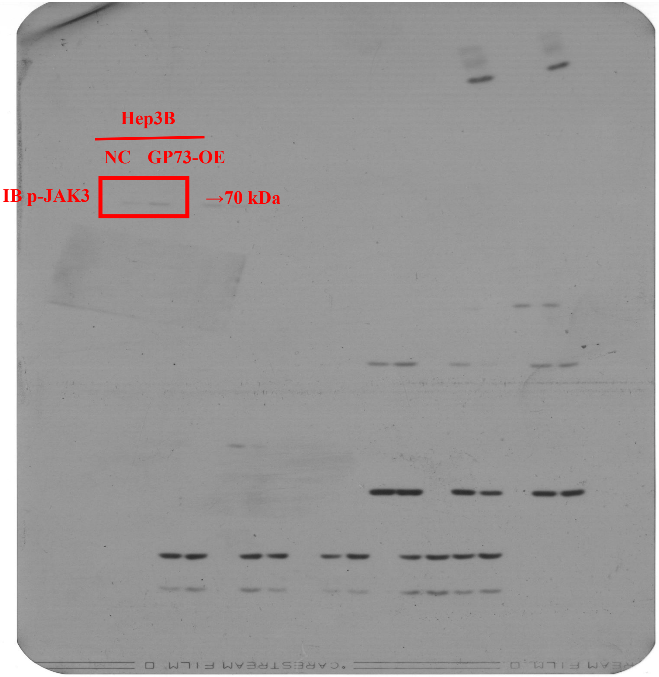
Hep3B
NC
GP73-OE
IB p-JAK3
→70 kDa

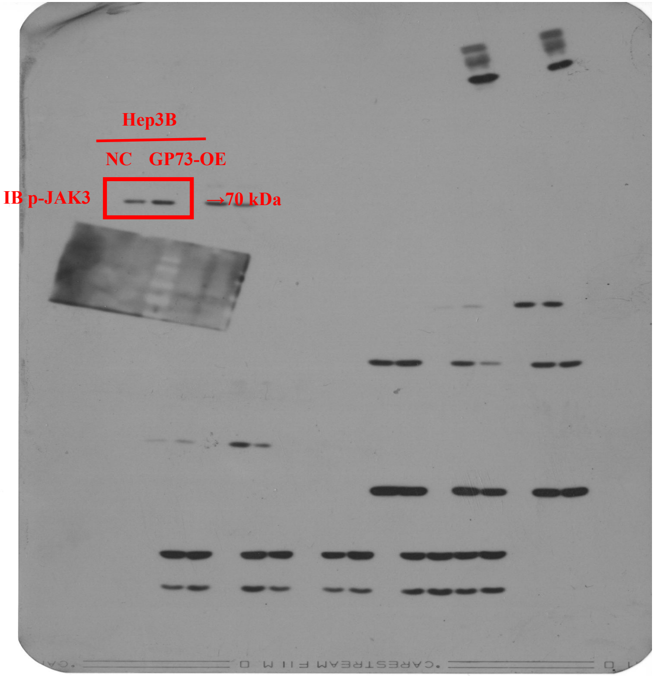
Hep3B
NC
GP73-OE
IB p-JAK3
→70 kDa

Figure4C

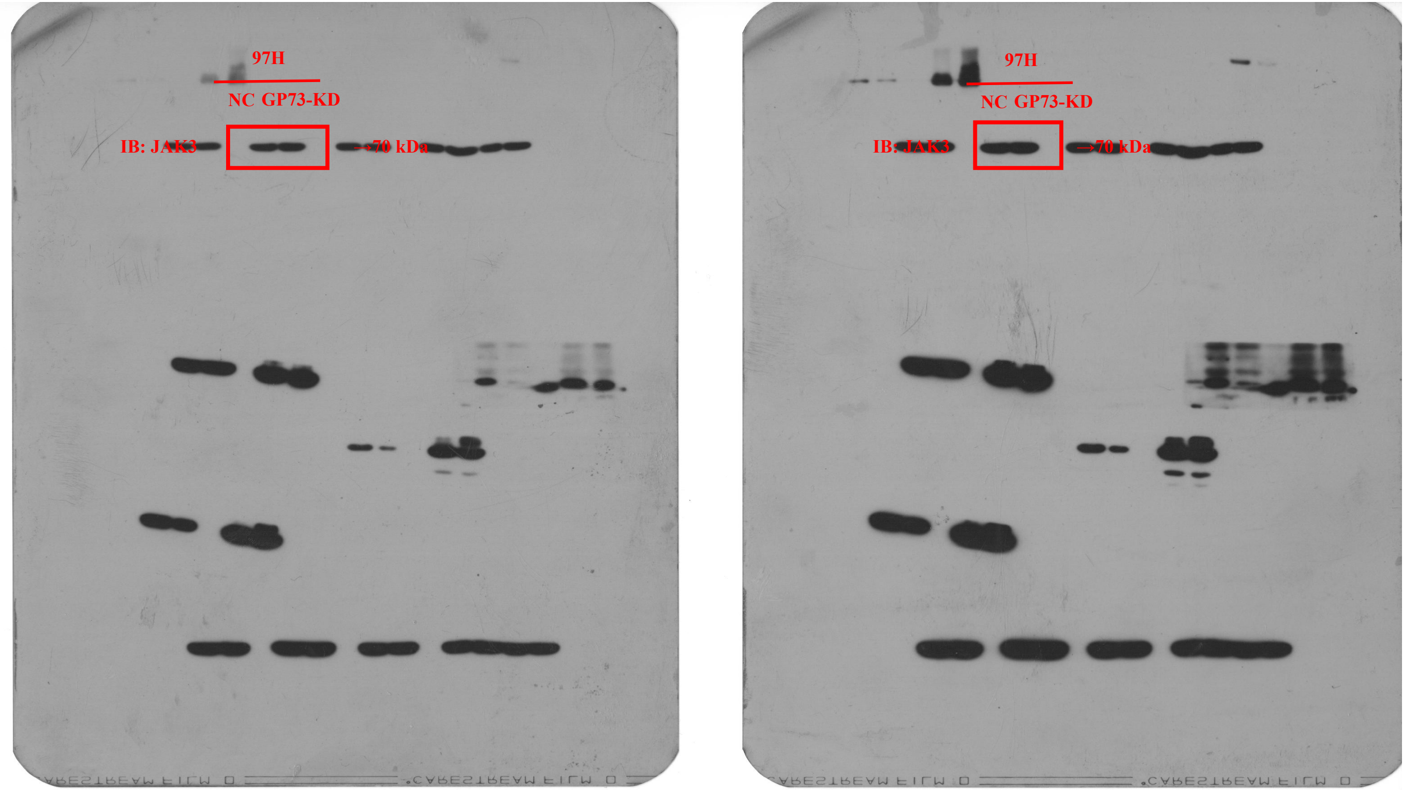

Figure4C

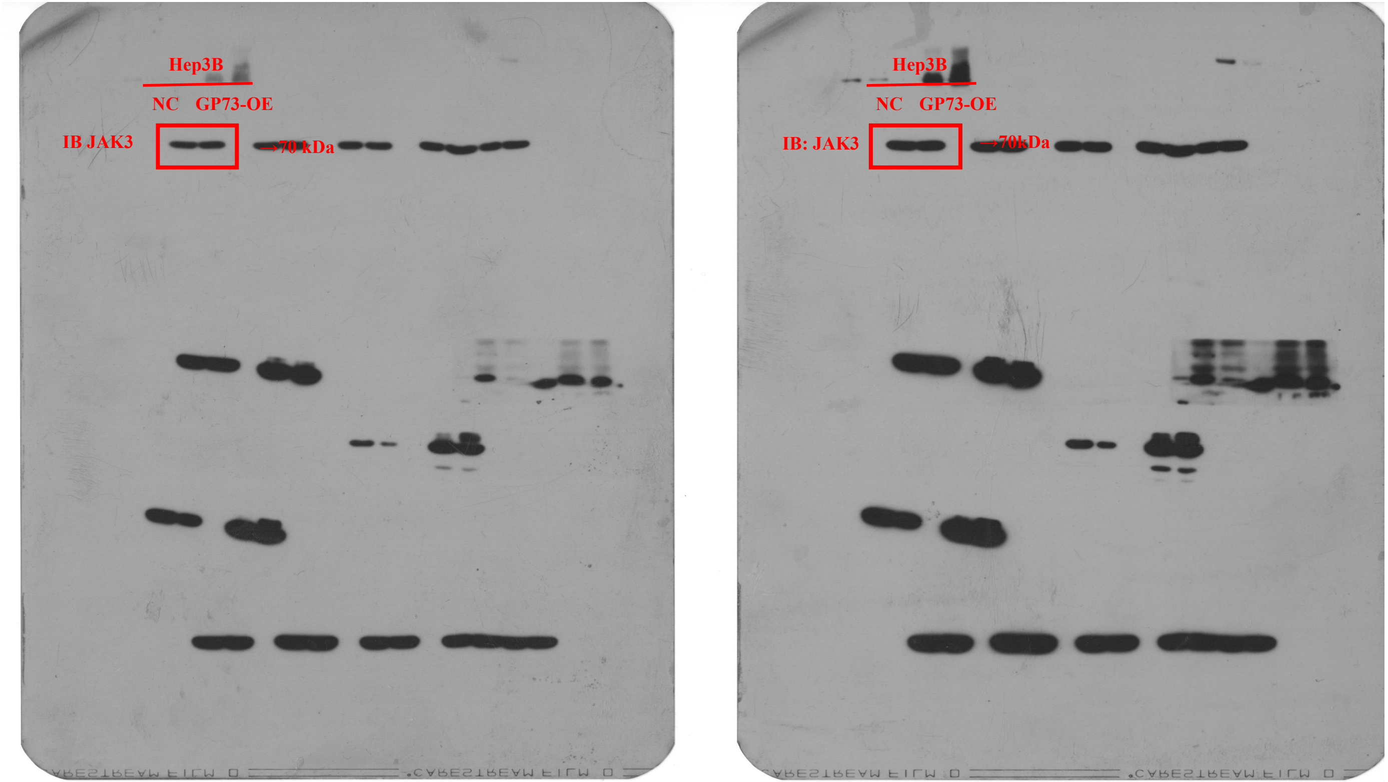

Figure4C

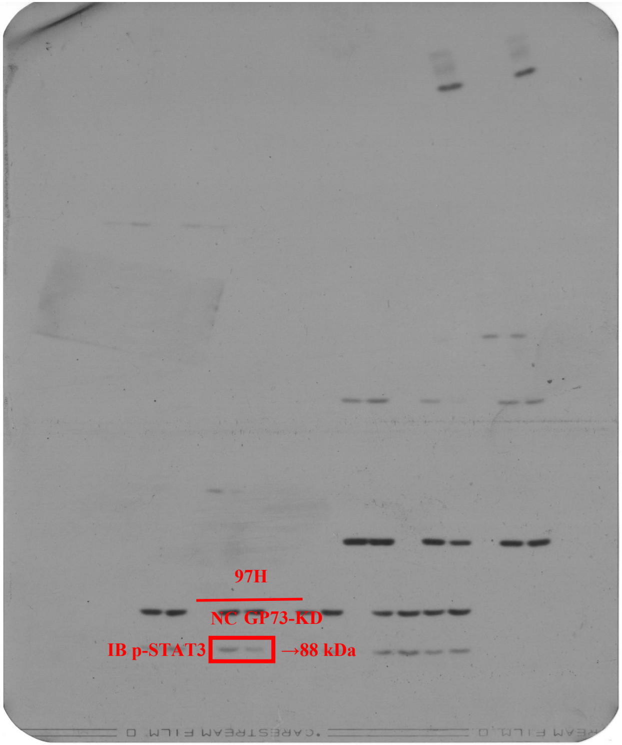

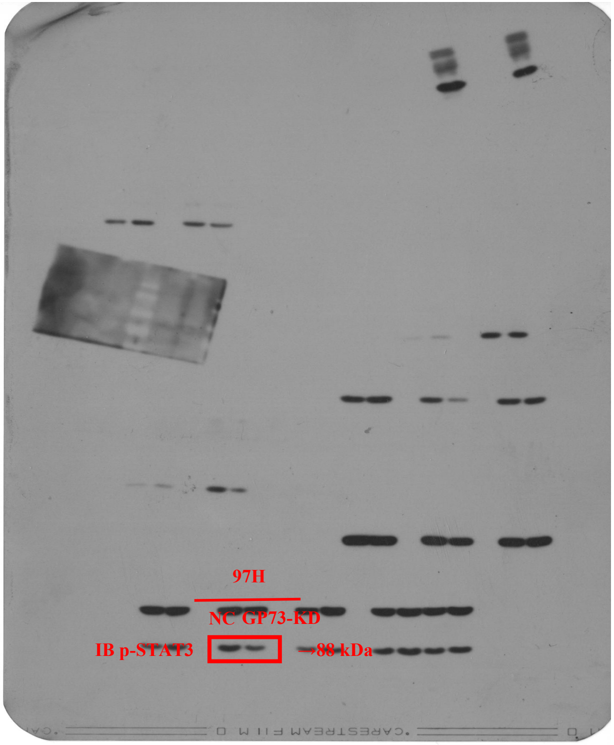

Figure4C

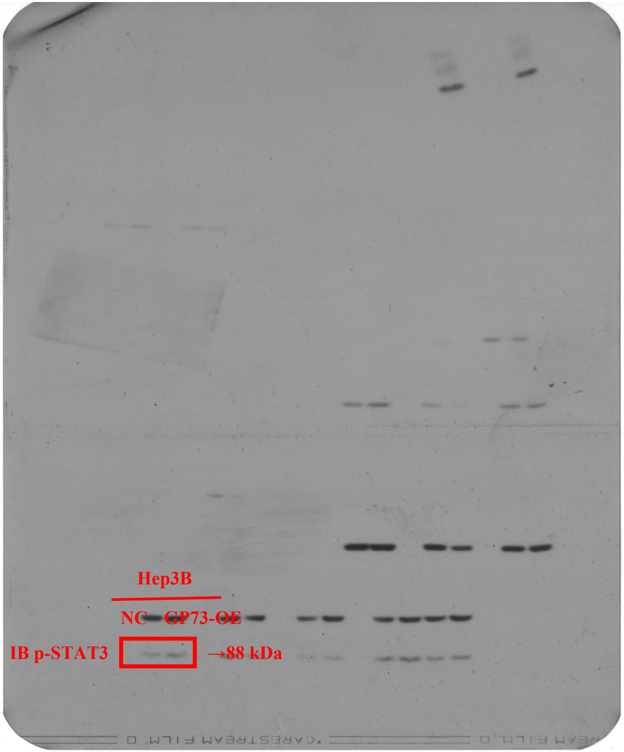
Hep3B
NC GP73-OE
IB p-STAT3
→88 kDa

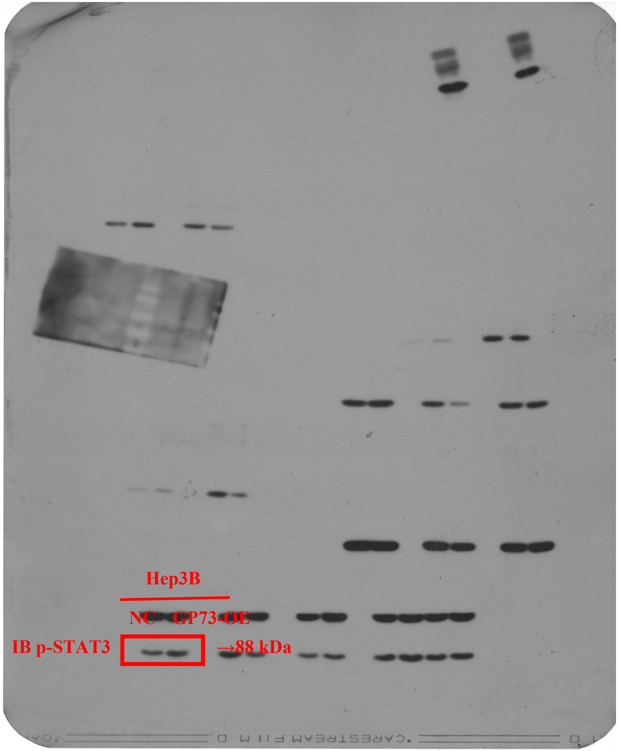
Hep3B
NC GP73-OE
IB p-STAT3
→88 kDa

Figure4C

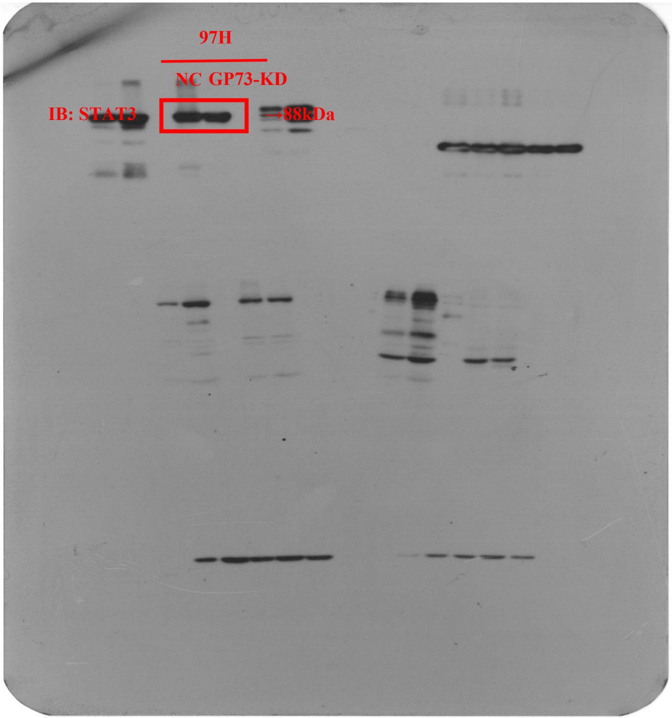

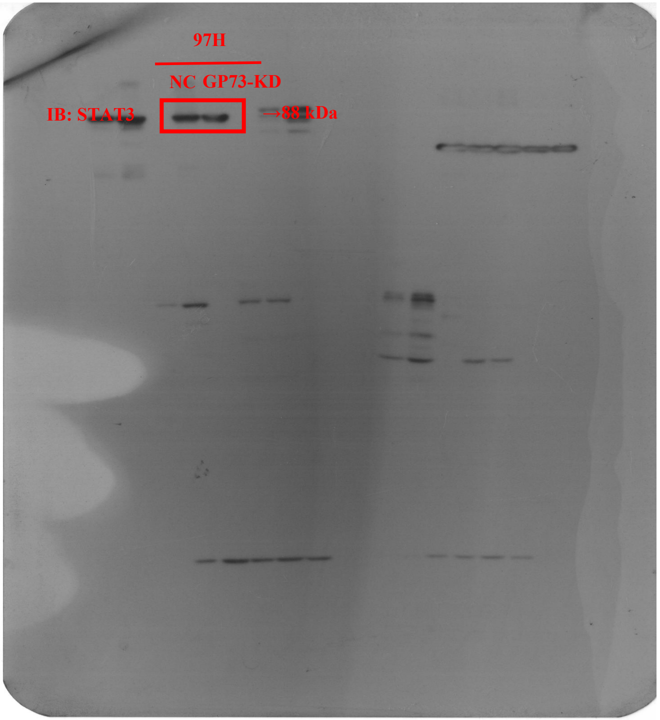

Figure4C

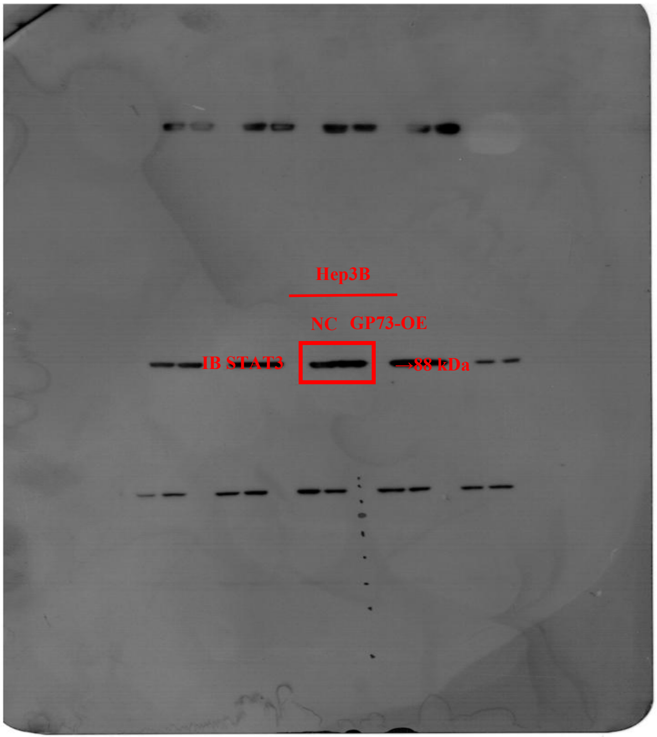
Hep3B
NC GP73-OE
IB STAT3
→88 kDa

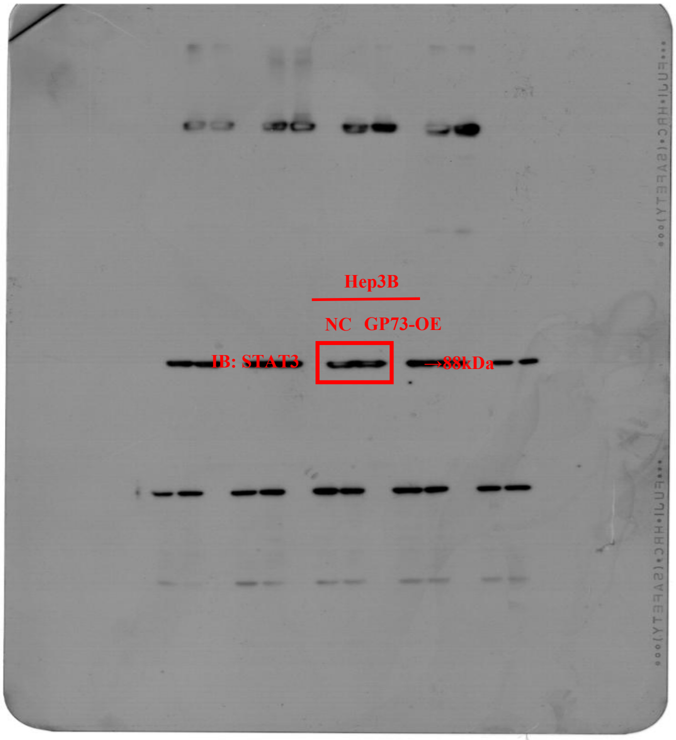
Hep3B
NC GP73-OE
IB: STAT3
→88kDa

Figure4C

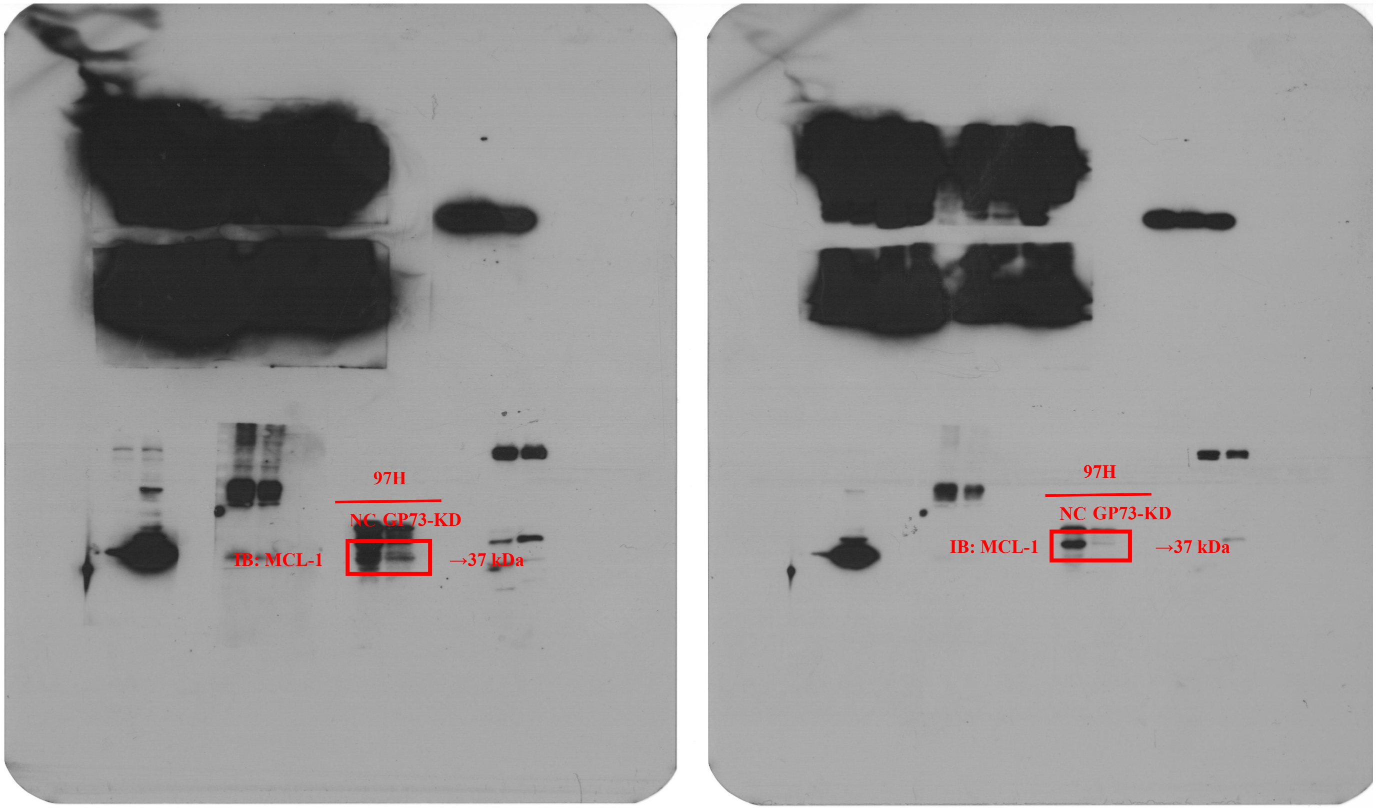

Figure4C

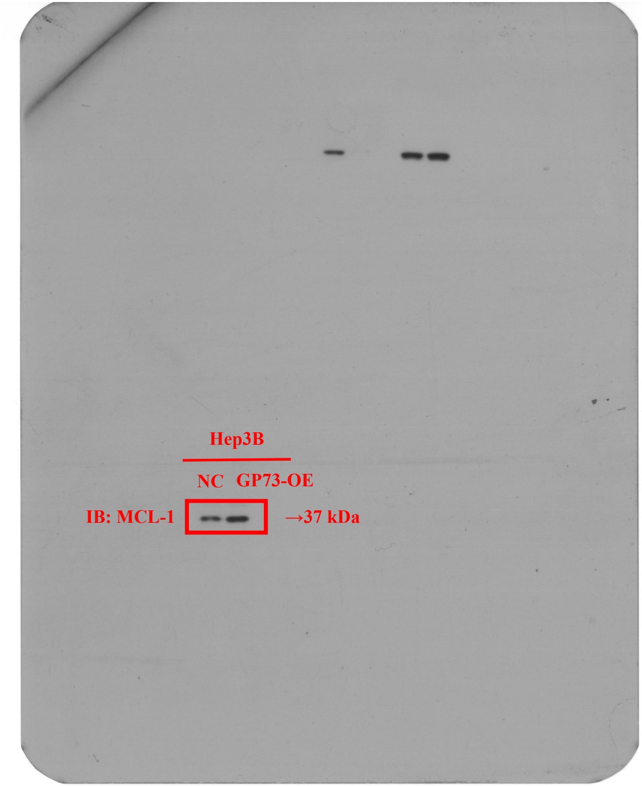

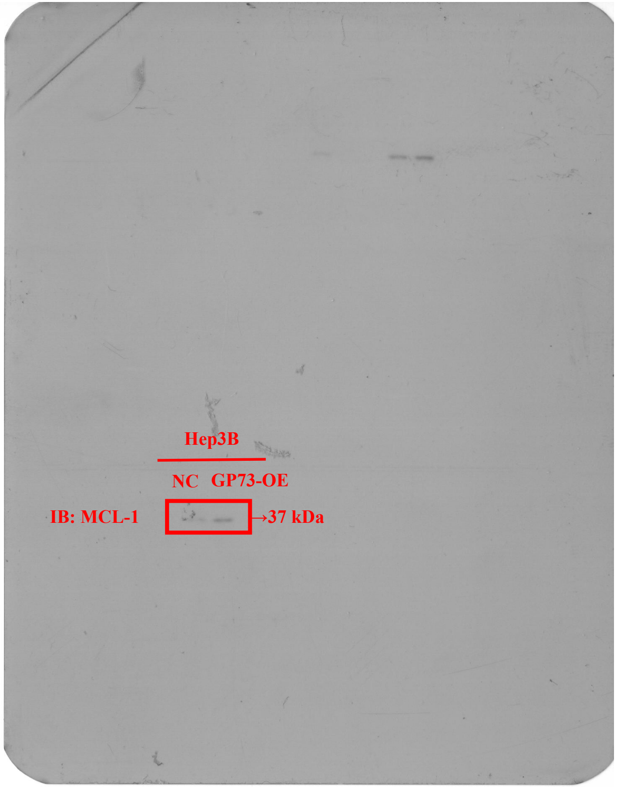

Figure4C

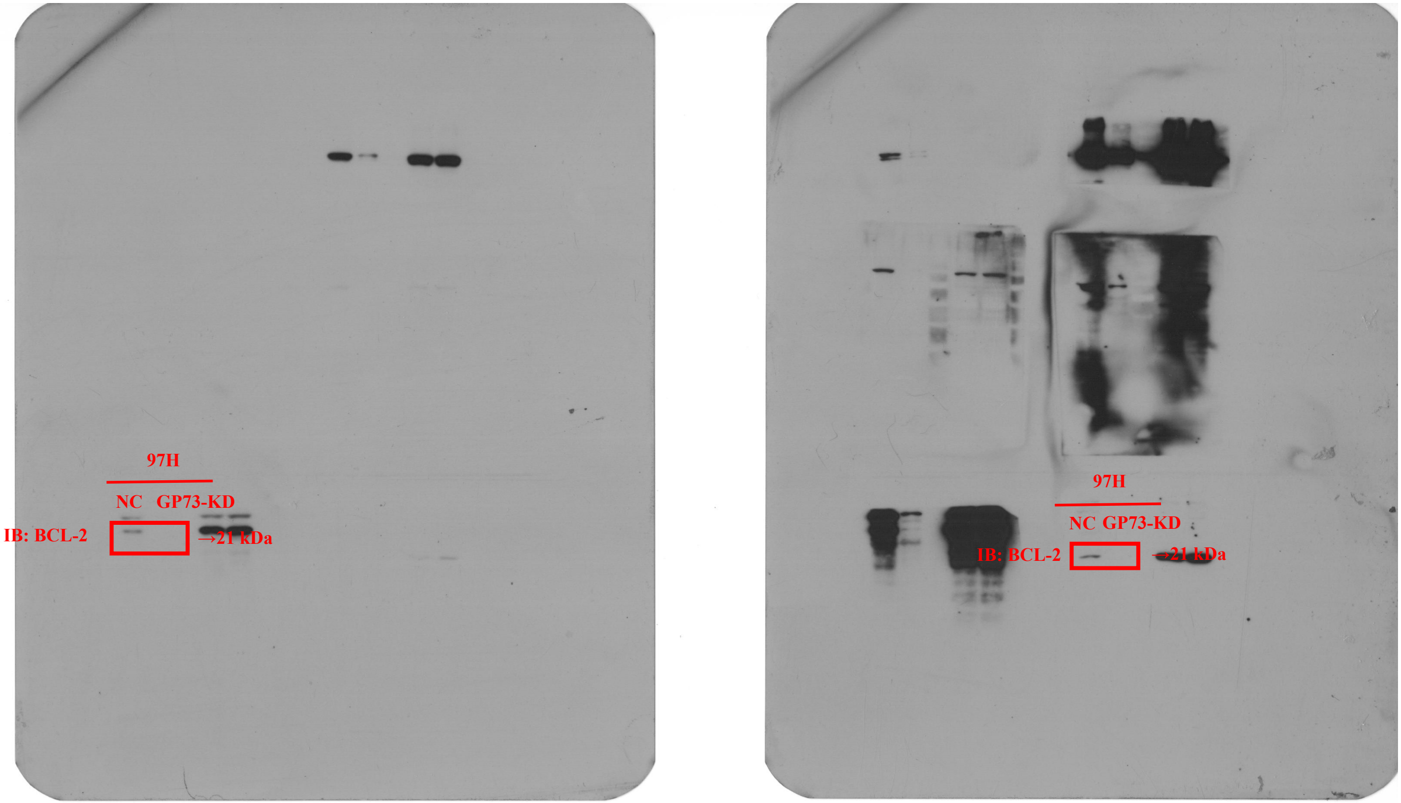

Figure4C

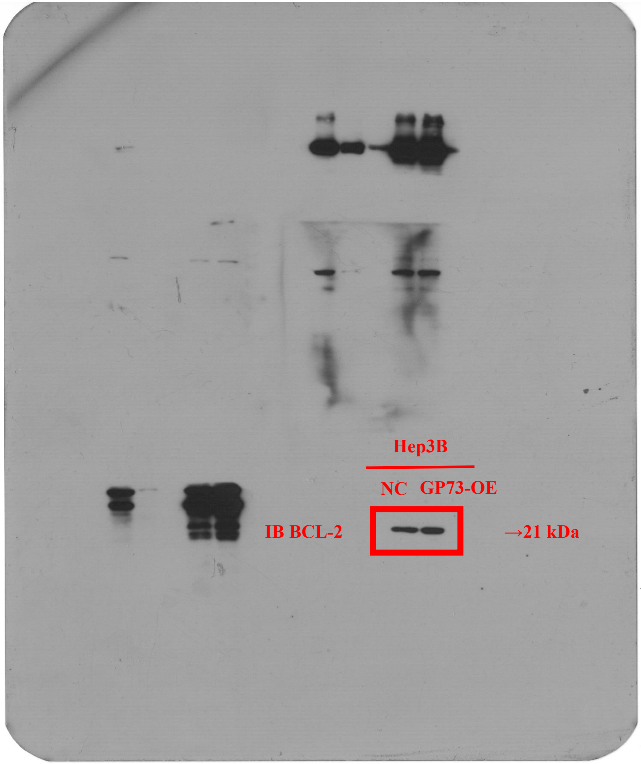

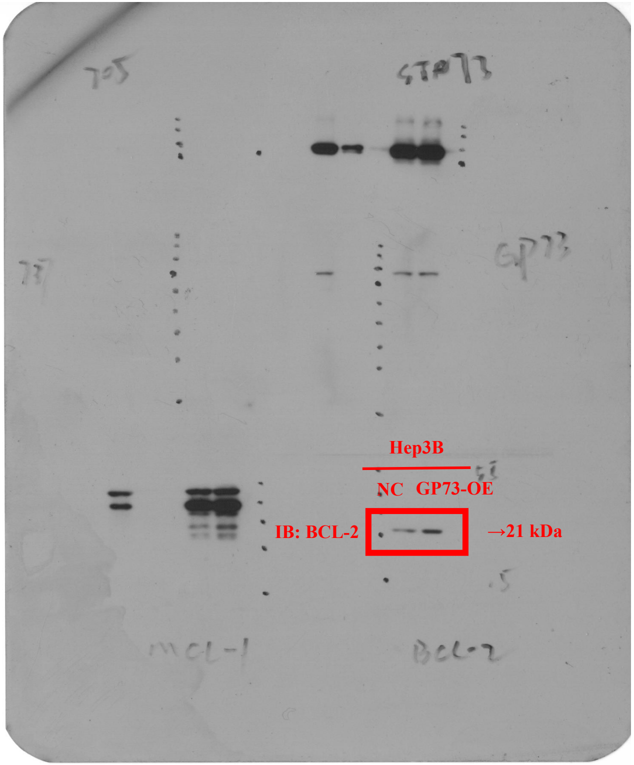

Figure4C

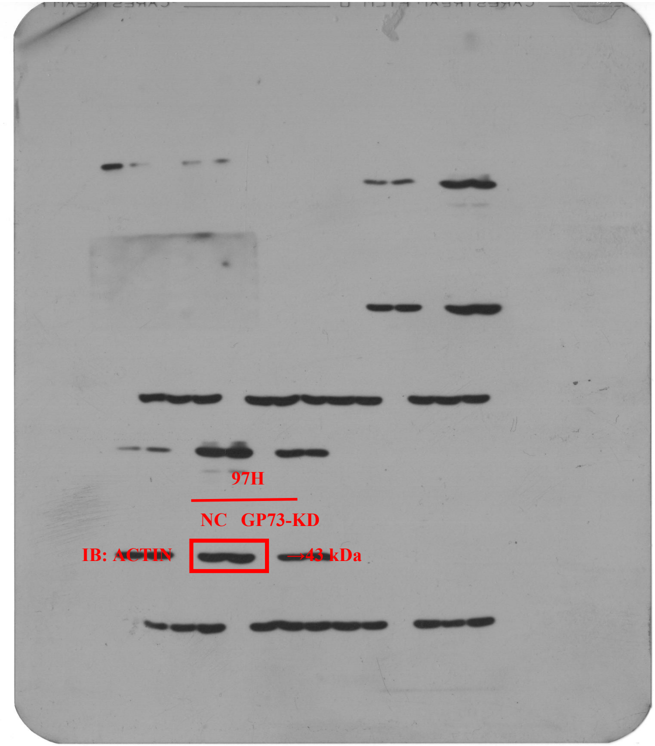

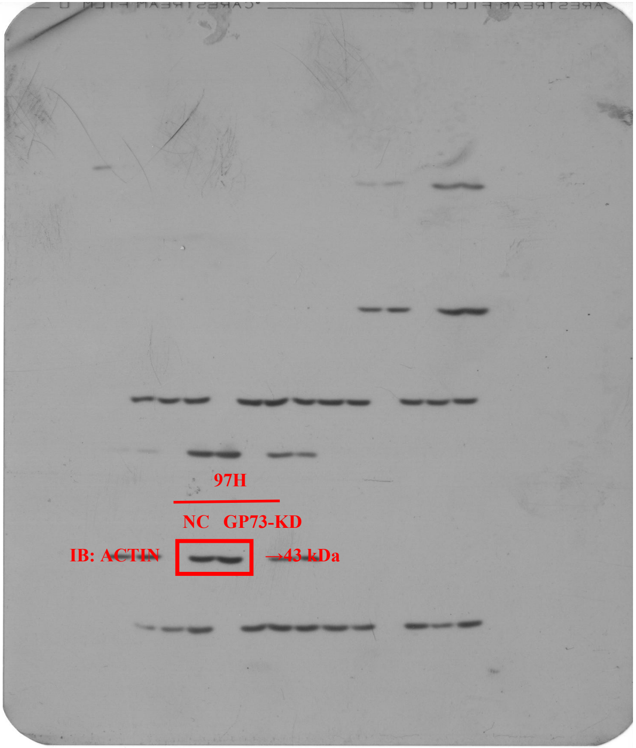

Figure4C

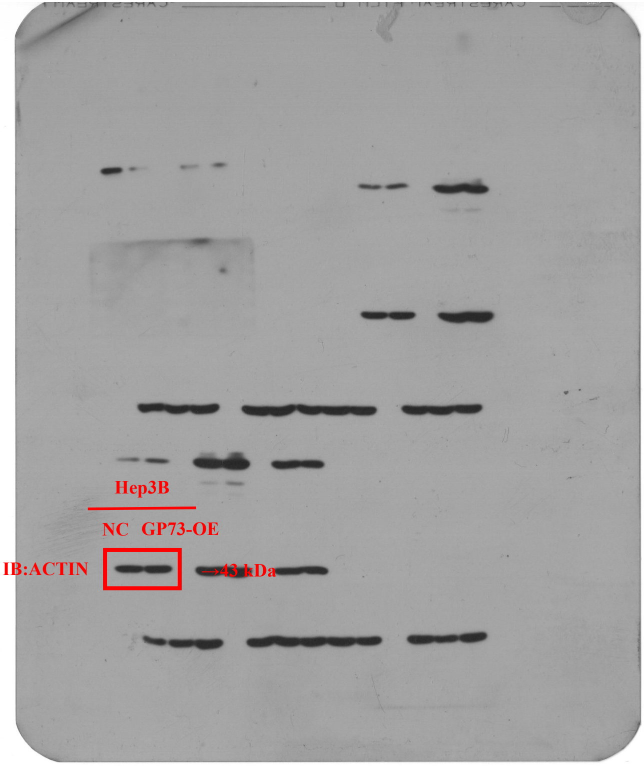
Hep3B
NC GP73-OE
IB:ACTIN
→43 kDa

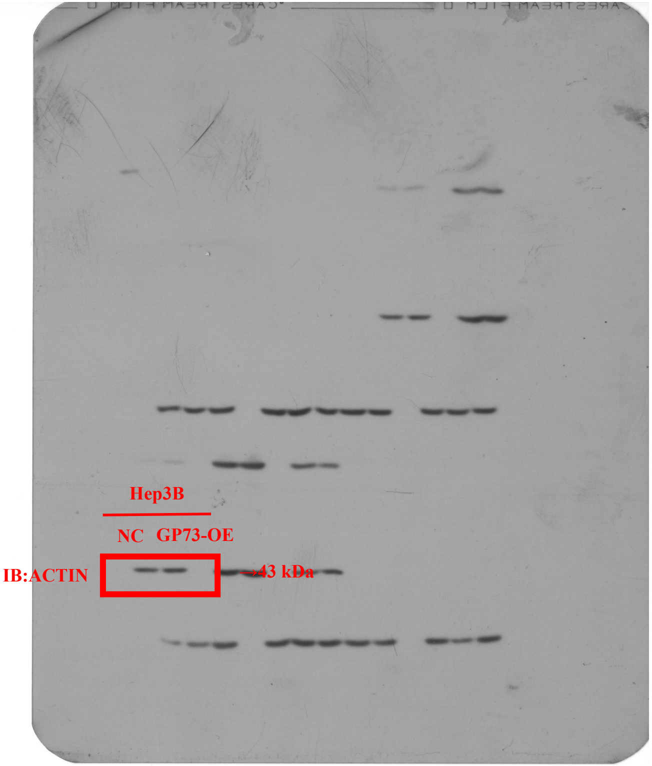
Hep3B
NC GP73-OE
IB:ACTIN
→43 kDa

Figure 5A

Co-IP assays detected the interaction between GP73 and JAK2.

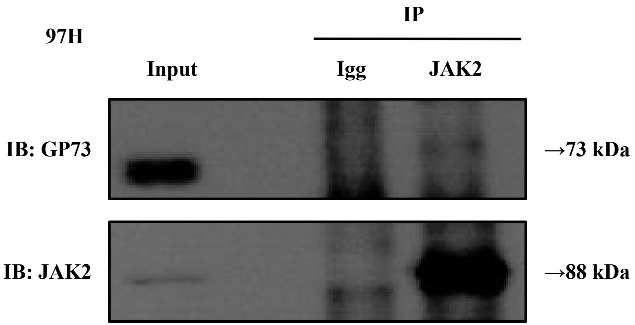

Figure 5A

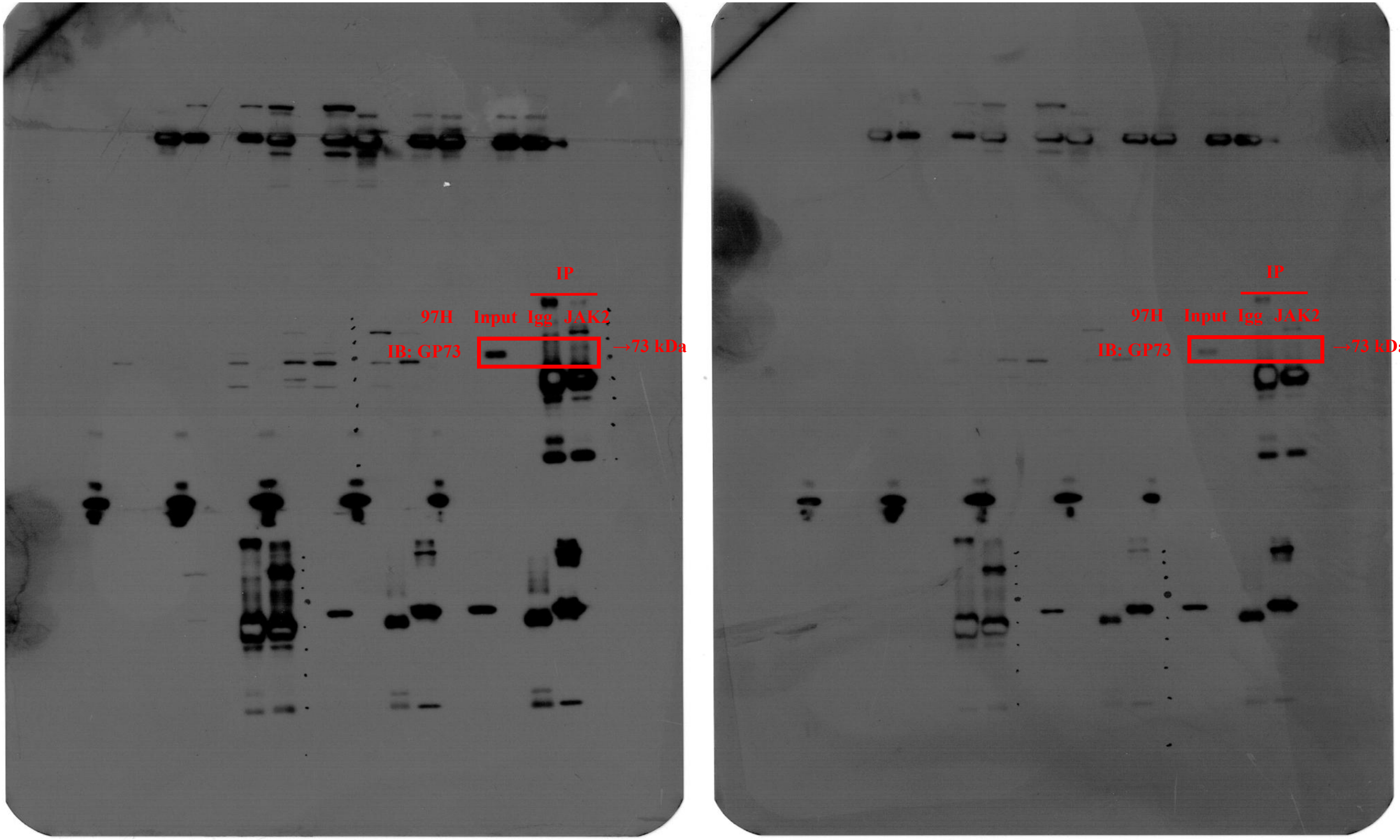
IP
97H
Input
Igg
JAK2
IB: GP73
→73 kDa
IP
97H
Input
Igg
JAK2
IB: GP73
→73 kDa

Figure 5A

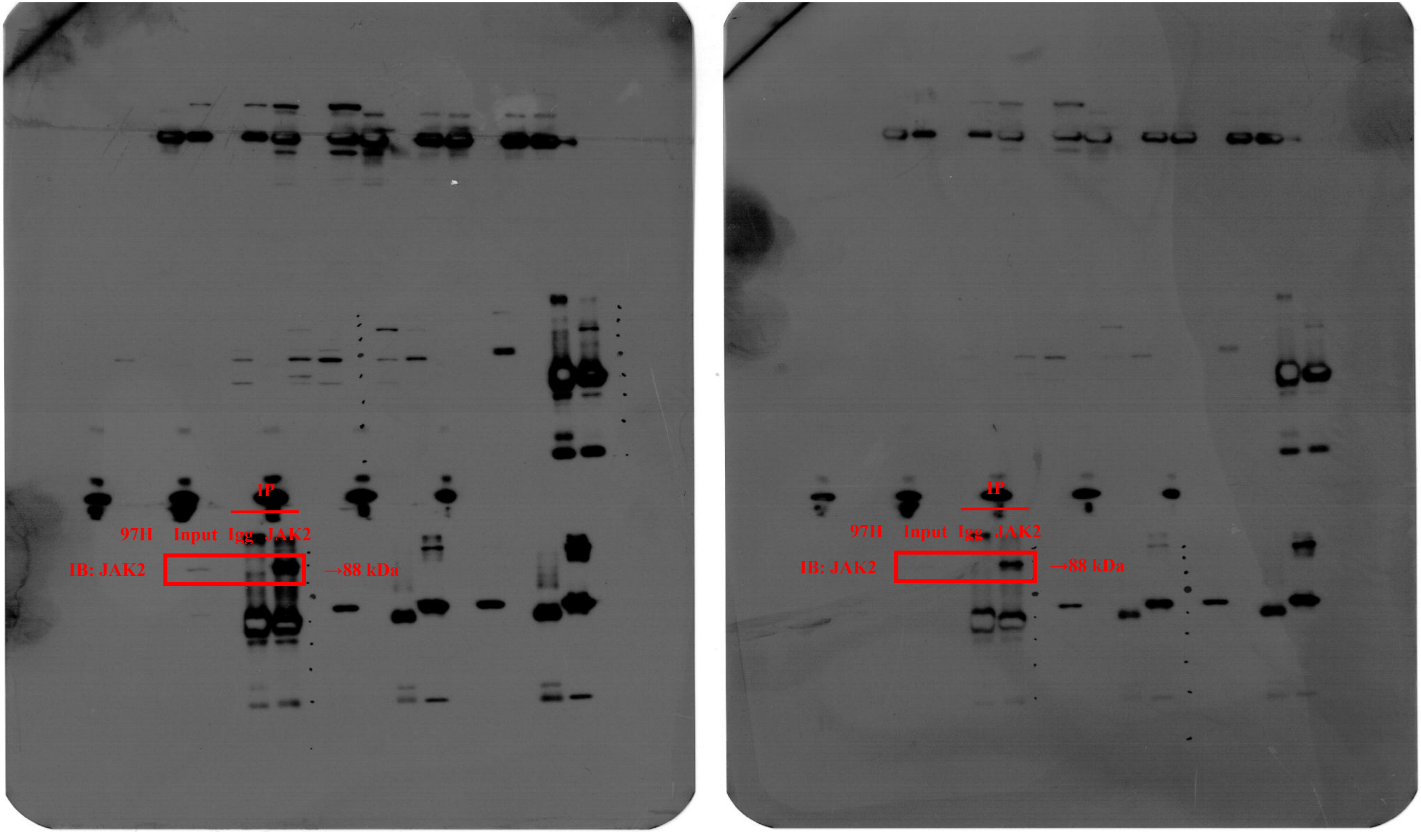
IP
97H
Input
Igg
JAK2
IB: JAK2
→88 kDa
IP
97H
Input
Igg
JAK2
IB: JAK2
→88 kDa

Figure 5B

Western blot detection of the expressions of STAT3 and the phosphorylated-STAT3 proteins STAT3-Tyr705 and STAT3-Ser727 in MHCC-97H-GP73-KD, MHCC-97H-GP73-NC, Hep3B-GP73-OE and Hep3B-GP73-NC cells. Data were representative of three similar observations or were shown as the mean±SD of three experiments.

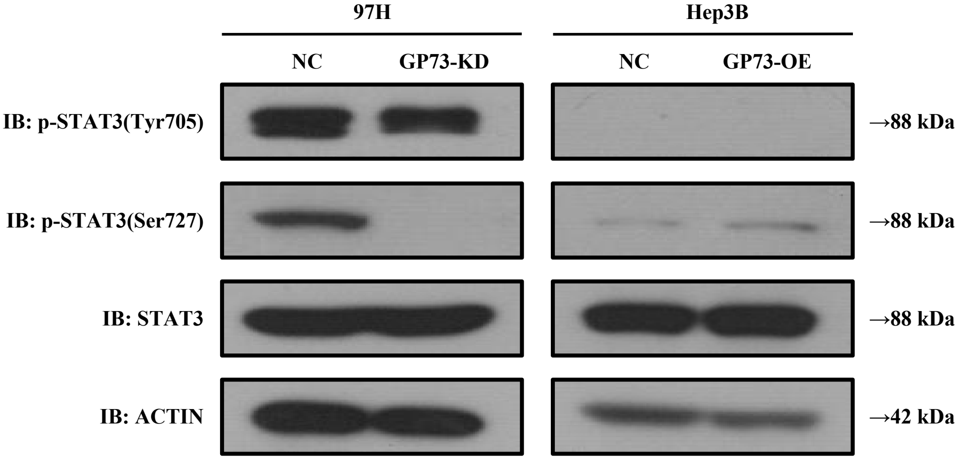

Figure 5B

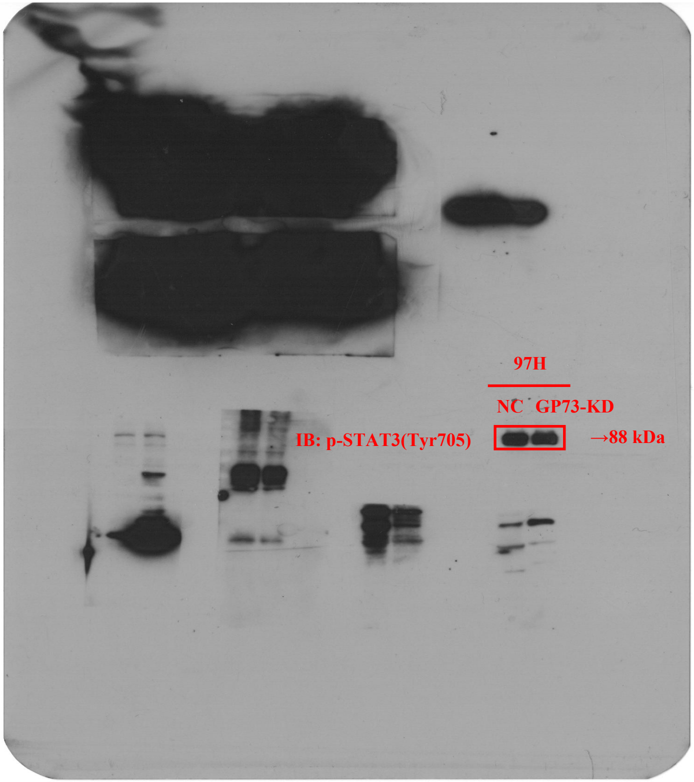
97H
NC GP73-KD
IB: p-STAT3(Tyr705)
→88 kDa

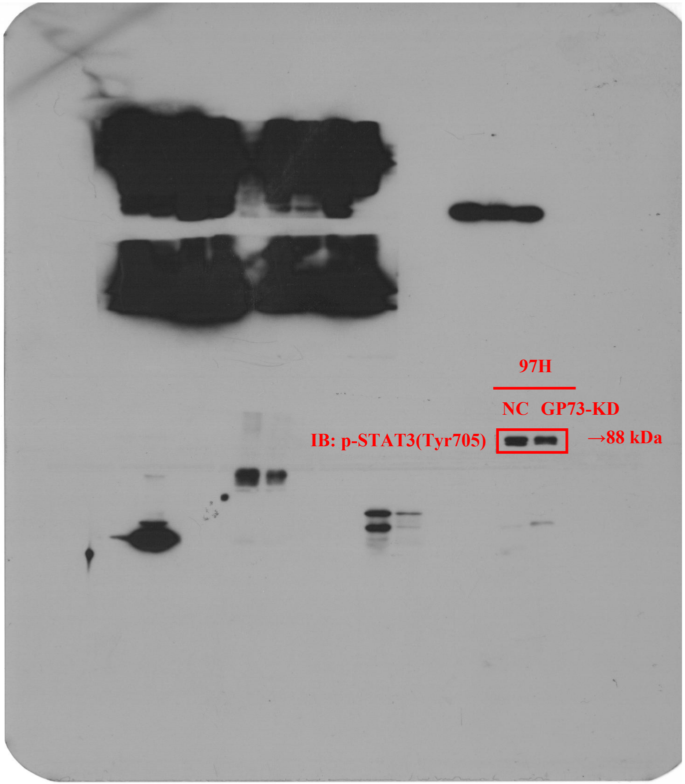
97H
NC GP73-KD
IB: p-STAT3(Tyr705)
→88 kDa

Figure 5B

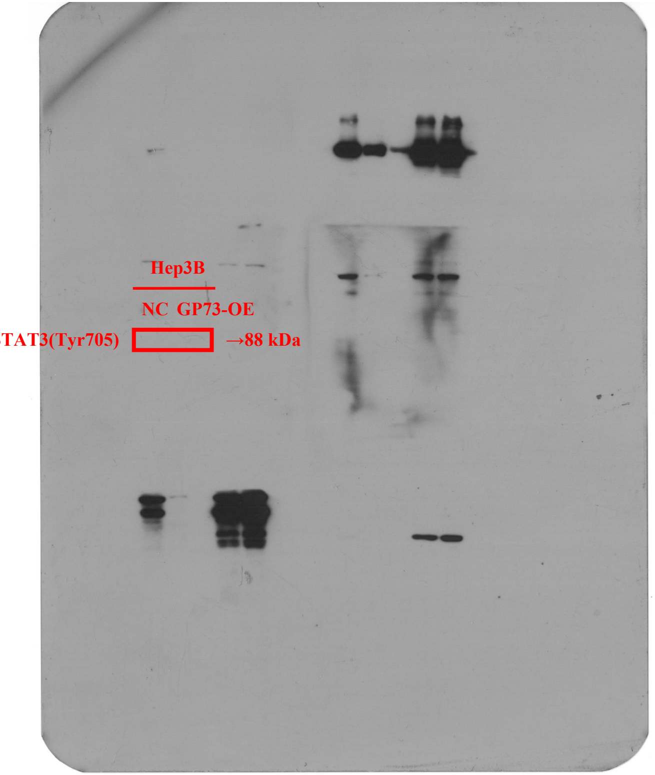
Hep3B
NC GP73-OE
TAT3(Tyr705)
→88 kDa

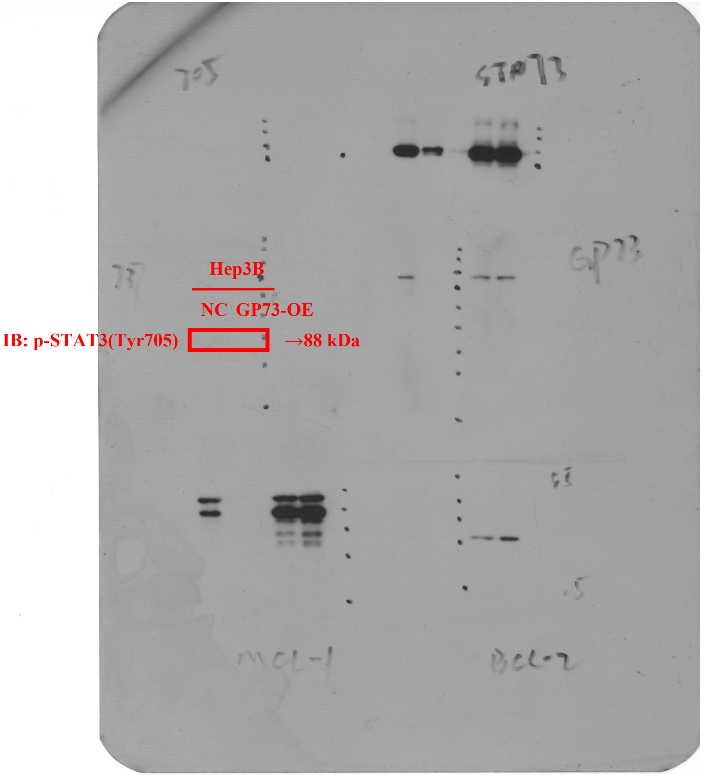
Hep3B
NC GP73-OE
IB: p-STAT3(Tyr705)
→88 kDa

Figure 5B

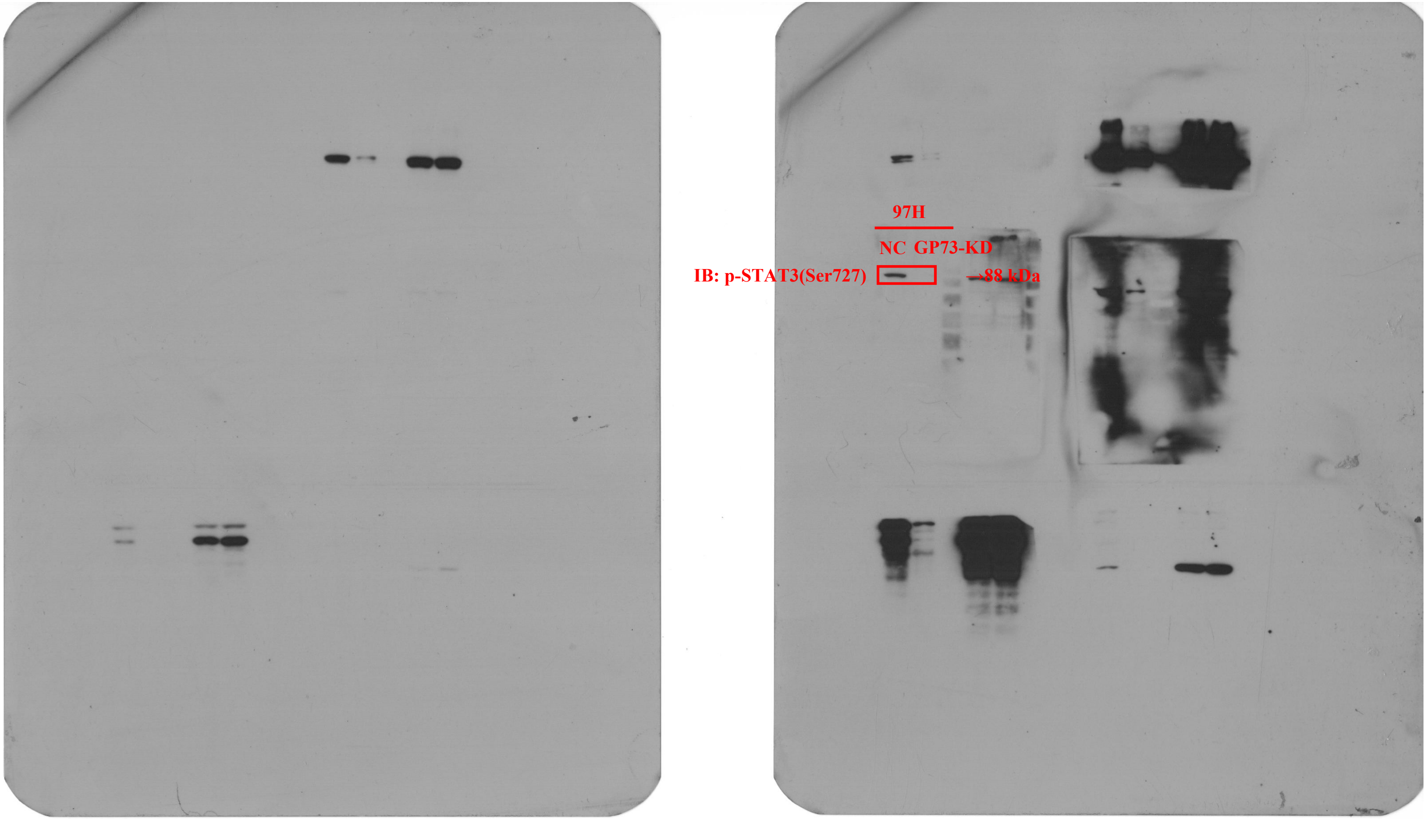

Figure 5B

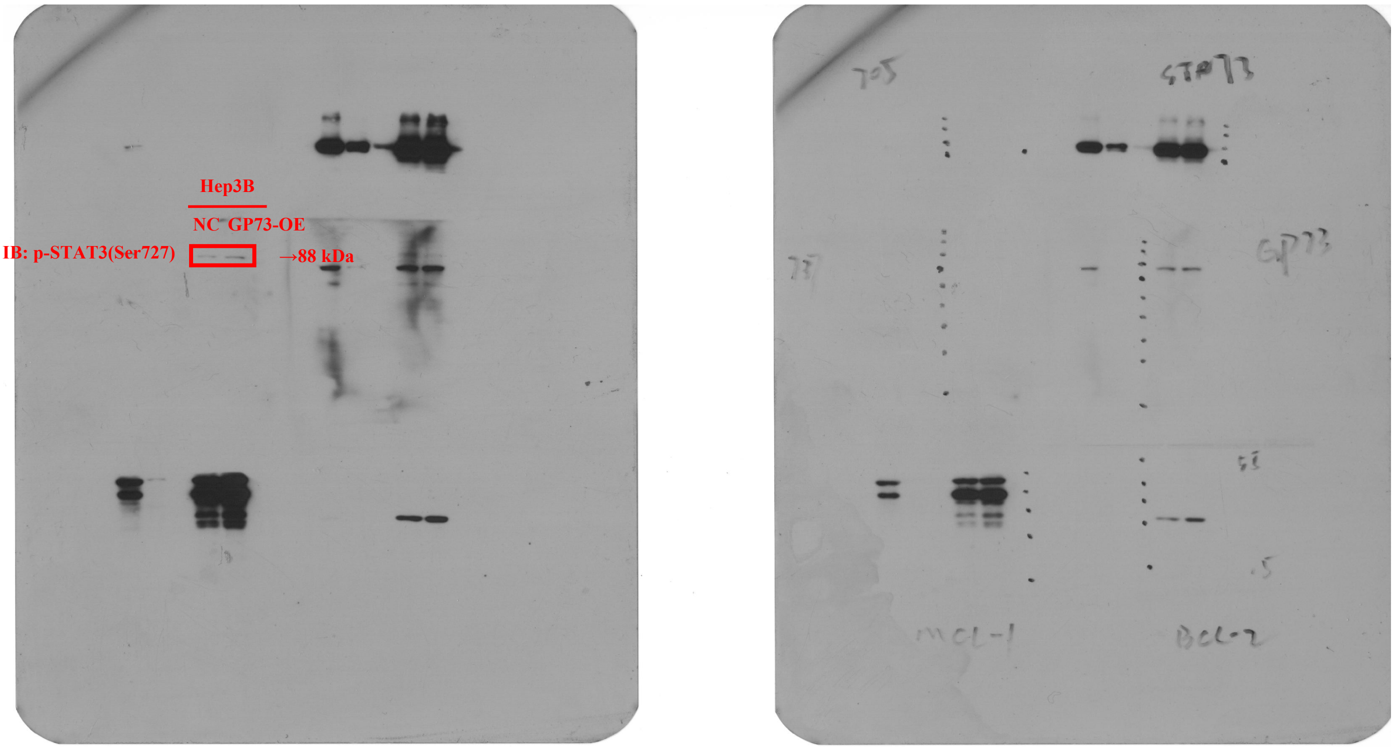

Figure 5B

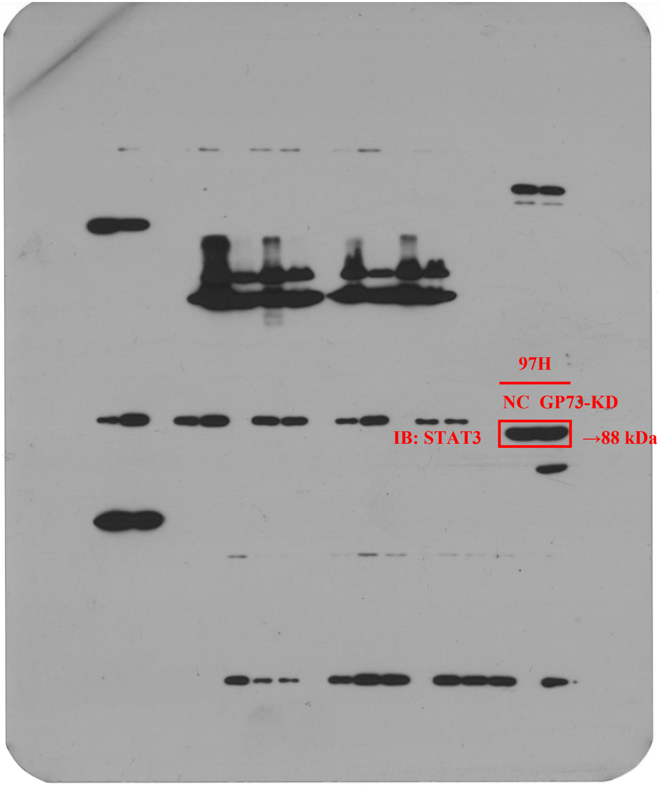

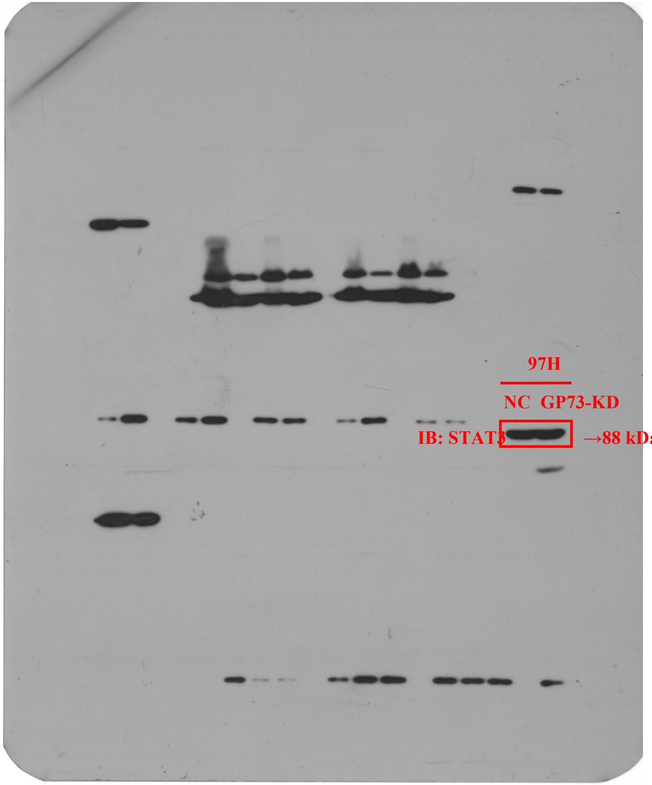

Figure 5B

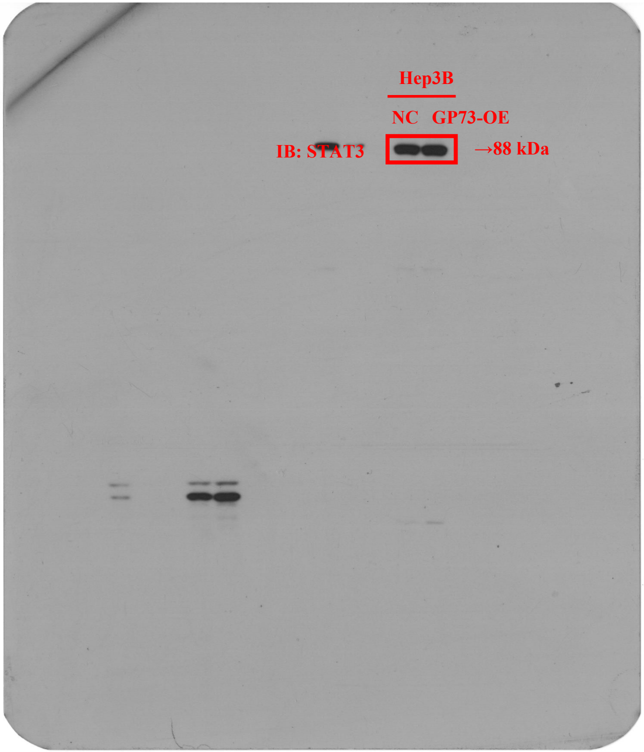
Hep3B
NC GP73-OE
IB: STAT3
→88 kDa

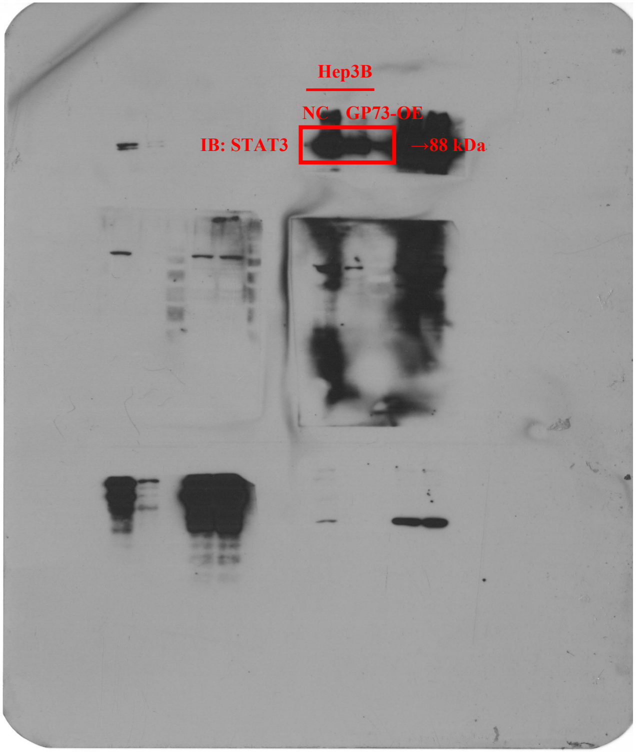
Hep3B
NC GP73-OE
IB: STAT3
→88 kDa

Figure 5B

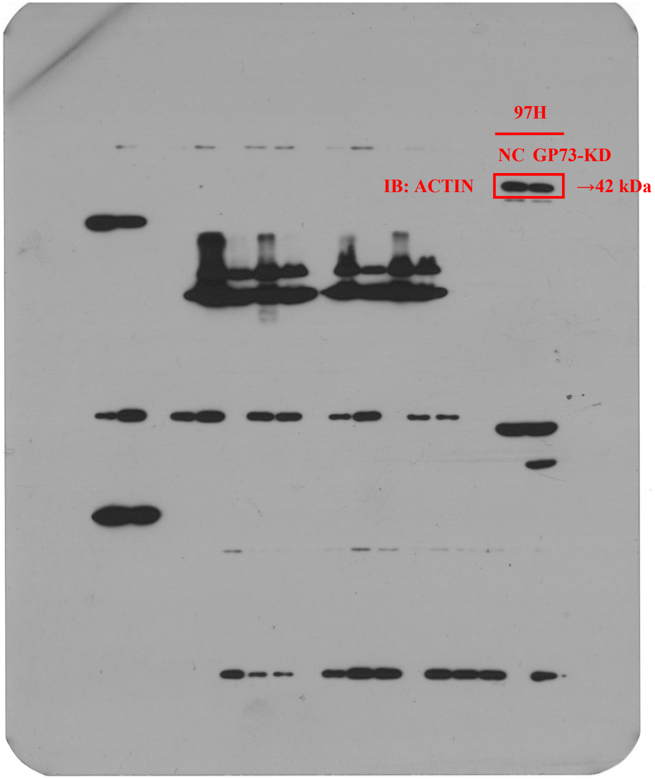

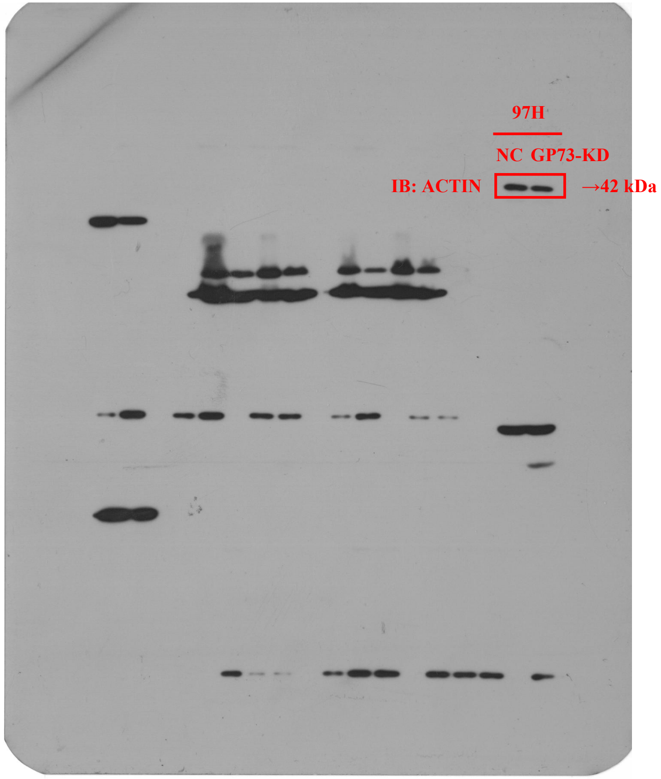

Figure 5B

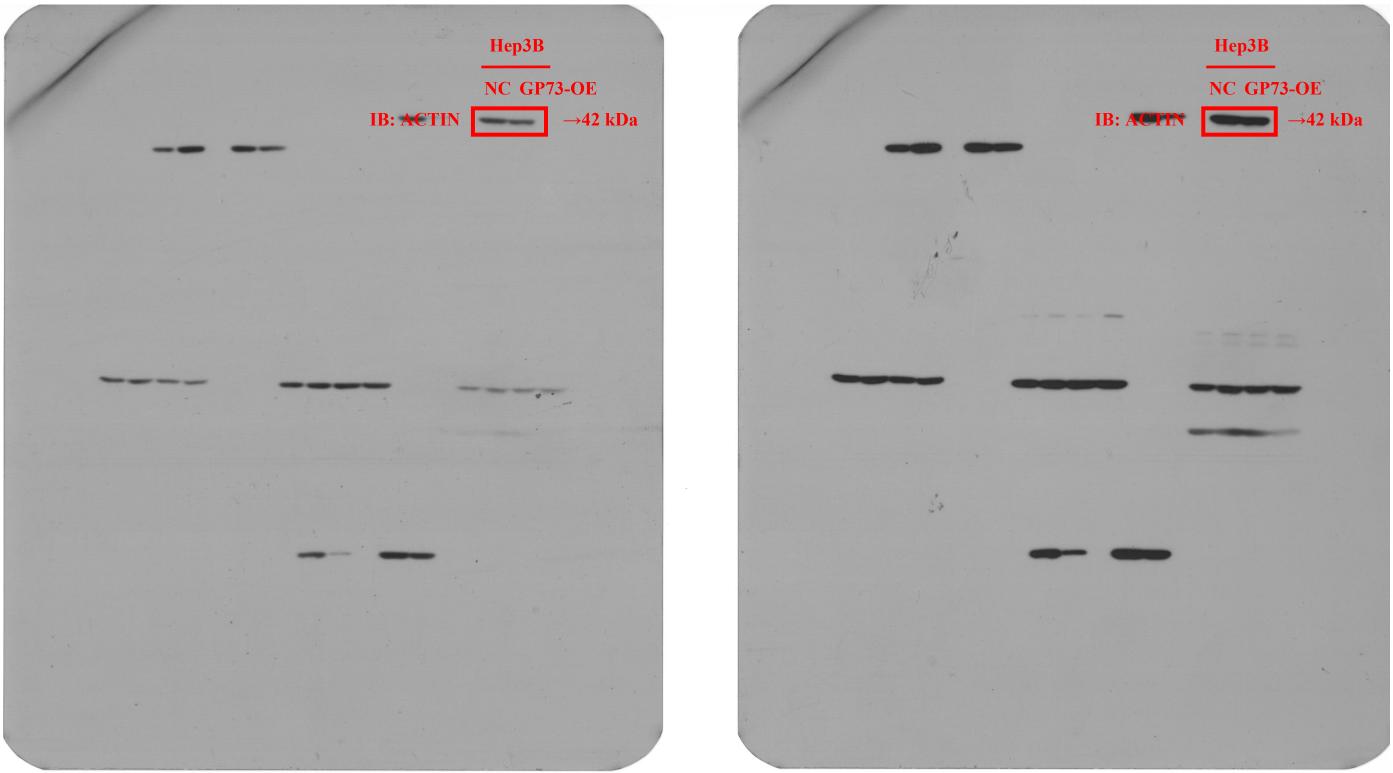
Hep3B
NC GP73-OE
IB: ACTIN
→42 kDa
Hep3B
NC GP73-OE
IB: ACTIN
→42 kDa

Figure 5G

Co-IP assays detected the interaction between GP73 and STAT3.

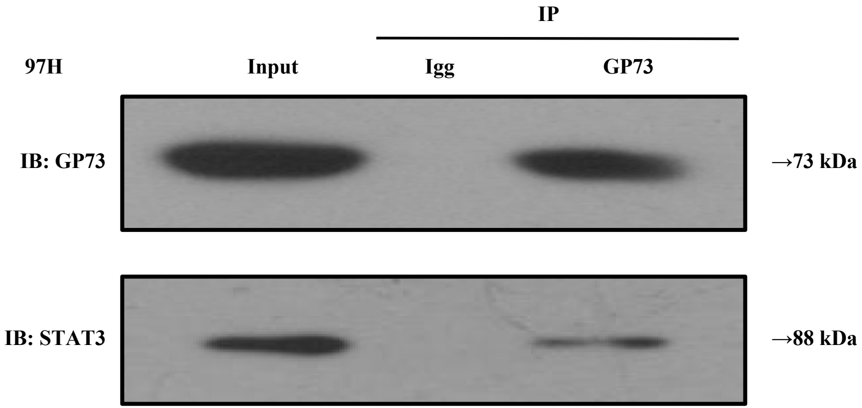

Figure 5G

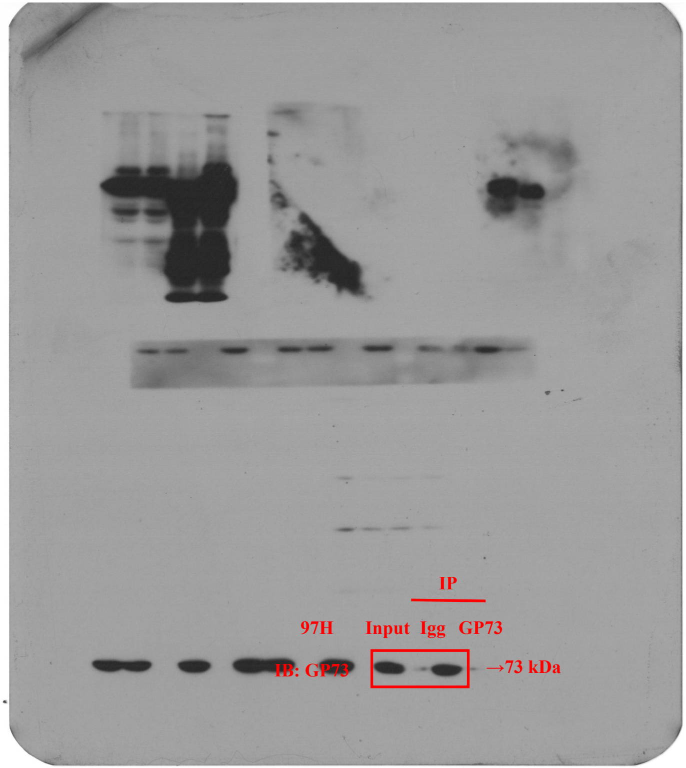
IP
97H
Input
Igg
GP73
IB: GP73
→73 kDa

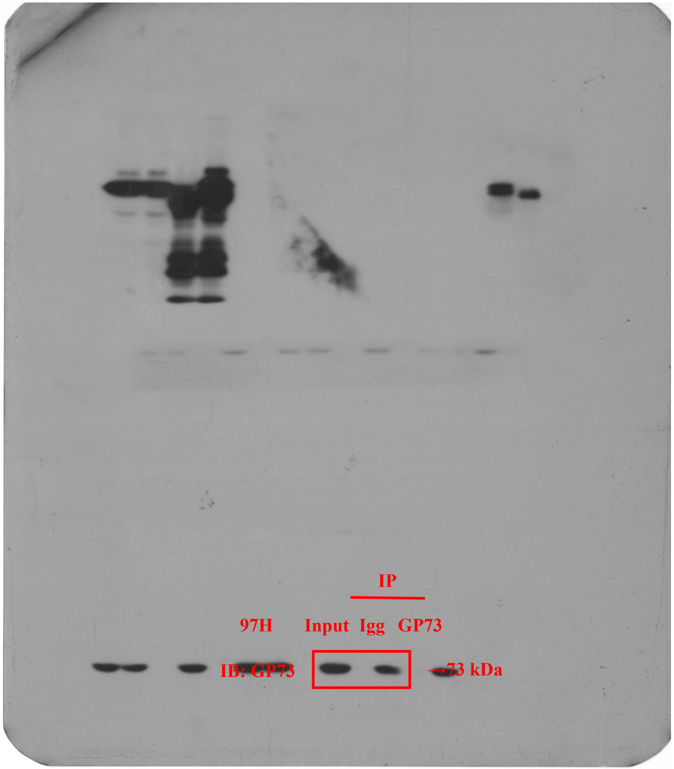
IP
97H
Input
Igg
GP73
IB: GP73
→73 kDa

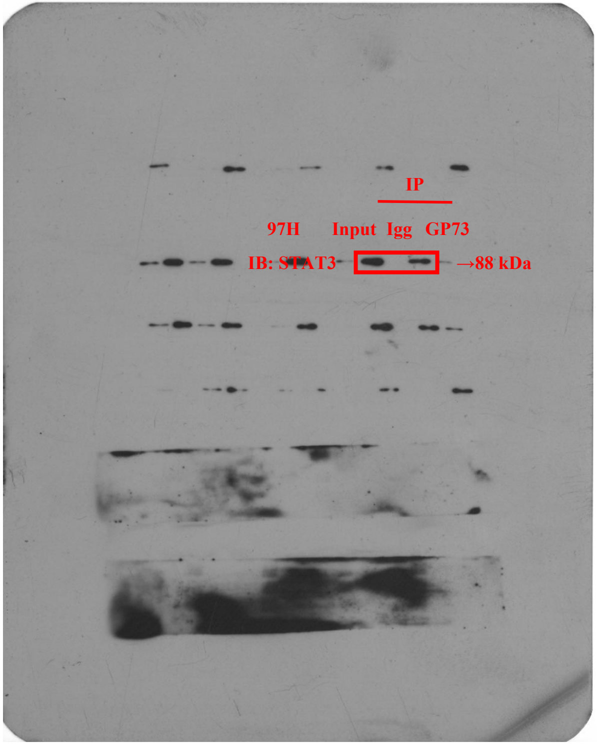
IP
97H
Input
Igg
GP73
IB: STAT3
→88 kDa

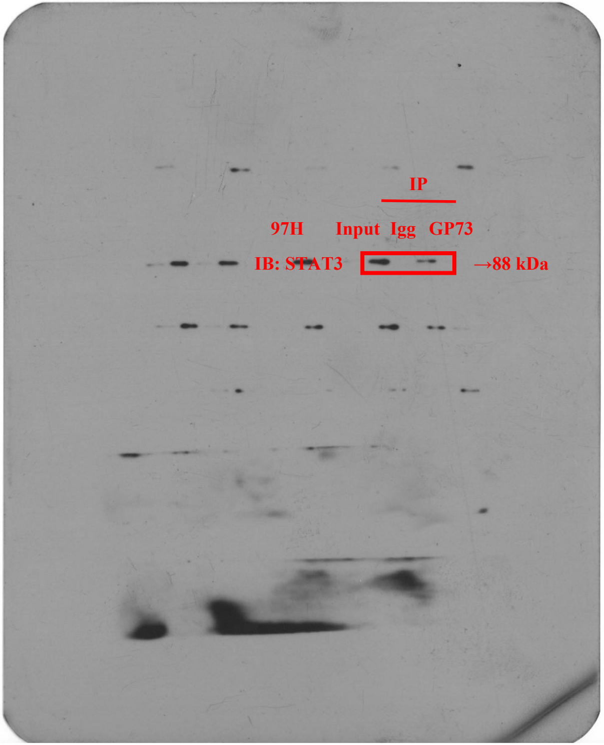
IP
97H
Input
Igg
GP73
IB: STAT3
→88 kDa

Figure 5K

Western blot detection of key genes expressions in the JAK2/STAT3 and ERS pathway in MHCC97H-GP73-KD, MHCC97H-GP73-NC, Hep3B-GP73-OE and Hep3B-GP73-NC cells.

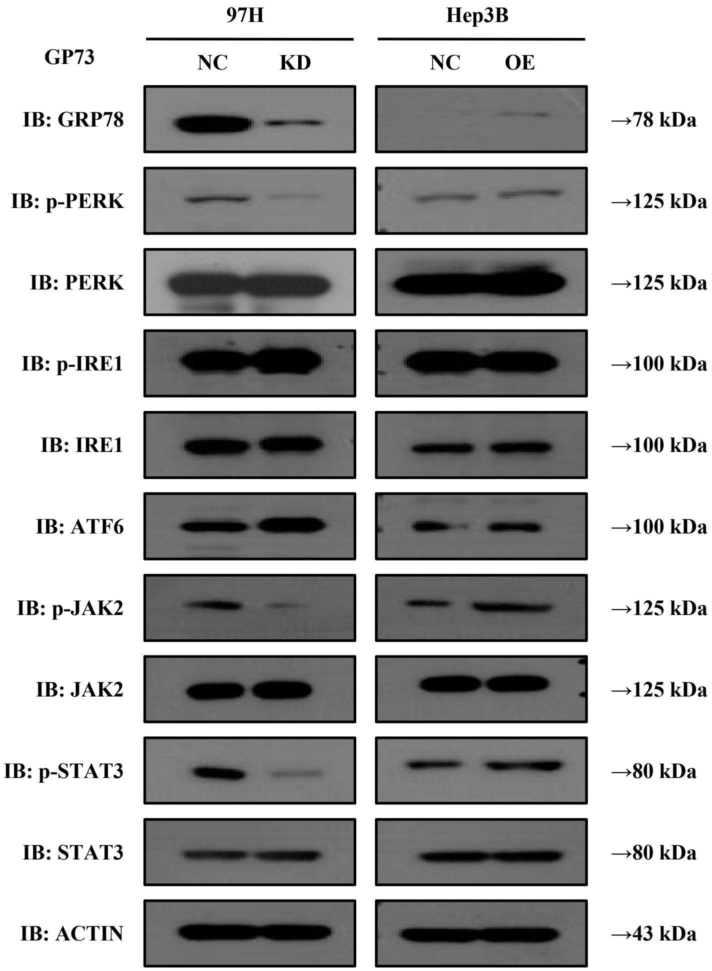

Figure 5K

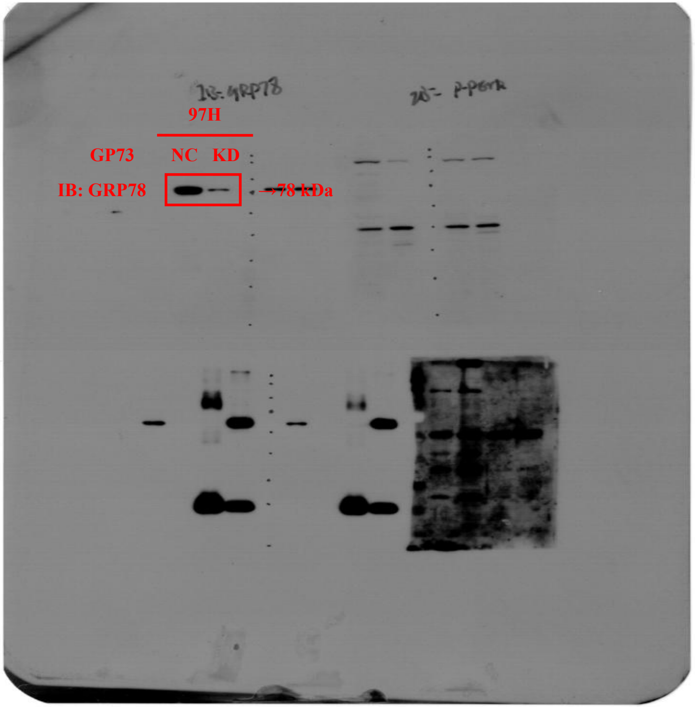
IB: GRP78
97H
GP73
NC
KD
IB: GRP78
→78 kDa

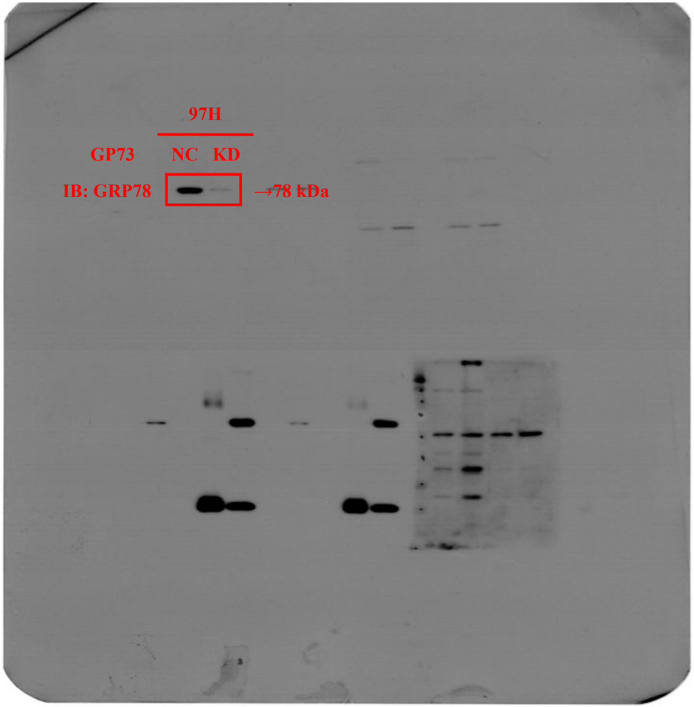
97H
GP73
NC
KD
IB: GRP78
→78 kDa

Figure 5K

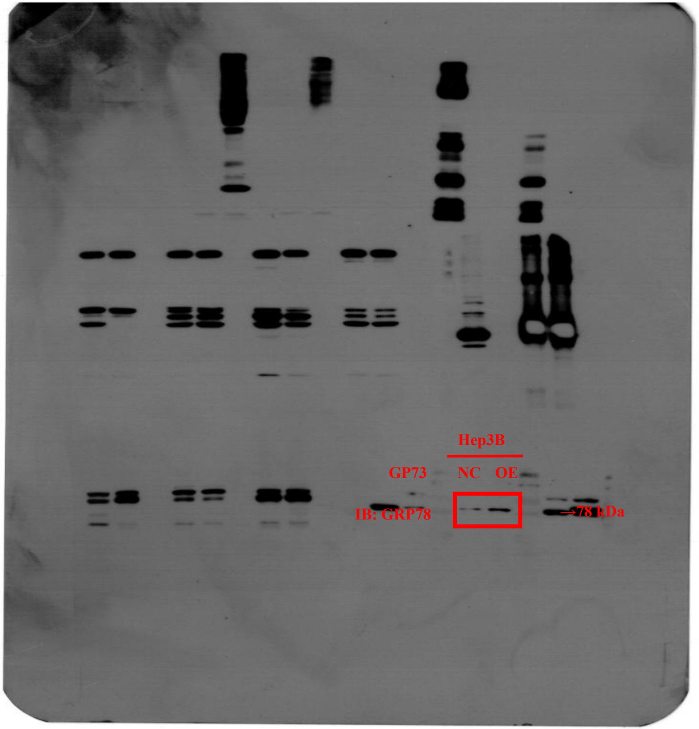

Hep3B
GP73
NC
OE
IB: GRP78
→78 kDa

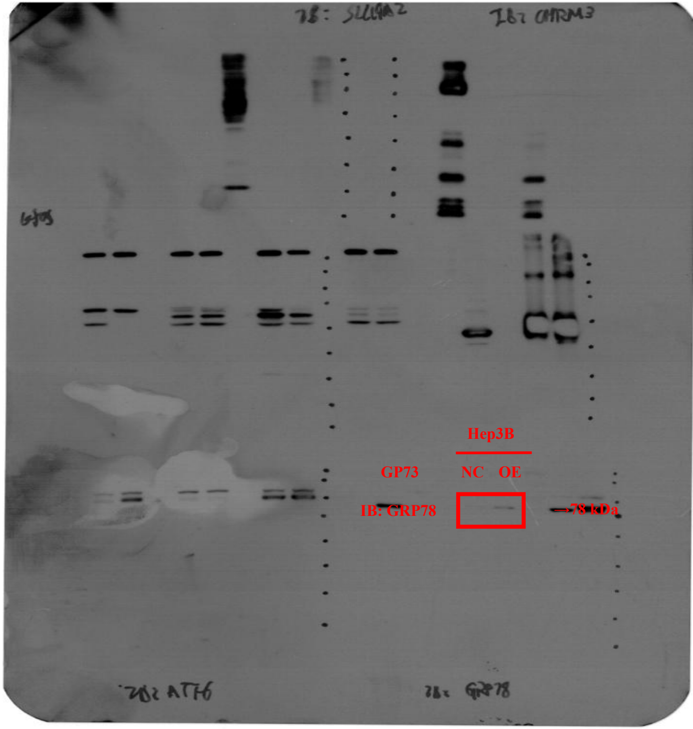

Hep3B
GP73
NC
OE
IB: GRP78
→78 kDa

Figure 5K

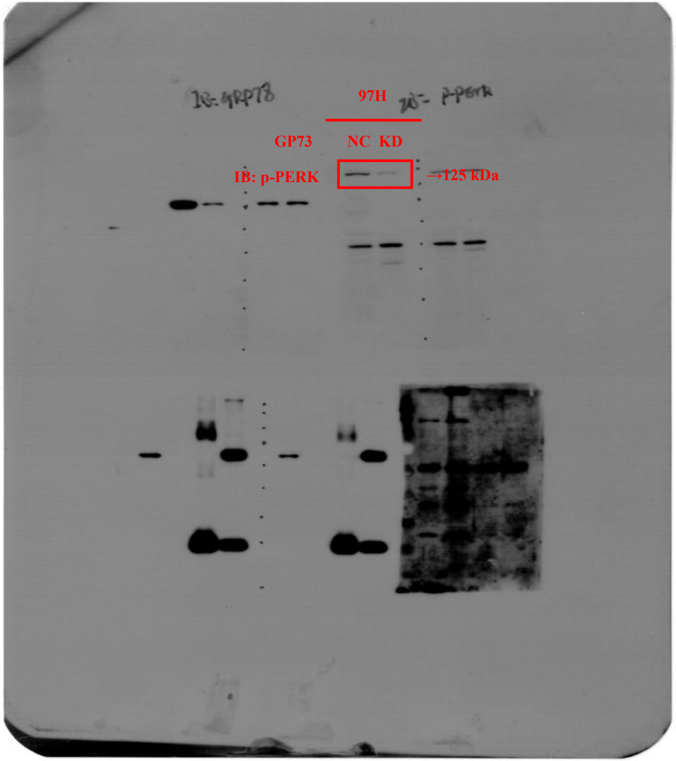

IB: GRP78
97H
IB: p-PERK
GP73
NC KD
IB: p-PERK
→125 kDa

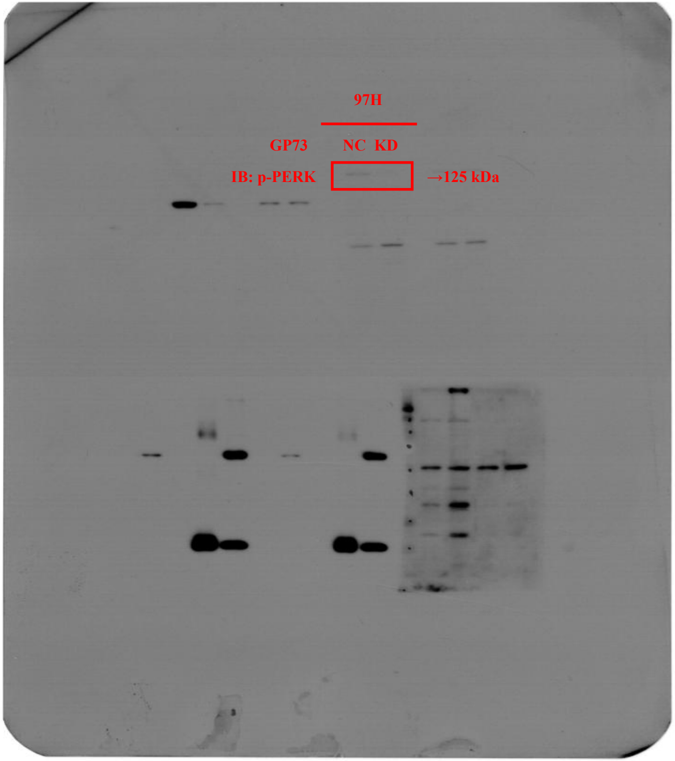

97H
GP73
NC KD
IB: p-PERK
→125 kDa

Figure 5K

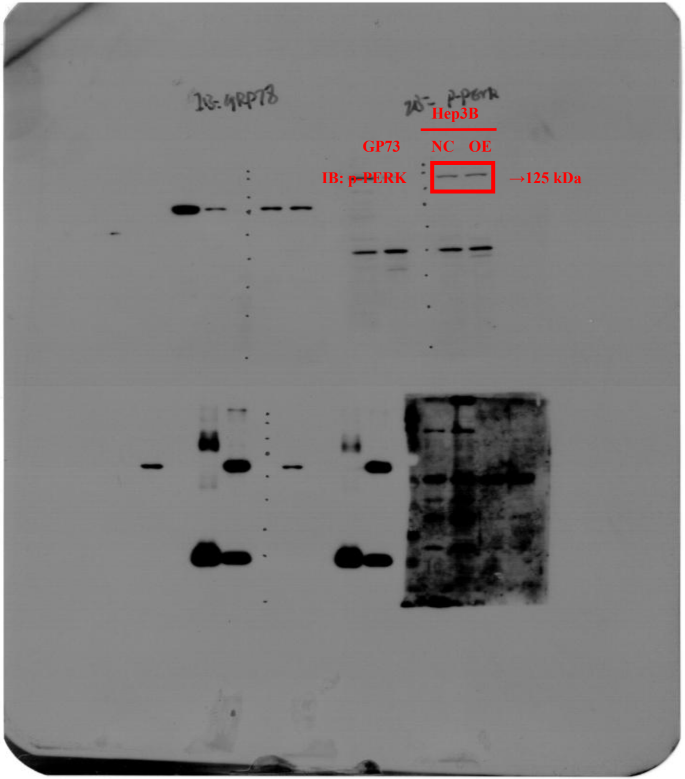

IB: GRP78
IB: p-PERK
Hep3B
GP73 NC OE
IB: p-PERK
→125 kDa

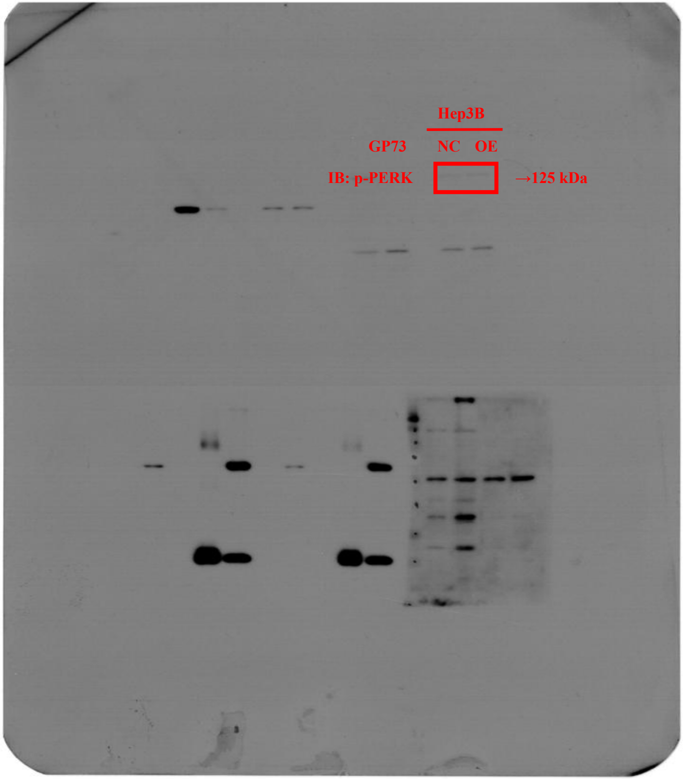

Hep3B
GP73 NC OE
IB: p-PERK
→125 kDa

Figure 5K

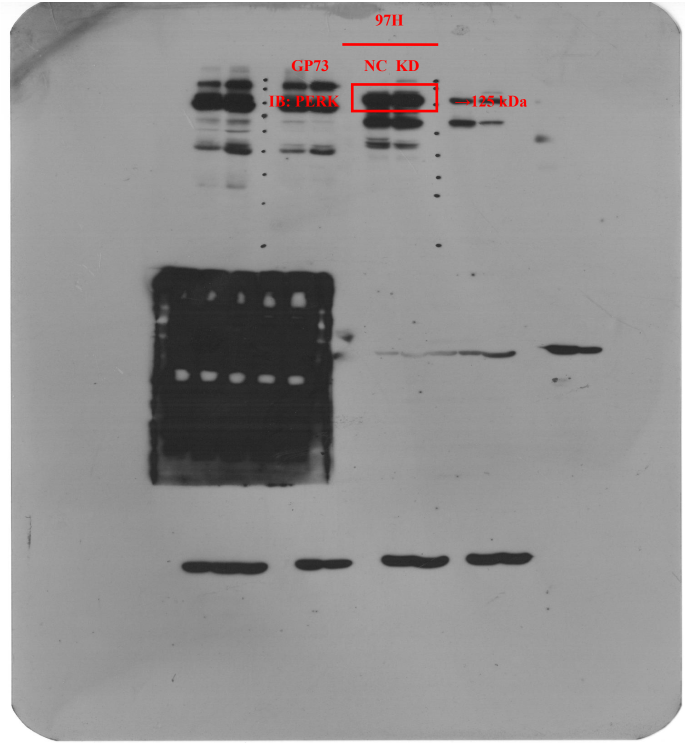

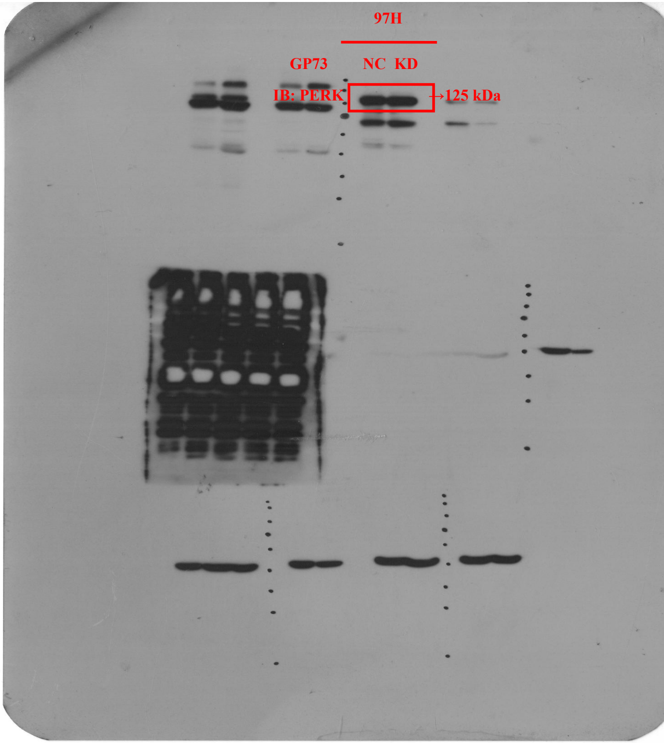

Figure 5K

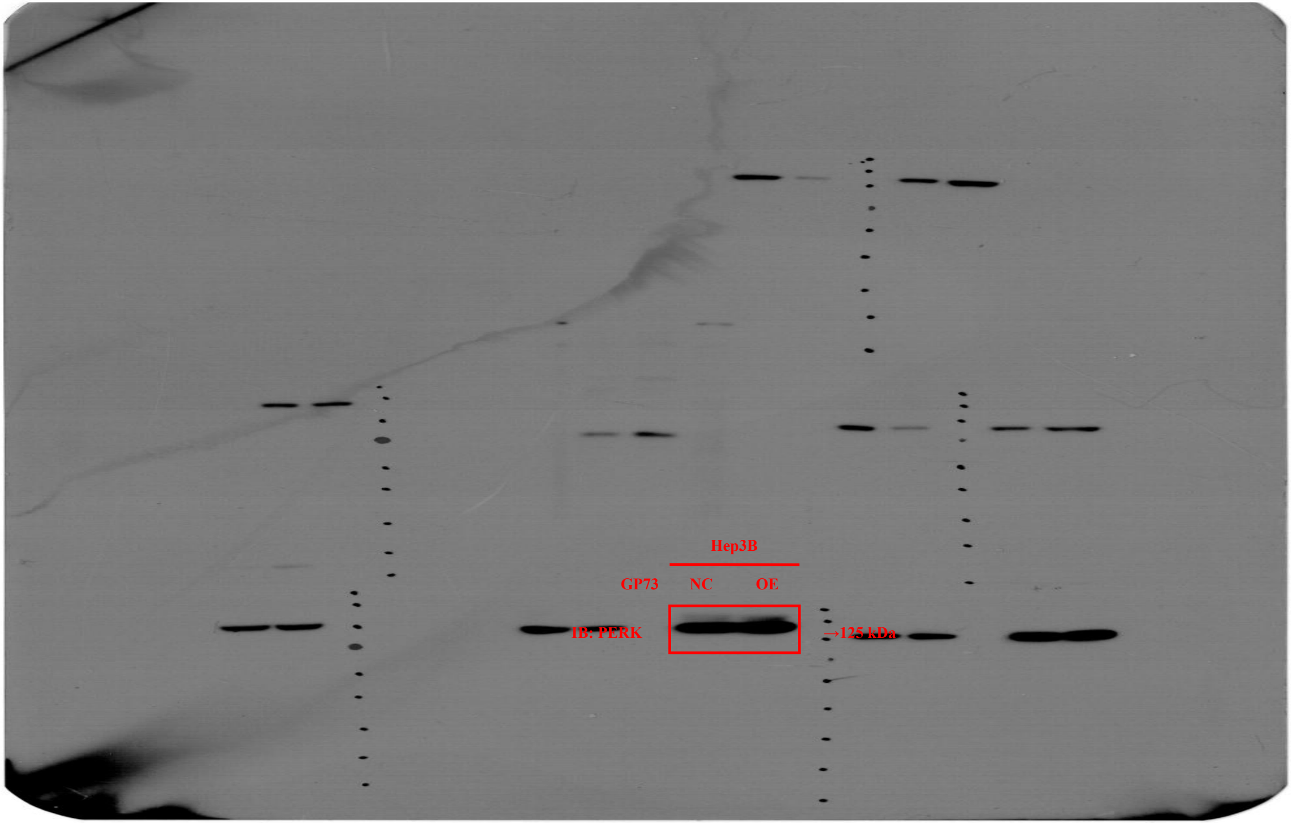

Figure 5K

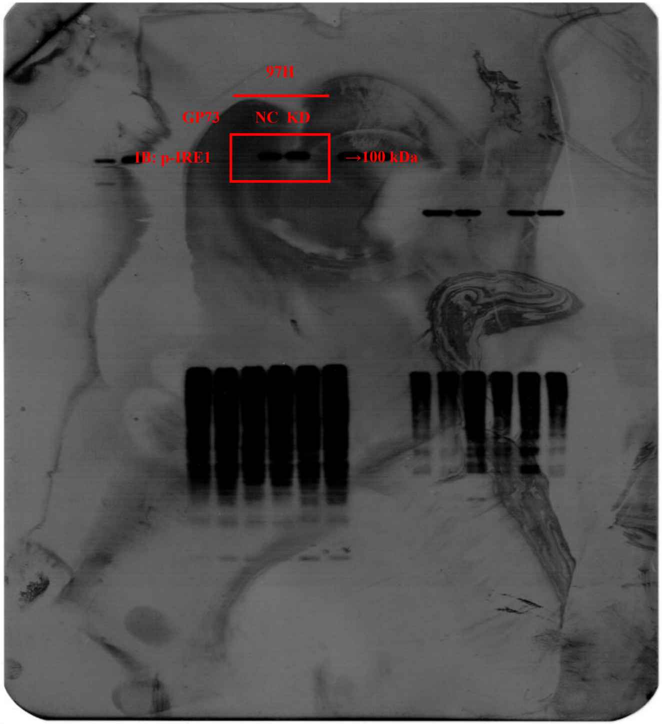

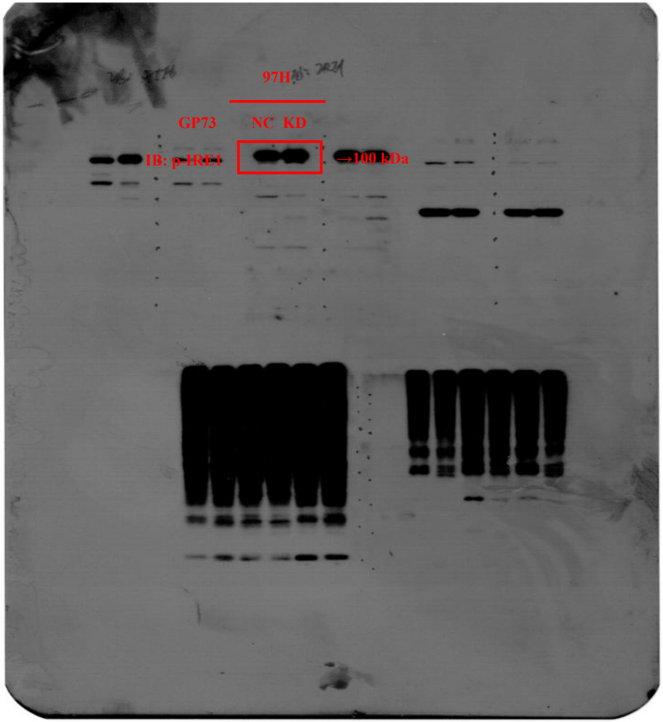

Figure 5K

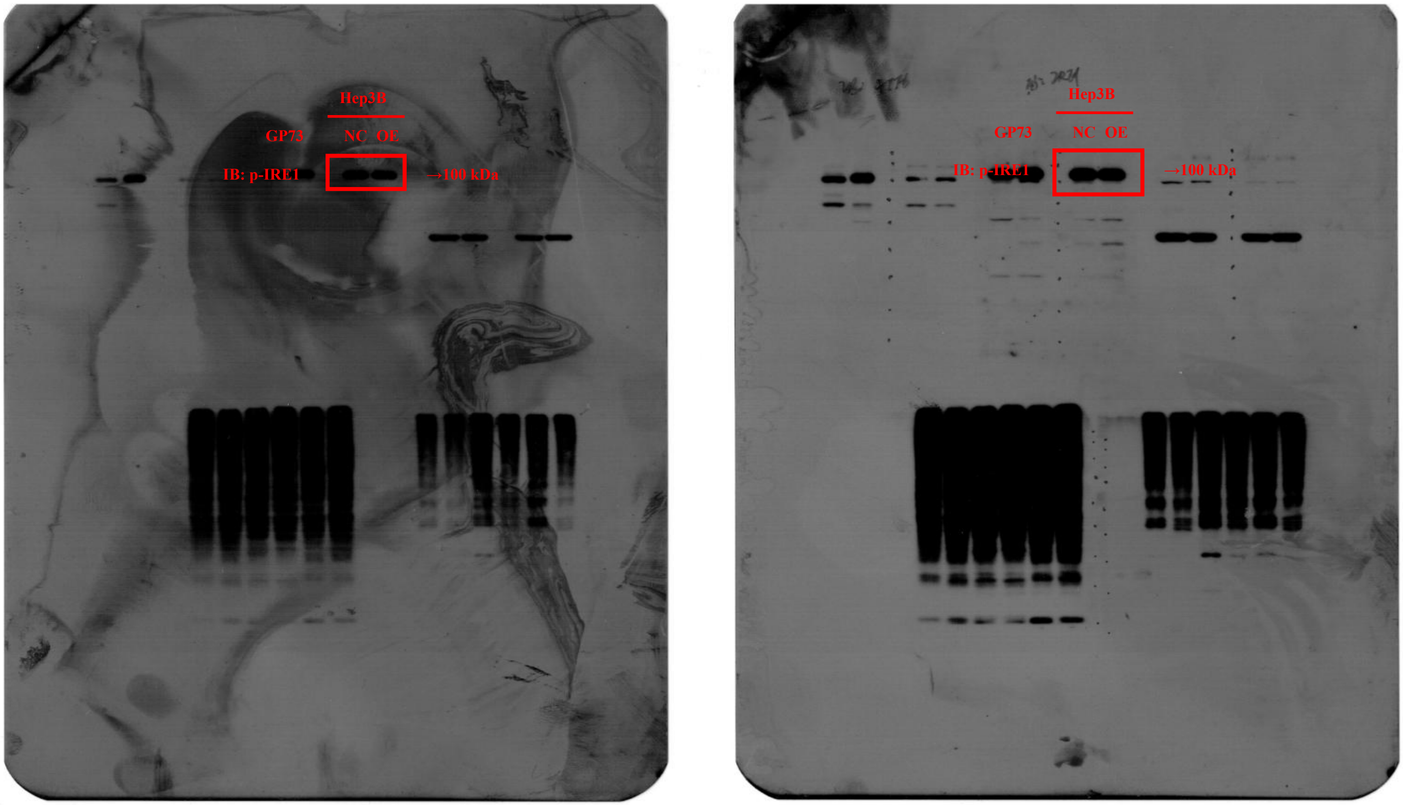

Hep3B
GP73
NC OE
IB: p-IRE1
→100 kDa
Hep3B
GP73
NC OE
IB: p-IRE1
→100 kDa

Figure 5K

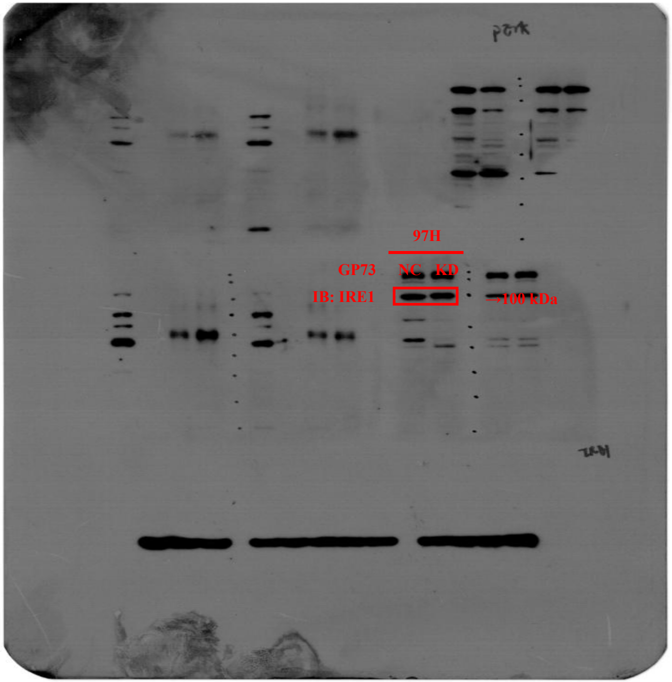

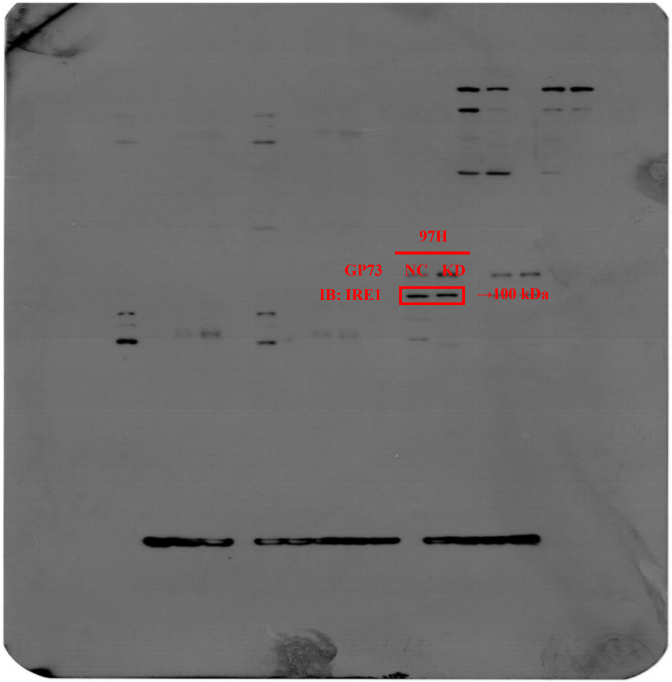

Figure 5K

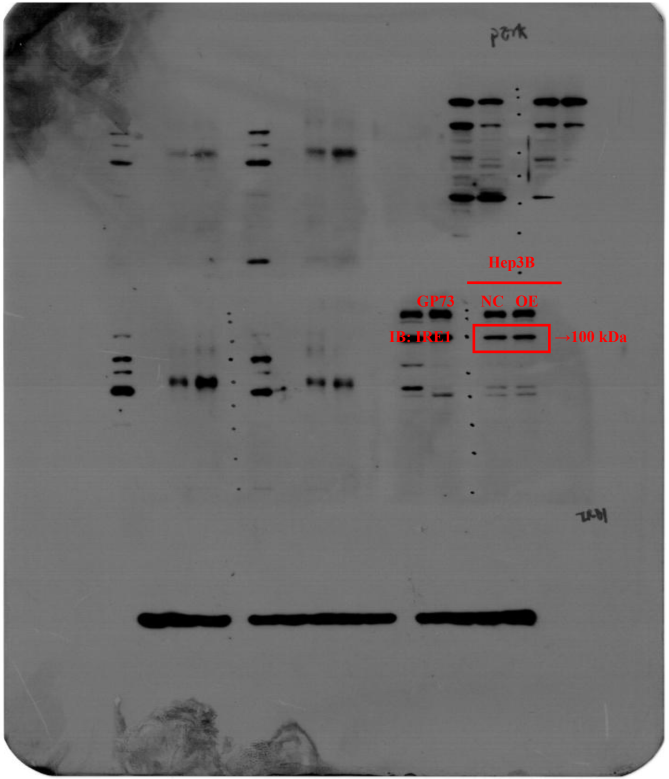

Hep3B
GP73
NC
OE
IB: IRE1
→100 kDa

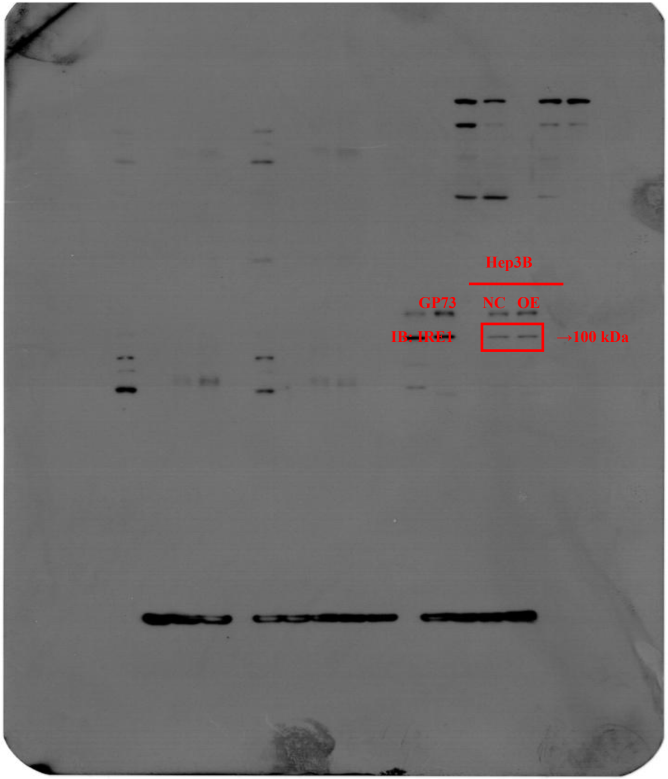

Hep3B
GP73
NC
OE
IB: IRE1
→100 kDa

Figure 5K

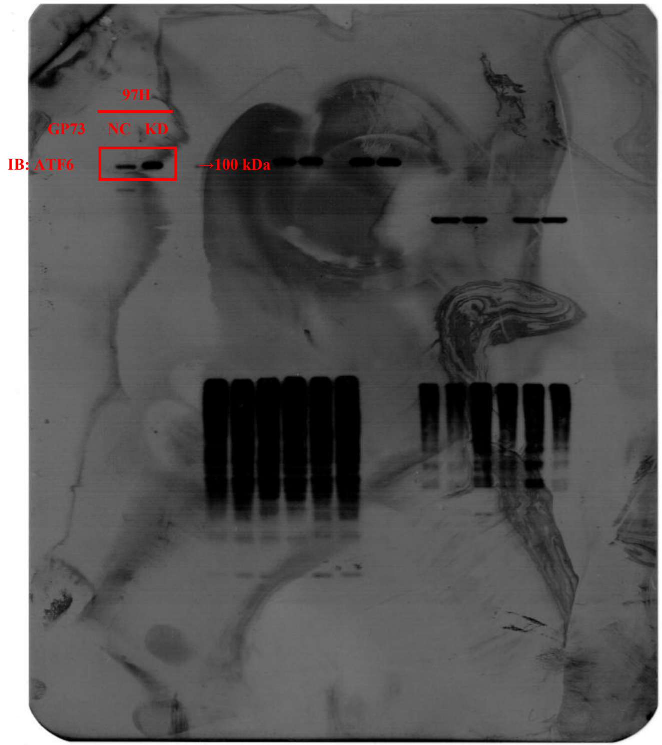

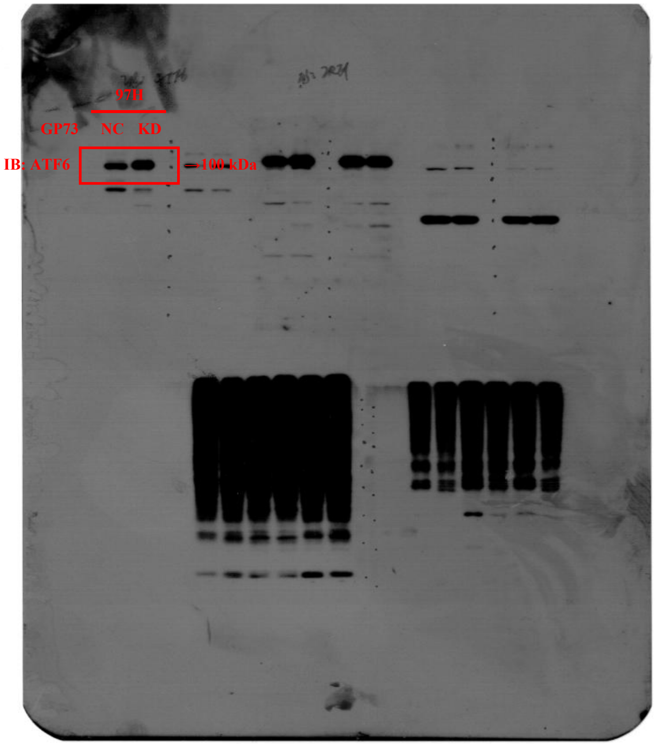

Figure 5K

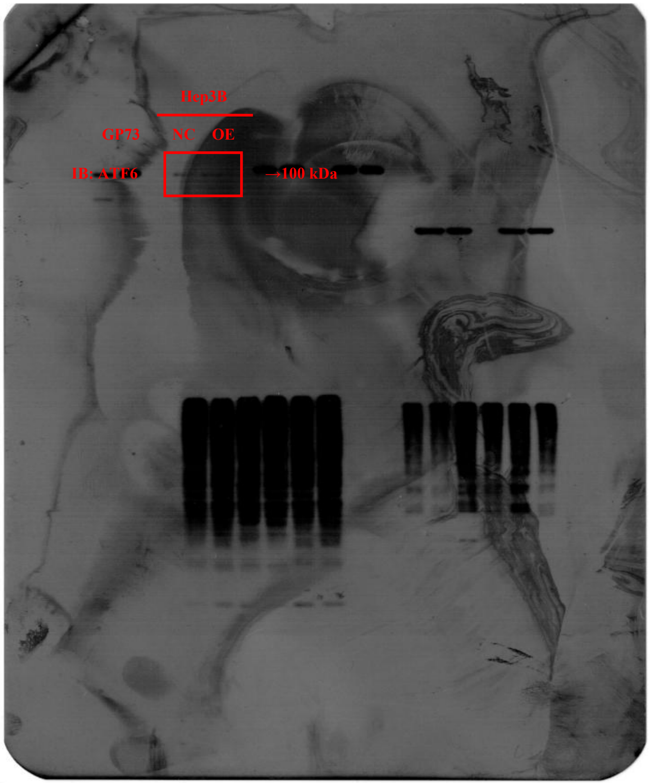
Hep3B
GP73
NC
OE
IB: ATF6
→100 kDa

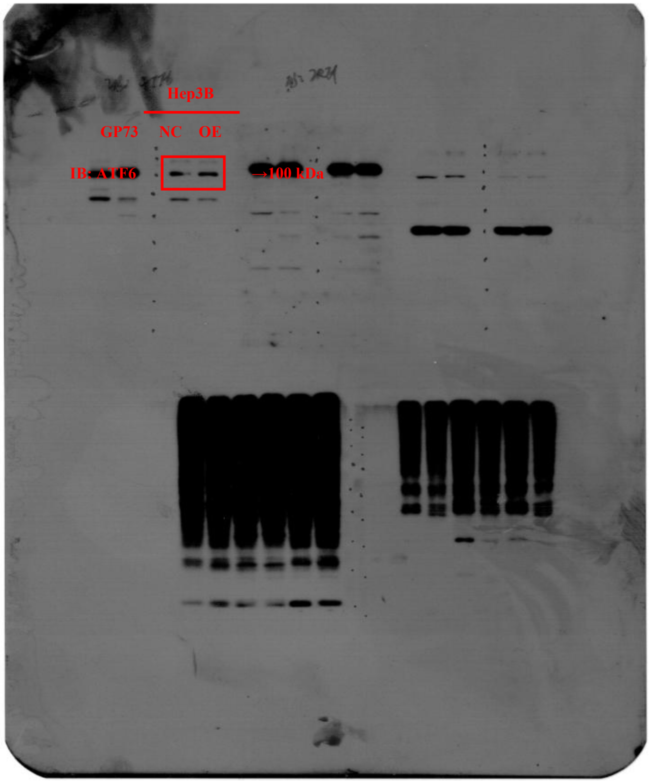
Hep3B
GP73
NC
OE
IB: ATF6
→100 kDa

Figure 5K

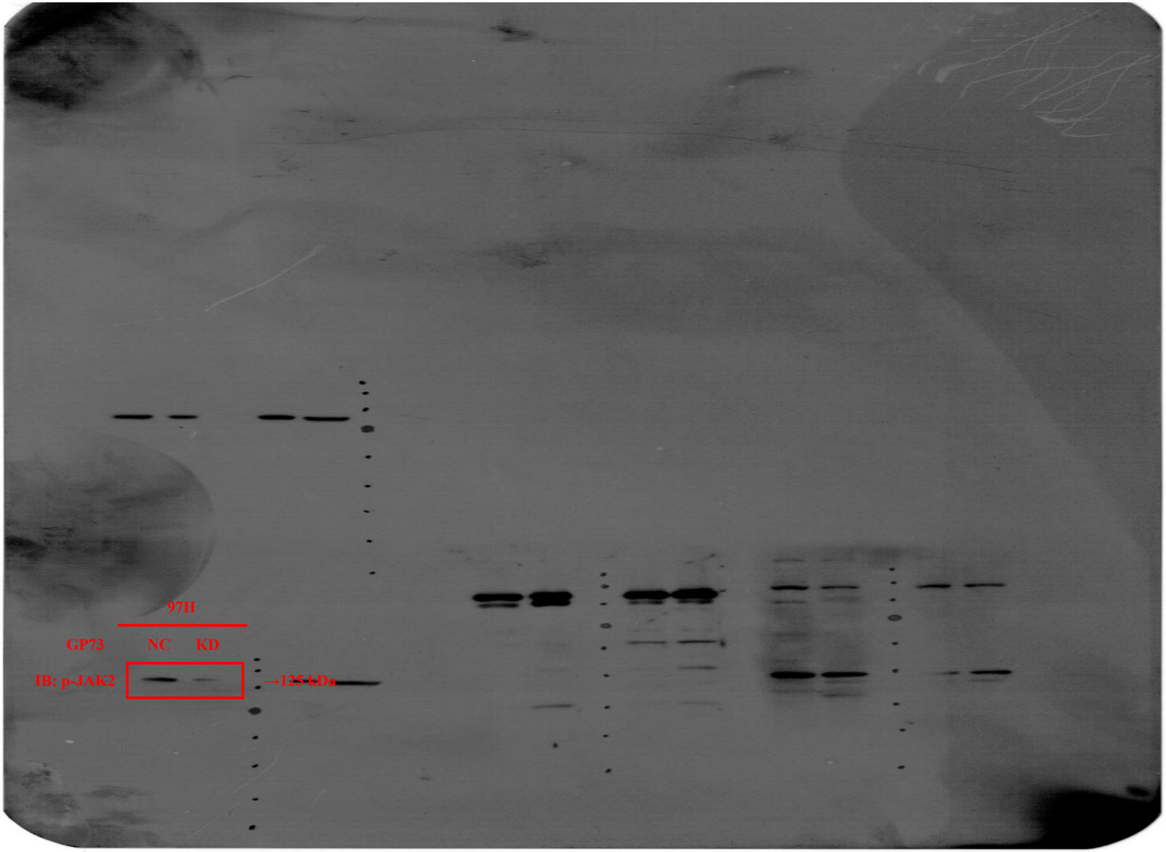

Figure 5K

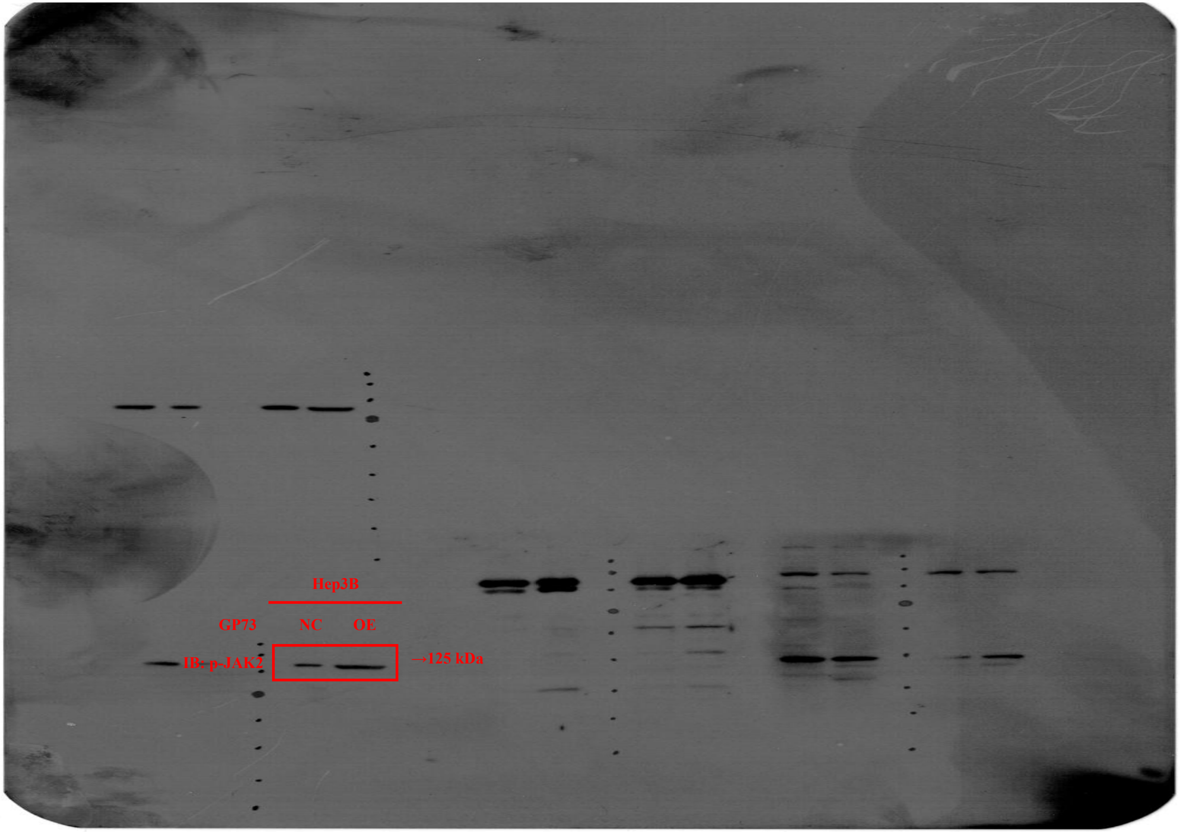

Figure 5K

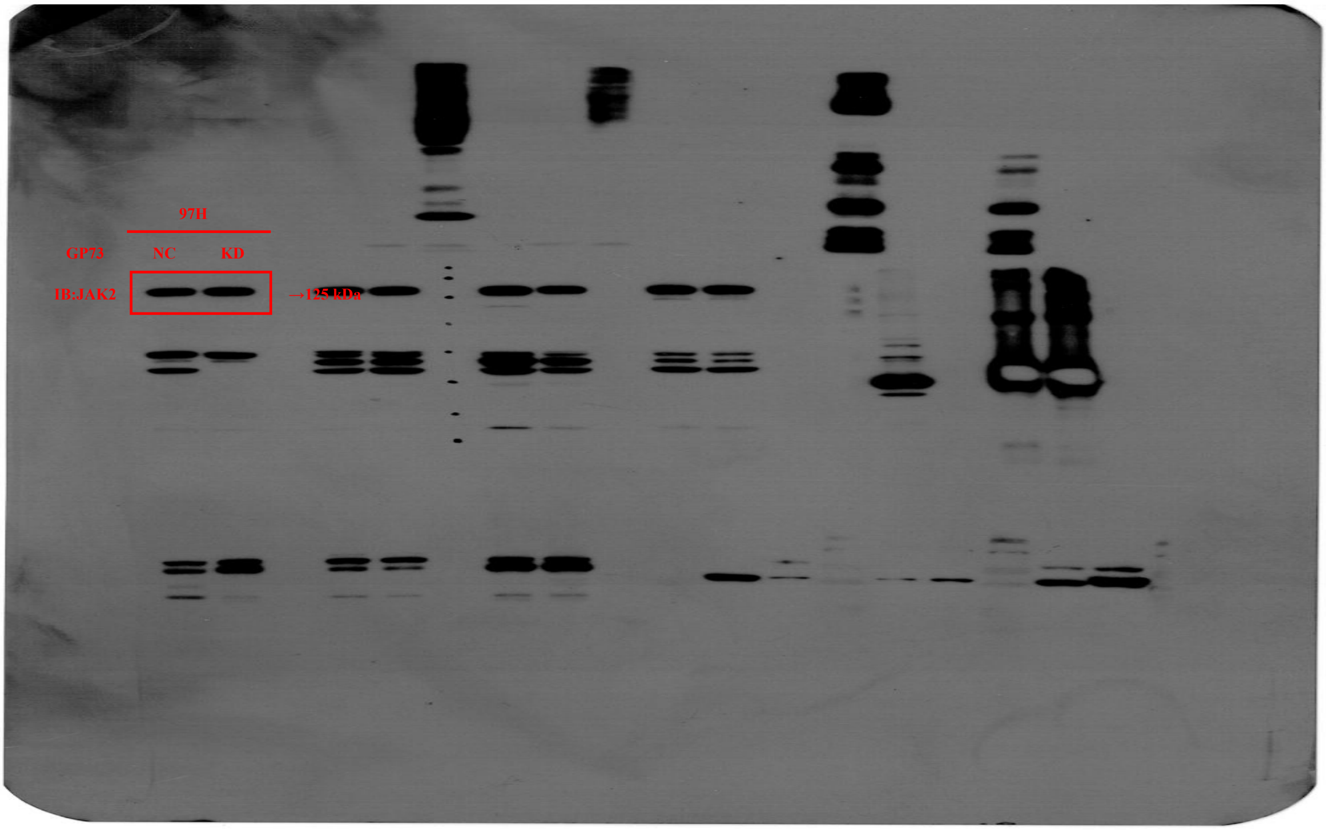

Figure 5K

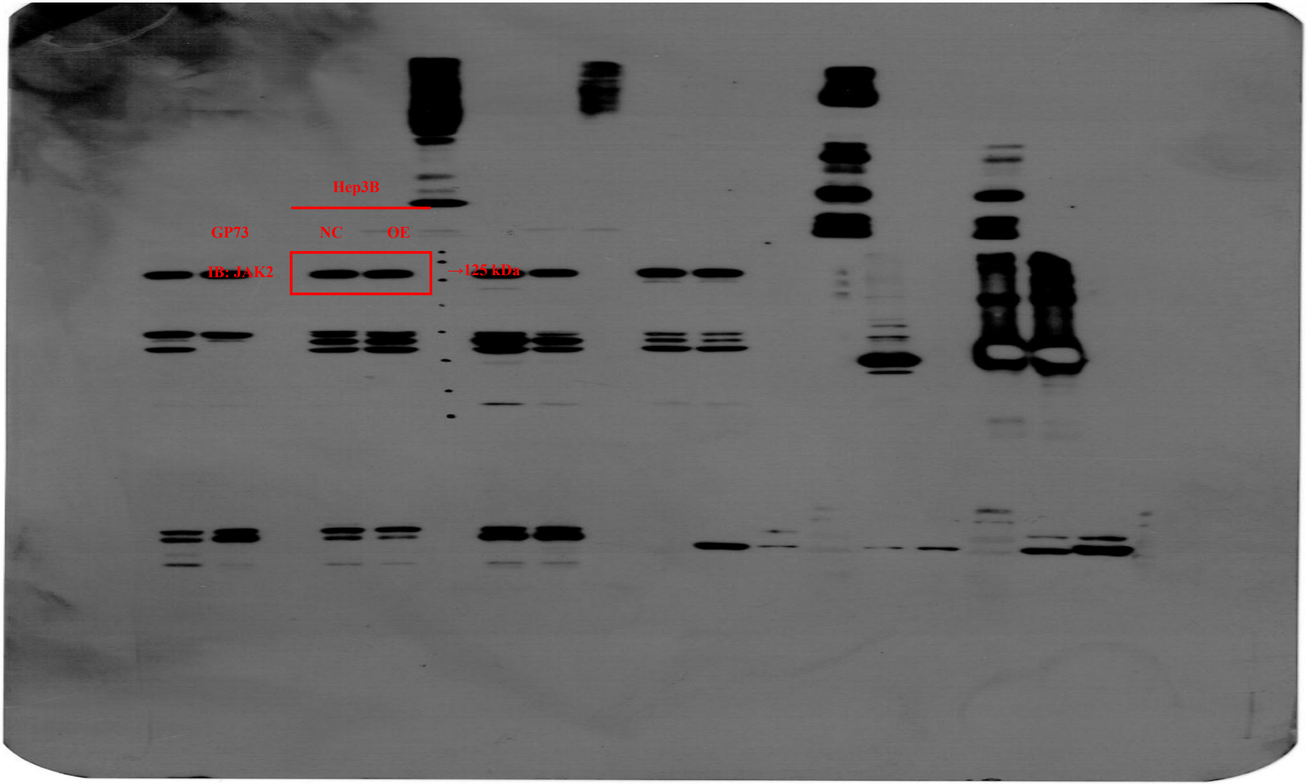

Figure 5K

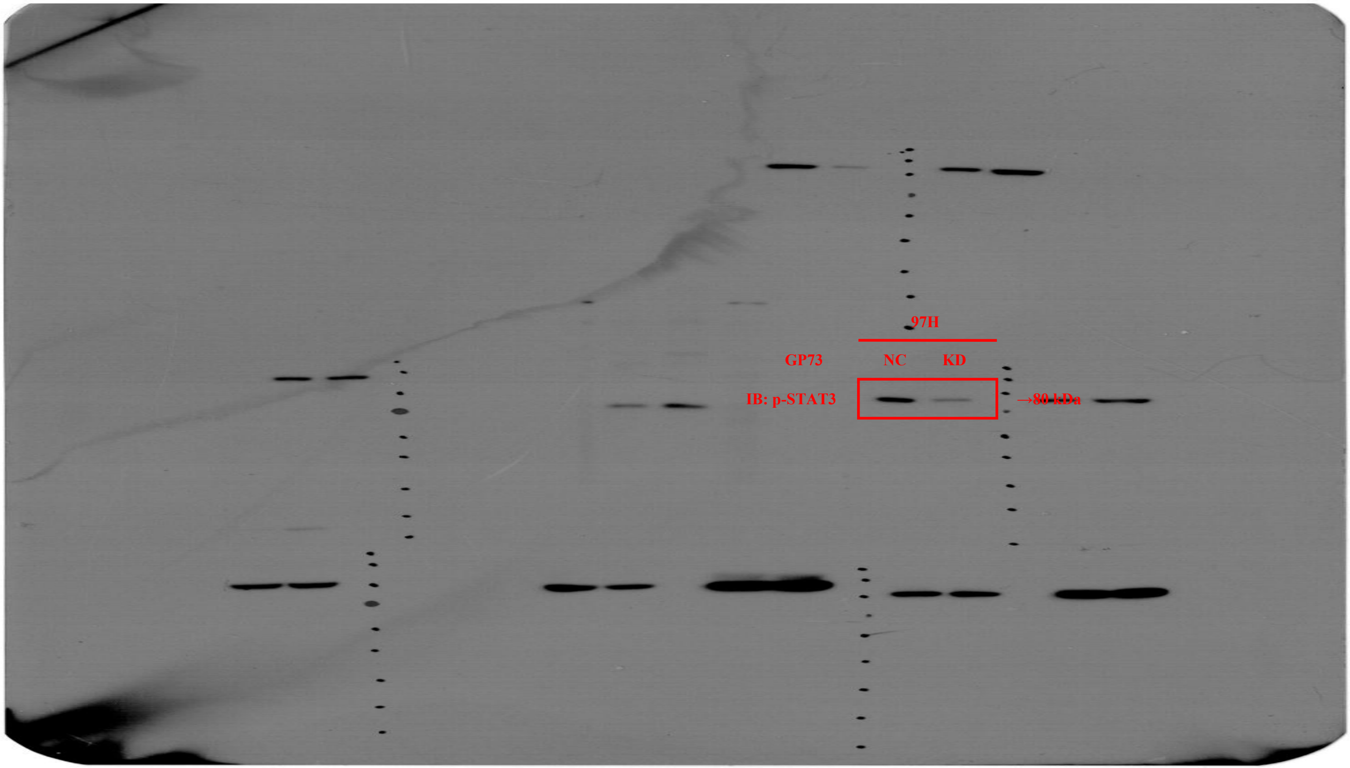

Figure 5K

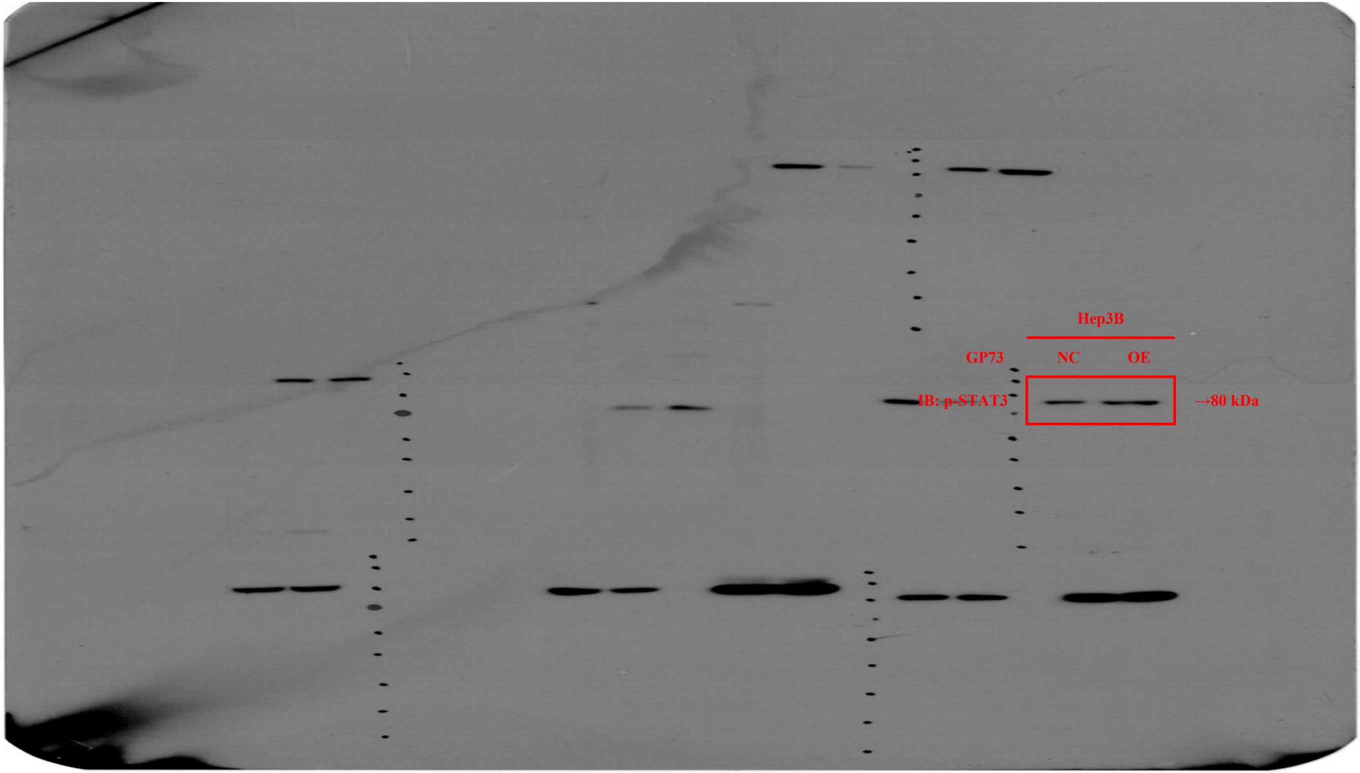

Figure 5K

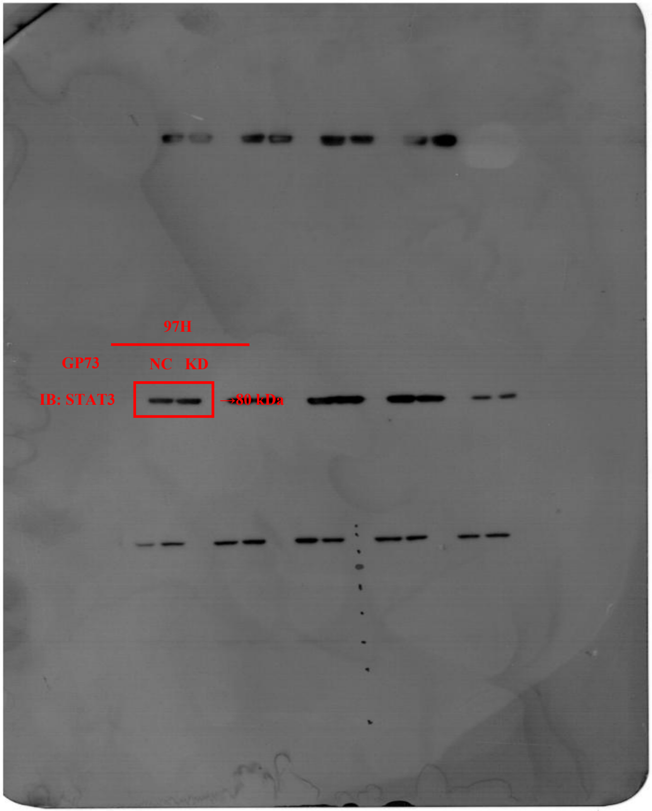

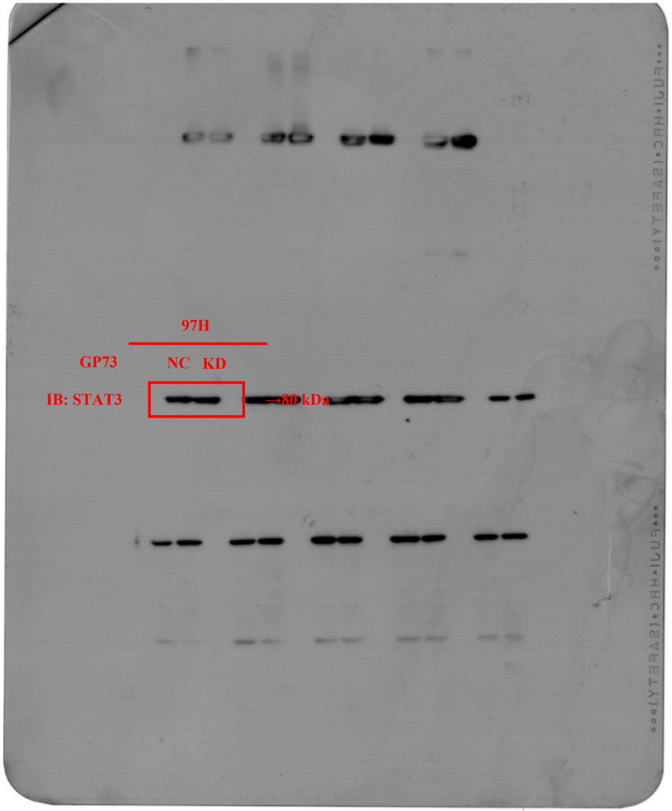

Figure 5K

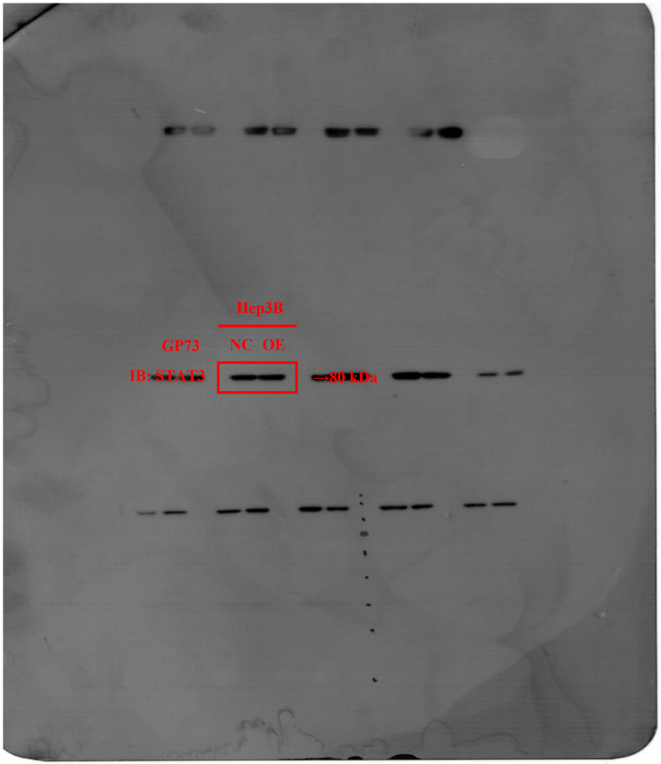
Hep3B
GP73
NC OE
IB: STAT3
→80 kDa

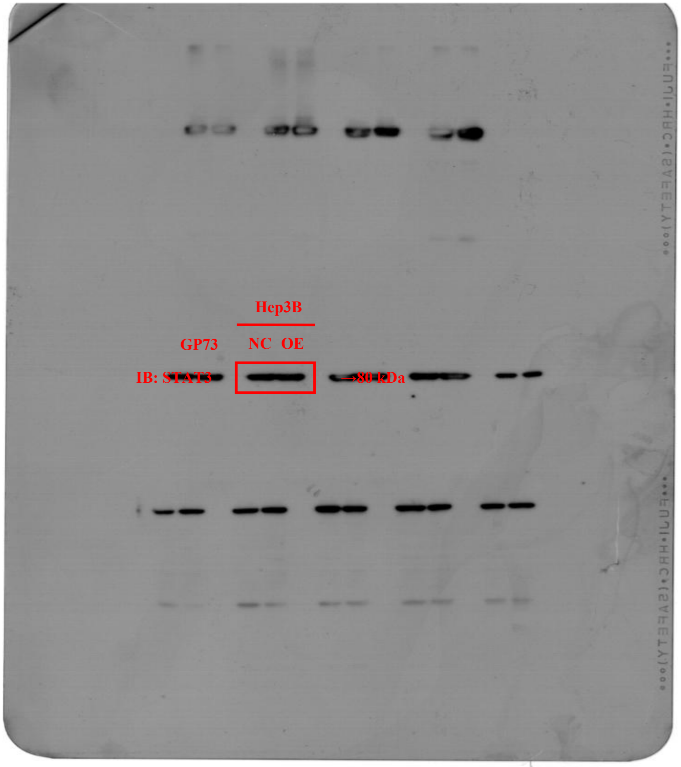
Hep3B
GP73
NC OE
IB: STAT3
→80 kDa

Figure 5K

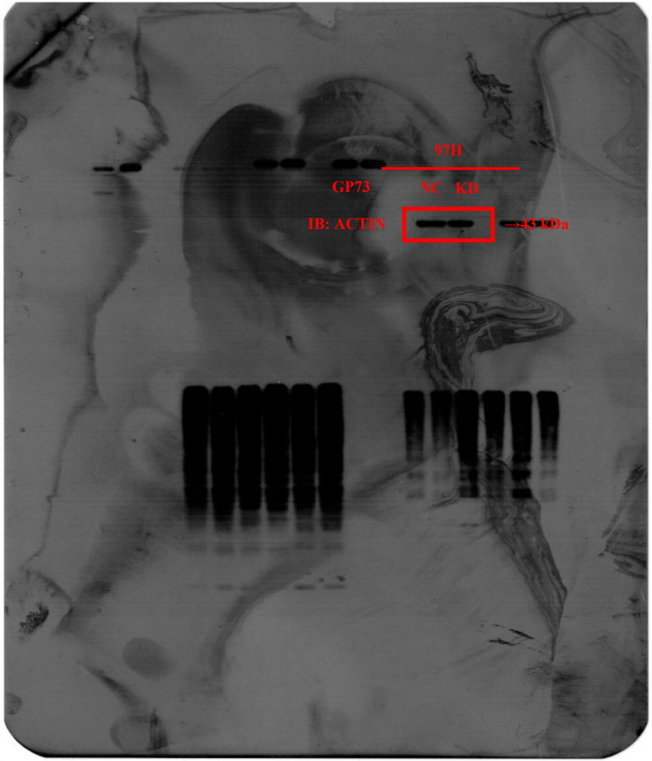

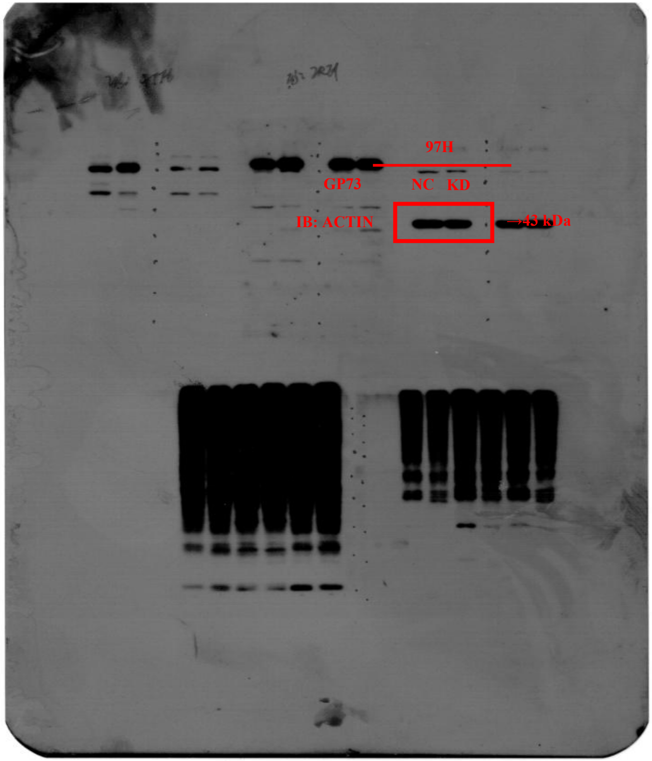

Figure 5K

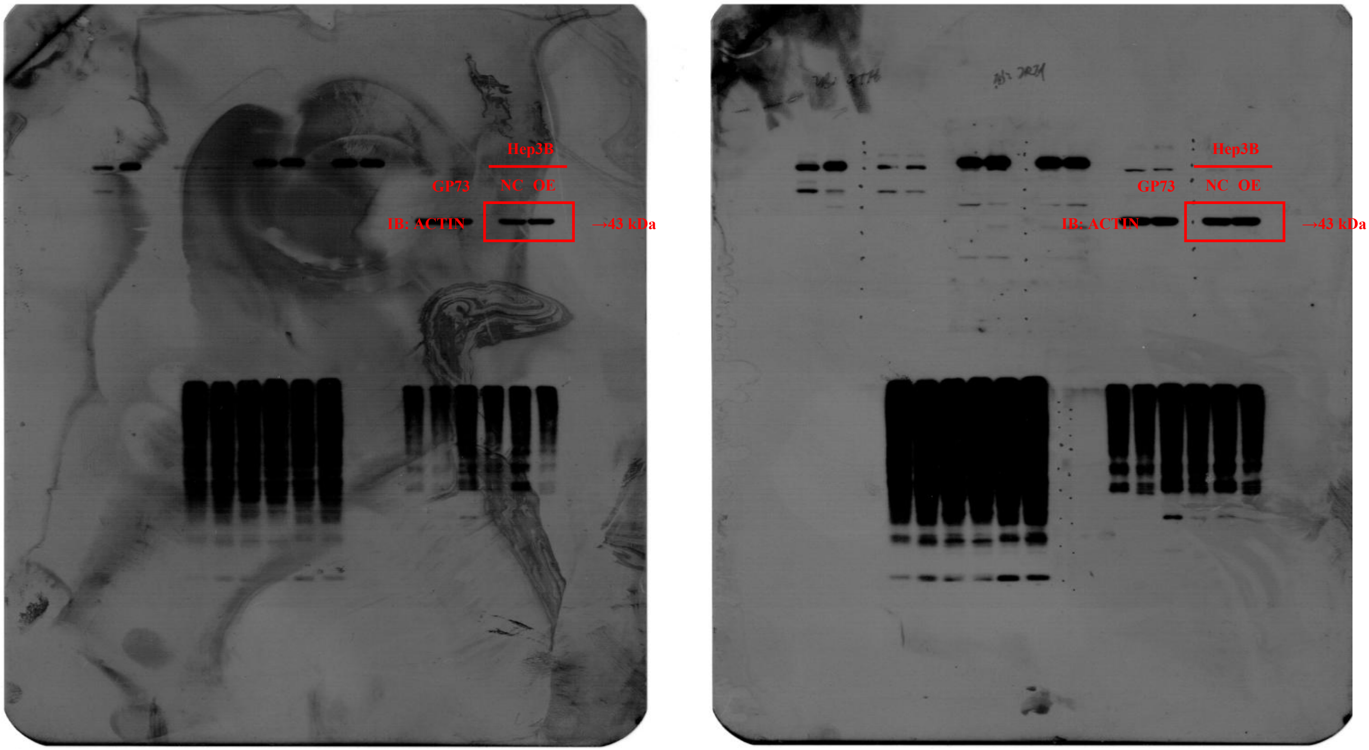
Hep3B
GP73
NC OE
IB: ACTIN
→43 kDa
Hep3B
GP73
NC OE
IB: ACTIN
→43 kDa

Figure 5L

Western blots determined the impact of over-expressed GRP78 on the alterations of key genes expressions on the JAK2/STAT3 and ERS pathways in MHCC97H-GP73-KD cells

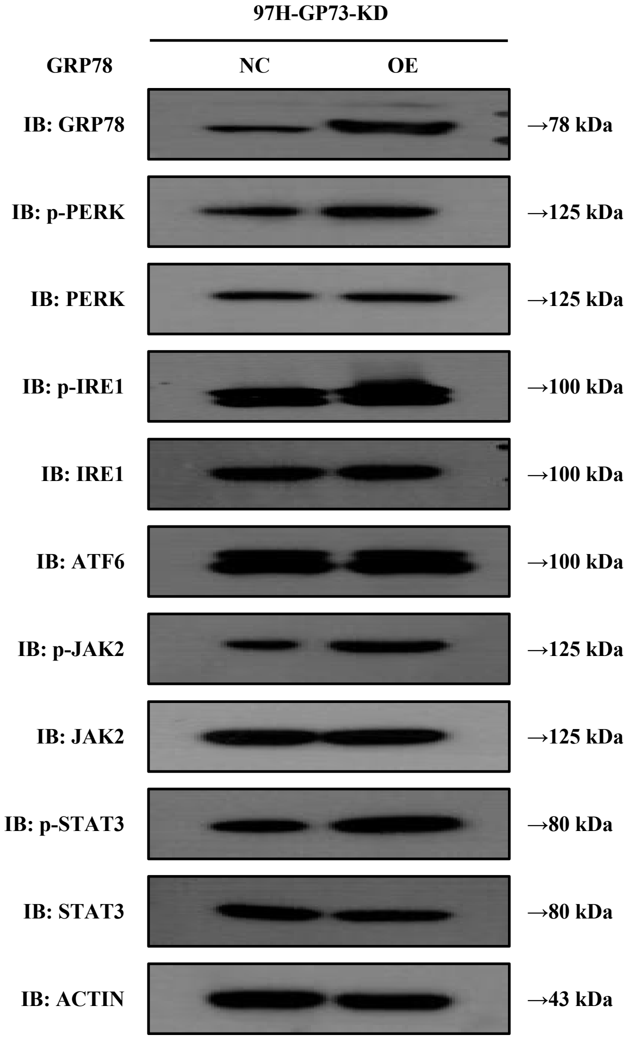

Figure 5L

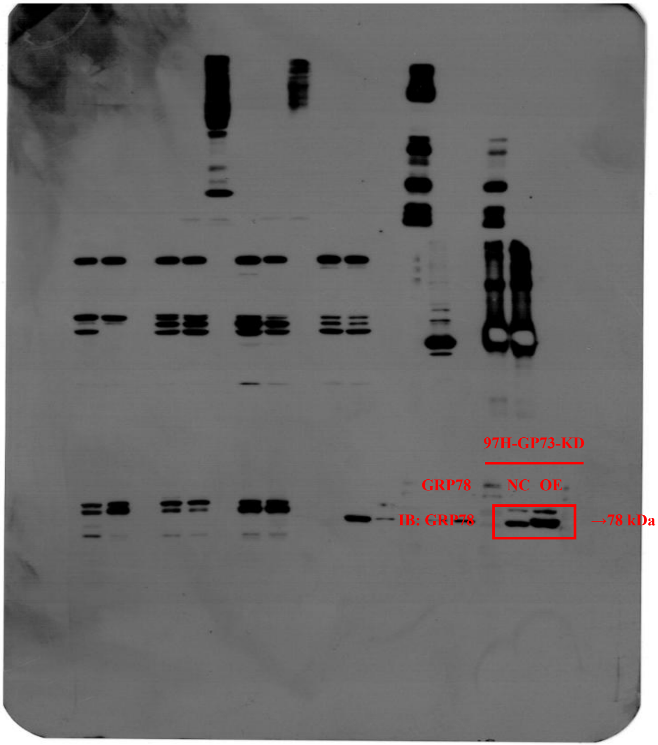
97H-GP73-KD
GRP78
NC OE
IB: GRP78
→78 kDa

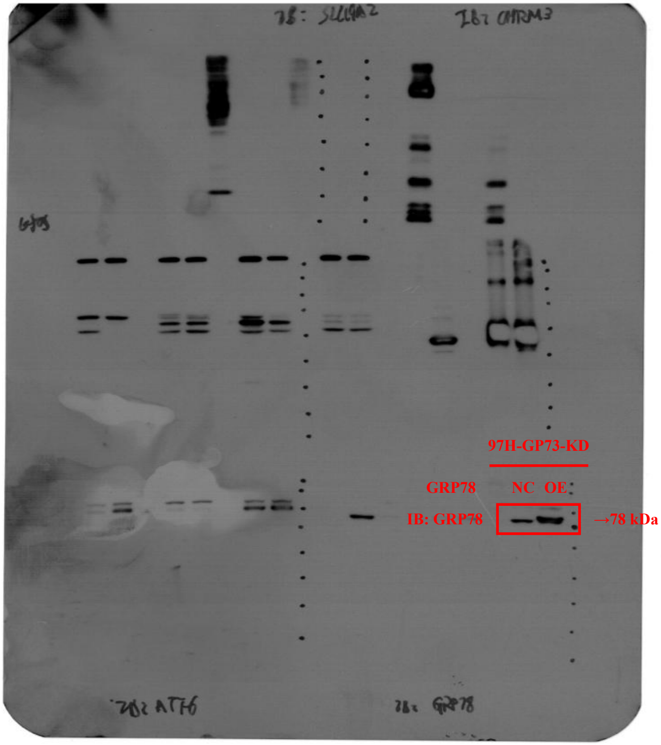
97H-GP73-KD
GRP78
NC OE
IB: GRP78
→78 kDa

Figure 5L

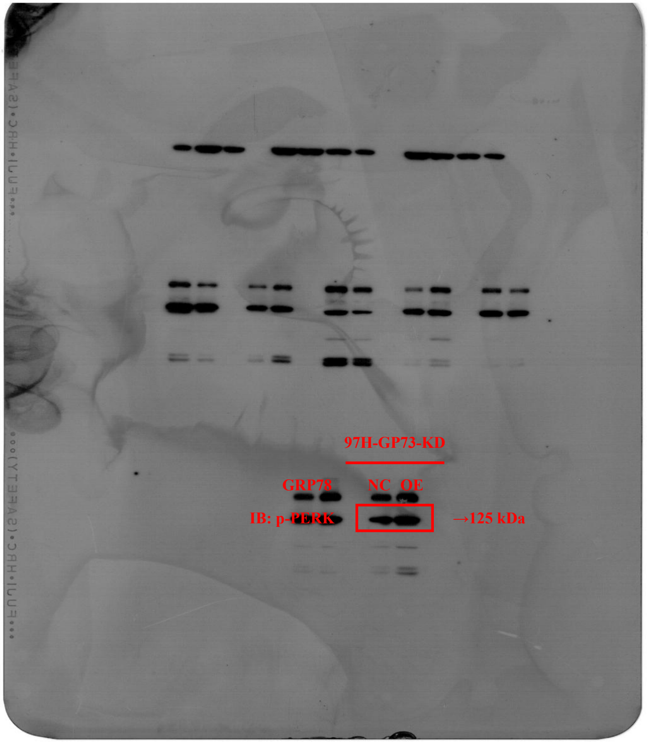

97H-GP73-KD
GRP78
NC
OE
IB: p-PERK
→125 kDa

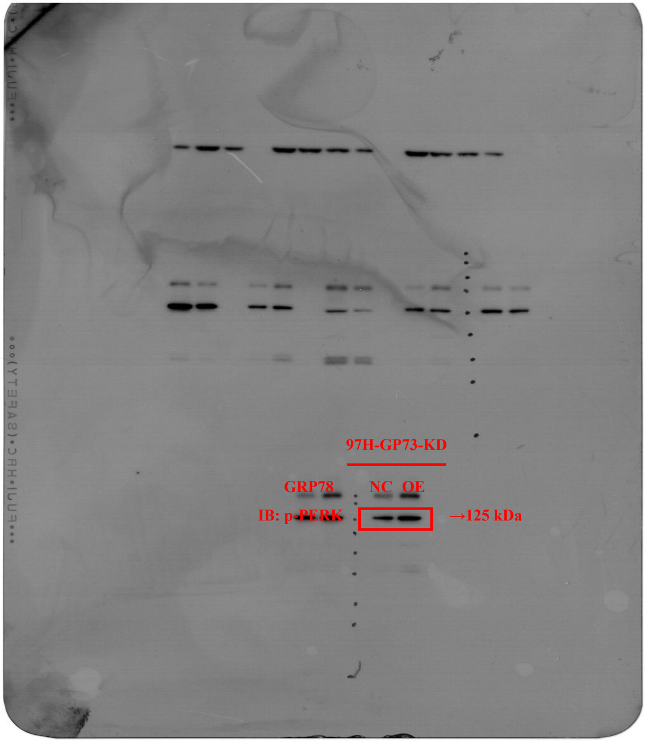

97H-GP73-KD
GRP78
NC
OE
IB: p-PERK
→125 kDa

Figure 5L

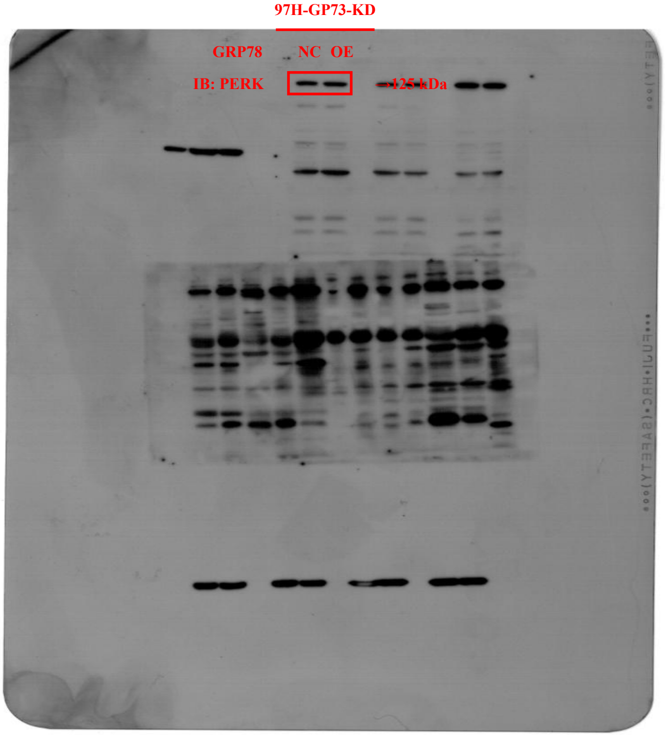
97H-GP73-KD
GRP78
NC OE
IB: PERK
→125 kDa

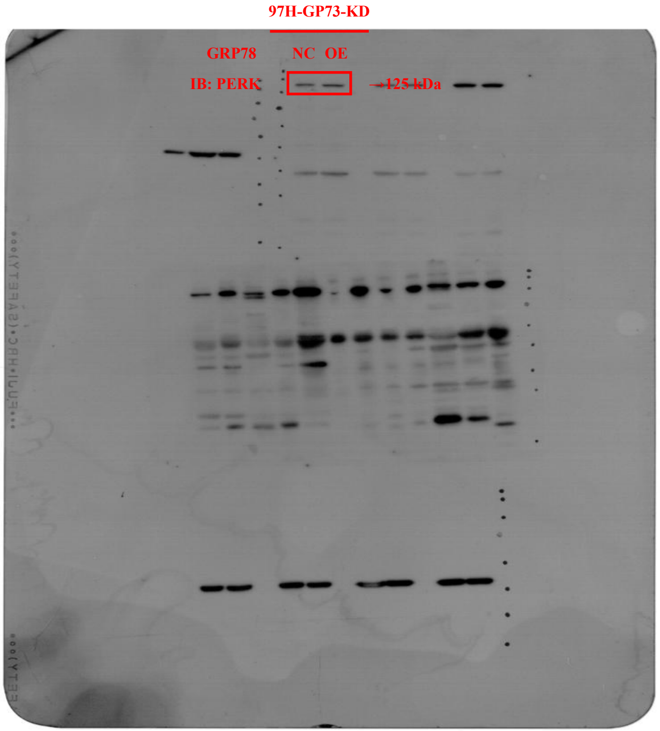
97H-GP73-KD
GRP78
NC OE
IB: PERK
→125 kDa

Figure 5L

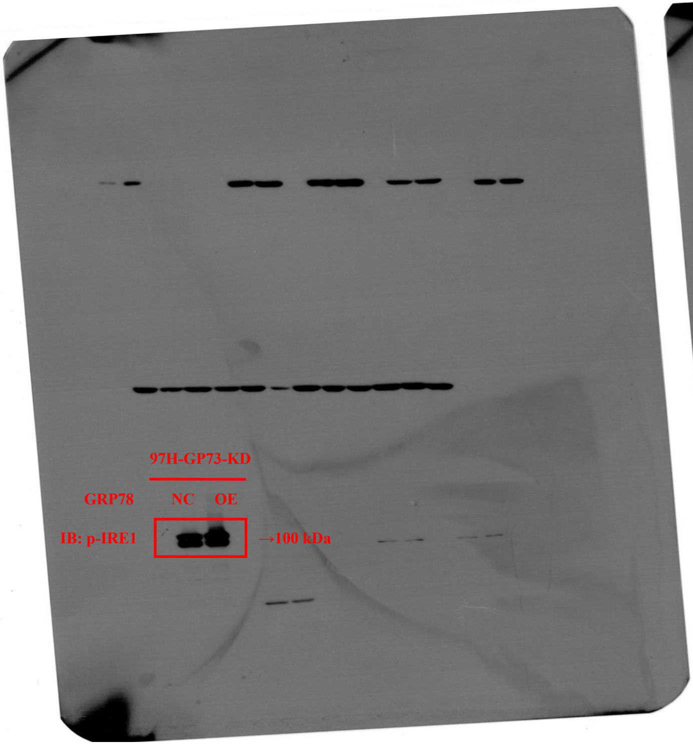
97H-GP73-KD
GRP78
NC
OE
IB: p-IRE1
→100 kDa

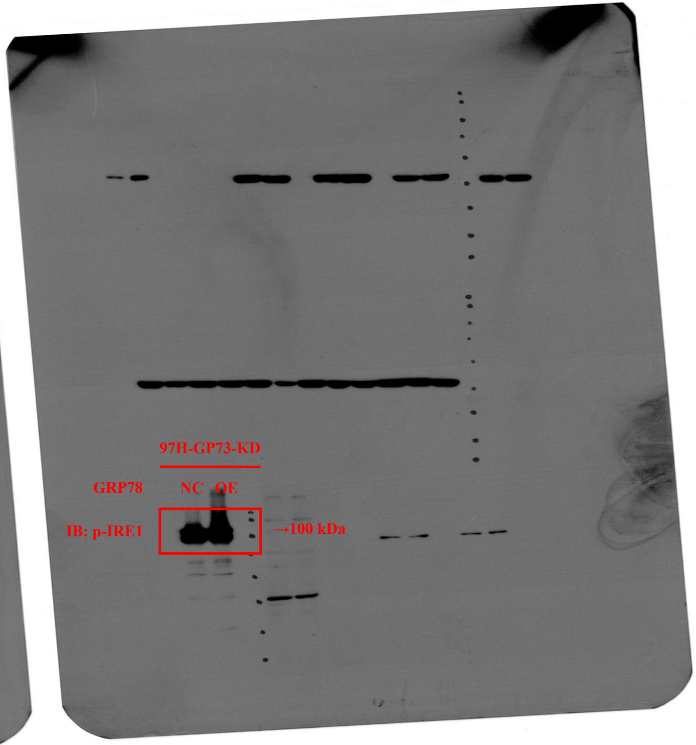
97H-GP73-KD
GRP78
NC
OE
IB: p-IRE1
→100 kDa

Figure 5L

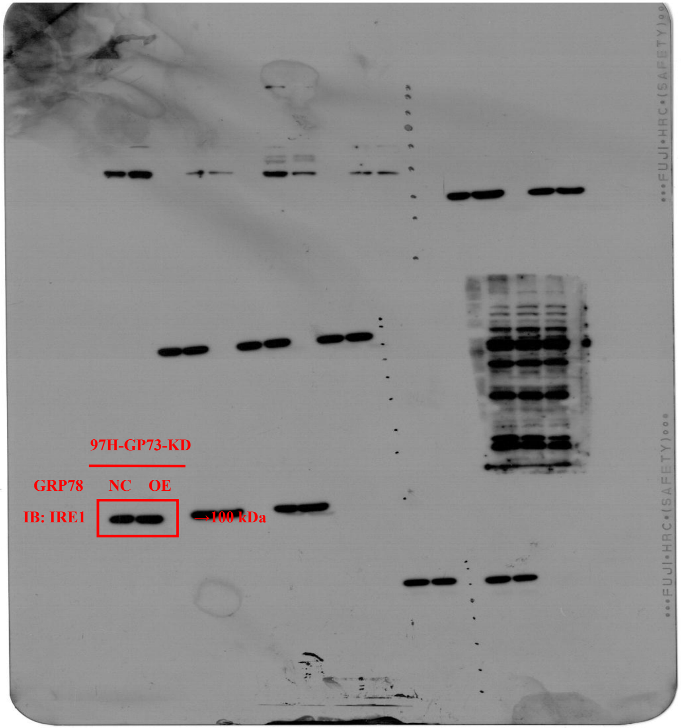

97H-GP73-KD
GRP78
NC
OE
IB: IRE1
→100 kDa

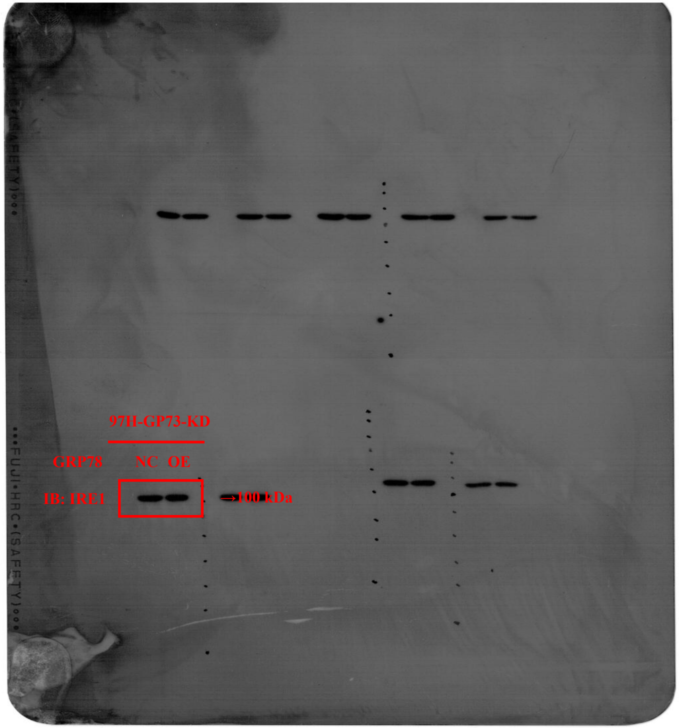

97H-GP73-KD
GRP78
NC
OE
IB: IRE1
→100 kDa

Figure 5L

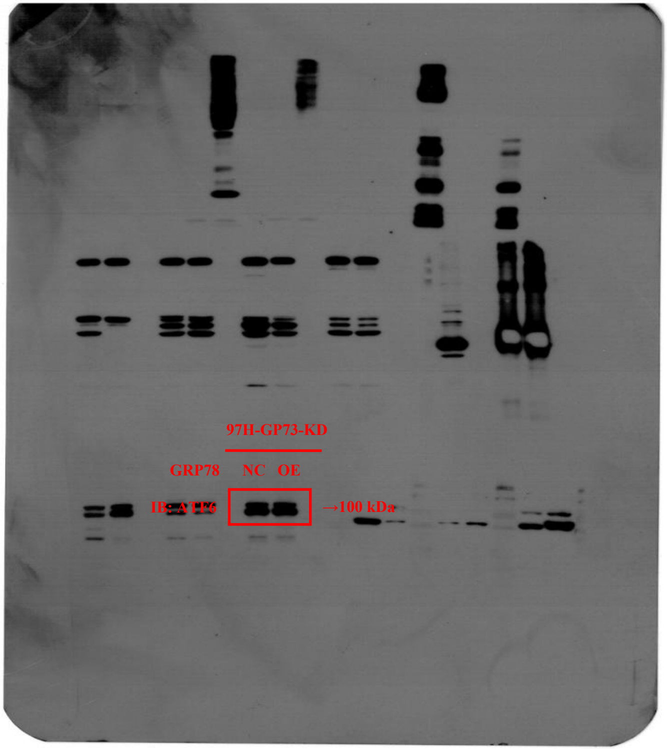
97H-GP73-KD
GRP78
NC
OE
IB: ATF6
→100 kDa

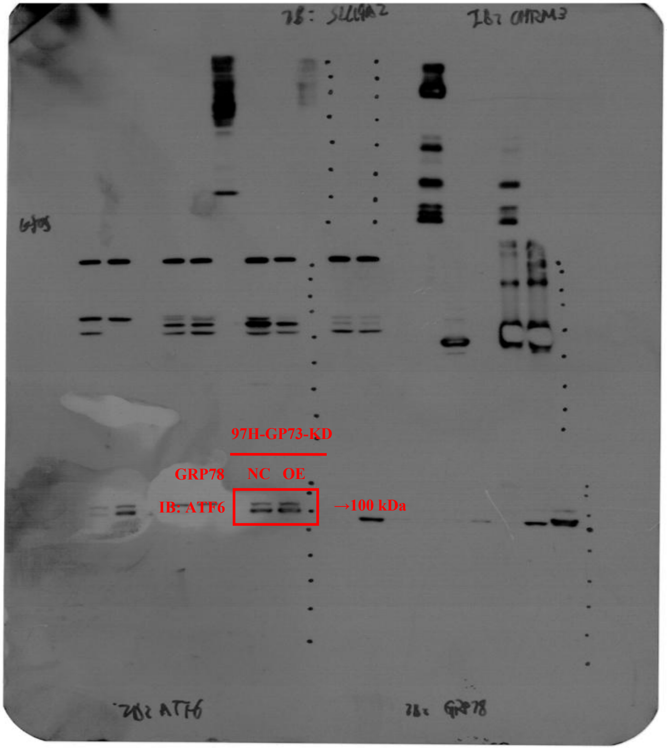
97H-GP73-KD
GRP78
NC
OE
IB: ATF6
→100 kDa

Figure 5L

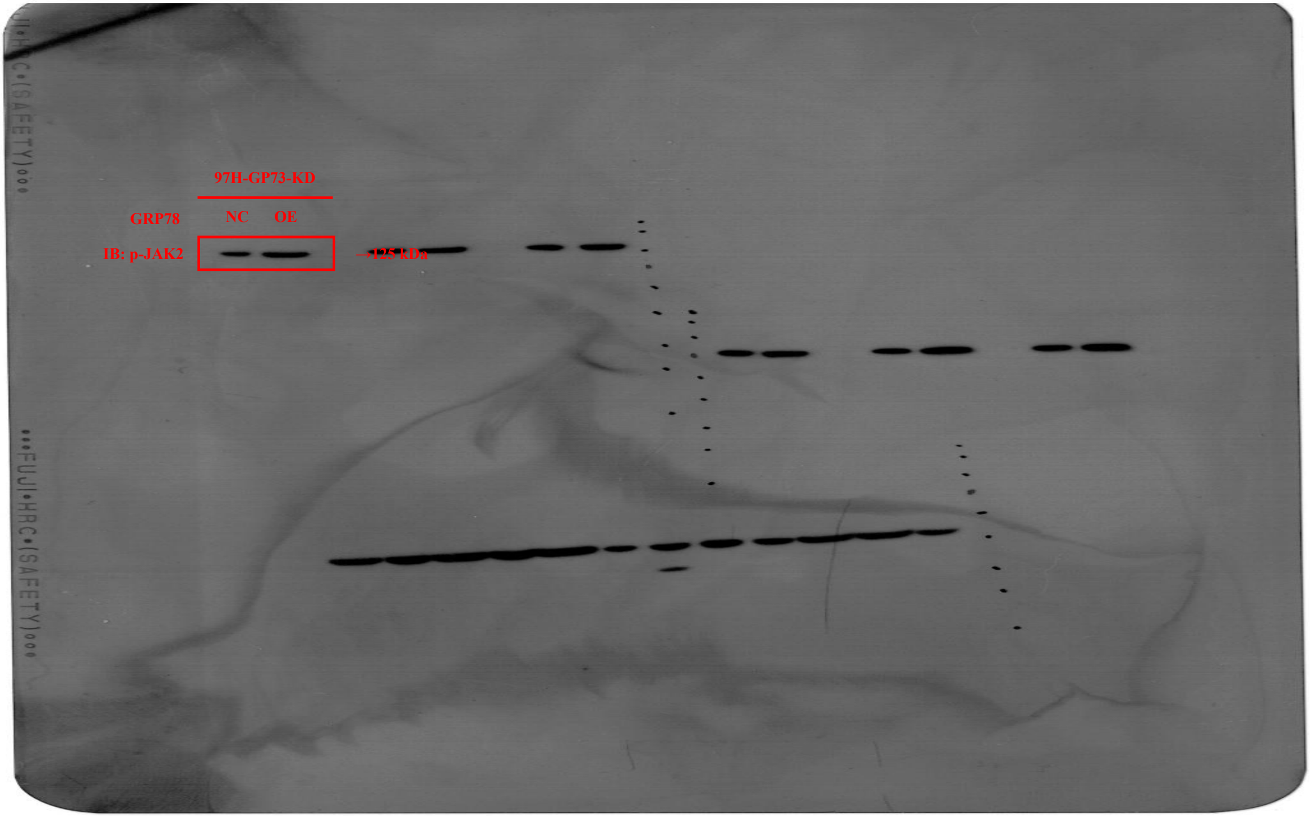

Figure 5L

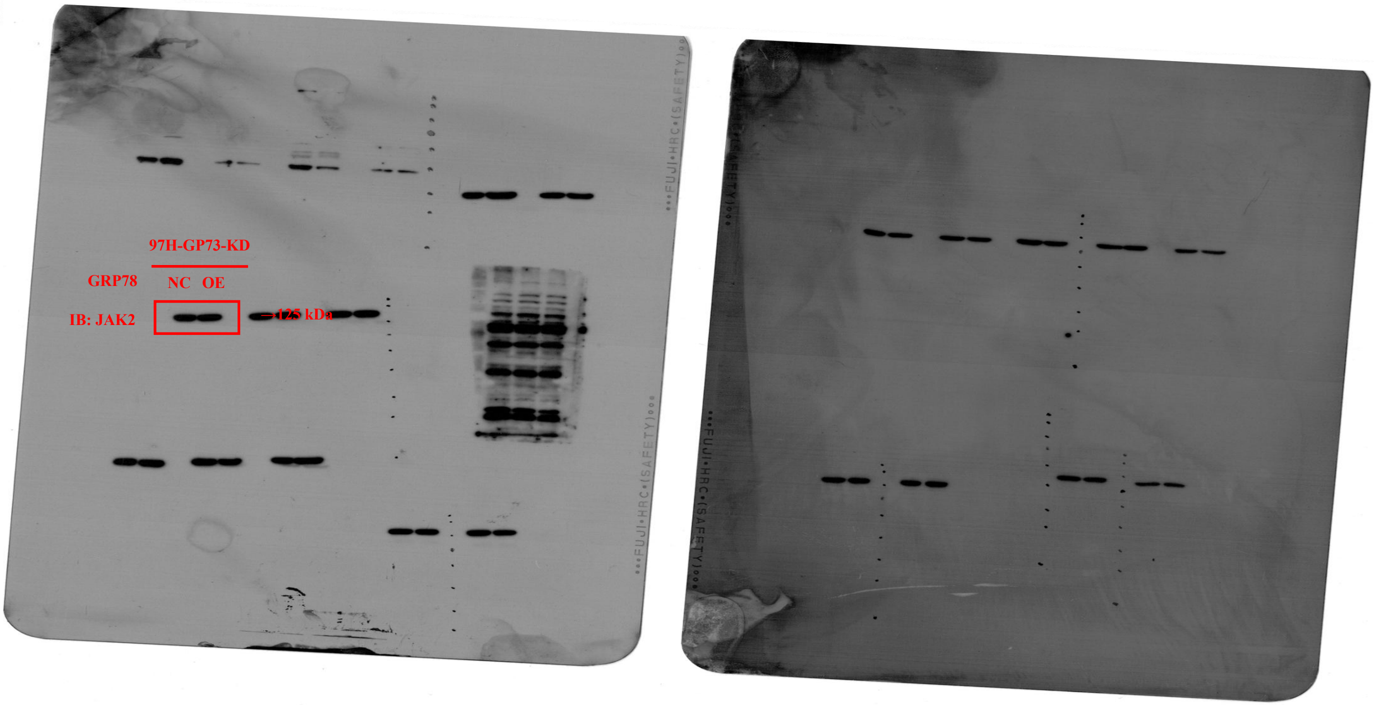

Figure 5L

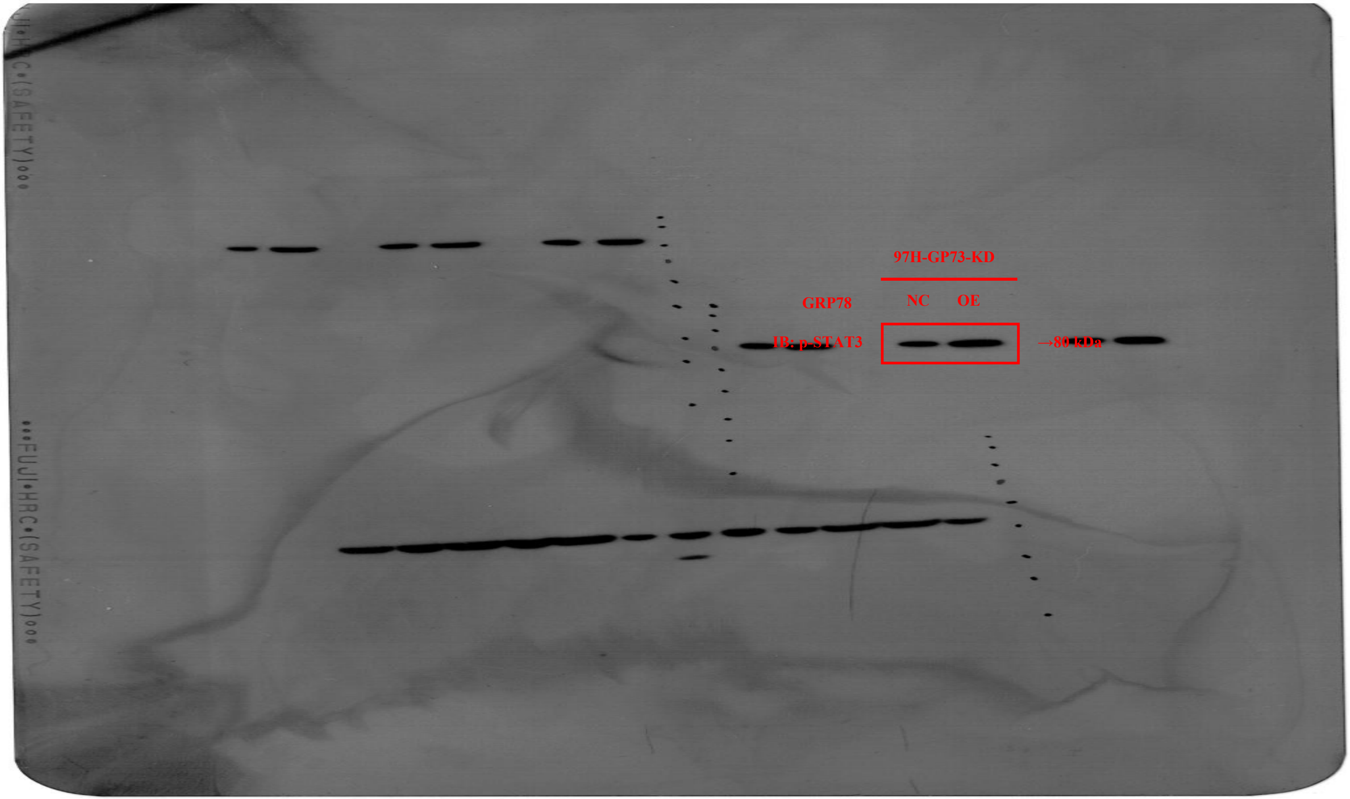

Figure 5L

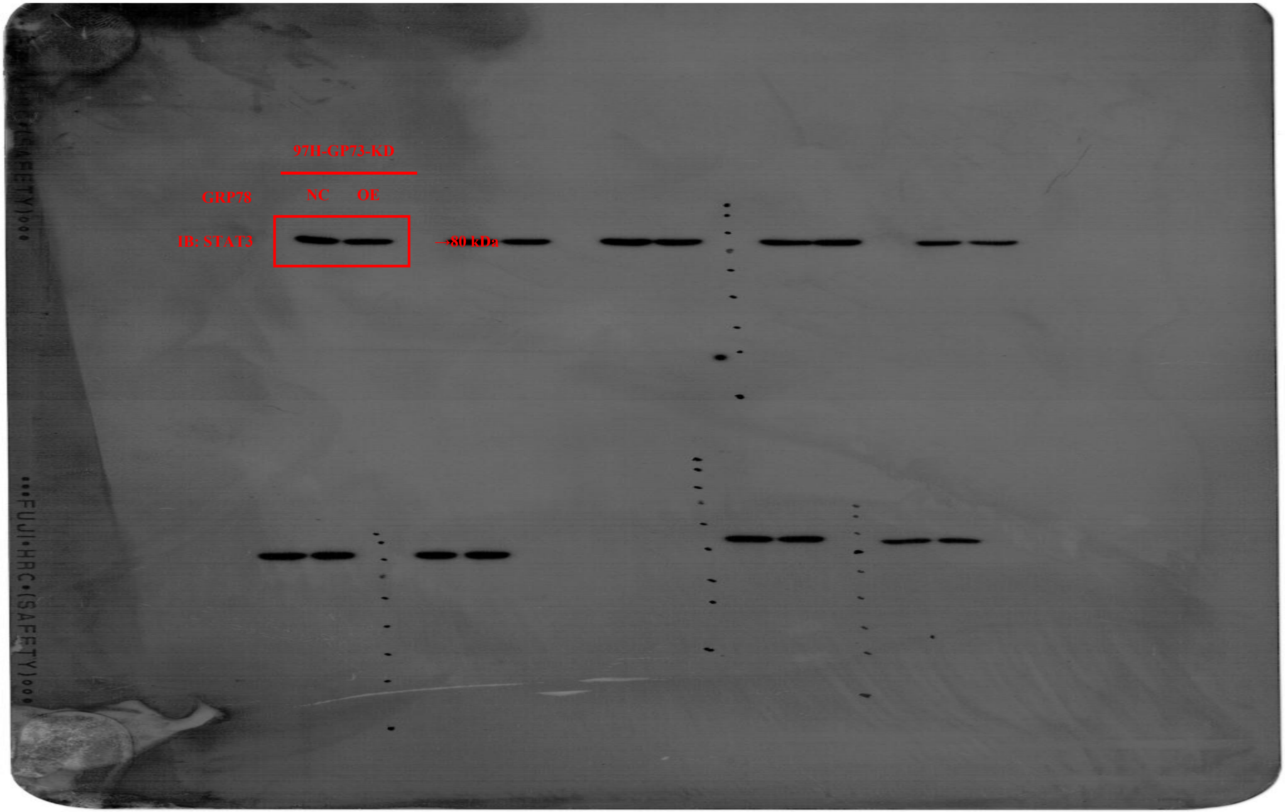

Figure 5L

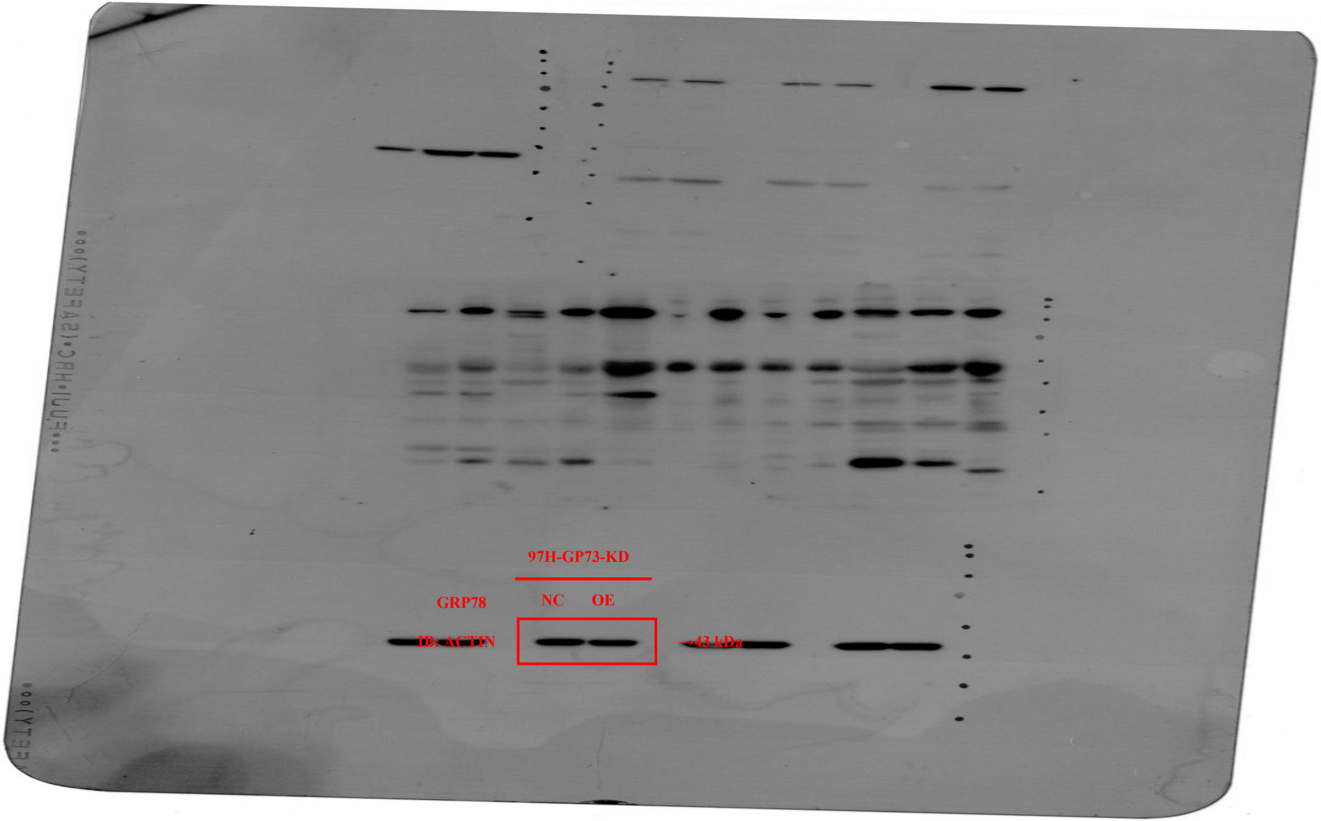

Figure 6B

Western blot detection of Pan kla, H3K18la, H4K5la and GP73 expressions in MHCC97H cells.

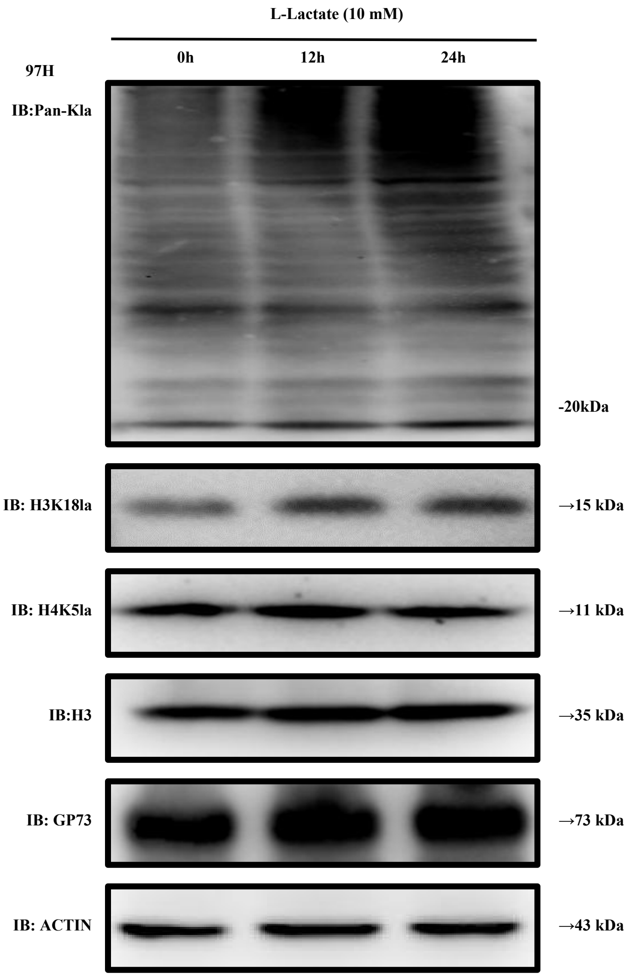

Figure 6B

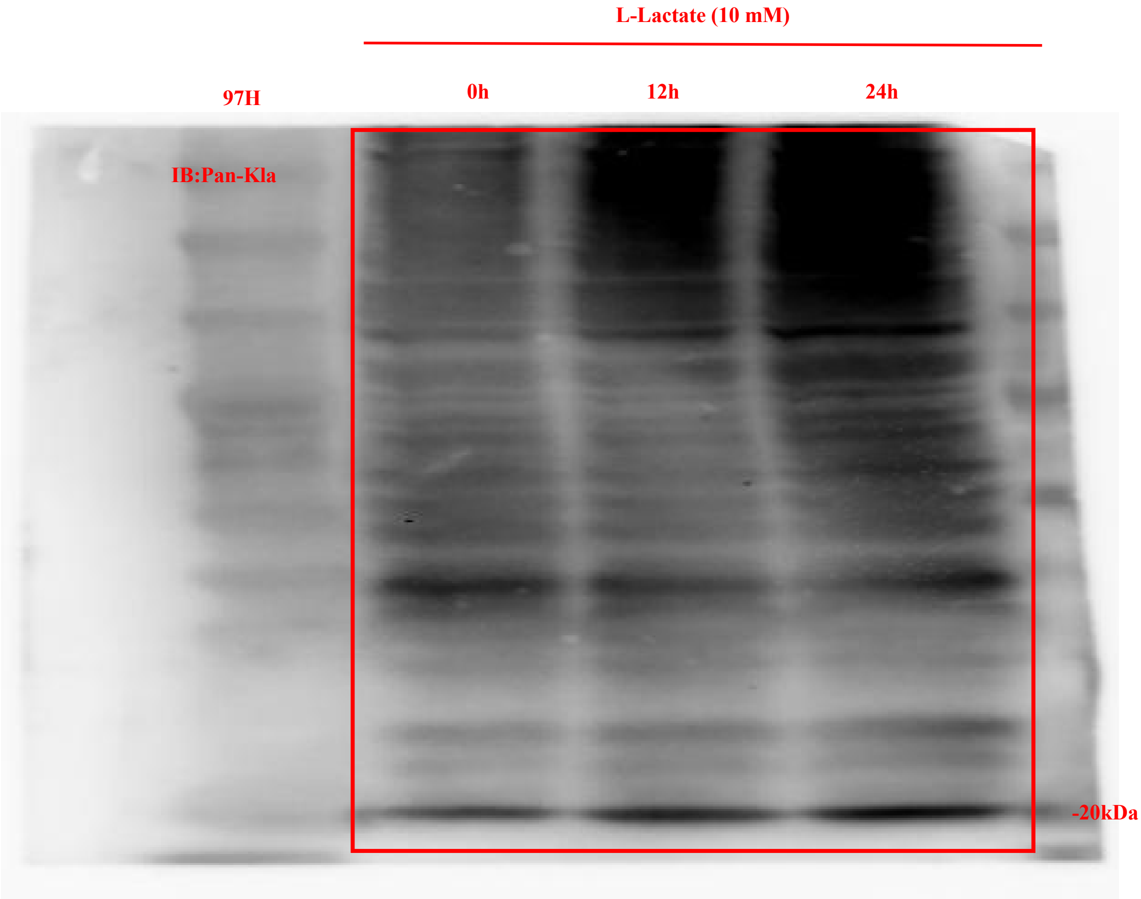

Figure 6B

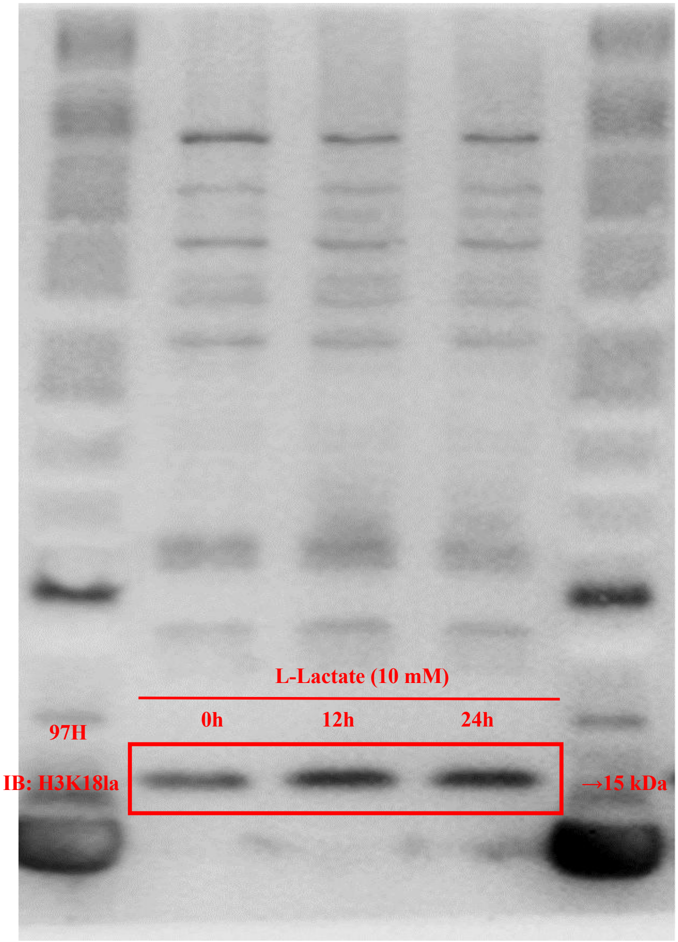

Figure 6B

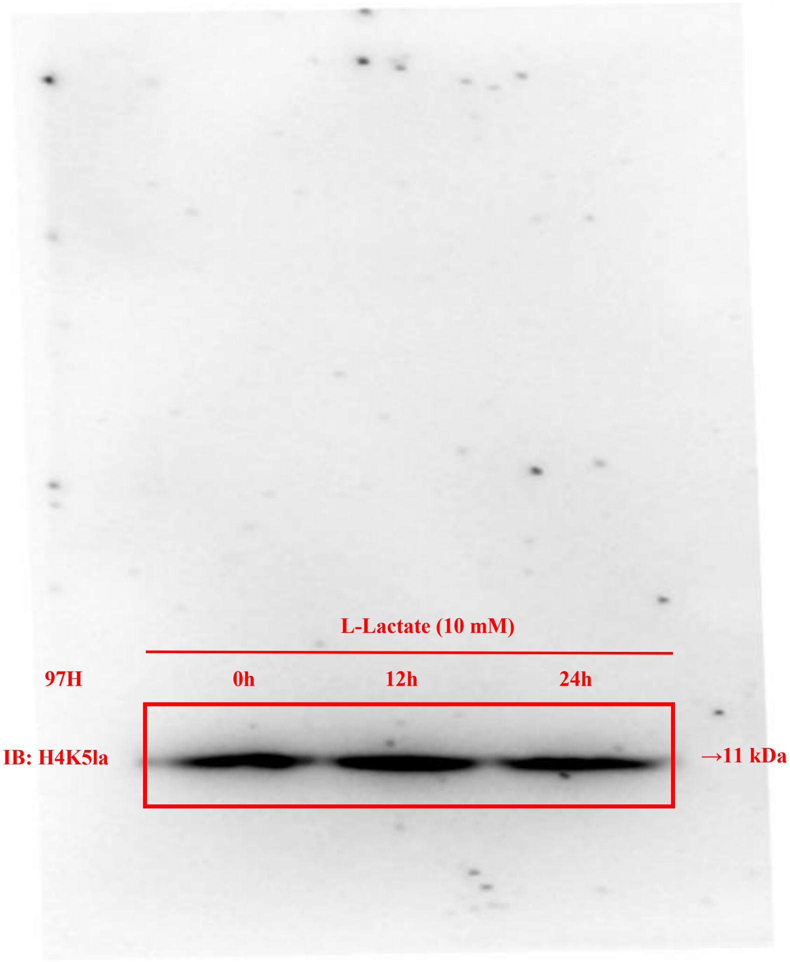

Figure 6B

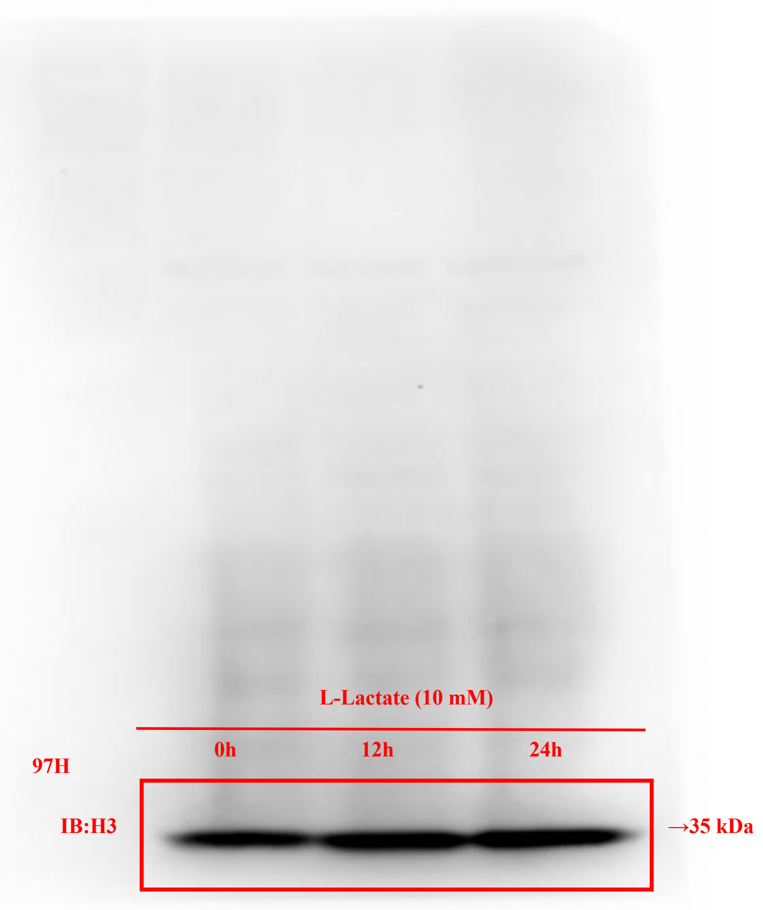

Figure 6B

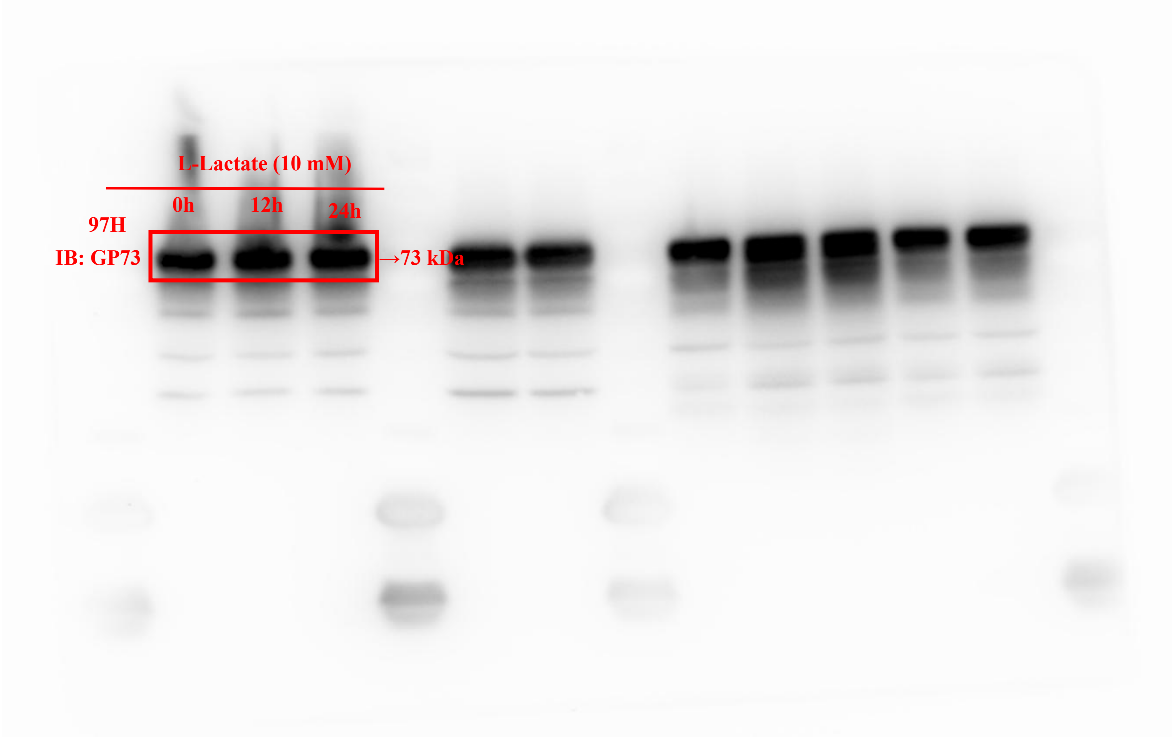

Figure 6B

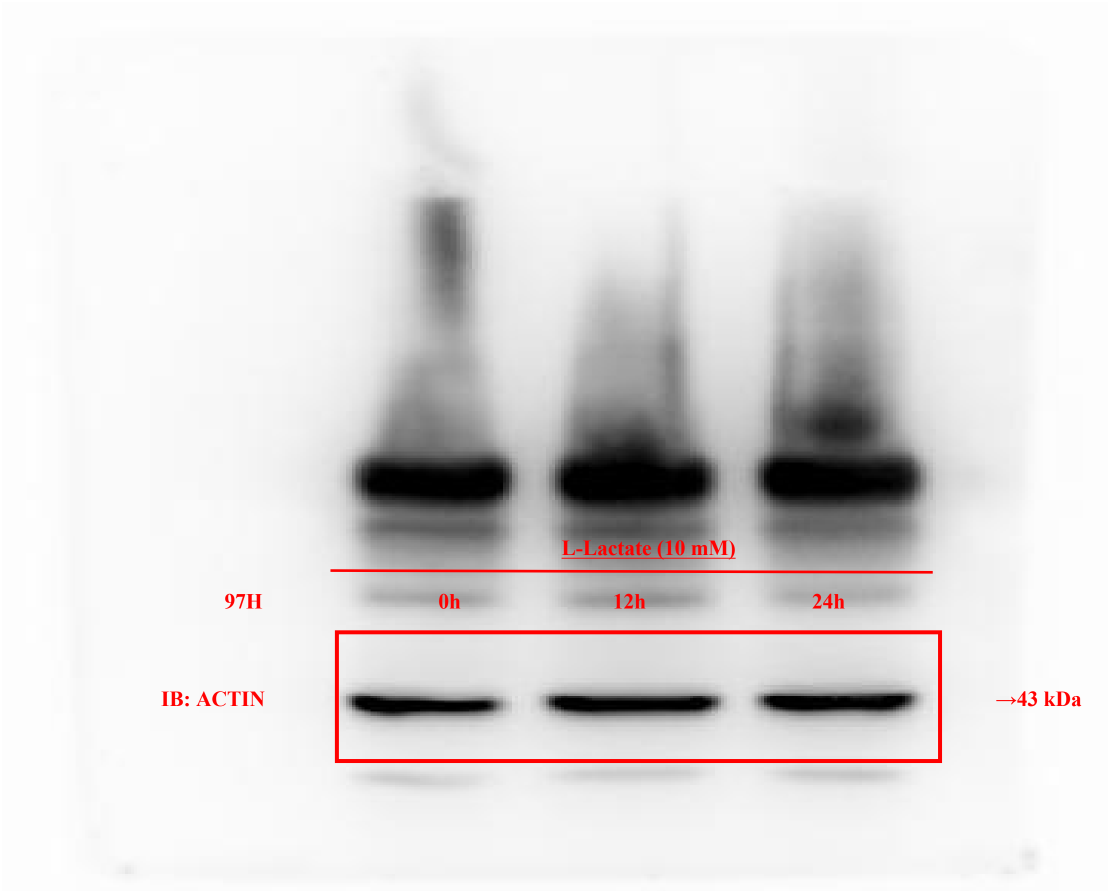

Figure 6C

2-DG and LDH-A inhibitor (sodium oxalate) indicates MHCC97H cells were treated with 2-DG (10uM, 20uM) and LDH-A inhibitors (sodium oxalate) (10uM, 20uM) for 24 hours.

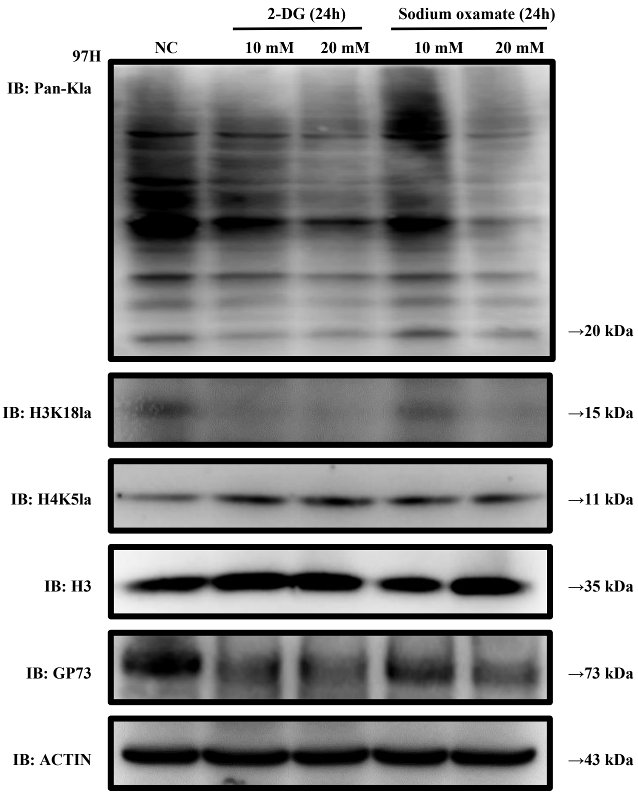

Figure 6C

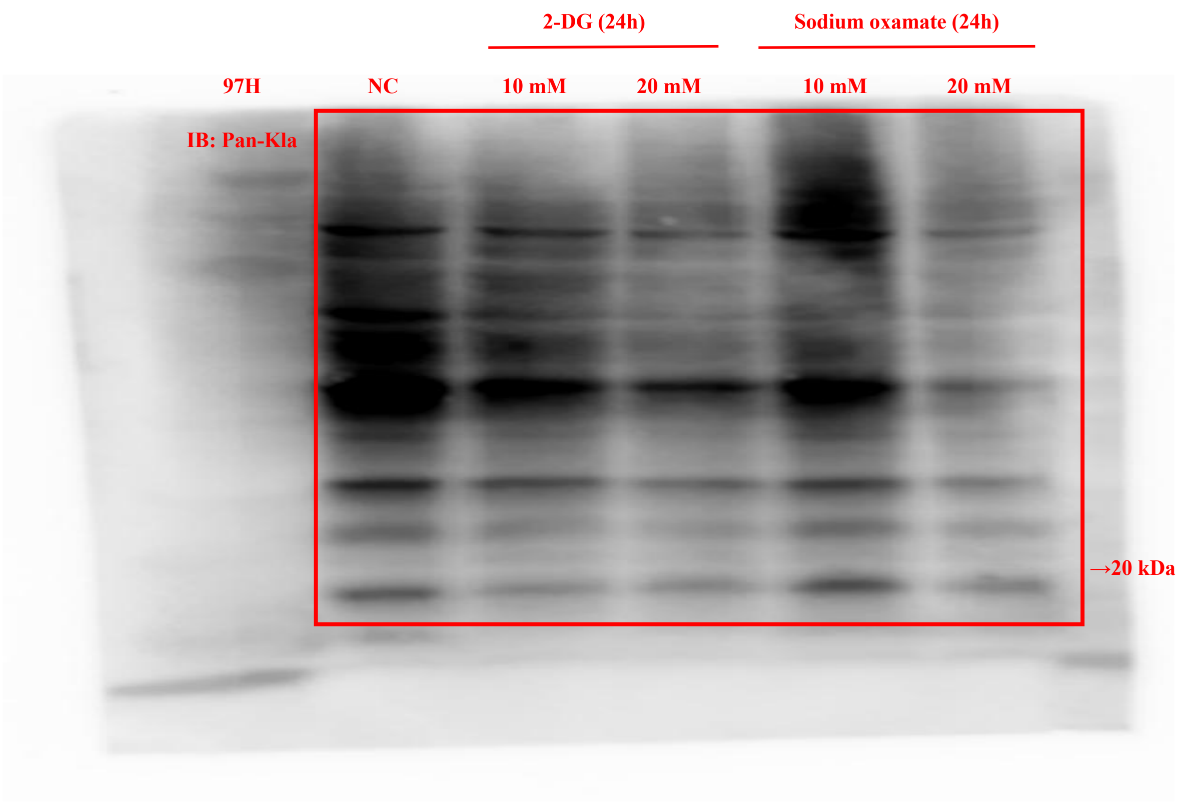

2-DG (24h)
Sodium oxamate (24h)
97H
NC
10 mM
20 mM
10 mM
20 mM
IB: Pan-Kla
→20 kDa

Figure 6C

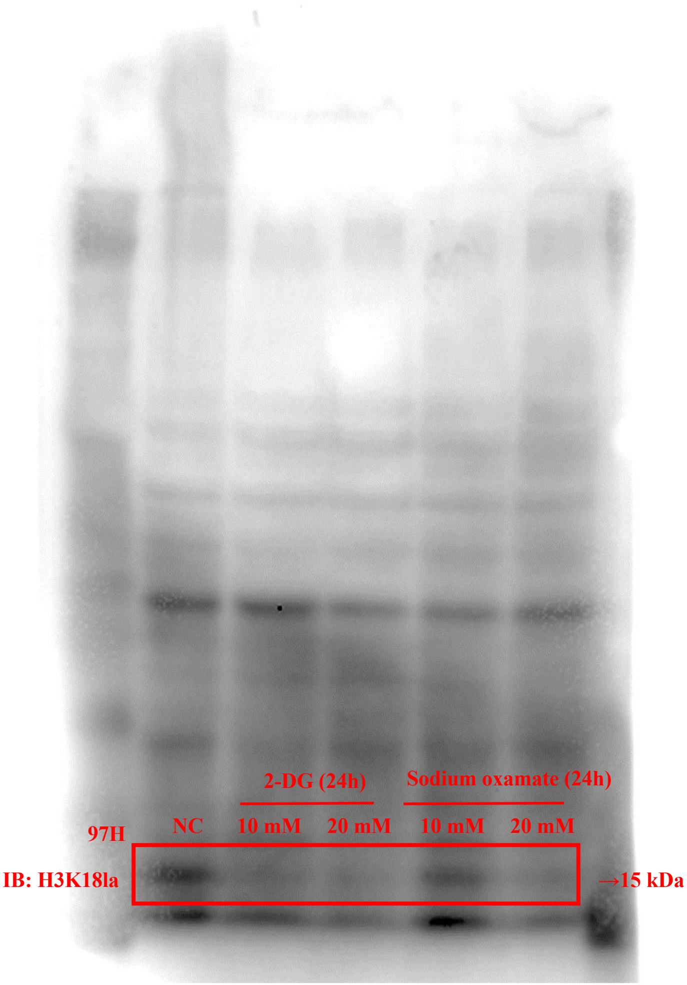
2-DG (24h)
Sodium oxamate (24h)
97H
NC
10 mM
20 mM
10 mM
20 mM
IB: H3K18la
→15 kDa

Figure 6C

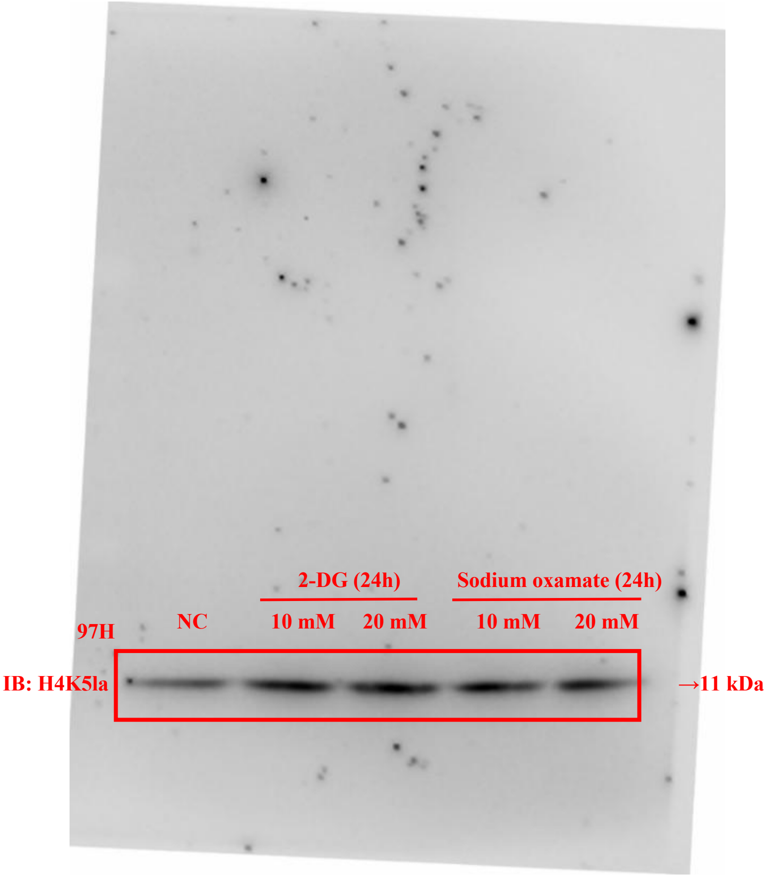

2-DG (24h)
Sodium oxamate (24h)
97H
NC
10 mM
20 mM
10 mM
20 mM
IB: H4K5la
→11 kDa

Figure 6C

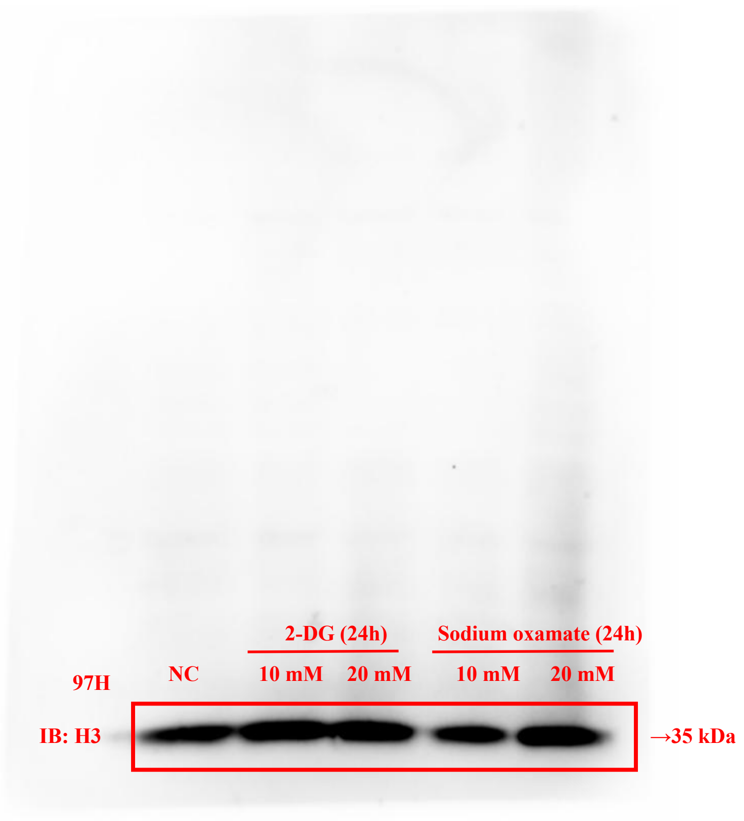
2-DG (24h)
Sodium oxamate (24h)
97H
NC
10 mM
20 mM
10 mM
20 mM
IB: H3
→35 kDa

Figure 6C

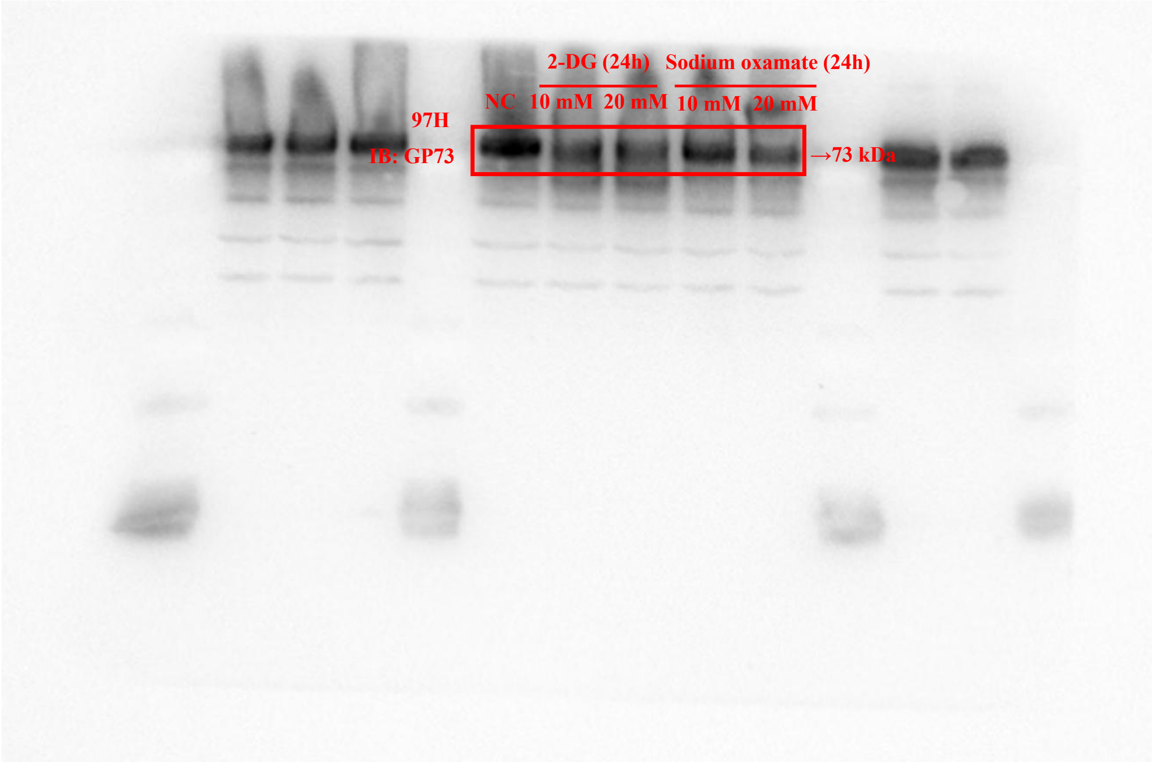

2-DG (24h)
Sodium oxamate (24h)
NC
10 mM
20 mM
10 mM
20 mM
97H
IB: GP73
→73 kDa

Figure 6C

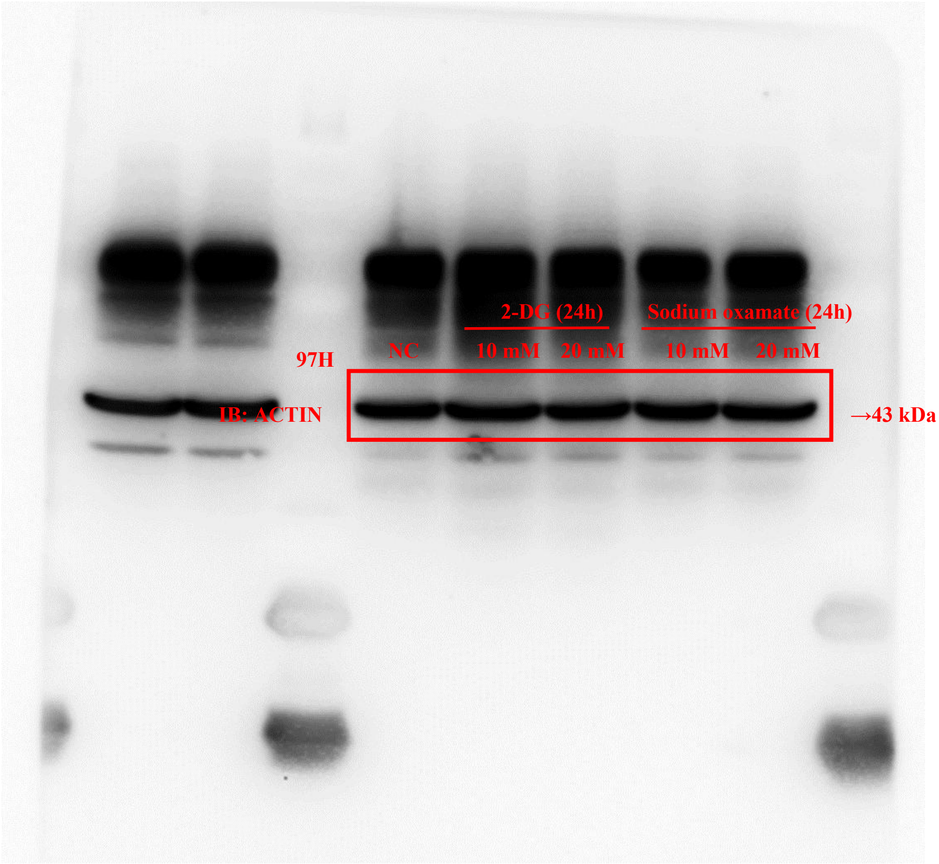

2-DG (24h)
Sodium oxamate (24h)
97H
NC
10 mM
20 mM
10 mM
20 mM
IB: ACTIN
→43 kDa

Figure 6D

Western blot detection of Pan kla, H3K18la, H4K5la and GP73 expressions in MHCC97H-LDHA-KD, MHCC97H-LDHB-KD and MHCC97H-LDHA-KD-LDHB-KD cells.

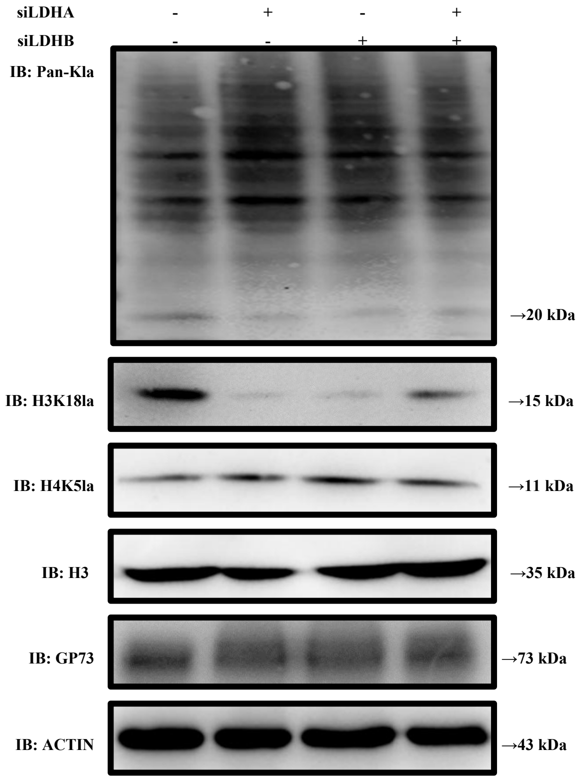

Figure 6D

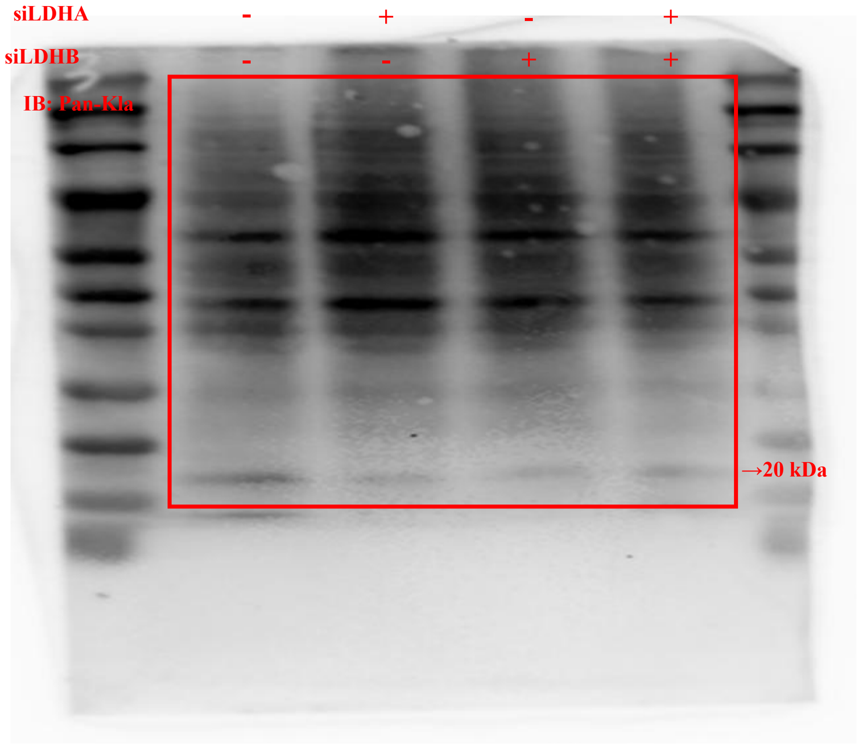

Figure 6D

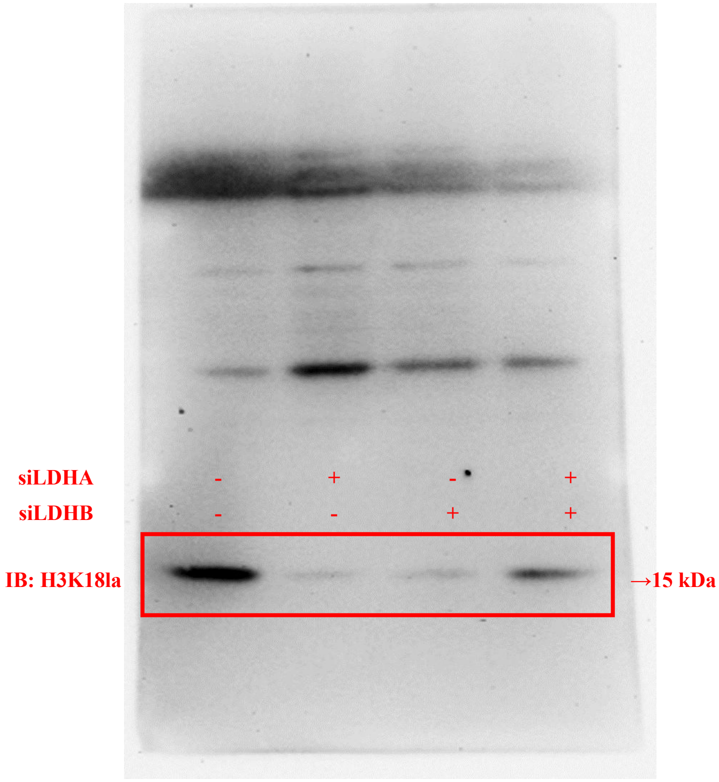

Figure 6D

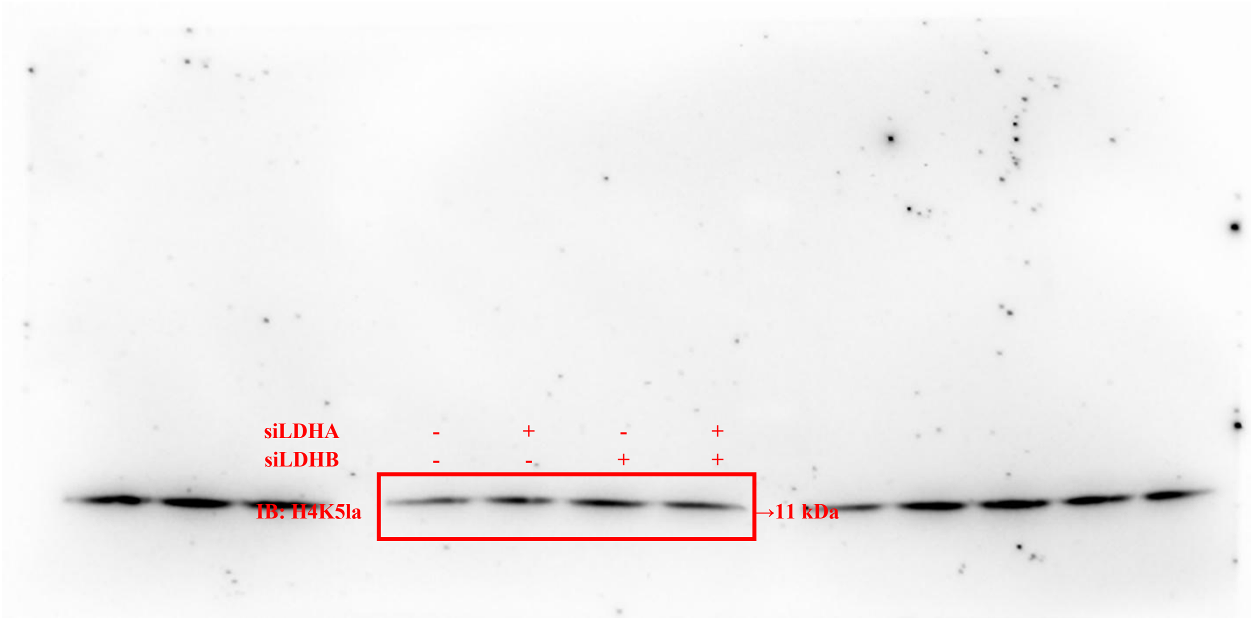

Figure 6D

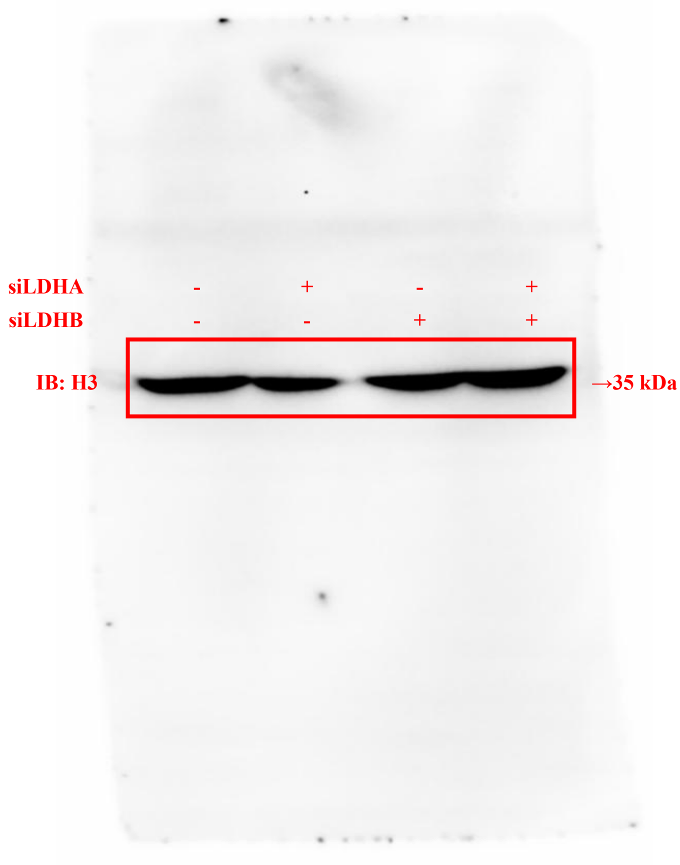

Figure 6D

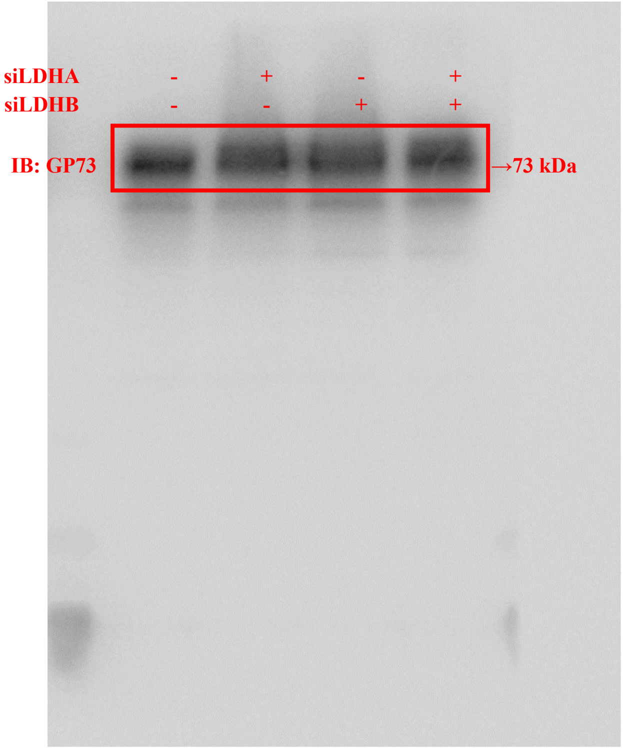

Figure 6D

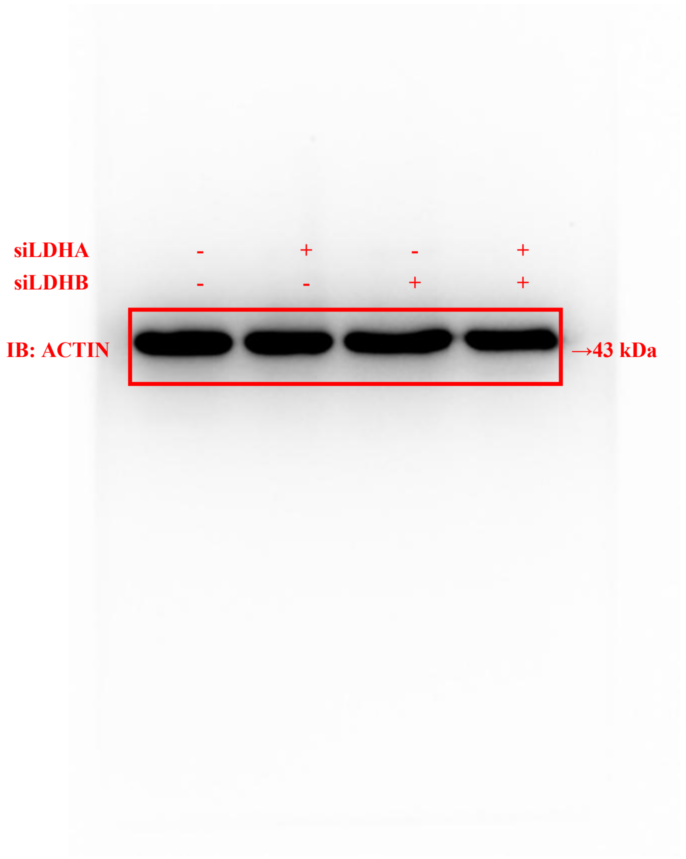

Figure 6E

Western blot detection of Pan kla, H3K18la, H4K5la and GP73 expressions in MHCC97H-P300-NC and MHCC97H-P300-KD cells.

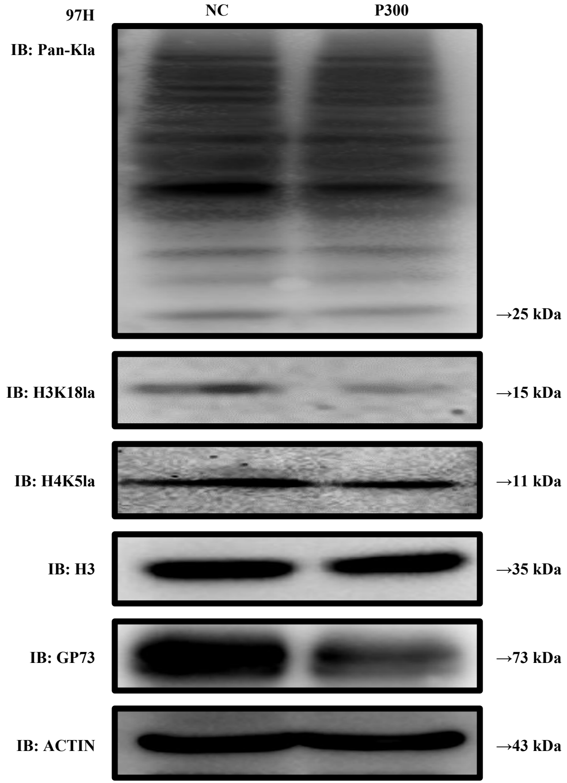

Figure 6E

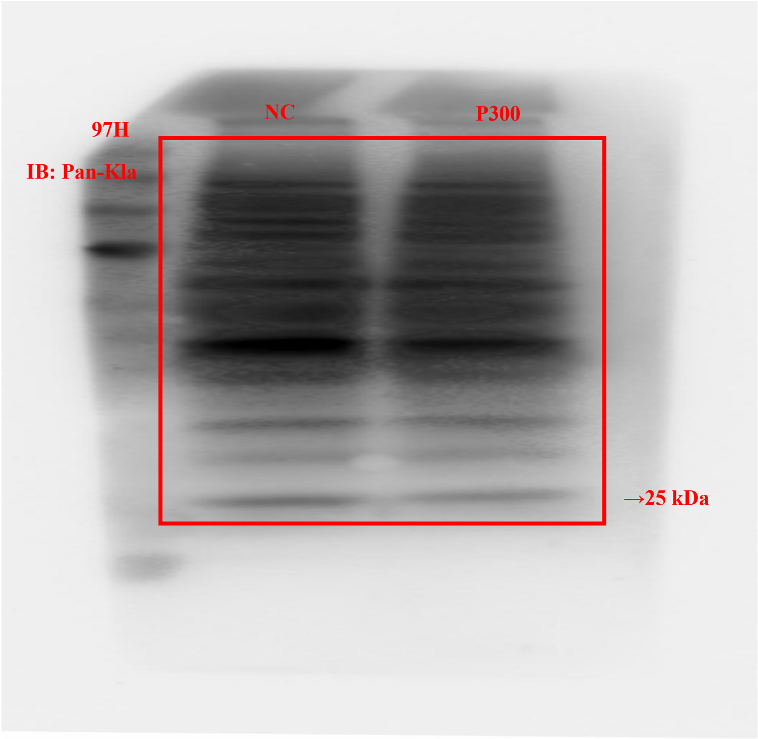

Figure 6E

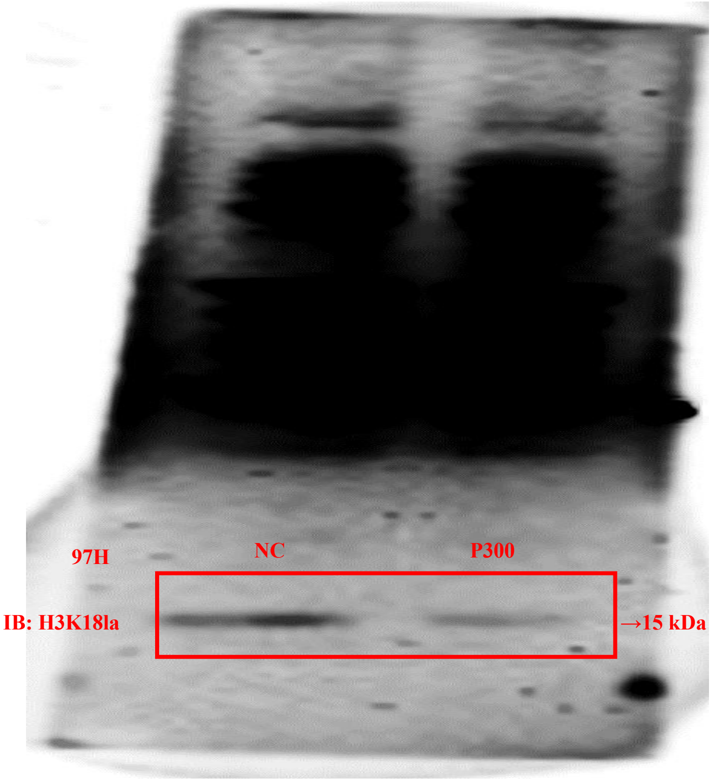

Figure 6E

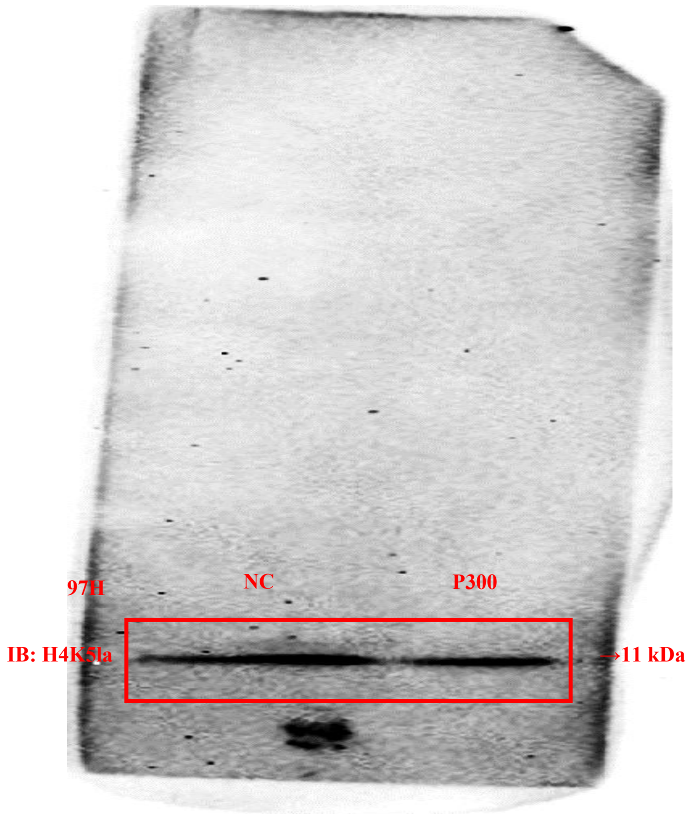

Figure 6E

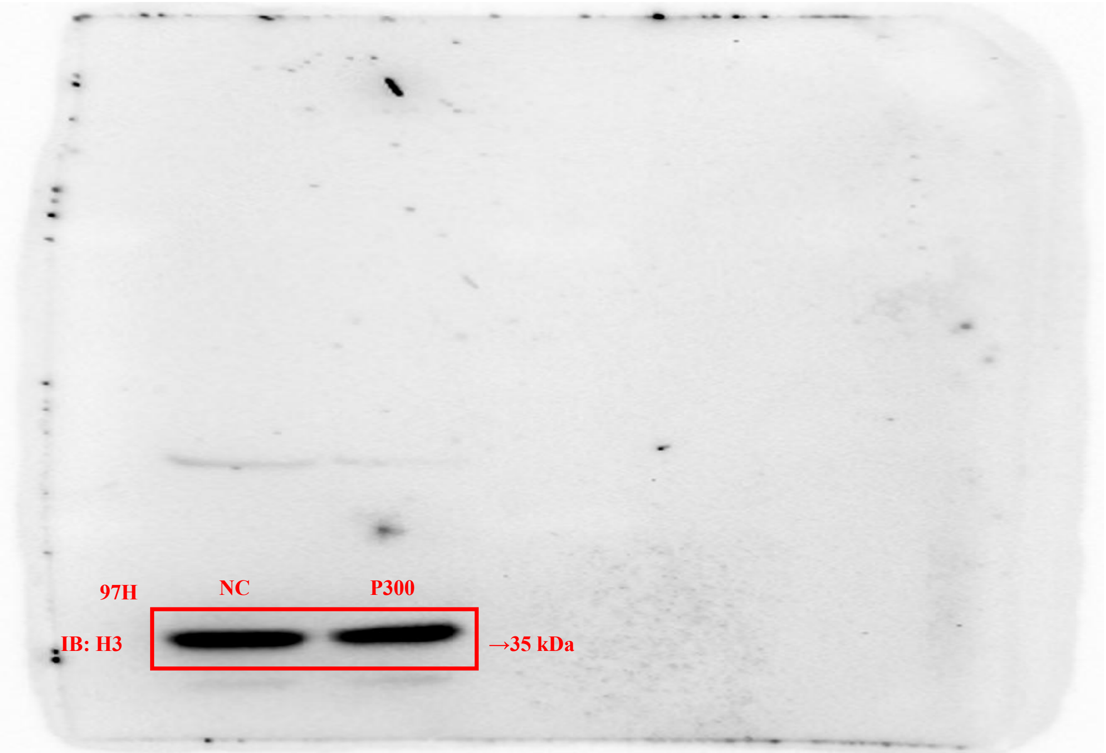

Figure 6E

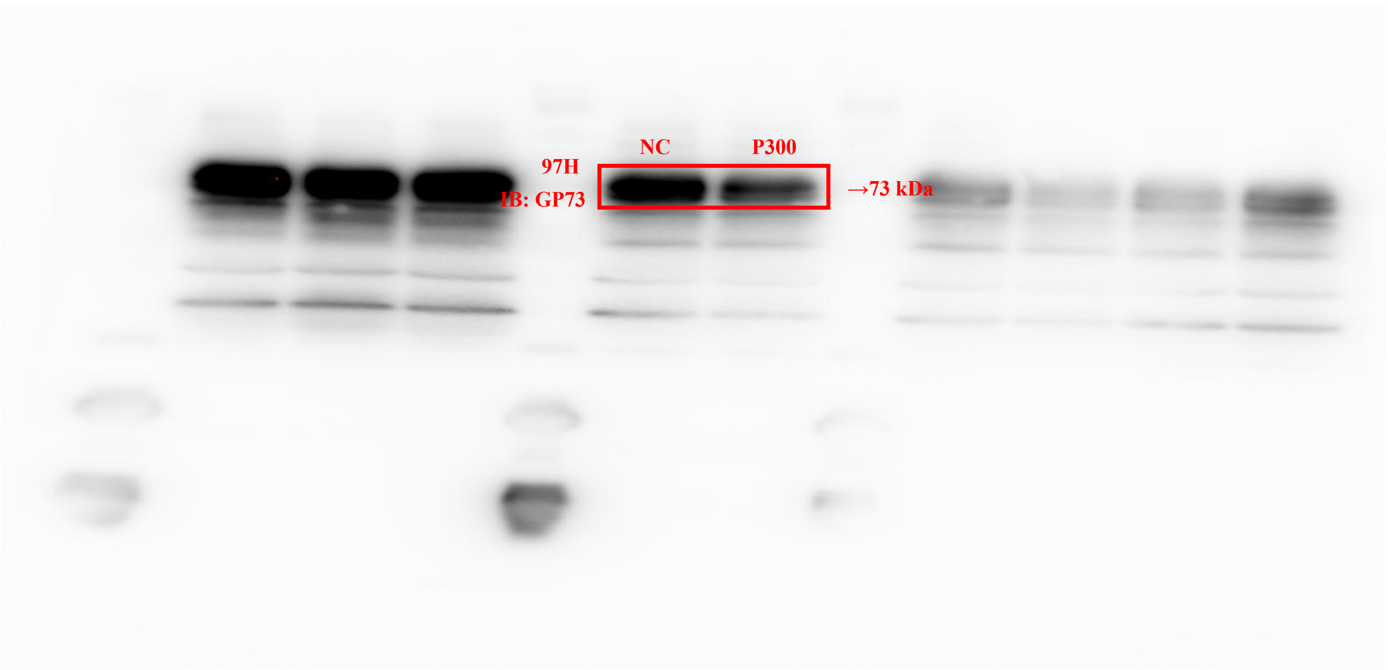

Figure 6E

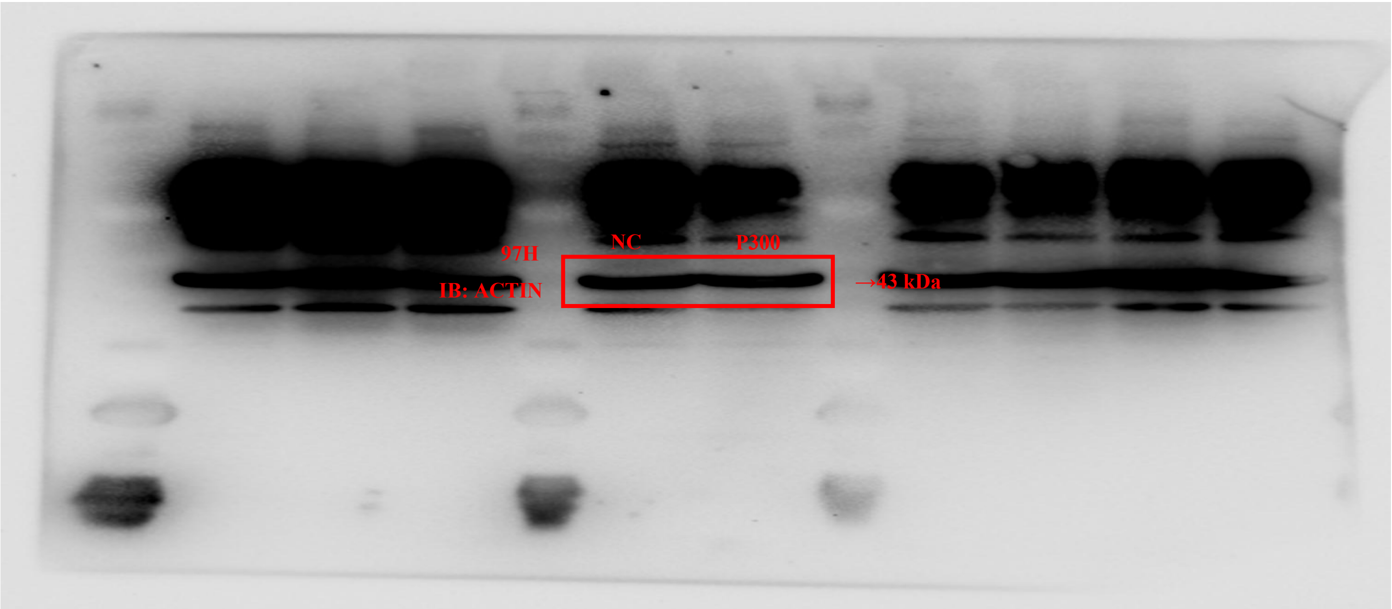

Figure 7A

siRNA-3 with the highest c-Myc knockdown efficiency was selected for follow-up experiments to detect and verify the changes of GP73 protein in MHCC97H HCC cells

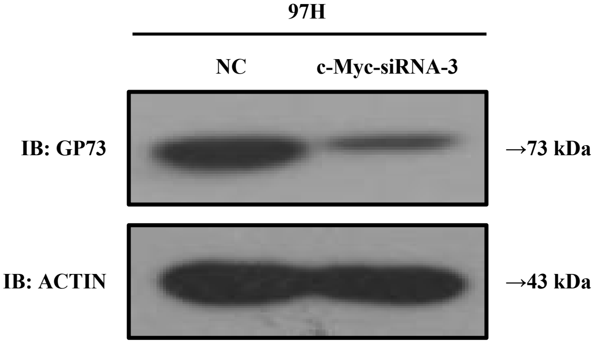

Figure 7A

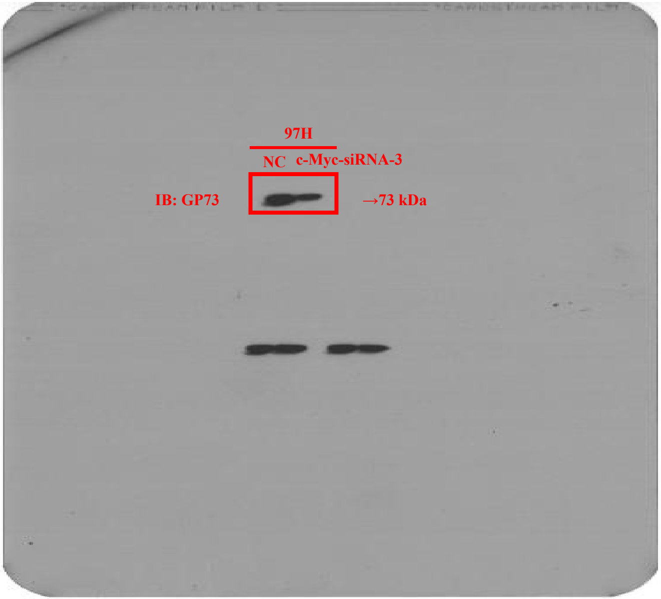
97H
NC
c-Myc-siRNA-3
IB: GP73
→73 kDa

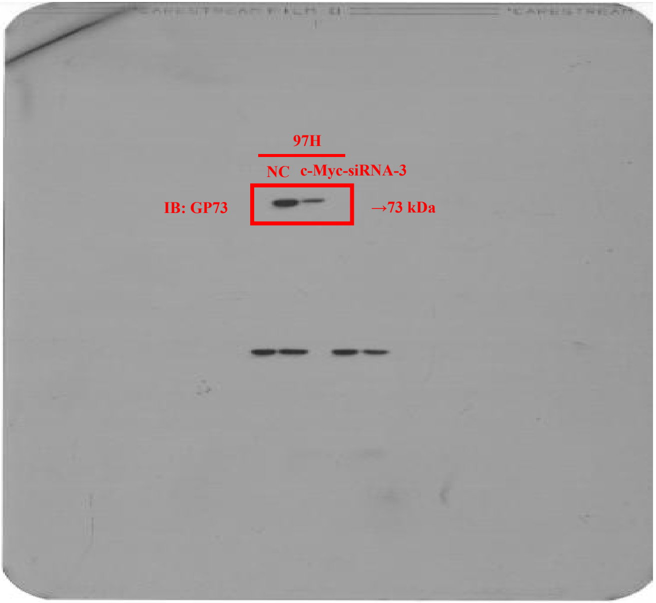
97H
NC
c-Myc-siRNA-3
IB: GP73
→73 kDa

Figure 7A

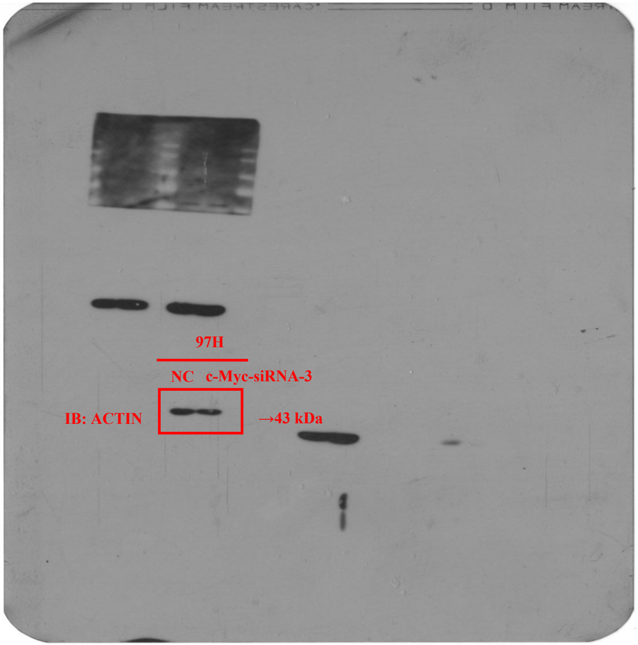
97H
NC c-Myc-siRNA-3
IB: ACTIN
→43 kDa

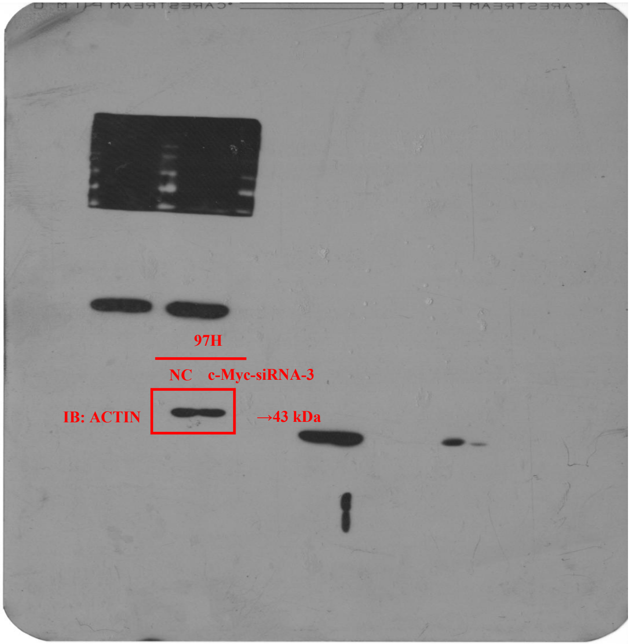
97H
NC c-Myc-siRNA-3
IB: ACTIN
→43 kDa

Figure 7B

Western blot detection of the expressions of GP73 and key genes in the JAK2/STAT3 pathway in MHCC97H-c-Myc-NC, MHCC97H-c-Myc-OE and MHCC97H-c-Myc-OE-C188-9 cells. C188-9 indicates MHCC97H-c-Myc-OE cells were treated with C188-9 (10uM) for 24 hours

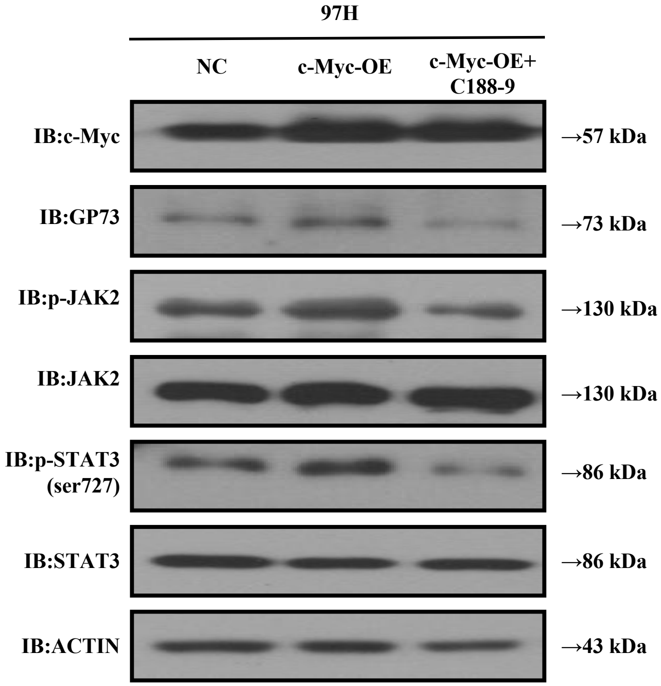

Figure 7B

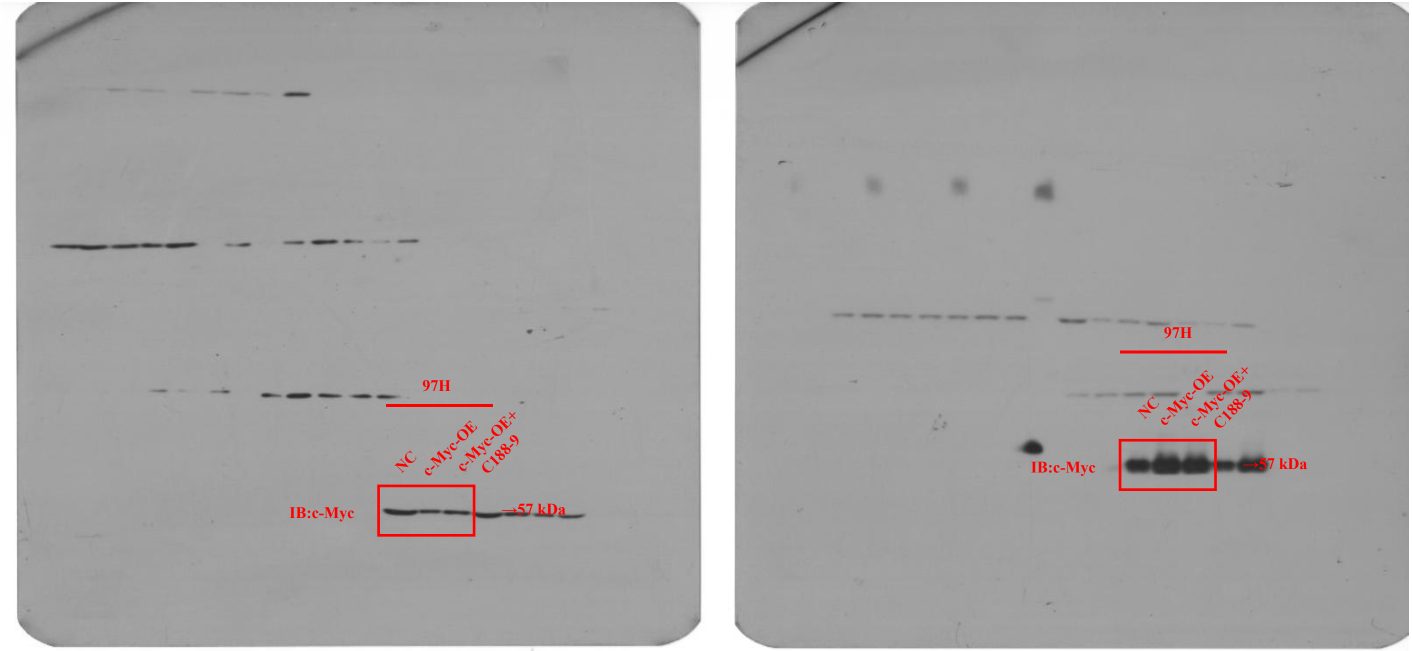
97H
NC
c-Myc-OE
c-Myc-OE+
C188-9
IB:c-Myc
→57 kDa
97H
NC
c-Myc-OE
c-Myc-OE+
C188-9
IB:c-Myc
→57 kDa

Figure 7B

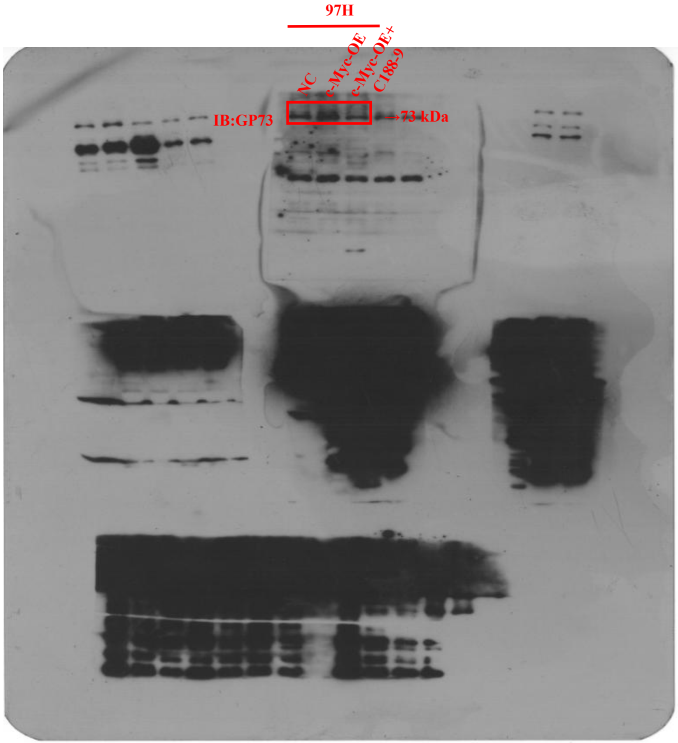
97H
NC
c-Myc-OE
c-Myc-OE+
C188-9
IB:GP73
→73 kDa

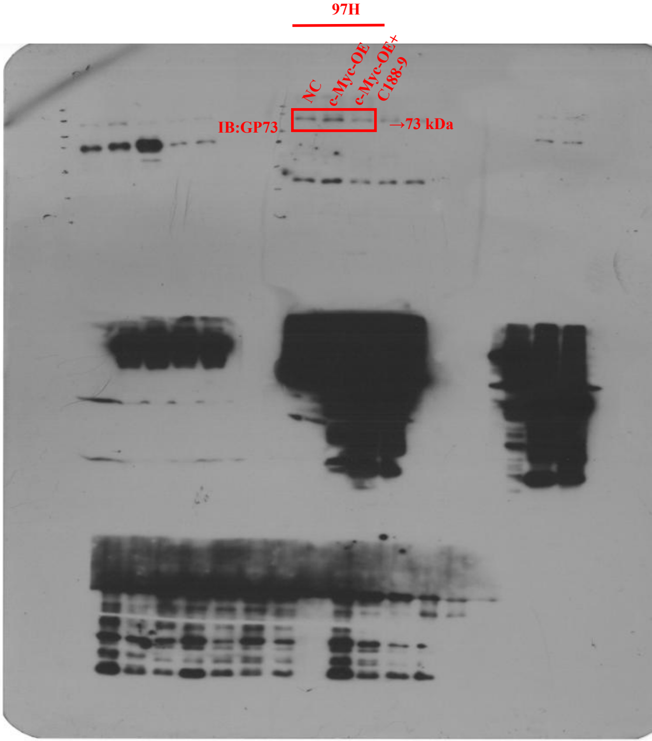
97H
NC
c-Myc-OE
c-Myc-OE+
C188-9
IB:GP73
→73 kDa

Figure 7B

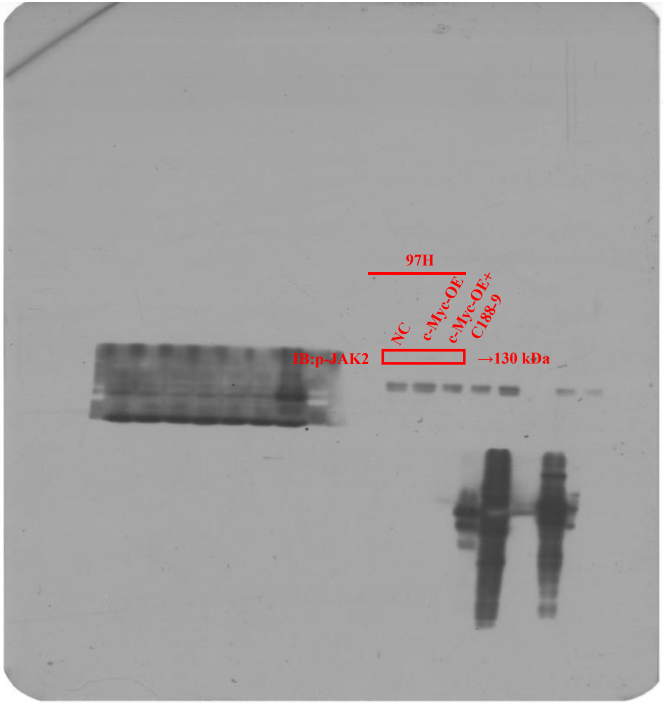
97H
NC
c-Myc-OE
c-Myc-OE+ C188-9
IB:p-JAK2
→130 kDa

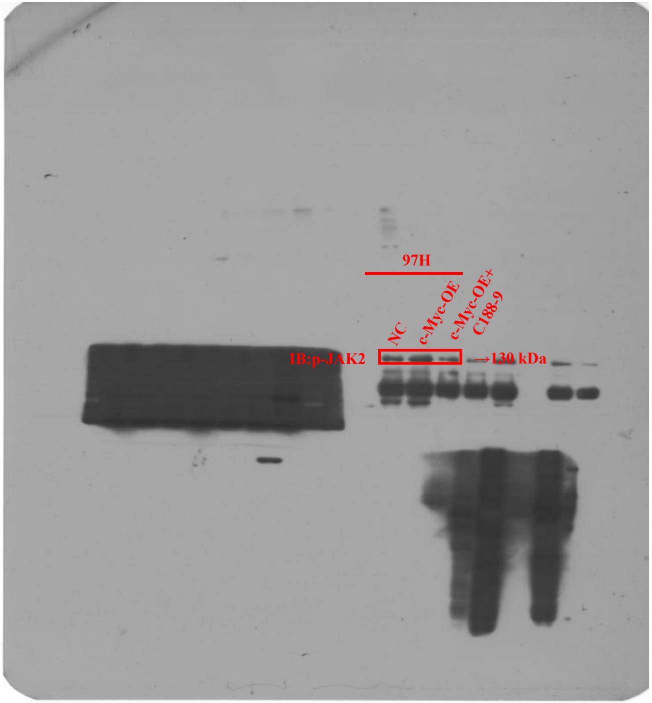
97H
NC
c-Myc-OE
c-Myc-OE+ C188-9
IB:p-JAK2
→130 kDa

Figure 7B

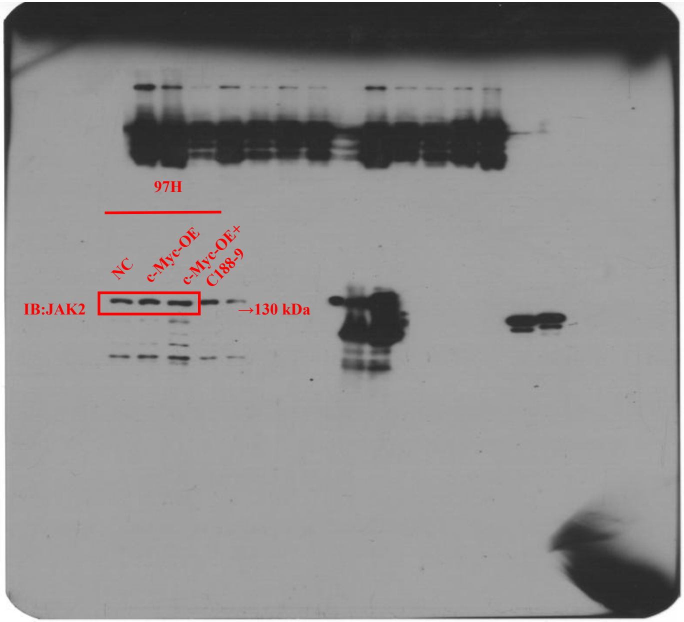

97H
NC
c-Myc-OE
c-Myc-OE+
C188-9
IB:JAK2
→130 kDa

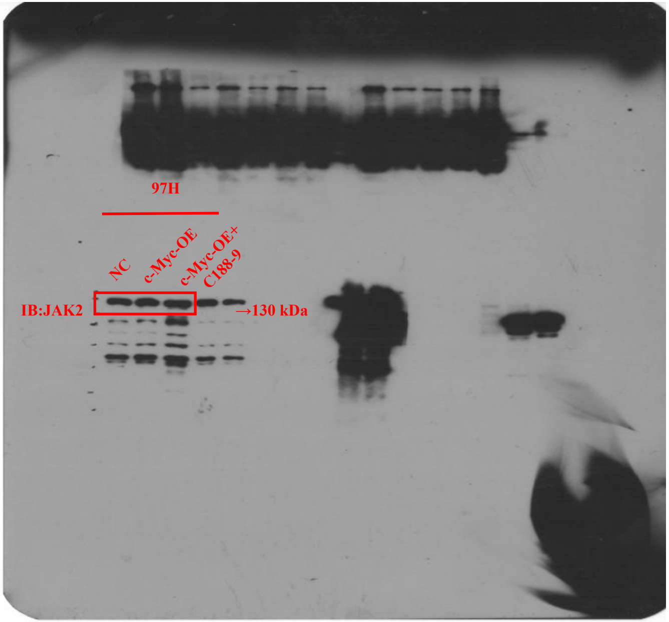

97H
NC
c-Myc-OE
c-Myc-OE+
C188-9
IB:JAK2
→130 kDa

Figure 7B

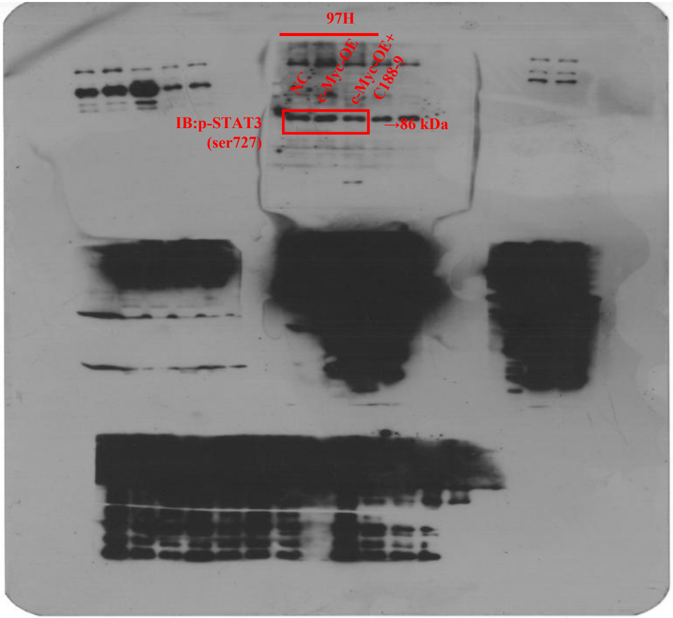
97H
NC
c-Myc-OE
c-Myc-OE+
C188-9
IB:p-STAT3
(ser727)
→86 kDa

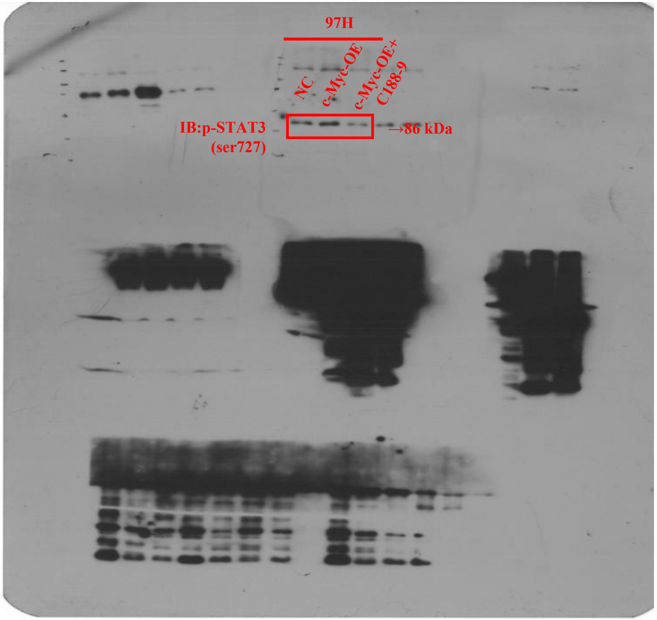
97H
NC
c-Myc-OE
c-Myc-OE+
C188-9
IB:p-STAT3
(ser727)
→86 kDa

Figure 7B

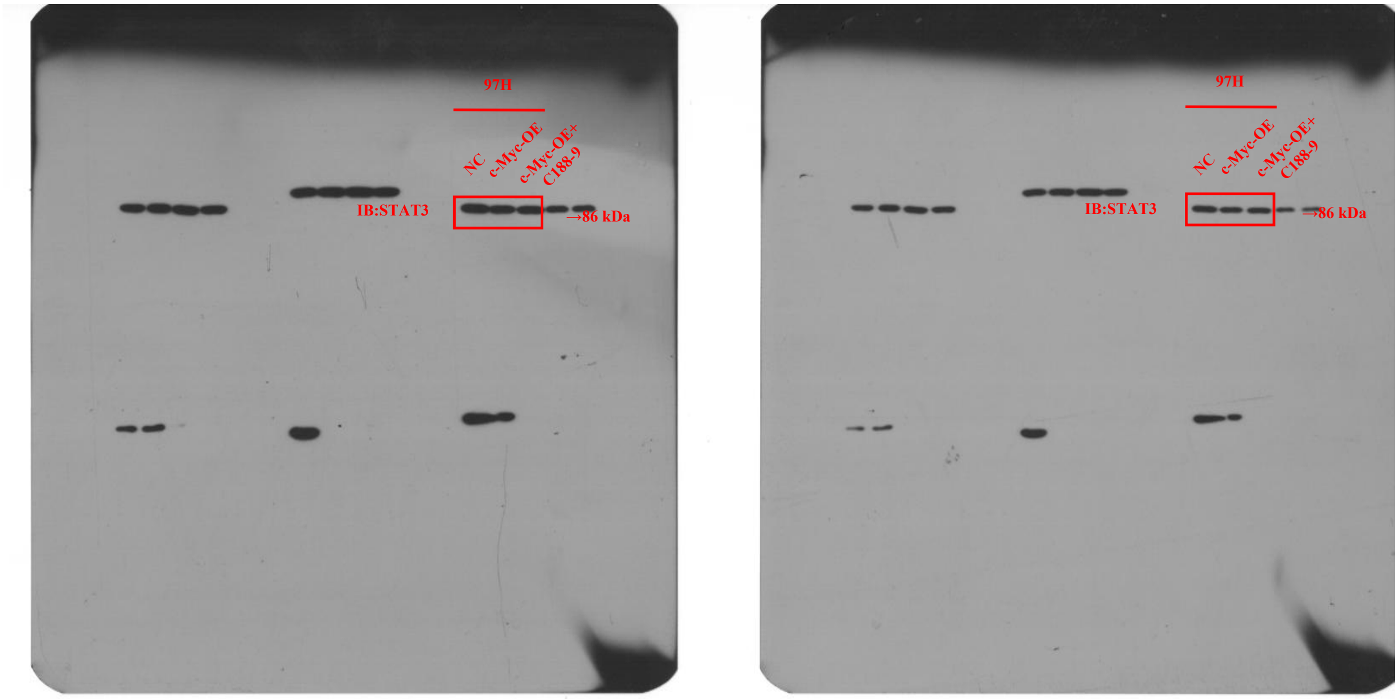

97H
NC
c-Myc-OE
c-Myc-OE+ C188-9
IB:STAT3
→86 kDa
97H
NC
c-Myc-OE
c-Myc-OE+ C188-9
IB:STAT3
→86 kDa

Figure 7B

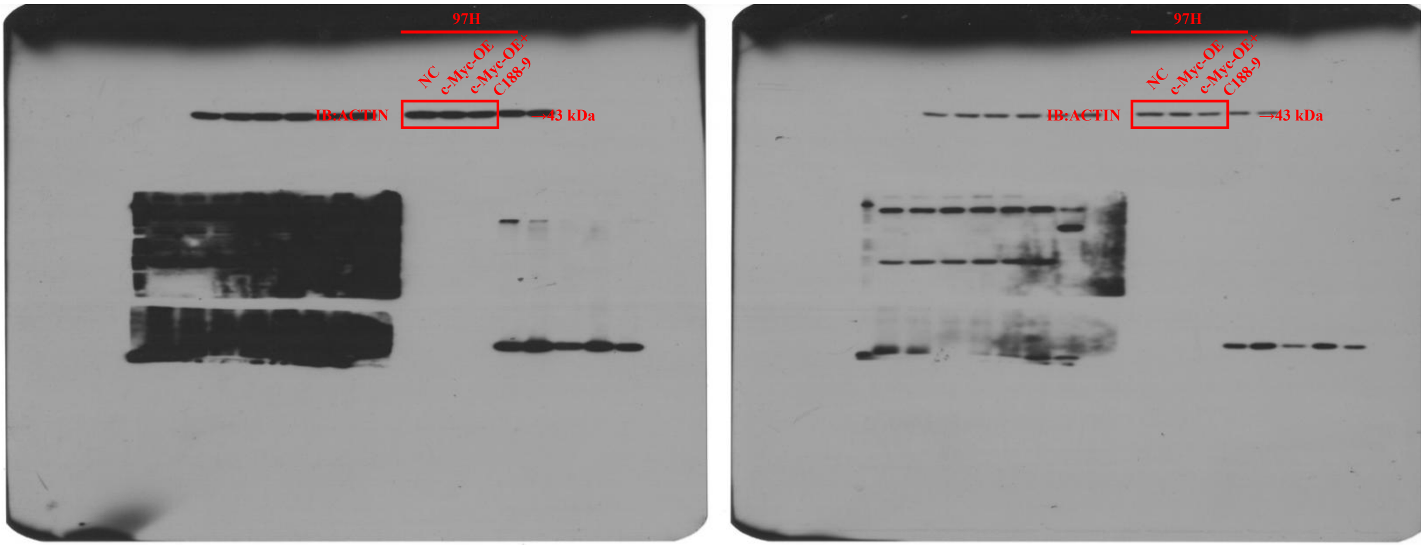
97H
NC
c-Myc-OE
c-Myc-OE+
C188-9
IB:ACTIN
→43 kDa
97H
NC
c-Myc-OE
c-Myc-OE+
C188-9
IB:ACTIN
→43 kDa

Figure 7L

Western blot detection determined the impact of modulation of c-Myc and C188-9 on the alteration of expressions of GP73, c-Myc and key genes in JAK2/STAT3 pathway in MHCC97H-GP73-KD and Hep3B-GP73-OE cells.

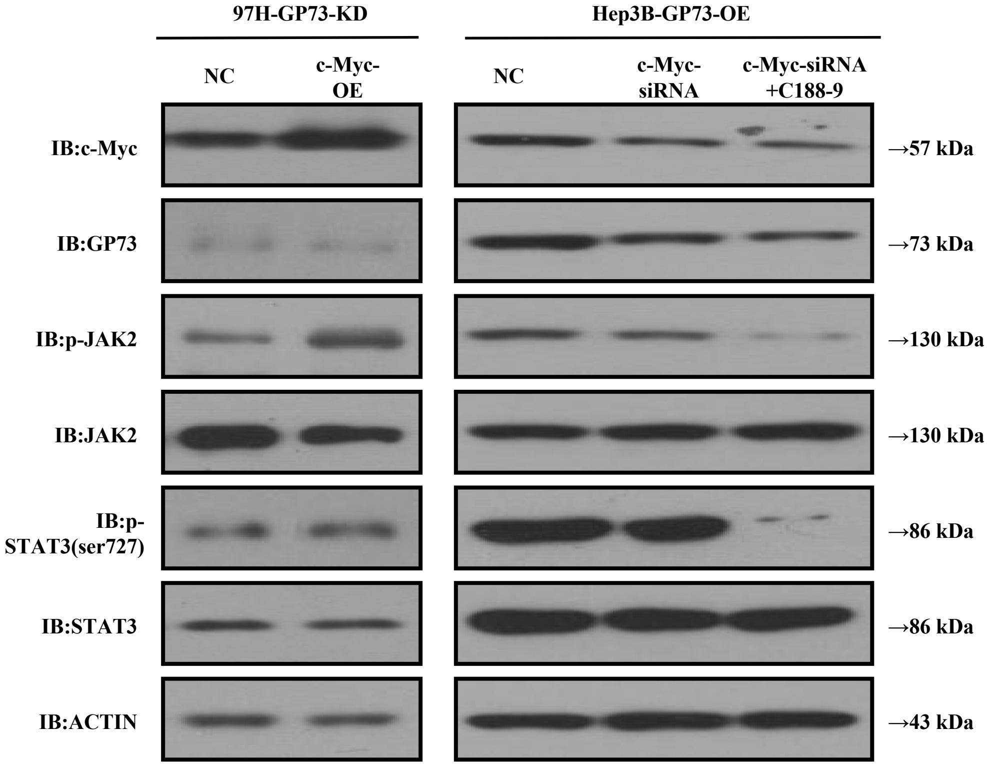

Figure 7L

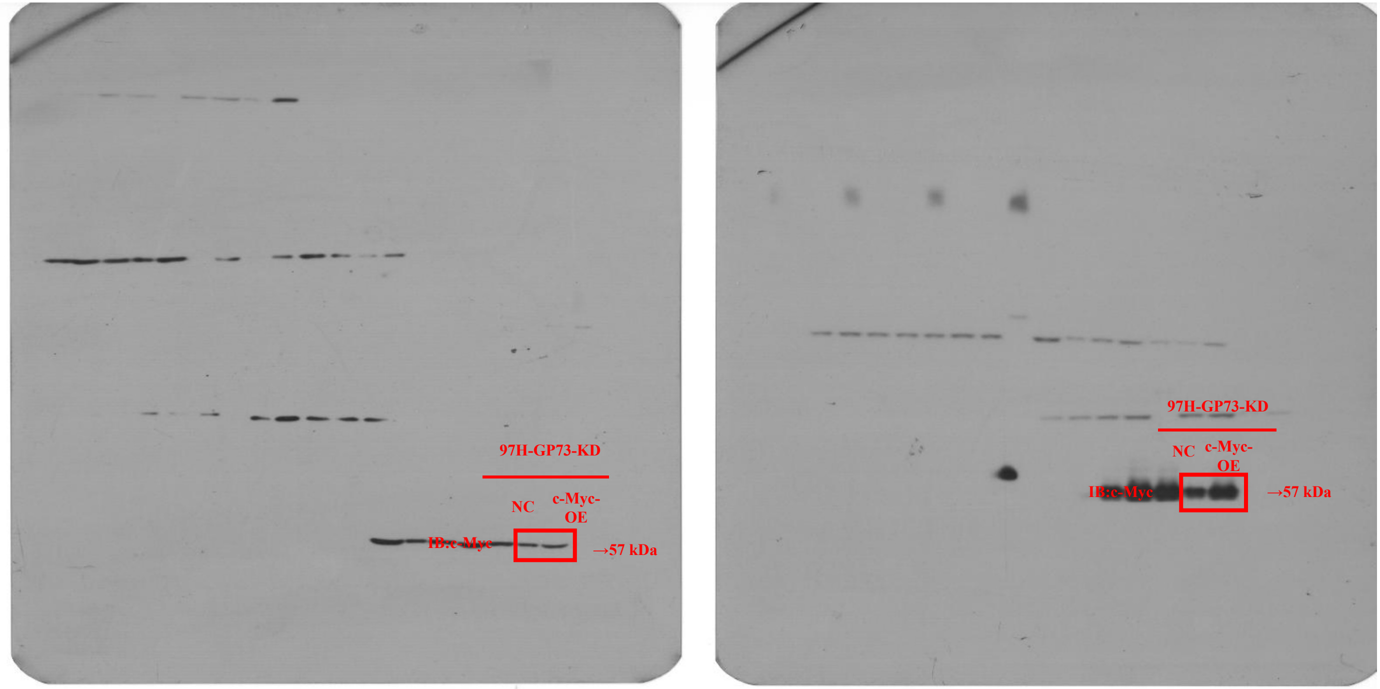
97H-GP73-KD
NC
c-Myc-OE
IB:c-Myc
→57 kDa
97H-GP73-KD
NC
c-Myc-OE
IB:c-Myc
→57 kDa

Figure 7L

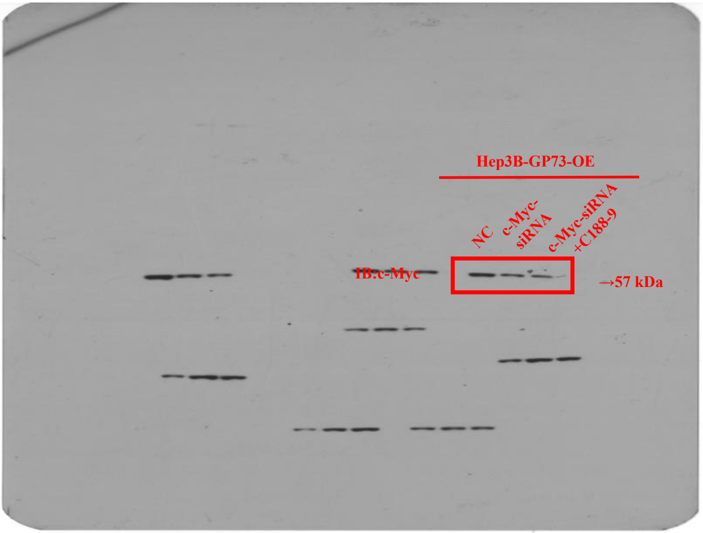
Hep3B-GP73-OE
NC
c-Myc-siRNA
c-Myc-siRNA +C188-9
IB:c-Myc
→57 kDa

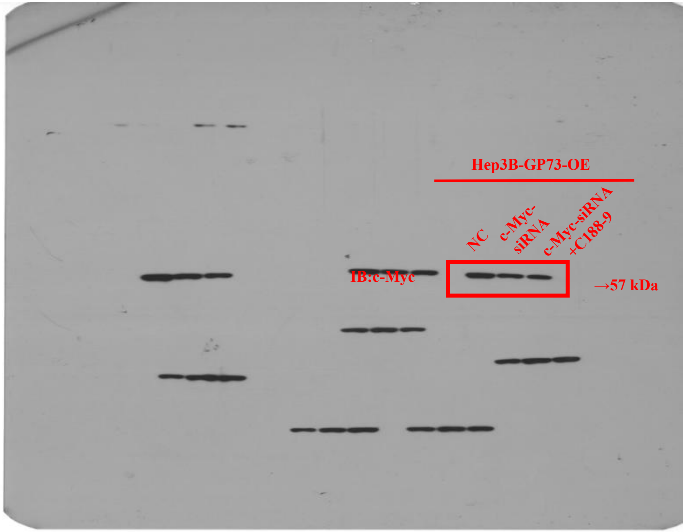
Hep3B-GP73-OE
NC
c-Myc-siRNA
c-Myc-siRNA +C188-9
IB:c-Myc
→57 kDa

Figure 7L

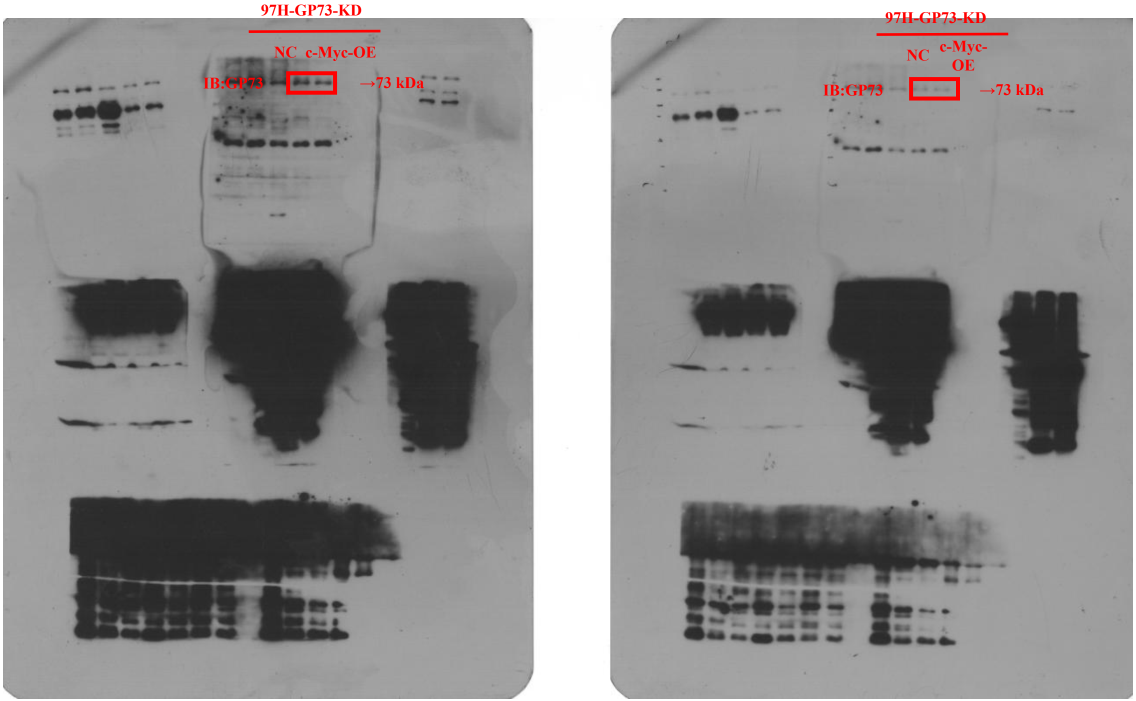
97H-GP73-KD
NC
c-Myc-OE
IB:GP73
→73 kDa
97H-GP73-KD
NC
c-Myc-OE
IB:GP73
→73 kDa

Figure 7L

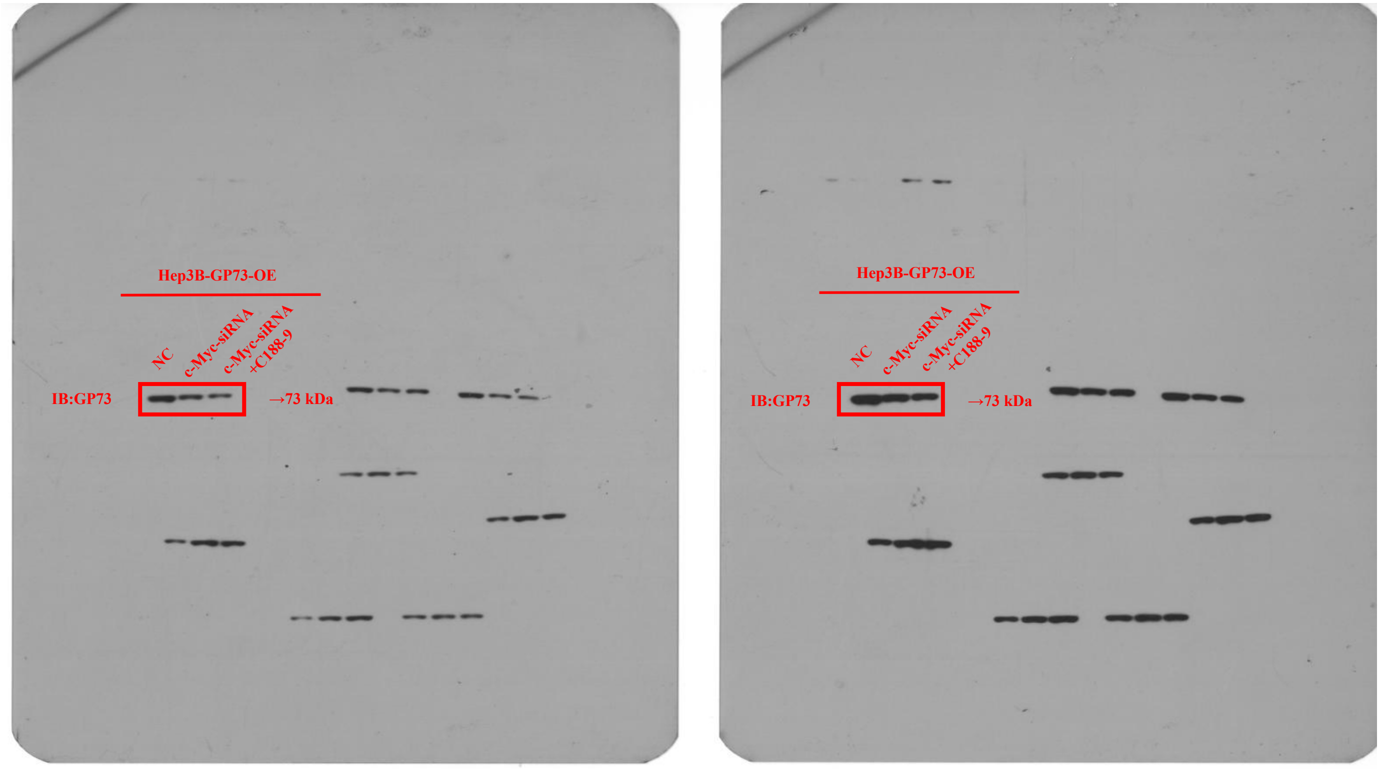
Hep3B-GP73-OE
NC
c-Myc-siRNA
c-Myc-siRNA +C188-9
IB:GP73
→73 kDa
Hep3B-GP73-OE
NC
c-Myc-siRNA
c-Myc-siRNA +C188-9
IB:GP73
→73 kDa

Figure 7L

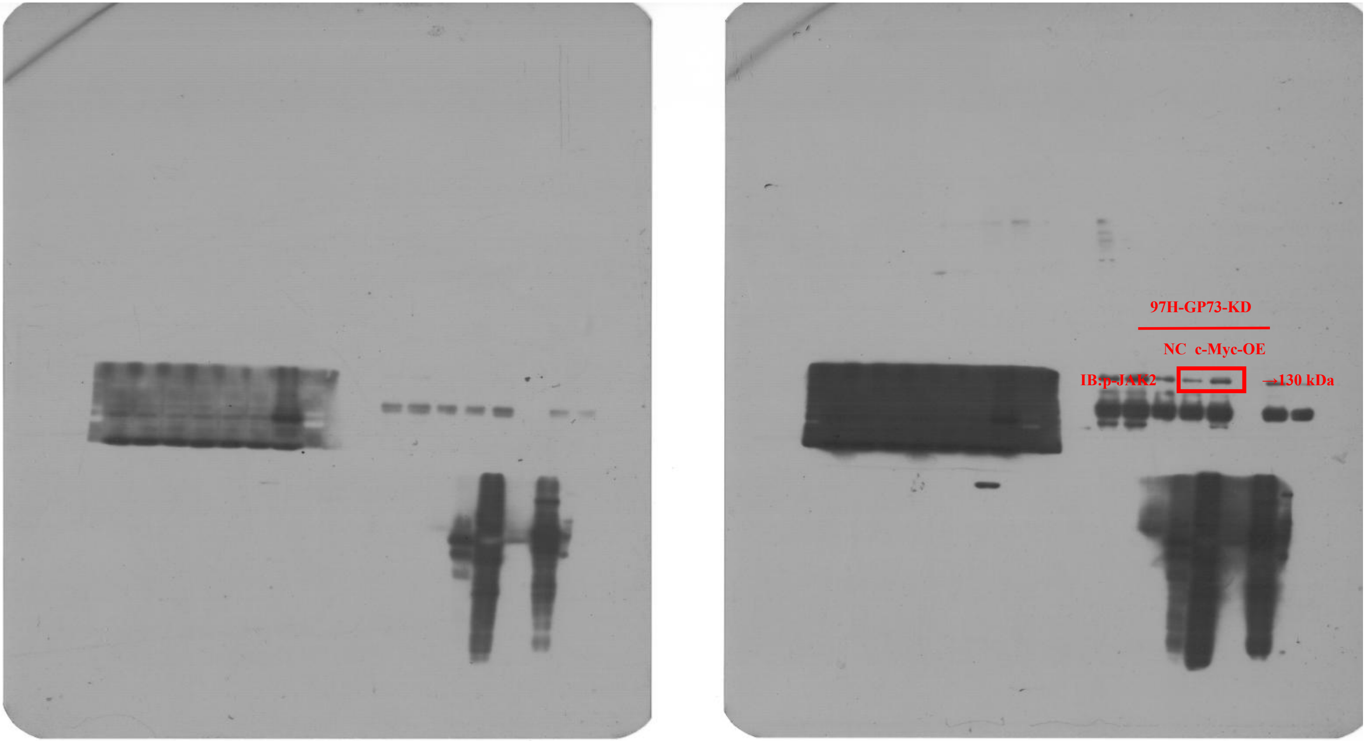

Figure 7L

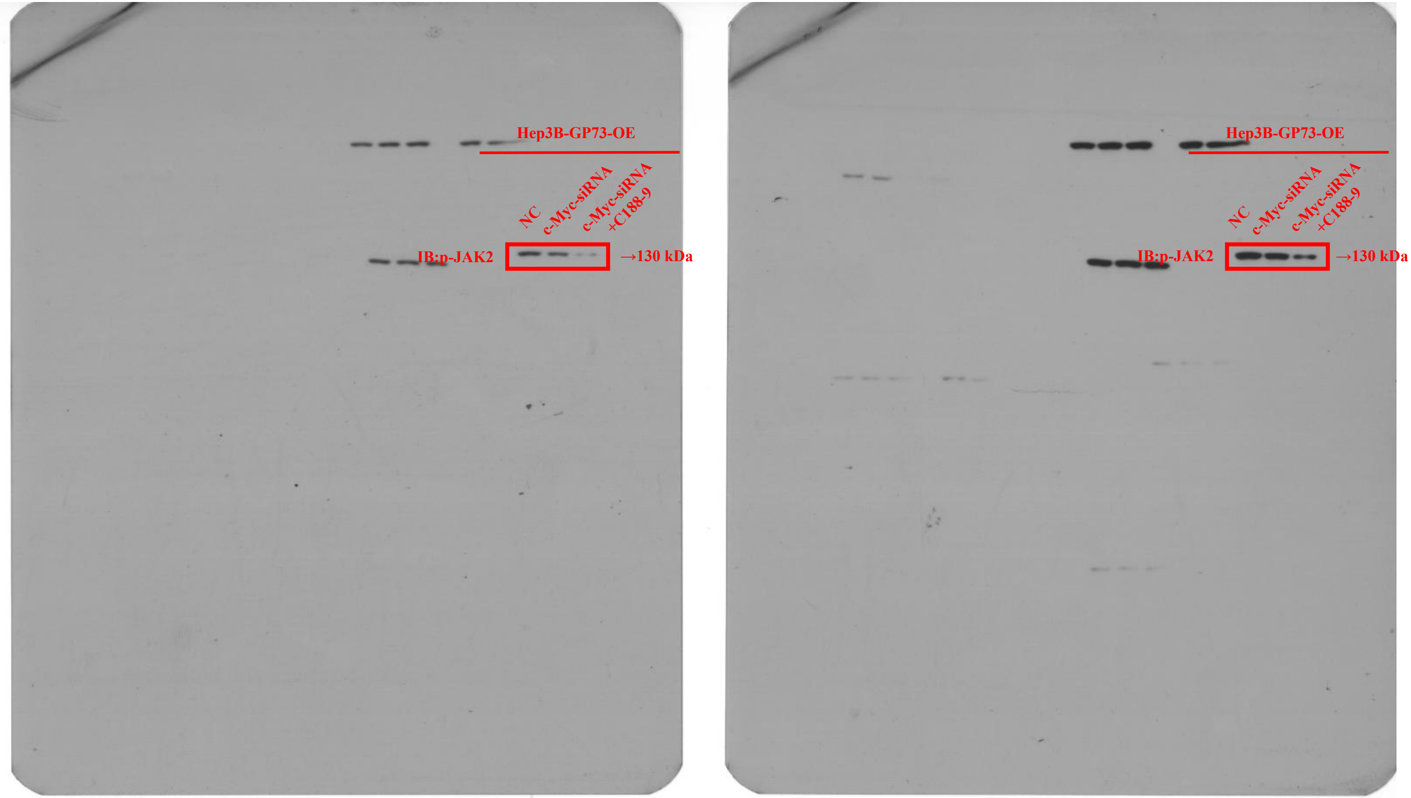
Hep3B-GP73-OE
NC
c-Myc-siRNA
c-Myc-siRNA +C188-9
IB:p-JAK2
→130 kDa
Hep3B-GP73-OE
NC
c-Myc-siRNA
c-Myc-siRNA +C188-9
IB:p-JAK2
→130 kDa

Figure 7L

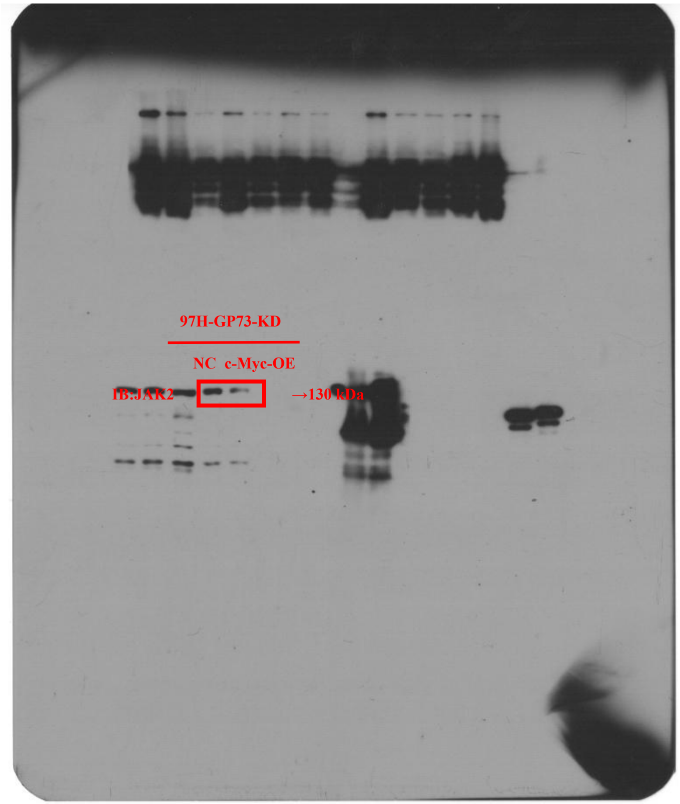

97H-GP73-KD
NC c-Myc-OE
IB:JAK2
→130 kDa

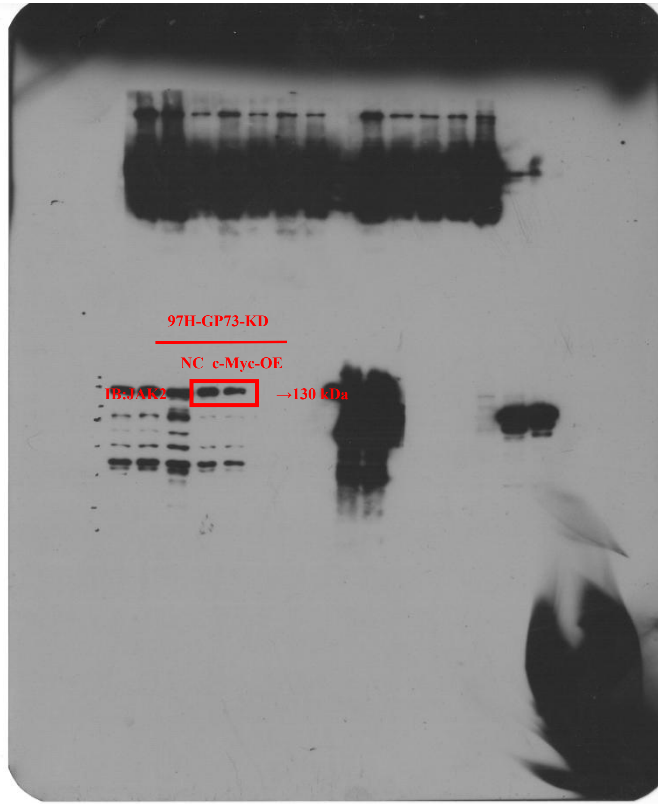

97H-GP73-KD
NC c-Myc-OE
IB:JAK2
→130 kDa

Figure 7L

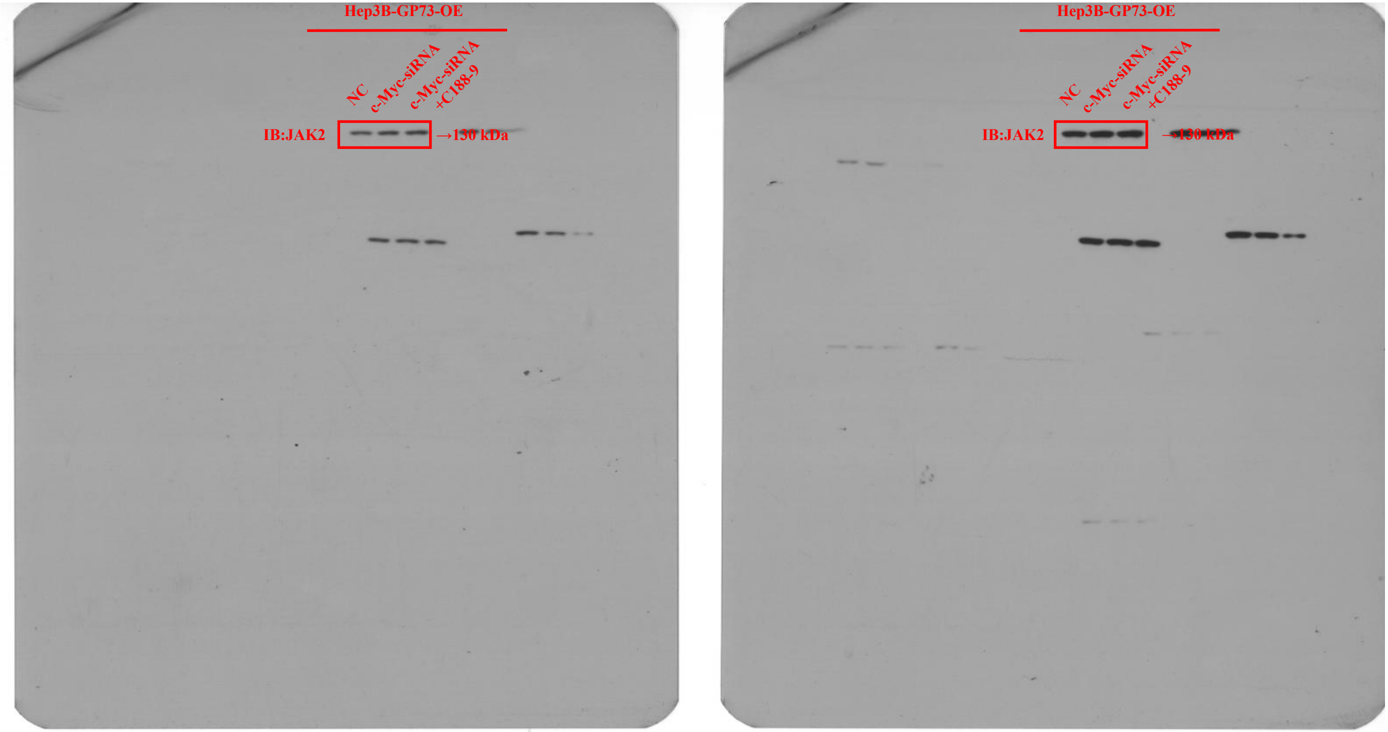
Hep3B-GP73-OE
NC
c-Myc-siRNA
c-Myc-siRNA
+C188-9
IB:JAK2
→130 kDa
Hep3B-GP73-OE
NC
c-Myc-siRNA
c-Myc-siRNA
+C188-9
IB:JAK2
→130 kDa

Figure 7L

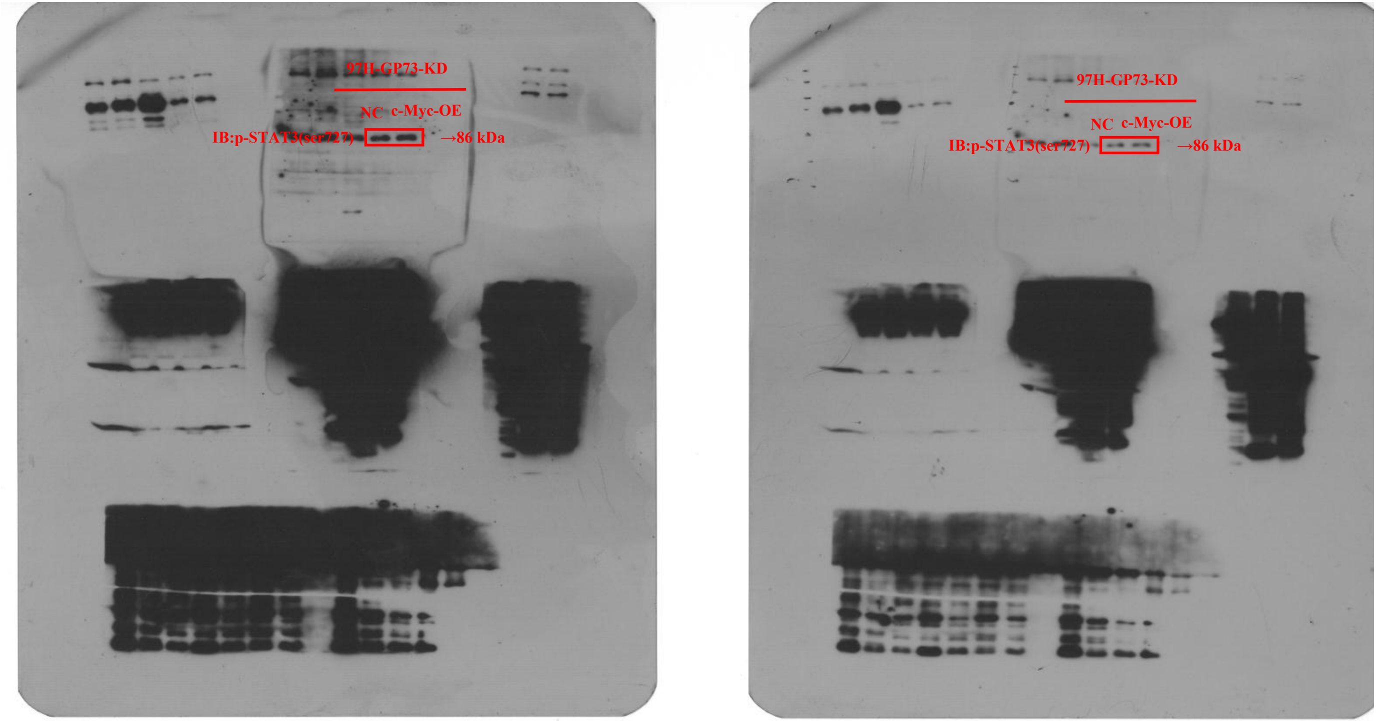
97H-GP73-KD
NC c-Myc-OE
IB:p-STAT3(ser727)
→86 kDa
97H-GP73-KD
NC c-Myc-OE
IB:p-STAT3(ser727)
→86 kDa

Figure 7L

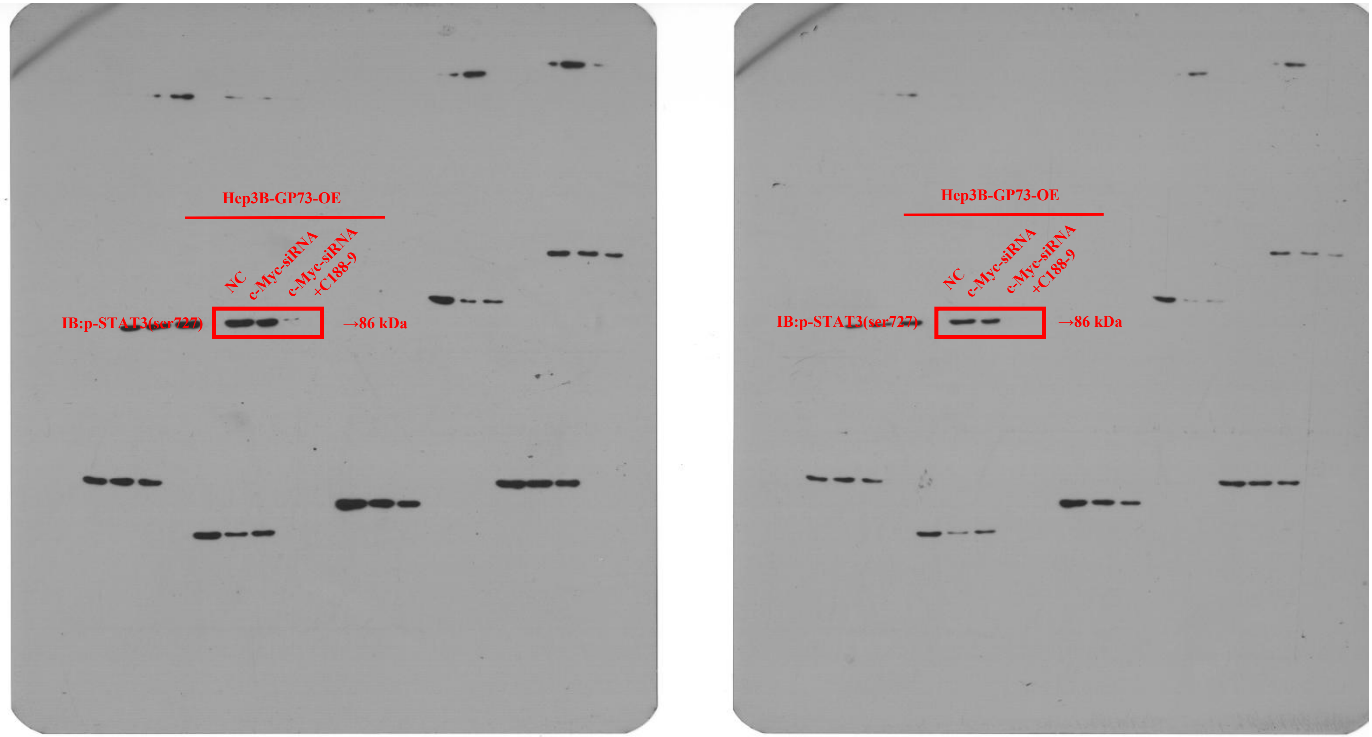

Hep3B-GP73-OE
NC
c-Myc-siRNA
c-Myc-siRNA +C188-9
IB:p-STAT3(ser727)
→86 kDa
Hep3B-GP73-OE
NC
c-Myc-siRNA
c-Myc-siRNA +C188-9
IB:p-STAT3(ser727)
→86 kDa

Figure 7L

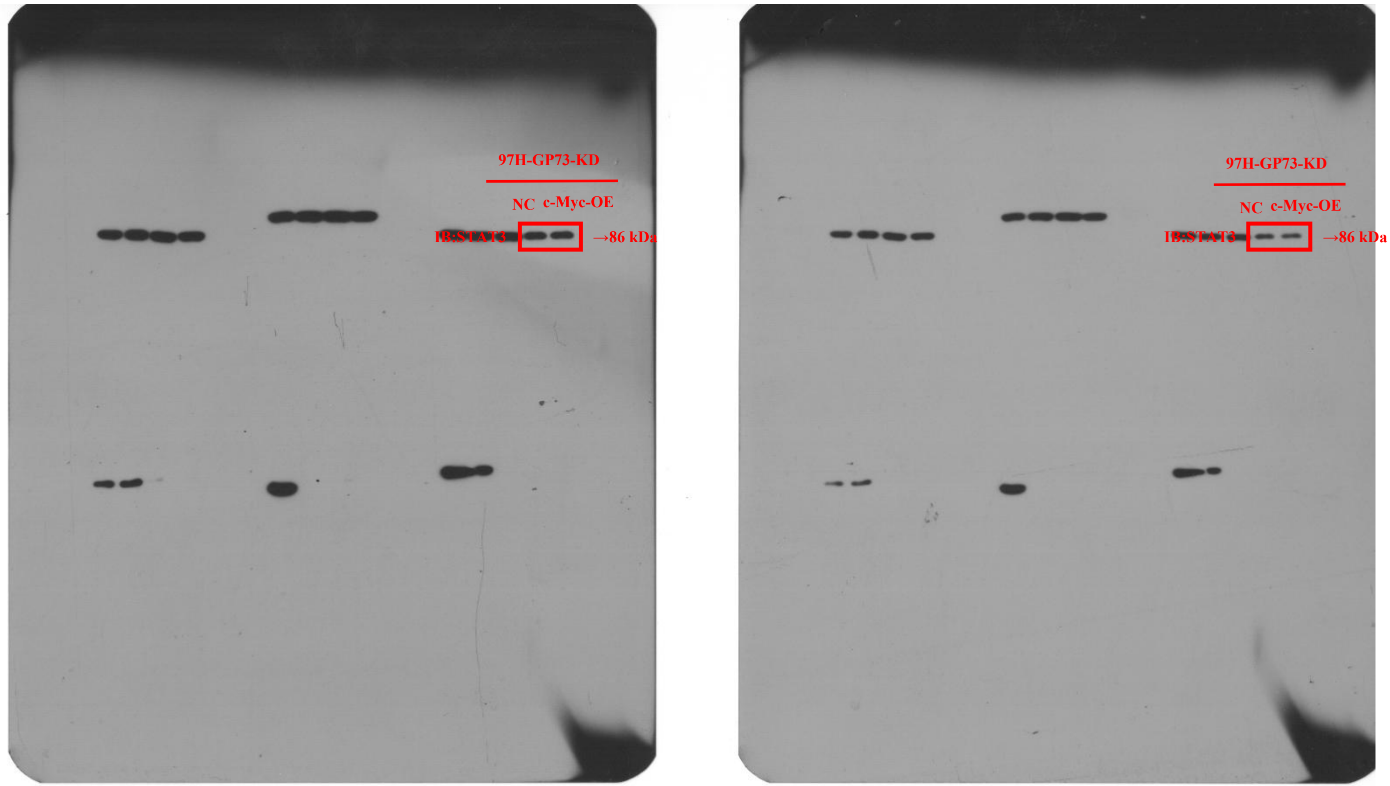
97H-GP73-KD
NC c-Myc-OE
IB:STAT3
→86 kDa
97H-GP73-KD
NC c-Myc-OE
IB:STAT3
→86 kDa

Figure 7L

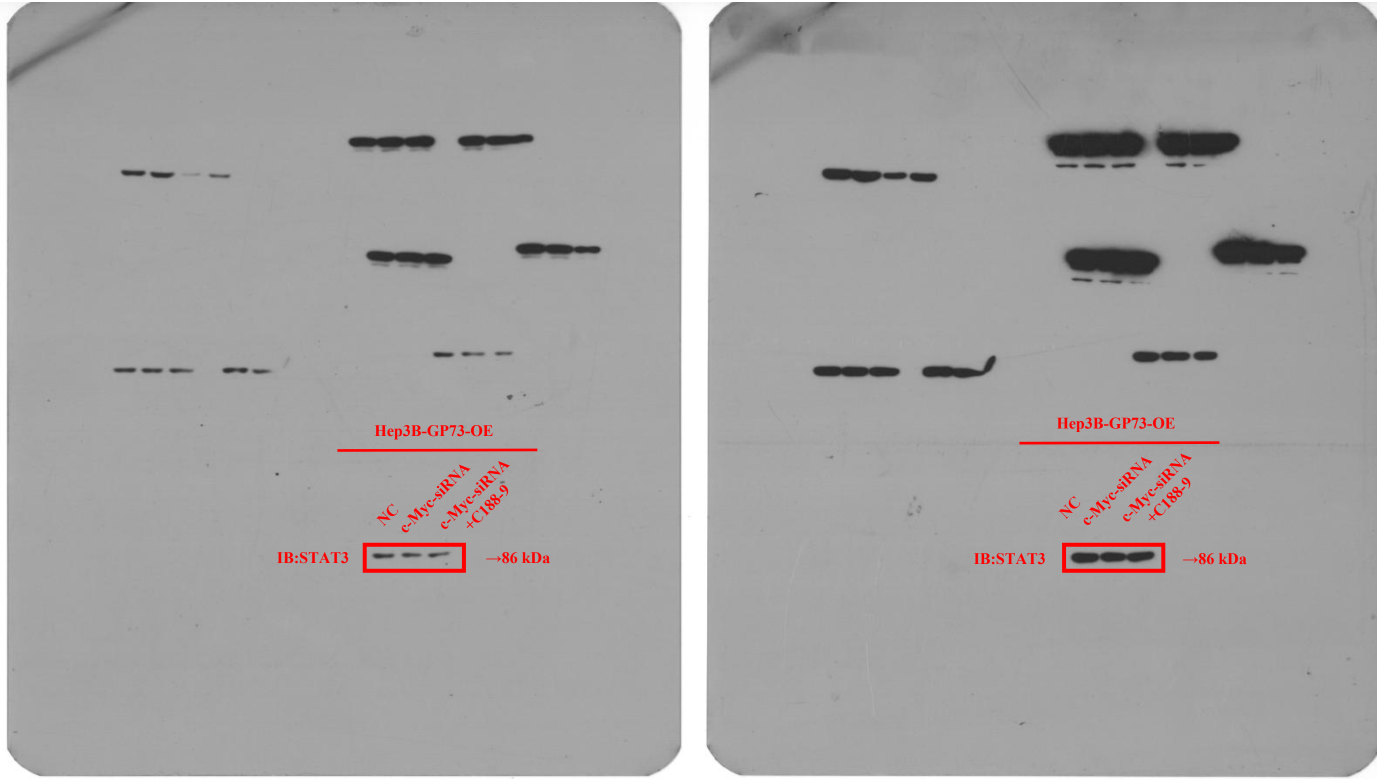
Hep3B-GP73-OE
NC
c-Myc-siRNA
c-Myc-siRNA +C188-9
IB:STAT3
→86 kDa
Hep3B-GP73-OE
NC
c-Myc-siRNA
c-Myc-siRNA +C188-9
IB:STAT3
→86 kDa

Figure 7L

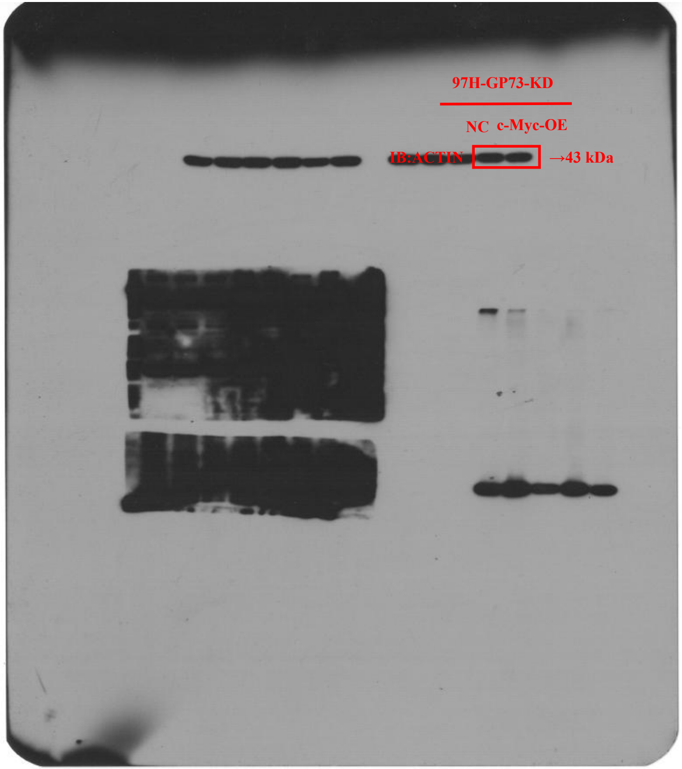

97H-GP73-KD
NC
c-Myc-OE
IB:ACTIN
→43 kDa

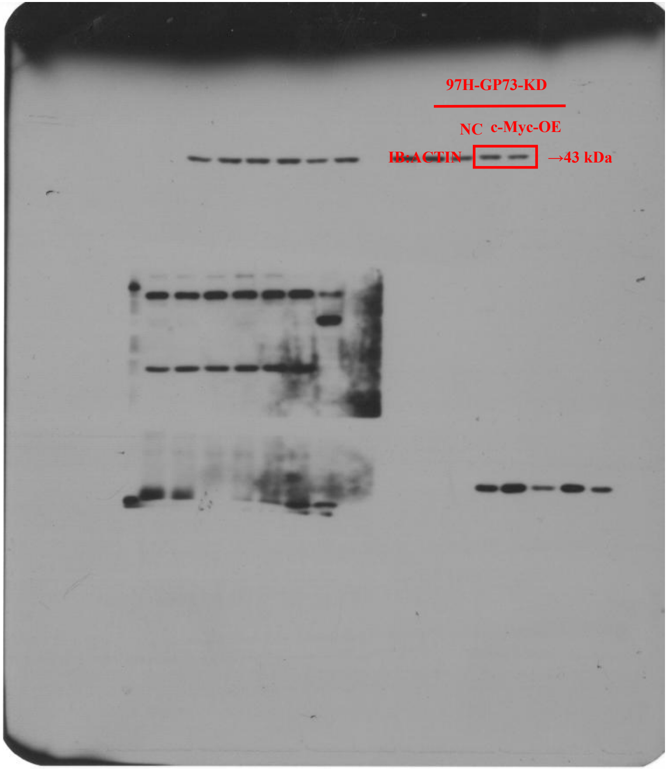

97H-GP73-KD
NC
c-Myc-OE
IB:ACTIN
→43 kDa

Figure 7L

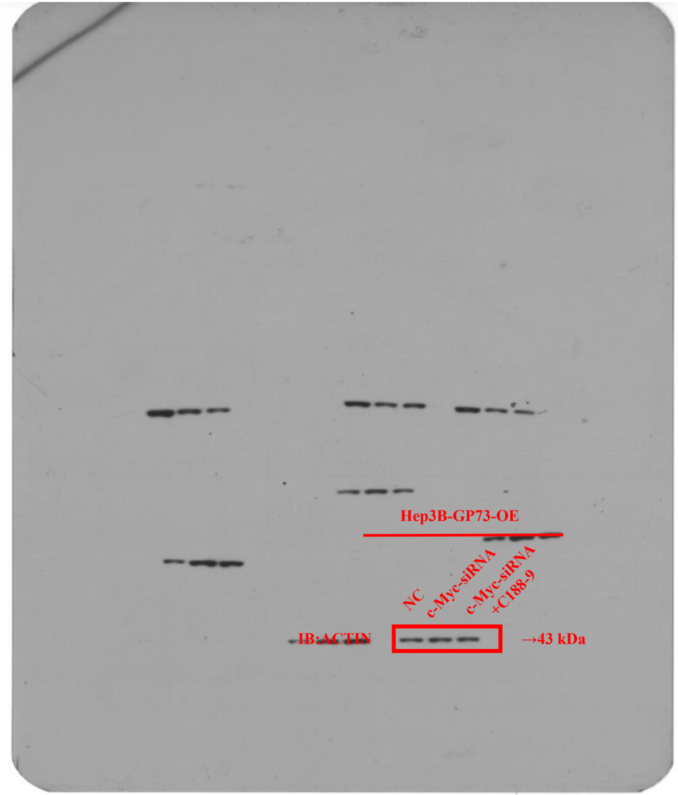

Hep3B-GP73-OE
NC
c-Myc-siRNA
c-Myc-siRNA +C188-9
IB:ACTIN
→43 kDa

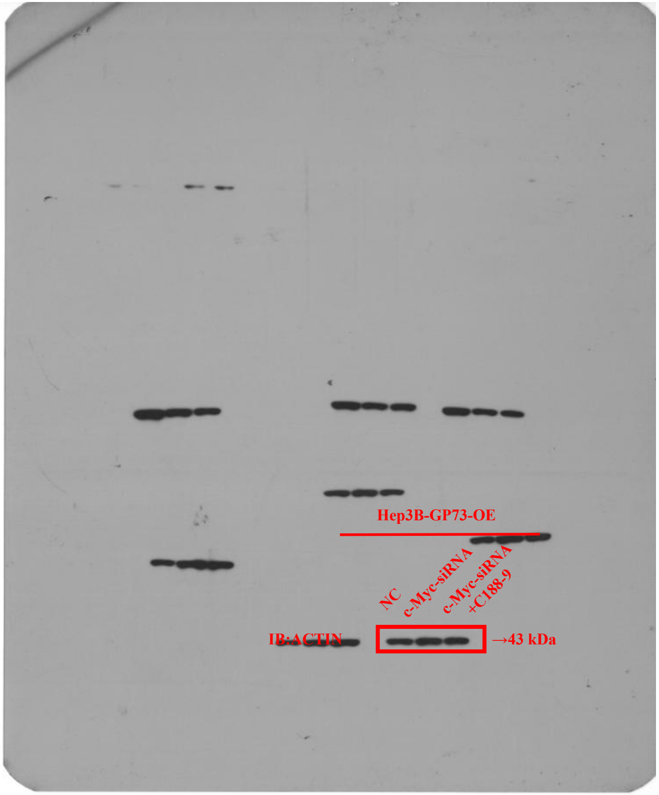

Hep3B-GP73-OE
NC
c-Myc-siRNA
c-Myc-siRNA +C188-9
IB:ACTIN
→43 kDa

Supplement Figure 3B

Western blot detection for evaluating the efficiency of knocked down GP73 in MHCC97H cells and over-expressed GP73 in Hep3B cells

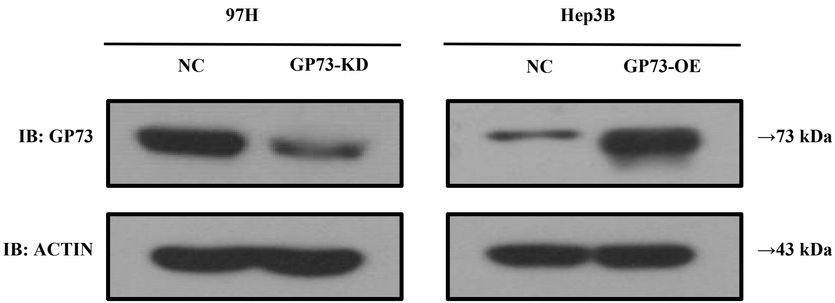

Supplement Figure 3B

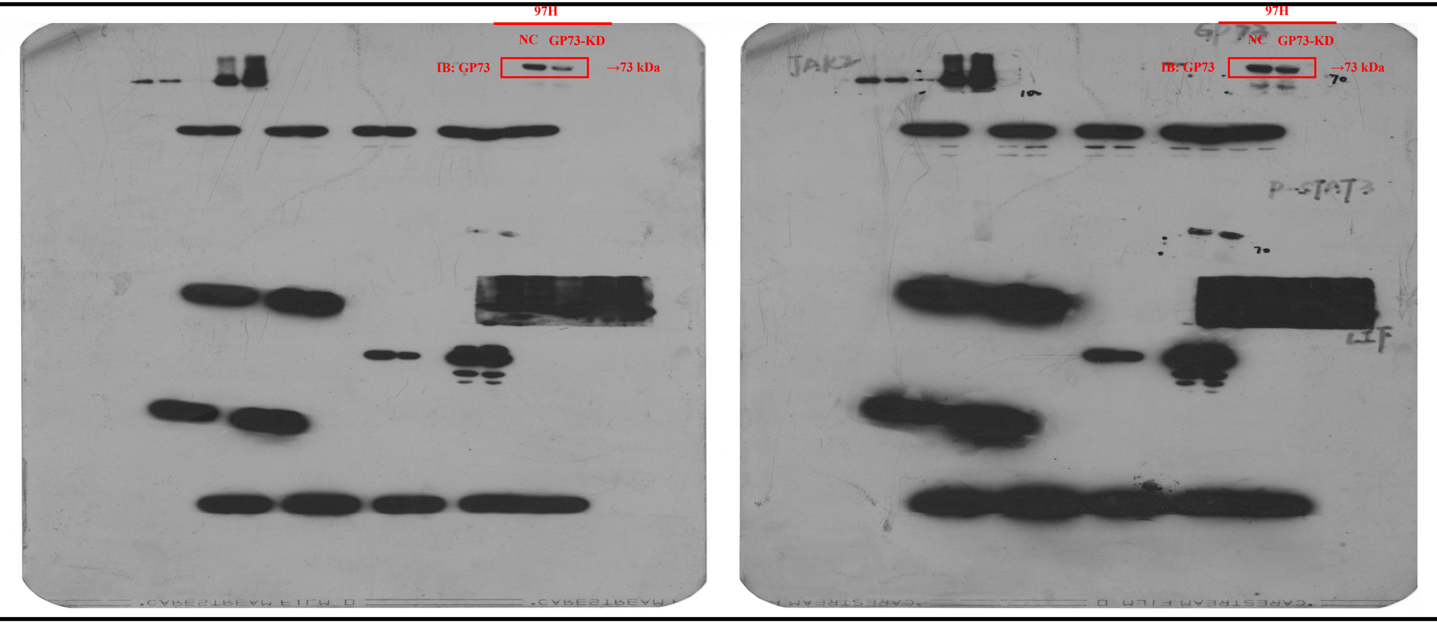

Supplement Figure 3B

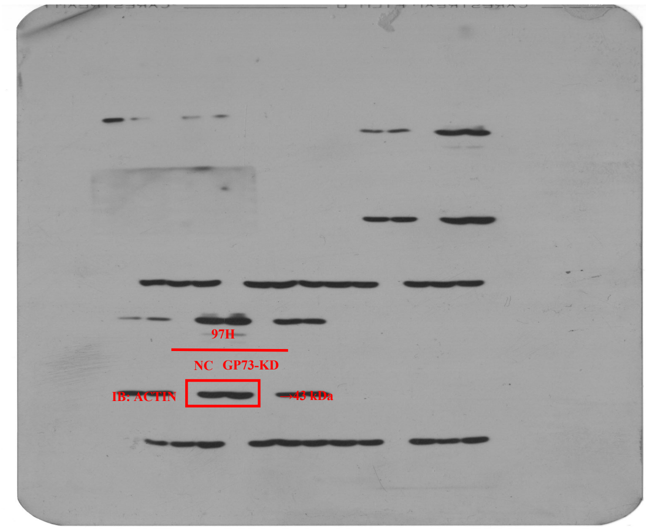

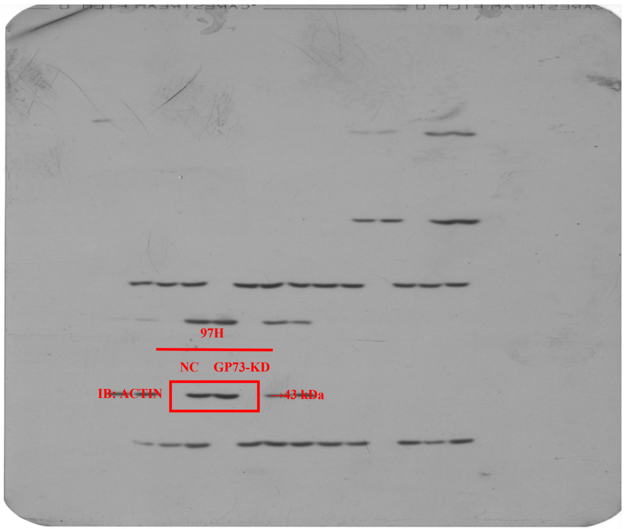

Supplement Figure 3B

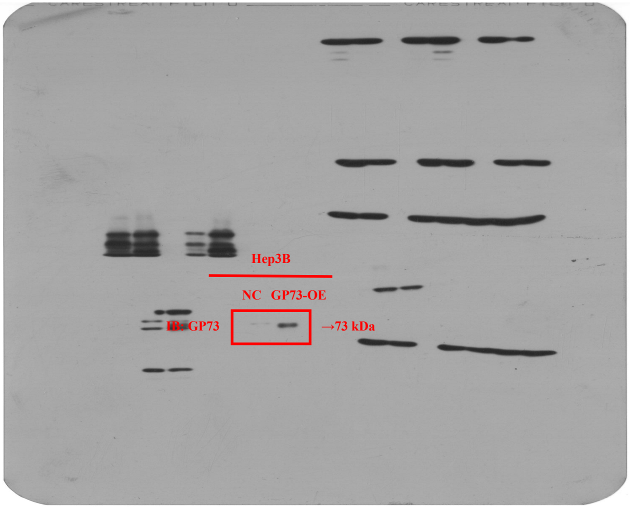

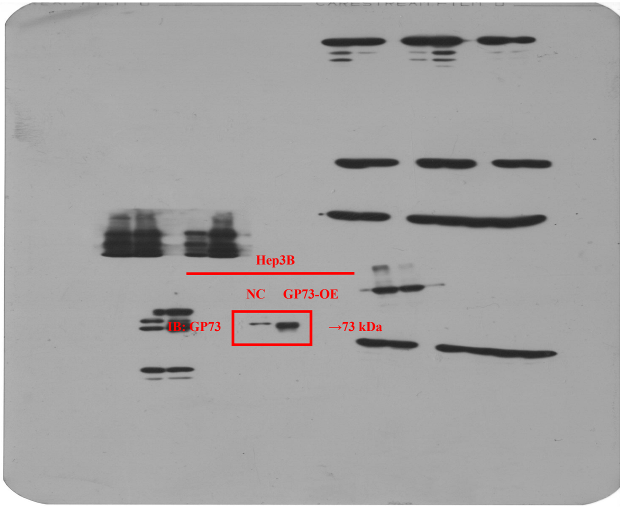

Supplement Figure 3B

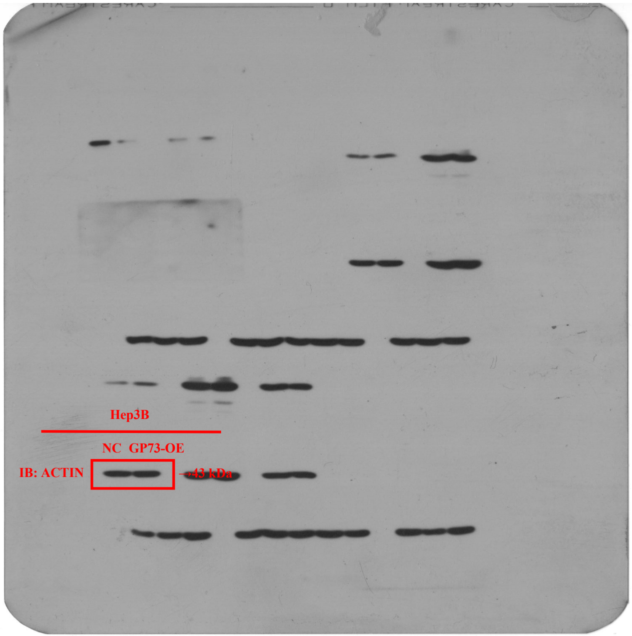

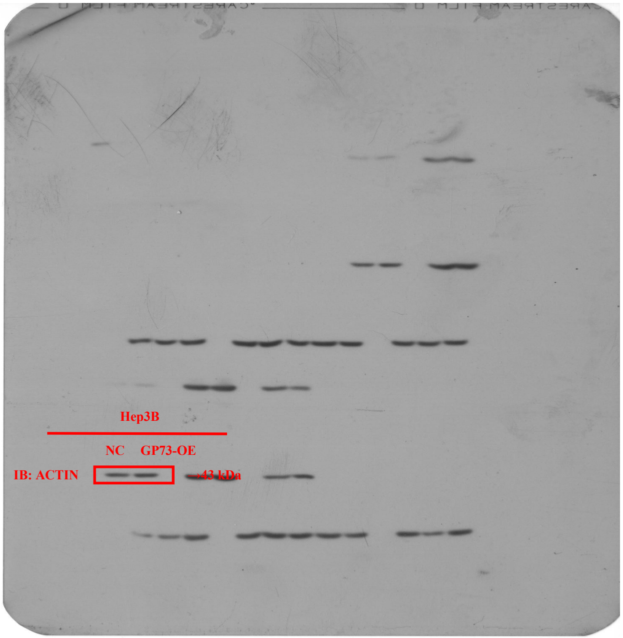

Supplement Figure 4G

Western blot detection of the mRNA and protein expression of STAT3, MCL-1 and BCL-2 in Hep3B-GP73-OE and Hep3B-GP73-OE-STAT3-KD cells.

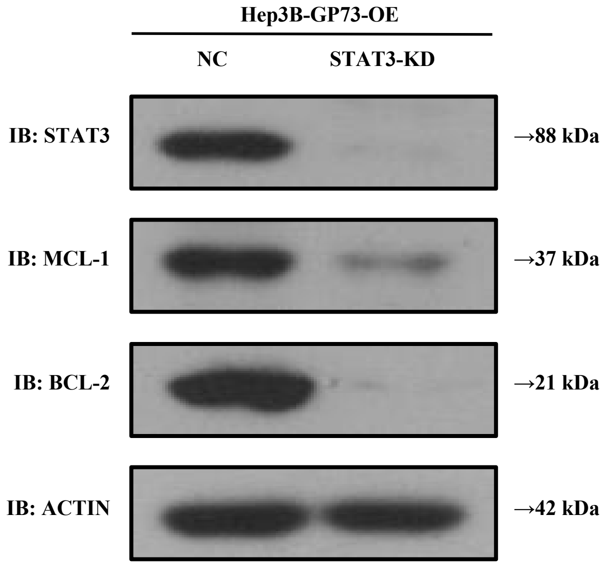

Supplement Figure 4G

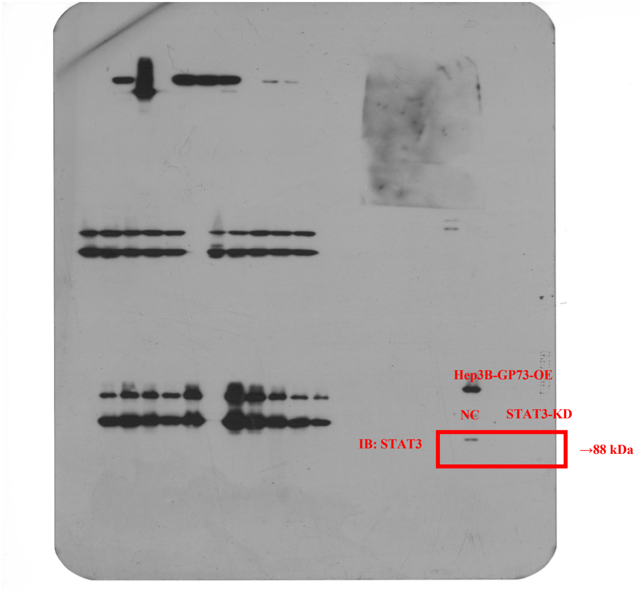

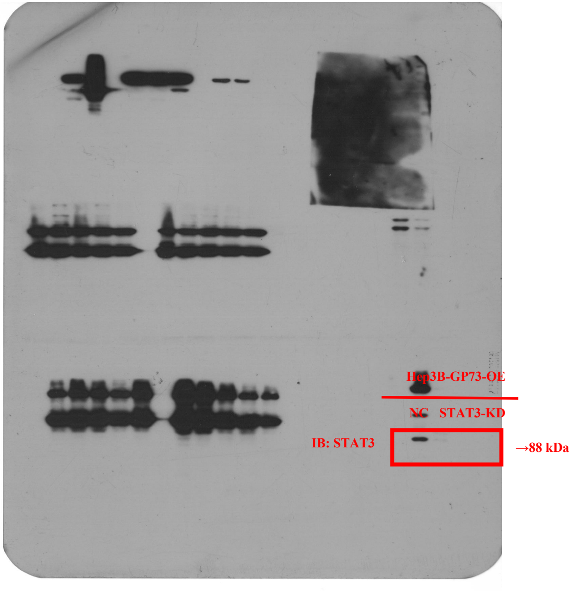

Supplement Figure 4G

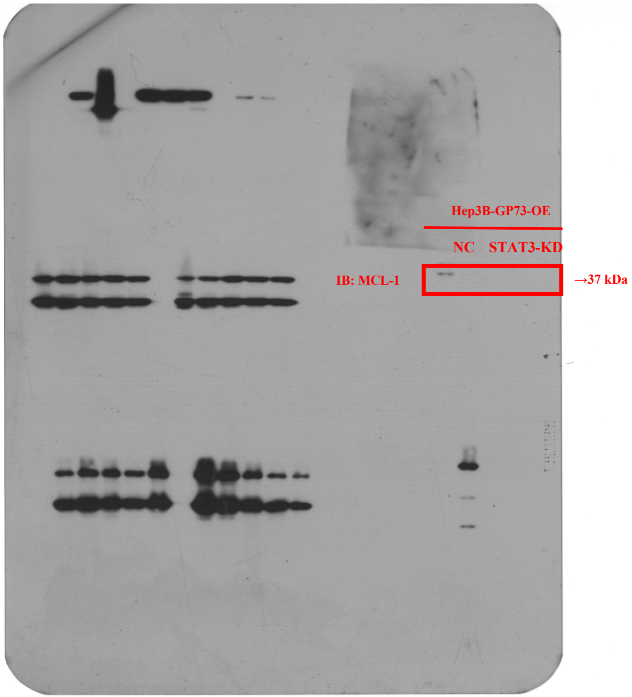

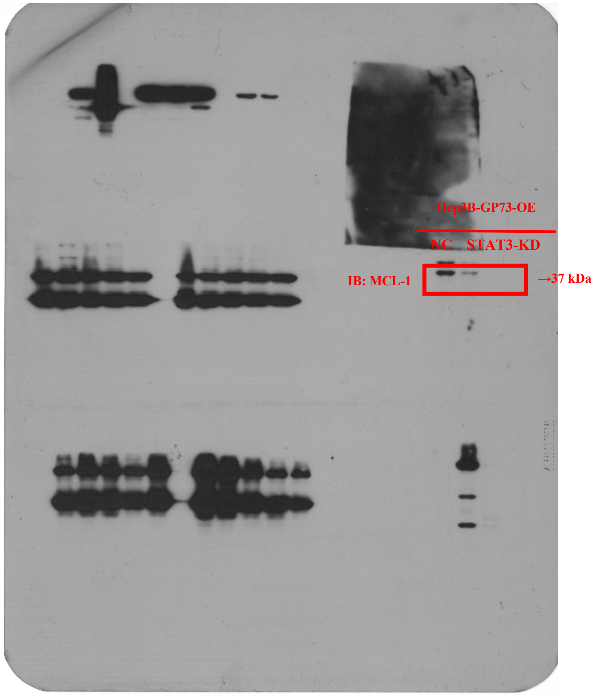

Supplement Figure 4G

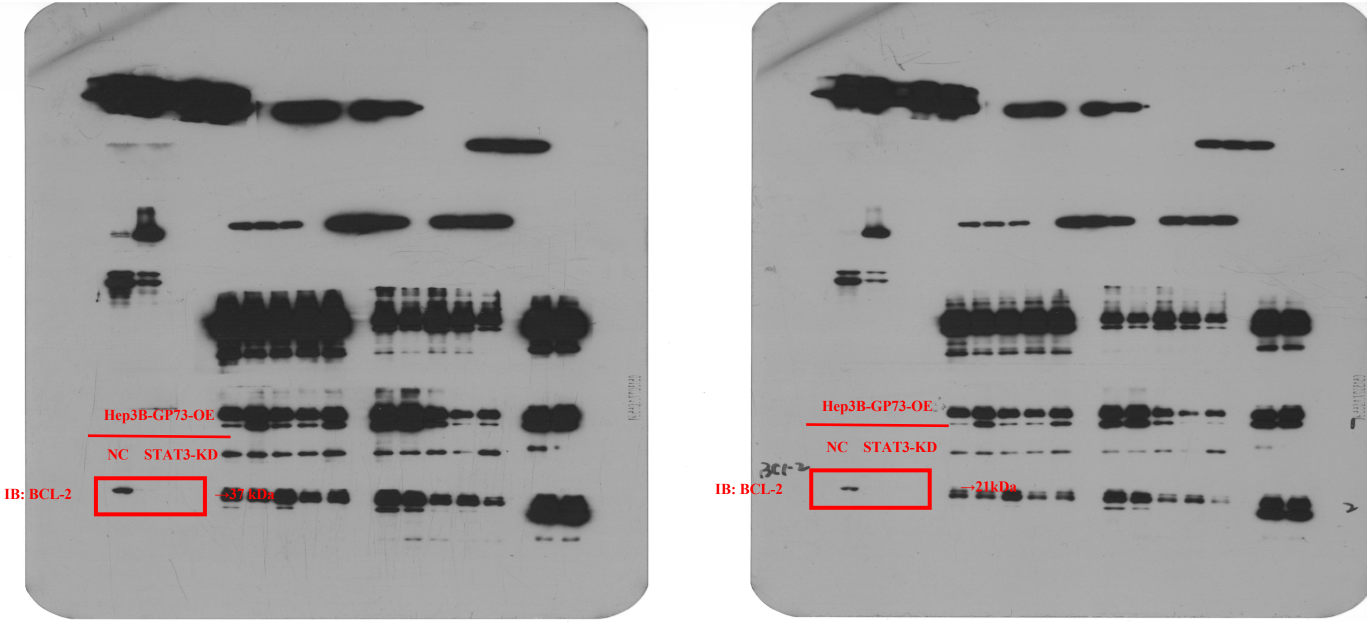

Supplement Figure 4G

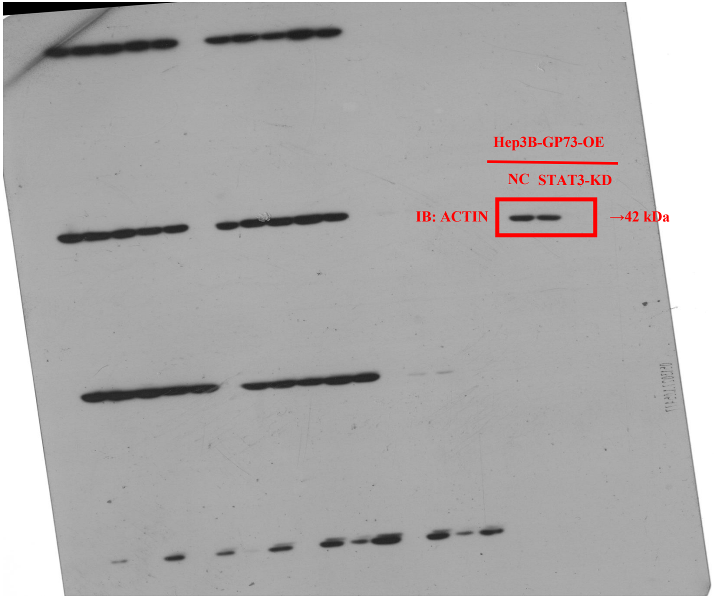

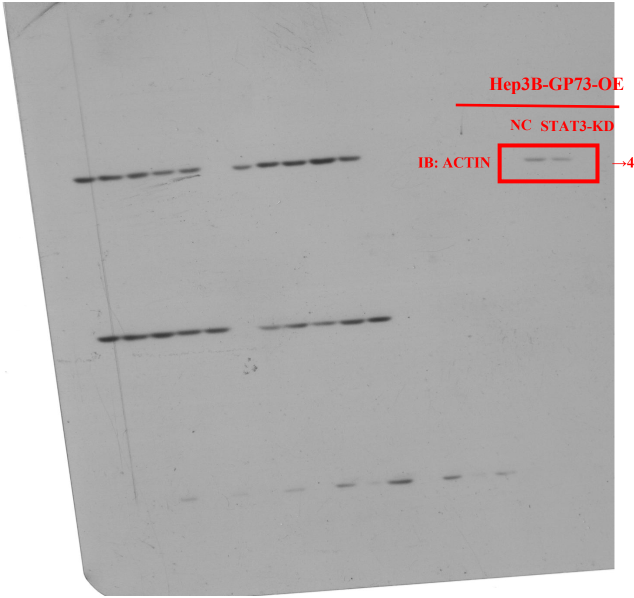

Supplement Figure 5C

Western blot detection of the mRNA and protein expressions of STAT3, MCL-1, BCL-2 in MHCC97H-GP73-KD and MHCC97H-GP73-KD-STAT3-OE cells

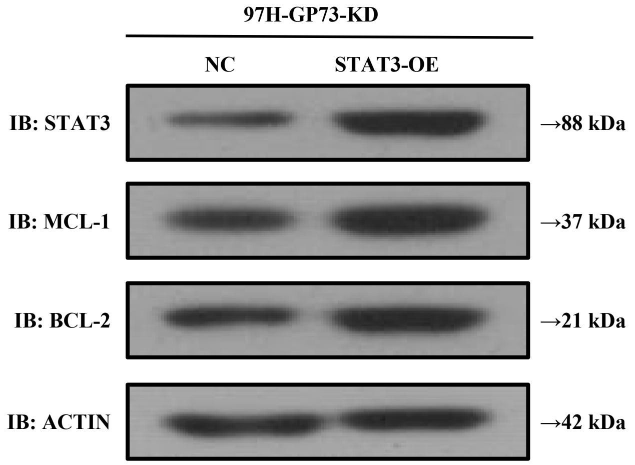

Supplement Figure 5C

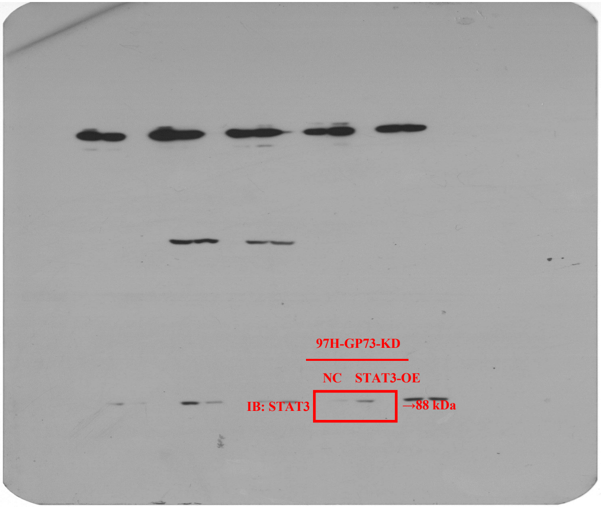

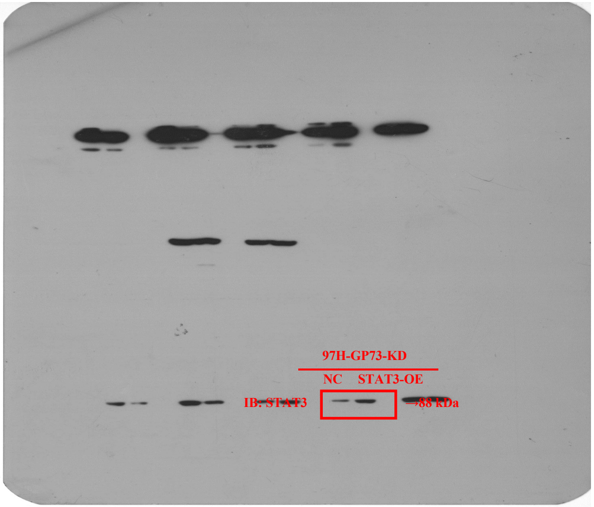

Supplement Figure 5C

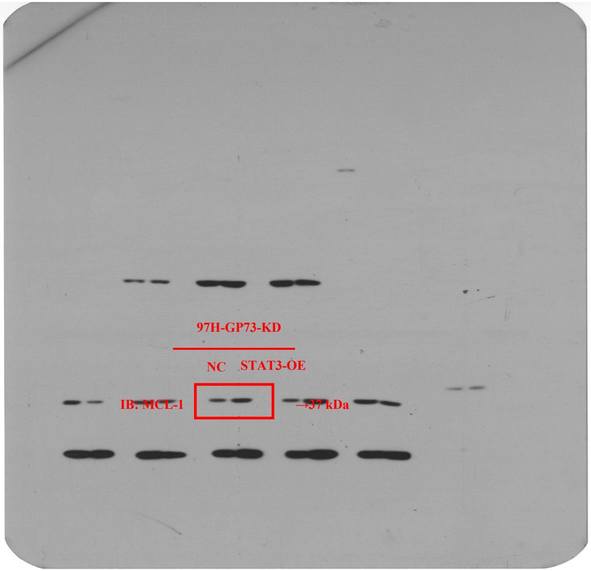

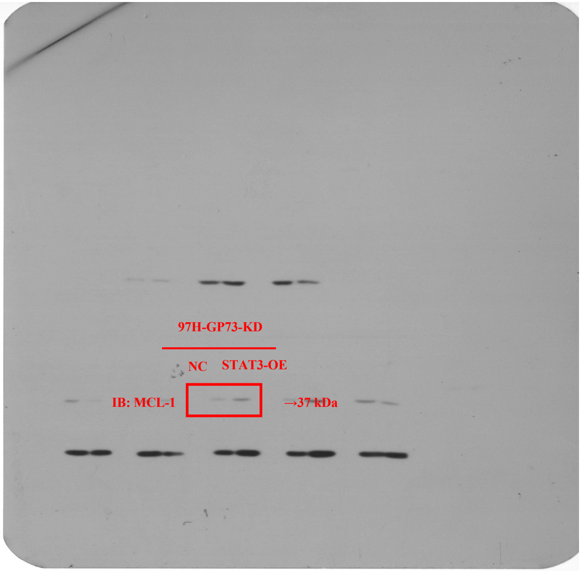

Supplement Figure 5C

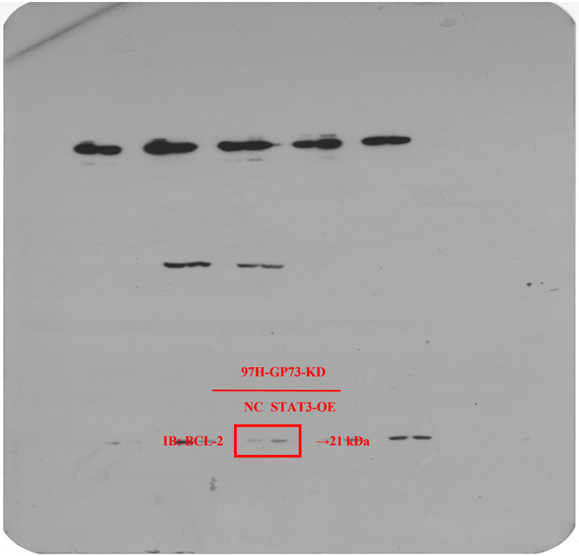

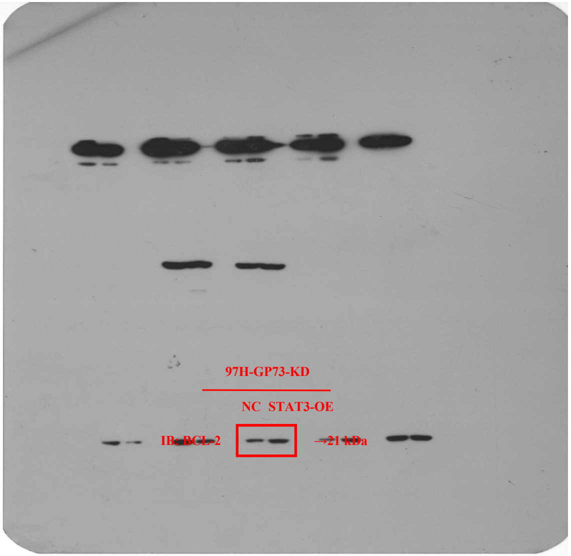

Supplement Figure 5C

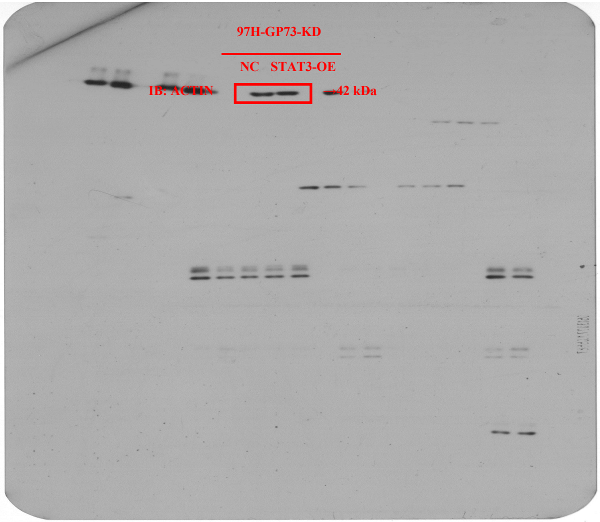

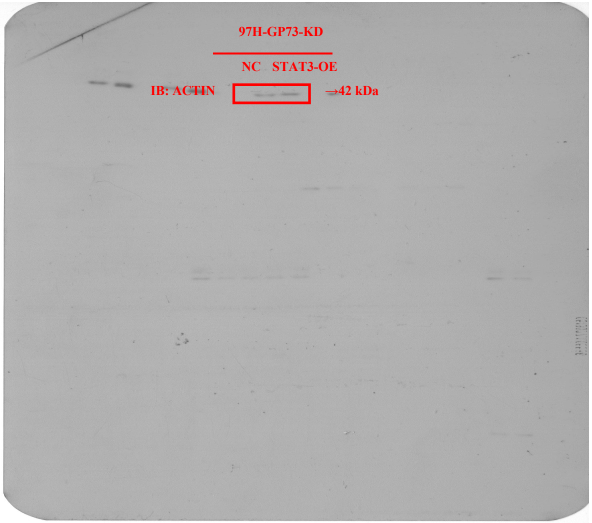

Supplement Figure 5G

Western blot detection of the expressions of STAT3, pSTAT3-Ser727, pSTAT3-Tyr705 in MHCC97H-NC, MHCC97H-GP73-KD, MHCC97H-GP73-KD-STAT3-OE, MHCC97H-GP73-KD-STAT3-Tyr705 and MHCC97H-GP73-KD-STAT3-Ser727 cells.

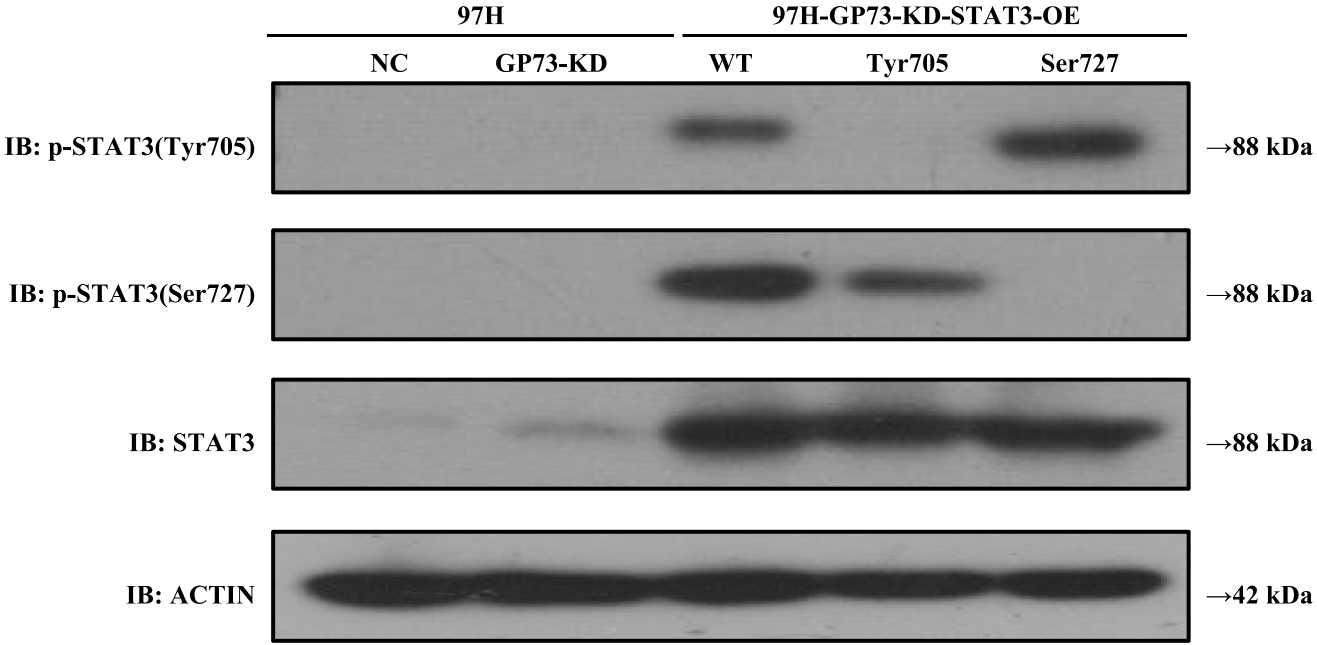

Supplement Figure 5G

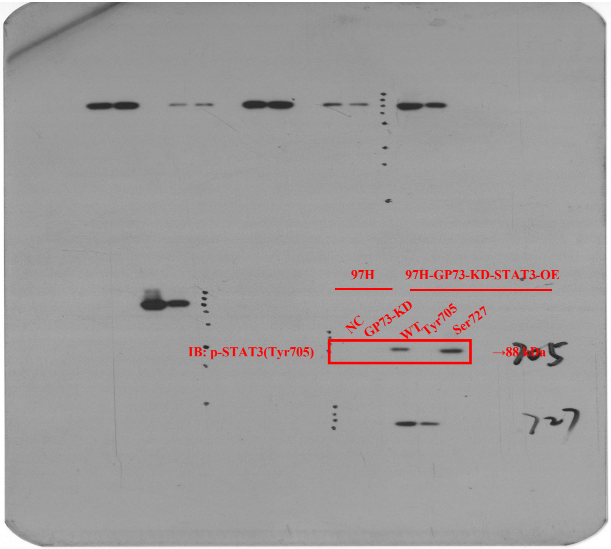

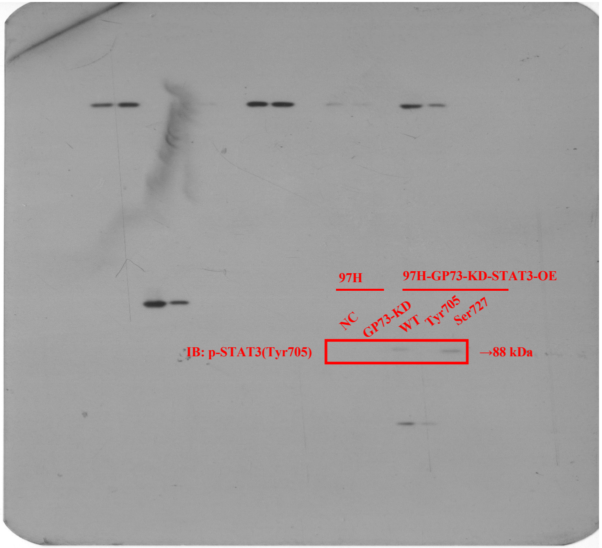

Supplement Figure 5G

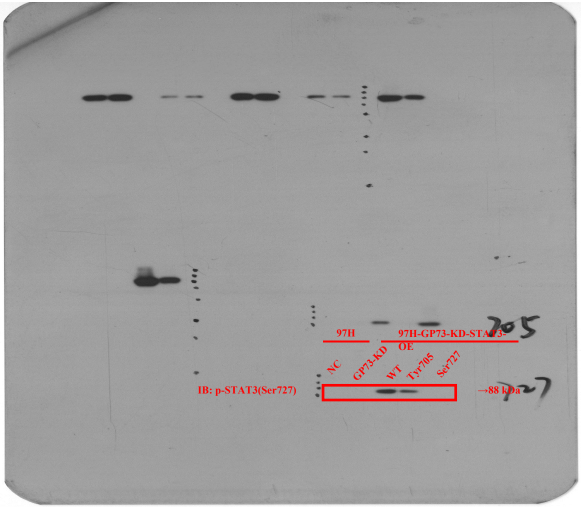

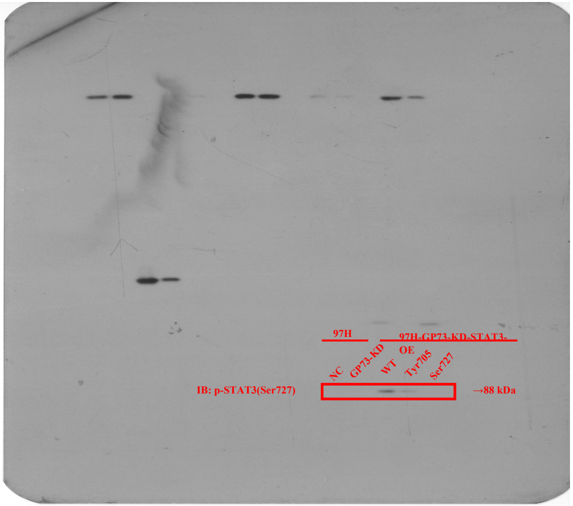

Supplement Figure 5G

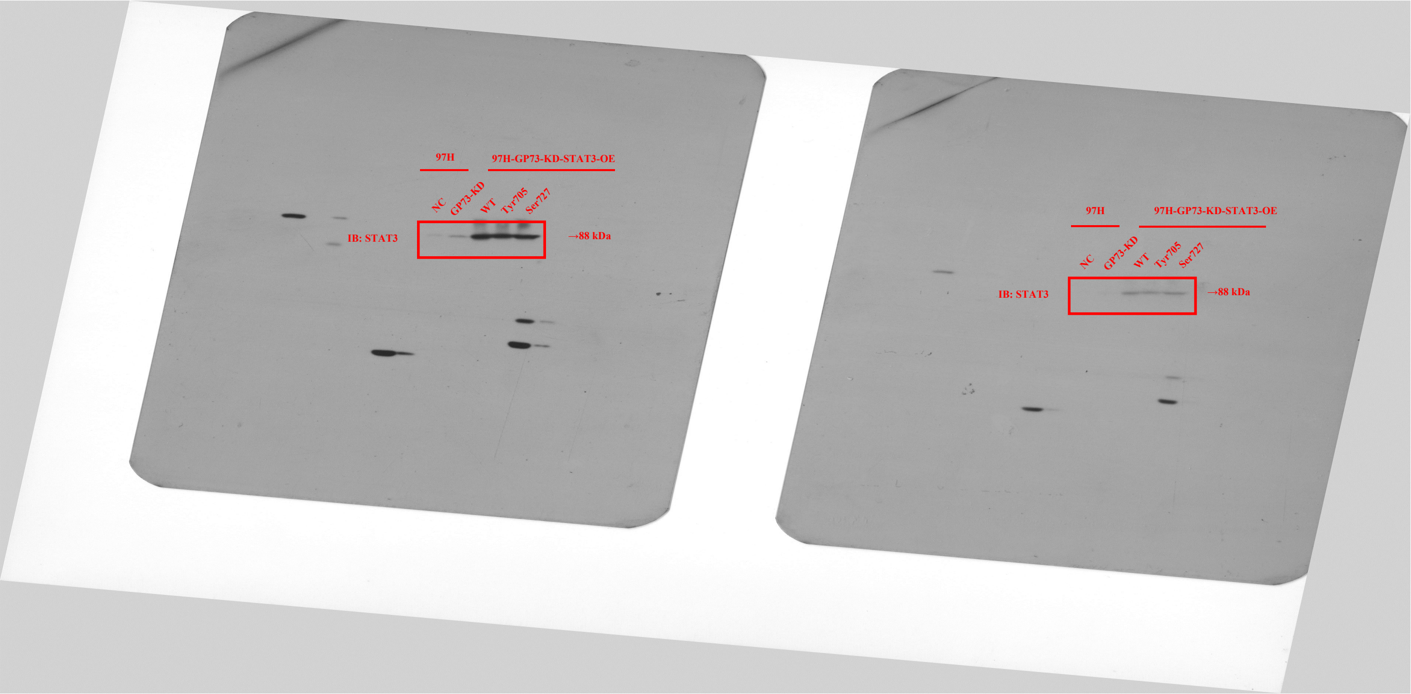

Supplement Figure 5G

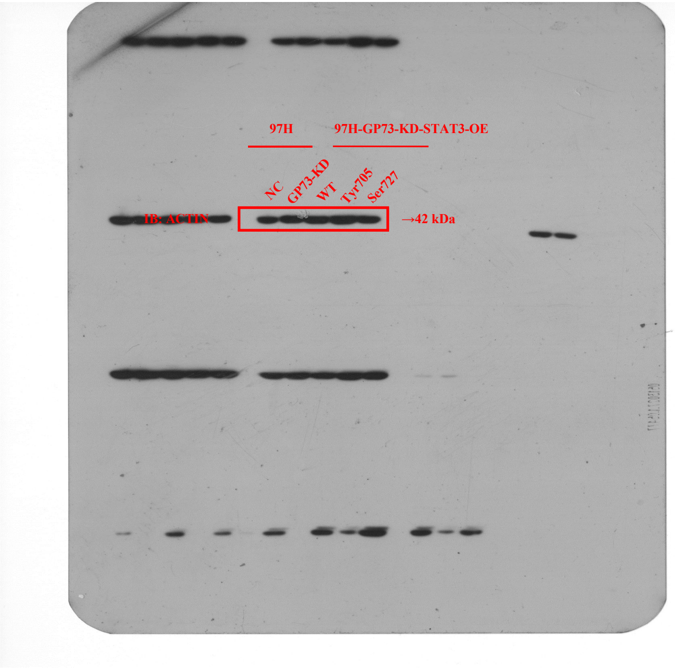

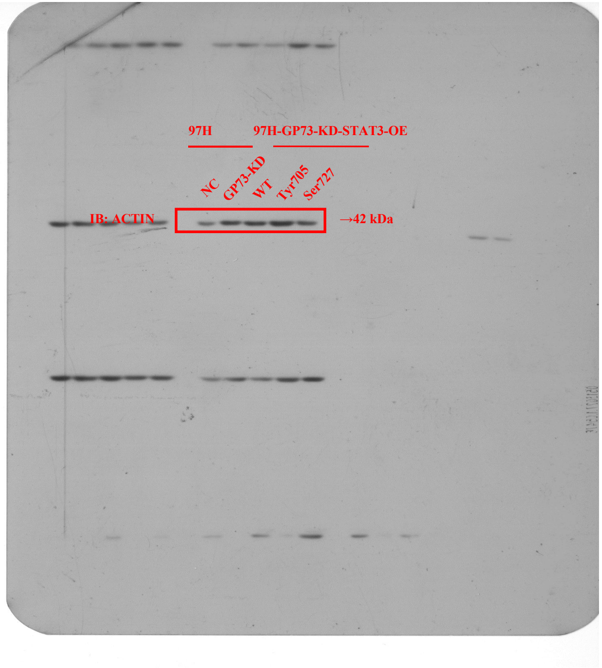

Supplement Figure 6F

Western blot detection of LDHA and LDHB expressions in MHCC97H-NC, MHCC97H-LDHA-KD, MHCC97H-LDHB-KD and MHCC97H-LDHA-KD-LDHB-KD cells.

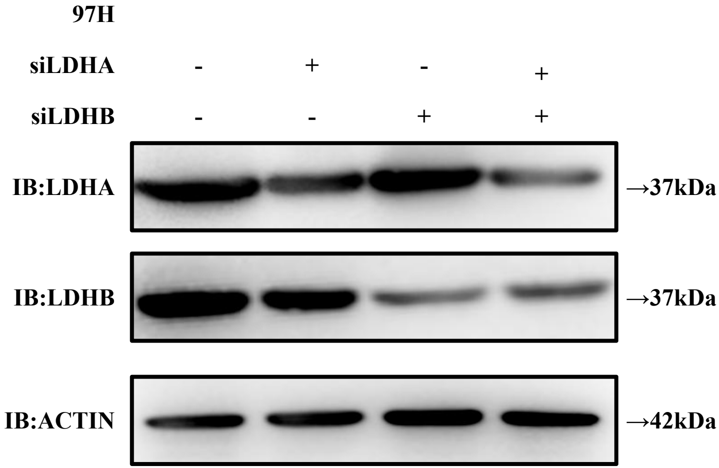

Supplement Figure 6F

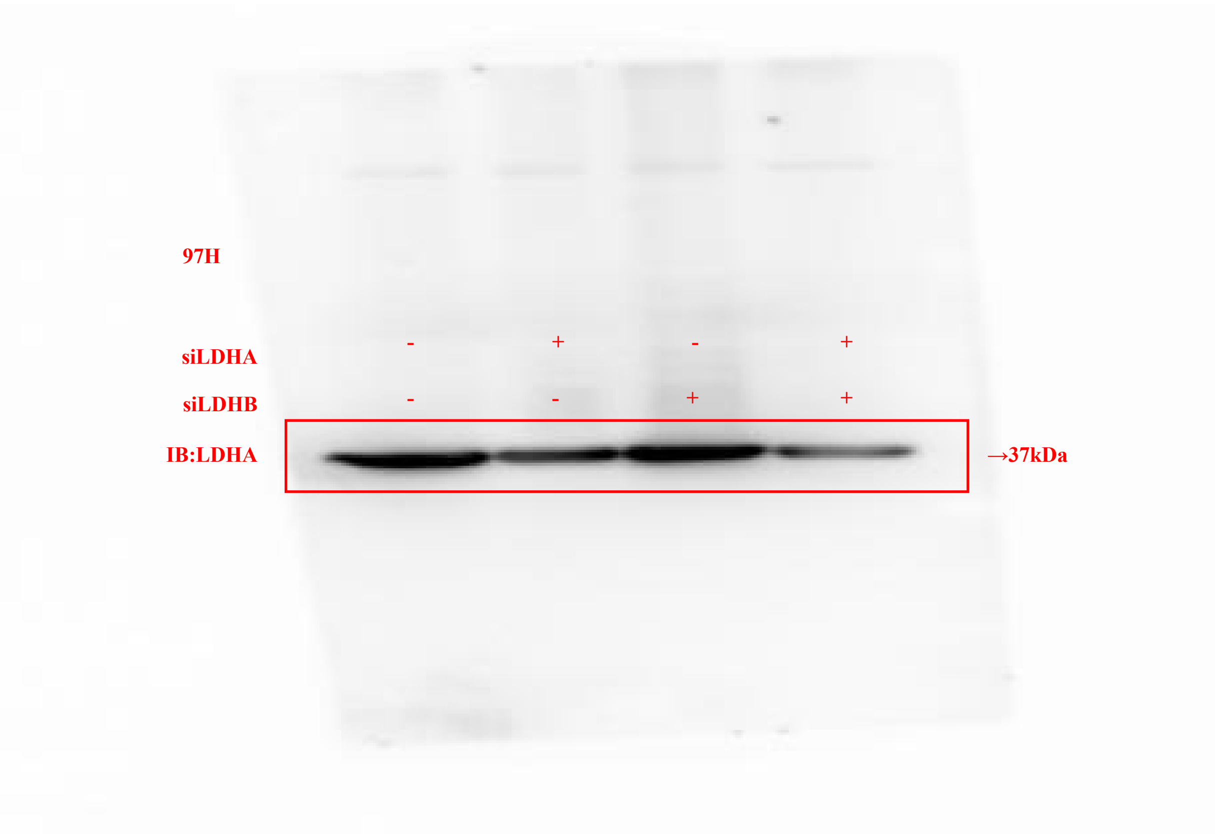

Supplement Figure 6F

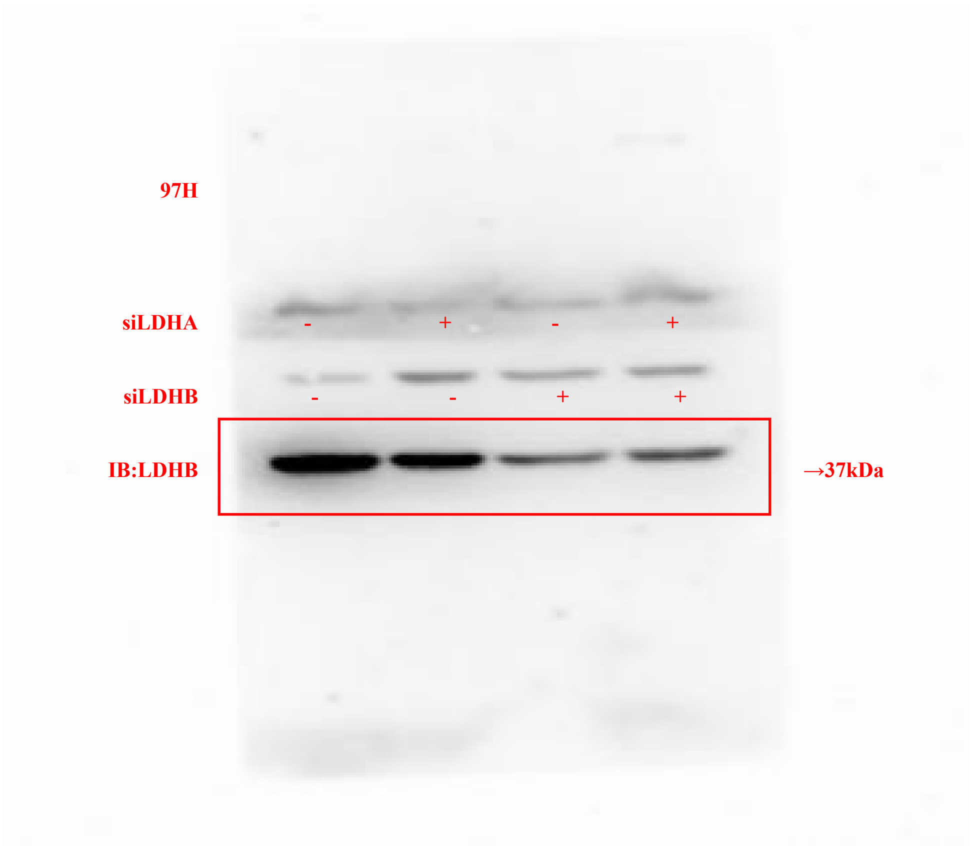

Supplement Figure 6F

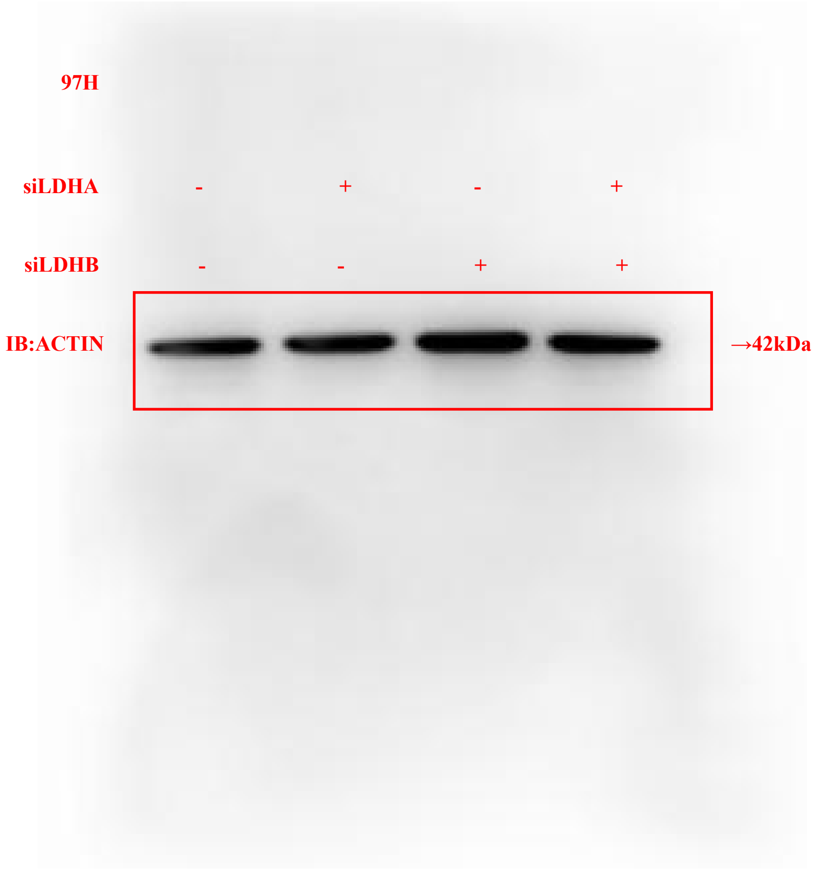

Supplement Figure 6I

Western blot detection of P300 expression in MHCC97H-NC and MHCC97H-P300-KD cells.

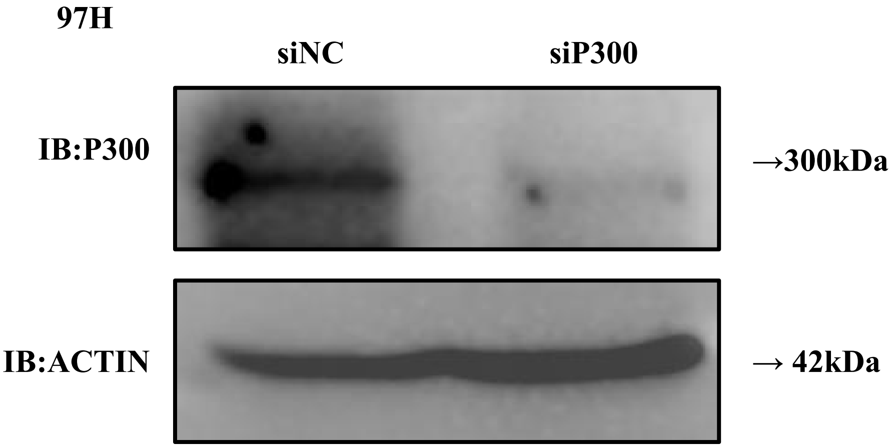

Supplement Figure 6I

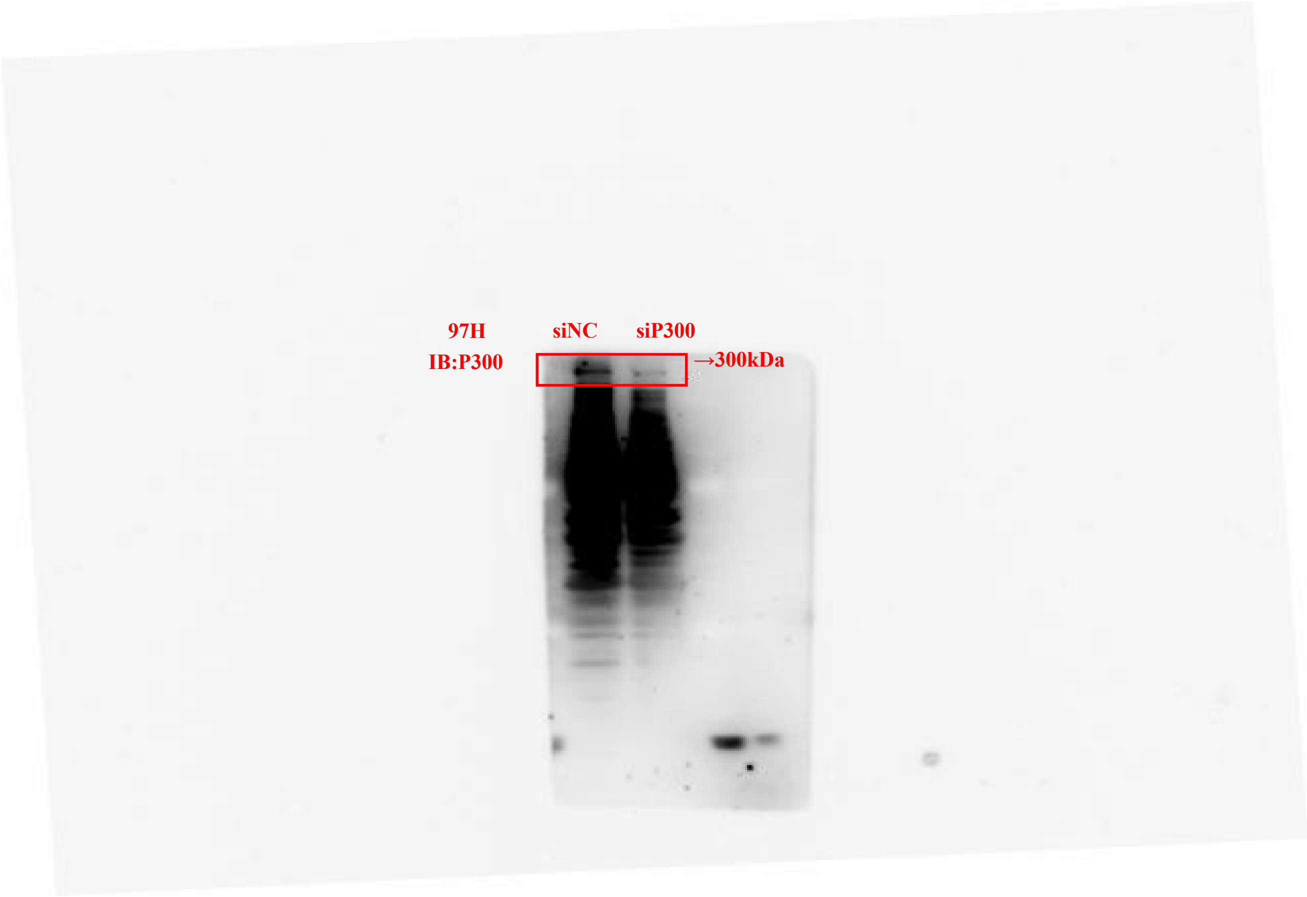

Supplement Figure 6I

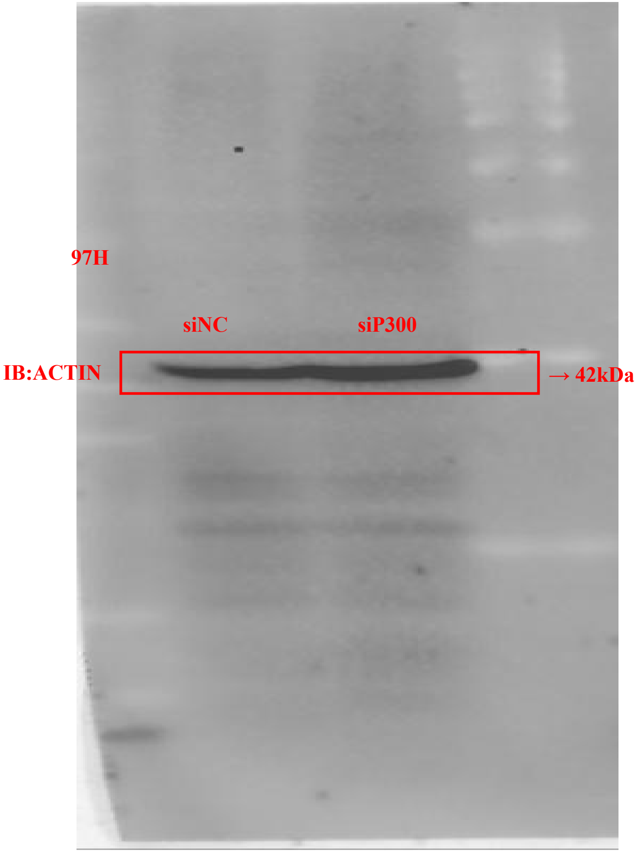

Supplement Figure 7F

Western blot detection of the expressions of GP73 and c-Myc in MHCC97H-NC, MHCC97H-GP73-KD, MHCC97H-c-Myc-OE and MHCC97H-c-Myc-OE-GP73-KD cells.

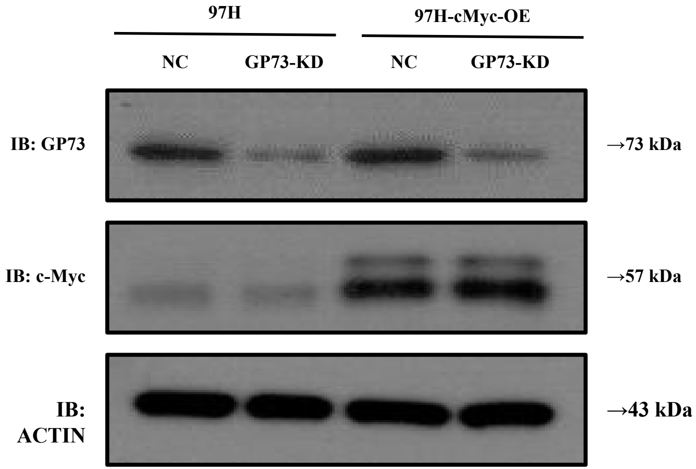

Supplement Figure 7F

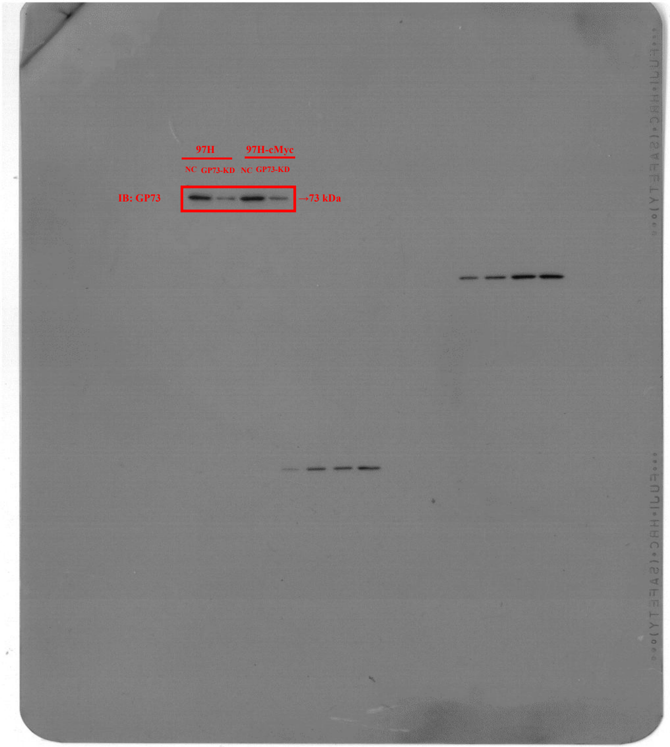

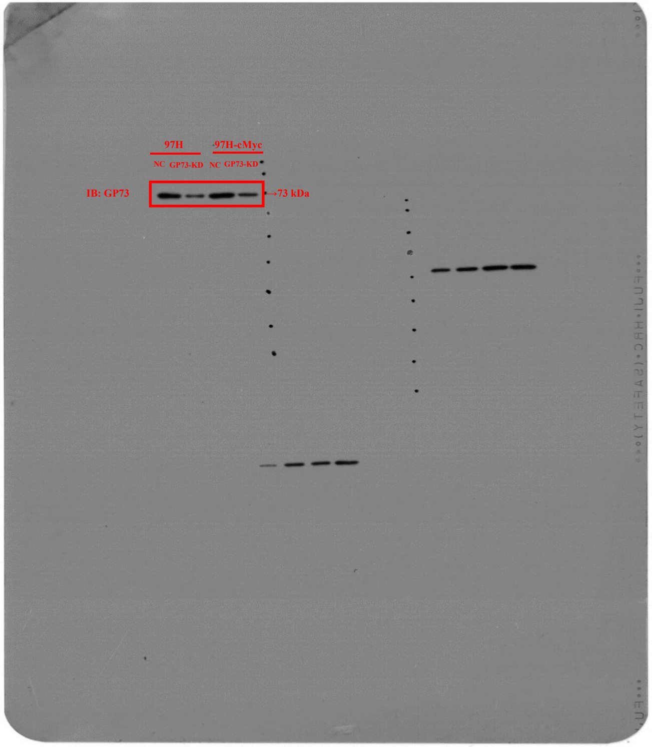

Supplement Figure 7F

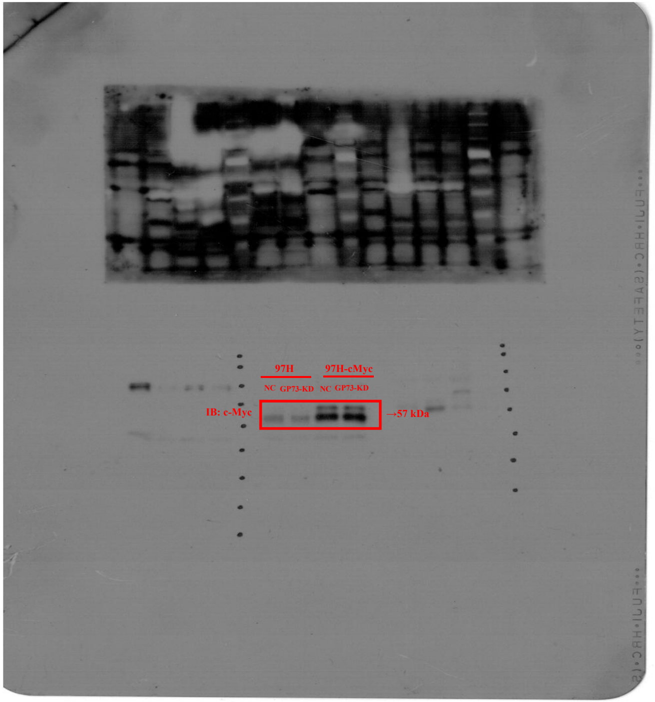

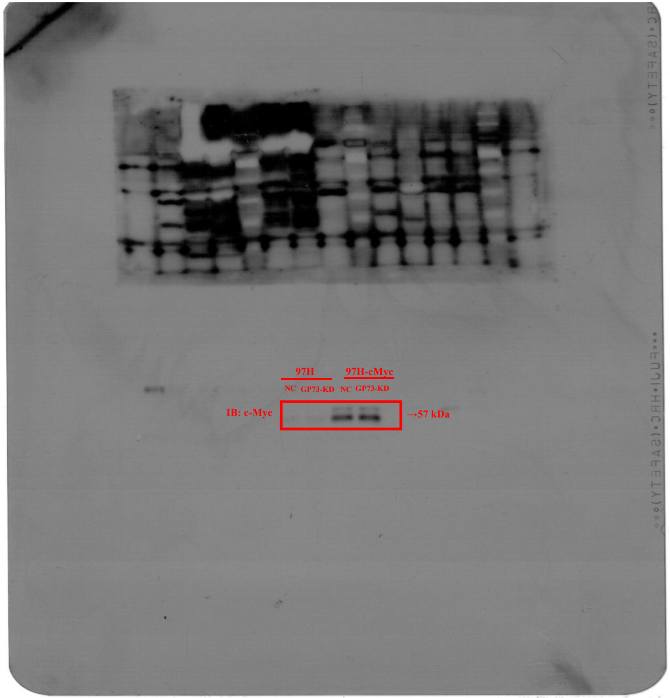

Supplement Figure 7F

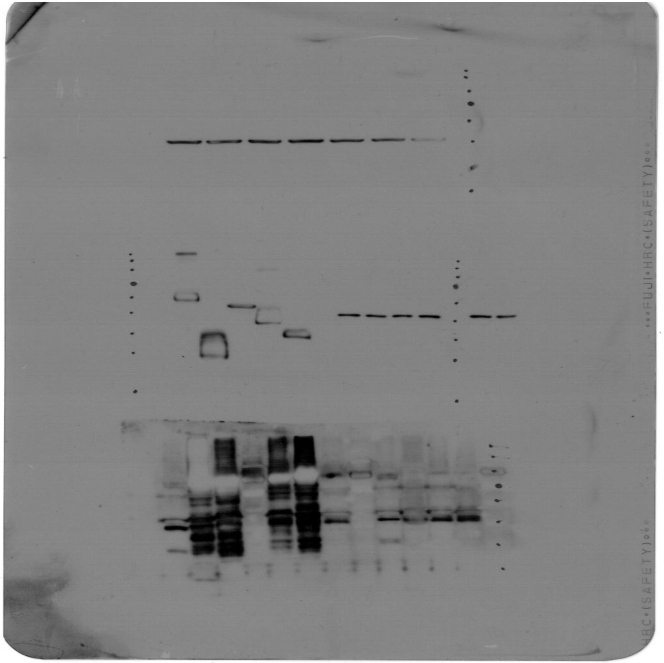

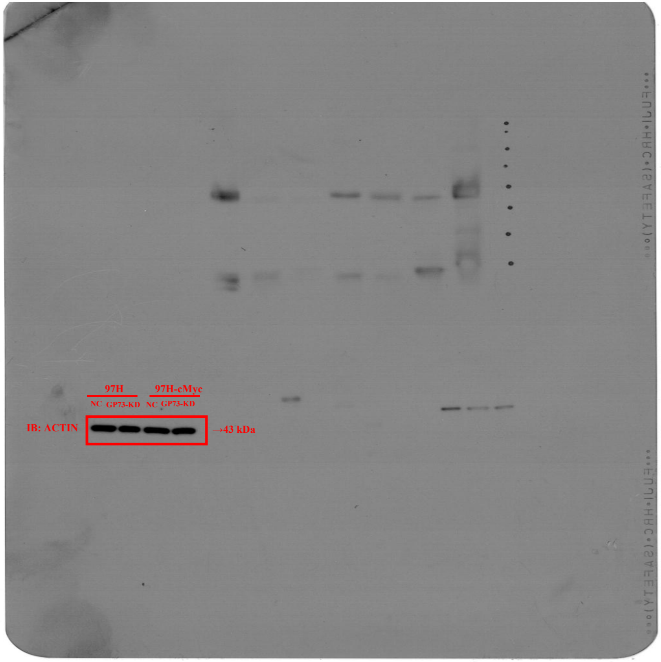

Supplement: Supplementary 1 — Supplementary Methods Supplementary Results Figs. S1 to S7 Tables S1 to S9 Files S1 to S4 [file research.0387.f1.zip › Supplemental file 3.pdf]
