# Supplementary material for: Integrating Single-Cell and Spatial Transcriptomics to Uncover and Elucidate GP73-Mediated Pro-Angiogenic Regulatory Networks in Hepatocellular Carcinoma
Source: Research (Wash D C). 2024 Jun 27;7:0387. doi: 10.34133/research.0387 (PMC11208919; doi:10.34133/research.0387)
Supplement: Supplementary 1 — Supplementary Methods Supplementary Results Figs. S1 to S7 Tables S1 to S9 Files S1 to S4 [file research.0387.f1.zip › Supplemental file 4.pdf]

Figure 3F

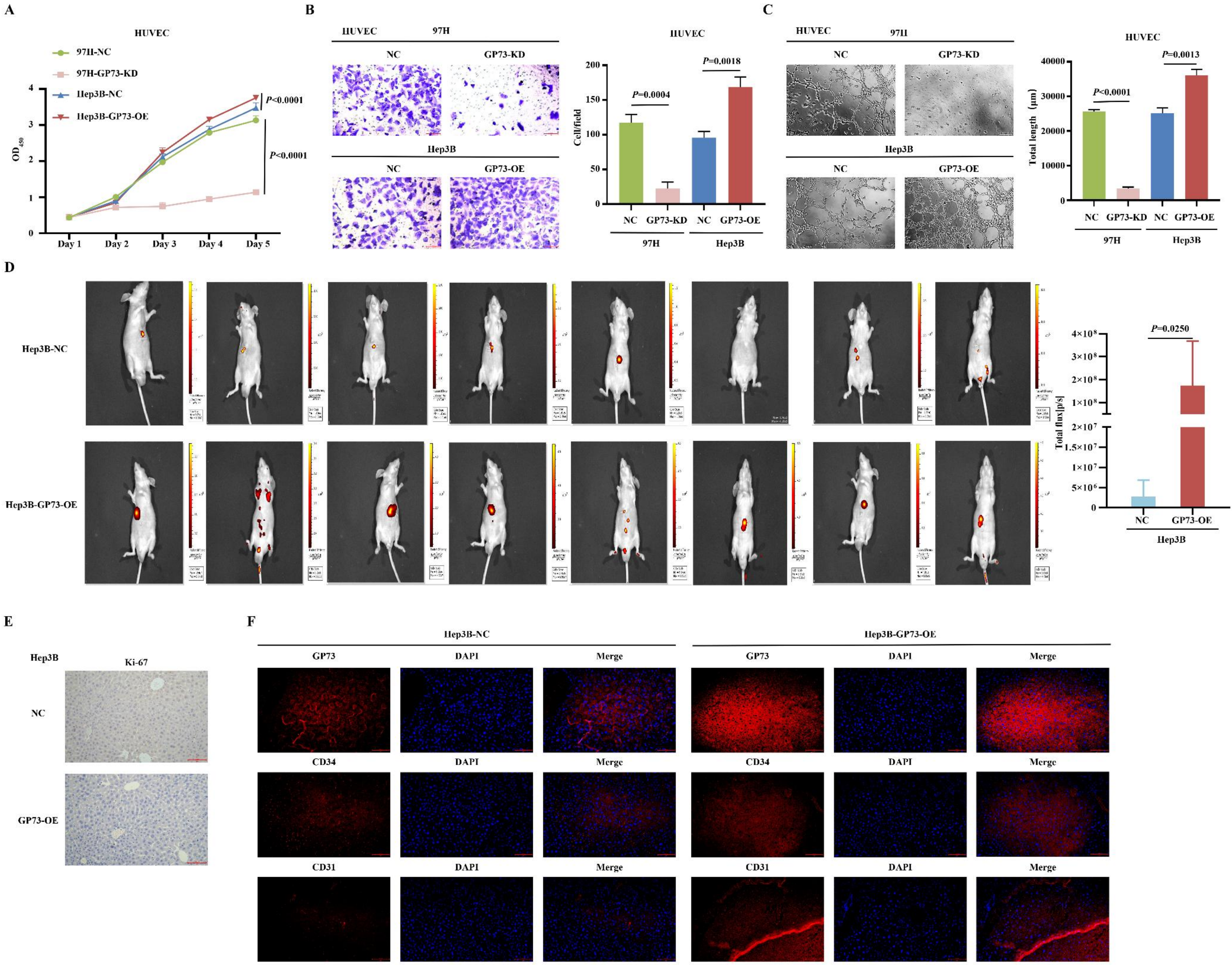
A
HUVEC
97H-NC
97H-GP73-KD
Hep3B-NC
Hep3B-GP73-OE
P<0.0001
P<0.0001
OD450
Day 1
Day 2
Day 3
Day 4
Day 5
B
HUVEC 97H
NC
GP73-KD
Hep3B
NC
GP73-OE
HUVEC
P=0.0004
P=0.0018
Cell/field
NC GP73-KD NC GP73-OE
97H Hep3B
C
HUVEC 97H
NC
GP73-KD
Hep3B
NC
GP73-OE
HUVEC
P<0.0001
P=0.0013
Total length (μm)
NC GP73-KD NC GP73-OE
97H Hep3B
D
Hep3B-NC
Hep3B-GP73-OE
P=0.0250
Total flux[p/s]
NC
GP73-OE
Hep3B
E
Hep3B
Ki-67
NC
GP73-OE
F
Hep3B-NC
Hep3B-GP73-OE
GP73
DAPI
Merge
CD34
CD31

Immunofluorescence staining showing increased expressions of GP73 in resected tumors originated from the Hep3B-GP73-OE cells compared to the control, original magnification, ×20.

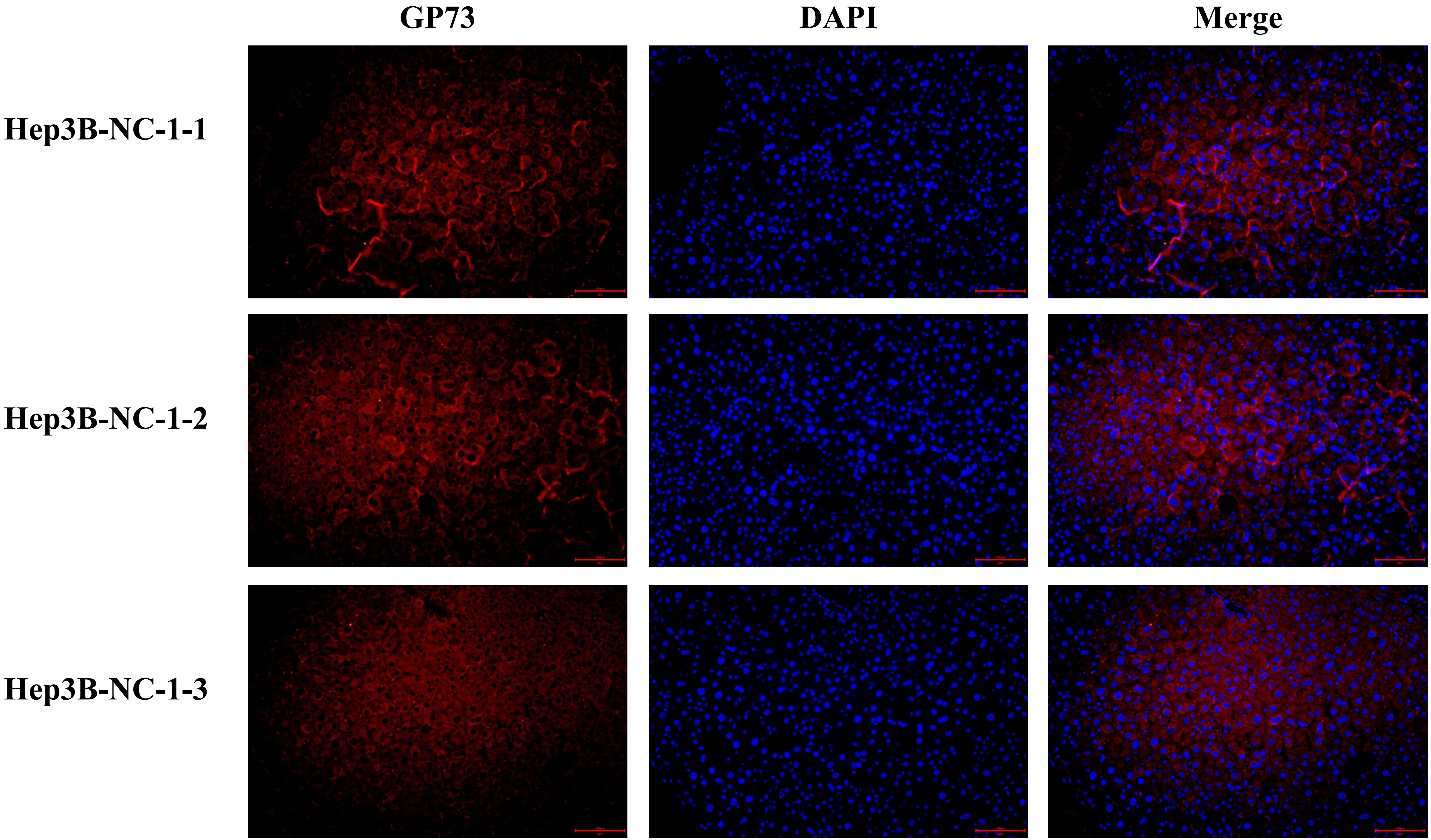
GP73
DAPI
Merge
Hep3B-NC-1-1
Hep3B-NC-1-2
Hep3B-NC-1-3

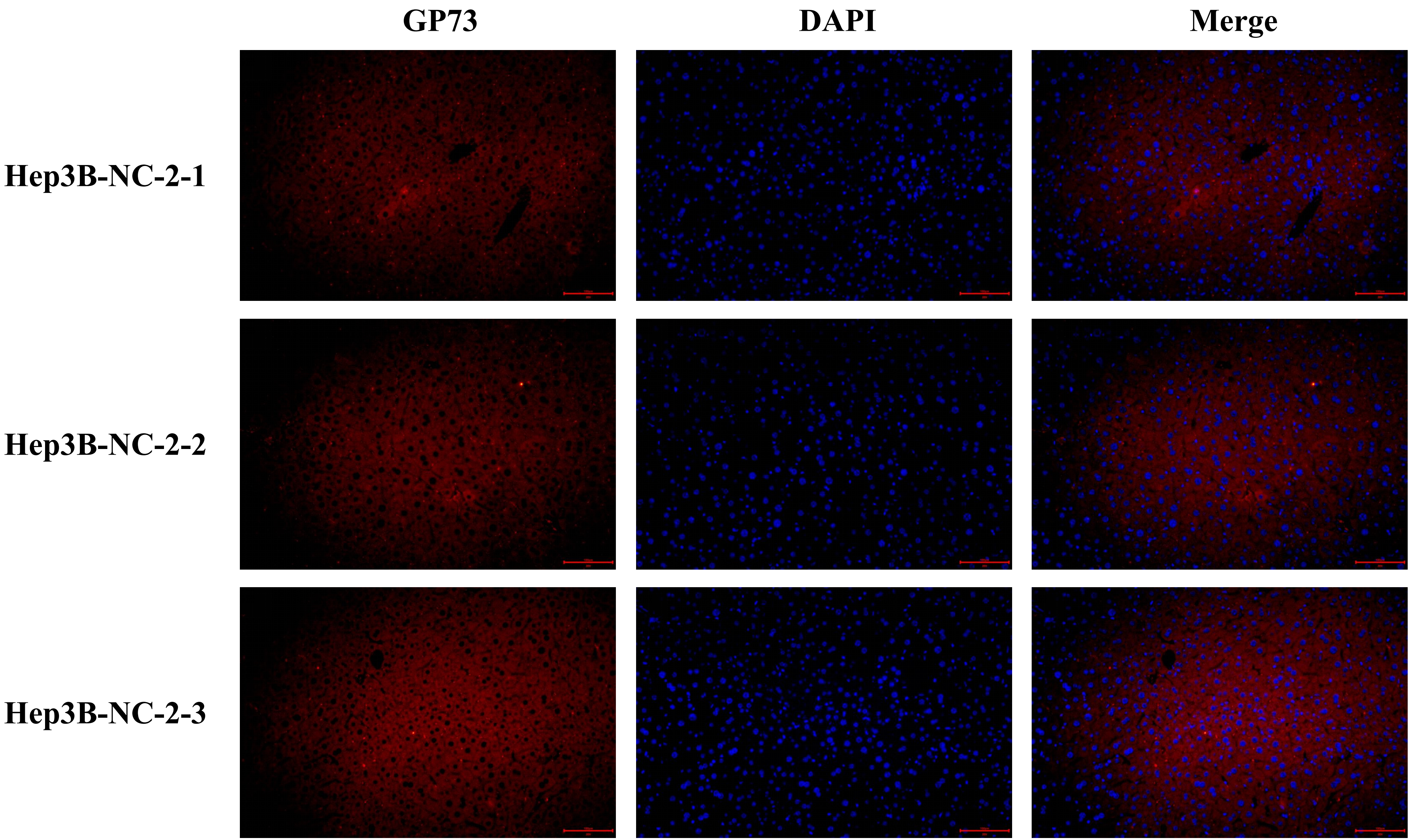
GP73
DAPI
Merge
Hep3B-NC-2-1
Hep3B-NC-2-2
Hep3B-NC-2-3

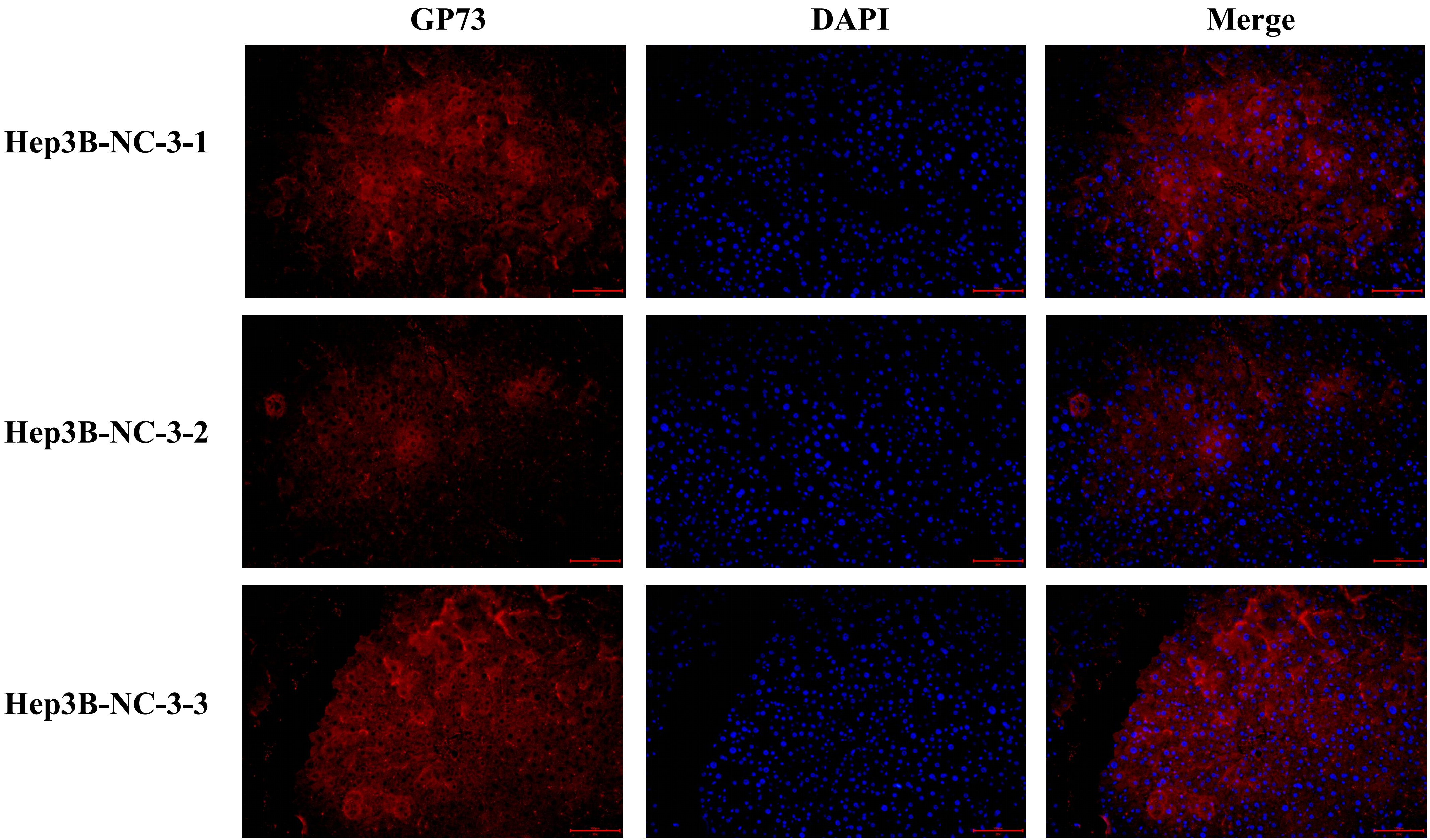
GP73
DAPI
Merge
Hep3B-NC-3-1
Hep3B-NC-3-2
Hep3B-NC-3-3

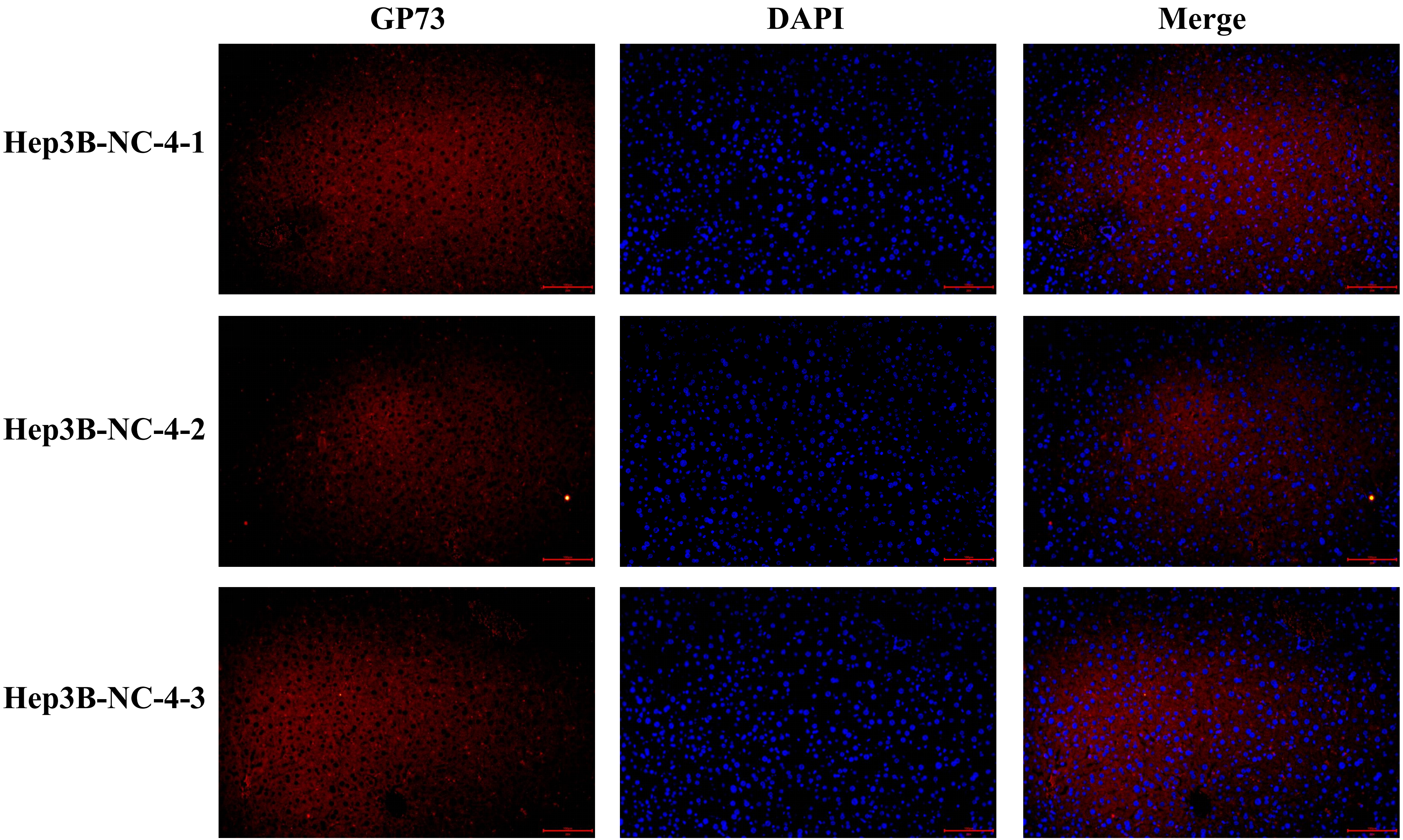
GP73
DAPI
Merge
Hep3B-NC-4-1
Hep3B-NC-4-2
Hep3B-NC-4-3

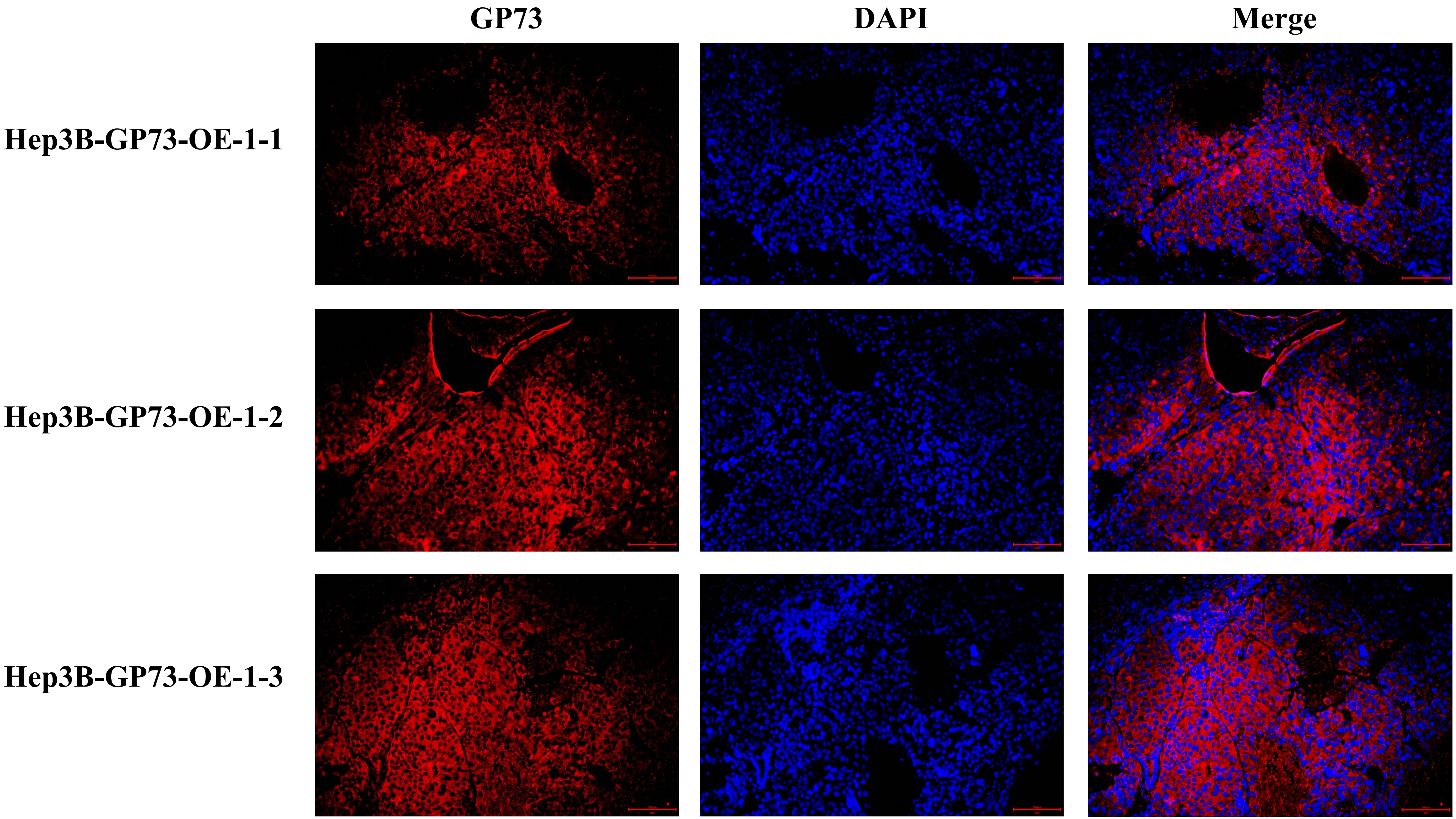
GP73
DAPI
Merge
Hep3B-GP73-OE-1-1
Hep3B-GP73-OE-1-2
Hep3B-GP73-OE-1-3

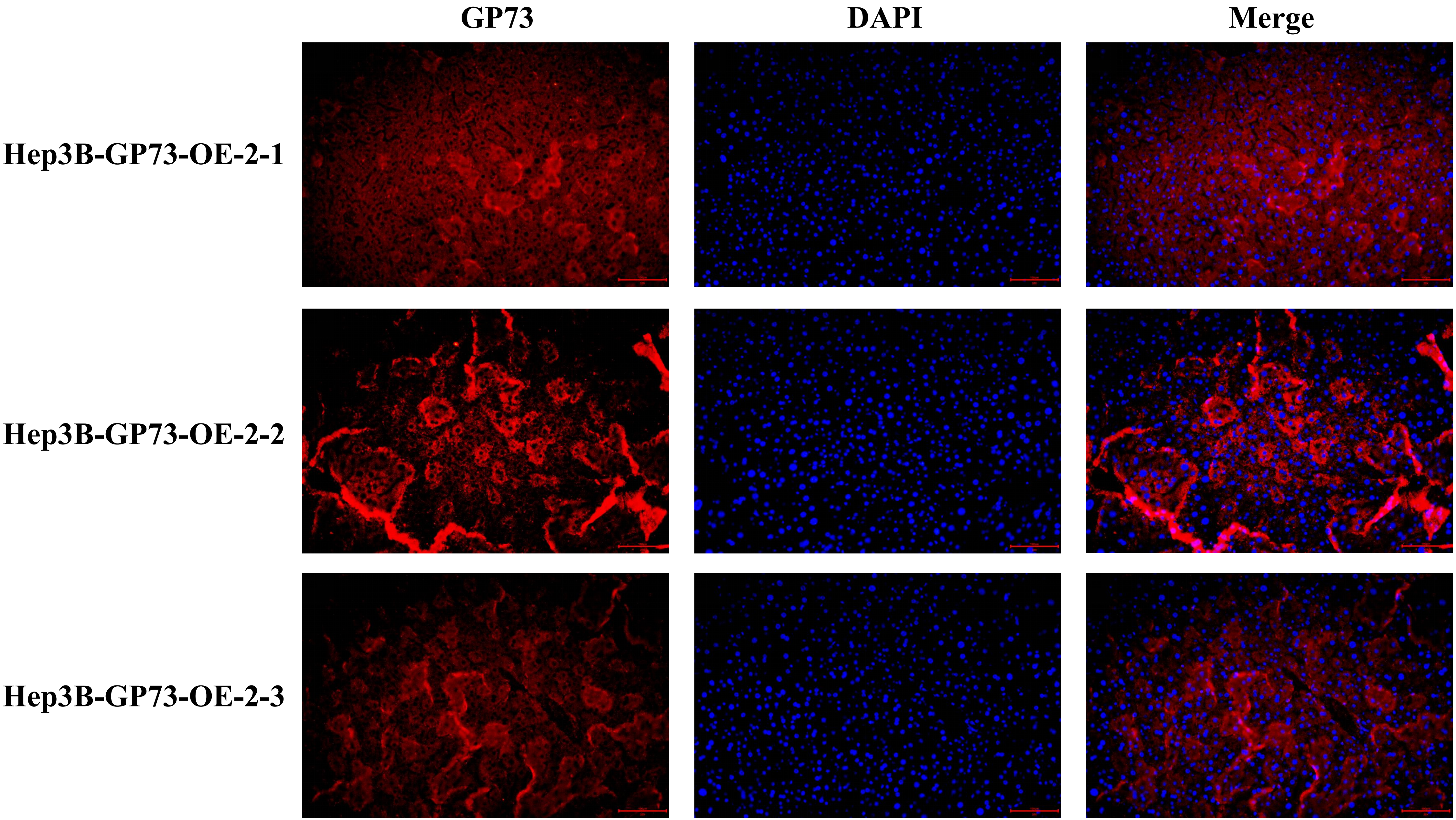
GP73
DAPI
Merge
Hep3B-GP73-OE-2-1
Hep3B-GP73-OE-2-2
Hep3B-GP73-OE-2-3

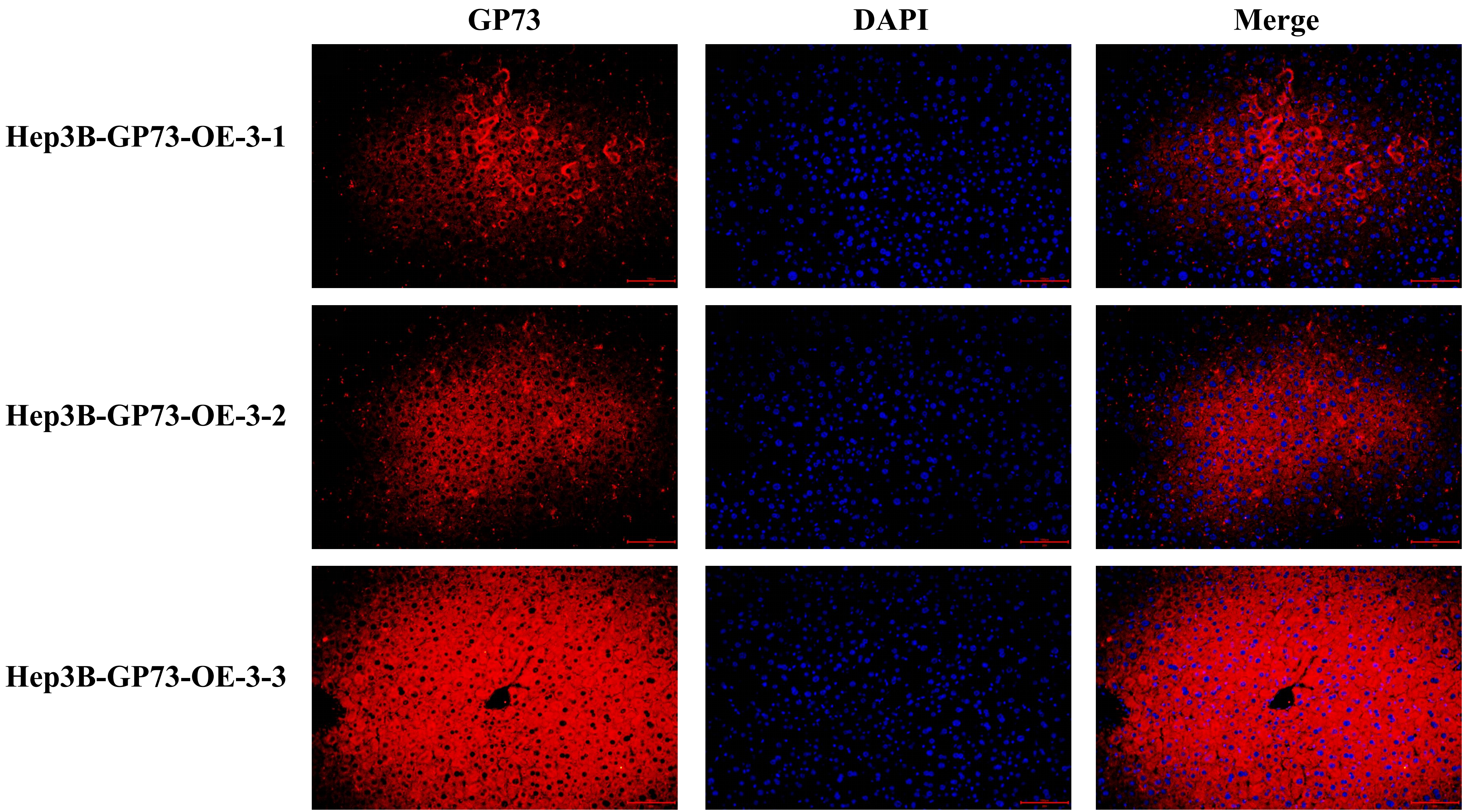
GP73
DAPI
Merge
Hep3B-GP73-OE-3-1
Hep3B-GP73-OE-3-2
Hep3B-GP73-OE-3-3

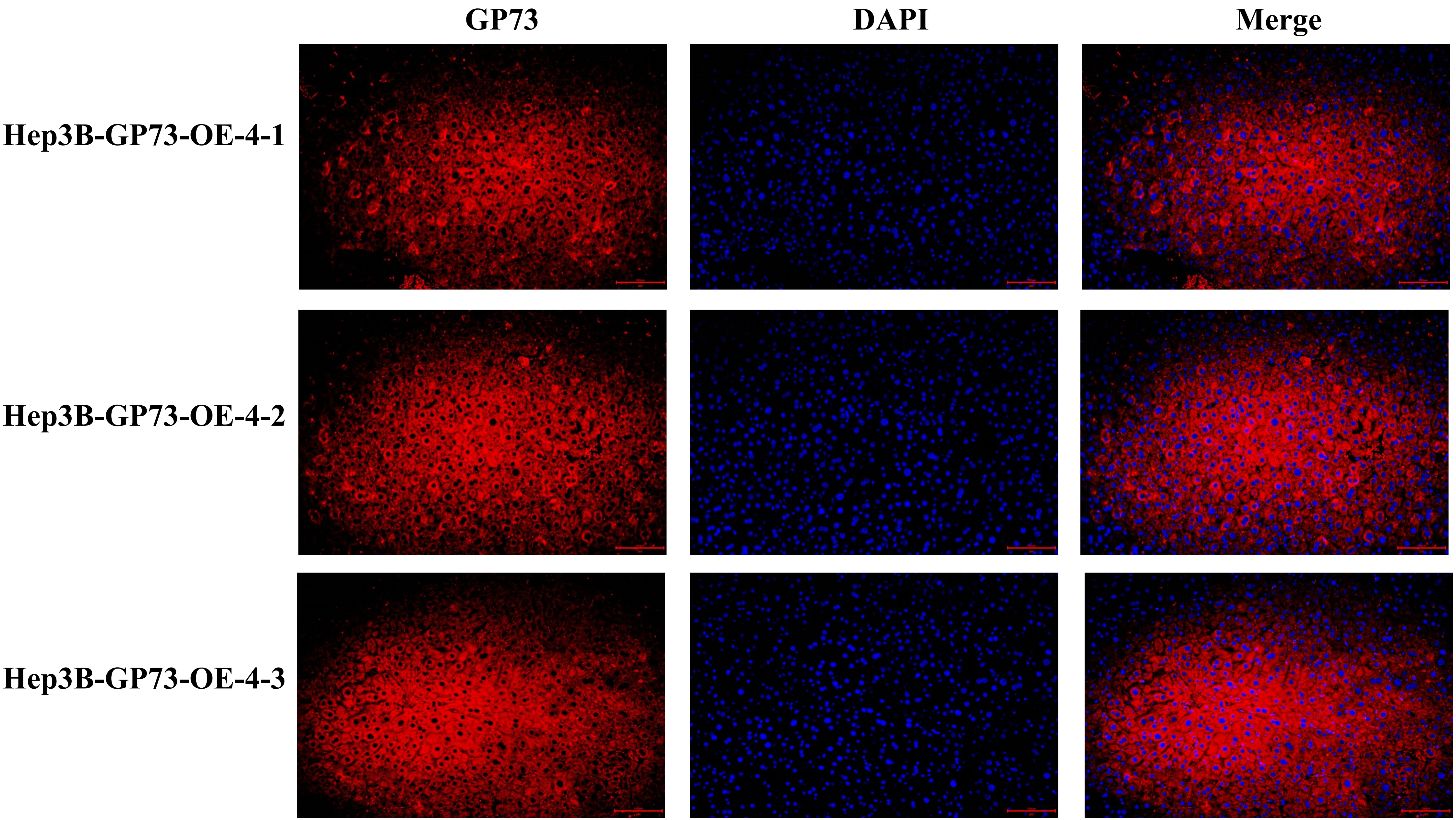

GP73
DAPI
Merge
Hep3B-GP73-OE-4-1
Hep3B-GP73-OE-4-2
Hep3B-GP73-OE-4-3

Immunofluorescence staining showing increased expressions of CD34 in resected tumors originated from the Hep3B-GP73-OE cells compared to the control, original magnification, ×20.

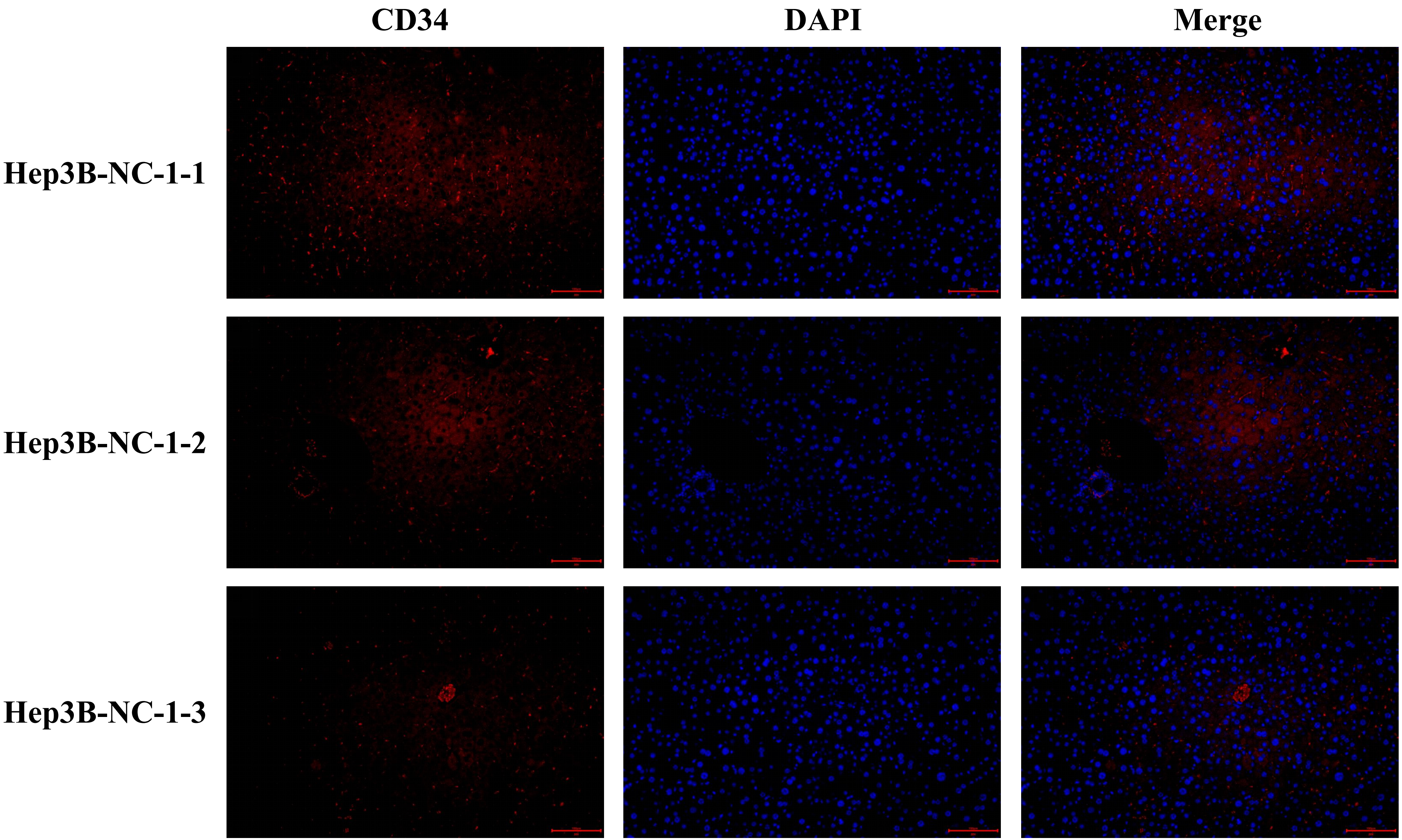
CD34
DAPI
Merge
Hep3B-NC-1-1
Hep3B-NC-1-2
Hep3B-NC-1-3

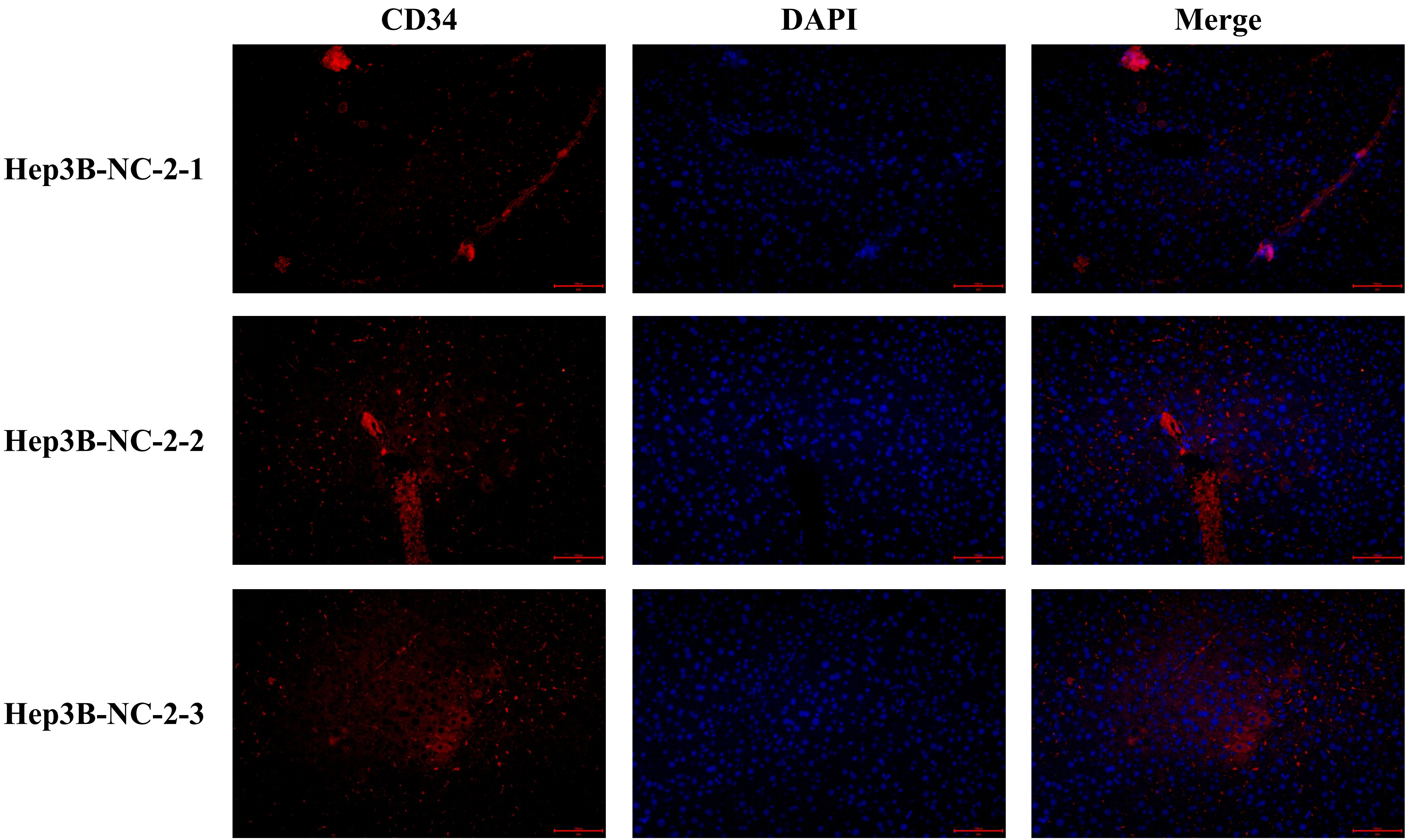
CD34
DAPI
Merge
Hep3B-NC-2-1
Hep3B-NC-2-2
Hep3B-NC-2-3

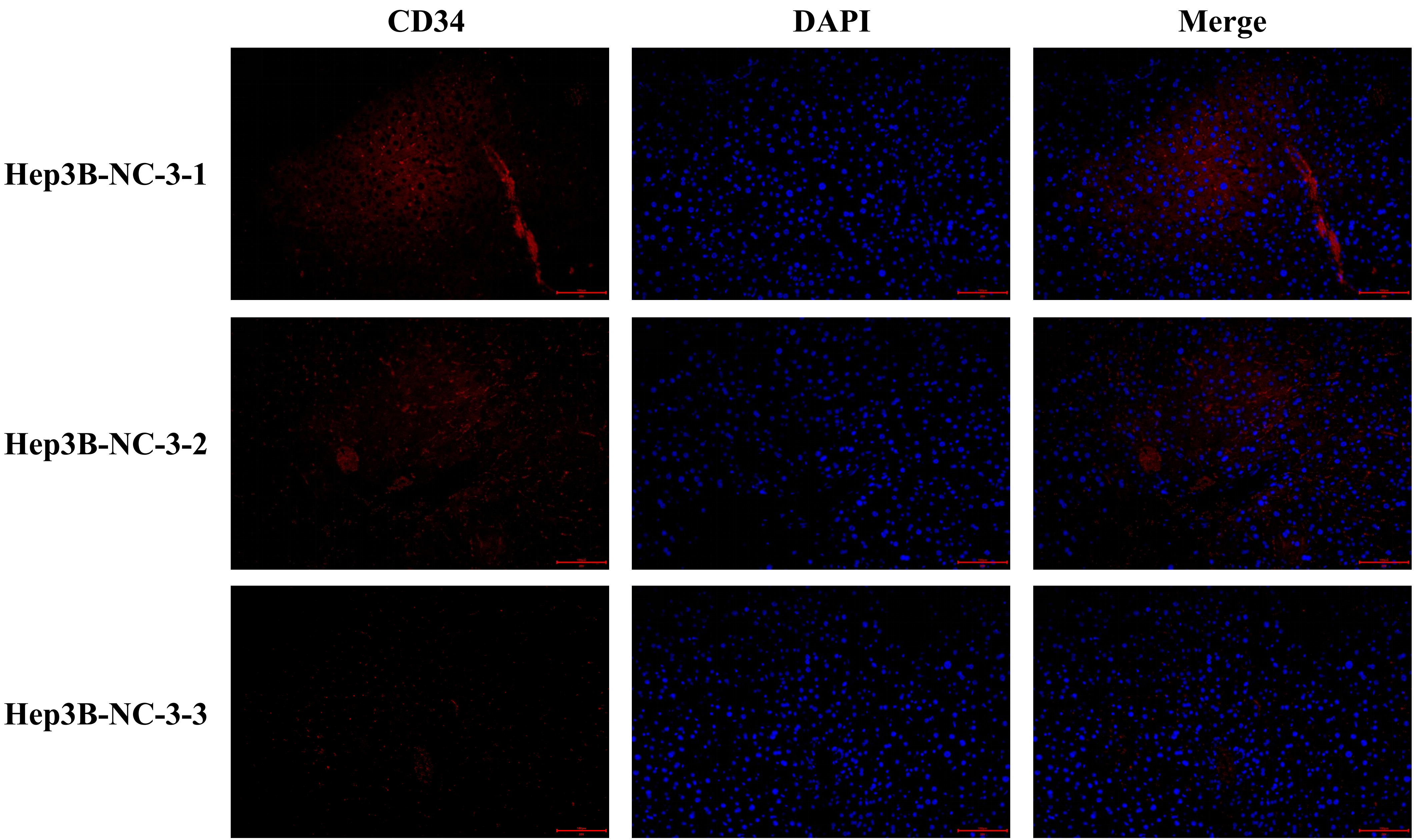
CD34
DAPI
Merge
Hep3B-NC-3-1
Hep3B-NC-3-2
Hep3B-NC-3-3

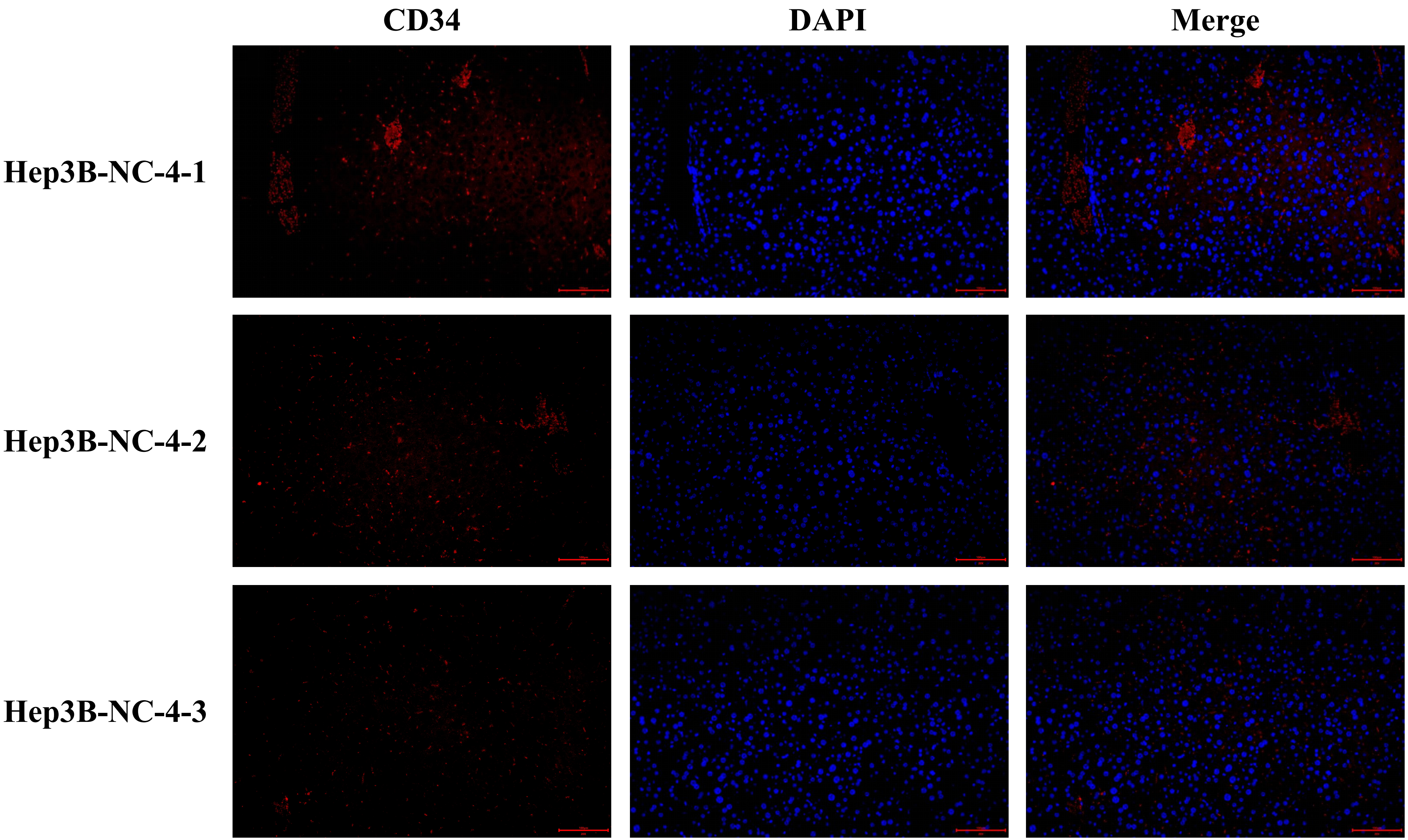
CD34
DAPI
Merge
Hep3B-NC-4-1
Hep3B-NC-4-2
Hep3B-NC-4-3

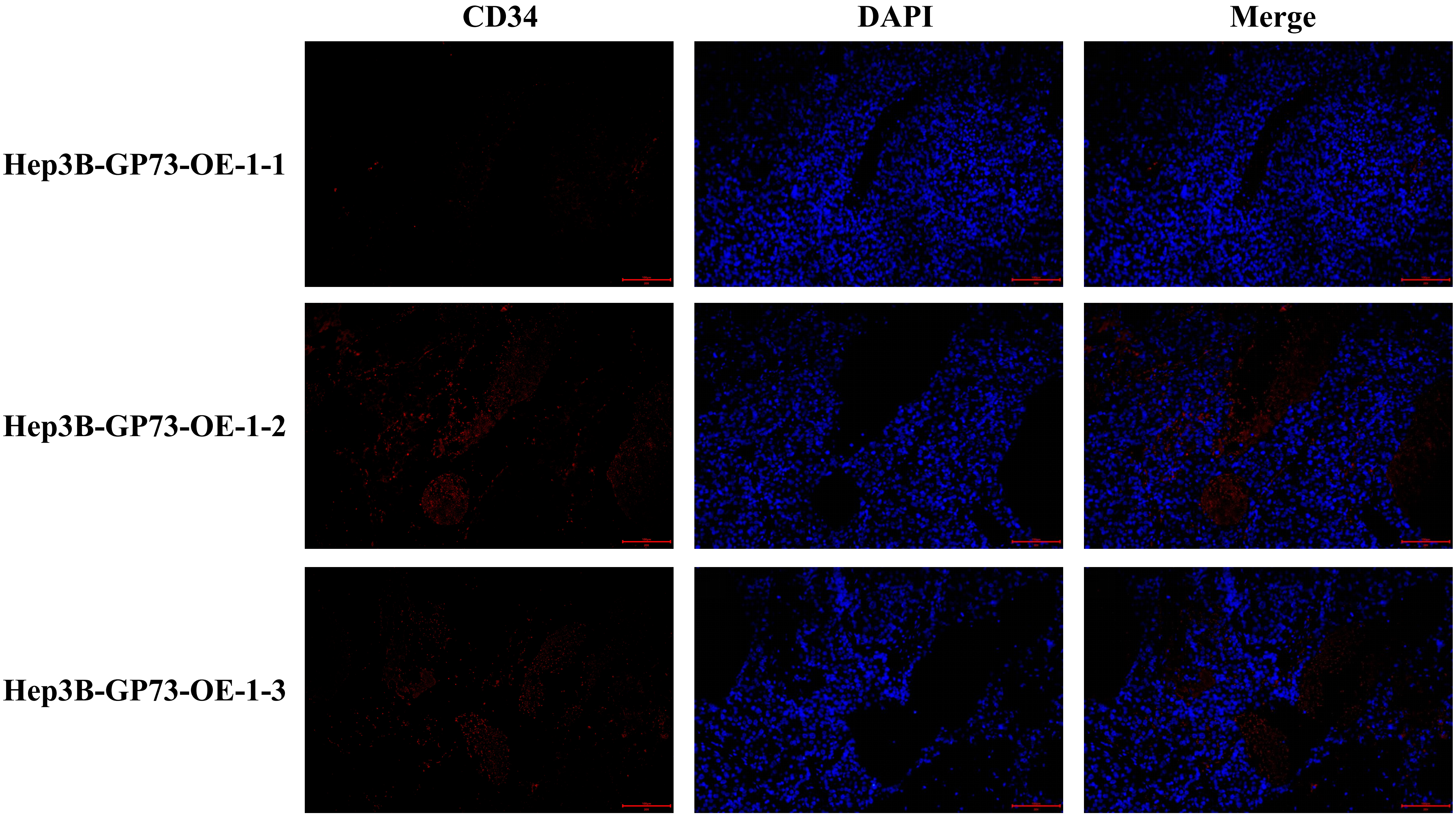
CD34
DAPI
Merge
Hep3B-GP73-OE-1-1
Hep3B-GP73-OE-1-2
Hep3B-GP73-OE-1-3

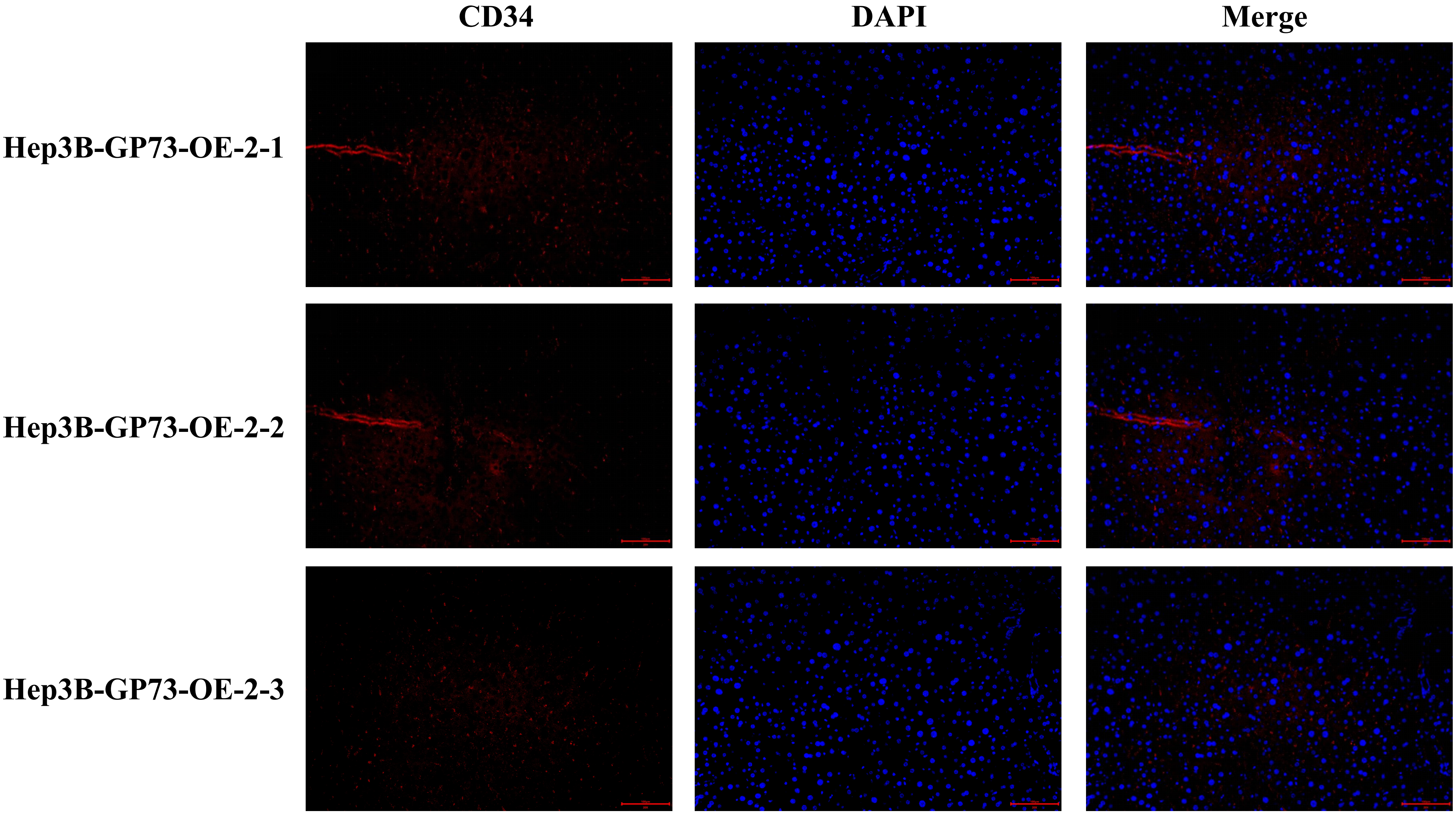
CD34
DAPI
Merge
Hep3B-GP73-OE-2-1
Hep3B-GP73-OE-2-2
Hep3B-GP73-OE-2-3

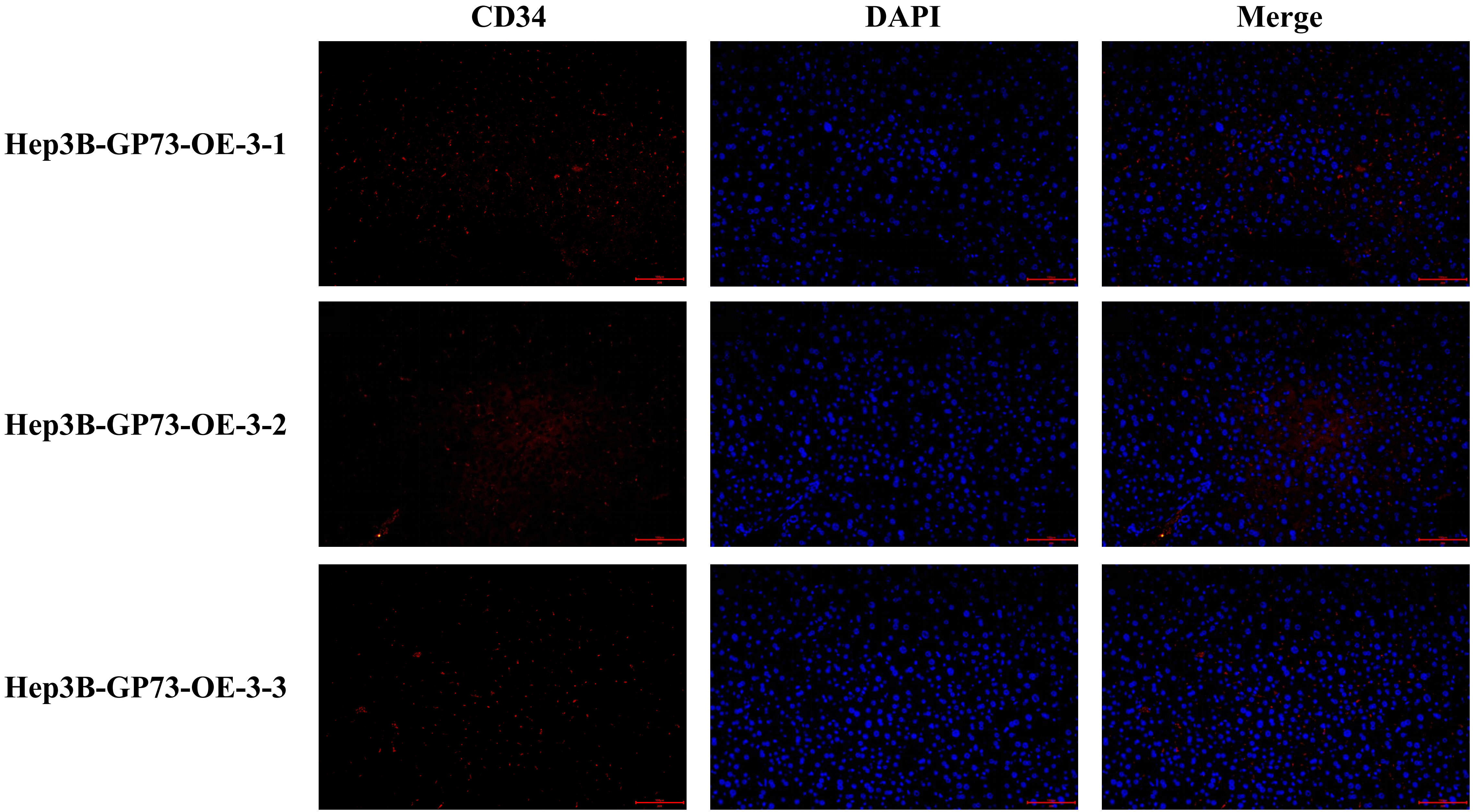
CD34
DAPI
Merge
Hep3B-GP73-OE-3-1
Hep3B-GP73-OE-3-2
Hep3B-GP73-OE-3-3

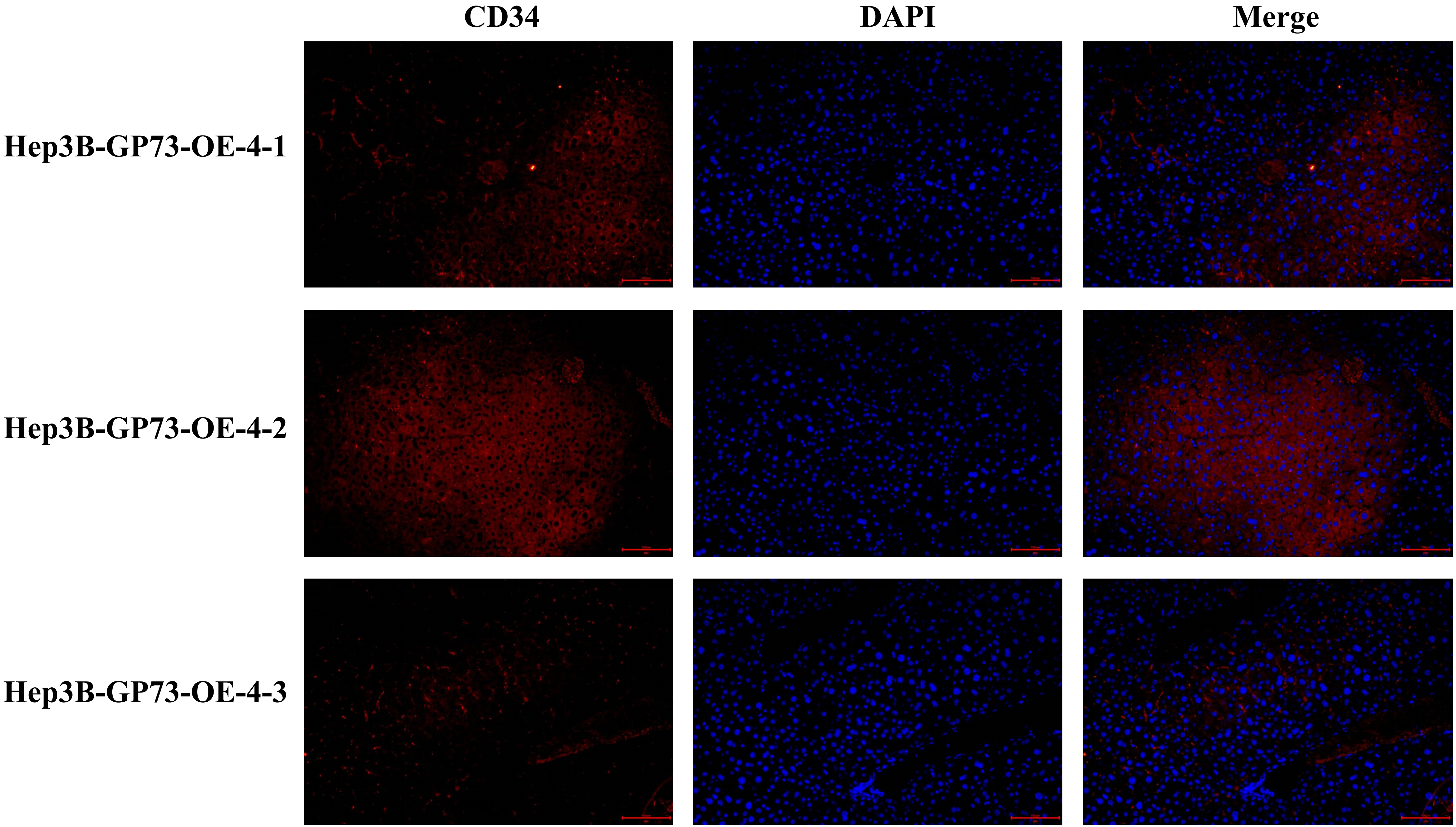
CD34
DAPI
Merge
Hep3B-GP73-OE-4-1
Hep3B-GP73-OE-4-2
Hep3B-GP73-OE-4-3

Immunofluorescence staining showing increased expressions of CD31 in resected tumors originated from the Hep3B-GP73-OE cells compared to the control, original magnification, ×20.

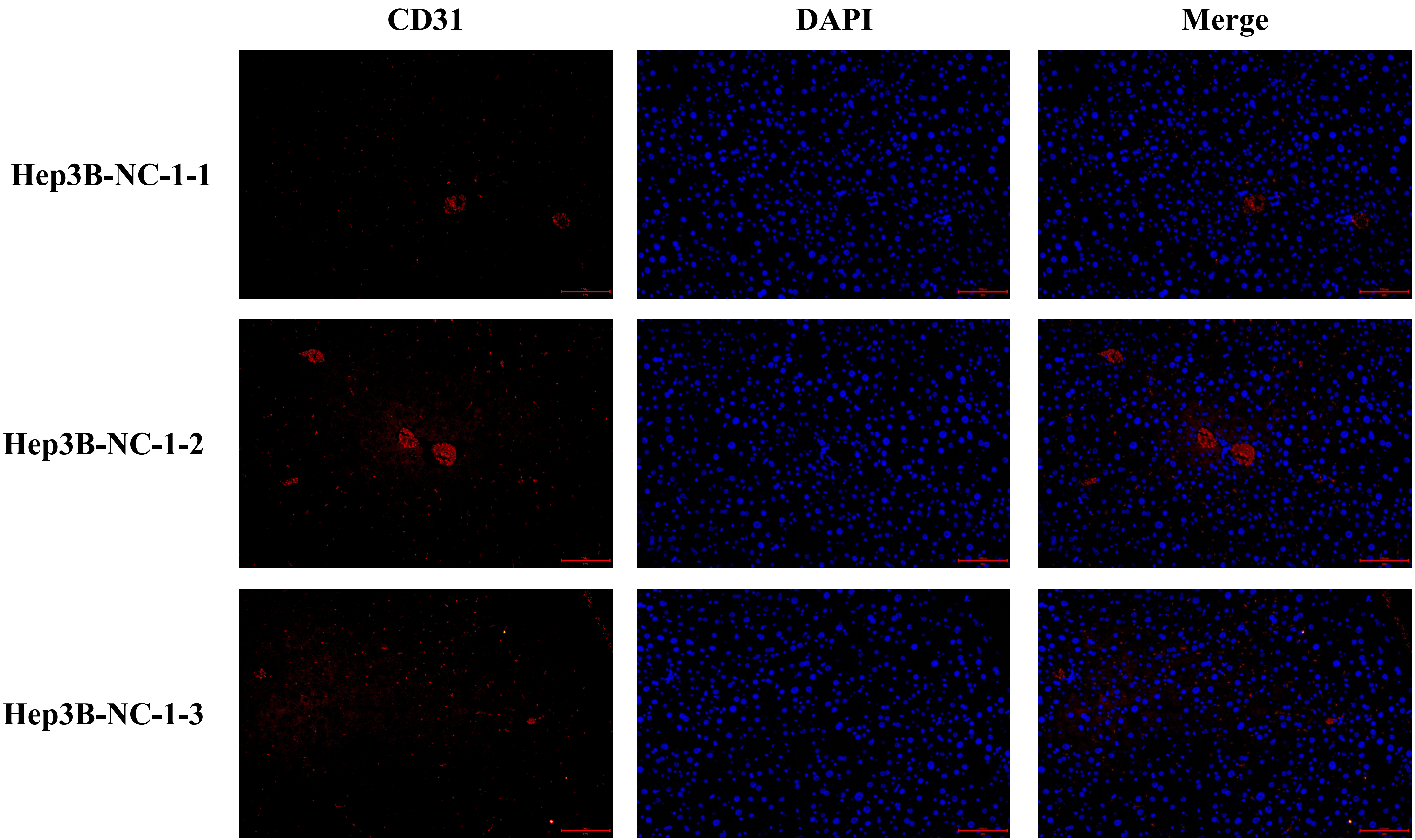
CD31
DAPI
Merge
Hep3B-NC-1-1
Hep3B-NC-1-2
Hep3B-NC-1-3

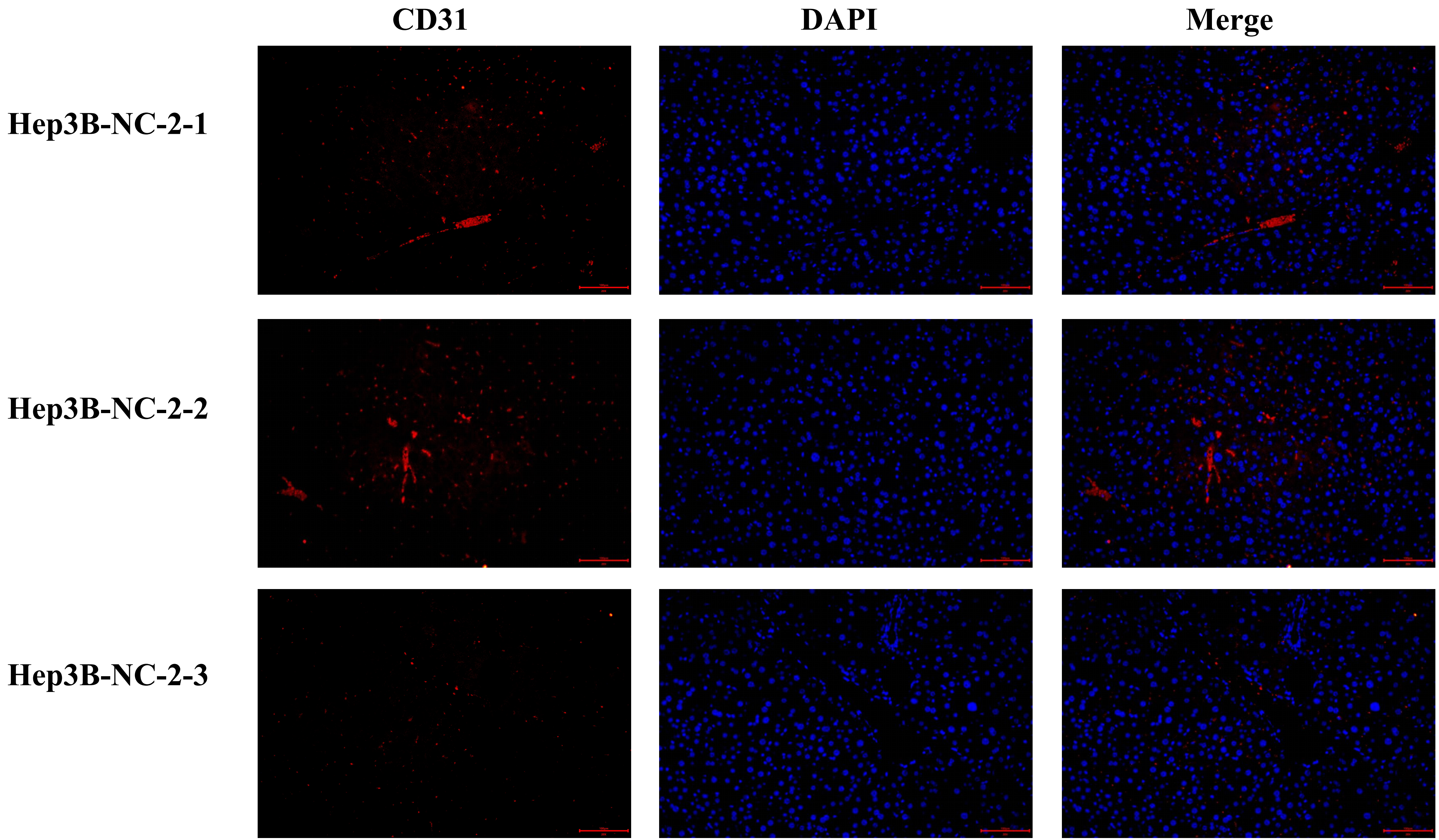
CD31
DAPI
Merge
Hep3B-NC-2-1
Hep3B-NC-2-2
Hep3B-NC-2-3

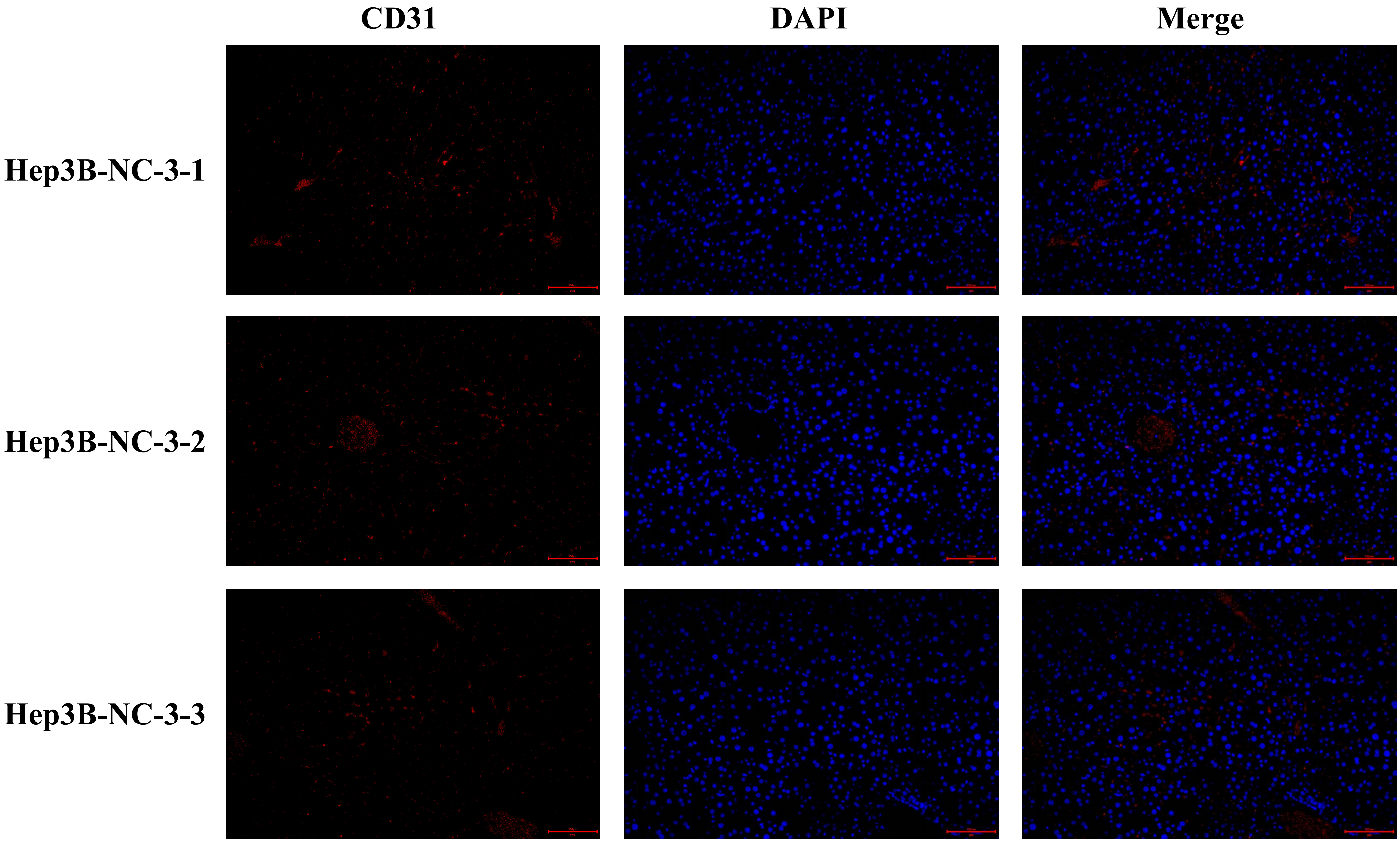
CD31
DAPI
Merge
Hep3B-NC-3-1
Hep3B-NC-3-2
Hep3B-NC-3-3

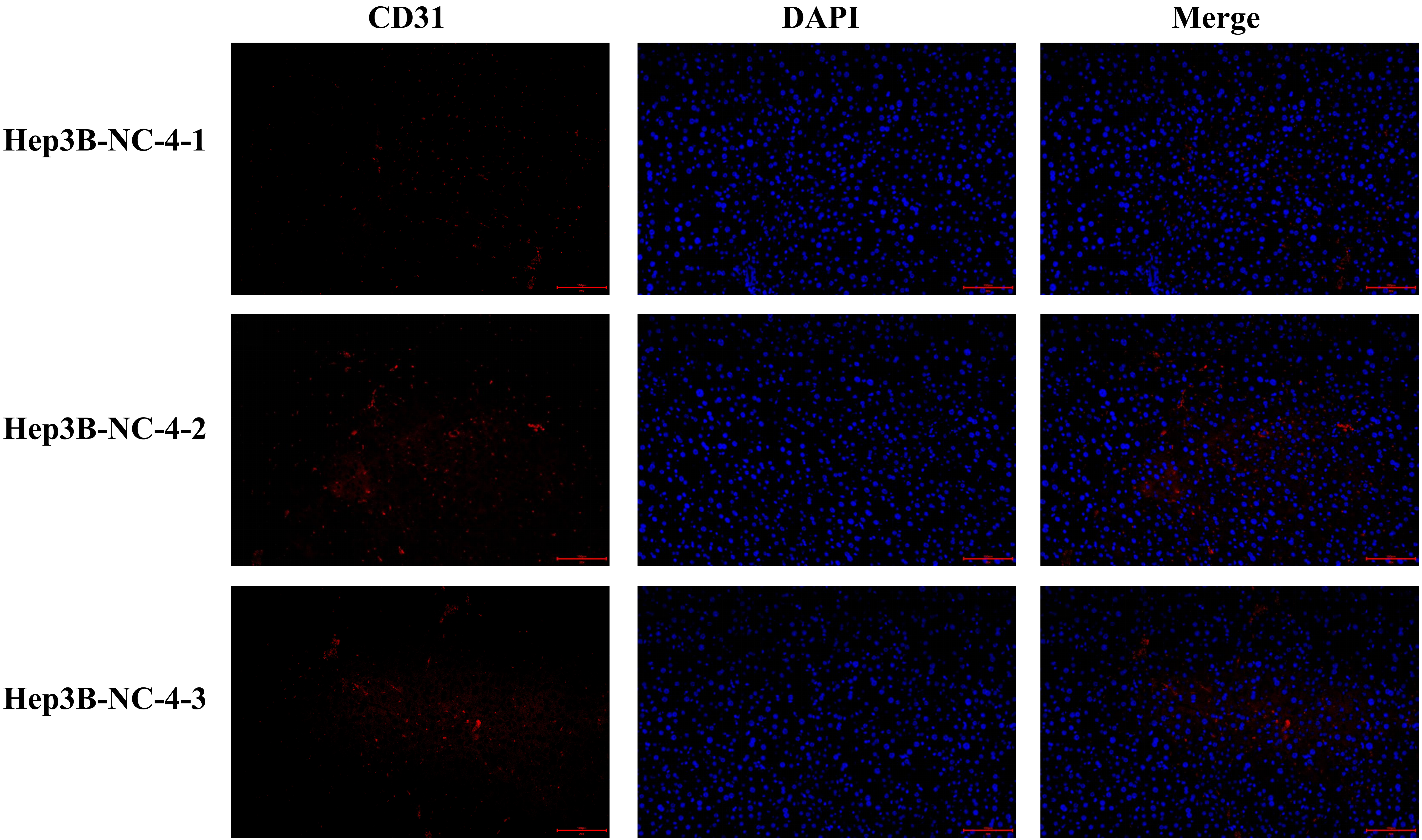
CD31
DAPI
Merge
Hep3B-NC-4-1
Hep3B-NC-4-2
Hep3B-NC-4-3

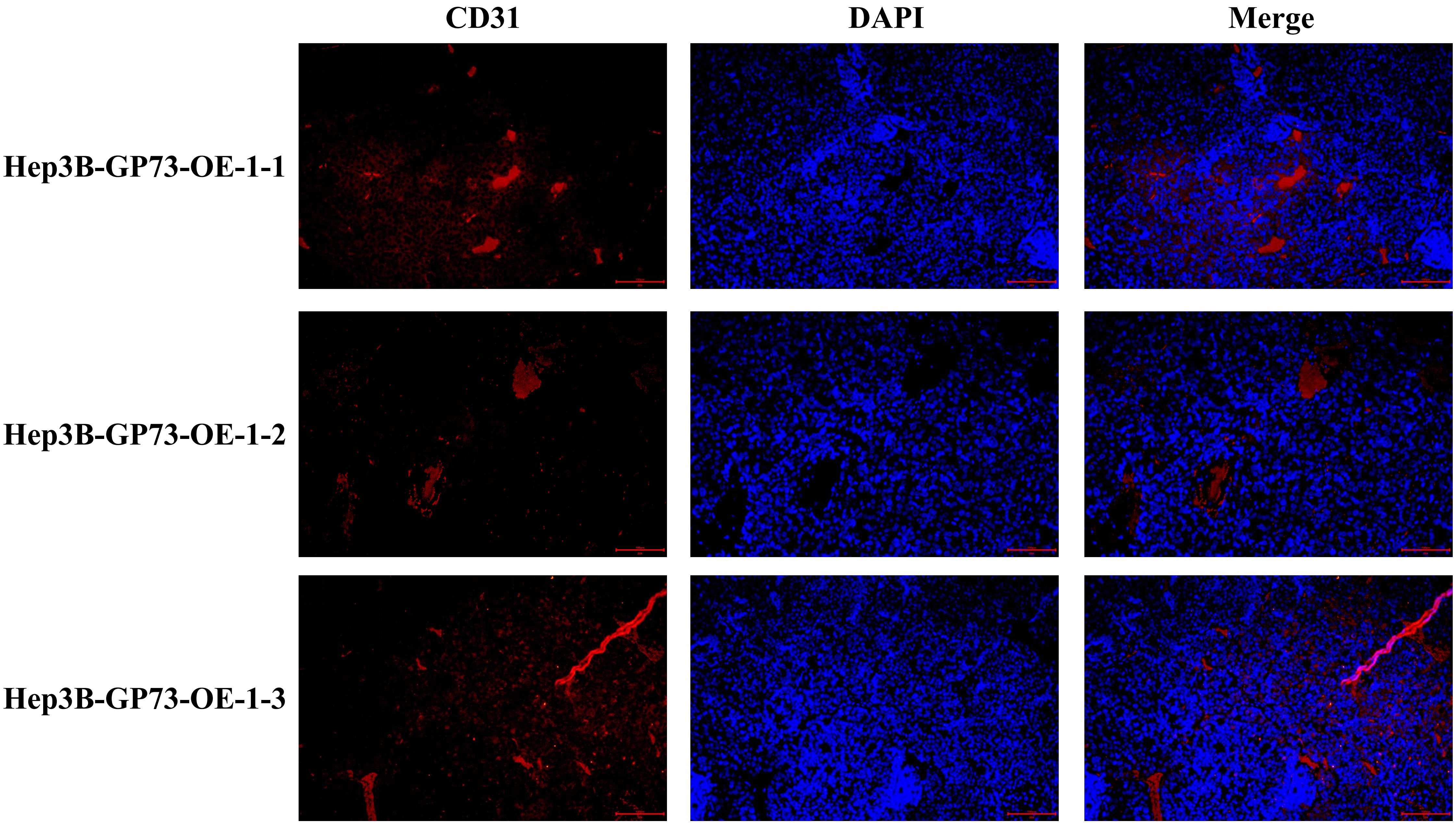
CD31
DAPI
Merge
Hep3B-GP73-OE-1-1
Hep3B-GP73-OE-1-2
Hep3B-GP73-OE-1-3

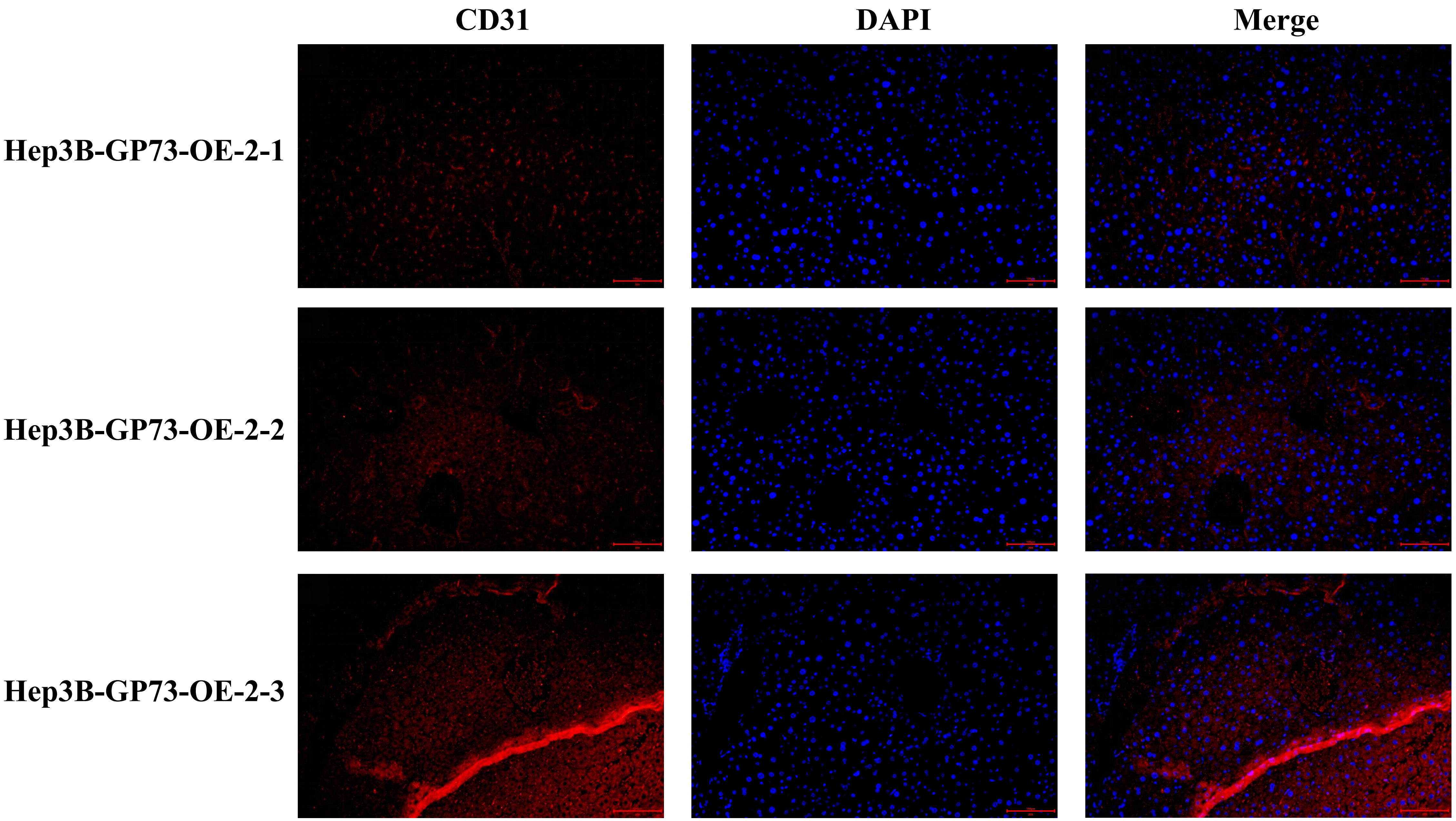
CD31
DAPI
Merge
Hep3B-GP73-OE-2-1
Hep3B-GP73-OE-2-2
Hep3B-GP73-OE-2-3

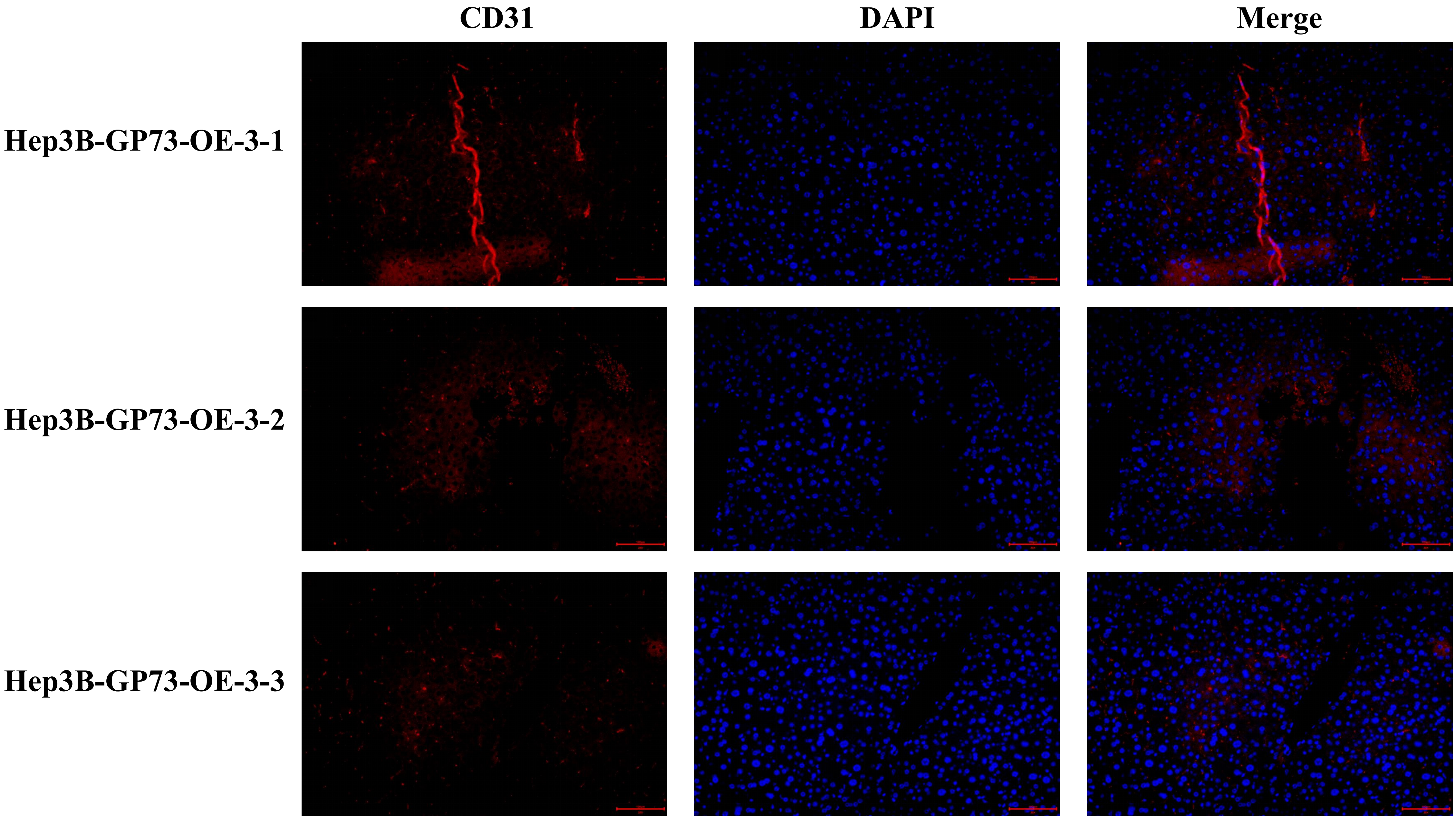
CD31
DAPI
Merge
Hep3B-GP73-OE-3-1
Hep3B-GP73-OE-3-2
Hep3B-GP73-OE-3-3

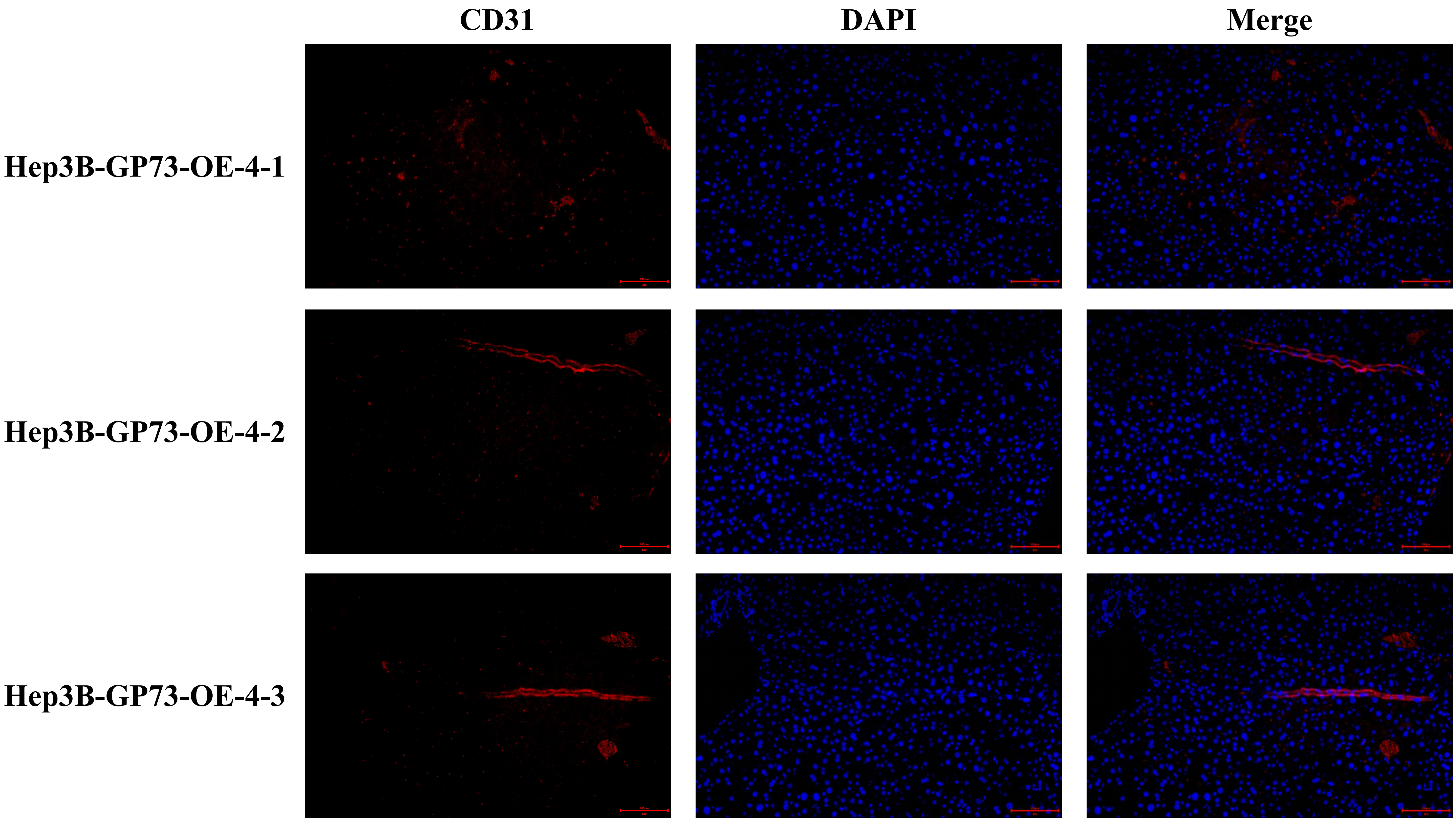
CD31
DAPI
Merge
Hep3B-GP73-OE-4-1
Hep3B-GP73-OE-4-2
Hep3B-GP73-OE-4-3

Figure 4I

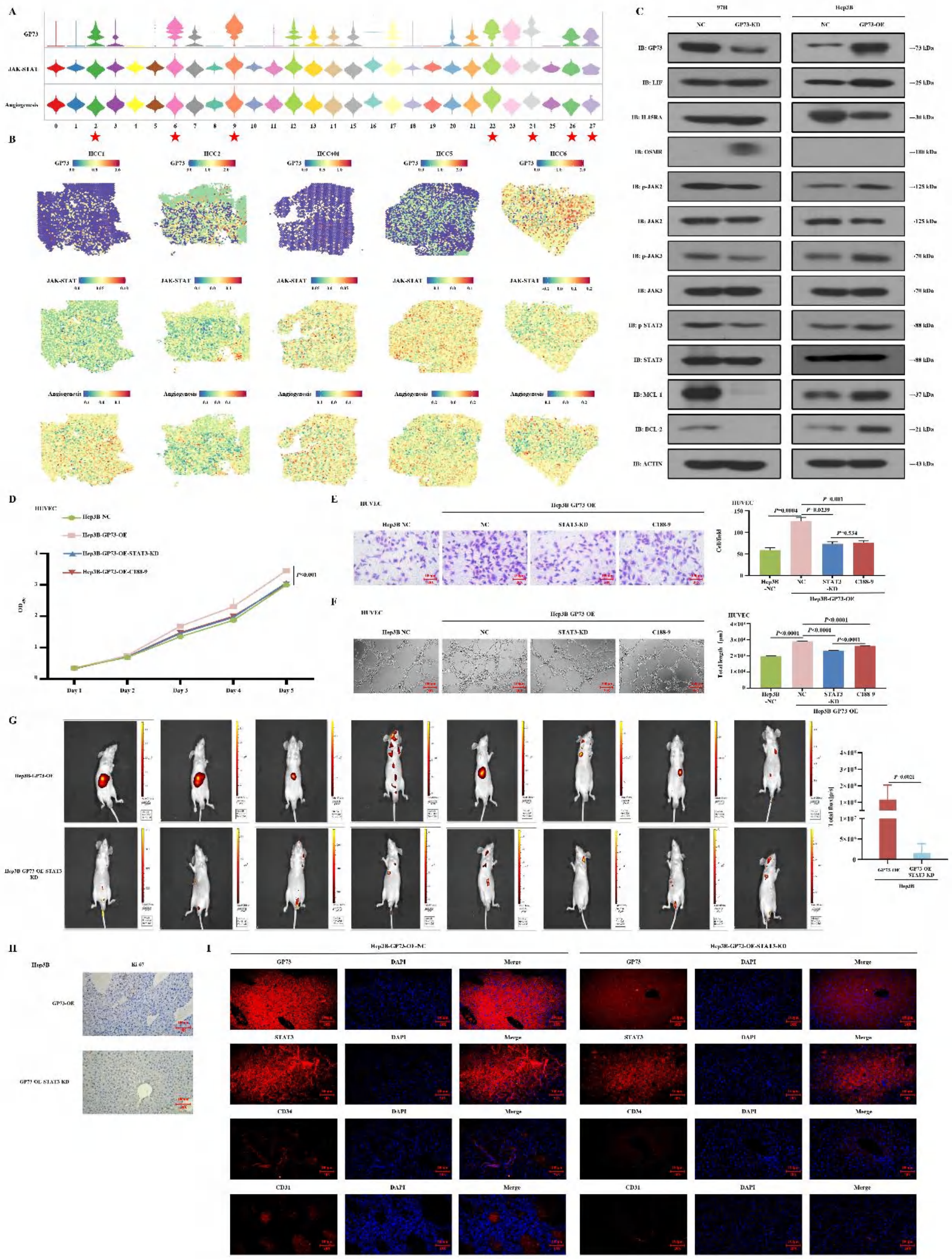

Immunofluorescence staining showing decreased expressions of GP73 in resected tumors originating from the Hep3B-GP73-OE-STAT3-KD cells compared to the Hep3B-GP73-OE cells, original magnification, ×20.

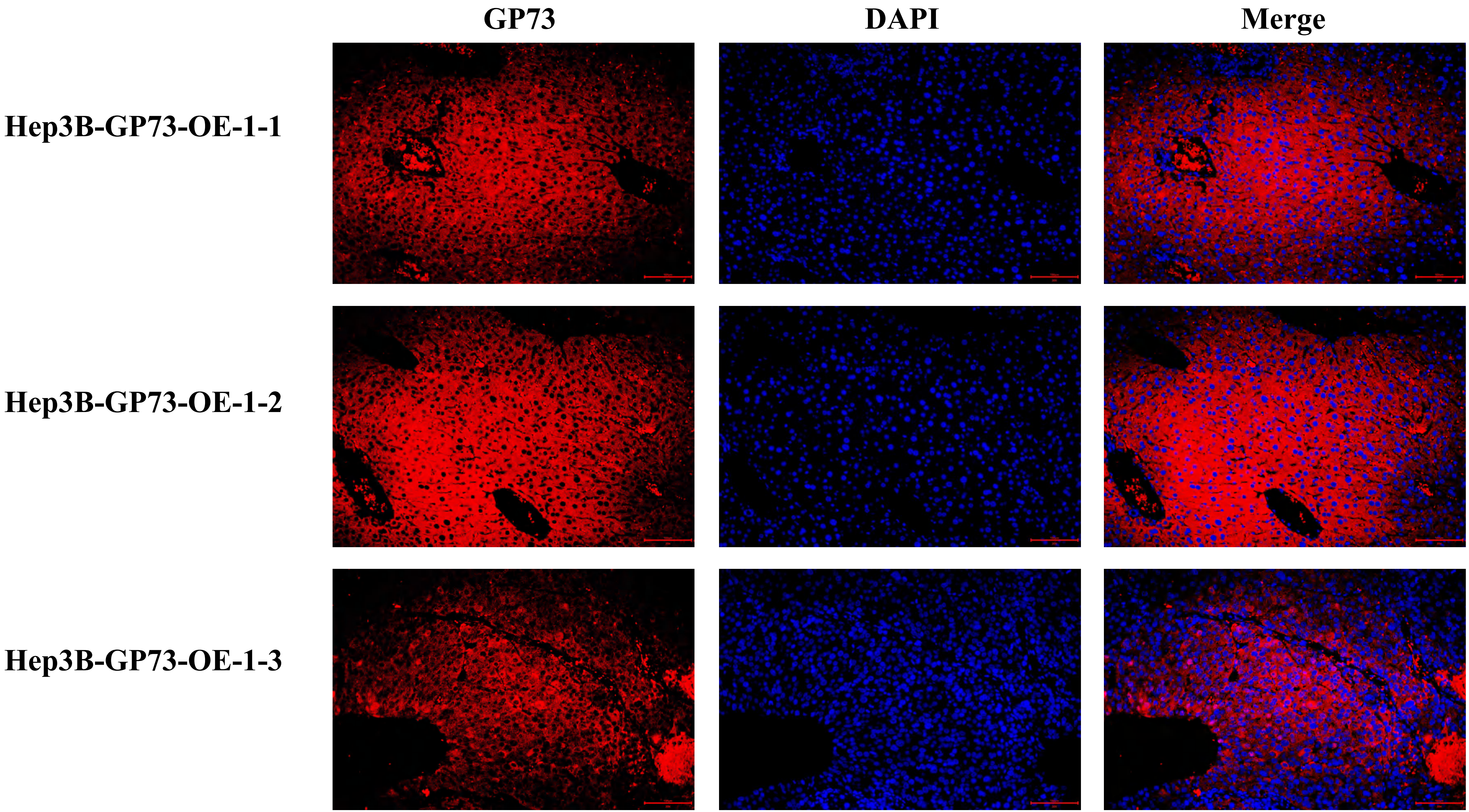
GP73
DAPI
Merge
Hep3B-GP73-OE-1-1
Hep3B-GP73-OE-1-2
Hep3B-GP73-OE-1-3

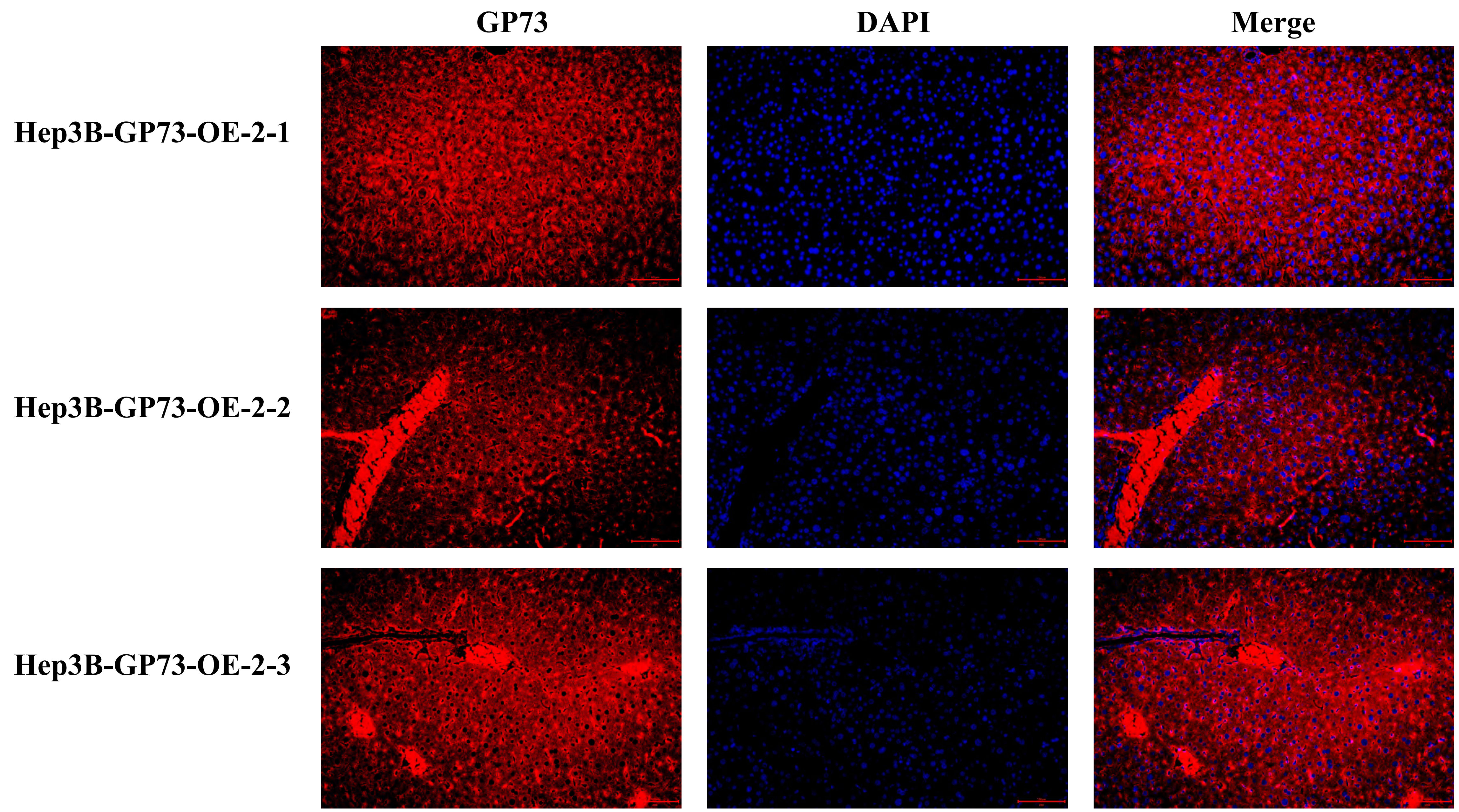
GP73
DAPI
Merge
Hep3B-GP73-OE-2-1
Hep3B-GP73-OE-2-2
Hep3B-GP73-OE-2-3

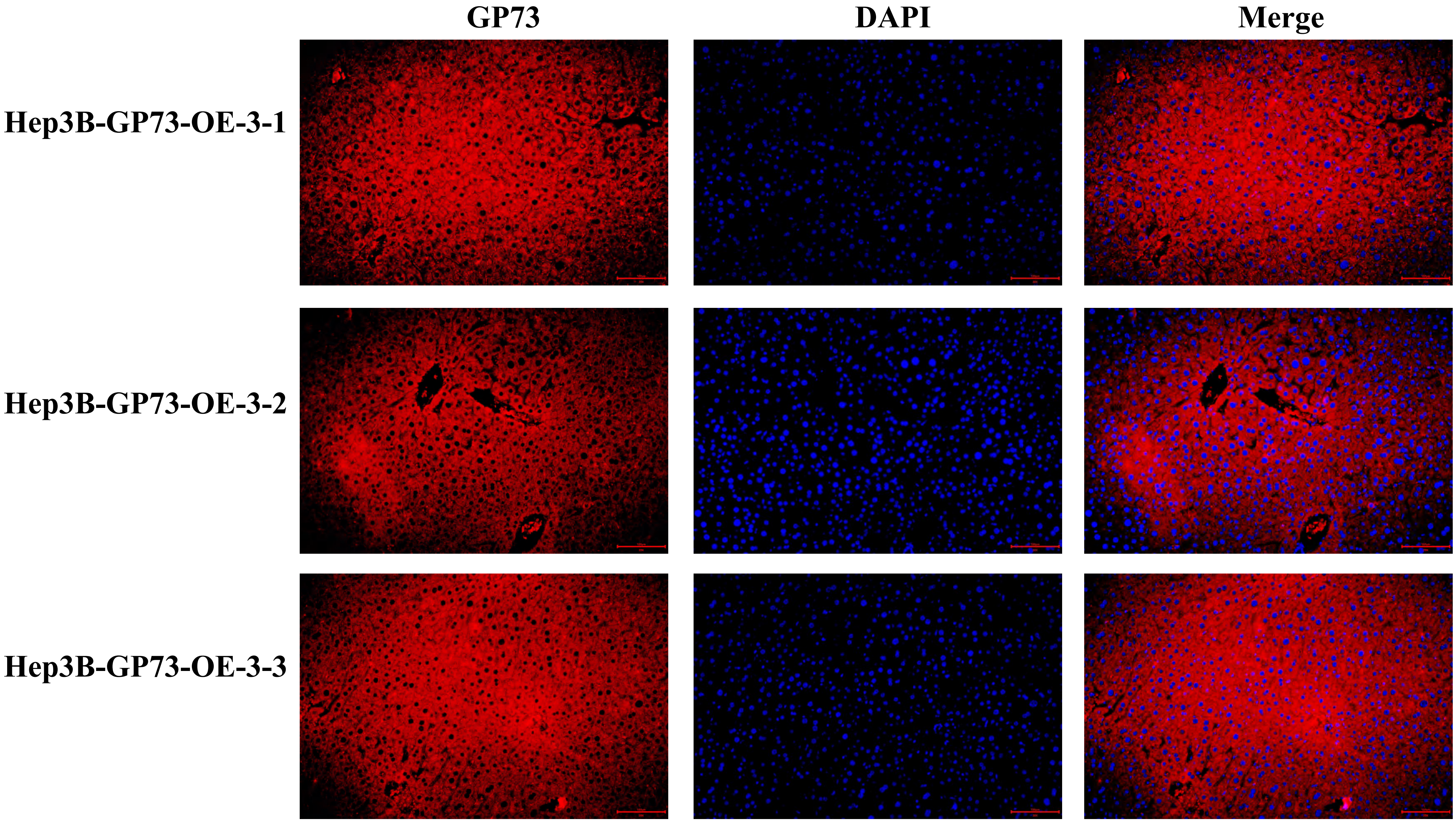
GP73
DAPI
Merge
Hep3B-GP73-OE-3-1
Hep3B-GP73-OE-3-2
Hep3B-GP73-OE-3-3

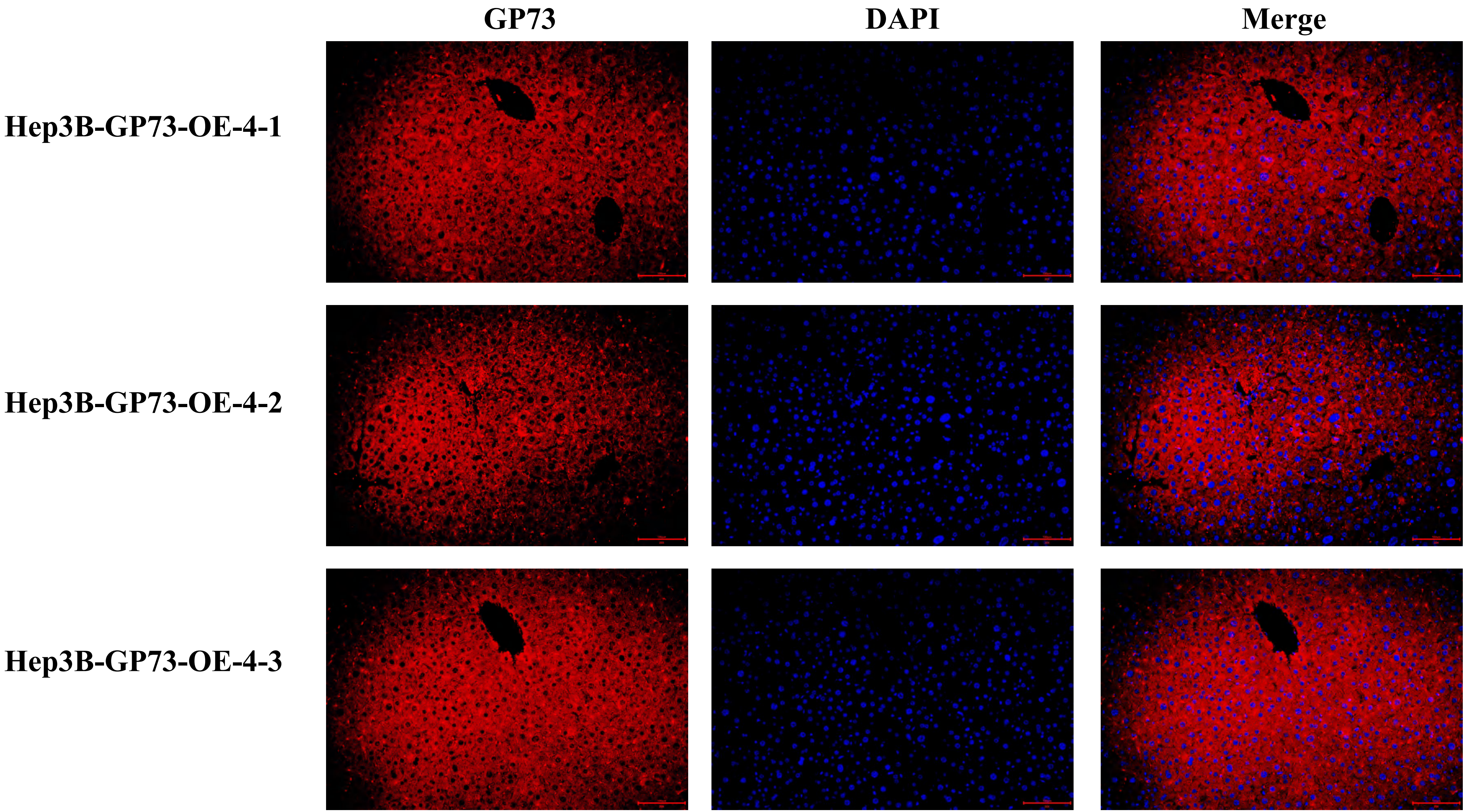
GP73
DAPI
Merge
Hep3B-GP73-OE-4-1
Hep3B-GP73-OE-4-2
Hep3B-GP73-OE-4-3

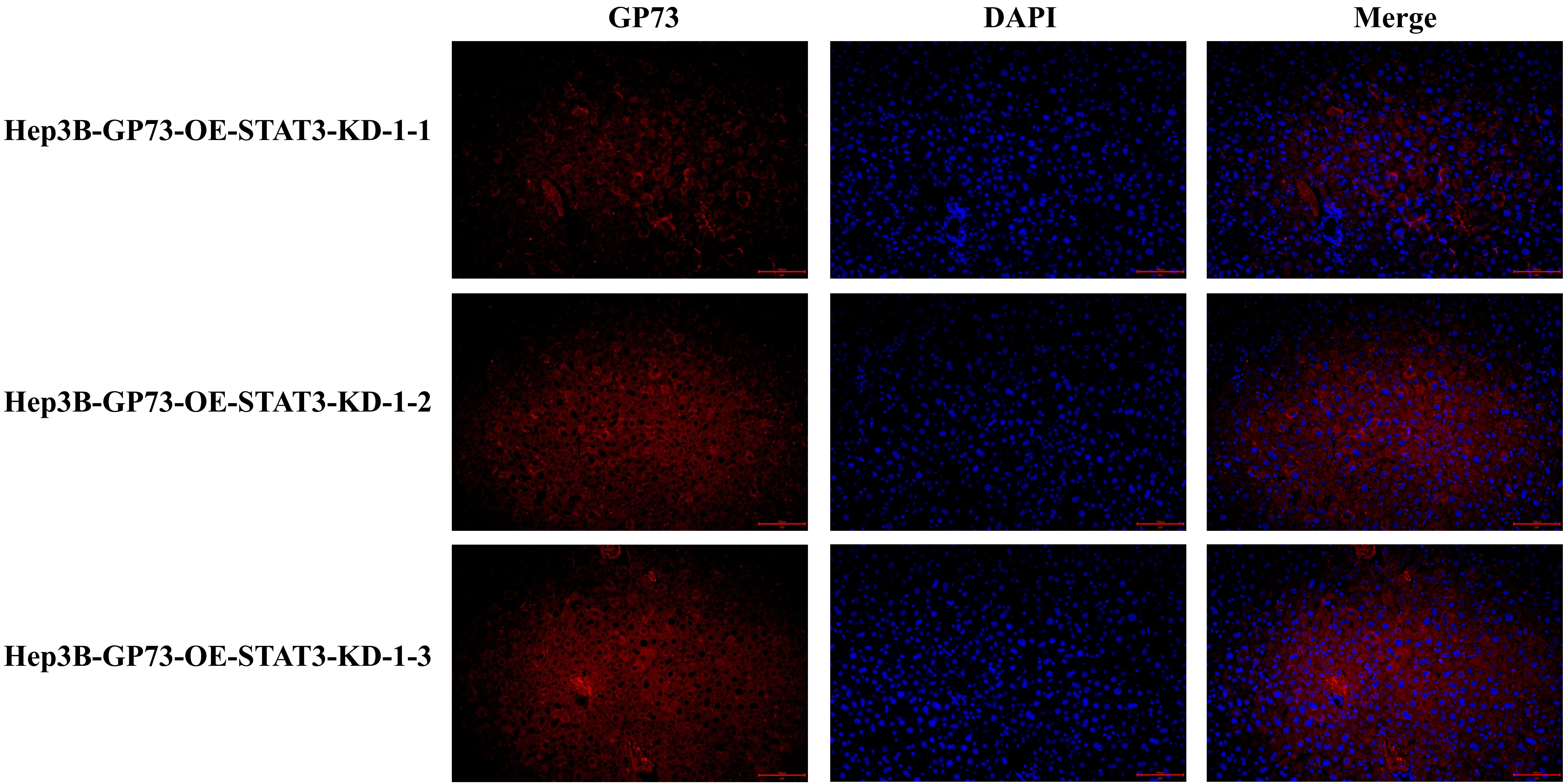
GP73
DAPI
Merge
Hep3B-GP73-OE-STAT3-KD-1-1
Hep3B-GP73-OE-STAT3-KD-1-2
Hep3B-GP73-OE-STAT3-KD-1-3

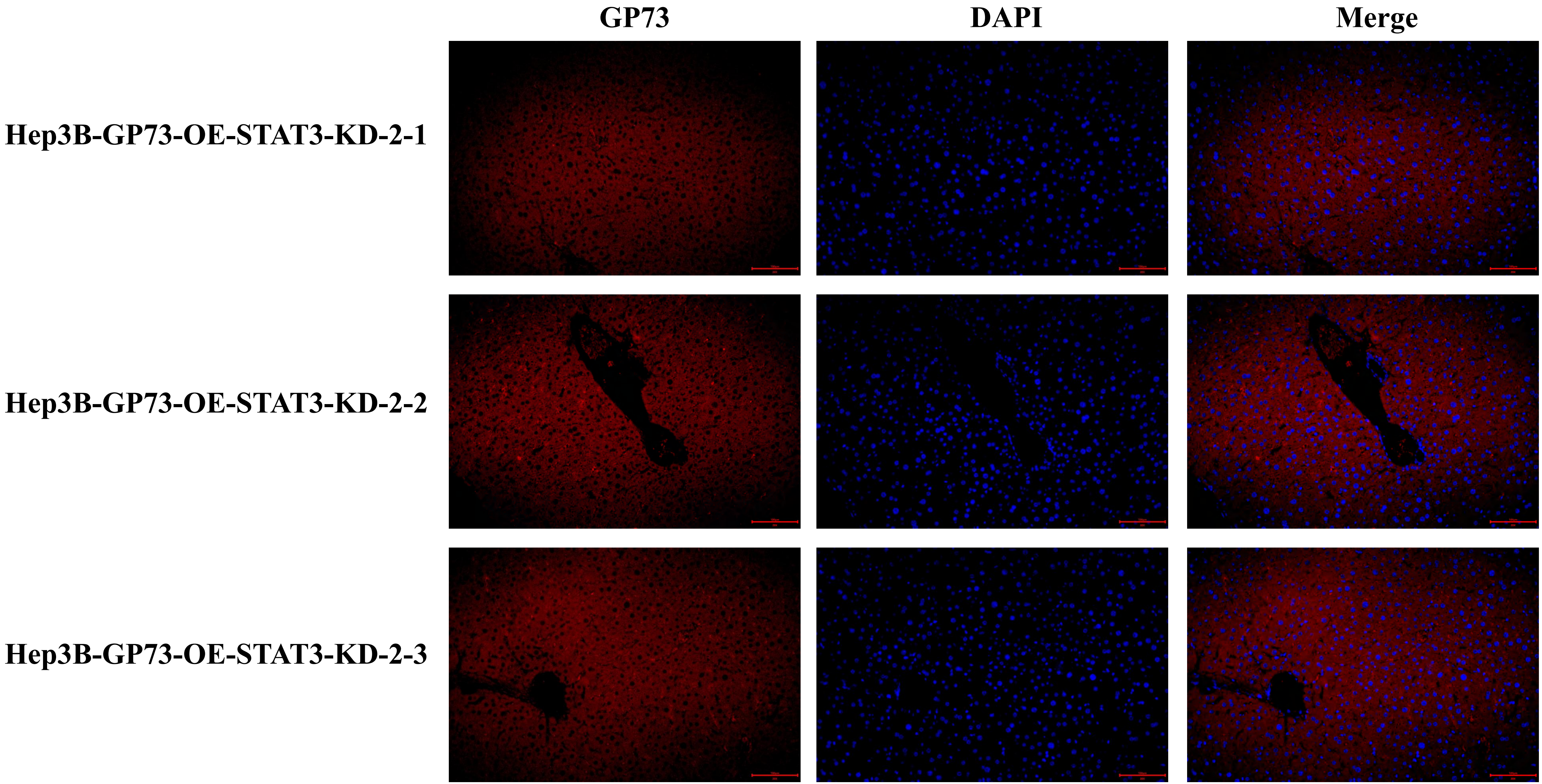
GP73
DAPI
Merge
Hep3B-GP73-OE-STAT3-KD-2-1
Hep3B-GP73-OE-STAT3-KD-2-2
Hep3B-GP73-OE-STAT3-KD-2-3

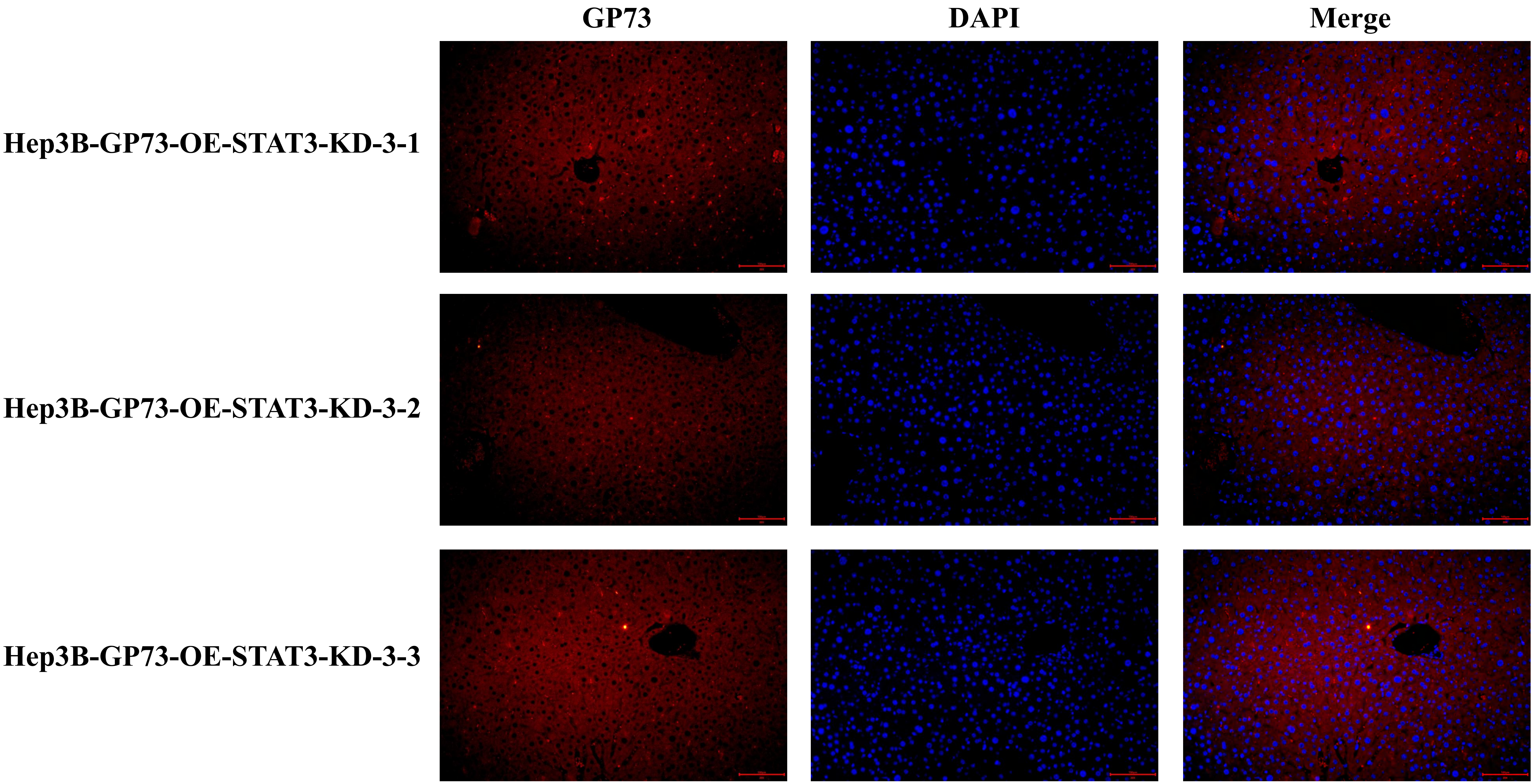
GP73
DAPI
Merge
Hep3B-GP73-OE-STAT3-KD-3-1
Hep3B-GP73-OE-STAT3-KD-3-2
Hep3B-GP73-OE-STAT3-KD-3-3

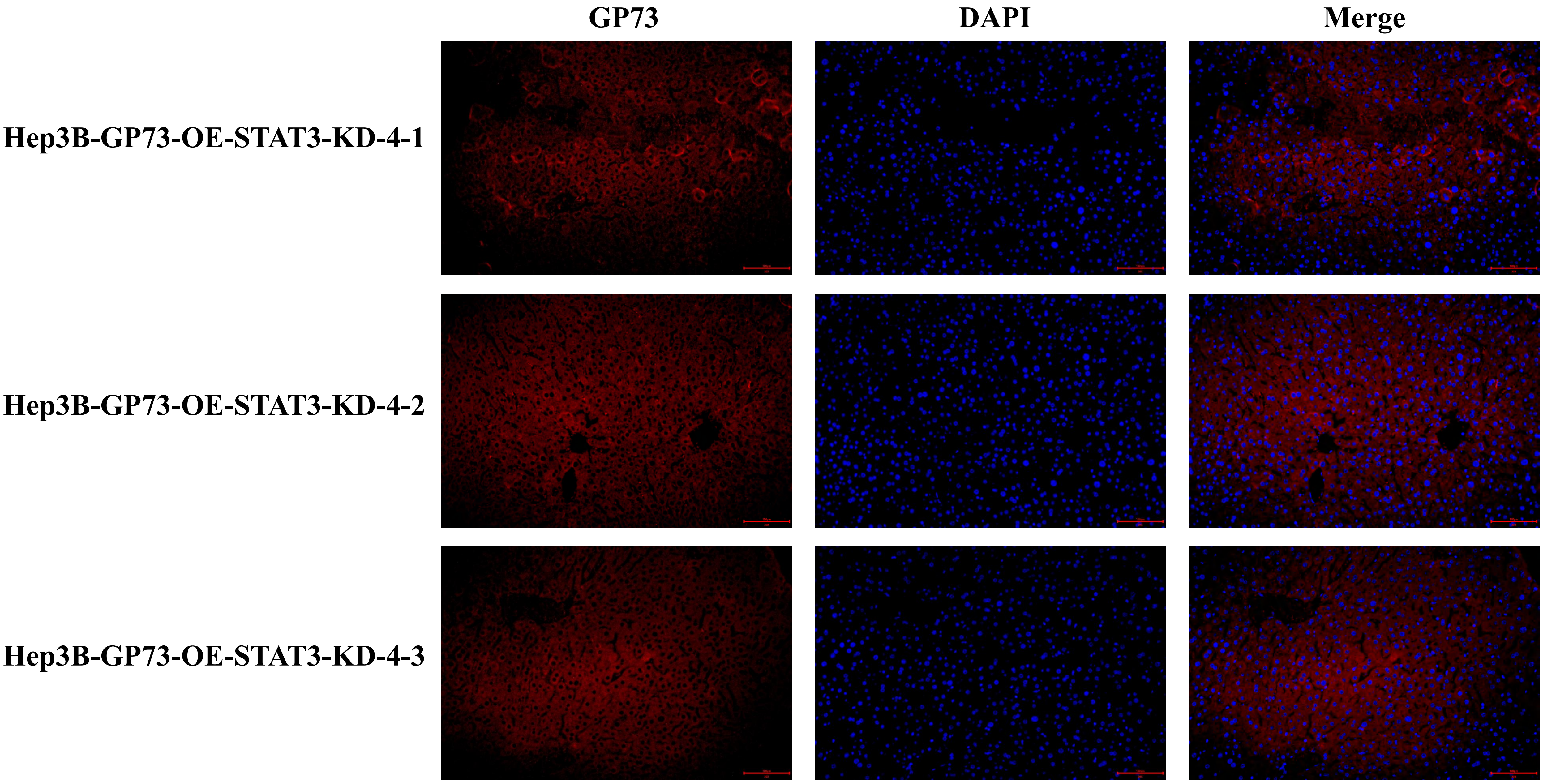
GP73
DAPI
Merge
Hep3B-GP73-OE-STAT3-KD-4-1
Hep3B-GP73-OE-STAT3-KD-4-2
Hep3B-GP73-OE-STAT3-KD-4-3

Immunofluorescence staining showing decreased expressions of STAT3 in resected tumors originating from the Hep3B-GP73-OE-STAT3-KD cells compared to the Hep3B-GP73-OE cells, original magnification, ×20.

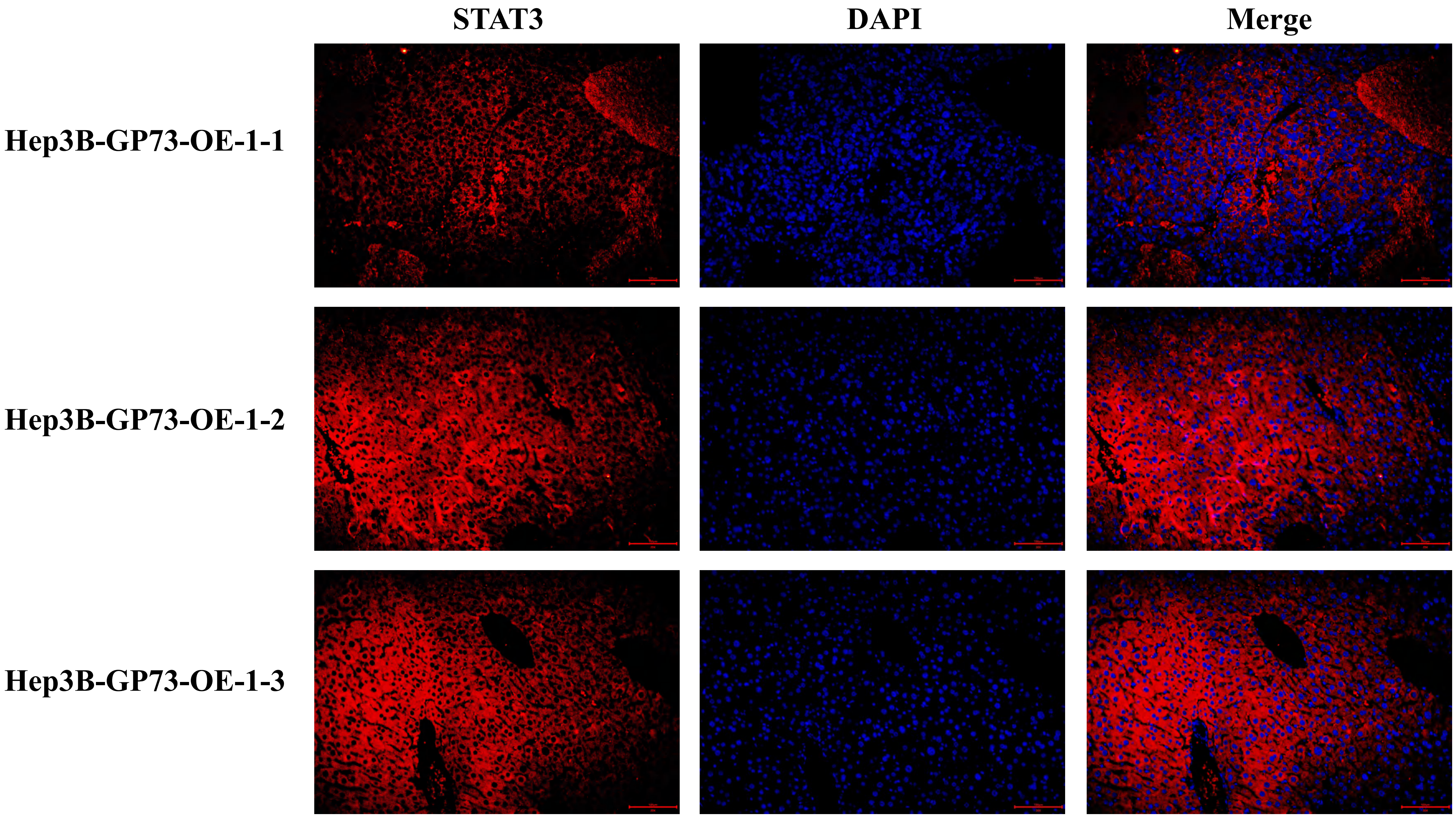
STAT3
DAPI
Merge
Hep3B-GP73-OE-1-1
Hep3B-GP73-OE-1-2
Hep3B-GP73-OE-1-3

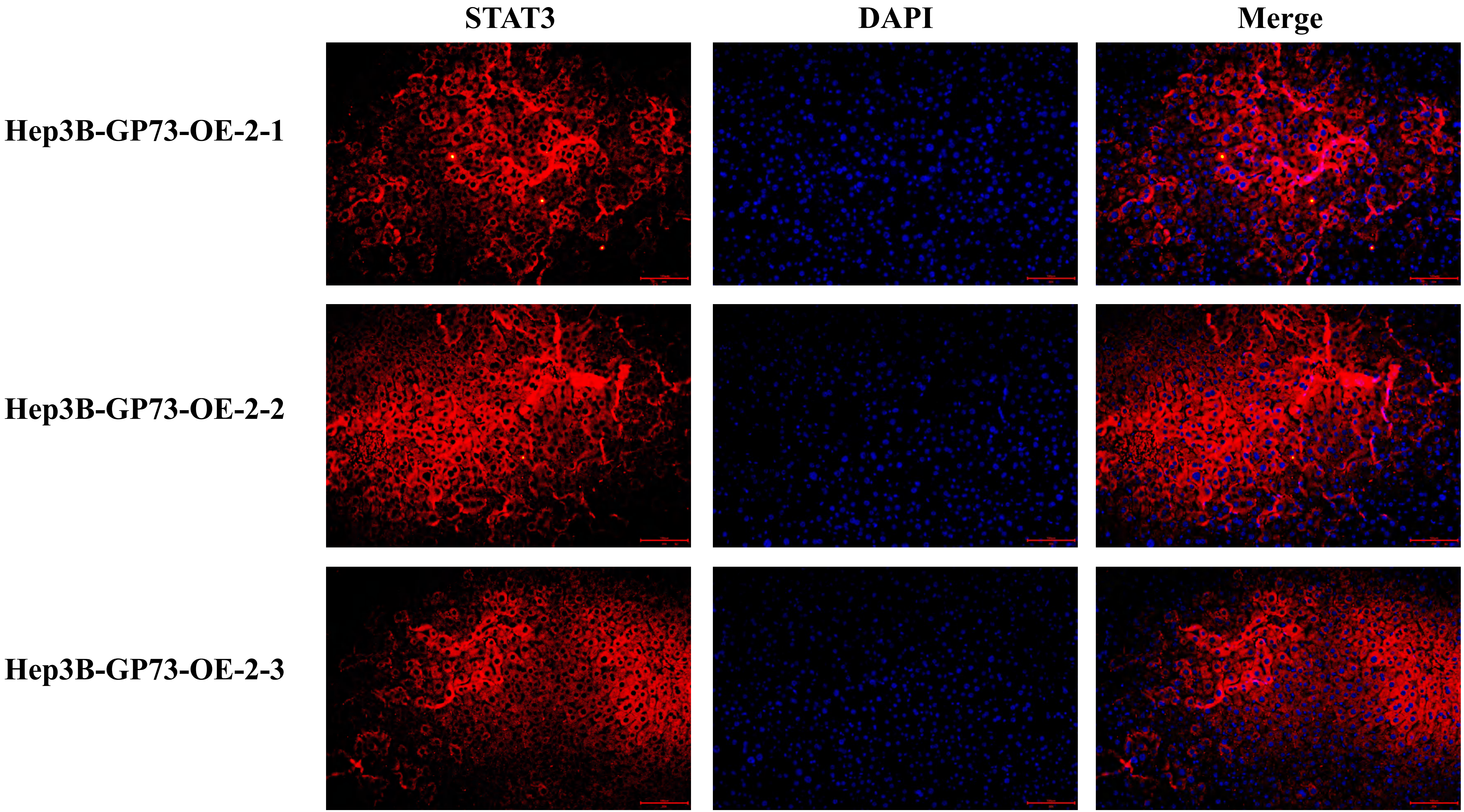
STAT3
DAPI
Merge
Hep3B-GP73-OE-2-1
Hep3B-GP73-OE-2-2
Hep3B-GP73-OE-2-3

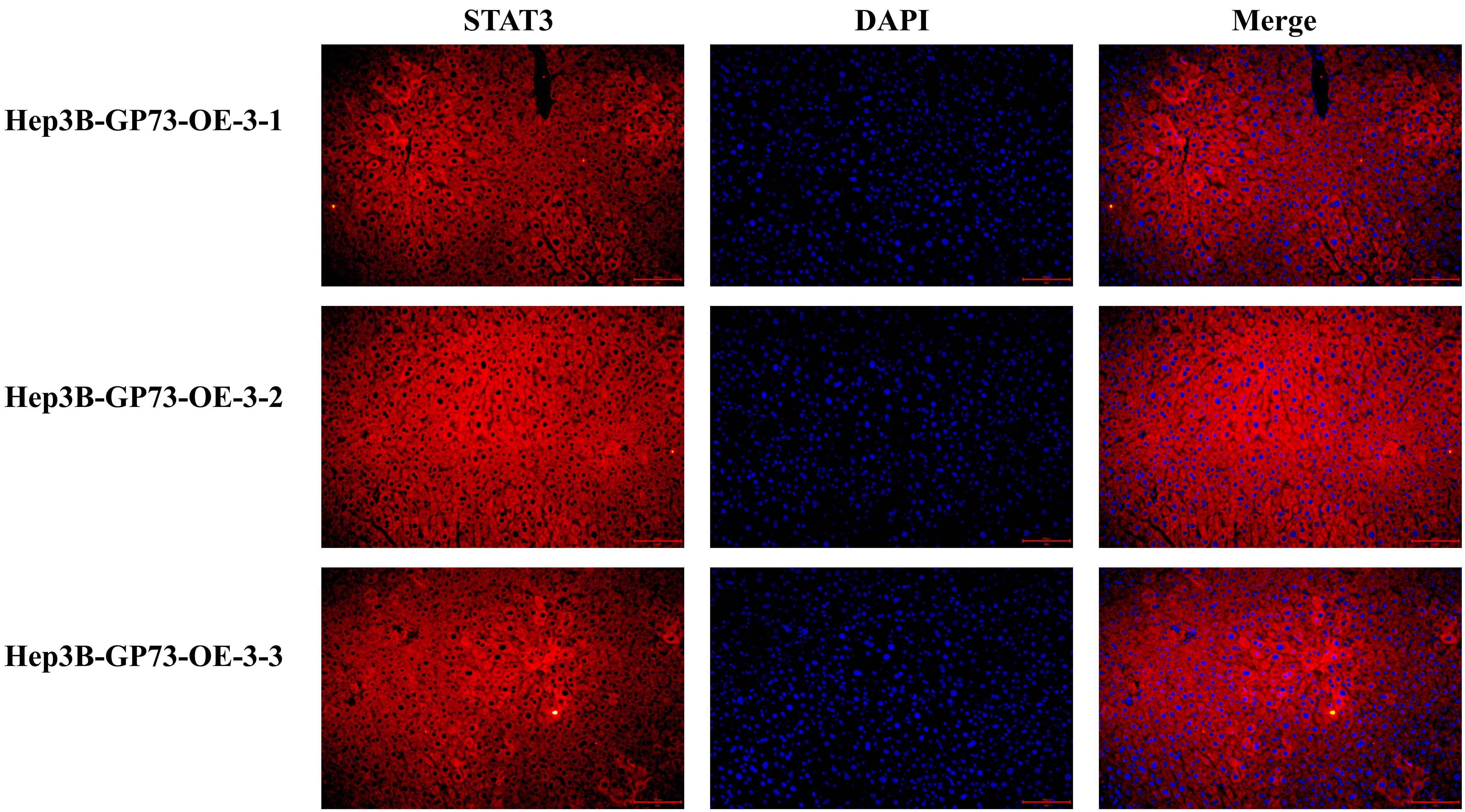

STAT3
DAPI
Merge
Hep3B-GP73-OE-3-1
Hep3B-GP73-OE-3-2
Hep3B-GP73-OE-3-3

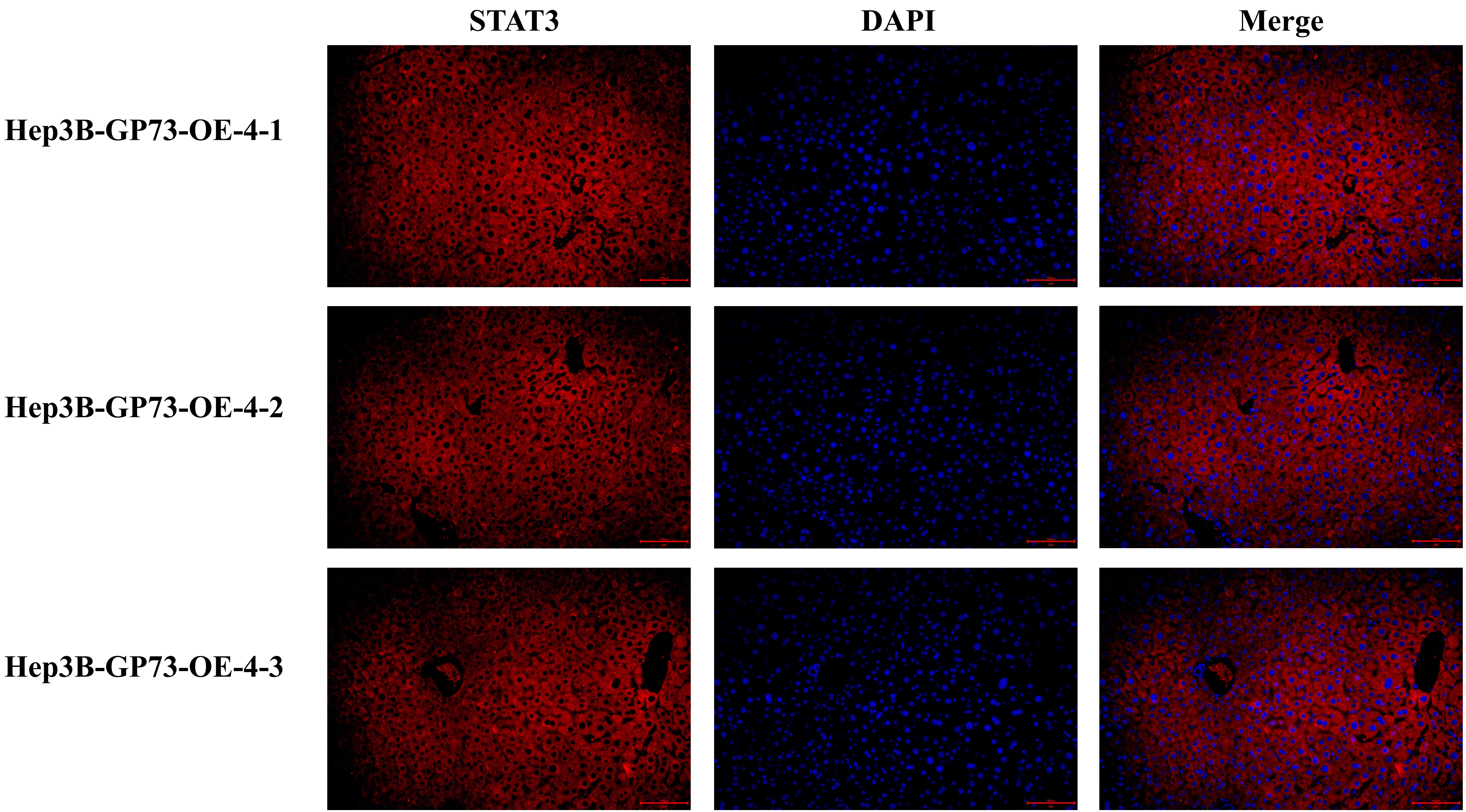
STAT3
DAPI
Merge
Hep3B-GP73-OE-4-1
Hep3B-GP73-OE-4-2
Hep3B-GP73-OE-4-3

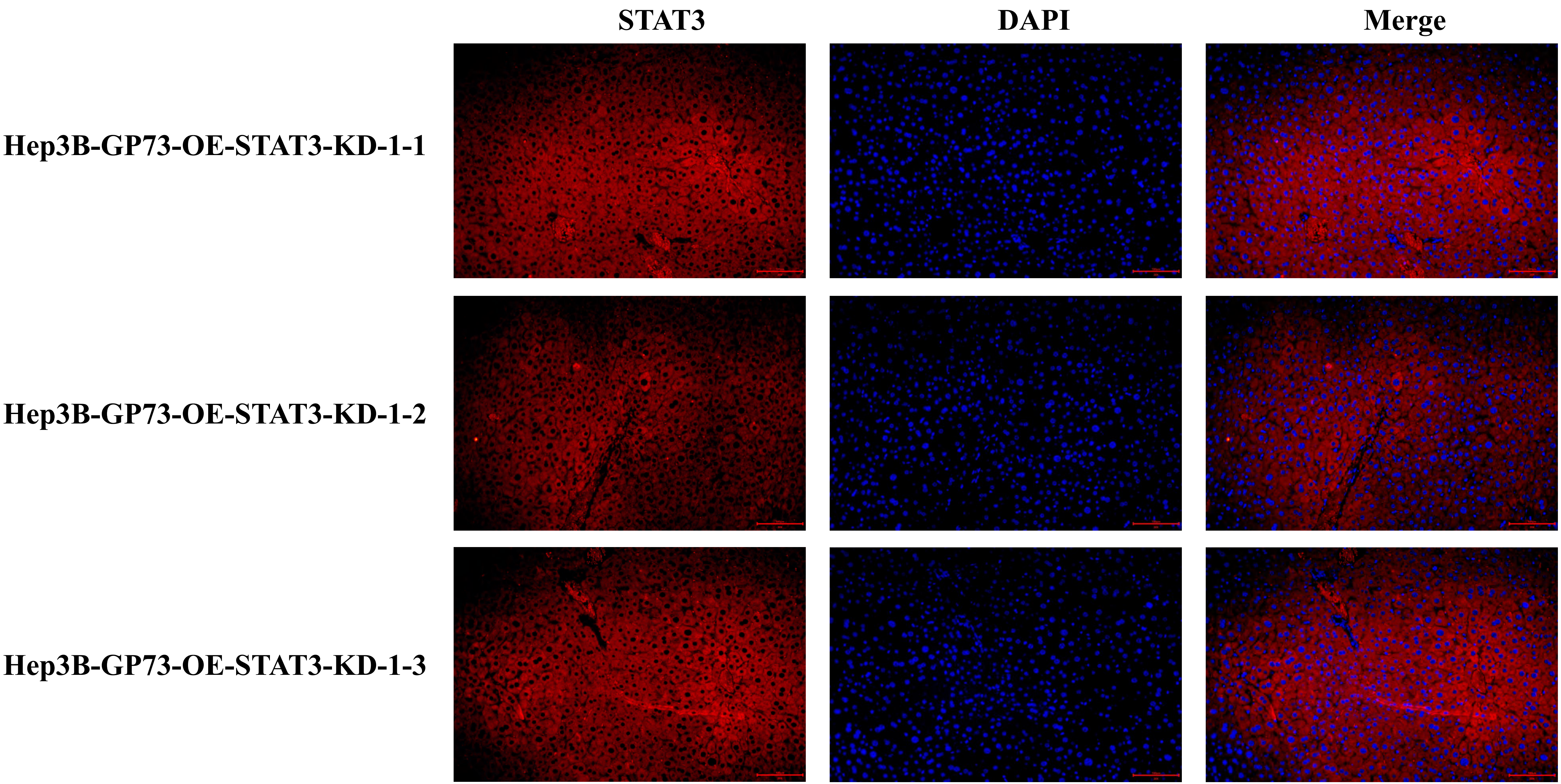
STAT3
DAPI
Merge
Hep3B-GP73-OE-STAT3-KD-1-1
Hep3B-GP73-OE-STAT3-KD-1-2
Hep3B-GP73-OE-STAT3-KD-1-3

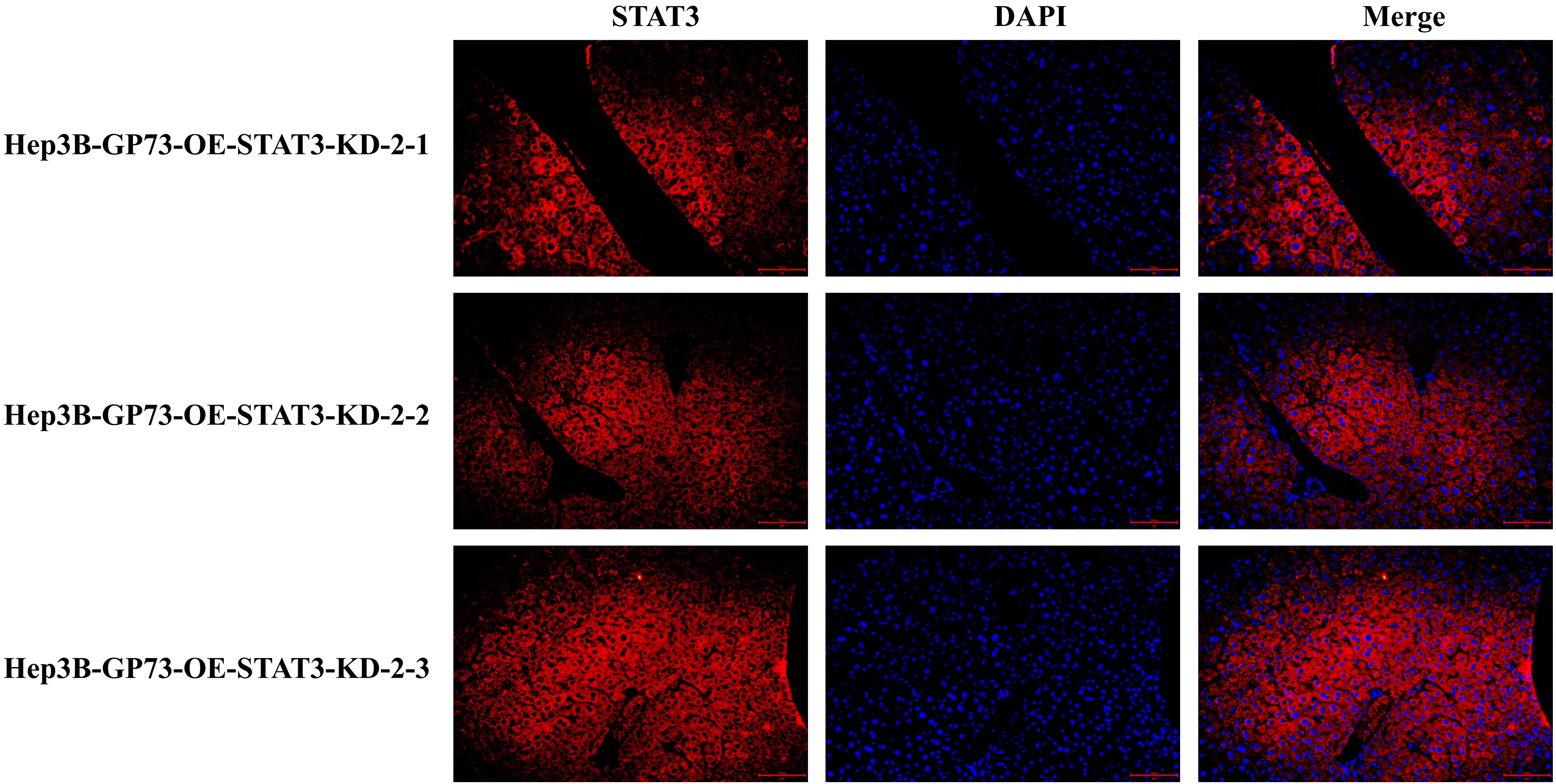
STAT3
DAPI
Merge
Hep3B-GP73-OE-STAT3-KD-2-1
Hep3B-GP73-OE-STAT3-KD-2-2
Hep3B-GP73-OE-STAT3-KD-2-3

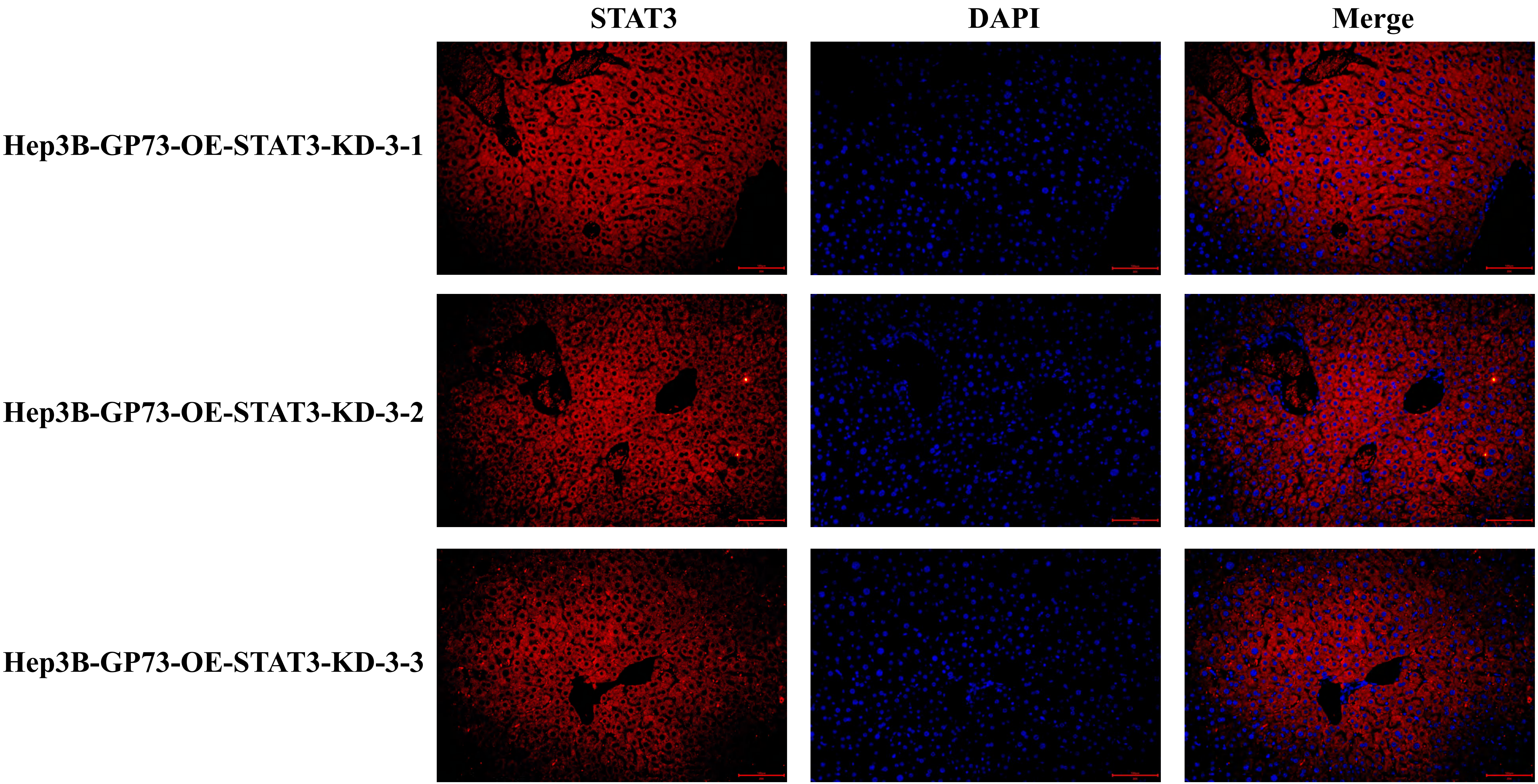

STAT3
DAPI
Merge
Hep3B-GP73-OE-STAT3-KD-3-1
Hep3B-GP73-OE-STAT3-KD-3-2
Hep3B-GP73-OE-STAT3-KD-3-3

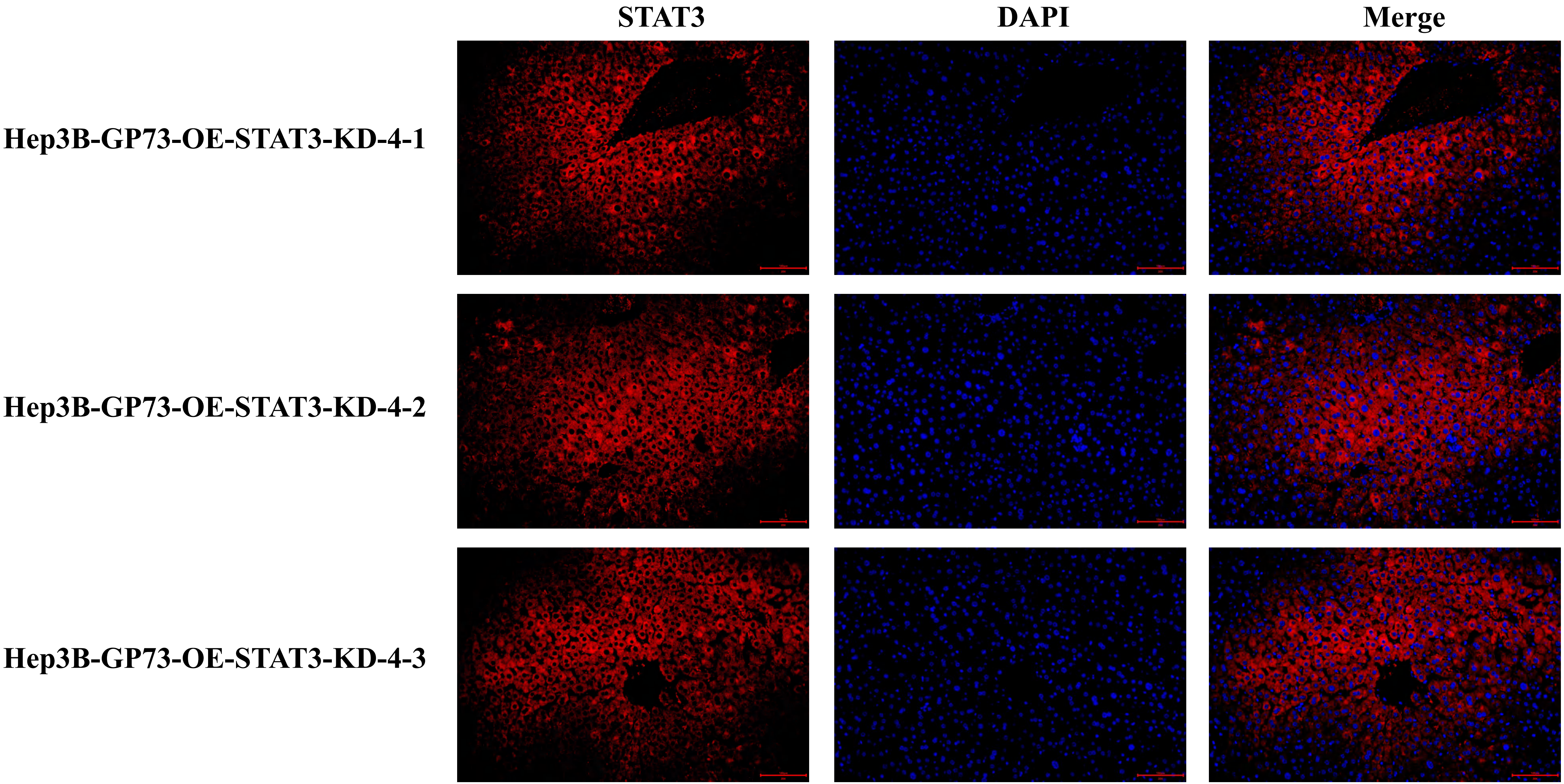
STAT3
DAPI
Merge
Hep3B-GP73-OE-STAT3-KD-4-1
Hep3B-GP73-OE-STAT3-KD-4-2
Hep3B-GP73-OE-STAT3-KD-4-3

Immunofluorescence staining showing decreased expressions of CD34 in resected tumors originating from the Hep3B-GP73-OE-STAT3-KD cells compared to the Hep3B-GP73-OE cells, original magnification, ×20.

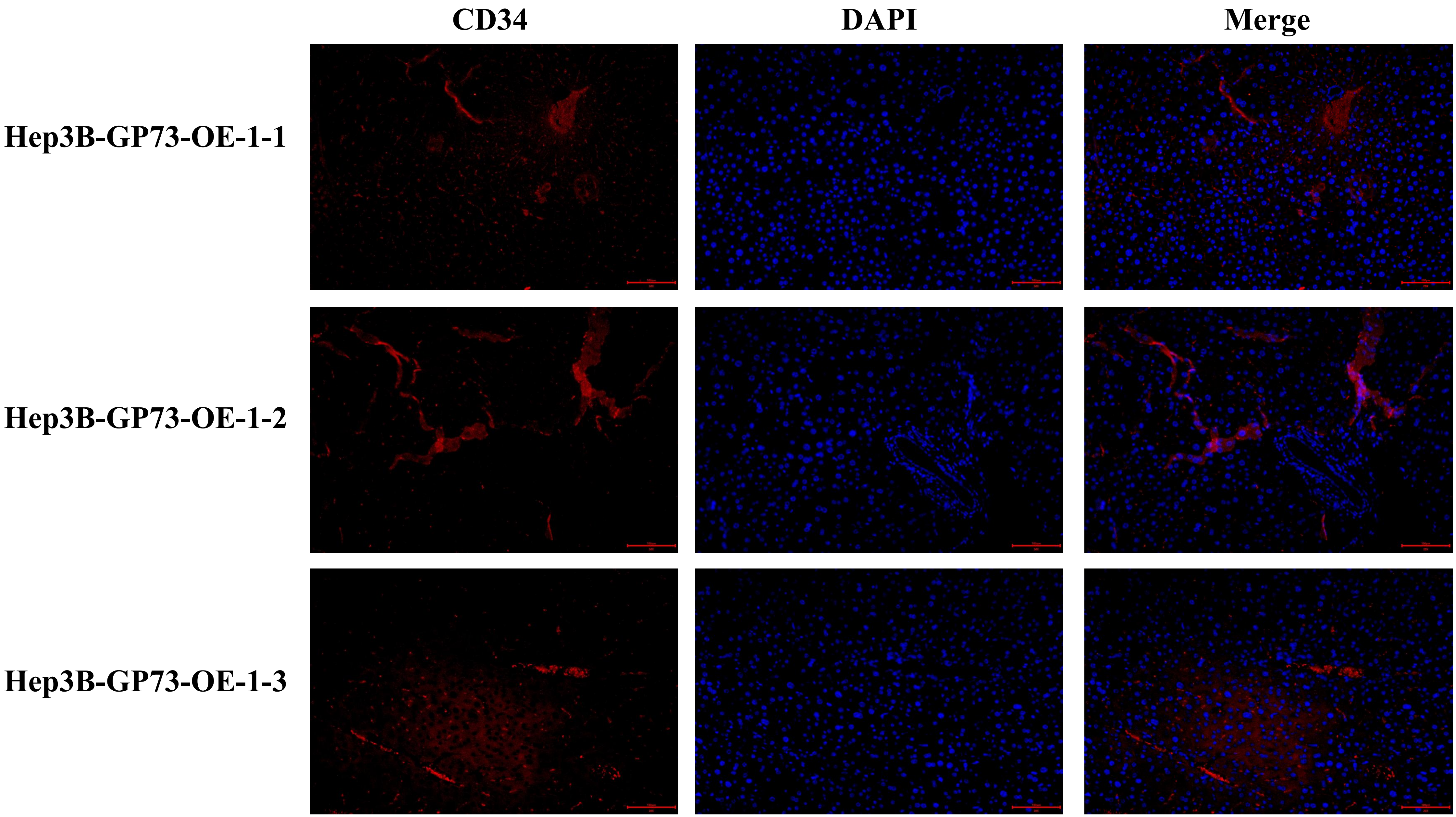
CD34
DAPI
Merge
Hep3B-GP73-OE-1-1
Hep3B-GP73-OE-1-2
Hep3B-GP73-OE-1-3

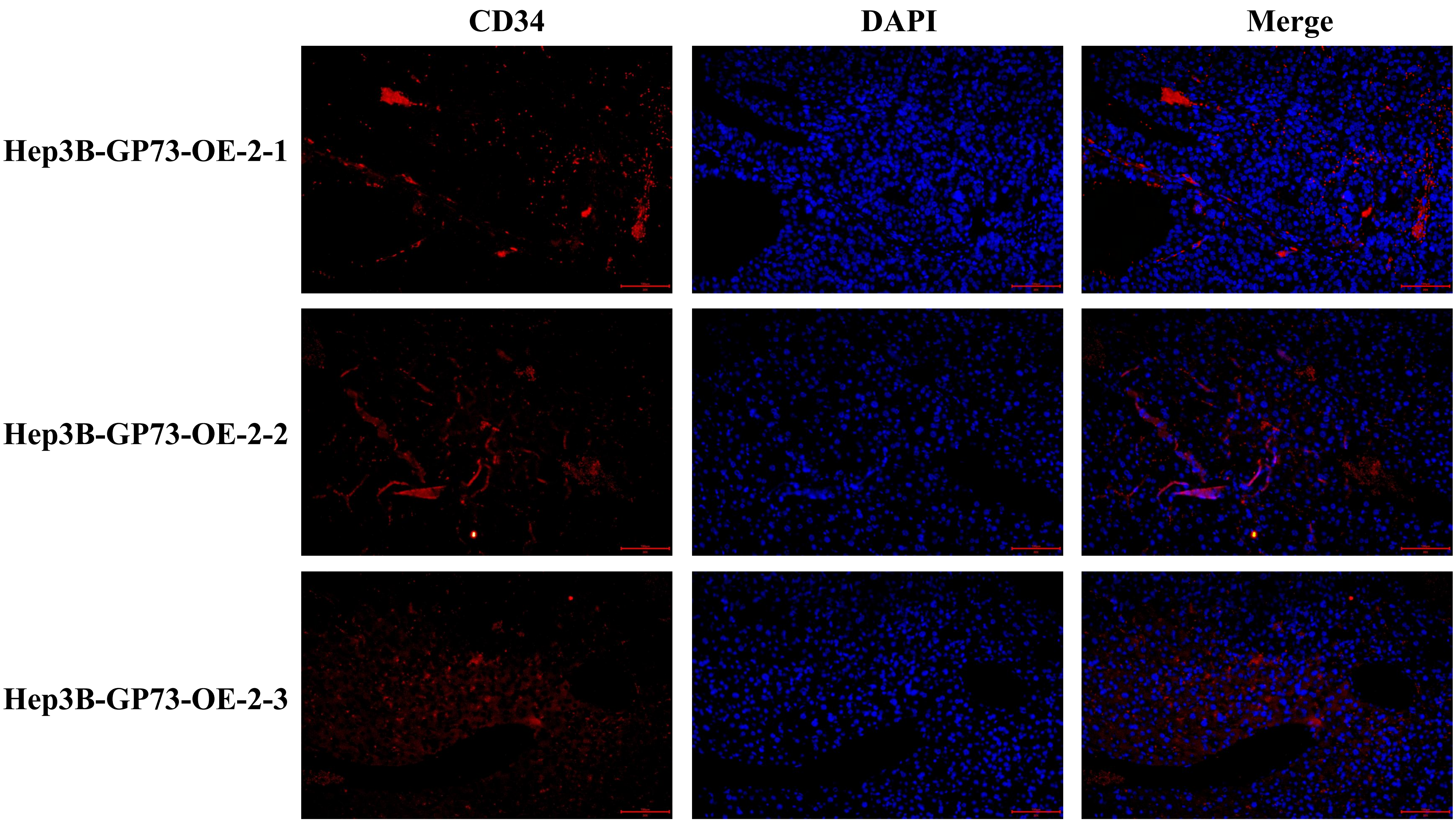

CD34
DAPI
Merge
Hep3B-GP73-OE-2-1
Hep3B-GP73-OE-2-2
Hep3B-GP73-OE-2-3

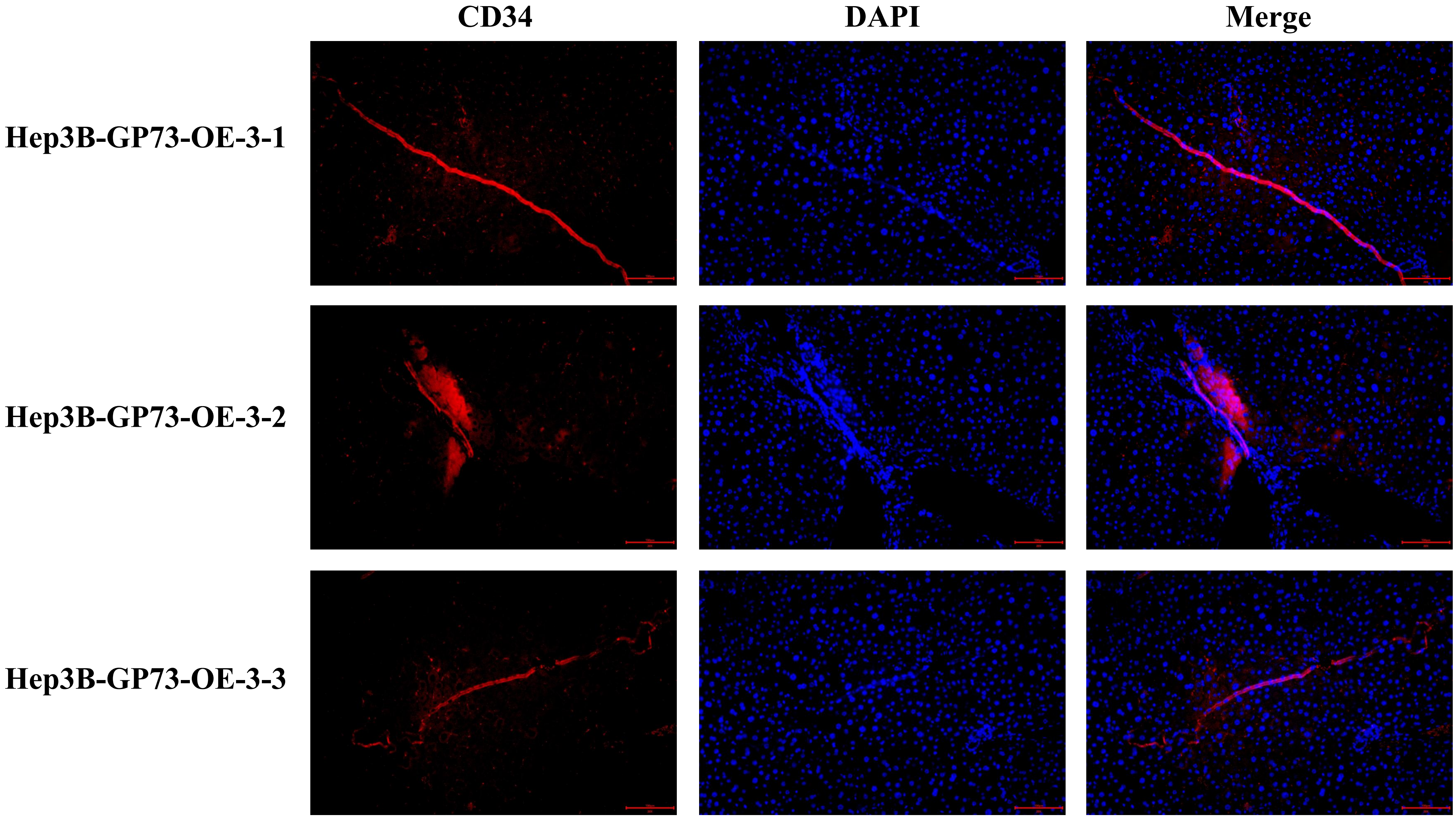

CD34
DAPI
Merge
Hep3B-GP73-OE-3-1
Hep3B-GP73-OE-3-2
Hep3B-GP73-OE-3-3

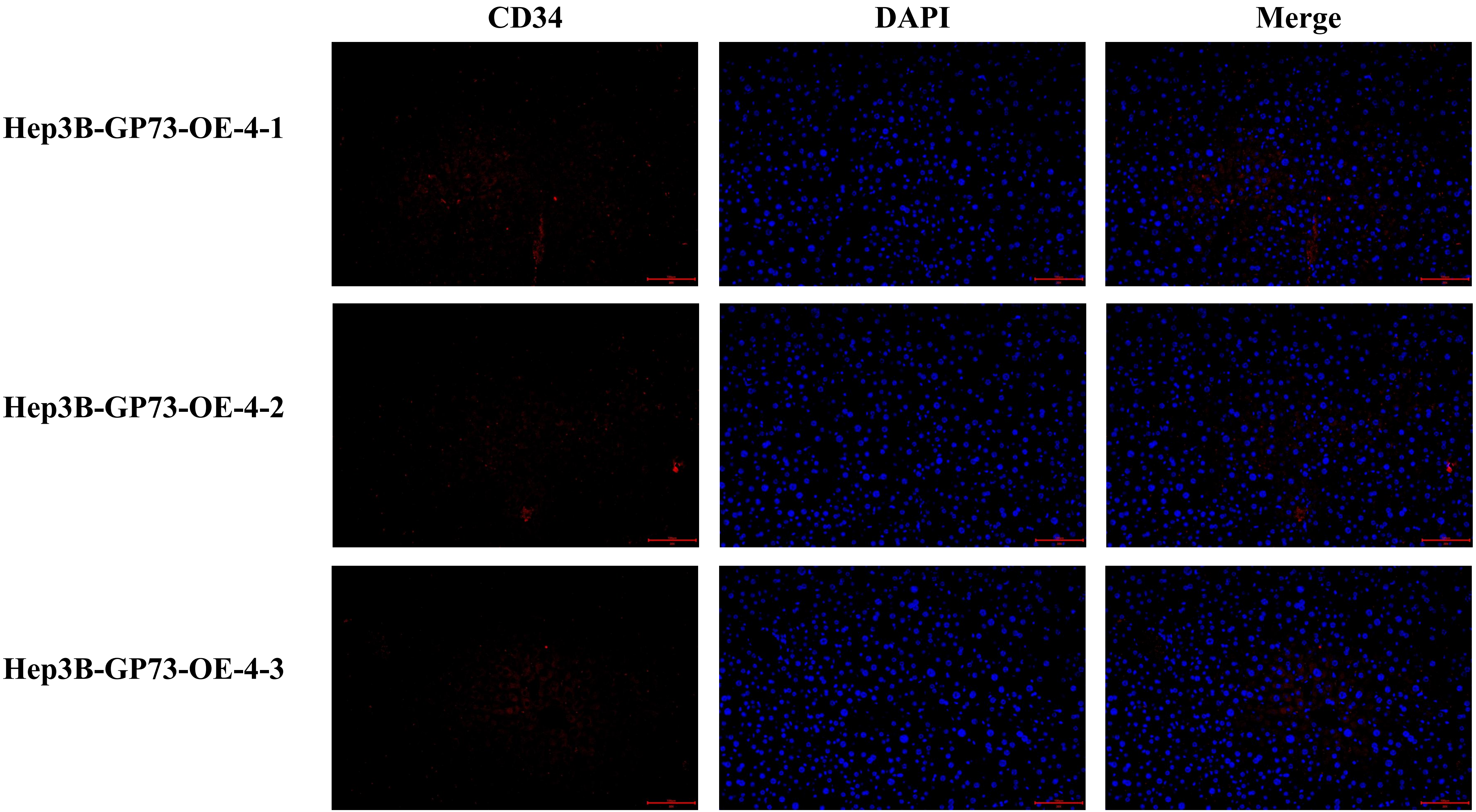
CD34
DAPI
Merge
Hep3B-GP73-OE-4-1
Hep3B-GP73-OE-4-2
Hep3B-GP73-OE-4-3

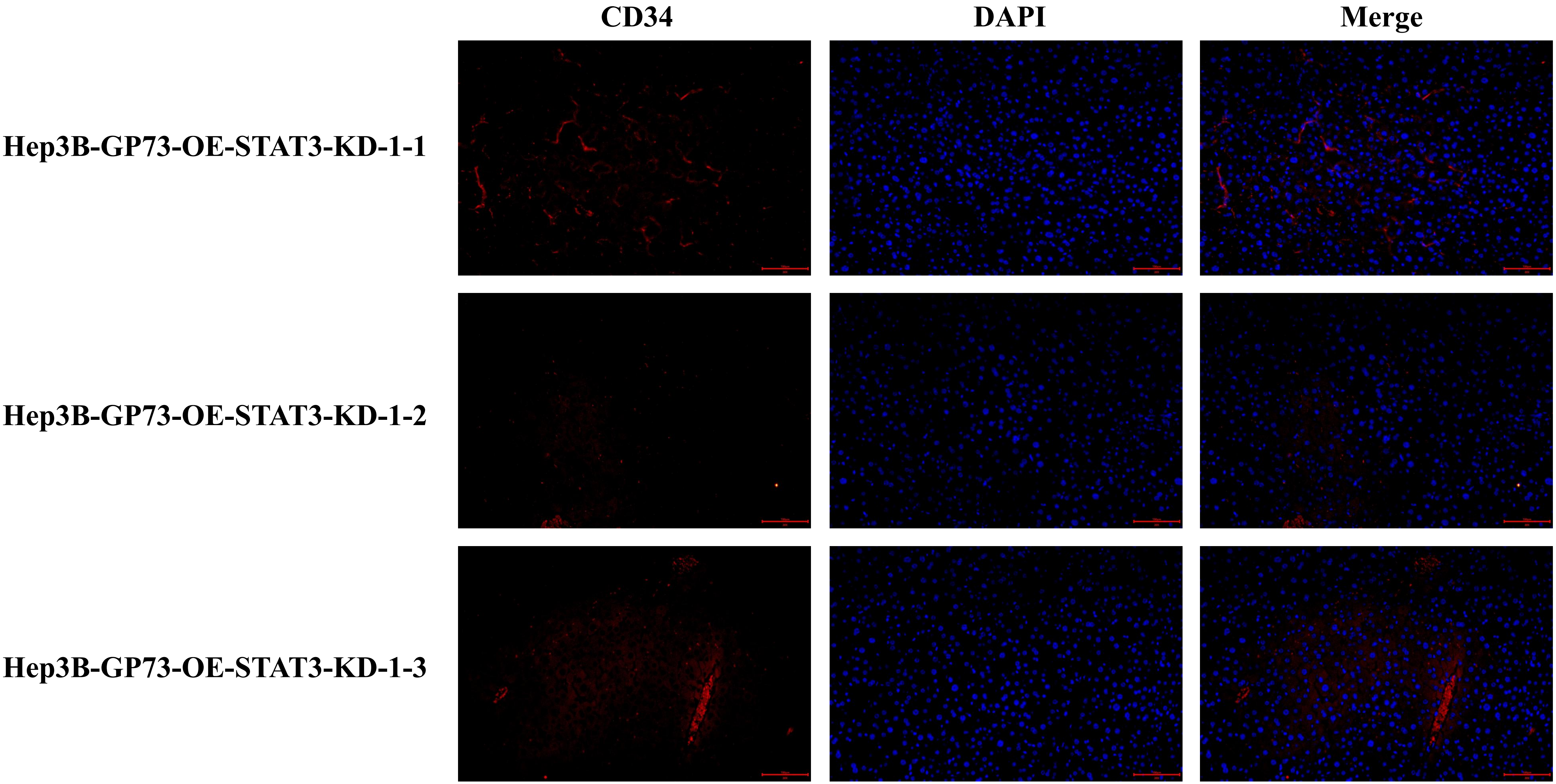
CD34
DAPI
Merge
Hep3B-GP73-OE-STAT3-KD-1-1
Hep3B-GP73-OE-STAT3-KD-1-2
Hep3B-GP73-OE-STAT3-KD-1-3

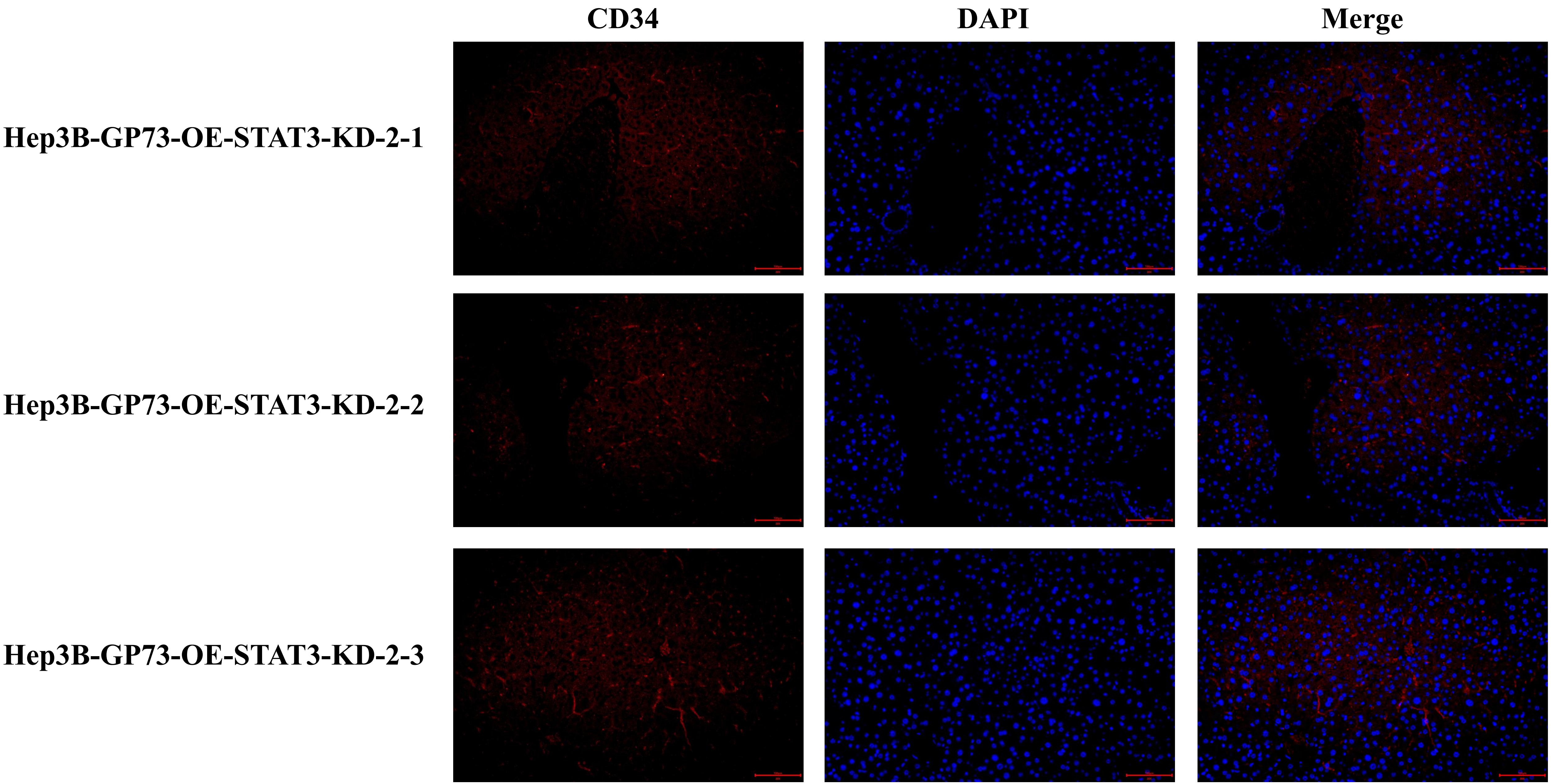
CD34
DAPI
Merge
Hep3B-GP73-OE-STAT3-KD-2-1
Hep3B-GP73-OE-STAT3-KD-2-2
Hep3B-GP73-OE-STAT3-KD-2-3

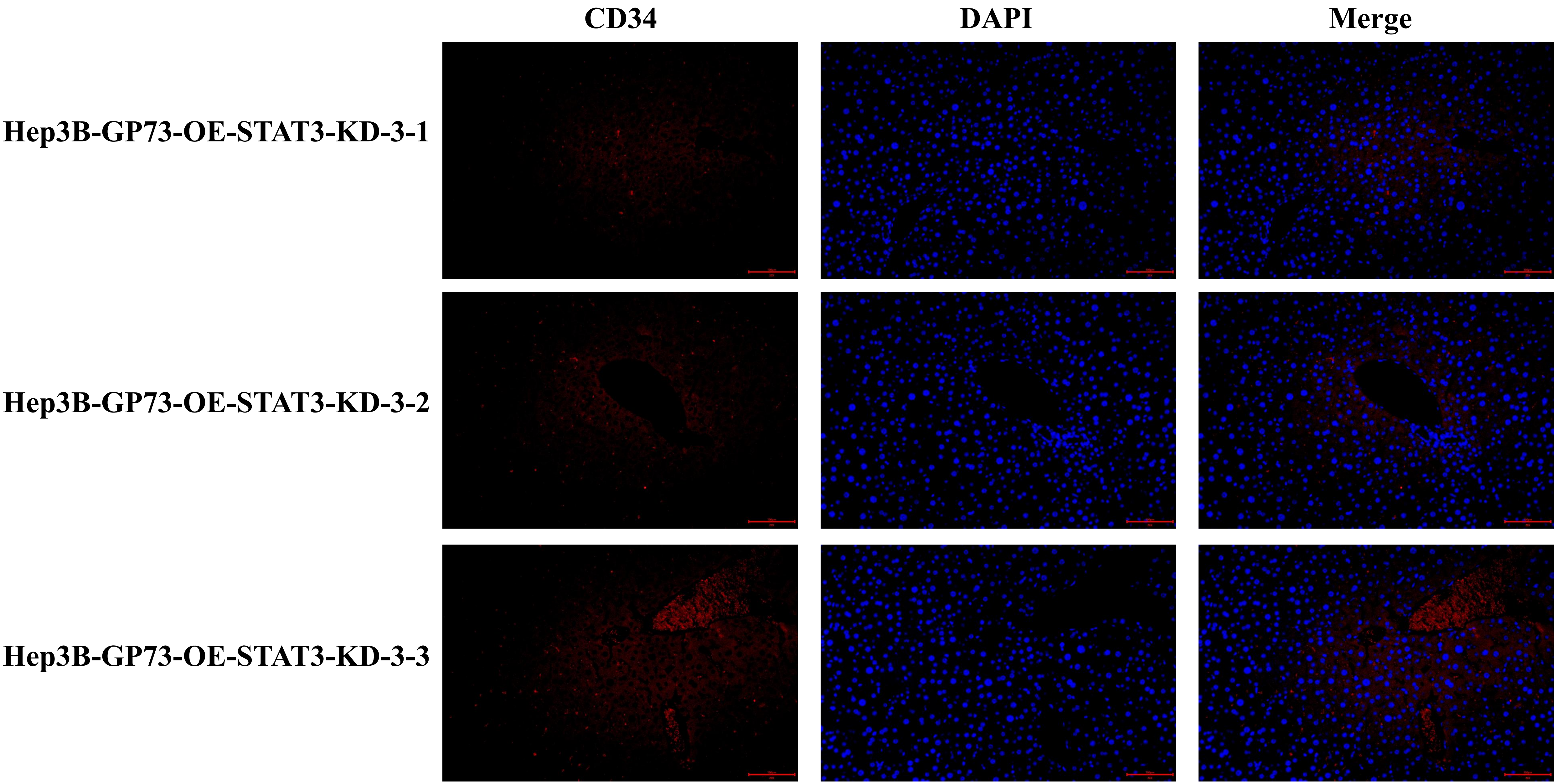
CD34
DAPI
Merge
Hep3B-GP73-OE-STAT3-KD-3-1
Hep3B-GP73-OE-STAT3-KD-3-2
Hep3B-GP73-OE-STAT3-KD-3-3

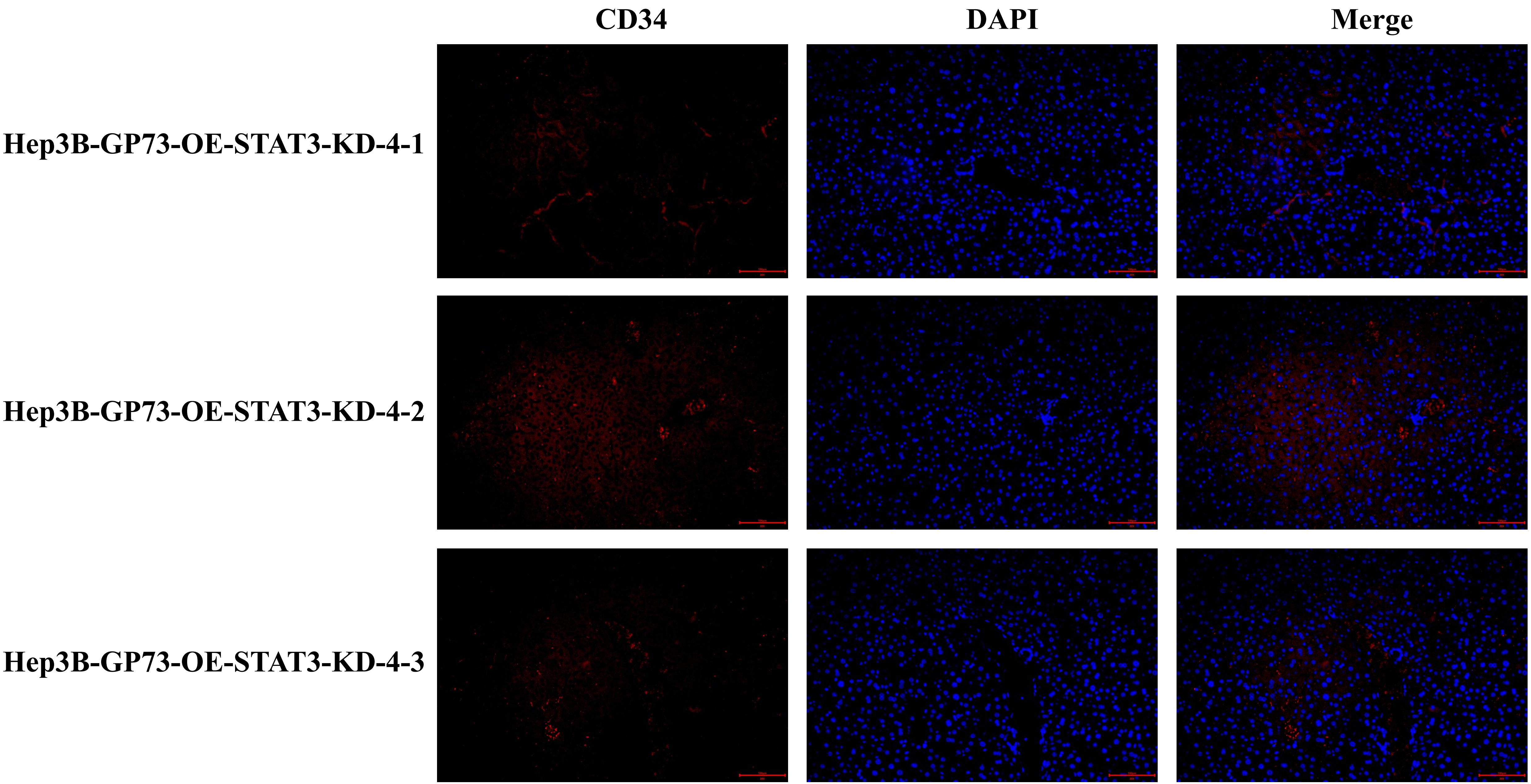
CD34
DAPI
Merge
Hep3B-GP73-OE-STAT3-KD-4-1
Hep3B-GP73-OE-STAT3-KD-4-2
Hep3B-GP73-OE-STAT3-KD-4-3

Immunofluorescence staining showing decreased expressions of CD31 in resected tumors originating from the Hep3B-GP73-OE-STAT3-KD cells compared to the Hep3B-GP73-OE cells, original magnification, ×20.

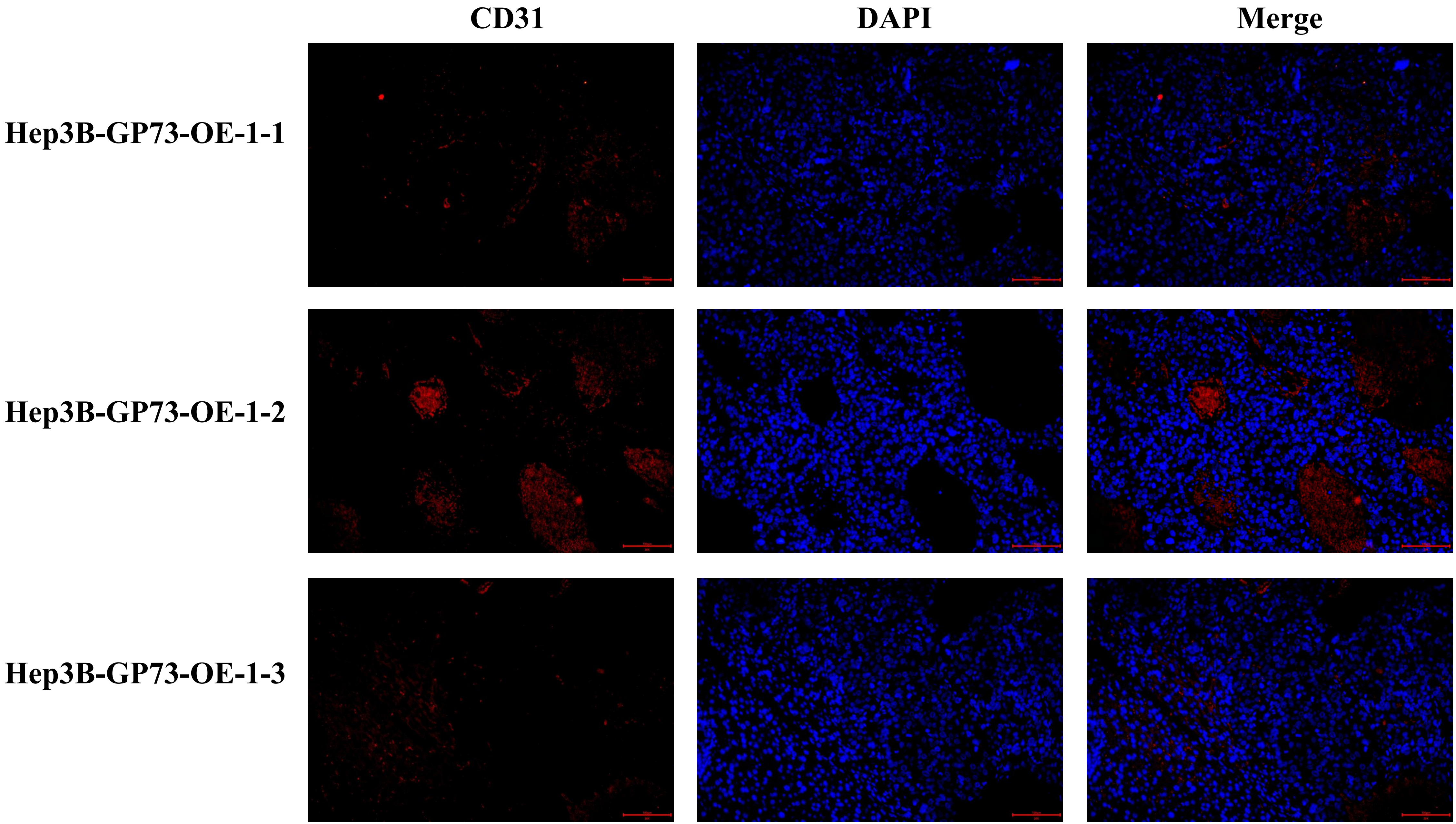
CD31
DAPI
Merge
Hep3B-GP73-OE-1-1
Hep3B-GP73-OE-1-2
Hep3B-GP73-OE-1-3

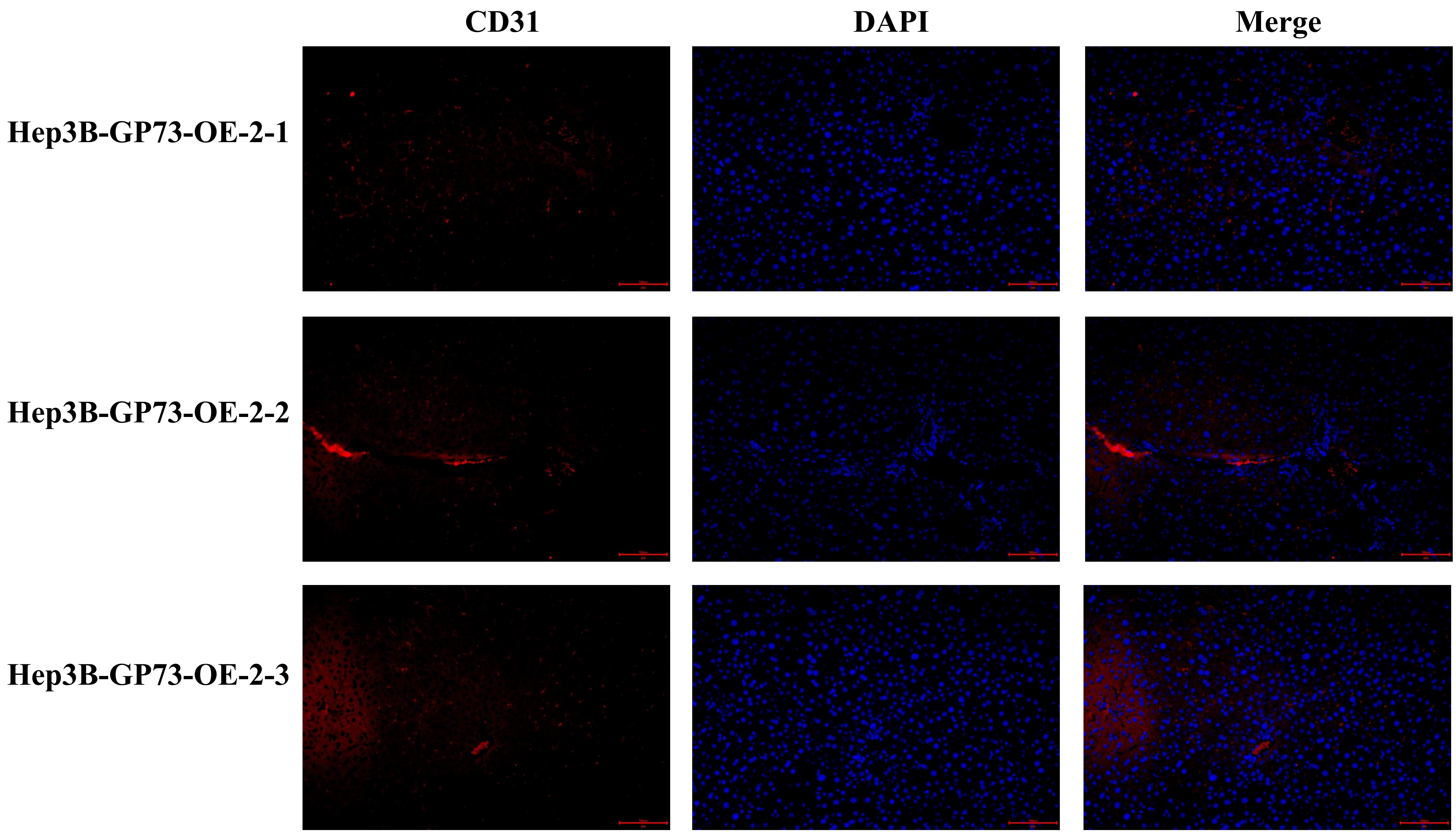
CD31
DAPI
Merge
Hep3B-GP73-OE-2-1
Hep3B-GP73-OE-2-2
Hep3B-GP73-OE-2-3

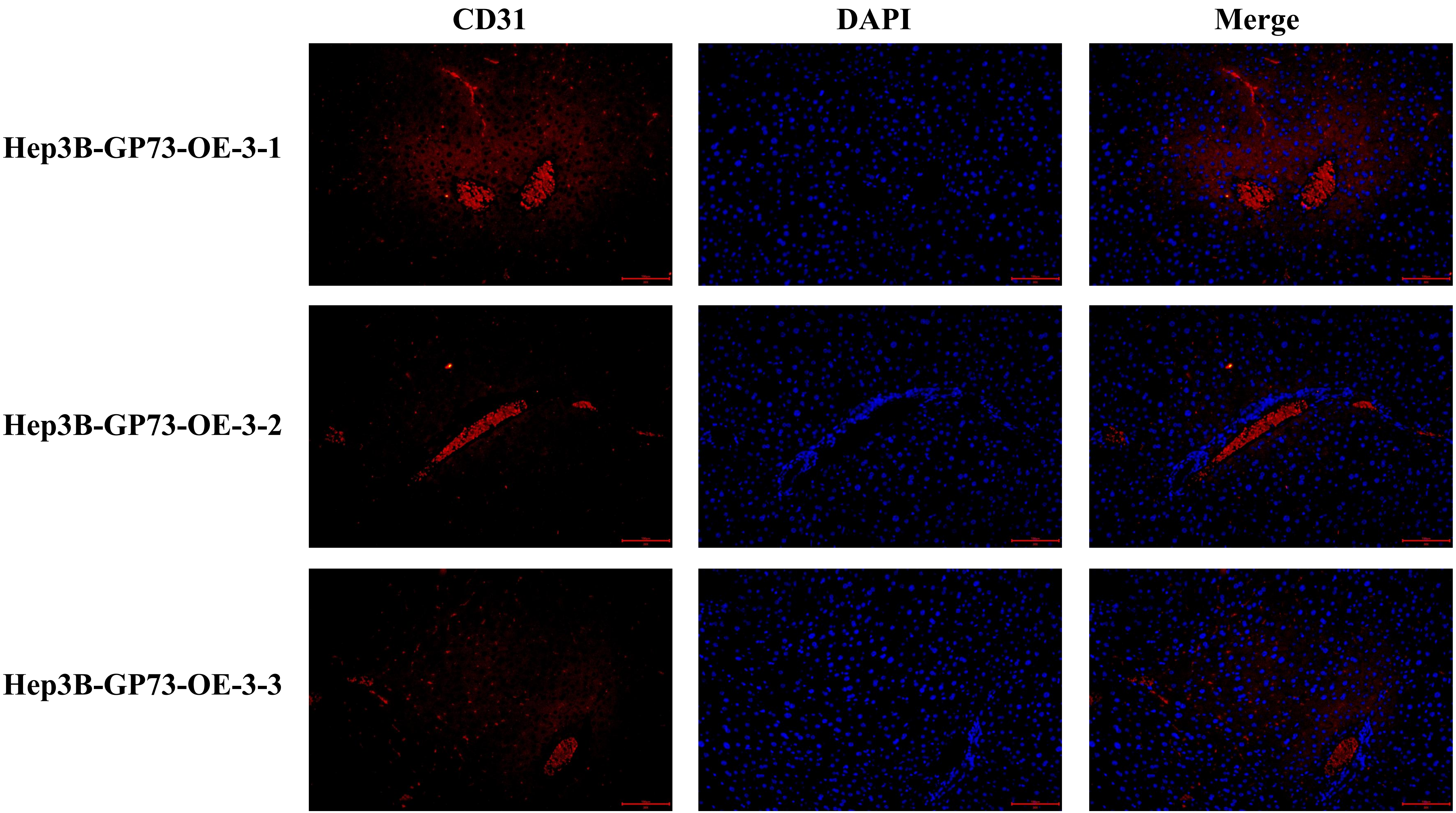
CD31
DAPI
Merge
Hep3B-GP73-OE-3-1
Hep3B-GP73-OE-3-2
Hep3B-GP73-OE-3-3

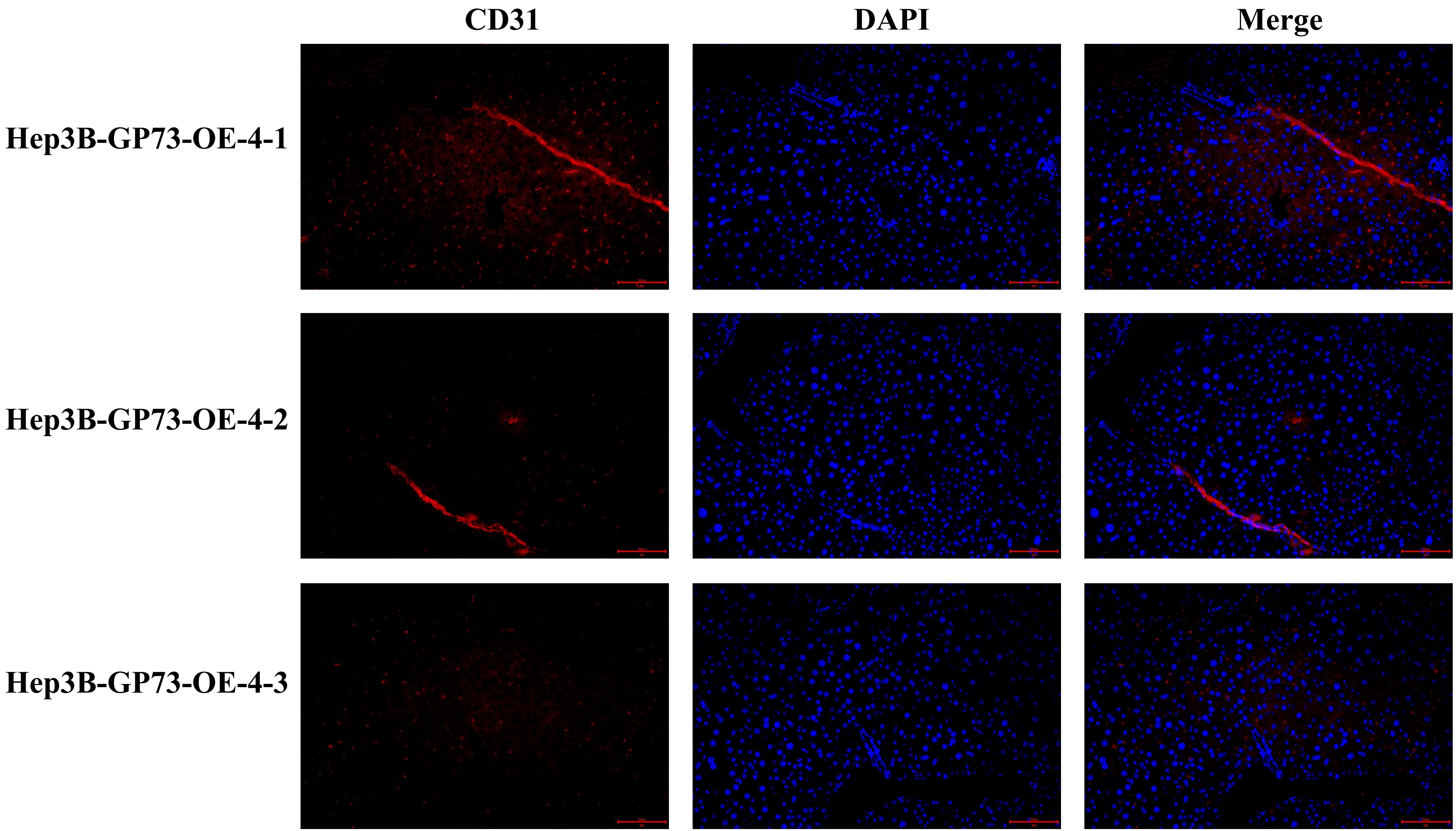
CD31
DAPI
Merge
Hep3B-GP73-OE-4-1
Hep3B-GP73-OE-4-2
Hep3B-GP73-OE-4-3

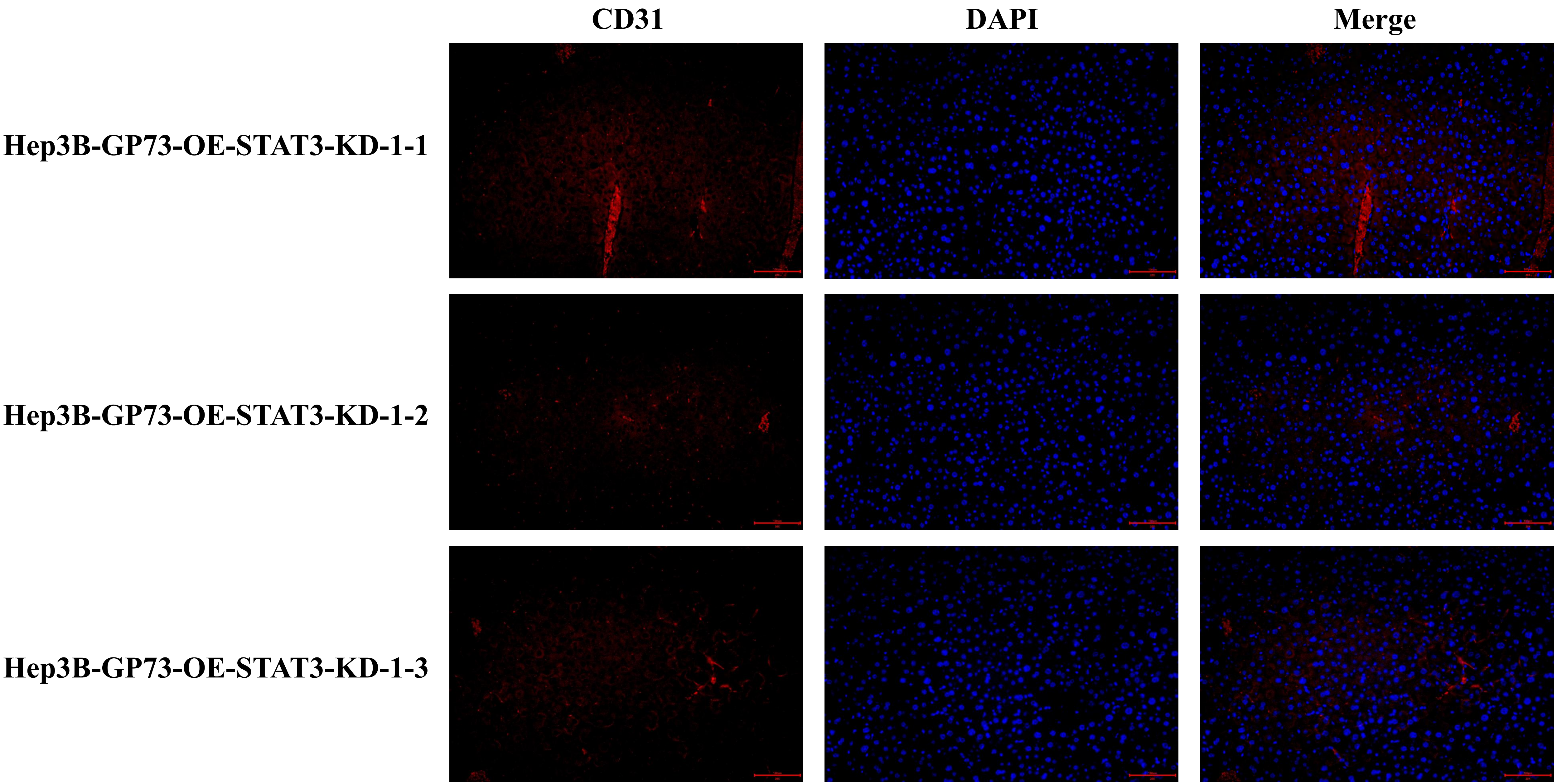

CD31
DAPI
Merge
Hep3B-GP73-OE-STAT3-KD-1-1
Hep3B-GP73-OE-STAT3-KD-1-2
Hep3B-GP73-OE-STAT3-KD-1-3

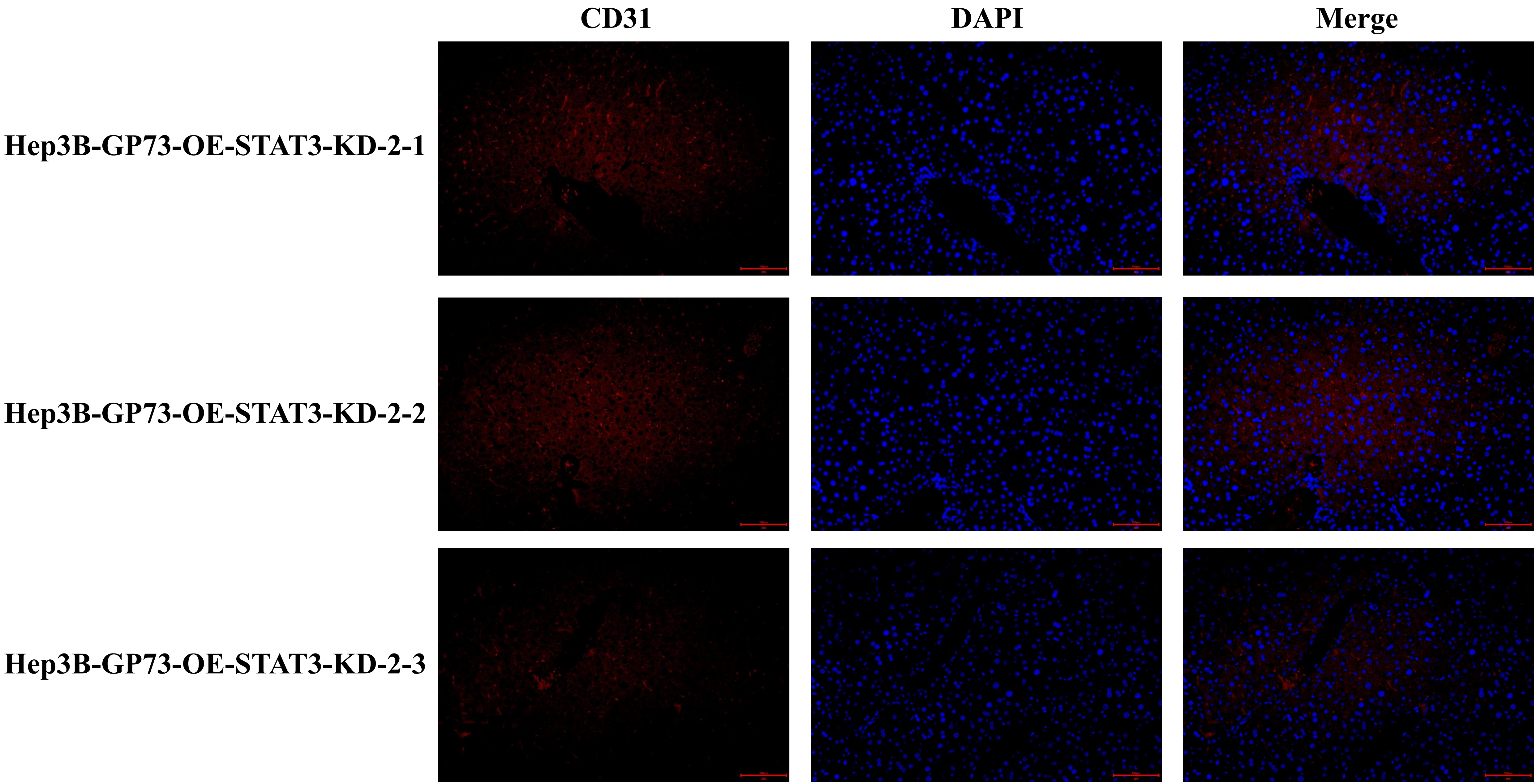
CD31
DAPI
Merge
Hep3B-GP73-OE-STAT3-KD-2-1
Hep3B-GP73-OE-STAT3-KD-2-2
Hep3B-GP73-OE-STAT3-KD-2-3

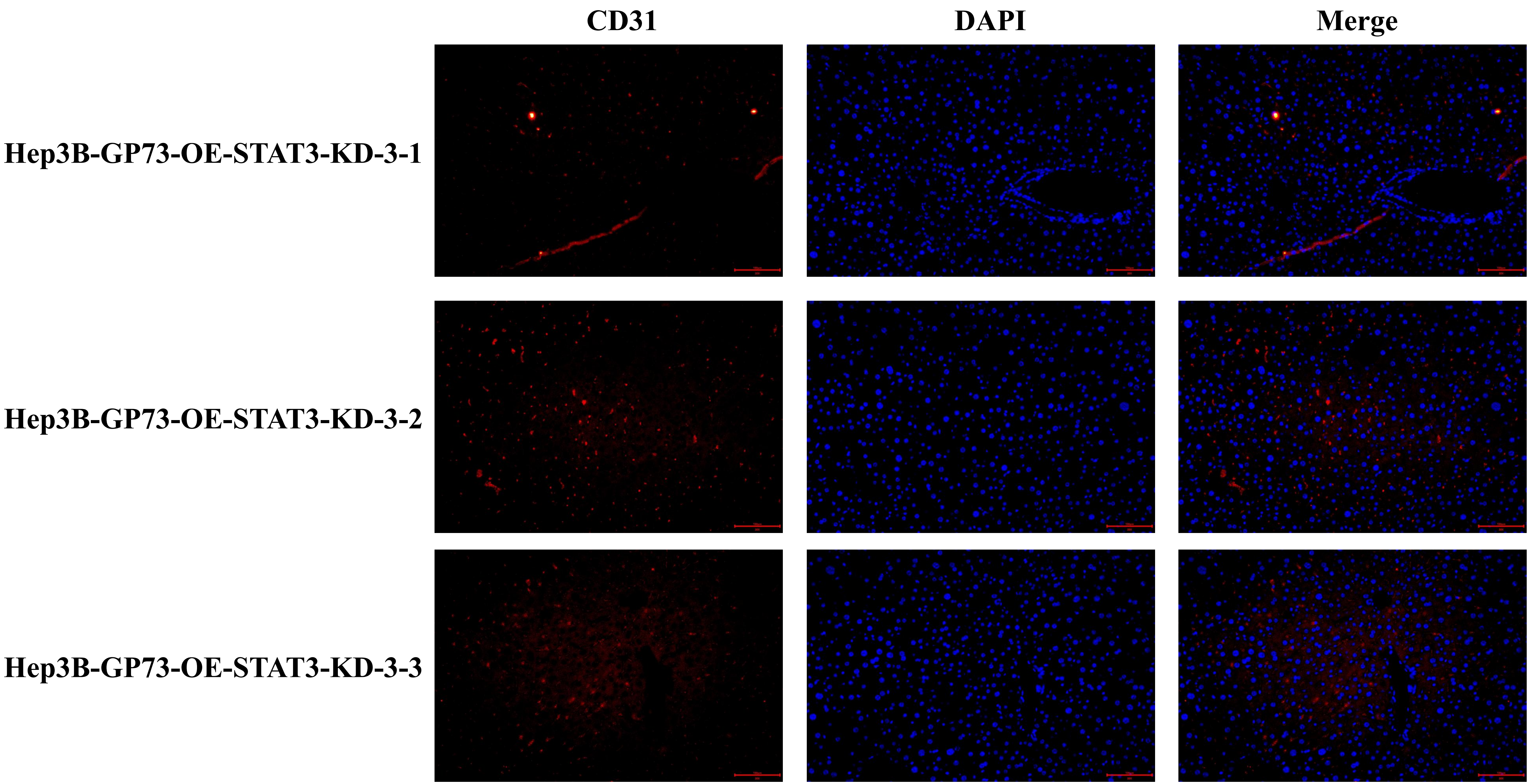
CD31
DAPI
Merge
Hep3B-GP73-OE-STAT3-KD-3-1
Hep3B-GP73-OE-STAT3-KD-3-2
Hep3B-GP73-OE-STAT3-KD-3-3

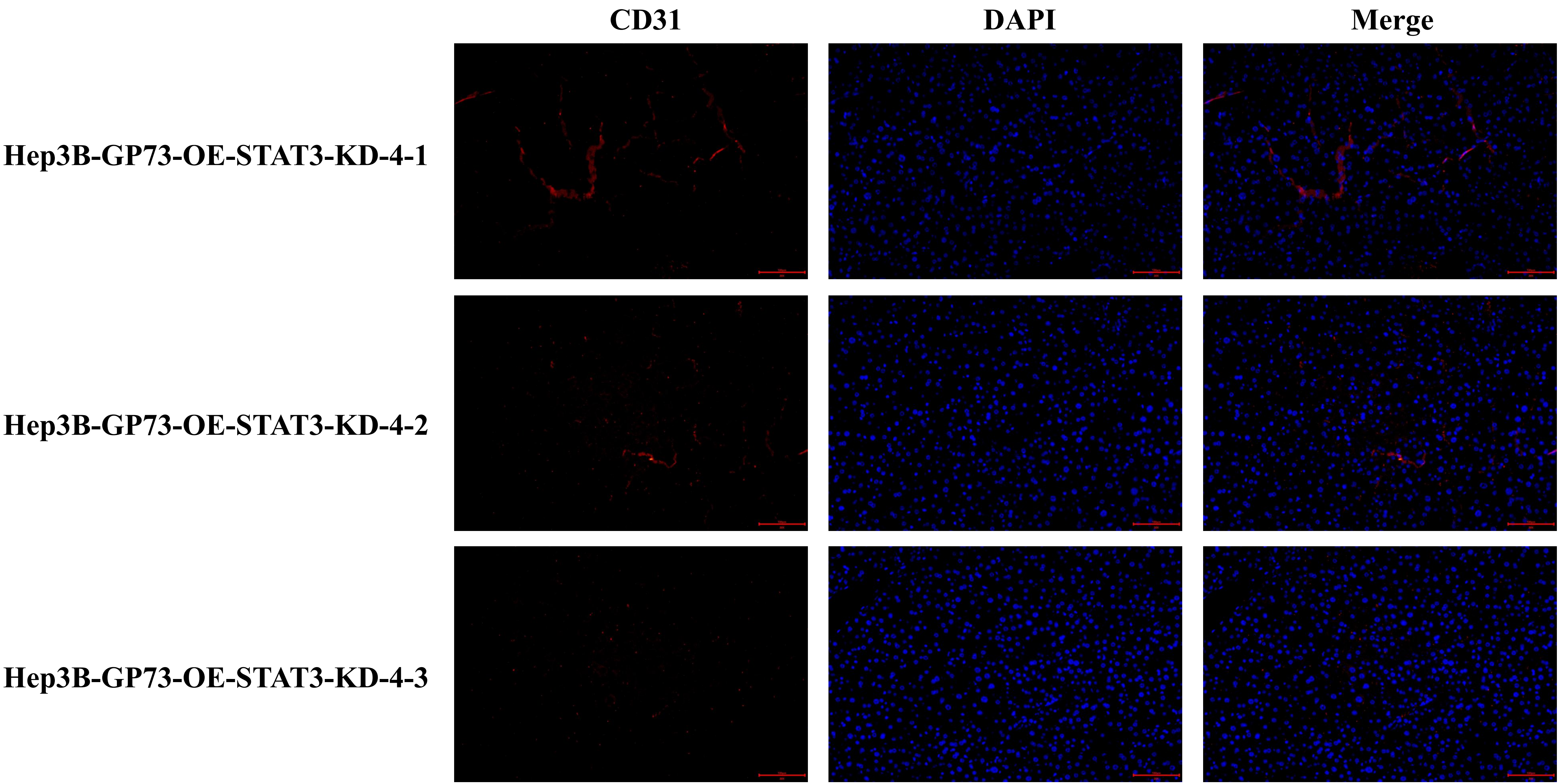
CD31
DAPI
Merge
Hep3B-GP73-OE-STAT3-KD-4-1
Hep3B-GP73-OE-STAT3-KD-4-2
Hep3B-GP73-OE-STAT3-KD-4-3

Figure 7K

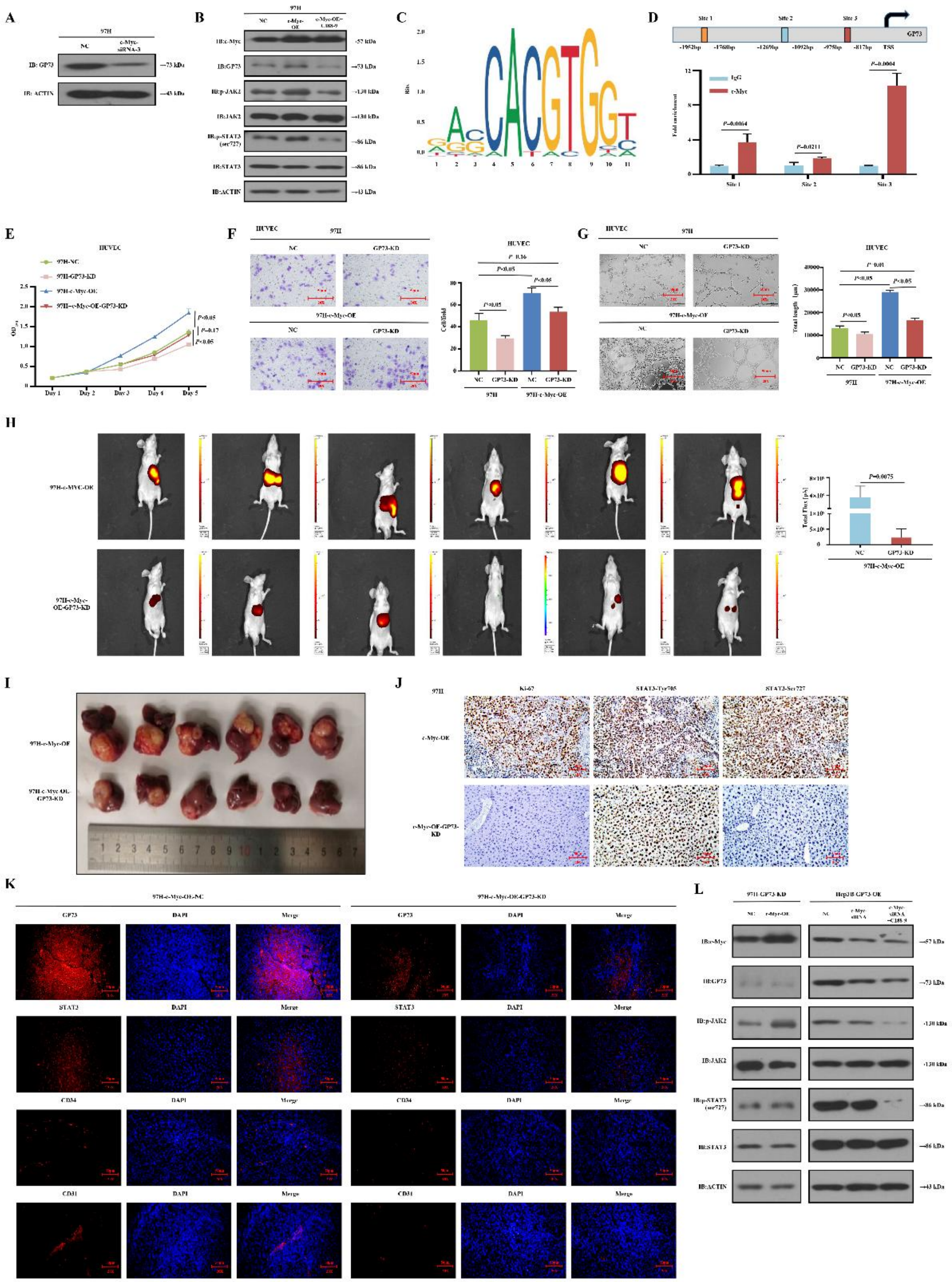

Immunofluorescence staining showing decreased expressions of GP73 in the resected tumors originated from the MHCC97H-c-Myc-OE-GP73-KD cells compared to the MHCC97H-c-Myc-OE cells, original magnification, ×20. Data were representative of three similar observations or were shown as the mean±SD of three experiments.

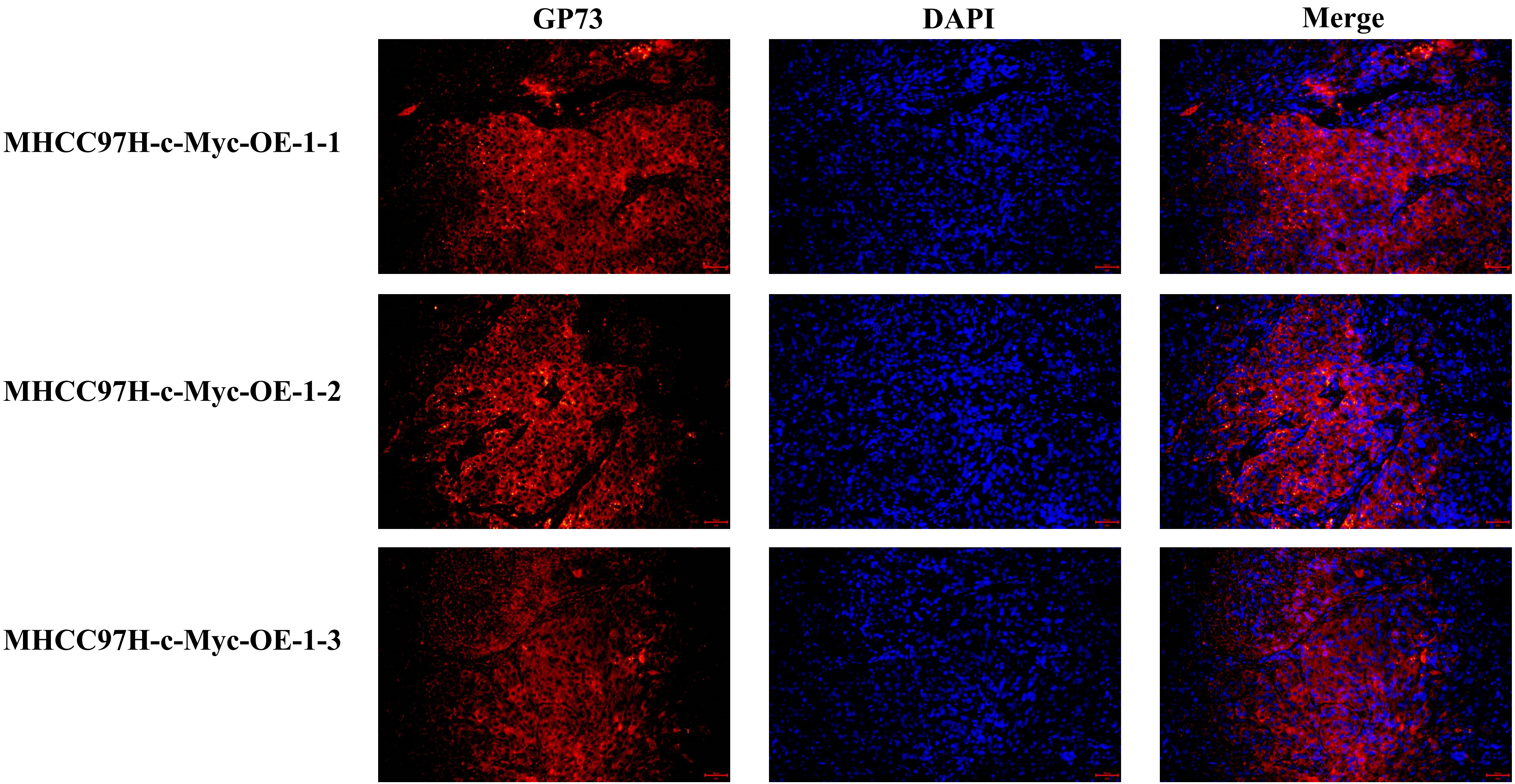
GP73
DAPI
Merge
MHCC97H-c-Myc-OE-1-1
MHCC97H-c-Myc-OE-1-2
MHCC97H-c-Myc-OE-1-3

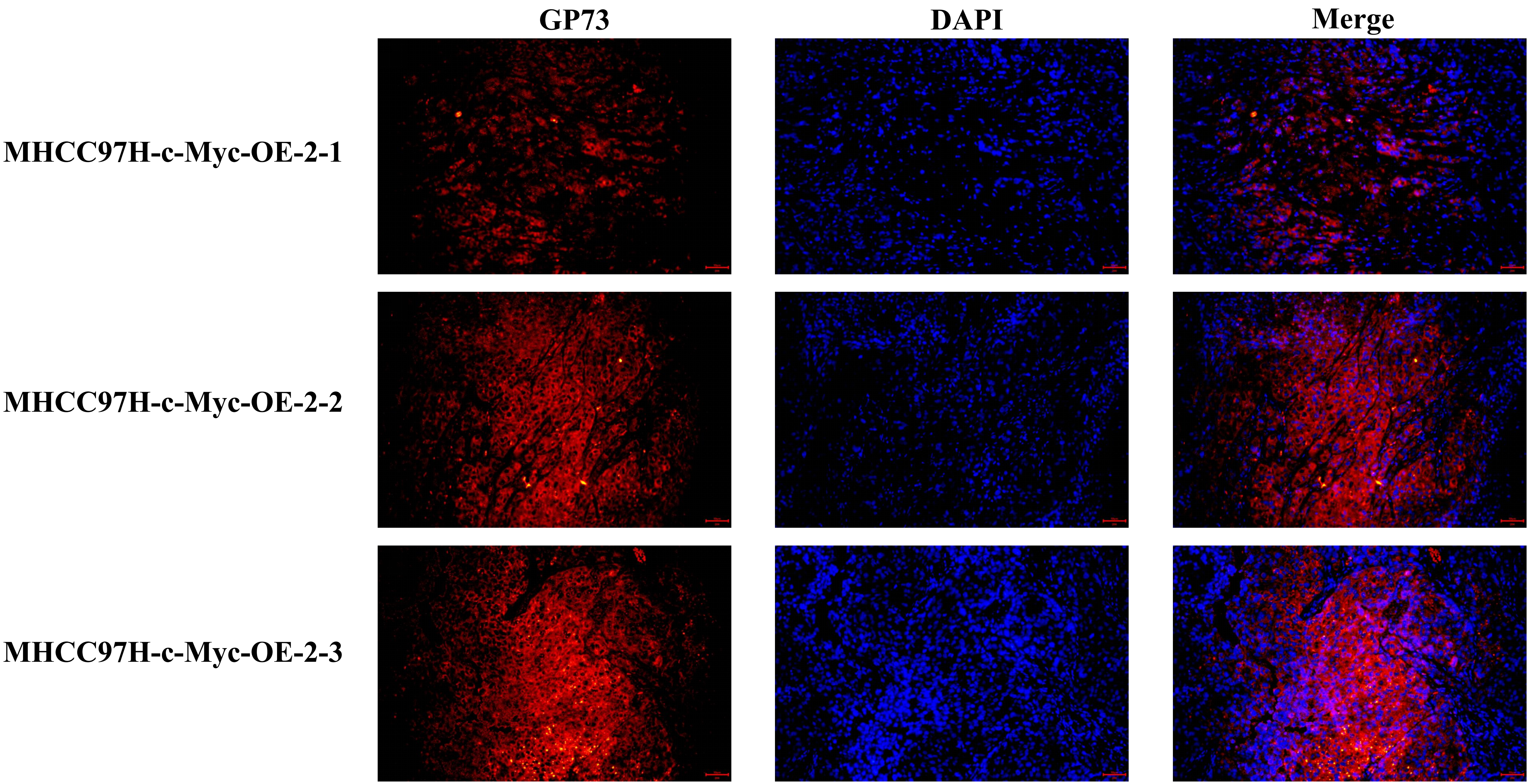
GP73
DAPI
Merge
MHCC97H-c-Myc-OE-2-1
MHCC97H-c-Myc-OE-2-2
MHCC97H-c-Myc-OE-2-3

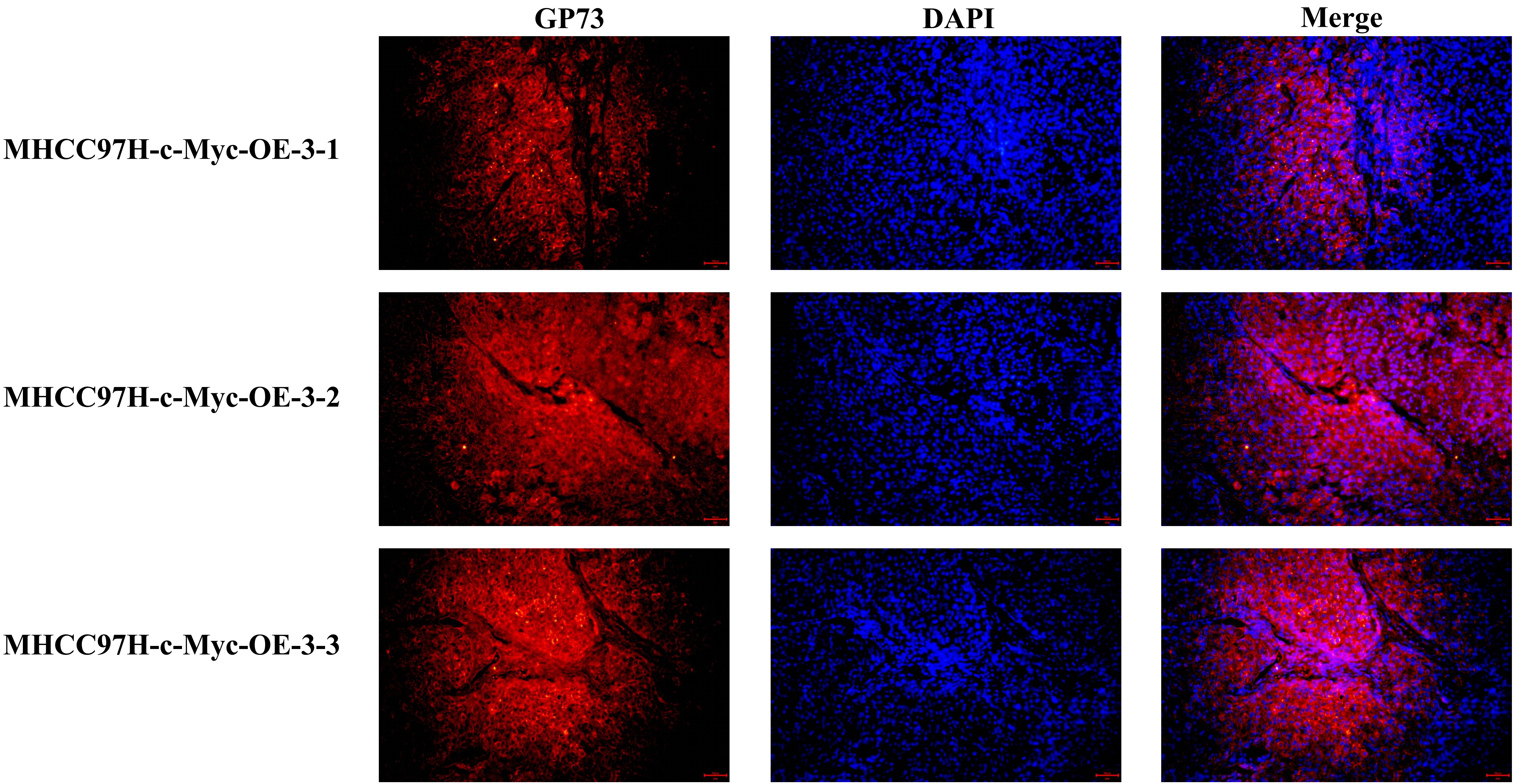
GP73
DAPI
Merge
MHCC97H-c-Myc-OE-3-1
MHCC97H-c-Myc-OE-3-2
MHCC97H-c-Myc-OE-3-3

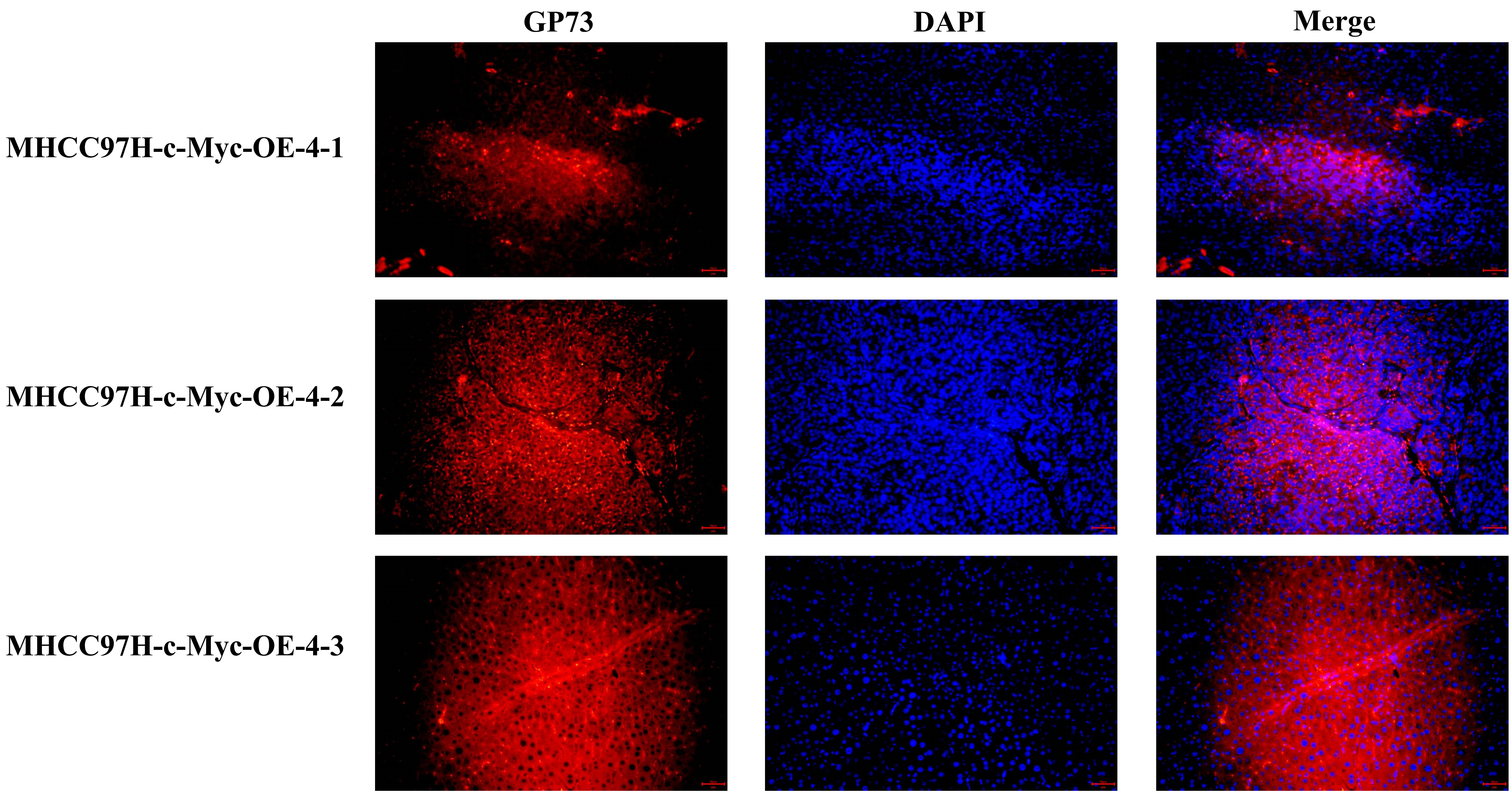
GP73
DAPI
Merge
MHCC97H-c-Myc-OE-4-1
MHCC97H-c-Myc-OE-4-2
MHCC97H-c-Myc-OE-4-3

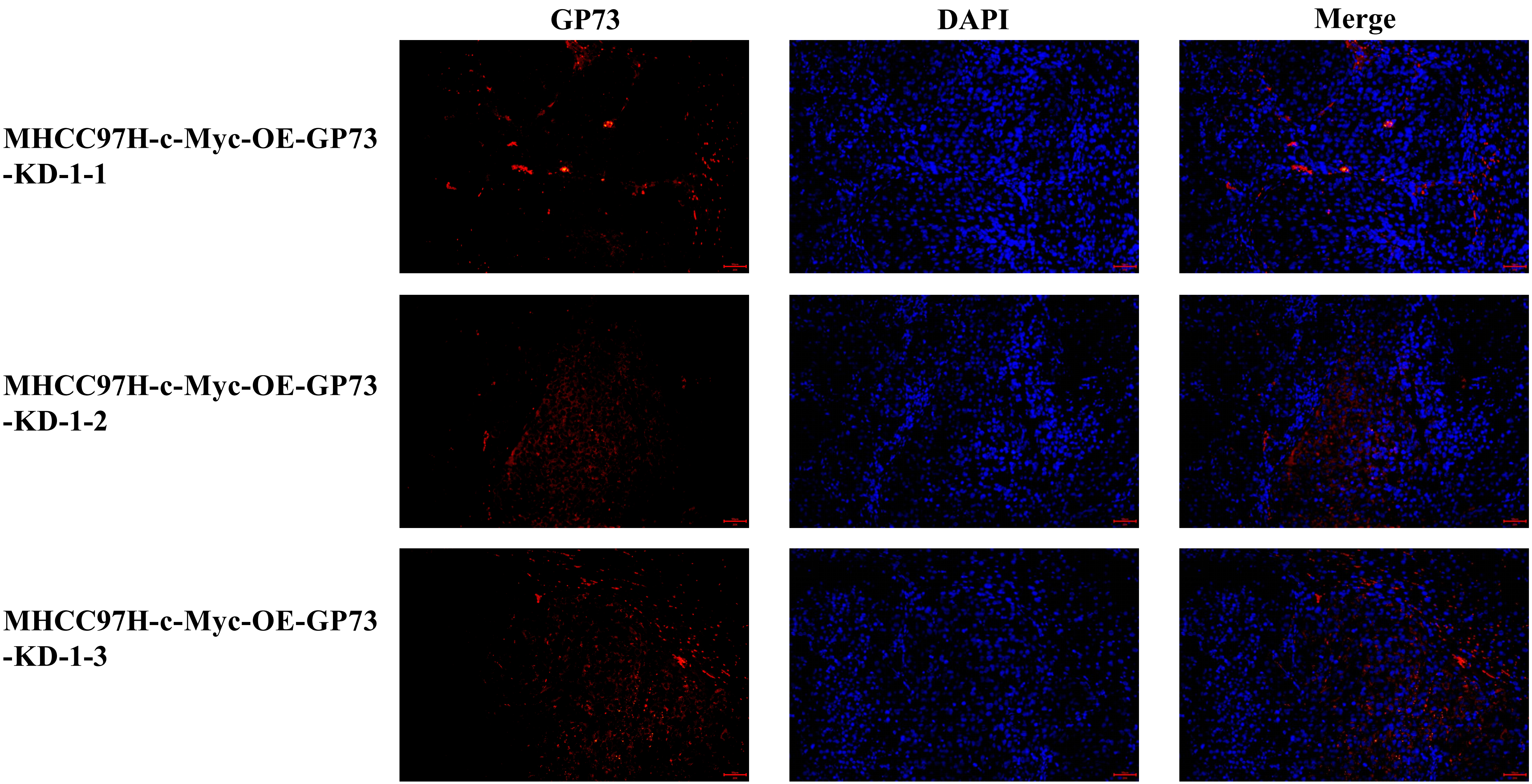
GP73
DAPI
Merge
MHCC97H-c-Myc-OE-GP73 -KD-1-1
MHCC97H-c-Myc-OE-GP73 -KD-1-2
MHCC97H-c-Myc-OE-GP73 -KD-1-3

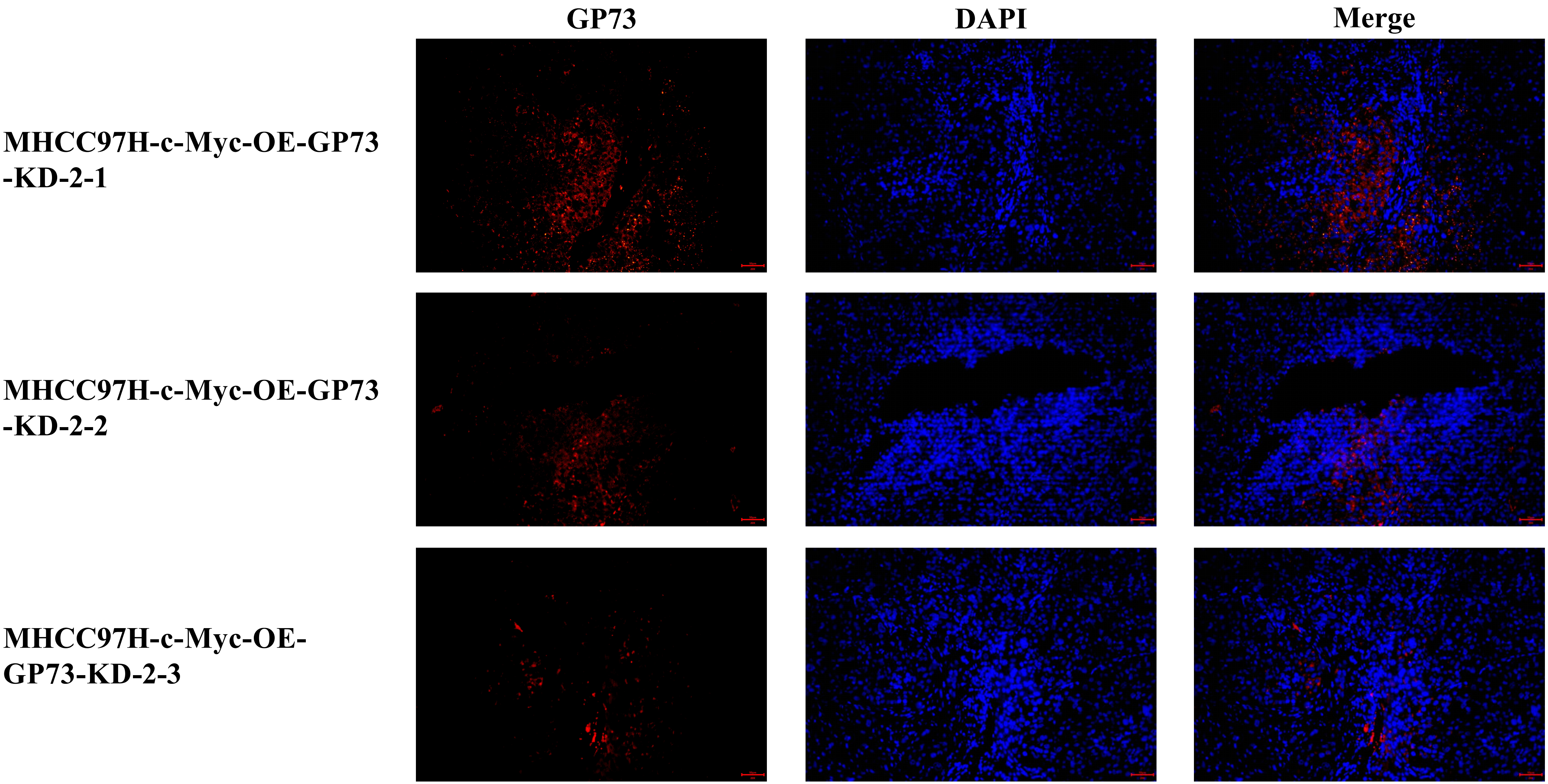
GP73
DAPI
Merge
MHCC97H-c-Myc-OE-GP73
-KD-2-1
MHCC97H-c-Myc-OE-GP73
-KD-2-2
MHCC97H-c-Myc-OE-
GP73-KD-2-3

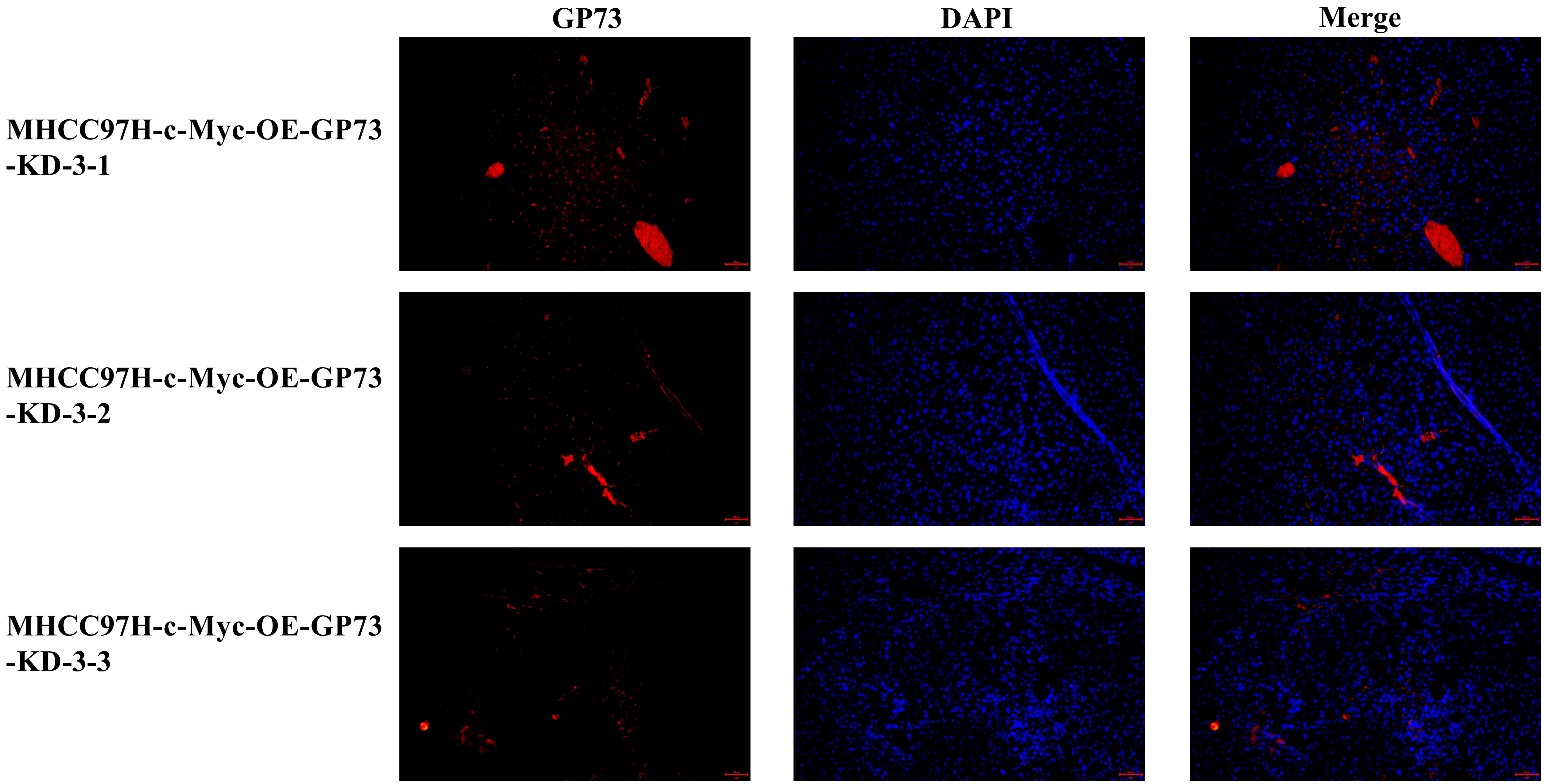
GP73
DAPI
Merge
MHCC97H-c-Myc-OE-GP73
-KD-3-1
MHCC97H-c-Myc-OE-GP73
-KD-3-2
MHCC97H-c-Myc-OE-GP73
-KD-3-3

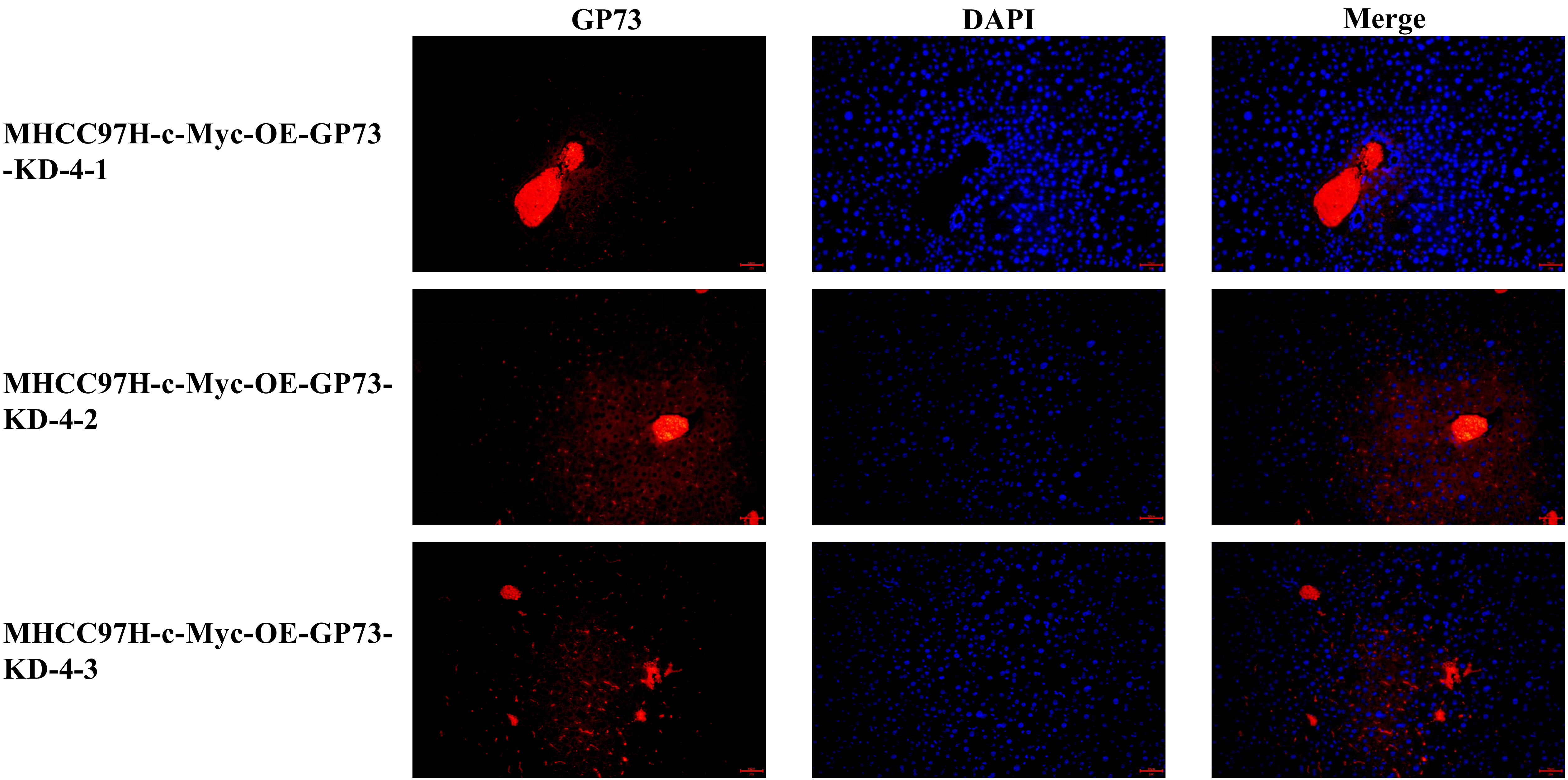
GP73
DAPI
Merge
MHCC97H-c-Myc-OE-GP73-KD-4-1
MHCC97H-c-Myc-OE-GP73-KD-4-2
MHCC97H-c-Myc-OE-GP73-KD-4-3

Immunofluorescence staining showing decreased expressions of STAT3 in the resected tumors originated from the MHCC97H-c-Myc-OE-GP73-KD cells compared to the MHCC97H-c-Myc-OE cells, original magnification, ×20. Data were representative of three similar observations or were shown as the mean±SD of three experiments.

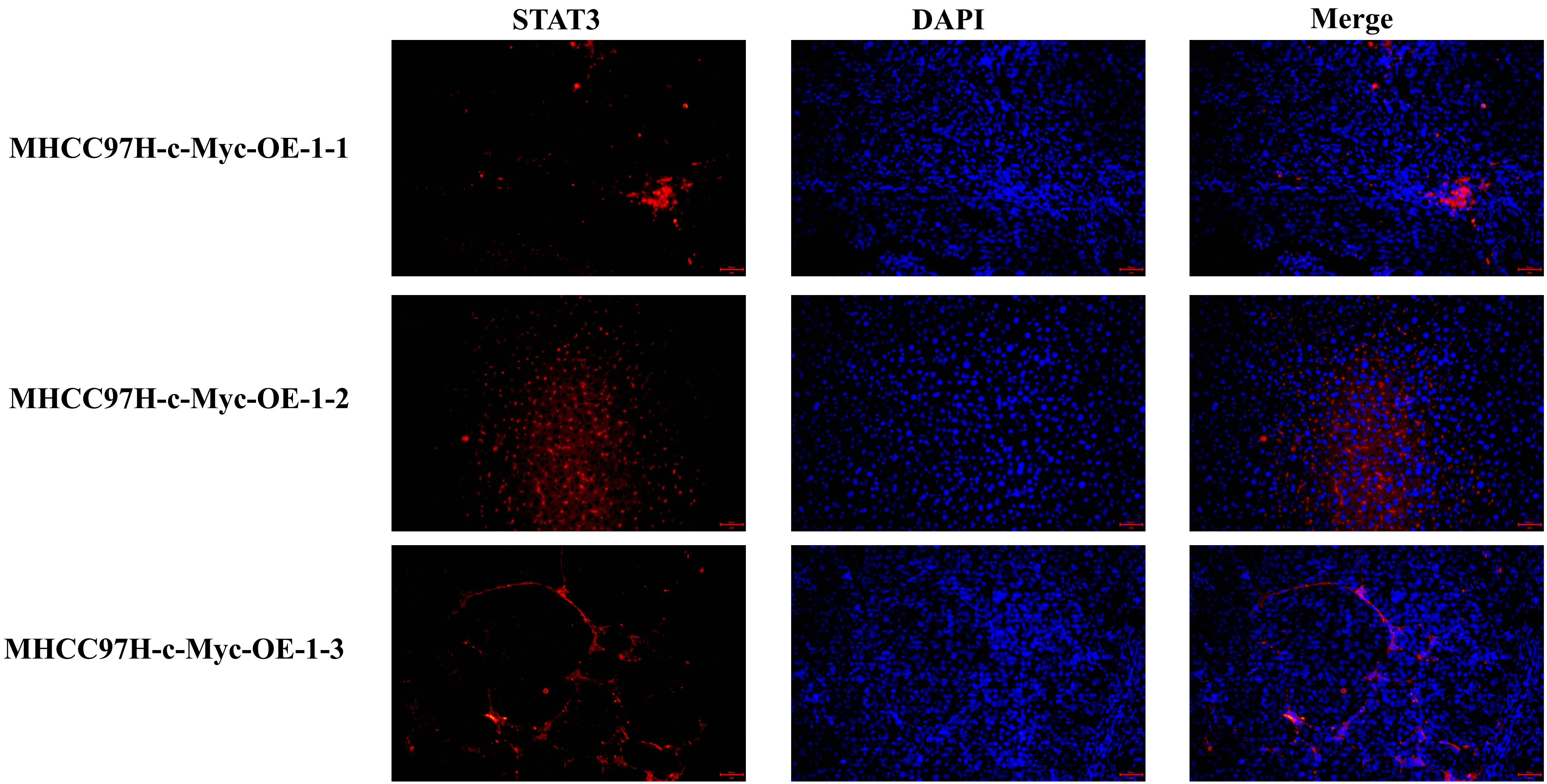
STAT3
DAPI
Merge
MHCC97H-c-Myc-OE-1-1
MHCC97H-c-Myc-OE-1-2
MHCC97H-c-Myc-OE-1-3

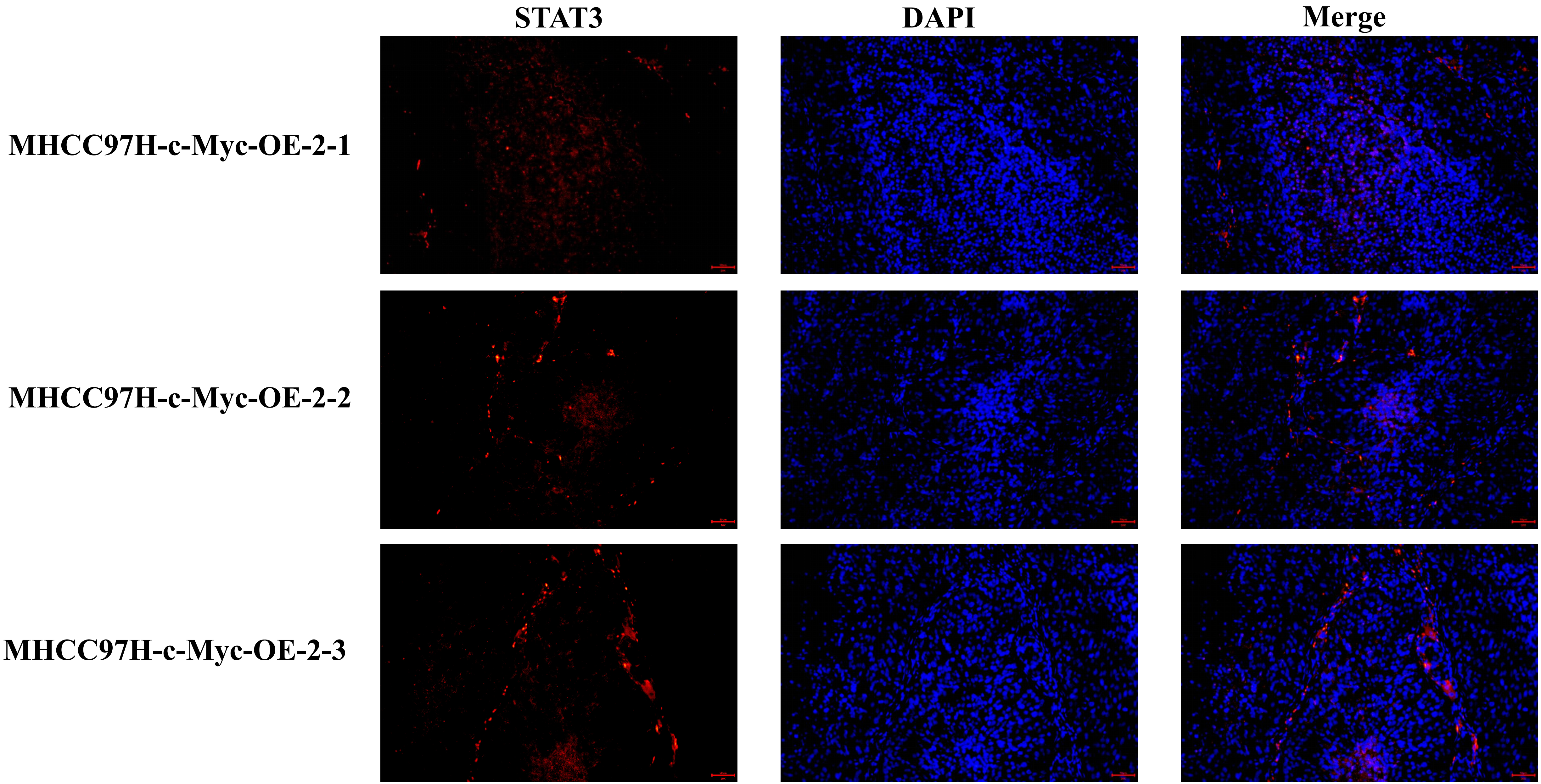
STAT3
DAPI
Merge
MHCC97H-c-Myc-OE-2-1
MHCC97H-c-Myc-OE-2-2
MHCC97H-c-Myc-OE-2-3

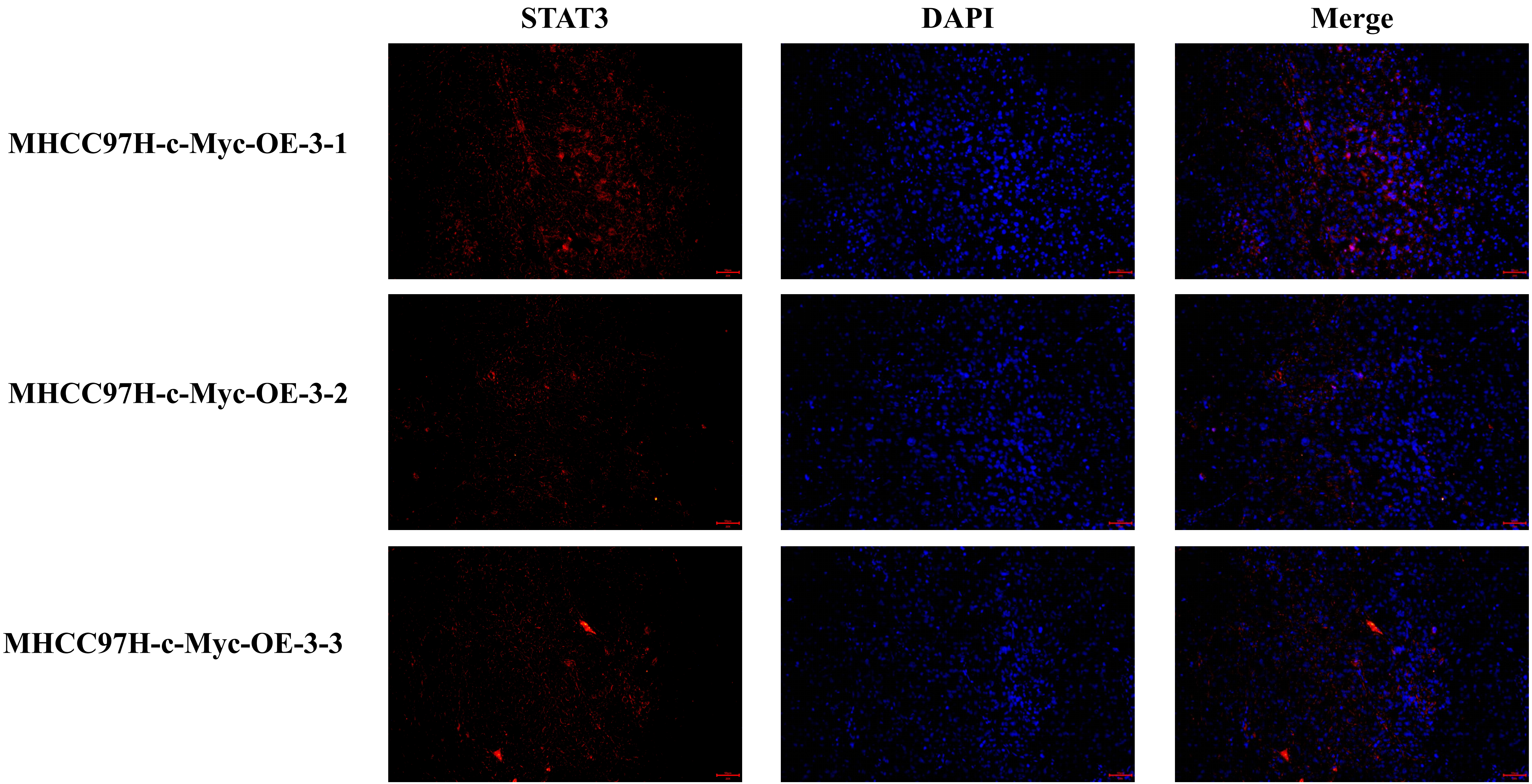
STAT3
DAPI
Merge
MHCC97H-c-Myc-OE-3-1
MHCC97H-c-Myc-OE-3-2
MHCC97H-c-Myc-OE-3-3

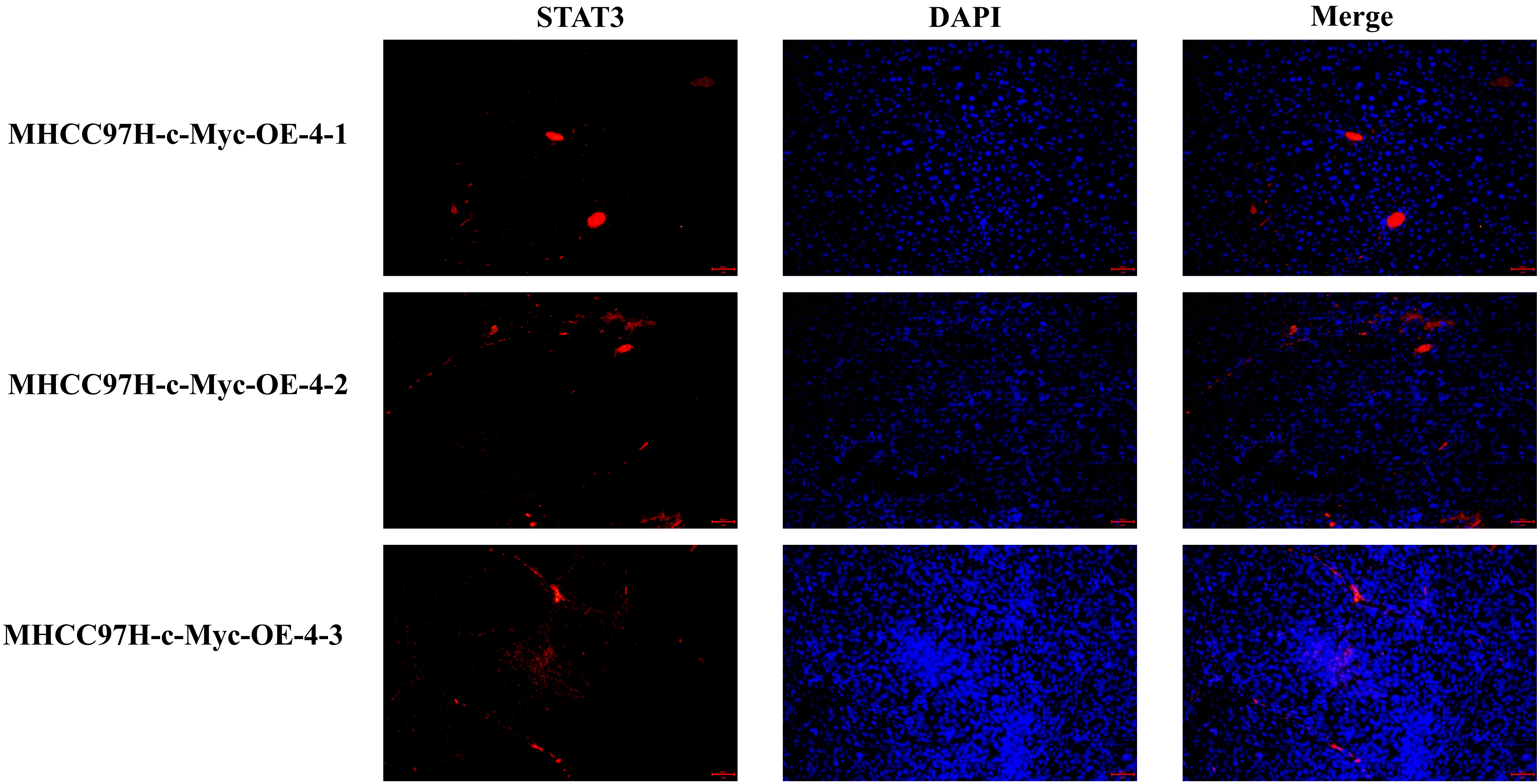
STAT3
DAPI
Merge
MHCC97H-c-Myc-OE-4-1
MHCC97H-c-Myc-OE-4-2
MHCC97H-c-Myc-OE-4-3

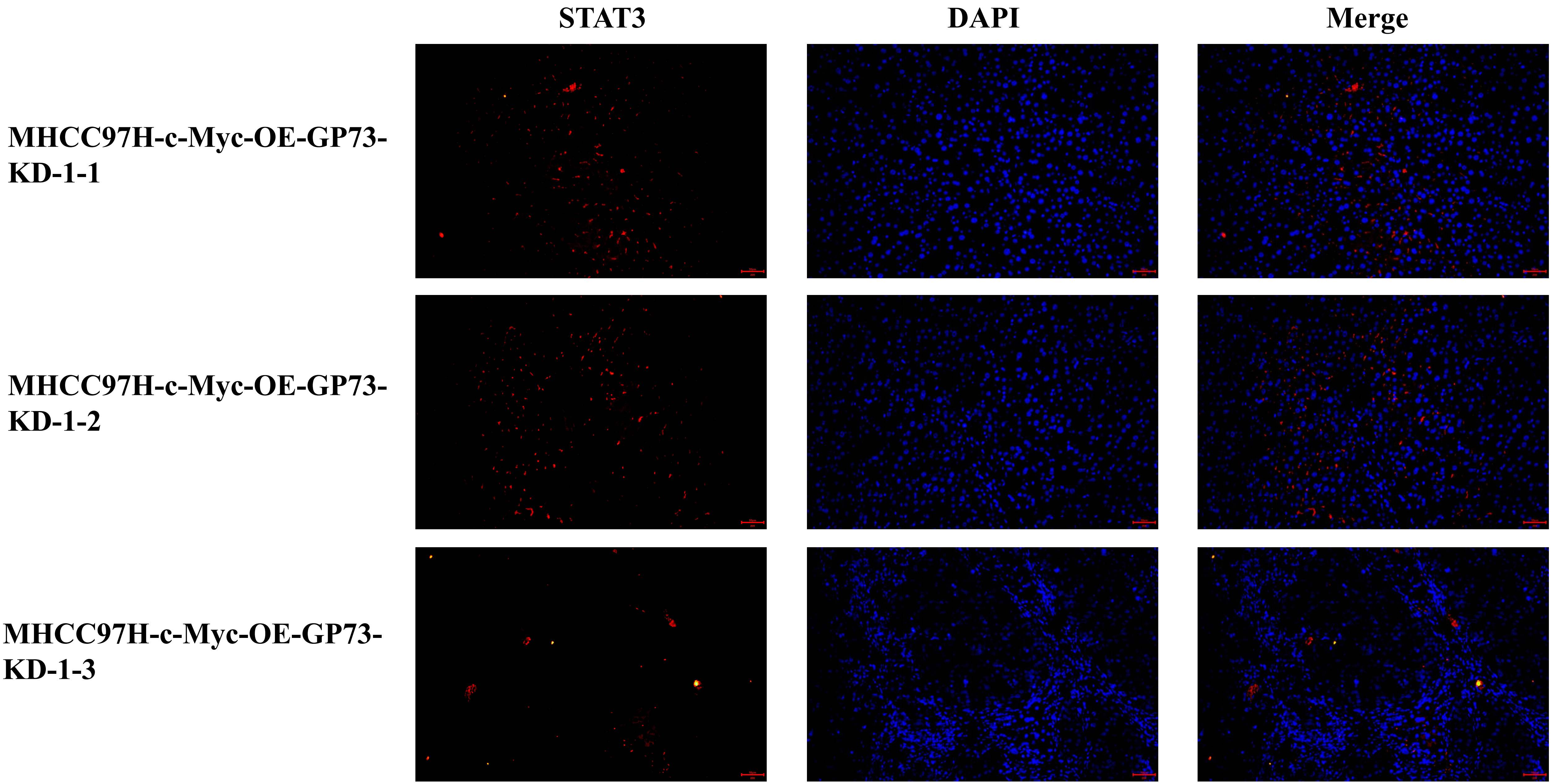
STAT3
DAPI
Merge
MHCC97H-c-Myc-OE-GP73-KD-1-1
MHCC97H-c-Myc-OE-GP73-KD-1-2
MHCC97H-c-Myc-OE-GP73-KD-1-3

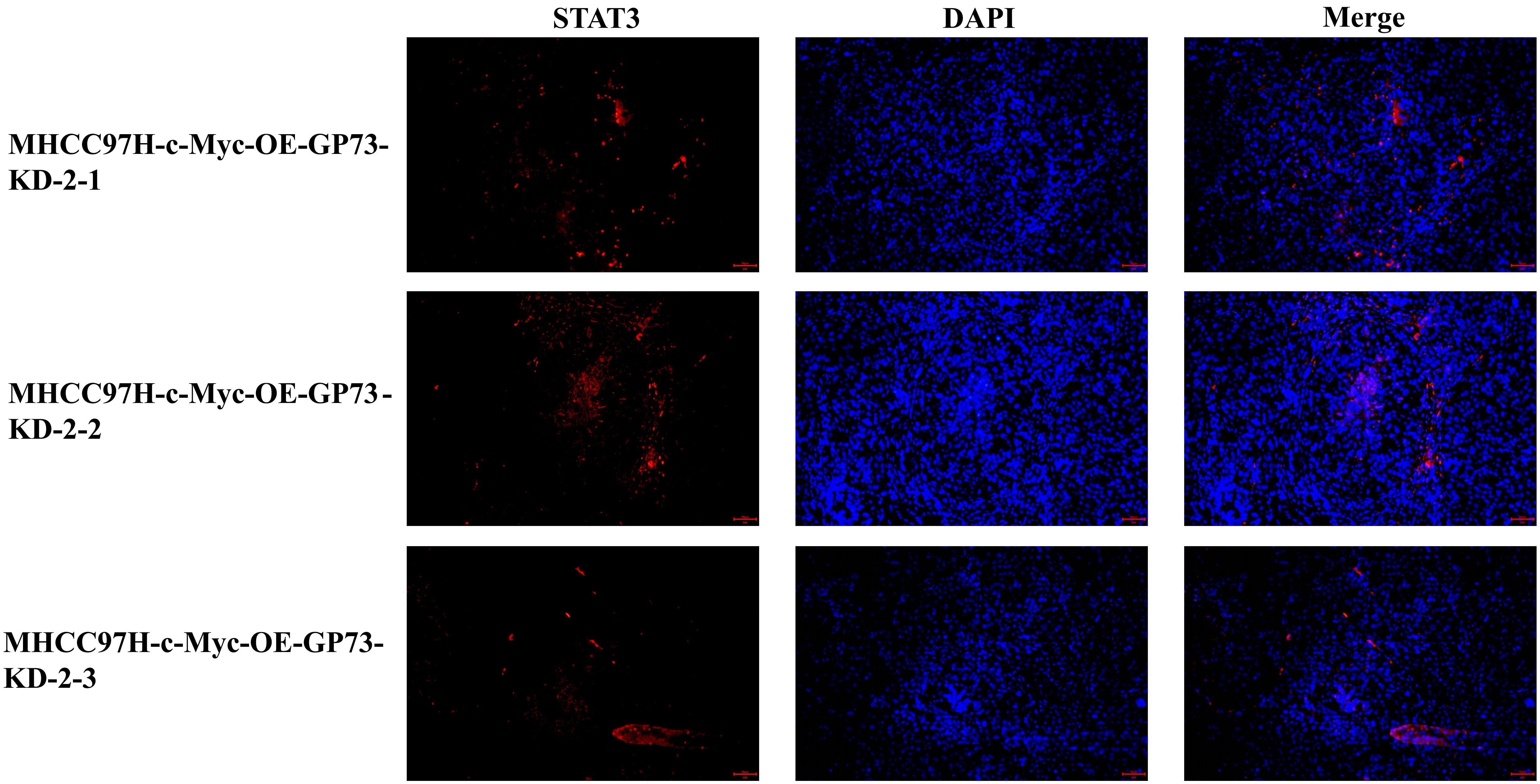
STAT3
DAPI
Merge
MHCC97H-c-Myc-OE-GP73-KD-2-1
MHCC97H-c-Myc-OE-GP73-KD-2-2
MHCC97H-c-Myc-OE-GP73-KD-2-3

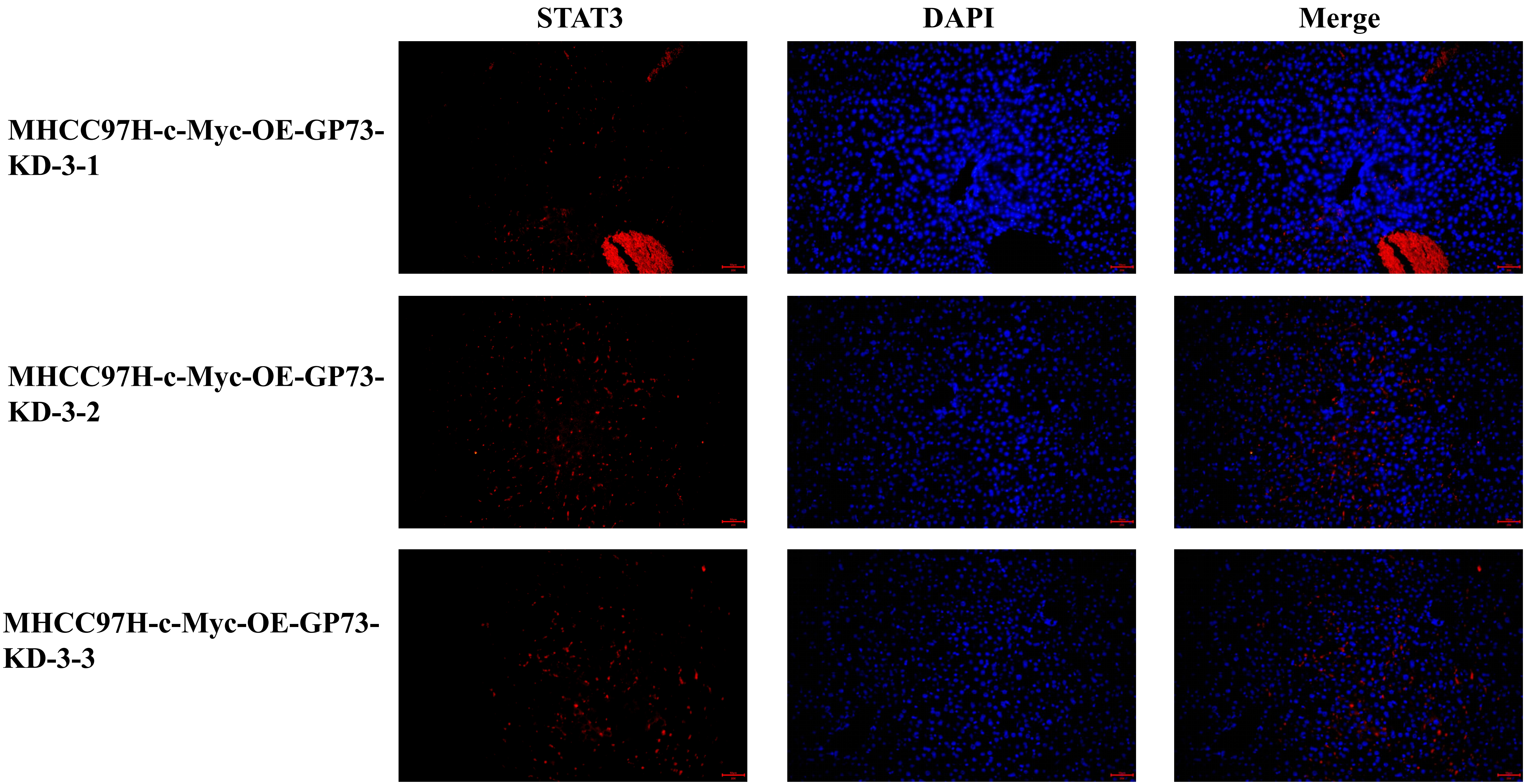
STAT3
DAPI
Merge
MHCC97H-c-Myc-OE-GP73-KD-3-1
MHCC97H-c-Myc-OE-GP73-KD-3-2
MHCC97H-c-Myc-OE-GP73-KD-3-3

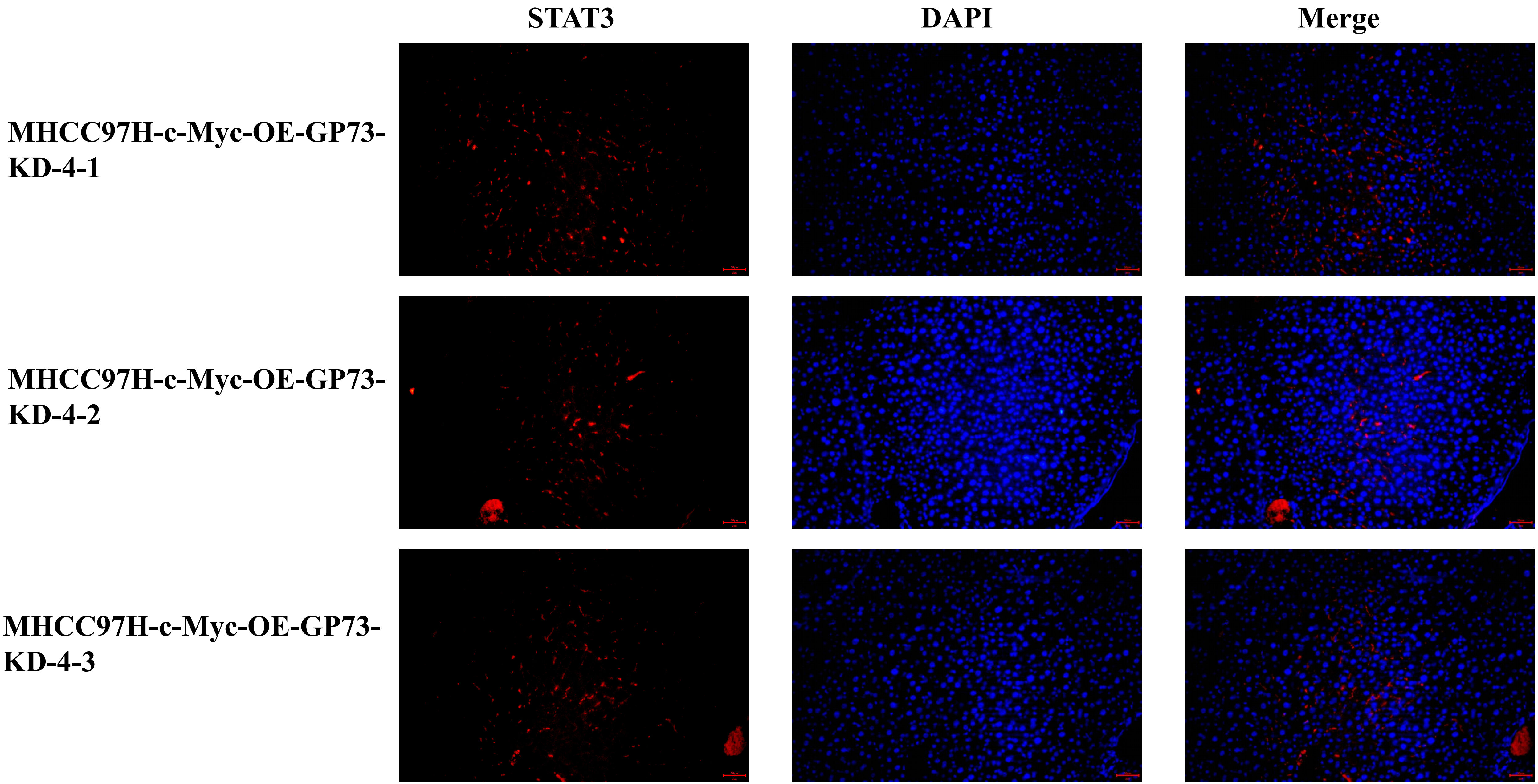
STAT3
DAPI
Merge
MHCC97H-c-Myc-OE-GP73-KD-4-1
MHCC97H-c-Myc-OE-GP73-KD-4-2
MHCC97H-c-Myc-OE-GP73-KD-4-3

Immunofluorescence staining showing decreased expressions of CD34 in the resected tumors originated from the MHCC97H-c-Myc-OE-GP73-KD cells compared to the MHCC97H-c-Myc-OE cells, original magnification, ×20. Data were representative of three similar observations or were shown as the mean±SD of three experiments.

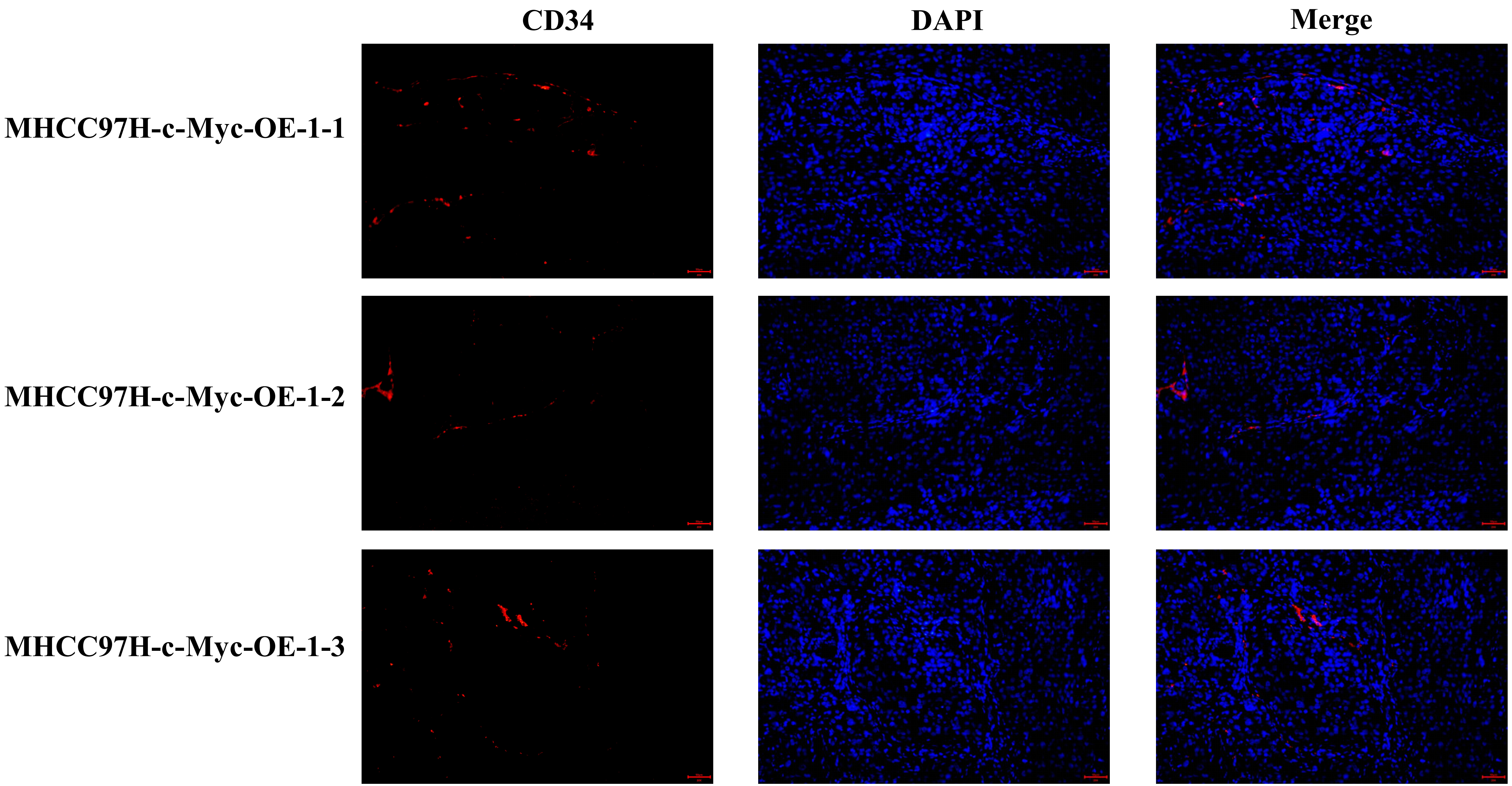
CD34
DAPI
Merge
MHCC97H-c-Myc-OE-1-1
MHCC97H-c-Myc-OE-1-2
MHCC97H-c-Myc-OE-1-3

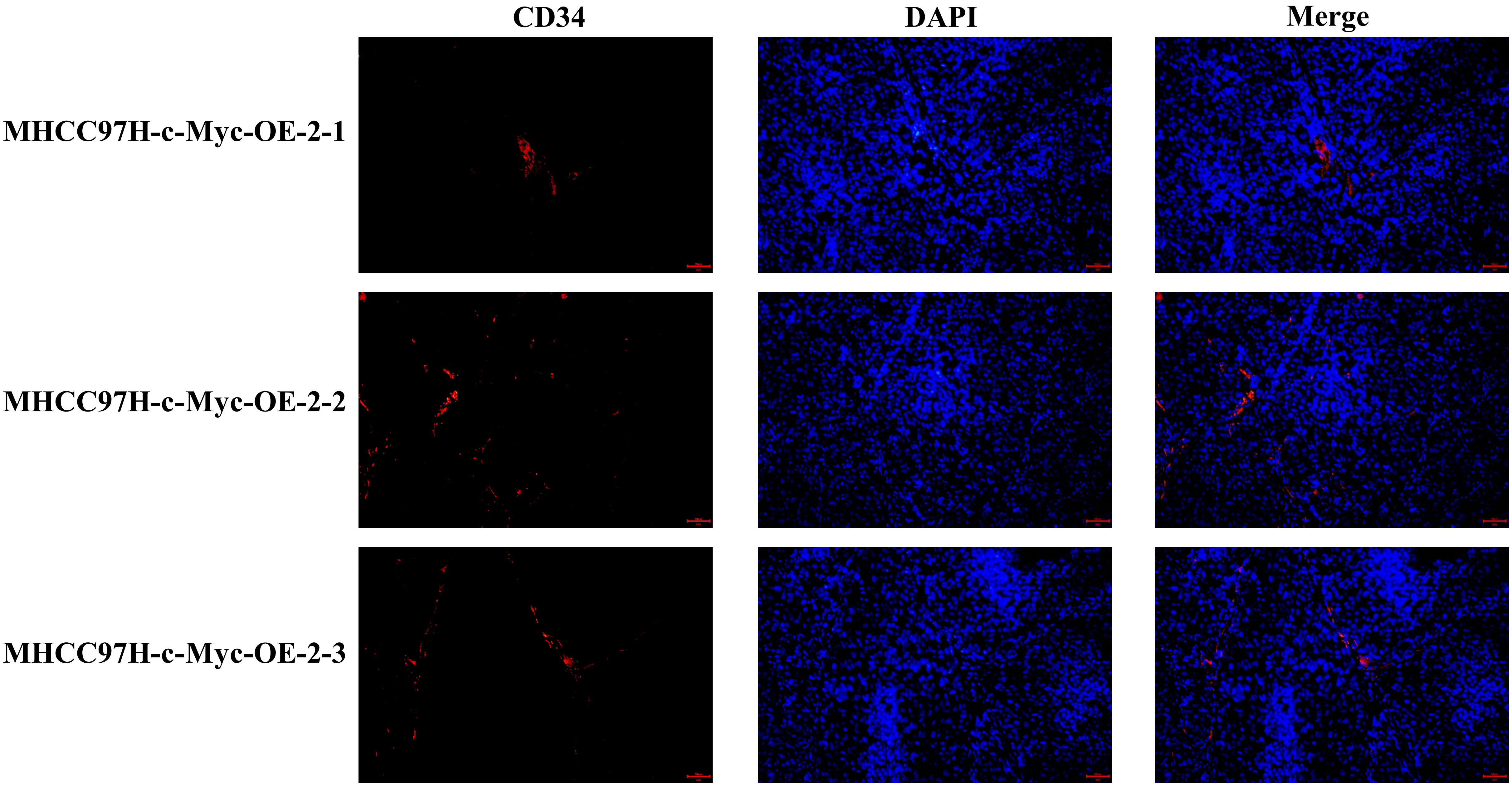
CD34
DAPI
Merge
MHCC97H-c-Myc-OE-2-1
MHCC97H-c-Myc-OE-2-2
MHCC97H-c-Myc-OE-2-3

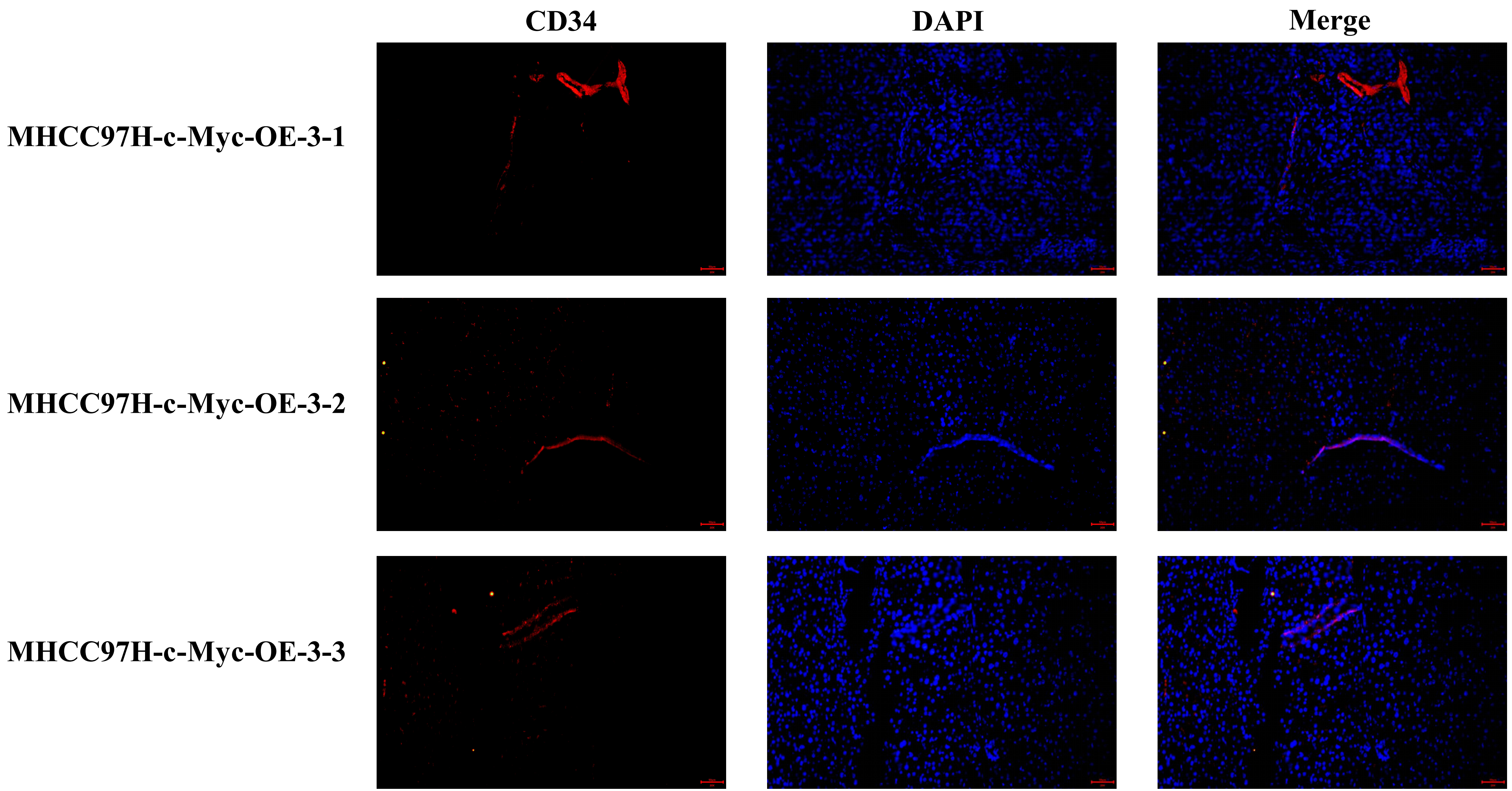
CD34
DAPI
Merge
MHCC97H-c-Myc-OE-3-1
MHCC97H-c-Myc-OE-3-2
MHCC97H-c-Myc-OE-3-3

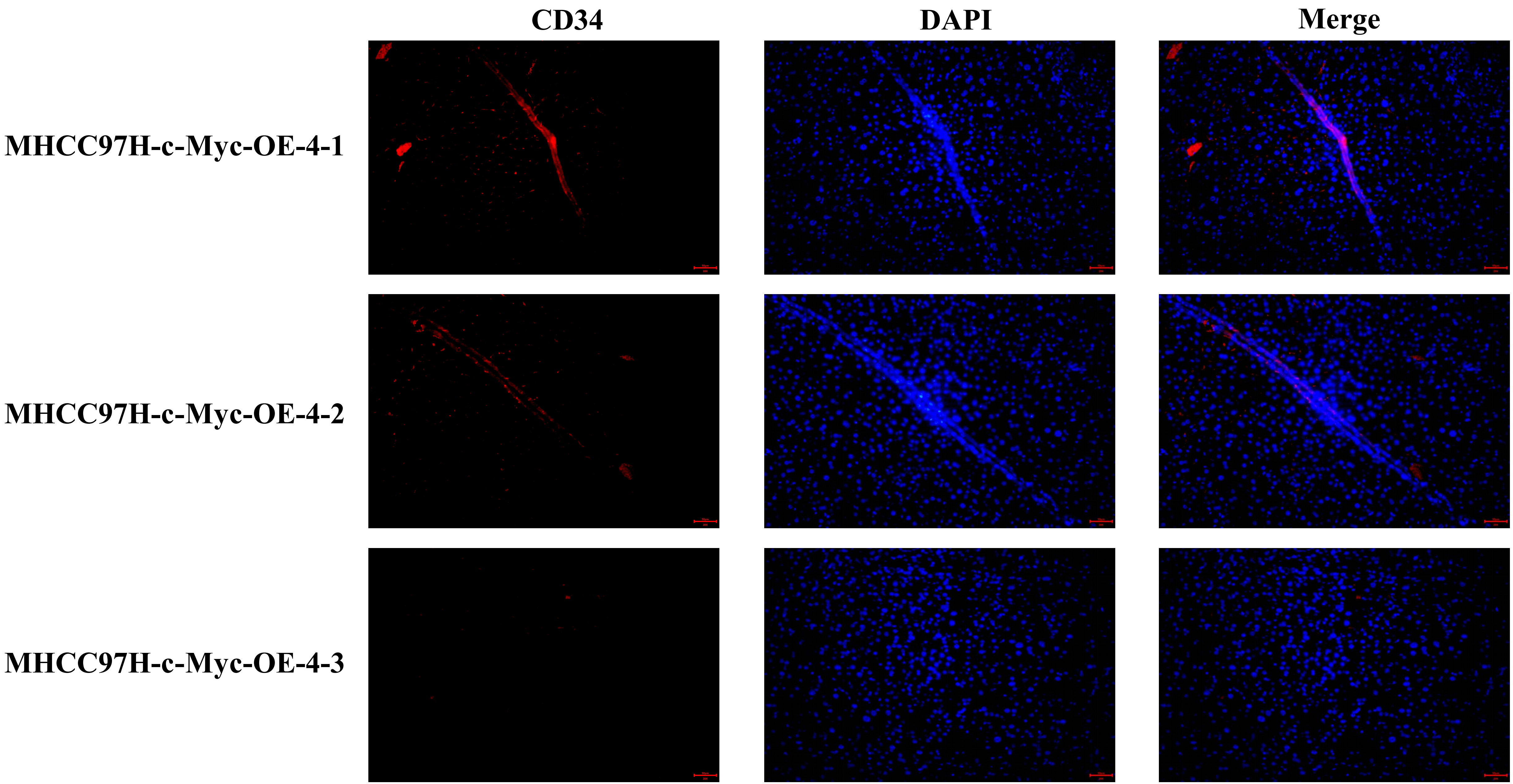
CD34
DAPI
Merge
MHCC97H-c-Myc-OE-4-1
MHCC97H-c-Myc-OE-4-2
MHCC97H-c-Myc-OE-4-3

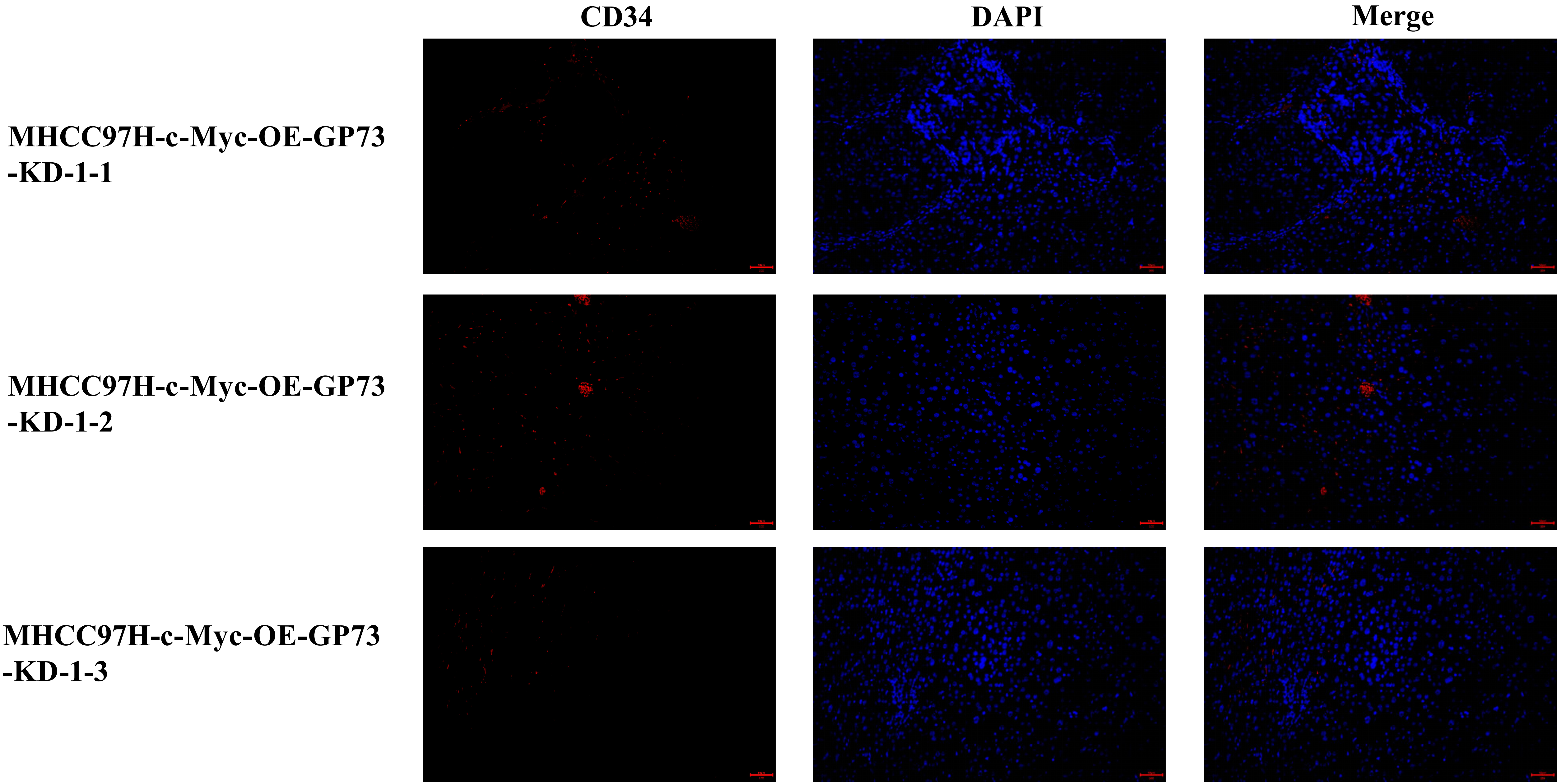
CD34
DAPI
Merge
MHCC97H-c-Myc-OE-GP73 -KD-1-1
MHCC97H-c-Myc-OE-GP73 -KD-1-2
MHCC97H-c-Myc-OE-GP73 -KD-1-3

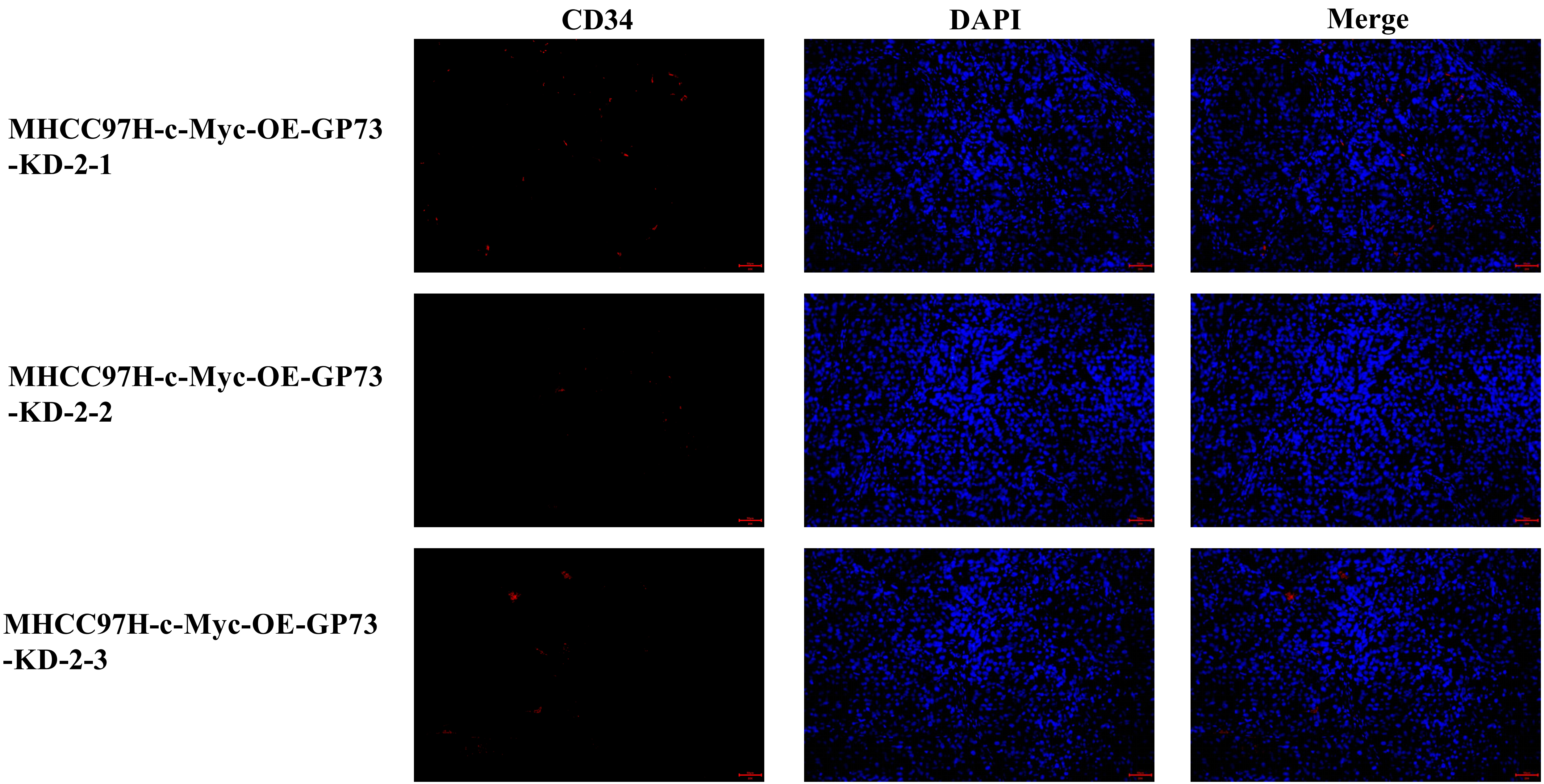
CD34
DAPI
Merge
MHCC97H-c-Myc-OE-GP73
-KD-2-1
MHCC97H-c-Myc-OE-GP73
-KD-2-2
MHCC97H-c-Myc-OE-GP73
-KD-2-3

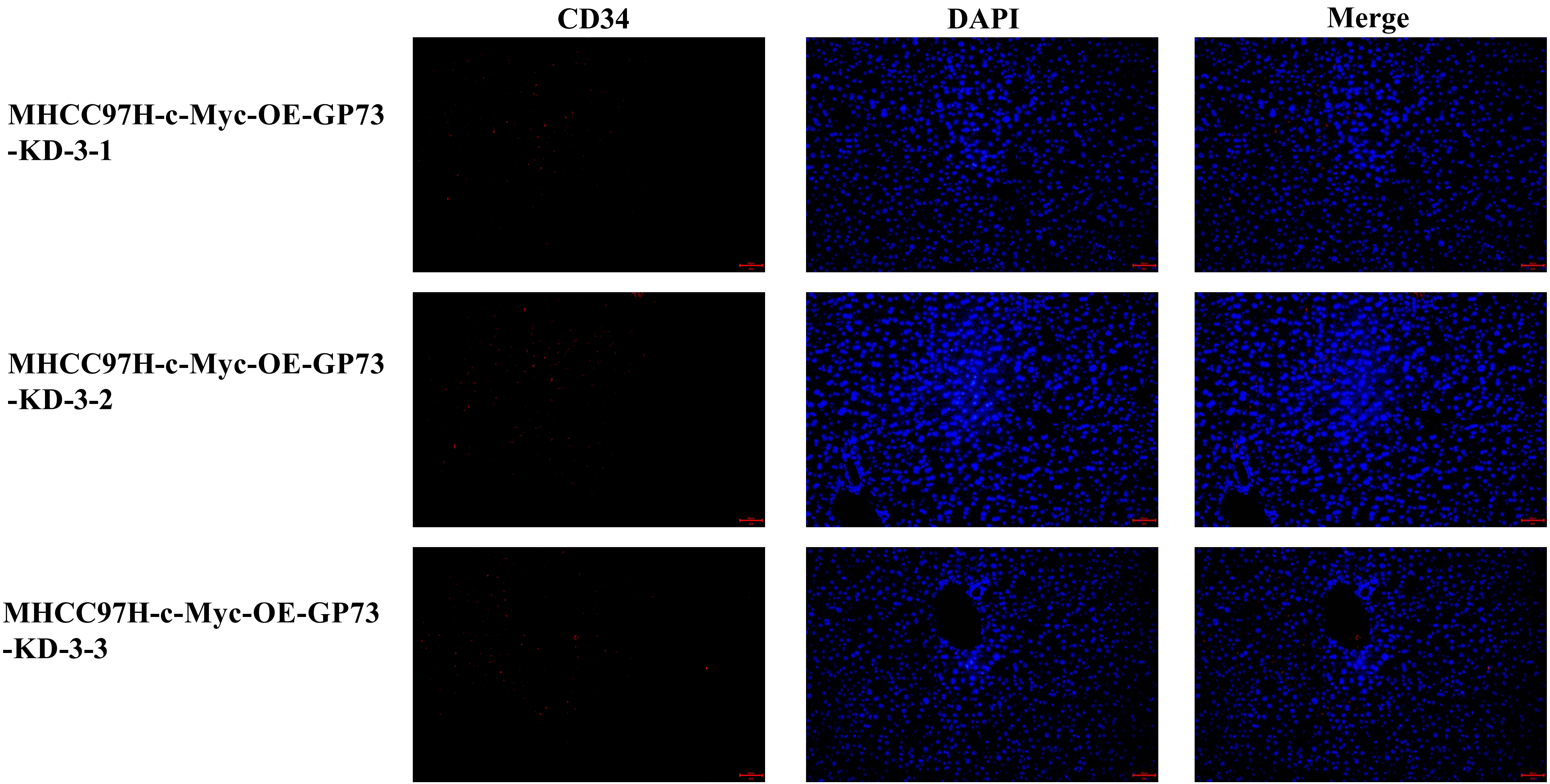
CD34
DAPI
Merge
MHCC97H-c-Myc-OE-GP73
-KD-3-1
MHCC97H-c-Myc-OE-GP73
-KD-3-2
MHCC97H-c-Myc-OE-GP73
-KD-3-3

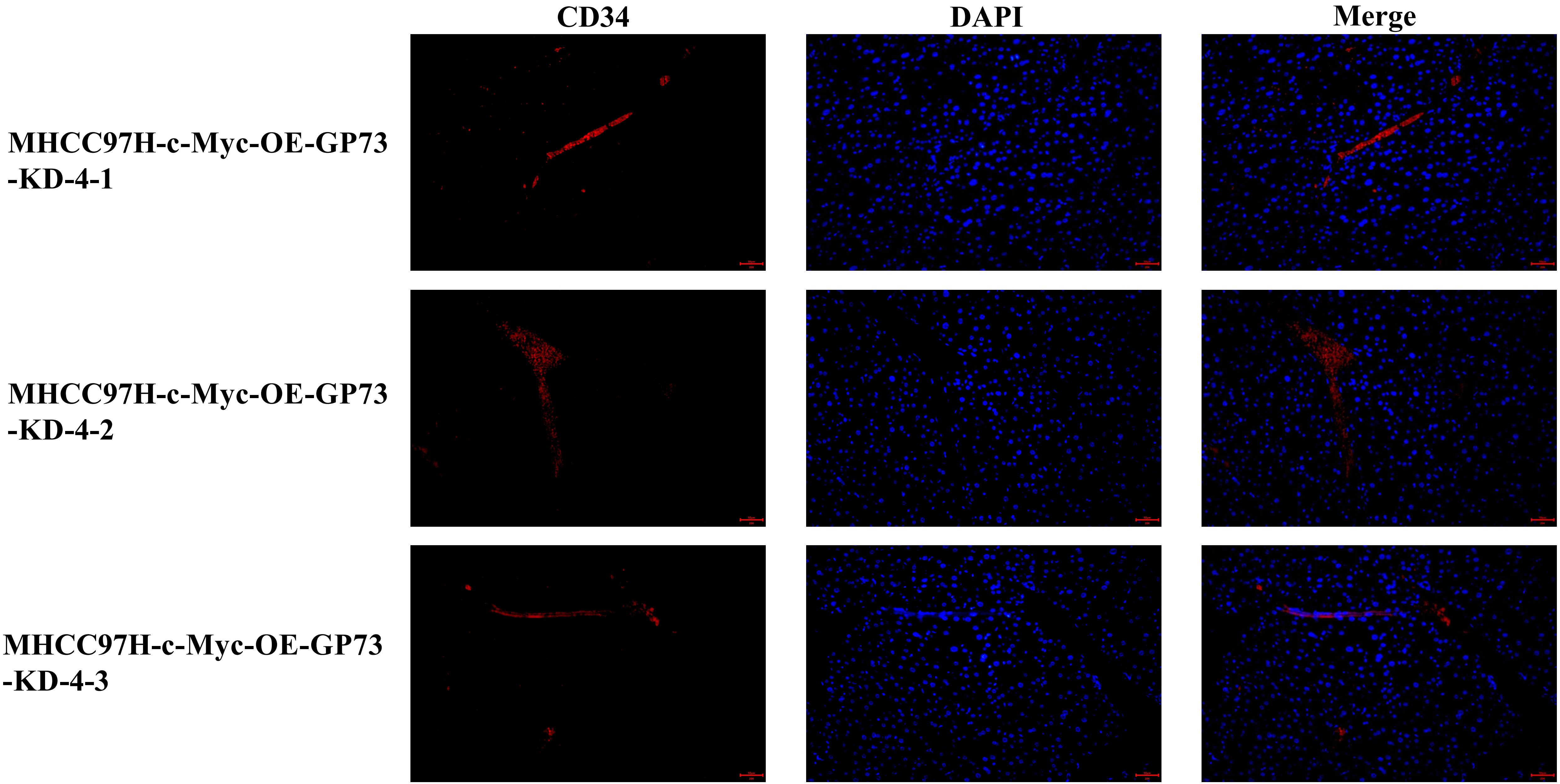
CD34
DAPI
Merge
MHCC97H-c-Myc-OE-GP73
-KD-4-1
MHCC97H-c-Myc-OE-GP73
-KD-4-2
MHCC97H-c-Myc-OE-GP73
-KD-4-3

Immunofluorescence staining showing decreased expressions of CD31 in the resected tumors originated from the MHCC97H-c-Myc-OE-GP73-KD cells compared to the MHCC97H-c-Myc-OE cells, original magnification, ×20. Data were representative of three similar observations or were shown as the mean±SD of three experiments.

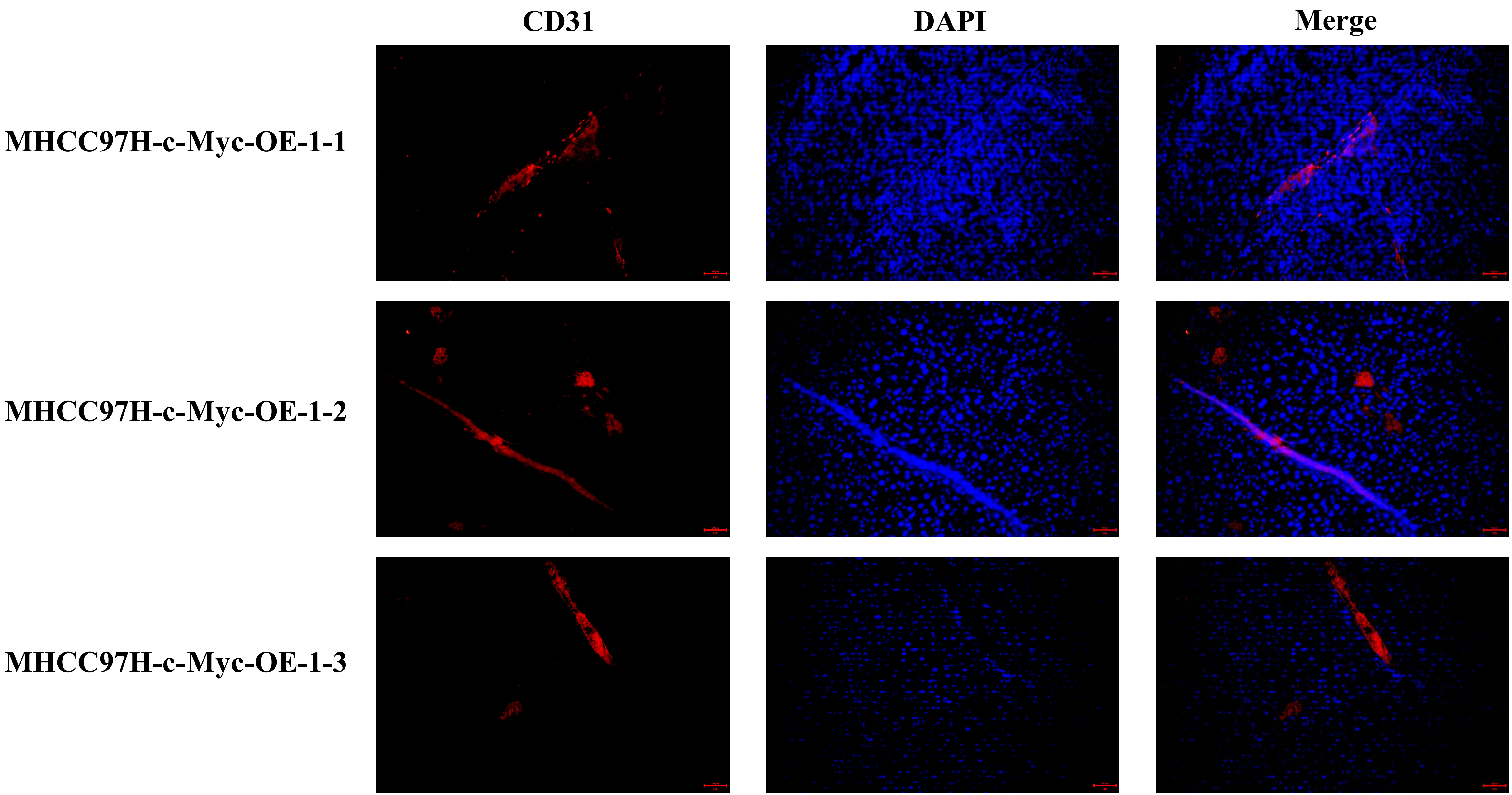
CD31
DAPI
Merge
MHCC97H-c-Myc-OE-1-1
MHCC97H-c-Myc-OE-1-2
MHCC97H-c-Myc-OE-1-3

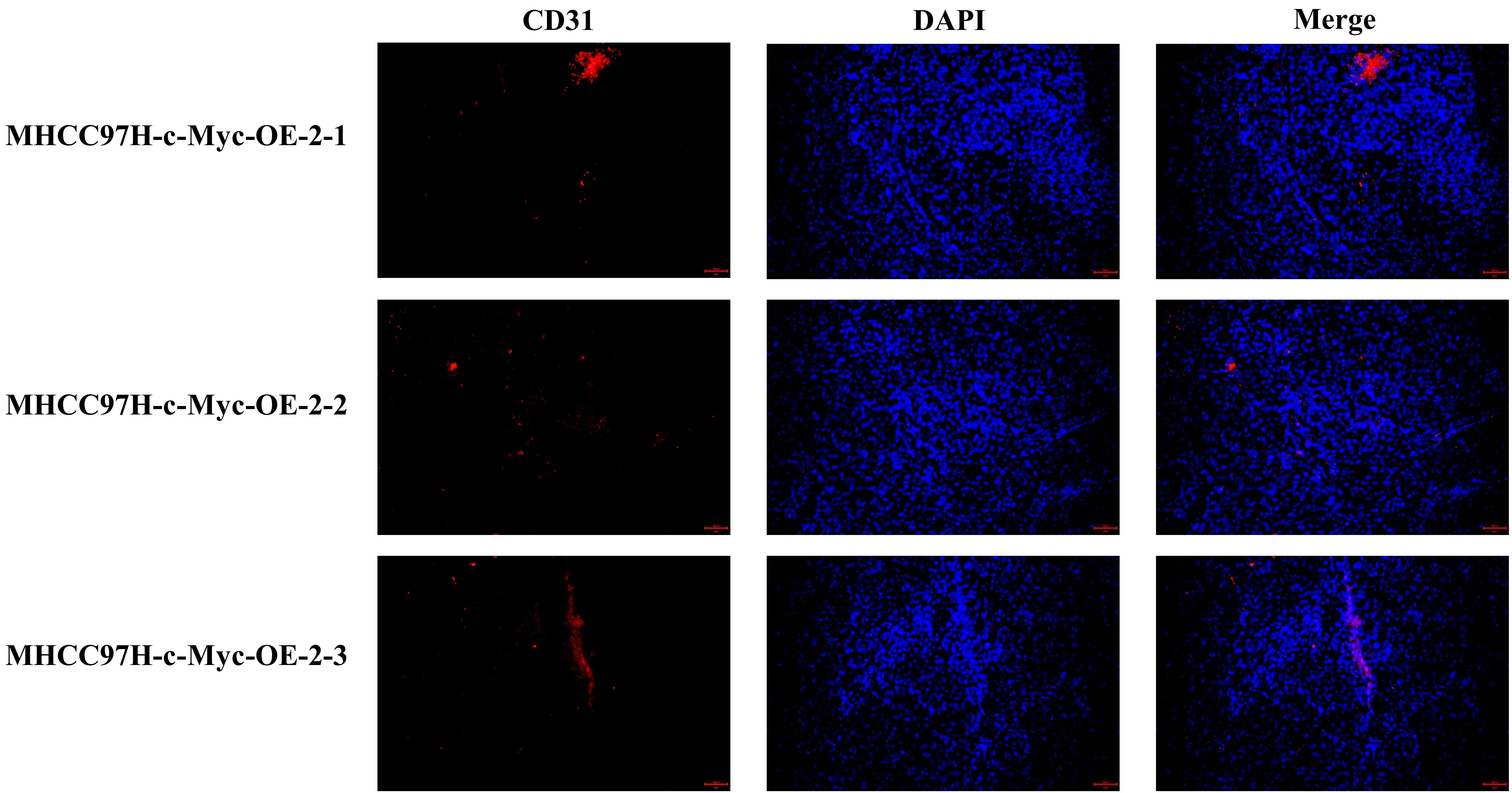
CD31
DAPI
Merge
MHCC97H-c-Myc-OE-2-1
MHCC97H-c-Myc-OE-2-2
MHCC97H-c-Myc-OE-2-3

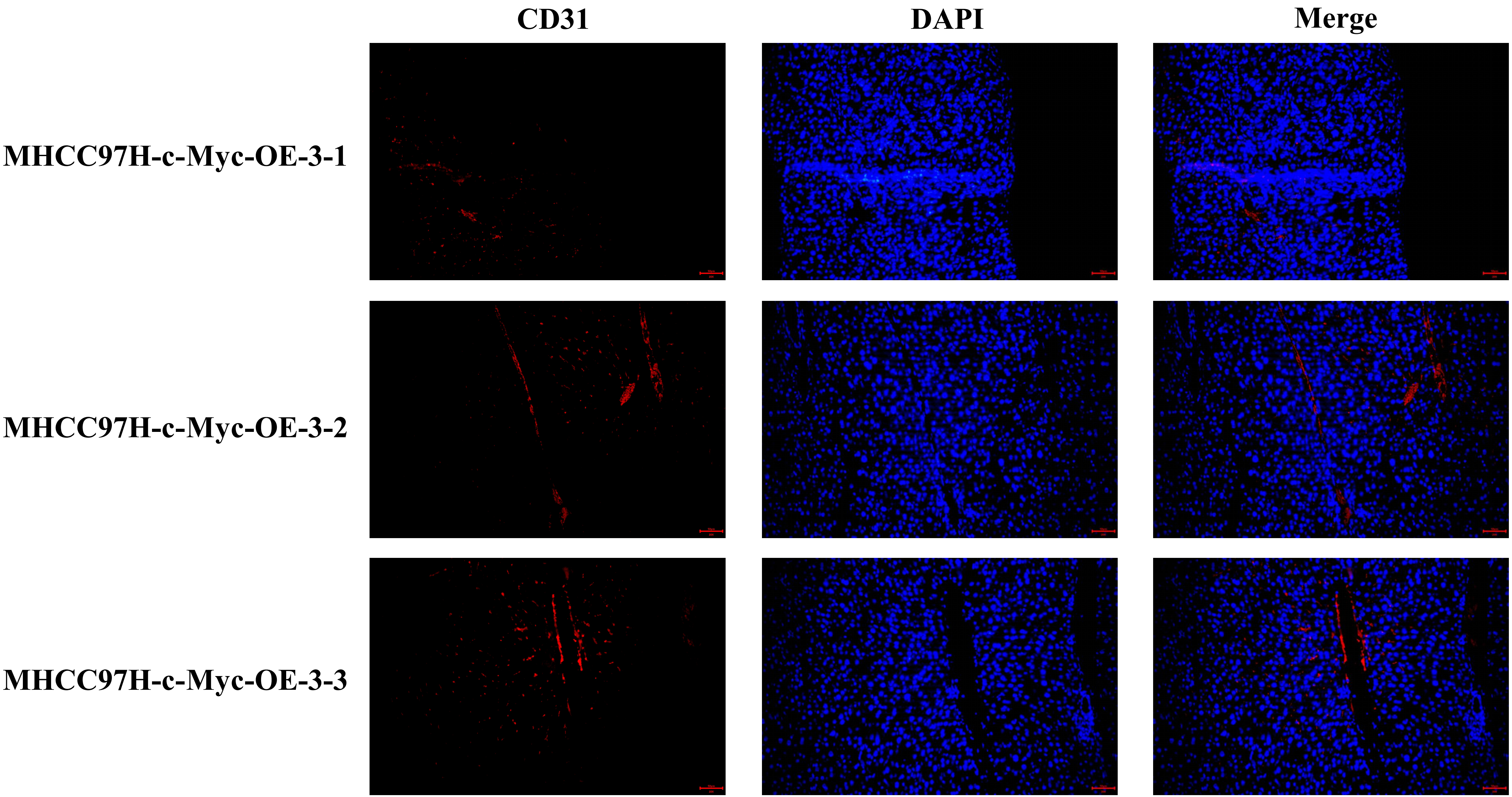
CD31
DAPI
Merge
MHCC97H-c-Myc-OE-3-1
MHCC97H-c-Myc-OE-3-2
MHCC97H-c-Myc-OE-3-3

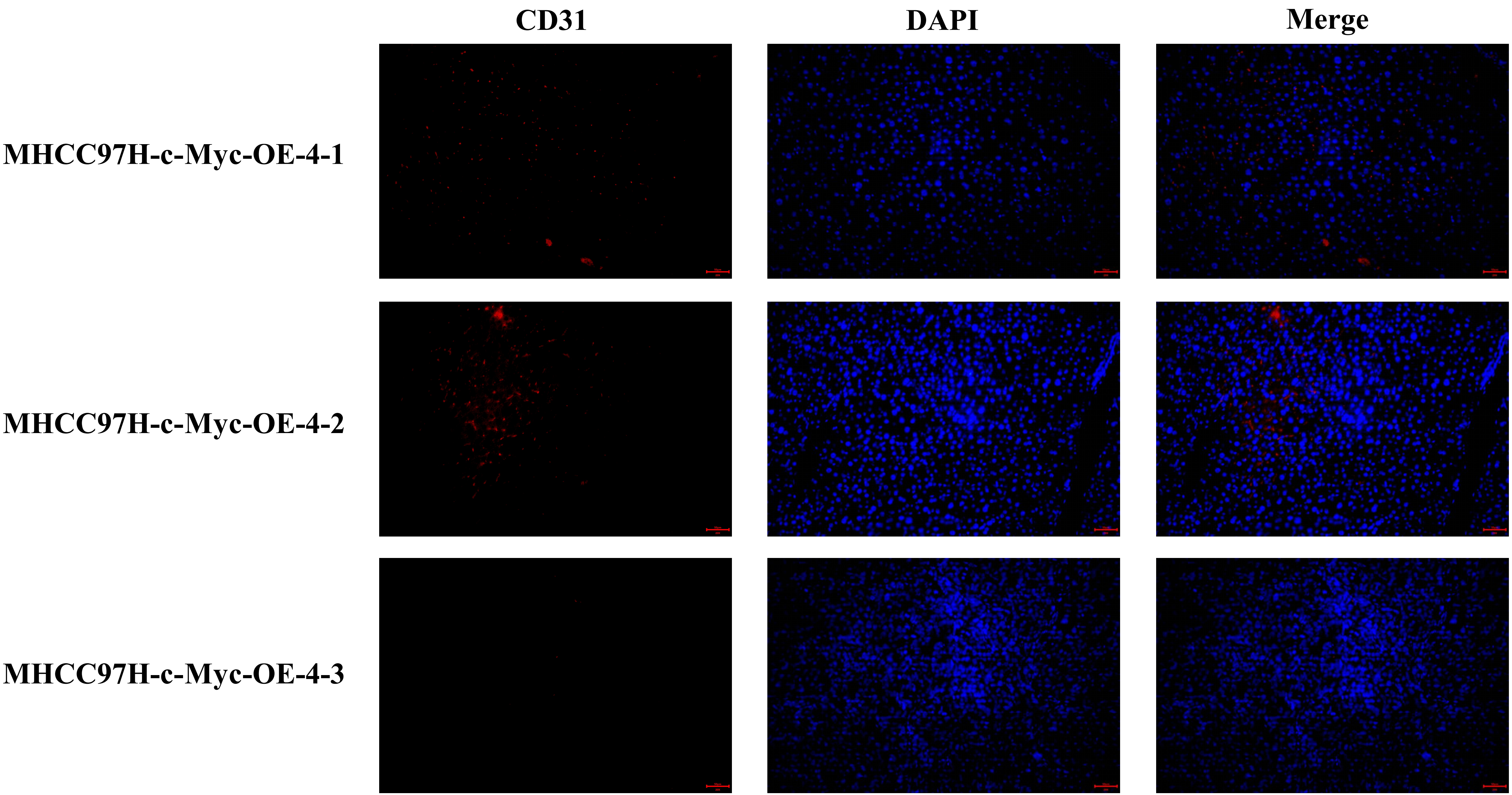
CD31
DAPI
Merge
MHCC97H-c-Myc-OE-4-1
MHCC97H-c-Myc-OE-4-2
MHCC97H-c-Myc-OE-4-3

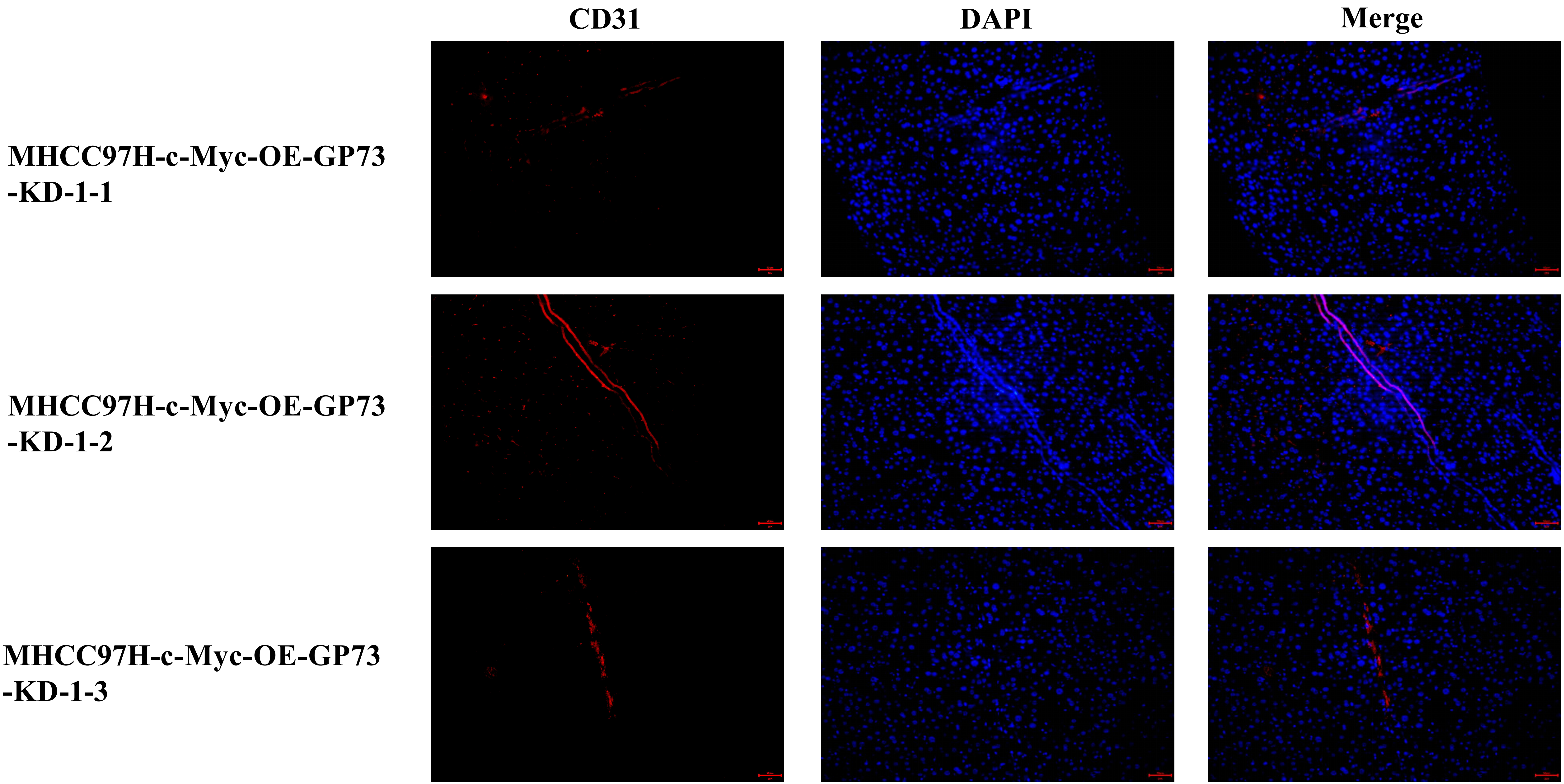
CD31
DAPI
Merge
MHCC97H-c-Myc-OE-GP73 -KD-1-1
MHCC97H-c-Myc-OE-GP73 -KD-1-2
MHCC97H-c-Myc-OE-GP73 -KD-1-3

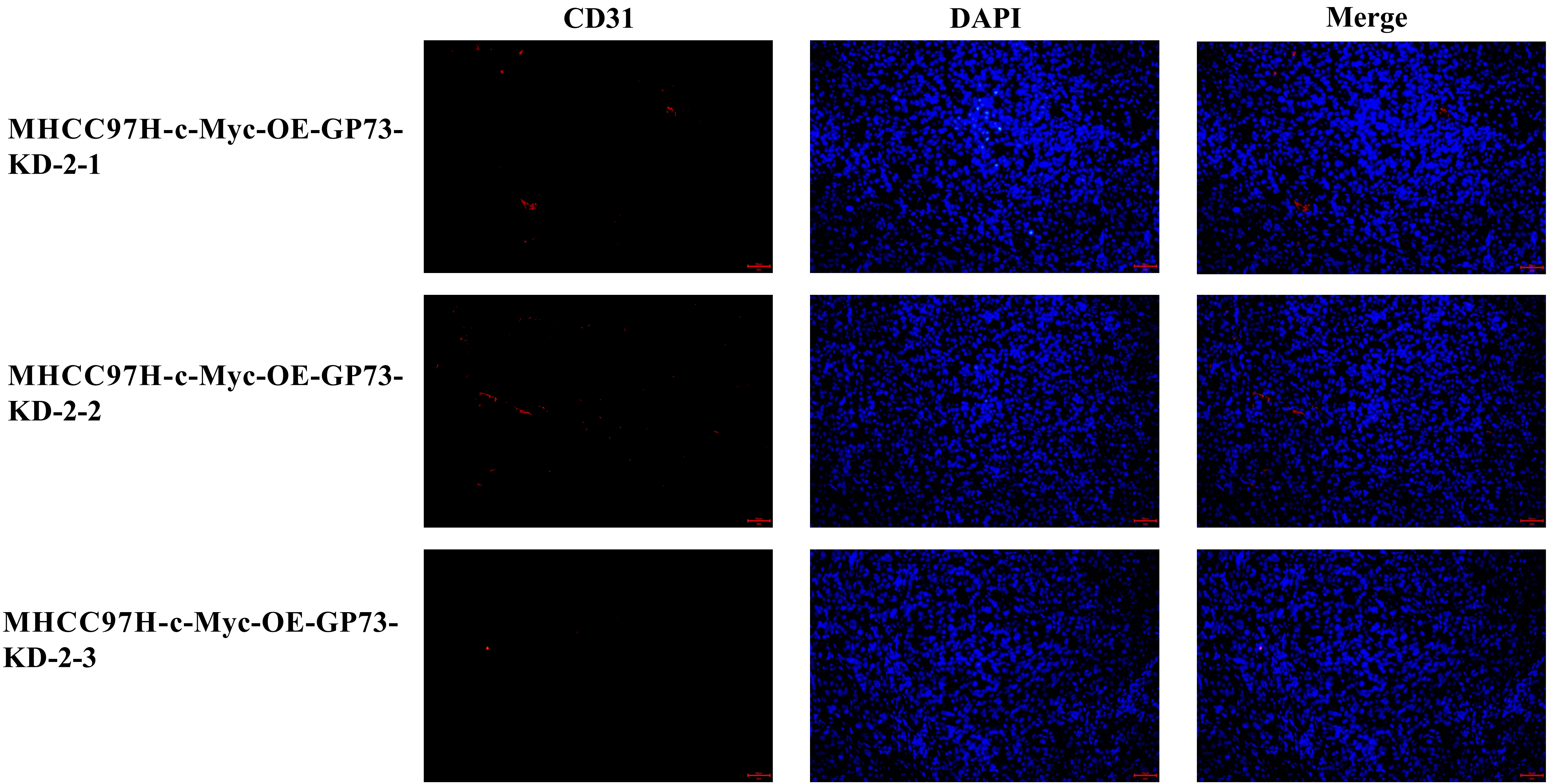
CD31
DAPI
Merge
MHCC97H-c-Myc-OE-GP73-KD-2-1
MHCC97H-c-Myc-OE-GP73-KD-2-2
MHCC97H-c-Myc-OE-GP73-KD-2-3

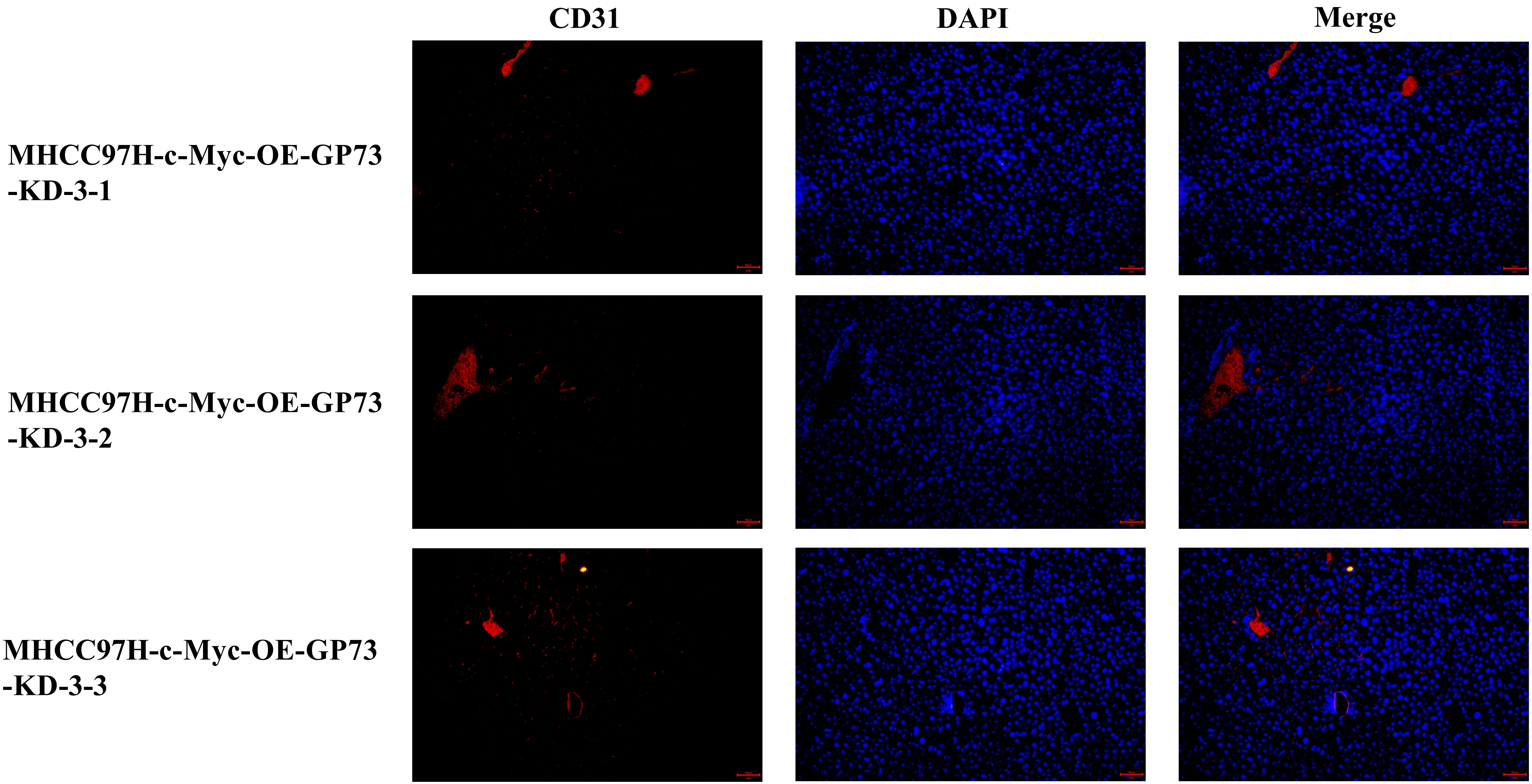
CD31
DAPI
Merge
MHCC97H-c-Myc-OE-GP73
-KD-3-1
MHCC97H-c-Myc-OE-GP73
-KD-3-2
MHCC97H-c-Myc-OE-GP73
-KD-3-3

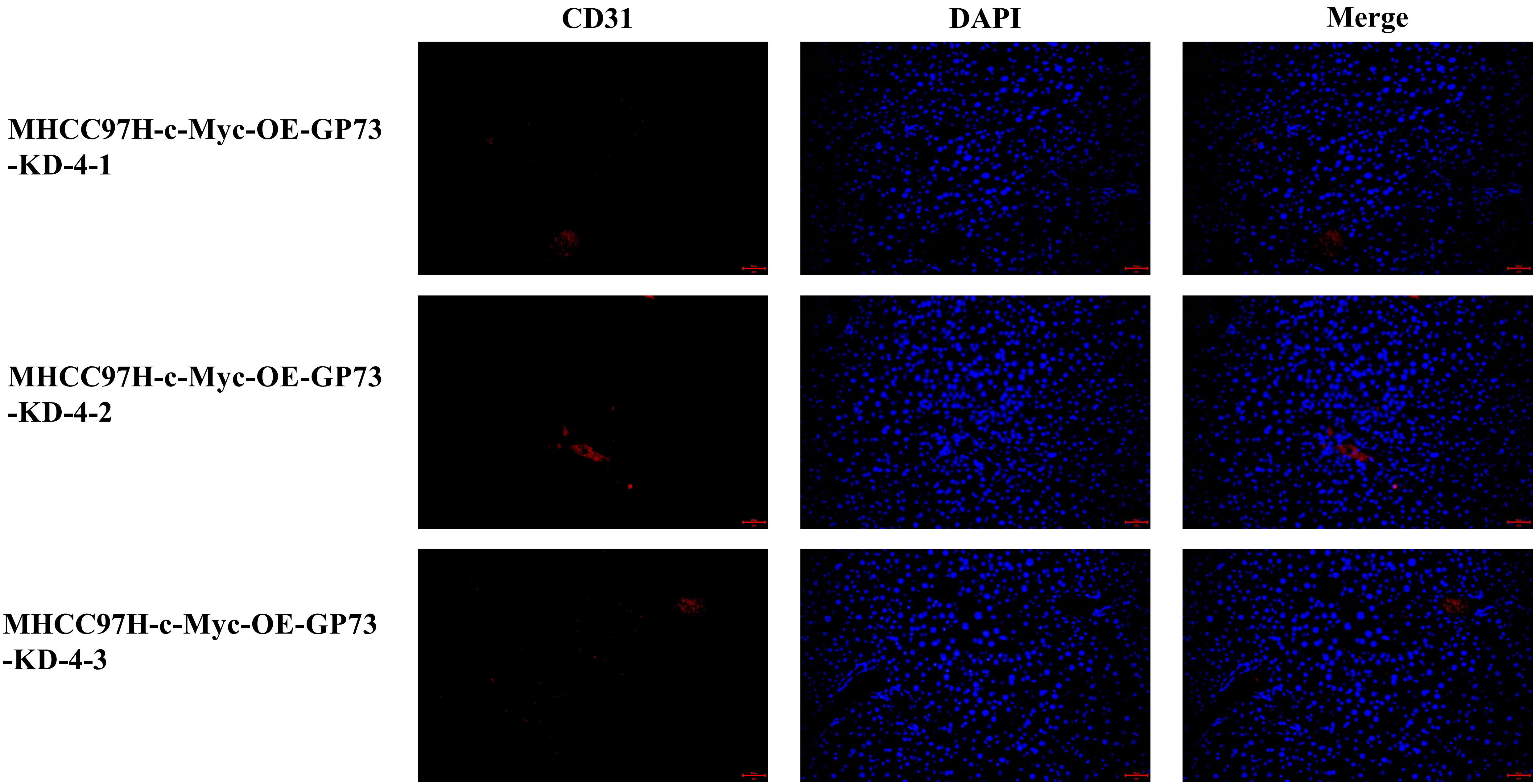
CD31
DAPI
Merge
MHCC97H-c-Myc-OE-GP73-KD-4-1
MHCC97H-c-Myc-OE-GP73-KD-4-2
MHCC97H-c-Myc-OE-GP73-KD-4-3
