# Supplementary material for: Integrating Single-Cell and Spatial Transcriptomics to Uncover and Elucidate GP73-Mediated Pro-Angiogenic Regulatory Networks in Hepatocellular Carcinoma
Source: Research (Wash D C). 2024 Jun 27;7:0387. doi: 10.34133/research.0387 (PMC11208919; doi:10.34133/research.0387)
Supplement: Supplementary 1 — Supplementary Methods Supplementary Results Figs. S1 to S7 Tables S1 to S9 Files S1 to S4 [file research.0387.f1.zip › Supplement methods.docx]

**Supplementary Materials and Methods.**

**Ethics review**

This research was in accordance with the declaration of Helsinki (Code of Ethics of the World Medical Association, last updated at the 64th WMA General Assembly, Fortaleza, Brazil, October 2013) for experiments involving humans. All of the participants in the study provided written informed consent by signing the respective paperwork and keeping their anonymity and privacy rights. The ethical approvals were obtained from the Ethics Committee of Guangxi Medical University (LW2022040).

**Analysis the independent factor of tumor response to lenvatinib treatment**

The clinicpathological characteristics and serum GP73 level in 238 HCC patients treated with lenvatinib were involved in the univariate logistic regression analyses. Then the indicators with statistical significance (*p*<0.05) were further subjected to multivariate logistic regression analysis to identify the independent factor of disease control rate (DCR) of anti-angiogenesis regimen (Table S3).

**Construction and Validation of a Prognostic Nomogram**

The HCC patients treated with lenvatinib were divided into the training cohort and validation cohort according to the 1:1 matching principle. Univariate Cox regression analyses were performed to determine prognostic factors in training cohort, which with statistical significance (*p*<0.05) were included in multivariate Cox regression analysis to identify independent prognostic risk factors (*p*<0.05) (Table S4). A nomogram was established integrating the prognostic risk factors to evaluate the 6-month and 1-year survival. Discriminatory predictive performance of the nomogram was verified by time-dependent receiver operating characteristic (t-ROC) curve. Correlation between the estimated risks and actual observed risks were verified by calibration curve. Clinical practicability of nomogram was analyzed by decision curve analysis (DCA). For facilitating application of the nomogram in clinical decision, X-tile statistical software was used to calculate the optimal cut-off values of risk scores and all patients were stratified into low- and high-risk groups. Overall survival (OS) curves were assessed by the Kaplan-Meier (KM) method and compared using the log-rank test. Data analysis was performed using SPSS23.0 (IBM, New York, USA) and R software (Version 3.2.2, Institute for Statistics and Mathematics, Vienna, VIC, Austria).

**Cellular communications analysis**

R package CellChat [v1.4.0] was used to analyze the cellular communication networks from scRNA-seq data[1]. From single-cell RNA-sequencing (scRNA-seq) data, the R toolkit CellChat (https://github.com/sqjin/CellChat, accessed on May 7th, 2022) was used to quantitatively infer and evaluate the intra-/inter-cellular communication networks coordinating with special biofunctions by utilizing network analysis and pattern recognition methodologies.

**Gene set enrichment analysis**

Gene Ontology (GO) enrichment analyses were conducted using a “clusterProfiler” package (v3.16.1) in R[2]. Heatmap indicated each GO term’s significance (-log_10_ *P* value). Angiogenesis gene signature Gene Set EnrichmentAnalysis (GSEA) analysis was performed through the GSEA functions in clusterProfiler package[2]. The complete GSEA results have been supplied and detailed in the Supplemental file 2.

**Sankey Diagram Plotting**

Using single cell-spatial transcriptomics data to analyze the correlations among GP73, c-Myc, Lactylation, JAK-STAT, and ERS, we selected the top15 related genes among them and plotted a Sankey diagram using the R package network 3D to visualize the co‐expression network[3].

**RNA extraction, reverse transcription and Quantitative Real-Time PCR (qRT-PCR)**

Total RNA was extracted from tissues and cells using the TRIzol reagent (11668019, Invitrogen, France). Complementary DNA (cDNA) was synthesized using Primescript RT Reagent (RR037B, TaKaRa, Japan) and the resulting products was amplified using SYBR® Green PCR Supermix kit (Q111-02/03, Vazyme, Chins) in a real-time fluorescent quantitative PCR instrument. qPCR primer sequences are detailed in Table S5. qRT-PCR for cDNA samples were performed on StepOnePlus Real-Time PCR system (4376600, Thermo Fisher, USA) using FastStart Universal SYBR Green Master Mix (4913850001, Roche, Switzerland) following manufacturer’s instructions. Relative mRNA expressions were evaluated using the comparative threshold cycle (Ct) method. Quantification was normalized using β-actin as the internal control and 2-∆∆Ct method was used as a relative quantification for qPCR data analysis.

**Western Blotting**

Cells were lysed in RIPA lysis buffer and proteins (10-30µg) were separated on 8-10% SDS/PAGE gel and transferred onto PVDF membranes (IPVH00010, Millipore, MA), then incubated with primary antibodies and HRP-conjugated secondary antibodies. Band intensities were observed using an enhanced chemiluminescence kit (20148, Thermo Fisher, USA) and quantified using densitometric analysis with β-actin as the loading control, then imaged in Java (ImageJ, Version 1.38, National Institutes of Health). Quantitative analysis of Western blot was performed by using the ImageJ software following protocols. Antibodies used are listed in Table S6. The raw data of protein expression has been uploaded. Please check the Supplemental file 3.

**Plasmids transfection and establishment of stable cell lines**

Transfer plasmids (PHY-022 and pLKO.1) and lentivirus plasmids (packaging plasmids psPAX2 and envelope plasmids pMD2.G) were provided by Hanyin Co. (Shanghai, China). The GP73, STAT3, cMYC, GRP78 over-expression (OE) and negative control (NC) sequences were cloned into transfer plasmids PHY-022 and lentivirus plasmids in 293T cells. The GP73, JAK2, STAT3, cMYC, LDHA, LDHB, P300 knockdown (KD) and negative control (NC) sequences were cloned into transfer plasmidsp LKO.1 and lentivirus plasmids in 293T cells. Correct clones were verified by sequencing (Hanyin Co., Shanghai, China). Then MHCC97H and Hep3B cells were infected with virus and polybrene in six-well plates the following day. Positive clones were selected with puromycin for 3-4 weeks to establish stable cells. Over-expression and knock-down efficiencies were verified by qRT-PCR and western blot. Primers used are listed in Table S7.

**Cell counting kit-8 (CCK-8) assay**

Cell proliferation was assessed using a Cell Counting Kit-8 kit (CCK-8, CK04, Dojindo Chemical Laboratory, Japan) following manufacturer’s protocol. HUVECs were diluted in serum-free medium and seeded in 96-well cell culture plates at 2000 cells/well. Equal volume of fresh medium containing 10% CCK-8 reagent was replaced at 24, 48, 72 and 96 h; then cells was incubated at 37°C for 3 hours for the cells growth rates determination. The absorbance was finally measured at 450nm using a microplate reader (5082; Grodig, Austria).

**Transwell migration assay**

Cells migration was evaluated using BD BioCoat™ Matrigel™ chambers (8μm pore size, 356234, Corning, USA) following manufacturer’s protocol. Cell suspensions without serum at a density of 5×10^4^ cells/well were added to the upper chamber, and 500μl medium containing 10% fetal bovine serum was added to the lower chamber. After incubation at 37°C for 48 hours, cells did not pass through the pore were gently removed with a cotton swab, the remaining cells in the lower chamber were fixed with 4% paraformaldehyde for 5 minutes and stained with 1% crystal violet for 30 minutes. The stained cells were washed three times with PBS, and then counted under an optical microscope (LEXT OLS4100, Olympus Corporation, Japan) and analyzed using the ImageJ (Version 1.38, National Institutes of Health) software.

**Tubule formation assay**

HUVECs were seeded at a density of 2×10^4^ in 96-well plates coated with 60μL Matrigel (BD356234; Corning, USA). After incubation at 37°C and 5% CO_2_ for 12 hours, the medium was removed and the cells were washed three times with PBS then fixed with 4% paraformaldehyde at room temperature for 15 minutes following images capture. The capillary-like branches was captured in 5 random microscopic fields with a computer-assisted microscope, and quantified using Image Pro‐Plus software (Media Cybernetics, Rockville, MD, USA) for evaluate the angiogenic ability of HUVECs.

***In vivo* Bioluminescence Imaging**

Mice were anesthetized with isoflurane and imaged at 24 hours after injection of DiR liposomes using the specific Excitation (EX) and Emission (EM) wavelengths with DiR (EX wavelengths:710-760nm; EM wavelengths:810-875nm). Bioluminescence images were captured and circular regions of interest (ROI) were calculated using Living Image v4.2 software (Perkin Elmer). An empty background was subtracted from the total signal, the *in vivo* bioluminescence activity were quantified by total photon flux within an ROI per second.

**Immunohistochemical Staining**

Resected tumor tissues were fixed with 10% formaldehyde, embedded in paraffin, and cut into 3-5μm sections placed on slides. Hematoxylin & Eosin (H&E) staining was done for histopathologcal examination. After deparaffinization, rehydration, and antigen retrieval by microwave procedures, the slides were incubated with diluted (1:500) primary antibodies at 4℃ overnight, and then incubated with IgG secondary antibodies (1:2500, Promega, USA) at room temperature for 30 minutes followed proceeding with the chromogen DAB substrate and hematoxylin staining. The percentage of positive cells was scored as follows: 0 (<5%), 1 (5–25%), 2 (26–50%), 3 (51–75%), and 4 (>75%). The staining intensity was scored as 0, 1, 2 and 3 points for no staining, light yellow, brownish yellow, and brown, respectively. Finally, the scores representing the proportion of positive cells and staining intensity were multiplied to determine the degree of IHC score[4] as follows: 1-4 points indicated weakly positive (+), 5-8 points indicated moderately positive (++), and ≥9 points indicated strongly positive (+++). Antibodies used are listed in Table S8.

**Cell Immunofluorescence**

Cells cultured in 24-well plates approximately 70% confluence was transfered onto cell slides, washed three times with pre-chilled PBS and fixed with 100% methanol at -20 °C overnight. Next, cells were fixed with 100% acetone at -20 °C for 1 minute and blocked with 5% bovine serum albumin (BSA) at room temperature for 1 hour, then incubated with primary antibodies at 4℃ overnight. Then, after three washes with PBS the cells were incubated with fluorescently- conjugated secondary antibodies at room temperature for 1 hour and nuclei were stained with DAPI at room temperature for 20 minutes. Images capture were used a DM2000 Leica microscope equipped with the LAS AF software (version 2.6.0.7266, Leica). Quantitative analysis of cell immunofluorescence was performed by using the ImageJ (Version 1.38, National Institutes of Health). Antibodies used are listed in Table S9. The images raw data have been supplied in the Supplemental file 4.

**Predicted binding sites from JASPAR database**

Promoter sequence of the GP73 gene FASTA format was downloaded from Gene module of the NCBI online tool (https://www.ncbi.nlm.nih.gov/pubmed/) and the binding sites between MYC and GP73 promoter were analyzed using the JASPAR website (https://jaspar.genereg.net/, accessed on May 7^th^, 2022) with a matching score threshold set to 500. Data were output after calculation through the JASPAR website.

**Angiogenesis qPCR Array**

RNA extraction and cDNA synthesis methods were as described above in qRT-PCR. An angiogenesis qPCR array containing 21 human key angiogenic genes were used for the detection of differential expressions of angiogenic genes by RT2 SYBR Green ROX qPCR Mastermix (330520, QIAGEN, Germany) following manufacturer's instructions. In 96-well plates, RNA extraction products was added specific primers of the 21 angiogenic genes and incubated at 95°C for 10 minutes. Then PCR reactions was performed on an ABI QuantStudio 12K Flex Sequence Detection System (QuantStudio® 12K Flex, Applied Biosystems, USA) with 40 cycles (95°C for 15 seconds, 60°C for 1 minute). Raw data were analyzed using an online Superarray Data Analysis Web Portal tool.

**Molecular docking determination**

The National Center for Biotechnology Information (NCBI, <https://www.ncbi.nlm.nih.gov/>, accessed on May 5th, 2023) provided GP73 and STAT3 RNA sequence for download. The RNAfold web server (<http://rna.tbi.univie.ac.at/cgibin/RNAWebSuite/RNAfold.cgi>, accessed on May 5th, 2023) were used for the prediction of RNA secondary structures. The 3D structure of GP73 and STAT3 proteins were extracted by using the Protein Data Bank (PDB, <https://www.rcsb.org/pages/contactus,> accessed on May 5th, 2023) and imported into AutoDock (version 4.2.6)[5] software for the determination of molecular docking between receptors and ligands. The binding ability and stability of active ingredients were evaluated by docking scores and visualized using a PyMOL (version 2.5) software[5].

**Co-immunoprecipitation (co-IP)**

In 6cm cell dishes, cells were cultured to approximately 70% confluence. After three washes with precooled PBS the cells were incubated on ice with immunoprecipitation buffer containing protease inhibitor for 30 minutes. Supernatant was obtained by removing cell fragments and nuclei by 12000rpm centrifugation for 20 minutes. A quarter of supernatant was used in Input group and the remaining was equally divided into IgG and Flag groups, then 0.5μg IgG and Flag antibodies were added and shaken slowly with uniform velocity at 4℃ for 12 hours. Then, 20μL protein A/G agarose magnetic beads were added to the immune complex. The mixture was slowly shaken for 2 hours then washed 3-5 times by PBS buffer, followed by the addition of 2X loading buffer and boiled for western blotting detection.

**Dual-Luciferase Reporter Assay**

Luciferase reporter assays were performed using the Dual Luciferase Reporter Assay Kit (DL101-01, Vazyme, China) following manufacturer's protocol. In six-well plates, HEK-293T cells were cultured to approximately 70% confluence and then co-transfected with the STAT3-WT, STAT3-MUT reporter plasmids separately together with GP73-NC, GP73-OE mimics. Twenty-four hours after transfection, firefly and Renilla luciferase activities were measured by a Dual-Luciferase Reporter Assay System (E1910, Promega, USA). To obtain p4xM67-tk-Luc, an oligonucleotide containing four copies of the sequence GGTTCCCGTAAATGCATCA (TTCCCGTAA is the STAT-binding site) was introduced in the AccI-BamHI sites of pTATA-tk-Luc upstream of the minimal promoter[6]. Diagnostic digest is EcoRI (3.6kb, 1.7kb, 0.6kb).4xM67 pTATA TK-Luc was a gift from Jim Darnell (Addgene plasmid # 8688; http://n2t.net/addgene:8688; RRID: Addgene #8688).

**Reference**

1. Jin S, Guerrero-Juarez CF, Zhang L, Chang I, Ramos R, Kuan C-H et al. Inference and analysis of cell-cell communication using CellChat. Nat Commun. 2021;12:1088.

2. Wu T, Hu E, Xu S, Chen M, Guo P, Dai Z et al. clusterProfiler 4.0: A universal enrichment tool for interpreting omics data. Innovation (Camb). 2021;2:100141.

3. Glover RE, Al-Haboubi M, Petticrew MP, Eastmure E, Peacock SJ, Mays N. Sankey diagrams can clarify ‘evidence attrition’: A systematic review and meta-analysis of the effectiveness of rapid diagnostic tests for antimicrobial resistance. J Clin Epidemiol. 2022;144:173–84.

4. Fedchenko N, Reifenrath J. Different approaches for interpretation and reporting of immunohistochemistry analysis results in the bone tissue - a review. Diagn Pathol. 2014;9:221.

5. Sava A, Buron F, Routier S, Panainte A, Bibire N, Profire L. New nitric oxide-releasing indomethacin derivatives with 1,3-thiazolidine-4-one scaffold: Design, synthesis, in silico and in vitro studies. Biomed Pharmacother. 2021;139:111678.

6. Besser D, Bromberg JF, Darnell JE, Hanafusa H. A single amino acid substitution in the v-Eyk intracellular domain results in activation of Stat3 and enhances cellular transformation. Mol Cell Biol. 1999;19:1401–9.
