# Supplementary material for: Integrating Single-Cell and Spatial Transcriptomics to Uncover and Elucidate GP73-Mediated Pro-Angiogenic Regulatory Networks in Hepatocellular Carcinoma
Source: Research (Wash D C). 2024 Jun 27;7:0387. doi: 10.34133/research.0387 (PMC11208919; doi:10.34133/research.0387)
Supplement: Supplementary 1 — Supplementary Methods Supplementary Results Figs. S1 to S7 Tables S1 to S9 Files S1 to S4 [file research.0387.f1.zip › Supplement Result.docx]

**Supplement Result**

**1. Construction and validation of a prognostic nomogram for patients with HCC treated with anti-angiogenesis therapy**

A total of 238 patients with HCC treated with lenvatinib were divided into training and validation cohorts according to the 1:1 random matching principle. All baseline variables between the two cohorts showed no significant differences (*P* > 0.05, Table 1). Cox univariate regression analysis was used to determine the prognostic factors of OS in the training cohort (Table S4), which were statistically significant (*P* < 0.05), and were included in a multivariate Cox regression analysis to identify independent prognostic risk factors. As a result, BCLC-C disease, combined immune checkpoint inhibitors therapy, serum GP73 level ≥178 ng/mL were independent prognostic indicators of OS (*P* < 0.05 for all, Table S4), which showed significant differences in OS evaluation (Figure S1A–C) and integrated to establish a prognostic nomogram (Figure S1D). The nomogram showed a good predictive efficacy with the AUC values for predicting 6-month and 1-year survival rates were 0.785 (95%CI: 0.652-0.917) and 0.739 (95%CI: 0.620-0.859) in training cohort, 0.856 (95%CI: 0.780-0.932) and 0.857 (95%CI: 0.783-0.930) in validation cohort (Figure S1E–F). The calibration curves showed that the estimated values of 6-month and 1-year survival were in satisfactory agreement with the actual observations in both the training and validation cohorts (Figure S1G–-J). Additionally, the DCA plot showed good clinical applicability of the nomogram, with a significant net benefit and threshold probability in predicting 6-month and 1-year survival (Figure S1K). Then, based on the optimal cutoff values of prediction the risk stratification were sort into low risk group < 2.27 and high risk group ≥ 2.27. This stratification showed significant differences in OS between the two risk groups (*P* < 0.001), proving that the nomogram had a good clinical decision-making ability (Figure S1L).

2. **Single-cell transcriptomics from GEO database implied the potential pro-angiogenic roles of GP73 in HCC**

We used the cBioPortal database to explore the genomic characteristics of GP73 in 372 HCC patients and found that the genetic alteration rate of GP73 was approximately 5.10% (19/372), among which high mRNA accounted for 4.56% (17/372), structural variants and amplification accounted for 0.27% (1/372), and missense mutations accounted for approximately 0.27% (1/372), indicating that GP73 rarely presents mutations at the genomic level (Figure S2G). Fourty-eight HCC single-cell datasets and five normal liver single-cell datasets from the GEO database were utilized for validation, and through a UMAP 10 types of cells were visualized identified (Figure S2H), among which malignant cells (SERPINA1, GPC3, AFP) accounted for the highest proportion, and abundant endothelial cells (SPARCL1, CDH5, VWF) were captured (Figure S2I). We found that GP73 was over-expressed in HCC and vascular endothelial cells (Figure S2J). HCC cells were then isolated and subdivided into 17 subpopulations (Figure S2K), among which GP73 expression was significantly elevated in HCC cell subclusters C5, C3, and C17 (Figure S2L). Interestingly, we found that elevated GP73 expression correlated with increased expressions of the pro-angiogenic ligands VEGFA, SPP1, CXCL2, ANGPTL4, and ANGPTL3 in all HCC cell subclusters (Figure S2M). A heatmap was then visualized showing the inter-cellular communications between over-expressed GP73 HCC cell subclusters and vascular endothelial cells through multiple pro-angiogenic receptor-ligand interactions, including CXCL5-ACKR1, VEGFA-VEGFR1/R2, TGFB1-(TGFBR1+TGFBR2), SPP1-(ITGAV+ITGB1), and ANGPTL4-CDH5 (Figure S2N). The results of validation using the HCC single-cell samples from GEO databases were consistent with our single-cell transcriptomics analysis of the HCC tissues, supporting GP73 as a novel angiogenesis niche gene with anti-angiogenic potential.
